# Dissecting the role of cancer‐associated fibroblast‐derived biglycan as a potential therapeutic target in immunotherapy resistance: A tumor bulk and single‐cell transcriptomic study

**DOI:** 10.1002/ctm2.1189

**Published:** 2023-02-11

**Authors:** Shaoquan Zheng, Jie‐Ying Liang, Yuhui Tang, Jindong Xie, Yutian Zou, Anli Yang, Nan Shao, Xiaying Kuang, Fei Ji, Xuefeng Liu, Wenwen Tian, Weikai Xiao, Ying Lin

**Affiliations:** ^1^ Department of Breast Surgery Breast Disease Center, The First Affiliated Hospital Sun Yat‐sen University Guangzhou China; ^2^ State Key Laboratory of Oncology in South China, Collaborative Innovation Center for Cancer Medicine Sun Yat‐sen University Cancer Center Guangzhou China; ^3^ Guangdong Provincial Key Laboratory of Malignant Tumor Epigenetics and Gene Regulation, Department of Medical Oncology, Sun Yat‐sen Memorial Hospital Sun Yat‐sen University Guangzhou China; ^4^ Department of Breast Oncology Sun Yat‐sen University Cancer Center Guangzhou China; ^5^ Department of Breast, Guangdong Provincial People's Hospital (Guangdong Academy of Medical Sciences) Southern Medical University Guangzhou China; ^6^ Department of Pathology, Guangdong Provincial People's Hospital (Guangdong Academy of Medical Sciences) Southern Medical University Guangzhou China

**Keywords:** cancer‐associated fibroblasts, immunotherapy, tumor biomarker, tumor microenvironment

## Abstract

**Introduction:**

Cancer‐associated fibroblasts (CAFs) are correlated with the immunotherapy response. However, the culprits that link CAFs to immunotherapy resistance are still rarely investigated in real‐world studies.

**Objectives:**

This study aims to systematically assess the landscape of fibroblasts in cancer patients by combining single‐cell and bulk profiling data from pan‐cancer cohorts. We further sought to decipher the expression, survival predictive value and association with immunotherapy response of biglycan (BGN), a proteoglycan in the extracellular matrix, in multiple cohorts.

**Methods:**

Pan‐cancer tumor bulks and 27 single‐cell RNA sequencing cohorts were enrolled to investigate the correlations and crosstalk between CAFs and tumor or immune cells. Specific secreting factors of CAFs were then identified by expression profiling at tissue microdissection, isolated primary fibroblasts and single‐cell level. The role of BGN was further dissected in additional three bulk and five single‐cell profiling datasets from immunotherapy cohorts and validated in real‐world patients who have received PD‐1 blockade using immunohistochemistry and immunofluorescence.

**Results:**

CAFs were closely correlated with immune components. Frequent crosstalk between CAFs and other cells was revealed by the CellChat analysis. Single‐cell regulatory network inference and clustering identified common and distinct regulators for CAFs across cancers. The BGN was determined to be a specific secreting factor of CAFs. The BGN served as an unfavourable indicator for overall survival and immunotherapy response. In the real‐world immunotherapy cohort, patients with high BGN levels presented a higher proportion of poor response compared with those with low BGN (46.7% vs. 11.8%) and a lower level of infiltrating CD8+ T cells was also observed.

**Conclusions:**

We highlighted the importance of CAFs in the tumor microenvironment and revealed that the BGN, which is mainly derived from CAFs, may be applicable in clinical practice and serve as a therapeutic target in immunotherapy resistance.

## Graphical abstract

- There are tight correlations between CAFs and immune cells in pan‐cancer bulk cohort.
- Single‐cell data reveals regulators for CAFs and frequent crosstalk with other cells.
- In various cancers, stromal biglycan is dominantly derived from CAFs.
- Biglycan is an unfavourable indicator for survival and immunotherapy response in cancer.

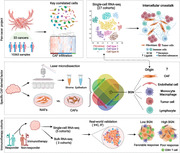

**Abbreviations**
BGN: biglycan
CAFs: cancer‐associated fibroblasts
ccRCC: clear cell renal cell carcinoma
CIBERSORT: cell‐type Identification By Estimating Relative Subsets Of RNA Transcripts
cMAP: The Connectivity Map
DEG: differentially expressed gene
ECM: extracellular matrix
EMT: epithelial–mesenchymal transition
EPIC: Estimate the Proportion of Immune and Cancer cells
ESTIMATE: Estimation of STromal and Immune cells in MAlignant Tumor tissues using Expression data
FPKM: Fragments per kilobase per million mapped fragments
GEO: Gene Expression Omnibus
GTEx: Genotype‐Tissue Expression Project
GO: Gene Ontology
HER2: human epidermal growth factor receptor 2
HR: hazard ratio
iCAFs: inflammatory cancer‐associated fibroblasts
ICB: immune checkpoint blockade
IF: immunofluorescence
IHC: immunohistochemistry
IRG: immune‐related gene
MCP‐counter: microenvironment cell populations‐counter
MP: Miller–Payne
myCAFs: myofibroblastic cancer‐associated fibroblasts
OS: overall survival
PCA: principal component analysis
PR: progesterone receptor
SCENIC: single‐cell regulatory network inference and clustering
scRNA‐seq: Single‐cell RNA sequencing
TCGA: The Cancer Genome Atlas
TME: tumor microenvironment
TNBC: triple‐negative breast cancer
TPM: transcripts per million
α‐SMA: α‐smooth muscle actin

## 1 INTRODUCTION

Cancer has long been recognized as a devastating disease. Global cancer statistics estimated that almost 10.0 million cancer deaths and 19.3 million new cancer cases occurred in 2020.^1^ Cancer does not merely consist of cancer cells. It also comprises stromal and immune cells, which serve as ‘soil’ for the ‘seeds’, namely, cancer cells. Together, these factors constitute a complex tumor microenvironment (TME).^2^ Researchers have long recognized the similarities between tumor stroma generation and wound healing, in which fibroblasts play a significant role.^3^ Cancer‐associated fibroblasts (CAFs) are a crucial part of stromal cells, and they exert their functions by affecting cancer and immune cells in the TME. CAFs are involved in extracellular matrix (ECM) deposition, which mechanically leads to the invasion of cancer cells, secretion of factors that promote cancer progression and antigen presentation.^4^

CAFs are highly heterogeneous and can be divided into different subpopulations with distinct biological functions, and they are labelled by specific markers. Hence, CAFs exert both pro‐tumor and anti‐tumor functions in different studies. CAFs are generally divided into myofibroblastic (myCAFs) and inflammatory (iCAFs) CAFs. The former is characterized by a high level of α‐smooth muscle actin (α‐SMA), whereas the latter exhibits abundant chemokine and cytokine production without myofibroblastic features.^5^ Recently, antigen‐presenting CAFs have been identified, which might play a vital role in immune modulation.^6^ Moreover, different CAFs subsets have been discovered in different cancers, indicating diverse driving factors for CAFs development in cancers.^7^, ^8^, ^9^, ^10^ Currently, CAFs are considered as therapeutic targets for cancer.^11^, ^12^ However, clinical trials of different fibroblast‐related targets in cancer have faced substantial challenges.^13^ Therefore, a comprehensive understanding of the role of CAFs in the TME and the diverse regulators of CAFs development is essential for optimizing therapeutic strategies.

Although clinical trials have demonstrated that immunotherapies are effective in different cohorts of patients, the mechanism of primary resistance remains poorly understood.^14^ Recently, evidence has begun to show that specific CAFs subpopulations might contribute to immunotherapy resistance. Patients with the fibrotic subtype exhibit immunosuppressive features and showed lower response to treatment than those with immune‐enriched/nonfibrotic signatures.^15^ In human ovarian and breast cancers, four CAFs subpopulations (CAF‐S1–S4) were identified. CAF‐S1 (FAP^hi^CD29^med‐hi^SMA^hi^) is the only subpopulation characterised by immunosuppressive features in cancer. Further clustering of CAF‐S1 revealed eight subclusters. These subclusters included cluster 0/ecm‐myCAFs, which showed the up‐regulation of the CTLA4/PD‐1 protein in regulatory T cells and the enhancement of the cluster 3/TGFβ‐myCAFs.^16^ Moreover, LRRC15+ CAFs promote immunosuppression and correlated with therapeutic resistance to immune checkpoint blockade (ICB) in multiple cancers.^17^ Together, these results imply the crucial role of CAFs, especially specific subclusters, in predicting or determining immunotherapy response. Deciphering fibroblast‐immune microenvironment interactions can enhance our understanding of primary resistance and immunotherapeutic strategies.

Single‐cell RNA sequencing (scRNA‐seq) has been developed in recent years to facilitate gene profiling analysis at the single‐cell level, providing an opportunity to clarify the heterogeneity of the tumor bulk and identify novel potential biomarkers and therapeutic targets. Herein, we sought to systematically assess the landscape of fibroblasts in patients with cancer by combining single‐cell and bulk profiling data from pan‐cancer cohorts. First, our analyses revealed the relative abundance of fibroblasts in 33 cancers and their intercellular interactions with malignant and immune cells, including secreted signalling, ECM–receptor and cell–cell contacts. Next, we explored the heterogeneity and homogeneity of CAFs in the transcriptional regulation of single‐cell clusters and provided insights into the mechanisms underlying CAFs development. We further identified biglycan (BGN), an extracellular protein, as a biomarker for CAFs and immunotherapy response. Finally, the expression, predictive value for survival and association with immunotherapy response to BGN were validated in multiple cohorts.

## 2 MATERIALS AND METHODS

### 2.1 Datasets

Transcriptome data of the bulk tumor were retrieved from Genotype‐Tissue Expression Project (GTEx), The Cancer Genome Atlas (TCGA) project and Gene Expression Omnibus (GEO) databases. A total of 10 363 tumor samples from 33 types of cancers that were pathologically diagnosed and had available RNA expression data were included in the TCGA project. The detailed constituent ratios and corresponding abbreviations in TCGA pan‐cancer cohort are shown in Figures S1a and S1b. The TCGA dataset were collected and analyzed using the TCGAbiolinks package in the R software.^18^ Pan‐cancer TCGA RNA count data were downloaded from the GDC Portal using the GDCdownload, GDCprepare and GDCquery functions. Transcripts per million (TPM) were calculated at the transcript level. Ensemble IDs were annotated and transformed into gene symbols using the Homo_sapiens.GRCh38.100. chr.gtf information, and the average expression value of the duplicated genes was calculated. 33 cancers were further classified into different groups according to pathology and cell origin: adenocarcinomas, kidney (kidney), lympho‐hematopoietic (Ly‐Hem), neuronal lineage (Neuronal), sarcomas (Sar), squamous cell carcinomas (Squamous), stem cell‐like tumor (SC) and those that do not belong to any of these groups (Misc).^19^ Triple‐negative breast cancer (TNBC) was defined in patients with negative progesterone receptor, estrogen receptor and human epidermal growth factor receptor 2 by using immunohistochemistry (IHC). Patients with esophageal carcinoma (ESCA) were further divided into ESCA‐Ad (adenocarcinoma) and ESCA‐Sq (squamous) groups according to the pathological results.

For survival analysis, those without active follow‐up information were excluded. Overall survival (OS) was calculated from the date of diagnosis to the date of last follow‐up or death. The mutation profiles of TP53, BRAF, EGFR and KRAS were obtained, and silent mutations were further removed in the TCGA dataset.

Fragments per kilobase per million mapped fragments (FPKM) data from the GTEx datasets were obtained from the Xena website (https://xenabrowser.net/) and transformed into TPM. The corresponding FPKM of the TCGA cohort was also obtained and transformed. The cohorts were further merged, and cancers with < 5 paired normal tissues were excluded.

Microdissection datasets of the epithelium and corresponding stroma from the lung (GSE22863), pancreas (GSE93326), breast (GSE10797), colon (GSE35602) and ovary (GSE38666) were downloaded from the GEO database (https://www.ncbi.nlm.nih.gov/geo/).^20^, ^21^, ^22^, ^23^, ^24^ To compare the mRNA expression profiles of normal fibroblasts and CAFs, mRNA microarrays of cultured fibroblasts from the breast (GSE29270), colon (GSE46824), lung (GSE22862) and pancreas (GSE21440) were obtained (Table S1).^24^, ^25^, ^26^, ^27^ Probe names were mapped according to the corresponding annotation files. The expression matrices were normalized using the limma package in R software if necessary, and the average expression value of duplicated gene names was calculated.^28^

### 2.2 Sample collection, IHC and immunofluorescence

Breast cancer patients who received neoadjuvant PD‐1 blockade plus chemotherapy, followed by surgery, were retrospectively identified in Guangdong Provincial People's Hospital from 1 January 2020 to 31 October 2021. A total of 32 patients were enrolled in the study. The surgically removed cancer specimens were collected. The response to therapy was evaluated using magnetic resonance imaging (MRI)and the Miller–Payne (MP) criteria. Patients with MP G4‐5 were classified into pathological responders and G1‐3 were classified as non‐responders.

IHC and immunofluorescence (IF) analyses were performed as previously described.^29^, ^30^ Briefly, the sections were deparaffinised and rehydrated using xylene and graded ethanol. Endogenous peroxidase activity was blocked using 3% hydrogen peroxide for 20 min at room temperature. Antigen retrieval was performed in a pressure cooker using a citrate buffer (CWBIO). Goat serum was used to block nonspecific binding to the sections for 1 h at room temperature (Solarbio). The slides were further incubated with antibodies against BGN (1:150; Proteintech) and CD8 (1:100; Biolegend) at 4°C overnight. Centrifuge tubes were obtained from NEST Biotechnology. For IHC, EnVision Detection Systems (Dako) were used to detect immunoreactivity. For IF, the slides were further incubated with Alexa Fluor 594 or Alexa Fluor 488 (1:2000; Thermo Fisher) secondary antibodies and observed using a ZEISS LSM880 microscope.

### 2.3 Marker profiling

Specific CAFs markers have been commonly used to estimate the relative fibroblast abundance in cancer.^8^, ^31^, ^32^, ^33^ Other CAFs markers were used to evaluate the activities of inflammatory and myofibroblastic CAFs (Table S2).^6^

Epithelial–mesenchymal transition (EMT) markers were employed to show the correlation between fibroblasts and metastatic potential (Table S3).^34^

Immune‐related genes (IRGs) were also included in the analysis, including the activation of immune receptors, IFN‐γ signatures, immune modulators, inhibitory immune receptors/ligands and myeloid lineage‐related genes. More detailed IRGs, including receptors, ligands, co‐stimulators, co‐inhibitors, antigen presentation and cell adhesion, were also analyzed (Table S4).^15^, ^35^

### 2.4 TME estimation

The stromal and immune scores were calculated to evaluate the immune and stromal components in the tumor bulk using the ‘Estimation of STromal and Immune cells in MAlignant Tumor tissues using the Expression data’ (ESTIMATE) method.^36^ The proportion of 22 tumor‐infiltrating immune cells was estimated by Cell‐type Identification By Estimating Relative Subsets Of RNA Transcripts (CIBERSORT).^37^ CAFs were estimated using three algorithms: ‘Estimate the Proportion of Immune and Cancer cells’ (EPIC), ‘Microenvironment Cell Populations‐counter’ (MCP‐counter) and xCell.^38^, ^39^, ^40^ Comprehensive analysis of TCGA cohorts using the above methods was performed with Timer 2.0 (http://timer.cistrome.org/) (Table S5).^41^ In a previous study, the TCGA cohort was divided into different model‐based immune subtypes: wound healing C1, IFN‐γ‐dominant C2, inflammatory C3, lymphocyte‐depleted C4, immunologically quiet C5 and TGF‐β‐dominant C6. The immune subtypes of TCGA pan‐cancer cohort were downloaded from Xena (Table S6, https://xenabrowser.net).^42^

### 2.5 Pan‐cancer clustering

To estimate the relative fibroblast abundance in different cancers, samples from 33 cancers were clustered according to the expression of the commonly used CAFs markers (ACTA2, PDGFRB, THY1, FAP, COL1A1, PDGFRA and PDPN). The ComplexHeatmap package was used for pan‐cancer sample clustering.^43^ The dendrogram tree was further divided into three CAFs infiltration groups (high, medium and low). Principal component analysis (PCA) was performed to determine the clustering results. The ESTIMATE scores and survival outcomes were compared between the groups for each cancer.

### 2.6 Single‐cell sequencing analysis

14 single‐cell sequencing datasets were collected as follows: basal cell carcinoma (BCC_GSE123813), bladder urothelial carcinoma (BLCA_GSE130001), breast cancer (BRCA_GSE114727), cholangiocarcinoma (CHOL_GSE125449), colorectal cancer (CRC_GSE146771), liver hepatocellular carcinoma (LIHC_GSE125449), kidney renal clear cell carcinoma (KIRC_GSE111360), head and neck squamous cell carcinoma (HNSC_GSE103322), neuroendocrine tumor (NET_GSE140312), stomach adenocarcinoma (STAD_GSE134520), skin cutaneous melanoma (SKCM_GSE123139), pancreatic adenocarcinoma (PAAD_CRA001160), ovarian serous cystadenocarcinoma (OV_GSE118828) and non‐small cell lung cancer (NSCLC_GSE131907) (Table S7).^44^, ^45^, ^46^, ^47^, ^48^, ^49^, ^50^, ^51^, ^52^ The global‐scaling normalization method was applied, and the cells were clustered using the uniform manifold approximation and projection method and annotated.

Tumor bulk‐profiling cohorts (GSE176307, GSE135222 and IMvigor210) and three scRNA‐seq datasets reporting the response to immunotherapy were also obtained, following the methods adopted by previous studies (Table S8).^16^, ^17^, ^53^, ^54^, ^55^, ^56^, ^57^ Detailed information regarding the response to immunotherapy is presented in Table S9.

To analyze the cell–cell interactions, the CellChat method was employed using the CellChat package (https://github.com/sqjin/CellChat).^58^ CellChat is an algorithm designed to predict the major incoming and outgoing intercellular communication networks. It contains a ligand–receptor interaction database based on the literature and Kyoto Encyclopedia of Genes and Genomes. CellChatDB is composed of 229 signalling families, including TGF‐β, BMP, EGF, chemokines and cytokines. Fibroblasts and myofibroblasts were assigned as CAFs prior to analysis. Secretion signalling, cell–cell contacts and ECM–receptor signalling in CAFs were analysed. The interaction strength of intercellular signalling was quantified and displayed within a single cancer.

The single‐cell regulatory network inference and clustering (SCENIC) algorithm was used to analyze cis‐regulation and identify specific transcription factors.^59^ During the SCENIC procedures, GENIE3 was used to identify genes that were co‐expressed with transcription factors, and RcisTarget was further applied to perform cis‐regulatory motif analysis. Finally, modules with significant motifs of upstream regulators were identified and named as regulons. The activity of every regulon in each cell type was quantified using the AUCell score, which indicates the regulatory network that drives cellular heterogeneity. In this part, SCENIC algorithm was applied to BLCA_GSE130001, BRCA_GSE114727, CHOL/LIHC_GSE125449, CRC_GSE146771, HNSC_GSE103322, NET_GSE140312, OV_GSE118828, PAAD_CRA001160, STAD_GSE134520, SKCM_GSE123139, KIRC_GSE111360, NSCLC_GSE131907 and BCC_GSE123813. SCENIC was performed using the SCENIC package in R software (https://scenic.aertslab.org/) and pySCENIC in Python 3.8.5 (https://github.com/aertslab/pySCENIC).

### 2.7 Functional analysis

The ggraph and circlize packages were used for the TME network analysis and display.^60^ The clusterProfiler package was used for pan‐cancer pathway enrichment.^61^ For each cancer, the samples were classified into two groups on the basis of the median value of BGN expression. Differentially expressed genes (DEGs) were analyzed using the edgeR package, and DEGs were further filtered.^62^, ^63^ DEGs were further annotated using Gene Ontology (GO) terms, and key significantly enriched signalling pathways were identified.

### 2.8 Connectivity Map

The Connectivity Map (cMAP) built 02 of the Broad Institute (https://portals.broadinstitute.org/cmap/) was used to identify the target drugs that may affect CAFs.^64^ This online tool was designed to evaluate the potential ability of compounds to activate or inhibit fibroblasts, based on their mRNA expression profiles. DEGs were identified according to median EPIC‐CAF values using the edgeR package. The gene symbols were further mapped to HG‐U133A probes on the GPL96 platform and the genes were sorted by decreasing fold change values. The top 1000 probes (500 down‐regulated and 500 up‐regulated) were selected for computation. A cMAP was applied to each cancer, and permuted results were obtained. The permuted results were further filtered to identify the compounds that could significantly (*p* < 0.05) influence (activate or inhibit) CAFs (Table S10).

### 2.9 Statistical analysis

Survival analysis was conducted using the survival and survminer packages. The best cutoff value in each cancer cohort was adopted to draw the Kaplan–Meier plot using the log‐rank test. Univariate Cox regression analysis was conducted to calculate the hazard ratios (HRs) for OS. The Shapiro–Wilk test was used to examine the normality of the variables. For comparison between two unpaired groups, Student's *t*‐test and Wilcoxon rank‐sum test were applied for normally and non‐normally distributed variables, respectively. For matched samples, paired Student's *t*‐test was applied. One‐way ANOVA was used for parametric multigroup comparisons. Correlation analysis was performed using Spearman's method. All *p* values were two‐tailed and are reported in the figures and figure legends. Statistical analysis was performed using R software (version 4.0.4, https://www.r‐project.org) or Python 3.8.5.

## 3 RESULTS

**FIGURE 1:**
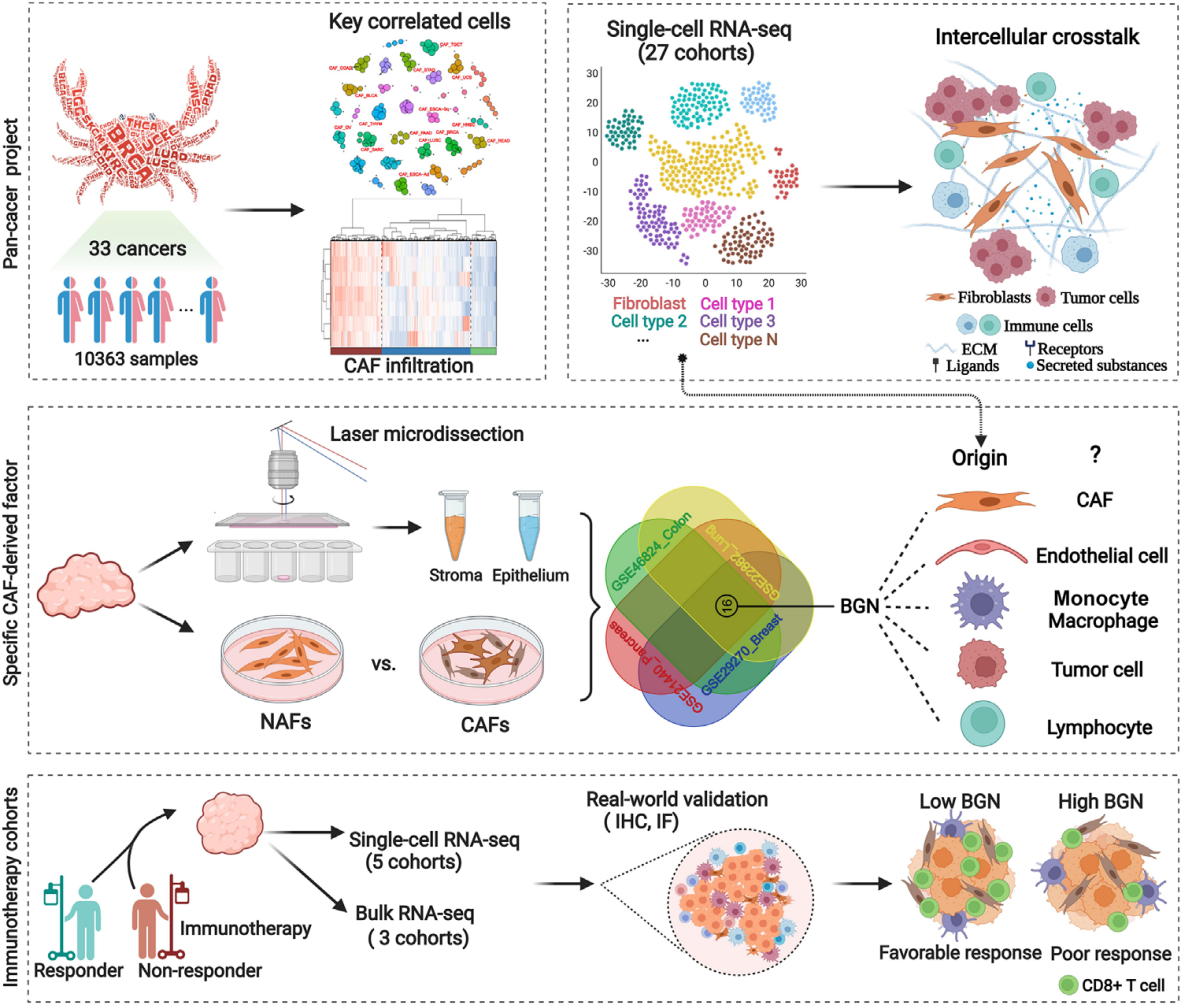
Schematic representation of the workflow in this study. 33 types of cancers from the TCGA pan‐cancer cohort were selected to explore the landscape of cancer‐associated fibroblasts. Tissue laser microdissection and single‐cell RNA sequencing were applied to examine biglycan expression and its value for immunotherapy.

The overall design of this study is illustrated in Figure 1. In summary, the correlations among crucial components in the TME were first analyzed in 33 cancers from TCGA. Strong associations between CAFs and immune cells were identified, and the cancer samples were further clustered according to the expression of classic CAFs markers. Subsequently, laser microdissection datasets were used to compare the expression profiles between stromal and tumor tissues. Finally, single‐cell sequencing data revealed fibroblast‐specific markers and their roles in predicting immunotherapy response.

### 3.1 Clinical relevance of CAFs and their correlation with other TME components

**FIGURE 2:**
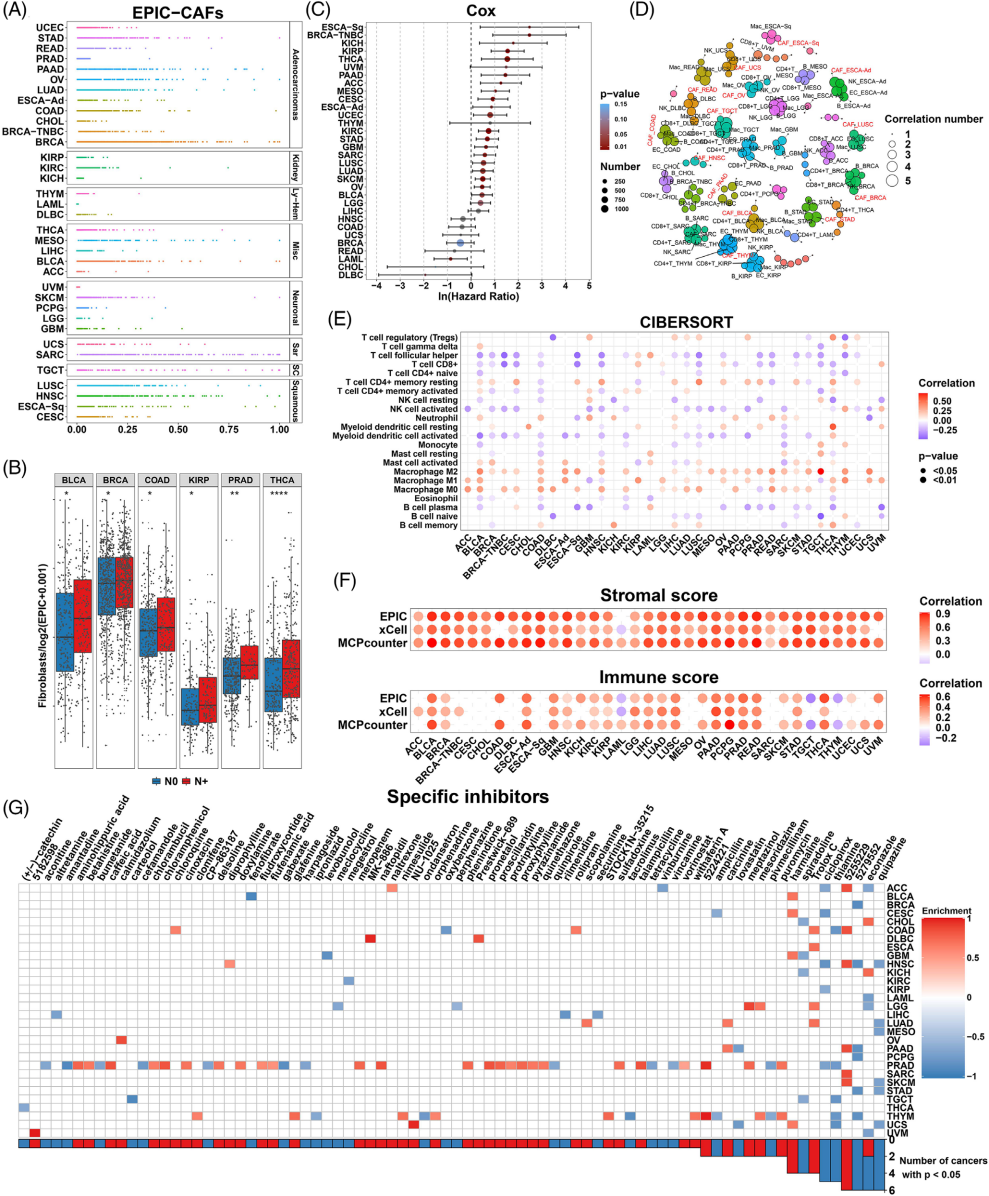
Clinical relevance and significant roles of CAFs in the tumor microenvironment. (A) Relative abundance of EPIC‐estimated fibroblasts in 33 cancers from the TCGA pan‐cancer cohort. (B) Boxplots comparing the abundance of fibroblasts in cancer samples with or without lymph node metastasis. N0 (lymph node negative) and N+ (lymph node positive) are represented as blue and red, respectively. *p* values from Student's *t*‐test. (C) Hazard ratio for overall survival of higher fibroblast abundance. *p* values from Cox regression model. (D) Crucial components in TME of cancers. Correlation number: The number of other cells correlated with specific cells in the TME (criteria: |Correlation *R*| > 0.3 and *p* < 0.05). *p* values from Spearman correlation analysis. (E) Correlation between fibroblast score (EPIC) and CIBERSORT LM22 immune cell score. Only significant values are shown (*p* < 0.05). Positive and negative correlations are represented as red and blue. *p* values from Spearman correlation analysis. (F) Correlation between fibroblast score (EPIC) and ESTIMATE immune/stromal scores. Only significant values are shown (*p* < 0.05). Positive and negative correlations are represented as red and blue. *p* values from Spearman correlation analysis. (G) Heatmap showing enrichment scores of specific compounds from the cMAP database to target fibroblasts for the TCGA pan‐cancer cohorts. The inhibitors are sorted from left to right in the order of ascending number of significantly enriched cancer types. Positive and negative enrichment scores are represented as red and blue. *p* values from cMAP analysis. *p* values are reported as follows: ns, nonsignificant; *, *p* < 0.05; **, *p* < 0.01; ***, *p* < 0.001; ****, *p* < 0.0001.

The EPIC algorithm was used to explore the abundance of fibroblasts in TME. Among the 33 cancers, acute myeloid leukaemia and uveal melanoma (UVM) showed relatively low CAFs scores, while most other cancers displayed relatively moderate to high levels of CAFs infiltration (Figure 2A). In thyroid carcinoma (THCA), prostate adenocarcinoma, kidney renal papillary cell carcinoma (KIRP), colon adenocarcinoma (COAD), breast invasive carcinoma (BRCA) and bladder urothelial carcinoma (BLCA), EPIC‐CAF scores in tumors with lymphatic metastasis were significantly higher than those in tumor without lymphatic metastasis (Figure 2B). Pan‐cancer analysis of CAFs scores and EMT markers was performed, which revealed that fibroblast scores highly correlated with CDH2 (N‐cadherin), a mesenchymal marker (Figures S1C–S1E, upper panel). We could observe that CAFs scores positively correlated with mesenchymal genes in most cancers. Moreover, negative correlation between CAFs scores and epithelial markers could also be observed in some cancers (Figures S1C–S1E, lower panel). To further investigate if the CAFs score could affect the clinical prognosis, univariate Cox regression was employed on the basis of the best cutoff value. For most cancers, a higher EPIC‐CAF score predicted worse prognosis (Figure 2C). Interestingly, a high EPIC‐CAF score correlated with impaired OS in TNBC, a breast cancer subtype, but was not significantly associated with OS in breast cancer as a whole (Figure 2C). These results implied that CAFs were generally poor prognostic indicators, but its roles differ among subtypes or different cancers.

Correlation analysis was performed on the TME components. In each cancer, the correlation between a specific cell and other cells was calculated, and only those that correlated (|*r*| > 0.3) with other cells (*n* > 2) were shown. The bubble size represented the number of strong correlations of a specific cell with other immune cells estimated by EPIC. Close correlations were observed between CAFs and immune cells in the microenvironment as they were significantly associated with many other kinds of cells in many cancers (Figure 2D). To further verify these associations, we adopted another algorithm, CIBERSORT, to assess the abundance of 22 immune components. In most cancers, CAFs had a negative correlation with CD8+ T cells and activated NK cells, both of which are well‐known for their anti‐tumor functions.^65^, ^66^ Conversely, M0 and M2 macrophages showed positive correlation with CAFs in most cancers (Figure 2E). These results indicated that CAFs could recruit tumor‐promoting immune cells, while excluding anti‐tumor cells, further remodelling the TME and promoting tumor progression. The ESTIMATE algorithm was used to calculate the overall stromal and immune scores of TME. Interestingly, based on the three different methods, CAFs abundance consistently correlated positively with both stromal and immune scores in most cancers (Figure 2F). These results demonstrated strong associations between CAFs and tumor immunity, making CAFs an attractive therapeutic target.

A recent study has reported novel nanoparticles that helped to target CAFs.^67^ Hence we sought to analyze the potential drug candidates. However, only a very limited number of small molecules could influence fibroblasts in cancers (*p* < 0.05) according to cMAP, a tool for predicting potentially effective small molecular agents using gene expression signatures. Quipazine, 5279552, and 5255229 were predicted as the most effective agents (in six cancers) (Figure 2G and Table S10), although substantial work is needed to validate their potency. Econazole seems to play paradoxical roles in different cancers, indicating that therapeutic targets related to CAFs might not be shared by all cancers.

### 3.2 CAF‐markers‐based infiltration classifier

**FIGURE 3:**
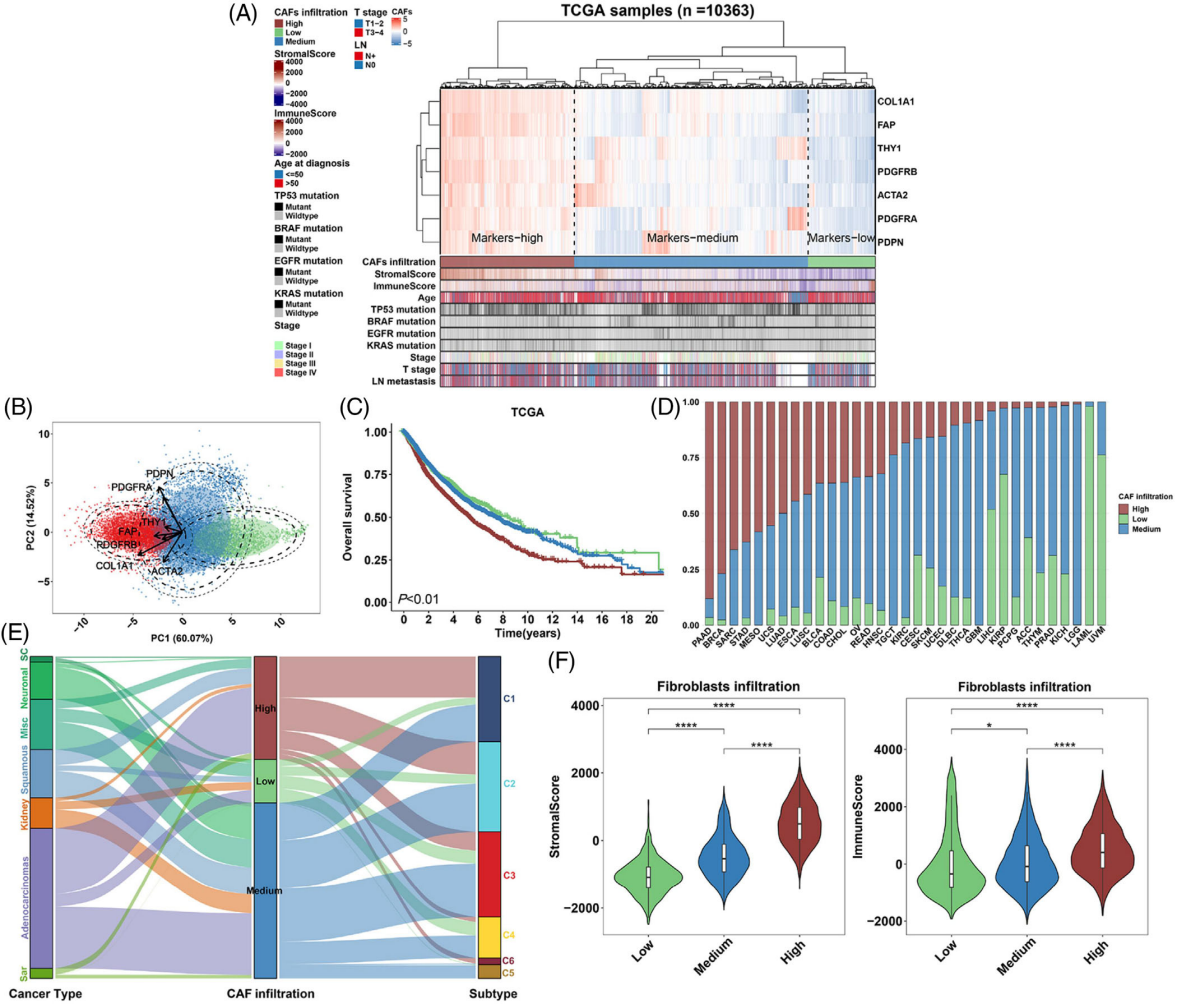
Relative CAFs infiltration levels in pan‐cancer cohorts. (A) Heatmap showing the clustering of classic CAFs markers and different levels of relative CAFs infiltration in the TCGA pan‐cancer cohort (*N* = 10 363). Bars under the heatmap show distributions of ESTIMATE, mutation and clinical features. For clustering of markers and samples, euclidean and ward. D2 methods were applied. (B) PCA plot showing PCA components (*x* and *y*‐axes) based on CAFs markers. Arrows, the profiling tendency of markers; eclipse, 80%; internal circle, 95% confidence interval; external circle, 98% confidence interval. (C) Kaplan–Meier plots showing the overall survival analysis of different CAF‐infiltration groups in TCGA pan‐cancer cohort. Low, medium and high CAFs infiltration were represented as green, blue and red. *p* value from log‐rank test. (D) Barplot showing the percentage of different CAFs infiltration levels (*y*‐axis) in 33 cancers of the TCGA pan‐cancer cohort. (E) Sankey diagram depicting the relationship of cancer types, CAFs infiltration levels and immune subtypes. High, medium and low infiltration levels are represented as red, blue and green, respectively. (F) Violin plots comparing the ESTIMATE stromal (left) and immune (right) scores in different infiltration groups. *p* values from Kruskal–Wallis test. *p* Values are reported as ns, nonsignificant; *, *p* < 0.05; **, *p* < 0.01; ***, *p* < 0.001; ****, *p* < 0.0001.

Considering the heterogeneity of CAFs, we used a clustering method to estimate fibroblast enrichment and understand their effects on clinical outcomes and associations with immune parameters. TCGA cohort was clustered into three groups with significantly different CAFs infiltration based on the gene expression of classic CAFs markers (Figure 3A). There was an ascending tendency of gene expression in the low infiltration group toward the high infiltration group. The clustering patterns of these genes were validated using PCA (Figure 3B). In most cancers, the high‐infiltration group exhibited higher stromal scores (Figures 3A, F and S2), further confirming the reliability of our clustering method. Kaplan–Meier analysis of the TCGA pan‐cancer cohort indicated that patients with high CAFs infiltration exhibited significantly worse OS (Figure 3C). In several types of cancers, high CAFs infiltration was associated with lymphatic metastasis and more advanced pathological T and tumor stages, whereas other clinical features did not seem to exhibit a common tendency throughout the entire TCGA cohort (Table S11 and Figure 3A). In this study, BRCA and PAAD showed the highest abundance of fibroblasts (Figure 3D), indicating the potentially important role of CAFs in these cancers. We further analyzed the survival outcomes of the different CAFs infiltration groups for each cancer. High CAFs infiltration predicted significantly poor OS in BLCA, GBM, KIRP, LUAD, MESO, OV, PAAD, STAD and THCA. In BRCA, LUSC and UVM, high infiltration indicated a tendency toward poor prognosis, although the difference was not statistically significant (Figure S3).

The distribution of CAFs infiltration in the different cancer types and immune subtypes was also investigated (Figure 3E). The three infiltration groups existed in all cancer types, except for sarcoma and germ cell tumors, which mostly consisted of the high and medium groups (Figure 3E). This result is consistent with the mesenchyme‐originated features of sarcoma.^68^ For immune subtype distribution, the TGF‐β‐dominant C6 cluster with a fraction of leukocytes mainly comprised high CAFs infiltration, which was expected, as fibroblast‐derived TGF‐β signalling is crucial for the TME. Interestingly, the high infiltration group was not involved in the immunologically quiet C5 cluster, which exhibited the lowest leukocyte fraction. Notably, higher CAFs infiltration, based on the clustering method, was also associated with higher ImmuneScores in many cancers (Figures 3F and S2). Overall, these results further indicated that fibroblasts promoted leukocyte infiltration and increased the complexity of the TME.

### 3.3 Single‐cell sequencing reveals specific transcriptional regulons for CAFs and intercellular crosstalk

**FIGURE 4:**
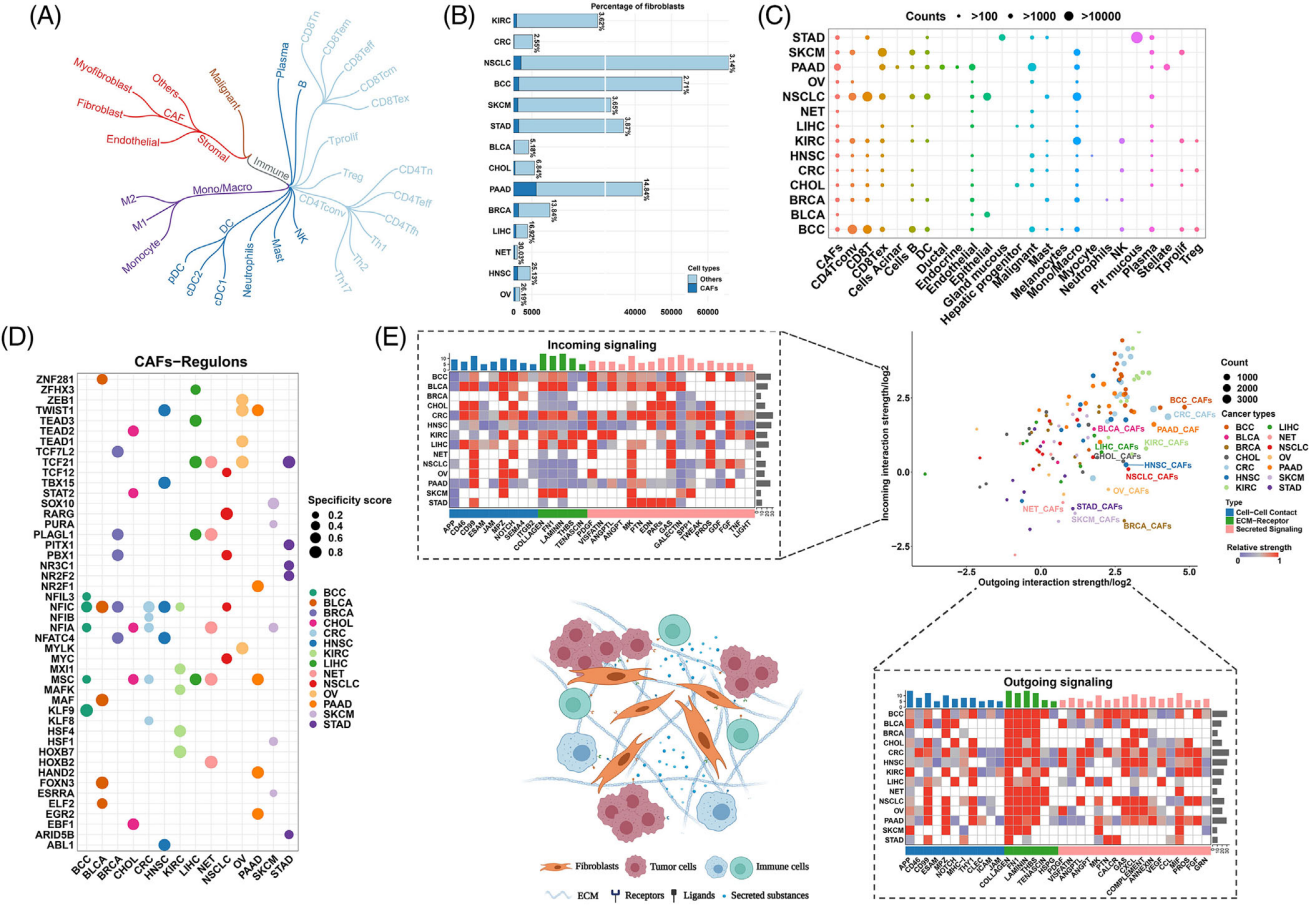
Intercellular crosstalk and transcriptional analysis of CAFs in single‐cell sequencing cohorts. (A) Brief classification of malignant, immune and stromal components in the tumor microenvironment. (B) Barplot showing the counts and percentage of fibroblasts in the enrolled cohorts. CAFs and other cells are represented as dark blue and light blue. (C) Bubble plot showing the counts of individual cells in cohorts. The cell counts are represented by the bubble size. (D) Bubble plot showing the top five transcriptional modules analyzed by SCENIC. Specificity scores are represented by the bubble size. (E) The relative strength of incoming (top left) and outgoing (lower right) signalling for cancer‐associated fibroblasts analyzed by CellChat (strength is scaled within individual cancers, and increasing value is represented from purple to red); Scatter plot showing the signalling for all cells in cohorts (cancers are represented as different c, cell numbers are represented as the dot size). Illustration of different kinds of intercellular communications for fibroblasts (lower left).

To facilitate the biological analysis of CAFs at the single‐cell level, pan‐cancer single‐cell sequencing datasets of 14 cancers were collected. The main cell types were malignant, immune and stromal cells (Figure 4A). The stromal cells mainly consist of CAFs and endothelial cells. The most common clusters of CAFs were fibroblasts secreting high levels of chemokines and cytokines and those expressing high levels of α‐SMA. The percentages and cell numbers of CAFs from the 14 single‐cell sequencing datasets are displayed in Figures 4B and C. Transcription factor network prediction and intercellular crosstalk estimation were performed using these datasets.

To identify specific transcriptional regulation networks for CAFs, a SCENIC analysis was performed. The detailed scores and cluster expression of the top regulons in the CAFs are shown in Figure S4. The top five regulons for fibroblasts in each tumor are shown in Figure 4D. We found that MSC, NFIA, NFIC and TCF21 were significantly effective regulons for fibroblasts in more than three cancers, indicating that they might be crucial in driving changes in cell state. Meanwhile, many regulons of fibroblasts, such as TCF7L2 in BRCA and NR2F1 in PAAD, further suggest the intertumoral heterogeneity of fibroblasts.

The cellular interaction signalling consists of three parts: ECM–receptor interaction, secreted signalling and cell–cell contact. The top representative signals are shown in Figure 4E. Intense outgoing signalling from CAFs was discovered using the CellChat algorithm, with the ECM–receptor interaction being the most obvious. Collagen, FN1, laminin and THBS play dominant roles in this process. Detailed ligand–receptor pairs are displayed in Figure S5 and Tables S12 and S13. Notably, collagen‐associated ligand–receptor pairs, such as COL1A1‐CD44 and COL6A1‐SDC1, are relatively unique signatures for CAFs because fibroblasts are one of the sources of collagens.^69^, ^70^ However, for other secreted signalling pathways, CAFs showed heterogeneous features among distinct cancers. GAS, CXCL, complement, annexin, MIF, PROS and FGF signalling were relatively common interactions, although none was consistently significant across all cancers. For cell–cell contacts, CD99, MPZ and THY1 were the common cellular interaction media in most cancers. For incoming signalling of CAFs, CD99, PDGF, MK and FGF have been identified as functional members in most cancers.^71^, ^72^, ^73^, ^74^

### 3.4 Stromal BGN is mainly expressed in fibroblasts

Given the intense outgoing signalling of CAFs, we sought to identify novel fibroblast‐specific secretory signatures. The expression profiles of tissue‐derived fibroblasts were collected and analyzed. Comparisons between fibroblasts from normal/benign breast, colon, lung and pancreatic tissues and the corresponding cancer tissues revealed that 16 protein‐coding genes were consistently up‐regulated in CAFs (Figure S6A). Among the 16 genes, ACTA2, the coding gene for α‐SMA, is one of the most well‐known CAFs markers. Six of these encode genes for secreted proteins, including BGN, CHPF, IGFBP7, NTF3, HHIPL1 and ST6GALNAC5.

**FIGURE 5:**
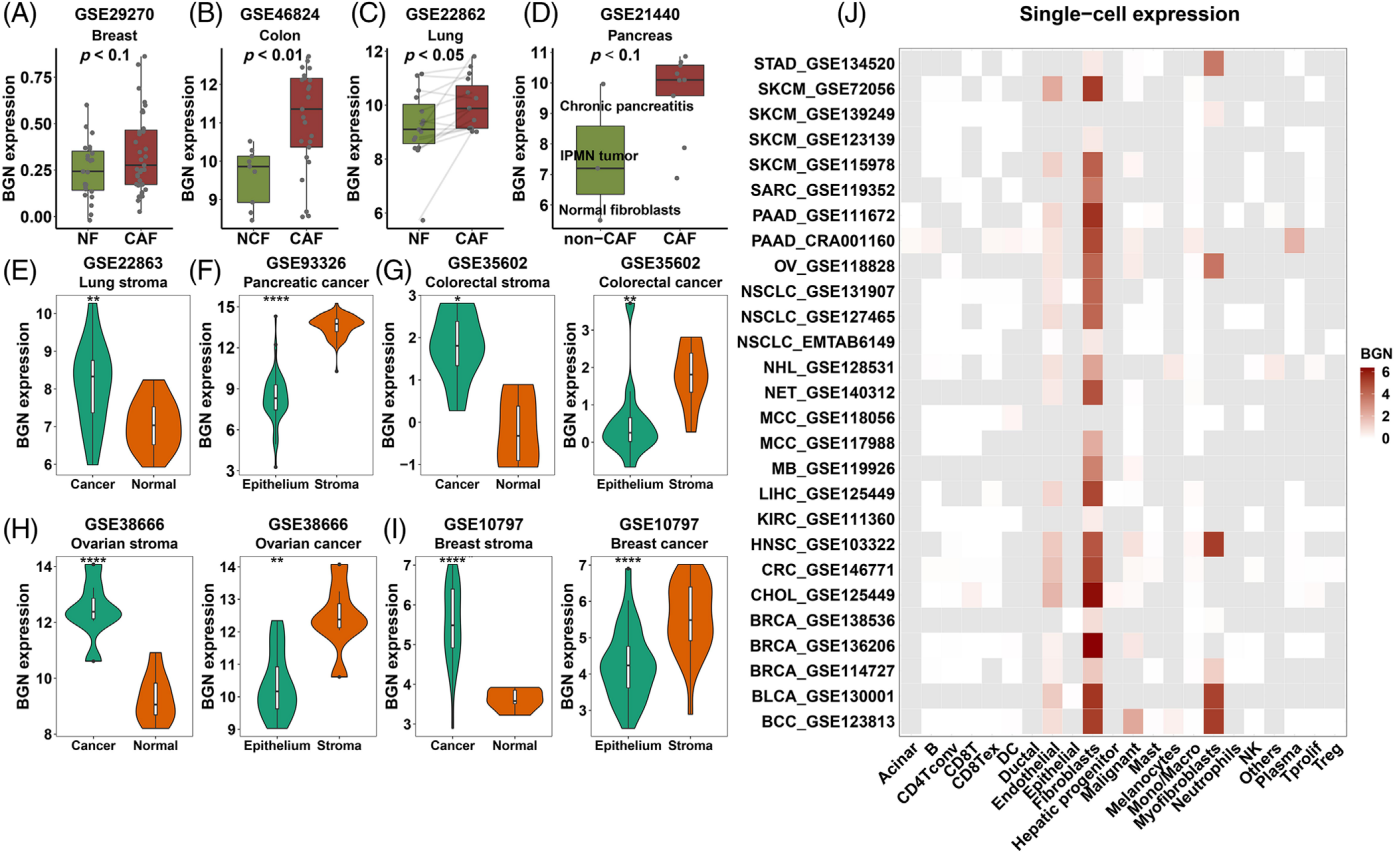
Stromal biglycan is mainly expressed in cancer‐associated fibroblasts. (A) Boxplots comparing BGN expression in CAFs and non‐CAFs of breast (A), colon (B), lung (C) and pancreatic (D) tissues. (E–I) Violin plots comparing the BGN expression of tissue microdissection: the stromal part of normal and cancer tissues of lung, colorectal, ovarian and breast samples; the cancer epithelium and stromal part of pancreatic, colorectal, ovarian, breast cancer. (J) Heatmap showing BGN expression in cells of scRNA‐seq datasets. The log2(TPM/10+1) value is shown, and cells not available are represented as grey tile. *p* value from paired/non‐paired Student's *t*‐test (normally distributed) and Wilcoxon rank‐sum test (non‐normally distributed). CAFs and non‐CAFs are represented as red and green. *P* values are reported as follows: ns, nonsignificant; *, *p* < 0.05; **, *p* < 0.01; ***, *p* < 0.001; ****, *p* < 0.0001.

We focused on BGN because the gene and fibroblasts are both closely related to ECM remodelling.^75^ BGN encodes biglycan, which is secreted in the ECM and plays a role in collagen assembly, muscle development and bone growth. Recent studies reported that BGN might be a crucial marker of fibroblasts in breast cancer and colorectal cancer.^75^, ^76^ However, its role in CAFs and its biological functions in cancer are still poorly understood. Herein, we found that BGN was uniformly up‐regulated in CAFs from different cancers, including breast, colon, lung and pancreatic cancers (Figures 5A–D). For further verification, we explored BGN expression in microdissected samples from multiple cancers. BGN expression was significantly higher in the stroma than in the epithelium in pancreatic, colorectal, ovarian and breast cancers (Figures 5F–I), implying the stromal origin of BGN. However, it was much lower in the normal stromal component than in the cancerous stromal component (Figures 5E–I). Therefore, we concluded that the upregulated BGN was mainly derived from cancerous stromal tissue.

To further clarify the specific cellular origin of BGN expression, various single‐cell sequencing cohorts were used for analysis. BGN was predominantly expressed in fibroblasts/myofibroblasts (CAFs) in various cancers (Figure 5J). The expression of BGN and classic CAFs markers (ACTA2, FAP and PDGFRB) in representative single‐cell sequencing datasets is shown in Figure S7. In addition, we found that they co‐localized with fibroblasts. We also observed that endothelial cells expressed low levels of BGN (Figure 5J). In addition, malignant cells in basal cell carcinoma also expressed a medium level of BGN (Figure 5J). Together, these results demonstrate that BGN was predominantly derived from CAFs and also partly from a small number of other cells.

### 3.5 Correlations between BGN and CAF infiltration, clinical outcomes and immune features

**FIGURE 6:**
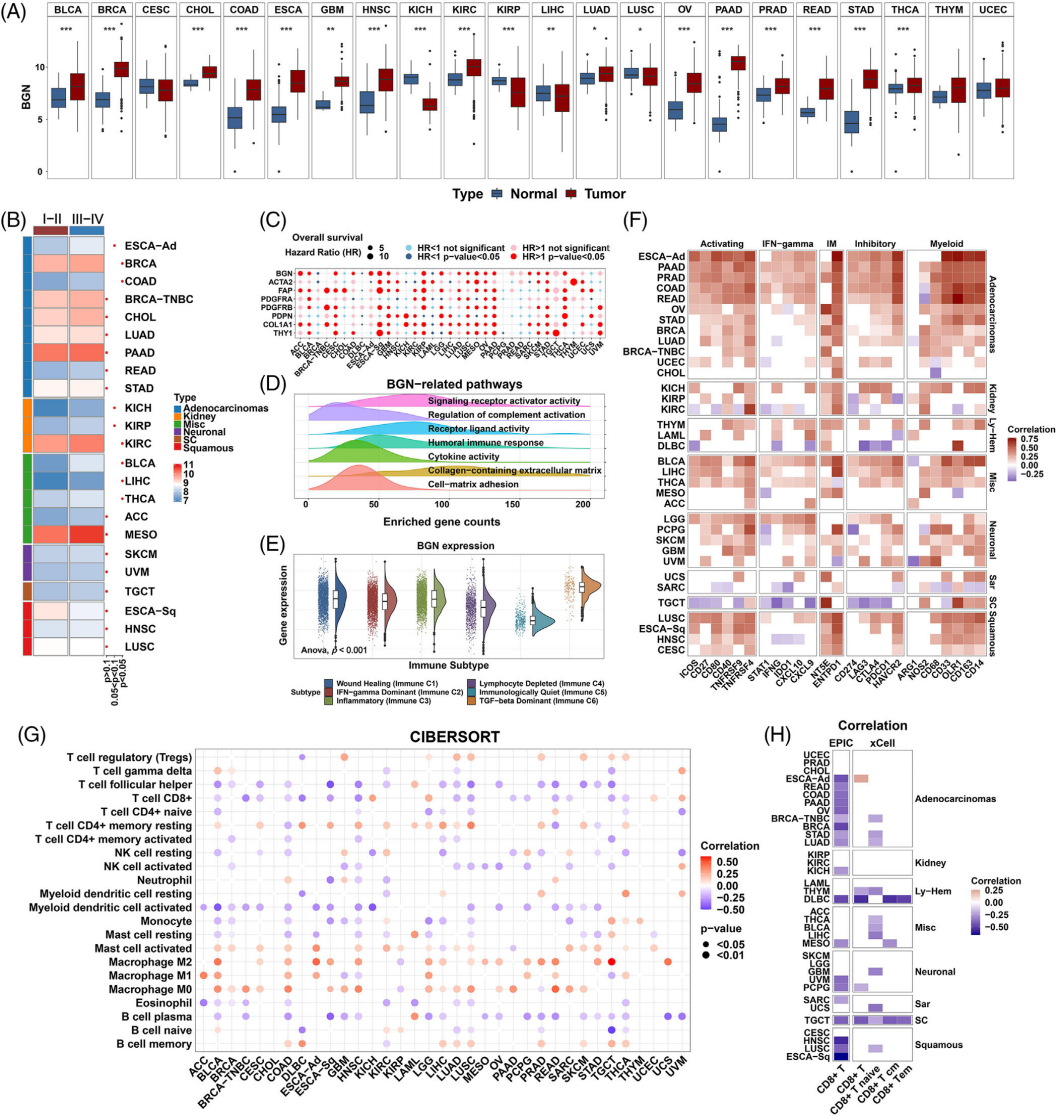
Clinical and tumor microenvironmental relevance of biglycan. (A) Boxplots comparing BGN expression between normal and cancer tissues from the merged TCGA‐GTEx cohort. Normal and tumor are represented as blue and red. *p* value from Student's *t*‐test. (B) Heatmap showing BGN expression in stage I–II and stage III–IV patients. *p* value from Student's *t*‐test. (C) Bubble plot showing the hazard ratio for BGN and classic CAFs markers analyzed by univariate Cox regression model. (D) Density plots showing Gene Ontology enrichment. The frequency (*y*‐axis) of enriched gene counts in cancer is shown. (E) Violin and dot plots showing BGN expression in immune subtypes of the TCGA cohort. *p* value from one‐way ANOVA test. (F) Heatmap showing the correlation between BGN and immune‐related genes. Correlation values without statistical significance are represented as blank. (G) Bubble plot showing the correlation of BGN expression and CIBERSORT LM22 immune cells. (H) Heatmap showing the correlation of BGN expression and CD8+ T cell infiltration calculated by EPIC and xCell algorithms. Positive and negative correlations are represented as red and blue. *p* value from Spearman correlation analysis. *p* values are reported as ns, nonsignificant; *, *p* < 0.05; **, *p* < 0.01; ***, *p* < 0.001; ****, *p* < 0.0001.

Pan‐cancer expression of BGN in bulk tumors is comprehensively displayed in Figure S6B, revealing that most of the cancers shared a relatively similar expression level of BGN, except for leukaemia (Figure S6B). In most of the cancers, BGN was significantly up‐regulated in tumor tissues, compared with that in unpaired or paired normal tissues (Figures 6A and S8). In addition, the expression of BGN was significantly higher in stages III‐IV BRCA, COAD, KIRC, BLCA, LIHC and THCA than in stages I–II tumors (Figure 6B). We also found that the expression of BGN positively correlated with ESTIMATE stromal scores, CAFs scores (calculated by MCP‐counter, EPIC and xCell algorithms), clustered CAFs infiltration levels and the expression of classic CAFs markers (Figures S6C and S6D), further supporting the fibroblast origin of BGN. We analyzed the correlation between BGN and inflammatory (iCAFs) and myofibroblastic CAFs (myCAFs) markers. The results indicated that BGN positively correlated with secreted molecules and myCAFs markers (Figure S9A).

Considering that BGN is generally upregulated in tumors, we suspected that it may participate in cancer development and further influence patients’ clinical outcomes. Indeed, Kaplan–Meier analysis indicated that lower BGN expression level predicted favourable OS in most cancers (Figure S10). Interestingly, the predictive value of BGN in breast cancer as a whole and in TNBC cohorts seemed to be contradictory. This might be due to the heterogeneity of fibroblast clusters across the molecular subtypes of breast cancer, which has been previously reported.^7^ Univariate Cox regression analysis also suggested that BGN was generally an adverse prognostic factor for OS, similar to the classic CAFs markers (Figure 6C).

GO enrichment was performed to elucidate the biological functions of BGN. The DEGs between the BGN high and BGN low groups were significantly enriched in ECM and immune‐related pathways (Figure 6D and Table S14). Consistently, the expression of BGN had a significant positive correlation with both the ESTIMATE stromal scores and immune scores (Figure S6E). For immune subtypes, patients in the C5 cluster (immunologically quiet) exhibited the lowest BGN level, while those in the C6 cluster (TGF‐β‐dominant) showed the highest level (Figure 6E). These results imply that BGN may be involved in ECM remodelling and immune activity.

To better characterize the BGN‐related immune profile, we examined the associations between the expression of BGN and IRGs and immune cell infiltration. On the one hand, BGN had a positive correlation with many functional IRGs, especially in gastrointestinal adenocarcinomas (Figures 6F and S9B). Notably, it was positively associated with both activating immune receptors (ICOS, CD27, CD80, CD40, TNFRSF9 and TNFRSF4) and inhibitory immune receptors/ligands (CD274, LAG3, CTLA4, PDCD1 and HAVCR2) (Figure 6F). TGF‐β was also one of the most highly correlated ligands (Figure S9B). On the other hand, BGN had a significant negative correlation with anti‐tumor immune cell infiltration, such as plasma cells, activated myeloid dendritic cells, activated NK cells, CD8+ T cells and follicular helper T cells, as determined by the CIBERSORT algorithm, but was positively correlated with tumor‐promoting macrophages (Figure 6G). The negative correlation between BGN level and CD8+ T cells was further confirmed by two other infiltration estimation algorithms (Figure 6H). The correlations between BGN and other immune components estimated by EPIC and xCell are also shown (Figures S9C and S9D).

### 3.6 BGN predicts poor response to immunotherapy

**FIGURE 7:**
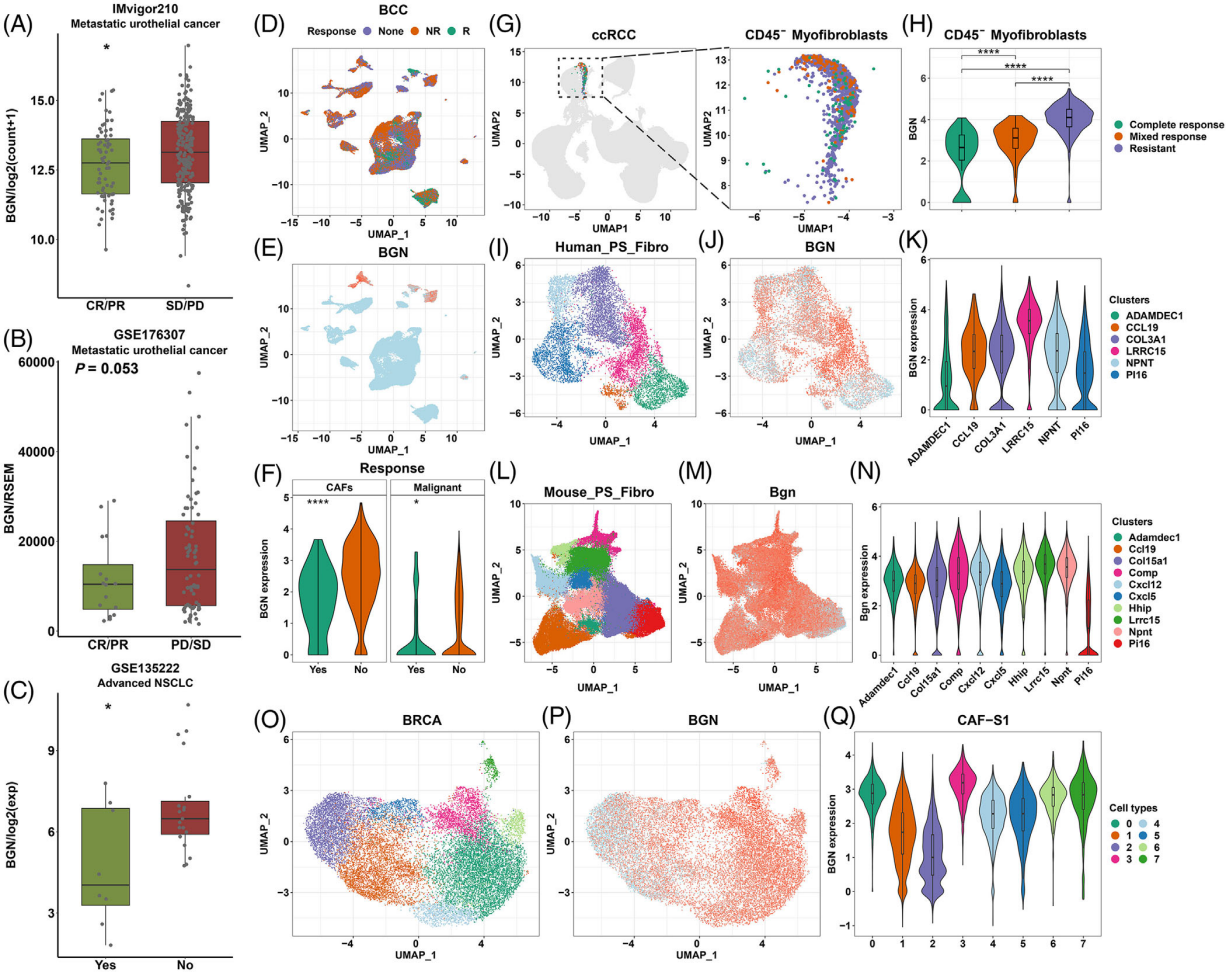
BGN predicts poor response of patients to immunotherapy. Boxplots comparing BGN expression in patients responding or not responding to immunotherapies in metastatic urothelial cancer (A, B) and advanced non‐small cell lung cancer (C). *p* values from Student's *t*‐test. BCC scRNA‐seq: Scatter plot showing the distribution of cells from NR (nonresponse), R (response) and None (no record) (D); BGN expression in cells (E); violin plots comparing BGN expression in CAFs and malignant cells of patients with or without response (F). Responses and nonresponses are represented as green and brown, respectively. *p* value from Student's *t*‐test. ccRCC scRNA‐seq: Scatter plots showing CD45‐ myofibroblasts (G). Violin plot shows BGN expression in fibroblasts of the complete response, mixed response and resistance groups (H). *p* value from one‐way ANOVA test and corrected. Complete response, mix response and resistance are represented as green, brown and purple, respectively. Cross‐tissue fibroblast subsets: I–K, Different subsets of human fibroblasts and BGN distribution; L and M, Different subsets of mouse fibroblasts and BGN distribution. Breast cancer CAF‐S1 subsets: Scatter plot showing fibroblast CAF‐S1 subsets (O) and BGN distribution (P); Violin plot showing BGN expression in subsets 0–7 (Q). *p* values are reported as follows: ns, nonsignificant; *, *p* < 0.05; **, *p* < 0.01; ***, *p* < 0.001; ****, *p* < 0.0001.

Owing to the close relationship between BGN and immune parameters, we suspected that BGN might serve as a novel biomarker for immunotherapy. Therefore, the correlations between BGN levels and immunotherapy response were analyzed in multiple cancers, including urothelial cancer, basal cell carcinoma, non‐small cell lung cancer, clear cell renal cell carcinoma (ccRCC) and breast cancer. Three bulk profiling datasets were used to compare the expression levels of BGN in patients with different responses to ICB (Figures 7A–C). As expected, BGN expression was significantly up‐regulated in the non‐responder group. To facilitate CAF‐specific analysis, single‐cell sequencing data from the immunotherapy cohorts were analyzed. In basal cell carcinoma, BGN was mainly located on clustered cells from patients with a poor response to anti‐PD‐1 treatment (Figures 7D and E). Meanwhile, the BGN levels before treatment in both CAFs and malignant cells were significantly higher in the non‐response group than in the responsive group, and the trend was particularly pronounced in CAFs (Figure 7F). We also found that the expression of BGN in myofibroblasts was gradually upregulated in patients with complete, mixed and resistant responses to combined treatment with ipilimumab and nivolumab (Figures 7G and H).

Previous studies have reported that specific clusters of CAFs may contribute to resistance to immunotherapy.^16^, ^17^ Hence, we further analyzed the expression levels of BGN in CAFs subpopulations based on single‐cell‐level datasets. As expected, the BGN level of LRRC15+ fibroblast clusters from humans was higher than that of other clusters (Figures 7I–K). A similar result was observed in purified fibroblast clusters from mice (Figures 7L–N). In breast cancer, eight clusters were identified in the FAP+/CAF‐S1 population (Figure 7O). The highest BGN expression was observed in clusters 0 and 3, which was characterized by the secretion of extracellular proteins and TGF‐β signalling, respectively (Figures 7P and Q).

**FIGURE 8:**
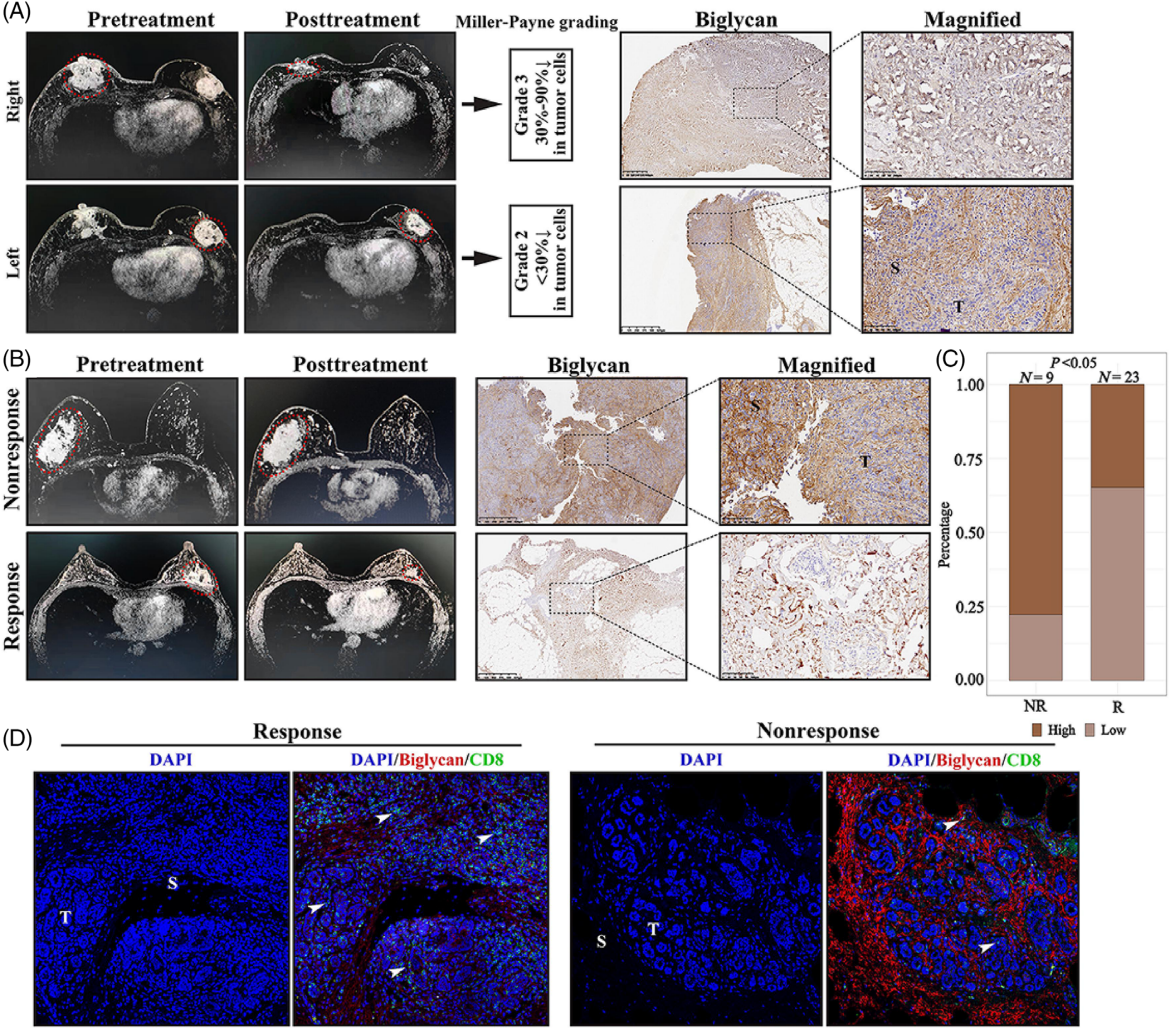
Validation of the correlation between biglycan and immunotherapy response in a clinical cohort. (A) The magnetic resonance imaging (left panel), Miller–Payne grade (middle panel) and immunohistochemical biglycan expression (right panel) in a patient diagnosed with bilateral breast cancer. (B) Representative images of magnetic resonance imaging (left panel) and immunohistochemical biglycan expression (right panel) in response and nonresponse groups. (C) Bar plot showing the proportion of high and low biglycan expression in NR (nonresponse) and R (response) groups. *p* value from Fisher exact test. (D) Representative image of immunofluorescence showing the biglycan expression and CD8+ T cell infiltration in response and nonresponse groups.

We further validated our results in a clinical cohort of breast cancer patients. Interestingly, in a patient diagnosed with bilateral breast cancer, cancer bulks on the left and right sides responded differently to neoadjuvant PD‐1 blockade and chemotherapy (Figure 8A). MRI showed a marked shrinking of the cancer on the right side. MP grading further confirmed the results of MRI and indicated that after neoadjuvant therapy, tumor cell reduction on the right and left sides were grade 3 and grade 2, respectively. As expected, the left cancer showed a higher BGN level than the right cancer in the stroma, which is consistent with the aforementioned results. In a neoadjuvant PD‐1 blockade and chemotherapy cohort, we revealed different expression levels of BGN in patients who were sensitive or resistant to treatment (Figure 8B). We observed a significantly higher BGN level in patients who did not respond to treatment than in those that responded to treatment (Figure 8C). Furthermore, a lower level of BGN seemed to correlate with greater infiltration of CD8+ T cells after neoadjuvant treatment (Figure 8D).

## 4 DISCUSSION

Recent studies have provided insights into CAFs and deepened our understanding of their heterogeneity and biological characteristics in cancers.^70^ Herein, we took advantage of pan‐cancer bulk and single‐cell sequencing datasets to elucidate the overall landscape of fibroblasts in cancer, including their clinical and immune relevance, specific transcriptional regulons and intercellular crosstalk. We further identified that BGN, mainly derived from CAFs, served as a negative prognostic factor for OS and a predictive biomarker for poor response to immunotherapy.

CAFs have historically been regarded as tumor‐promoting components because of their effects on the remodelling of ECM and production of growth factors.^4^ Previous studies also reported therapeutic strategies on CAFs.^77^ Besides, we also reported a novel CAFs‐related pattern in TNBC.^78^ Indeed, in the pan‐cancer cohort analyses of this study, CAFs correlated with poor OS in most cancers. The result remained constant regardless of whether CAFs infiltration was estimated using the EPIC algorithms or the clustering method. However, we also observed contradictory prognostic values for CAFs in the overall breast cancer and TNBC cohorts. This is one piece of evidence that illustrates the heterogeneity of CAFs and suggests the existence of different CAFs subsets in distinct molecular subtypes of breast cancer, as previously reported.^7^ Moreover, CAFs may exhibit anti‐tumorigenic effects under certain circumstances. To understand the mechanisms driving CAFs heterogeneity, we utilized scRNA‐seq data to trace the specific transcriptional regulons of CAFs. As expected, many regulons were tumor‐specific, which might be explained by the different origins of CAFs in different tumors.

In recent years, the crosstalk between CAFs and immune cells has attracted a lot of attention. In the current study, the abundance of CAFs had a significant negative correlation with infiltrating cytotoxic CD8+ T cells and activated NK cells in most cancers, indicating that CAFs, as suggested by previous studies, might prevent cytotoxic T cell recruitment within tumors and further attenuate the anti‐tumor immune response.^79^ Additionally, there was also a strong correlation between the abundance of CAFs and macrophages, and this is not surprising because CAFs are involved in macrophage differentiation and polarization through secreted signalling. Further analyses at the single‐cell level will enable a better understanding of the cellular crosstalk. Cell–cell contact, secreted signalling and ECM are vital media through which CAFs communicate with other cells. On this basis, we identified a prominent fibroblast‐specific secretion signal called BGN (the gene encoding biglycan). Our findings, based on microdissection and scRNA‐seq cohorts, supported that BGN expression is mainly derived from CAFs across multiple tumors and is upregulated in the cancerous stroma. We also observed that endothelial cells expressed a small amount of BGN, which was consistent with a previous report that these cells are a potential cellular source of fibroblasts.^5^ Therefore, CAFs may interact with immune cells by secreting BGN and contribute to the immunosuppressive TME.

Immunotherapies, including therapies with different ICBs, have shown promising therapeutic efficacy against malignancies.^80^, ^81^ Nevertheless, some patients do not respond to ICB, and the biological mechanisms underlying primary resistance are still poorly understood. This study, for the first time, showed that BGN correlated with poor response to ICB in several types of human solid tumors, either at the bulk  or single‐cell level. BGN is highly expressed in immunosuppressive subsets of fibroblasts, namely, clusters 0 and 3 of CAF‐S1 and the LRRC15+ cluster. Previous studies have reported that these clusters are associated with resistance to ICB.^16^, ^17^ Moreover, real‐world clinical practice validated that breast cancer patients with low BGN levels, including those with bilateral breast cancer, were more likely to respond to neoadjuvant PD‐1 blockade combined with chemotherapy. Therefore, BGN may account for immunotherapy resistance in these fibroblast clusters. Together, this evidence implies the predictive value of BGN in ICB response. However, the mechanisms underlying BGN expression and ICB response remain unclear. Interestingly, BGN was positively associated with a series of IRGs that were either immune activated or inhibitory. This result implies that BGN may induce immune tolerance in the TME. In addition, we found that the TGF‐β‐dominant immune subtype displayed the highest level of BGN, and there was a strong correlation between BGN level and TGF‐β expression. It has been reported that TGF‐β attenuates the immunotherapy response; this may also provide an explanation for the predictive role of BGN, despite its ambiguous causality.^57^ Furthermore, BGN showed negative correlation with the infiltration of anti‐tumor cytotoxic cells, especially CD8+ T cells, in pan‐cancer analysis, which was consistent with a recent study in breast cancer demonstrating that the inhibition of stromal BGN increased CD8+ T cell infiltration in tumor‐bearing mice.^82^ Recent studies have supported the inhibitory effects of BGN on CD8+ T cell infiltration and provided the basis for targeting BGN to overcome primary therapeutic resistance. Other studies have demonstrated that BGN serves as a new ligand for CD14 in macrophages with high affinity and mediates TLR2/4‐dependent inflammatory signaling.^83^, ^84^ Hence, we hypothesized that BGN might also attenuate ICB‐associated responses by modulating macrophages to establish an immunosuppressive TME. Alternatively, BGN, a proteoglycan in the ECM, may serve as a physical barrier to isolate and protect cancer cells from cytotoxic cells. Further investigation is warranted to confirm these hypotheses.

This study had several limitations. First, different clusters of CAFs were analyzed as a whole in the scRNA‐seq cohorts. Different subsets of fibroblasts and their specific intercellular cross‐talk might have been overlooked. Second, all analyses in the current study were based on RNA expression of transcriptome data. However, epigenetic factors, including translational and post‐translational modifications, can cause bias at the protein level. In vivo and in vitro experiments are needed to confirm the key findings of this pan‐cancer study. Thirdly, we admit that some patients with unresectable tumor might not be suitable for sampling and was not enrolled in these public cohorts. But this bias was common in previous studies and currently hard to resolve. Finally, different ratios of the stroma to tumor in bulk‐seq cohorts could cause potential bias, as samples with limited stromal components pose a challenge to the accuracy of the analysis of fibroblasts.

## 5 CONCLUSIONS

This study focused on depicting the overall landscape of CAFs in various types of cancer. CAFs generally correlated with clinical outcomes and immune activity in cancer. BGN, a relatively CAF‐specific secretion signature, correlated with poor prognosis and is a predictive biomarker for poor response to ICB. Further research exploring the modulation of the transcription, translation, transportation, excretion and extracellular behaviour of BGN will further elucidate its role in the TME and its contribution to cancer development and immunotherapy resistance.

## CONFLICT OF INTEREST

No conflict of interest is declared.

## Supporting information

Supporting Information

Supporting Information

Supporting Information

Supporting Information

Supporting Information

Supporting Information

Supporting Information

Supporting Information

Supporting Information

Supporting Information

Supporting Information

Supporting Information

Supporting Information

Supporting Information

Supporting Information

Supporting Information

Supporting Information
