# Supplementary material for: Dissecting the role of cancer‐associated fibroblast‐derived biglycan as a potential therapeutic target in immunotherapy resistance: A tumor bulk and single‐cell transcriptomic study
Source: Clin Transl Med. 2023 Feb 11;13(2):e1189. doi: 10.1002/ctm2.1189 (PMC9920016; doi:10.1002/ctm2.1189)

a

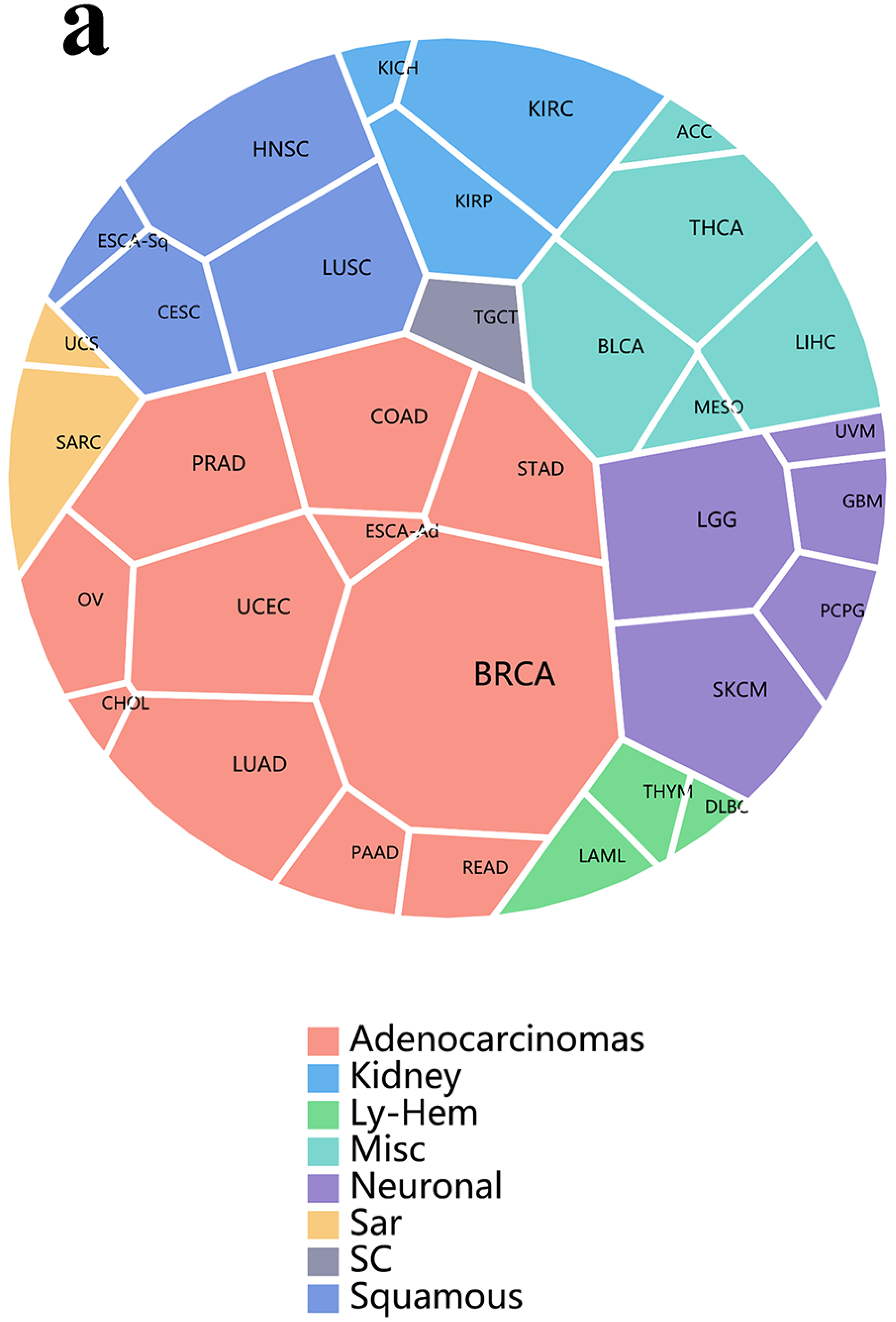

b

Tumor Type	Abbreviation
Adrenocortical Carcinoma	ACC
Bladder Urothelial Carcinoma	BLCA
Breast Invasive Carcinoma	BRCA
Cervical Squamous Cell Carcinoma and Endocervical Adenocarcinoma	CESC
Cholangiocarcinoma	CHOL
Colon Adenocarcinoma	COAD
Lymphoid Neoplasm Diffuse Large B-cell Lymphoma	DLBC
Esophageal Carcinoma	ESCA
Glioblastoma multiforme	GBM
Head and Neck Squamous Cell Carcinoma	HNSC
Kidney Chromophobe	KICH
Kidney Renal Clear Cell Carcinoma	KIRC
Kidney Renal Papillary Cell Carcinoma	KIRP
Acute Myeloid Leukemia	LAML
Brain Lower Grade Glioma	LGG
Liver Hepatocellular Carcinoma	LIHC
Lung Adenocarcinoma	LUAD
Lung Squamous Cell Carcinoma	LUSC
Mesothelioma	MESO
Ovarian Serous Cystadenocarcinoma	OV
Pancreatic Adenocarcinoma	PAAD
Pheochromocytoma and Paraganglioma	PCPG
Prostate Adenocarcinoma	PRAD
Rectum Adenocarcinoma	READ
Sarcoma	SARC
Skin Cutaneous Melanoma	SKCM
Stomach Adenocarcinoma	STAD
Testicular Germ Cell Tumors	TGCT
Thyroid Carcinoma	THCA
Thymoma	THYM
Uterine Corpus Endometrial Carcinoma	UCEC
Uterine Carcinosarcoma	UCS
Uveal Melanoma	UVM

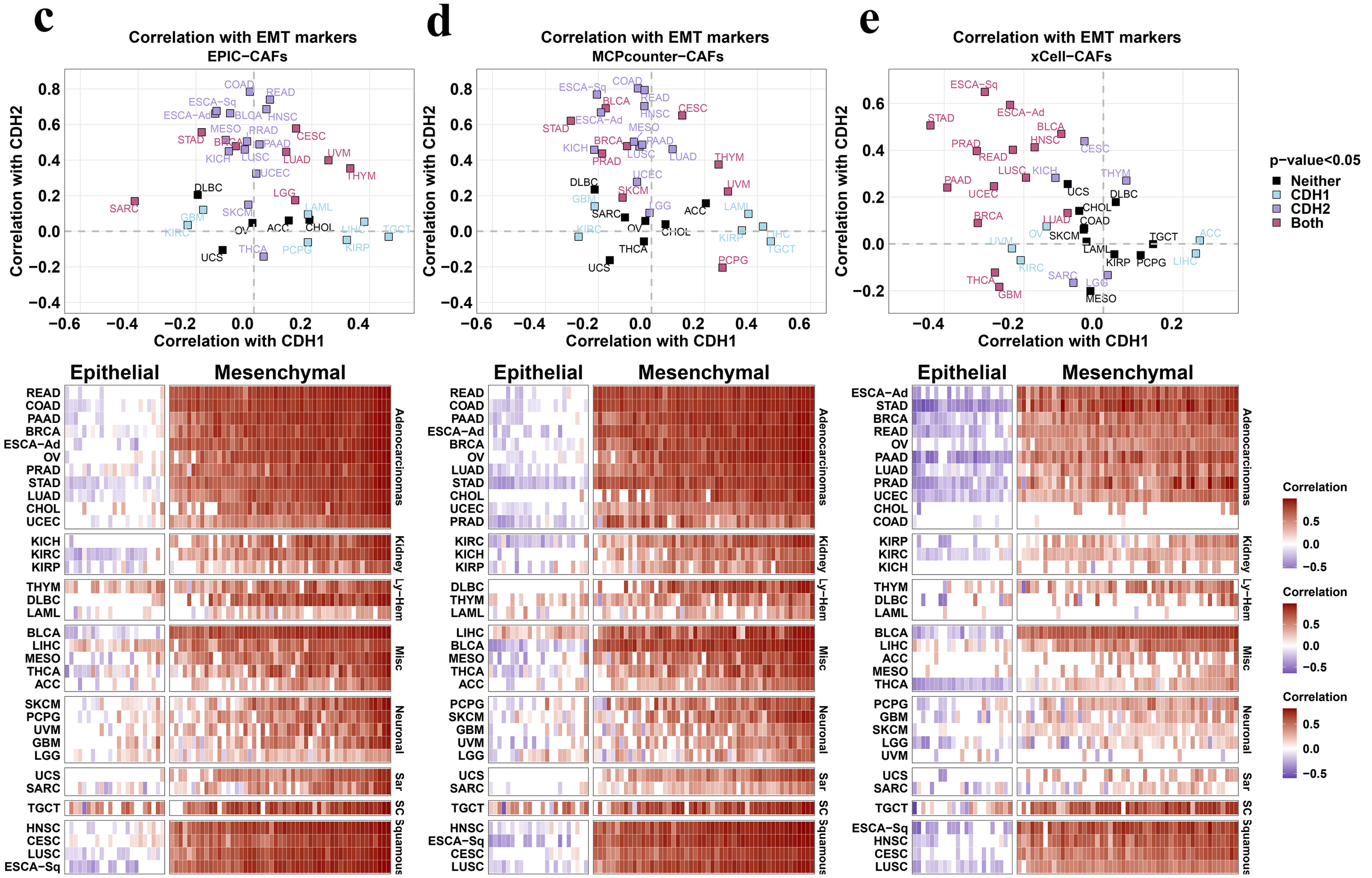

Supplement: Supplementary file 1 — Supporting Information [file CTM2-13-e1189-s010.pdf]
