# Supplementary material for: Dissecting the role of cancer‐associated fibroblast‐derived biglycan as a potential therapeutic target in immunotherapy resistance: A tumor bulk and single‐cell transcriptomic study
Source: Clin Transl Med. 2023 Feb 11;13(2):e1189. doi: 10.1002/ctm2.1189 (PMC9920016; doi:10.1002/ctm2.1189)
Supplement: Supplementary file 2 — Supporting Information [file CTM2-13-e1189-s013.pdf]

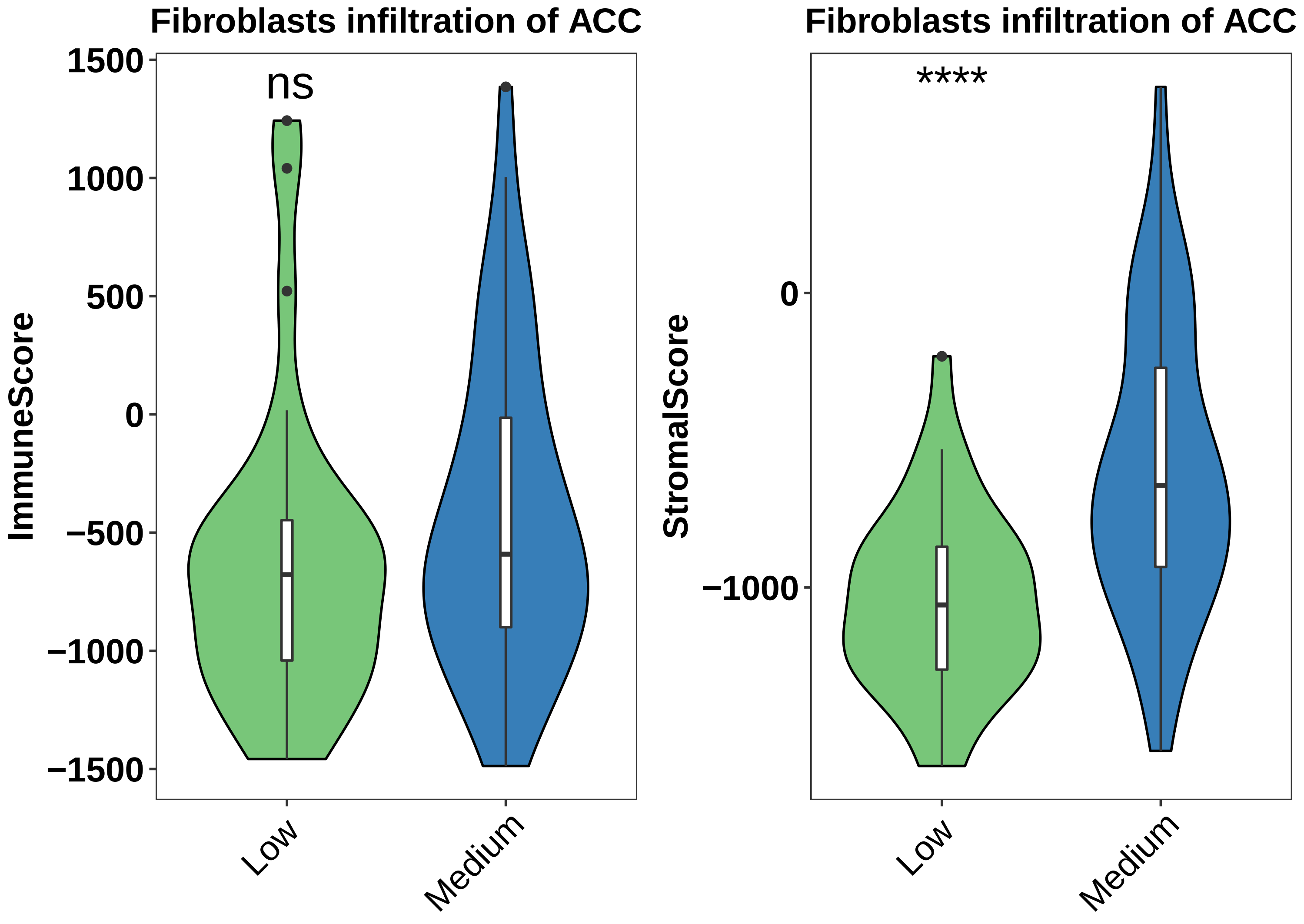
Fibroblasts infiltration of ACC
ImmuneScore
1500
1000
500
0
−500
−1000
−1500
ns
Low
Medium
Fibroblasts infiltration of ACC
StromalScore
0
−1000
****
Low
Medium

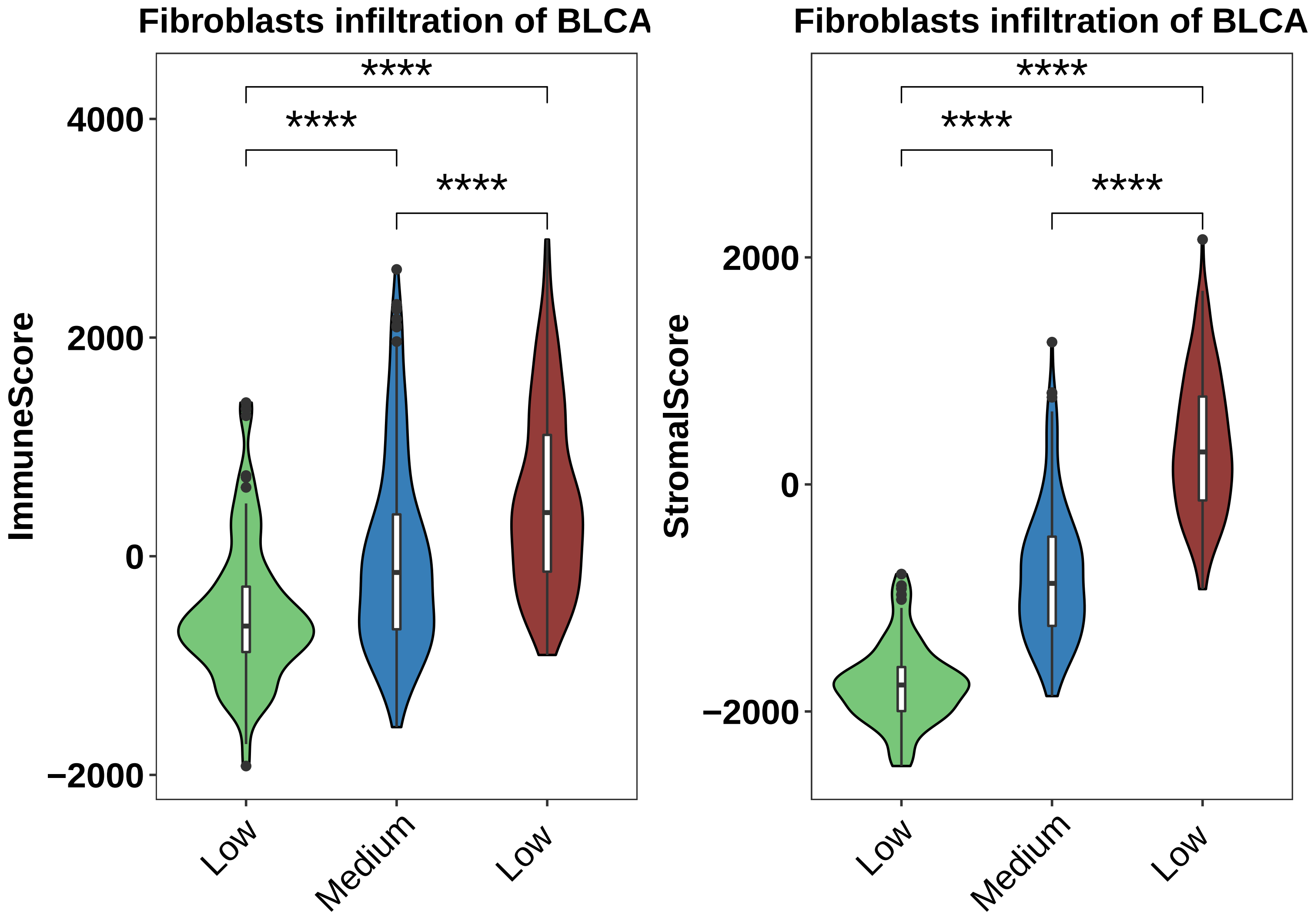

Fibroblasts infiltration of BLCA
ImmuneScore
4000
2000
0
−2000
****
****
****
Low
Medium
Low
Fibroblasts infiltration of BLCA
StromalScore
2000
0
−2000
****
****
****
Low
Medium
Low

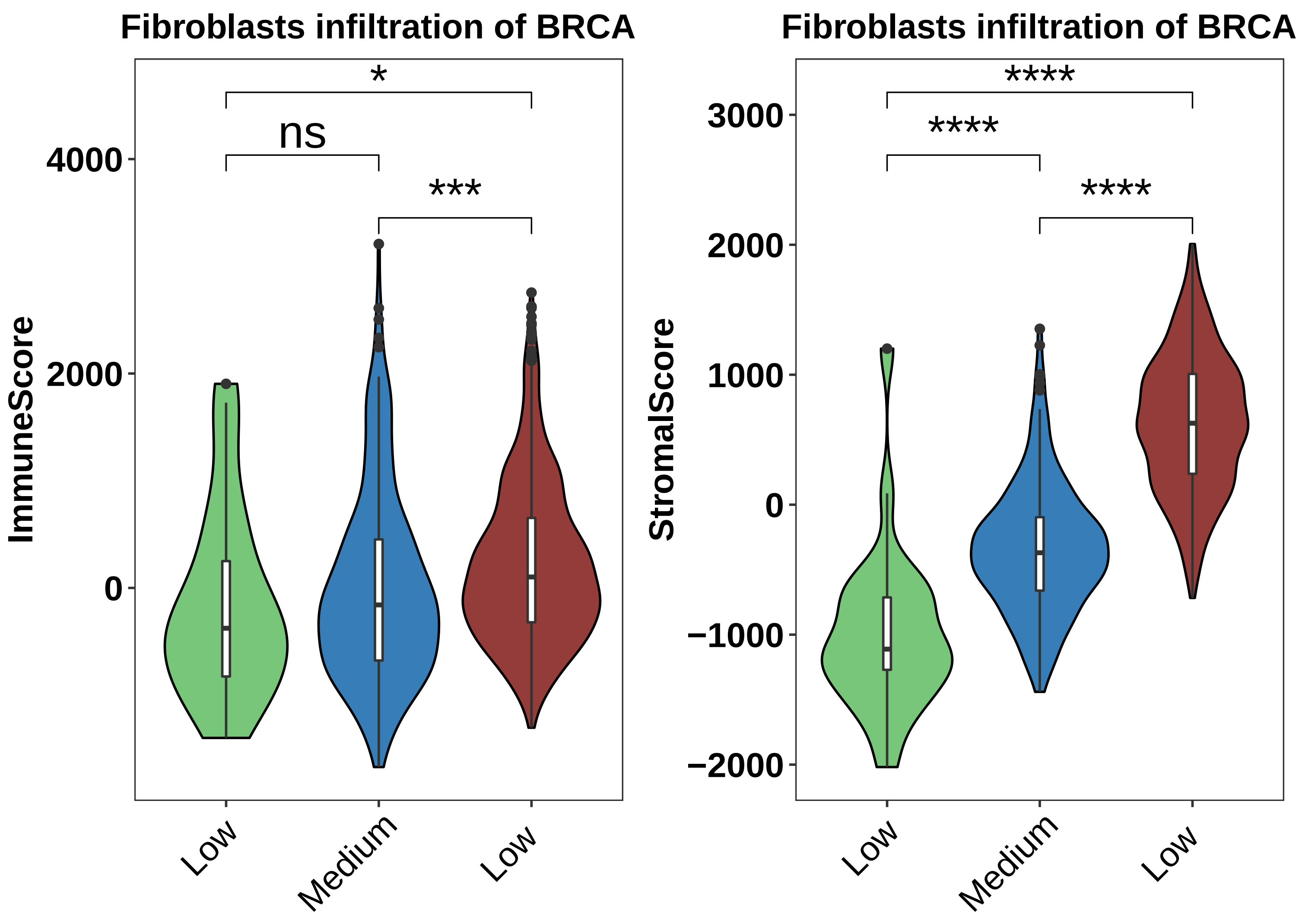

Fibroblasts infiltration of BRCA
ImmuneScore
4000
2000
0
*
ns
***
Low
Medium
Low
Fibroblasts infiltration of BRCA
StromalScore
3000
2000
1000
0
−1000
−2000
****
****
****
Low
Medium
Low

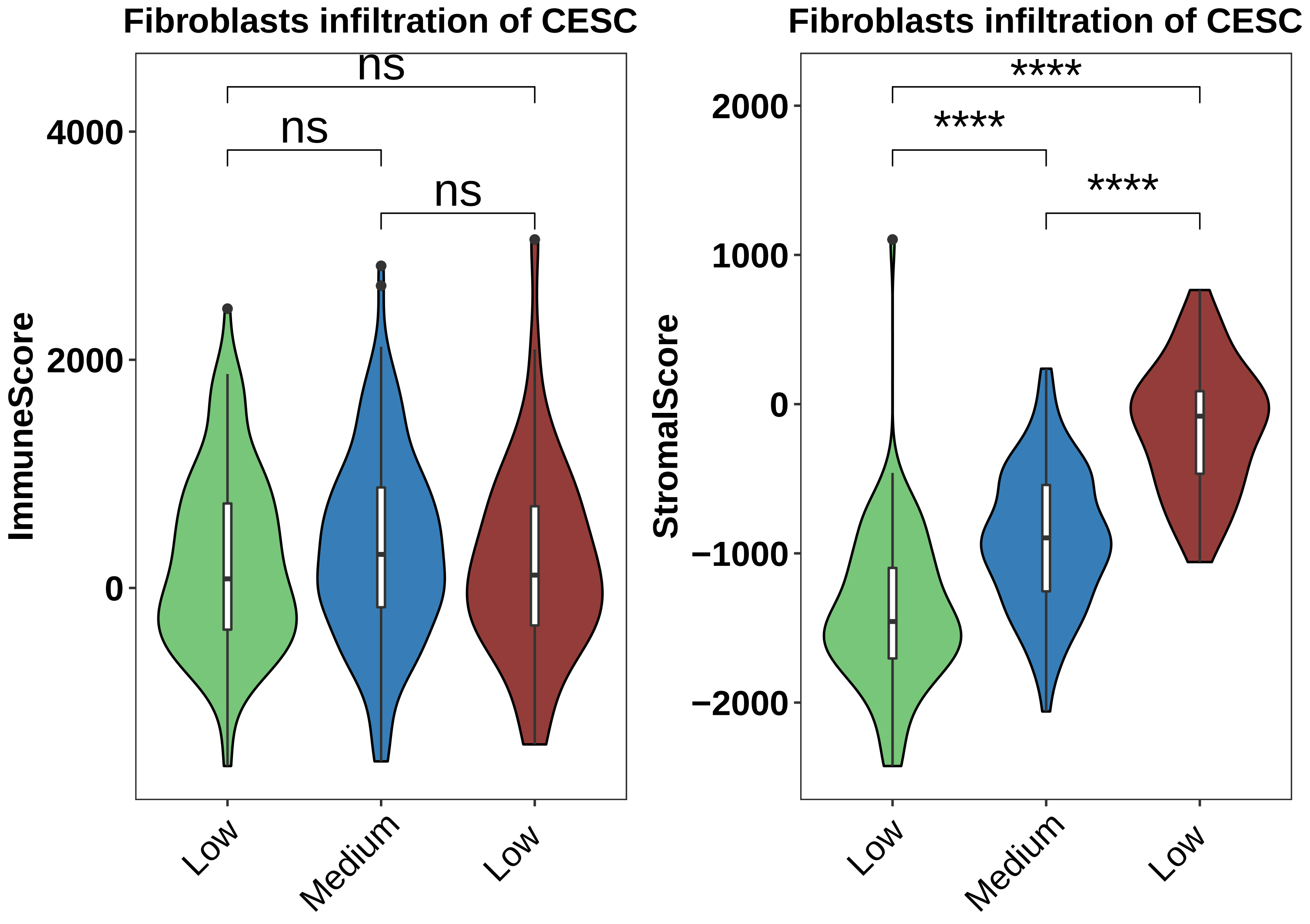
Fibroblasts infiltration of CESC
ImmuneScore
4000
2000
0
ns
ns
ns
Low
Medium
Low
Fibroblasts infiltration of CESC
StromalScore
2000
1000
0
−1000
−2000
****
****
****
Low
Medium
Low

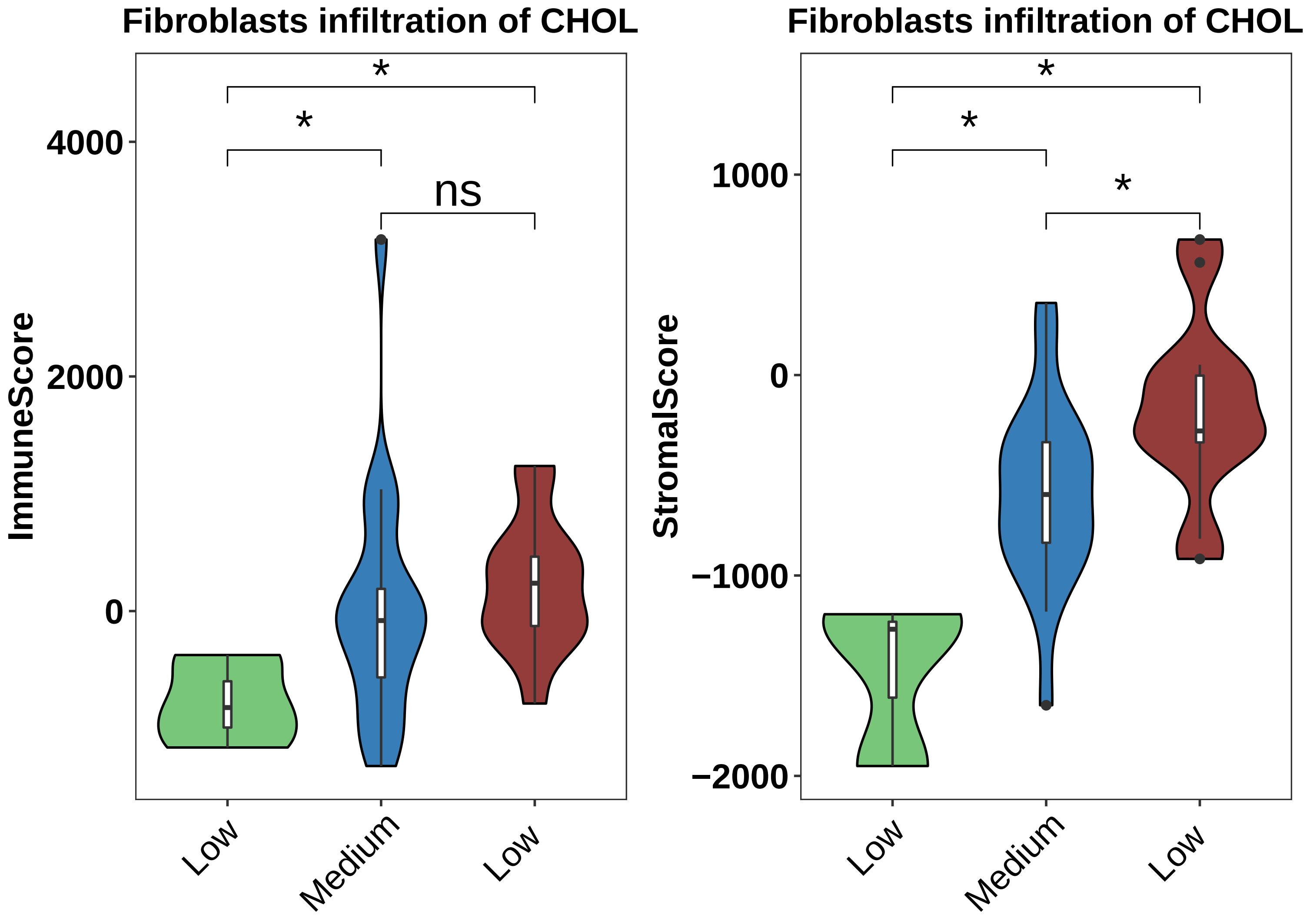

Fibroblasts infiltration of CHOL
ImmuneScore
4000
2000
0
*
*
ns
Low
Medium
Low
Fibroblasts infiltration of CHOL
StromalScore
1000
0
−1000
−2000
*
*
*
Low
Medium
Low

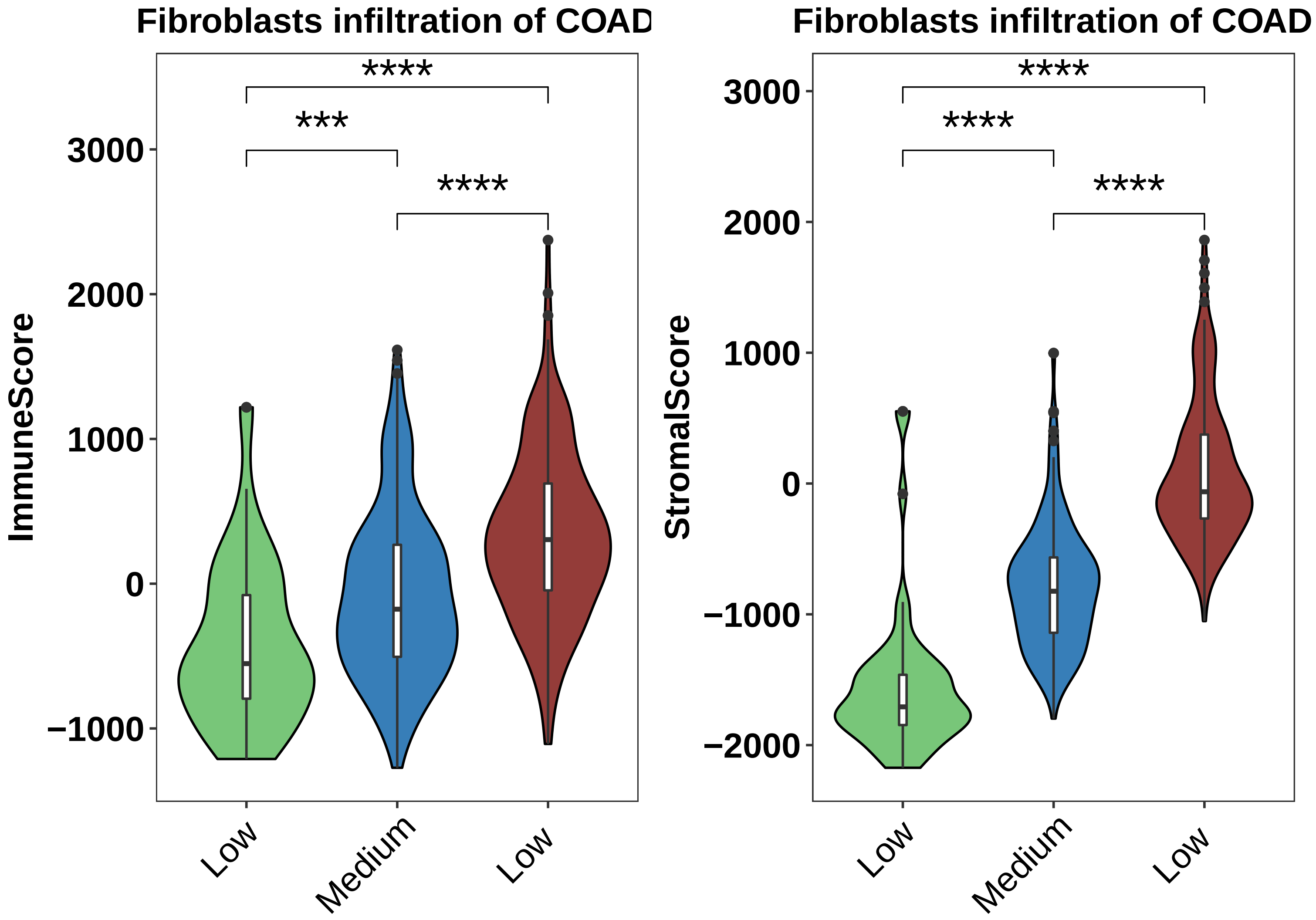
Fibroblasts infiltration of COAD
ImmuneScore
3000
2000
1000
0
−1000
****
***
****
Low
Medium
Low
Fibroblasts infiltration of COAD
StromalScore
3000
2000
1000
0
−1000
−2000
****
****
****
Low
Medium
Low

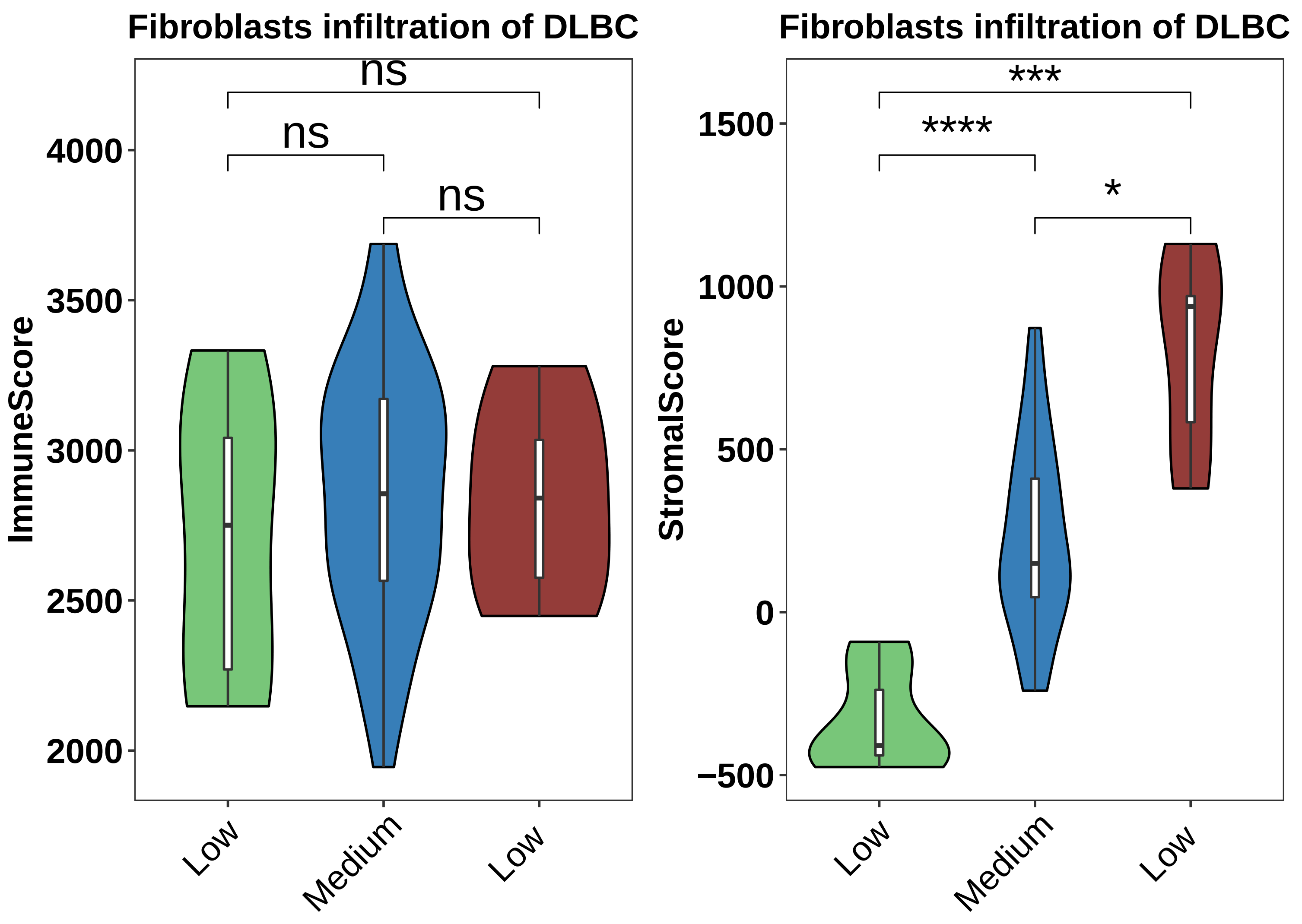

Fibroblasts infiltration of DLBC
Fibroblasts infiltration of DLBC
ImmuneScore
StromalScore
ns
ns
ns
***
****
*
4000
3500
3000
2500
2000
1500
1000
500
0
−500
Low
Medium
Low
Low
Medium
Low

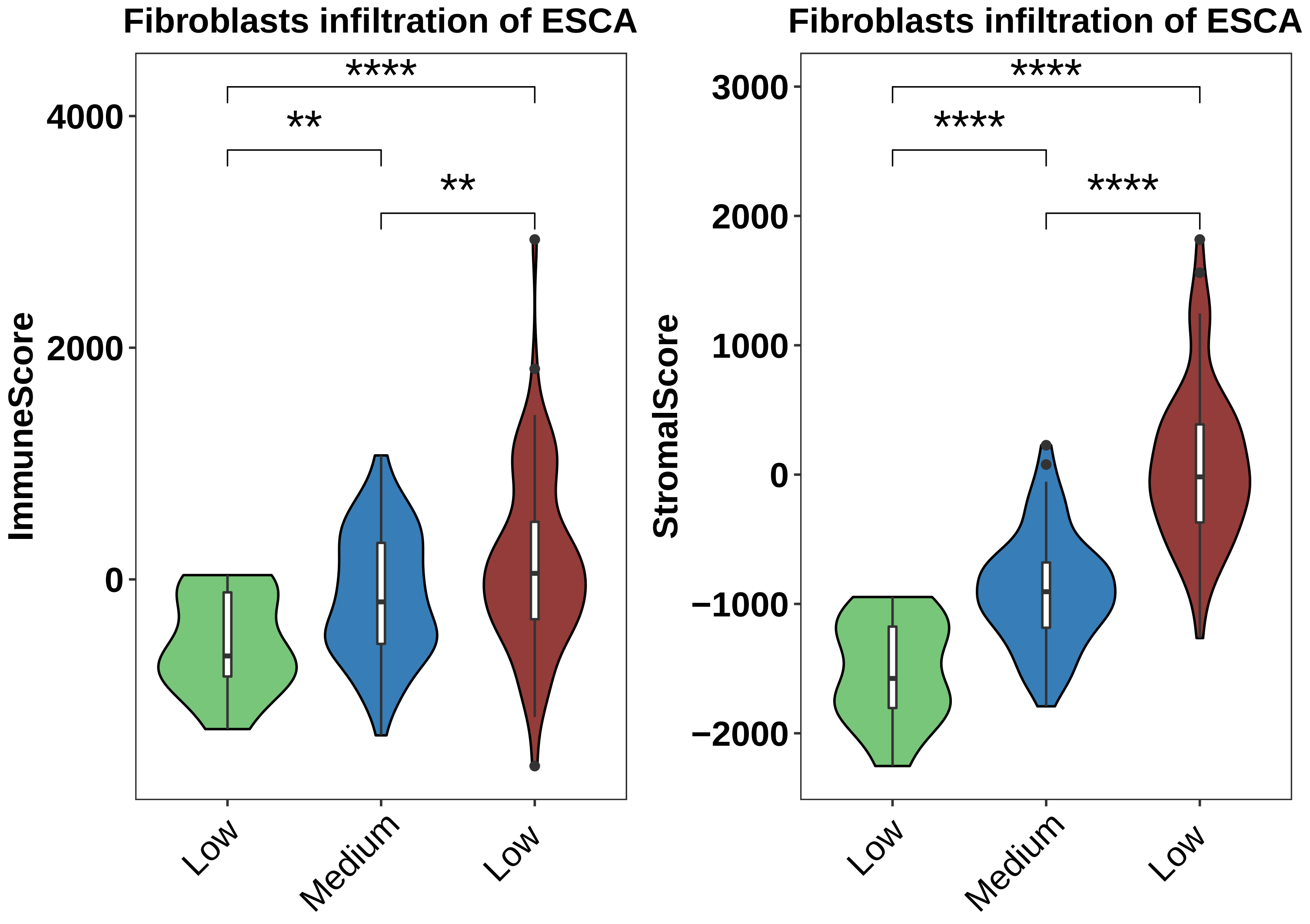

Fibroblasts infiltration of ESCA
ImmuneScore
4000
2000
0
****
**
**
Low
Medium
Low
Fibroblasts infiltration of ESCA
StromalScore
3000
2000
1000
0
−1000
−2000
****
****
****
Low
Medium
Low

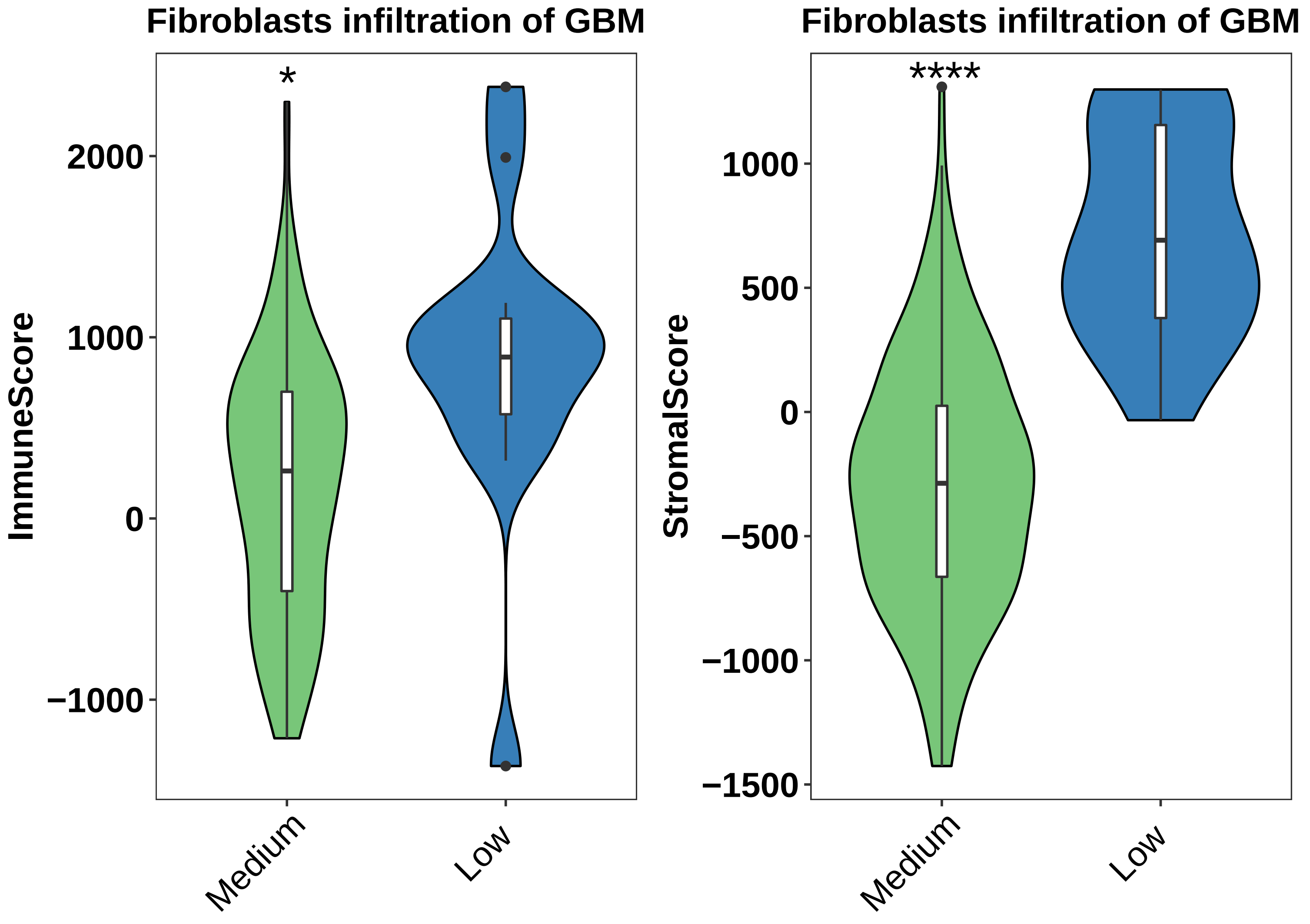

Fibroblasts infiltration of GBM
ImmuneScore
*
2000
1000
0
−1000
Medium
Low
Fibroblasts infiltration of GBM
StromalScore
****
1000
500
0
−500
−1000
−1500
Medium
Low

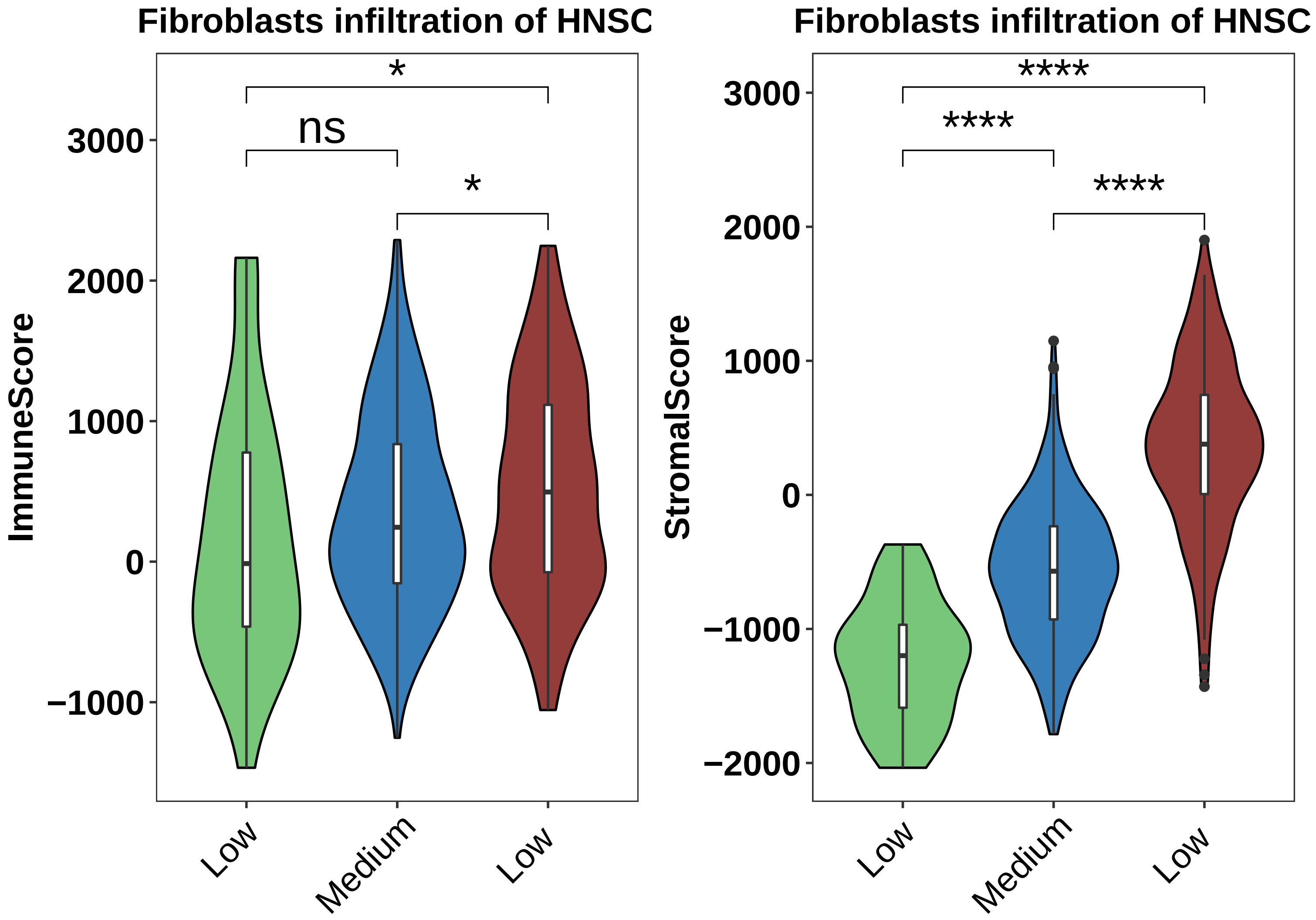
Fibroblasts infiltration of HNSC
ImmuneScore
3000
2000
1000
0
−1000
ns
*
*
Low
Medium
Low
Fibroblasts infiltration of HNSC
StromalScore
3000
2000
1000
0
−1000
−2000
****
****
****
Low
Medium
Low

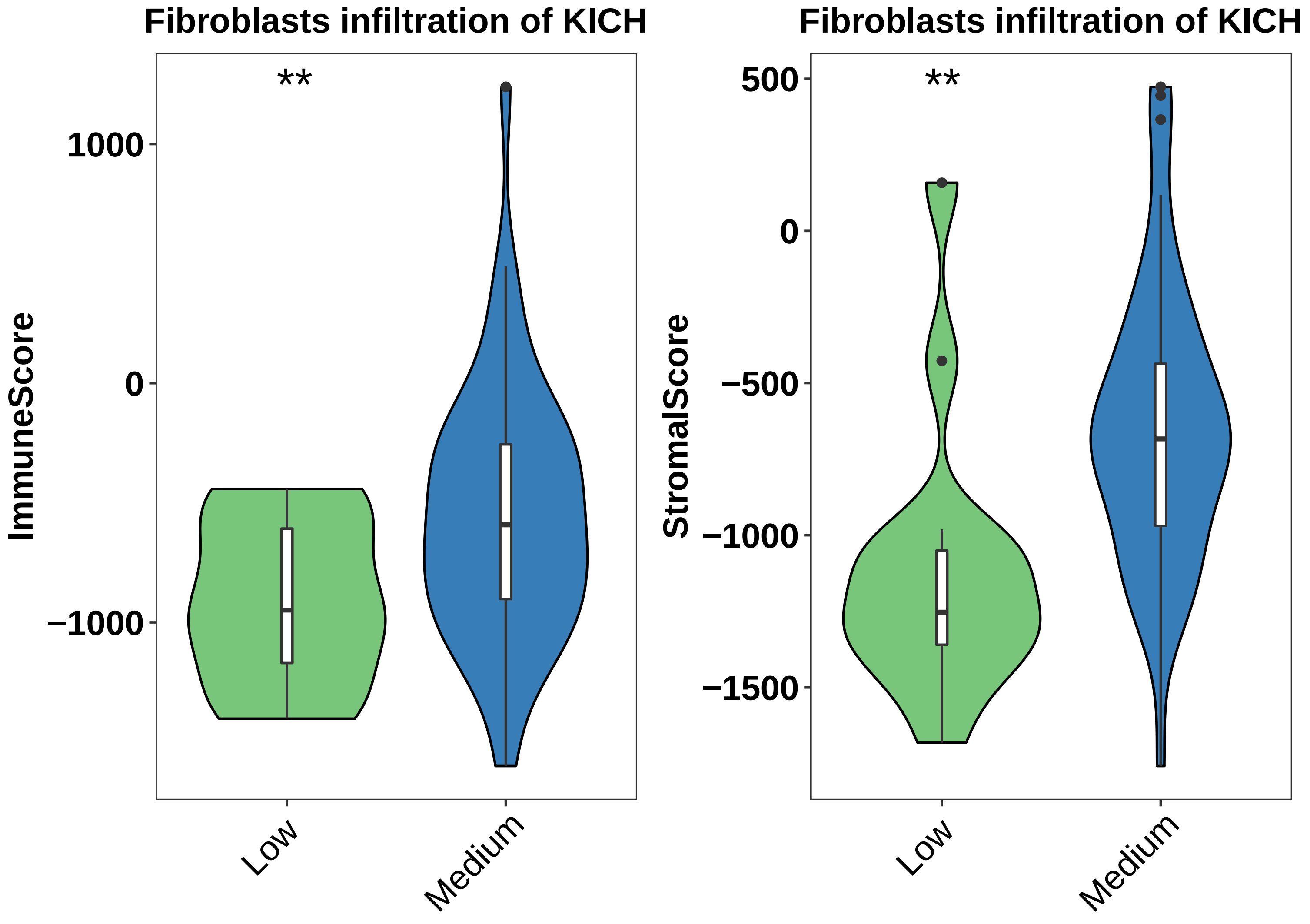

Fibroblasts infiltration of KICH
**
1000
0
−1000
ImmuneScore
Low
Medium
Fibroblasts infiltration of KICH
**
500
0
−500
−1000
−1500
StromalScore
Low
Medium

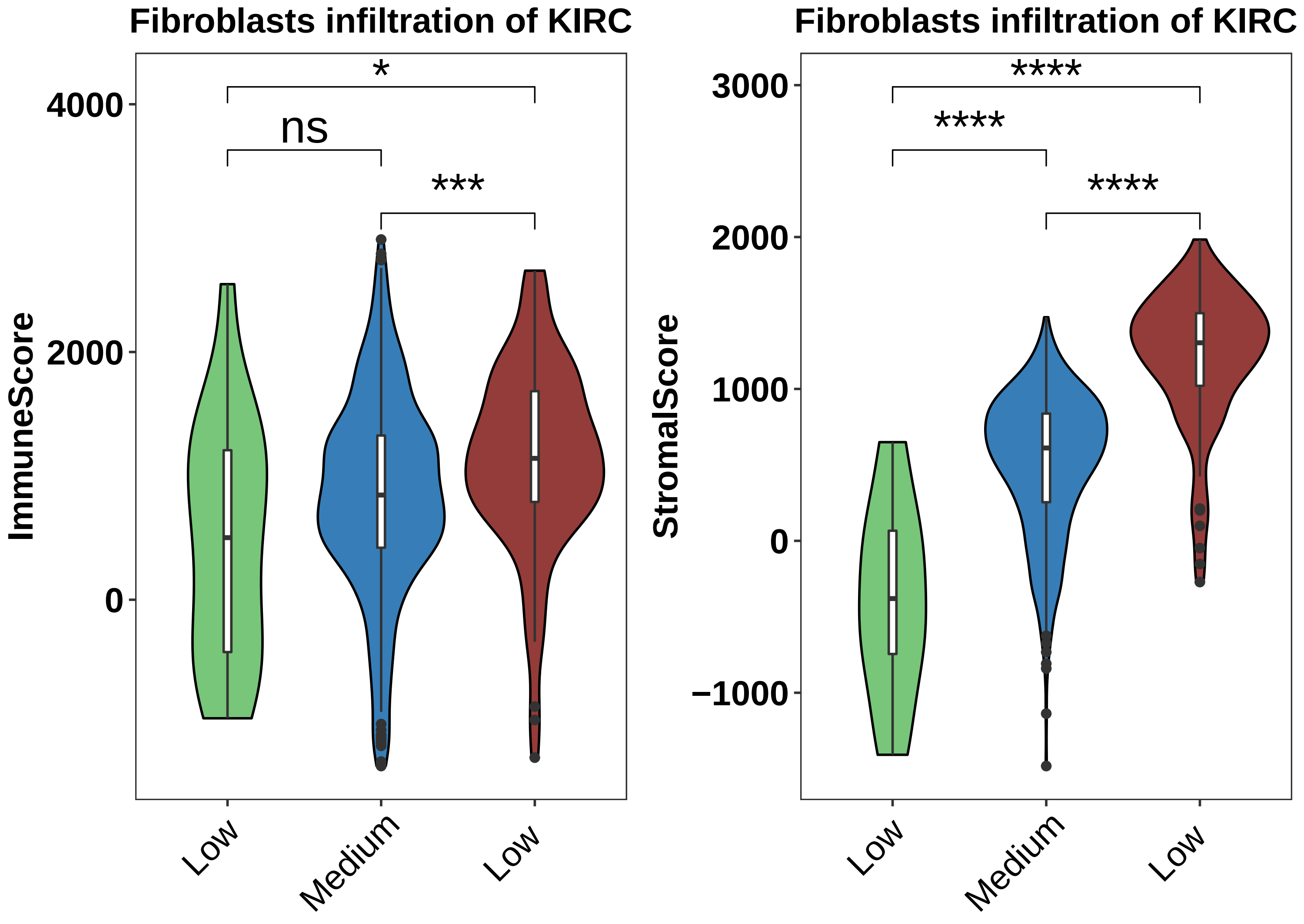

Fibroblasts infiltration of KIRC
ImmuneScore
4000
2000
0
*
ns
***
Low
Medium
Low
Fibroblasts infiltration of KIRC
StromalScore
3000
2000
1000
0
−1000
****
****
****
Low
Medium
Low

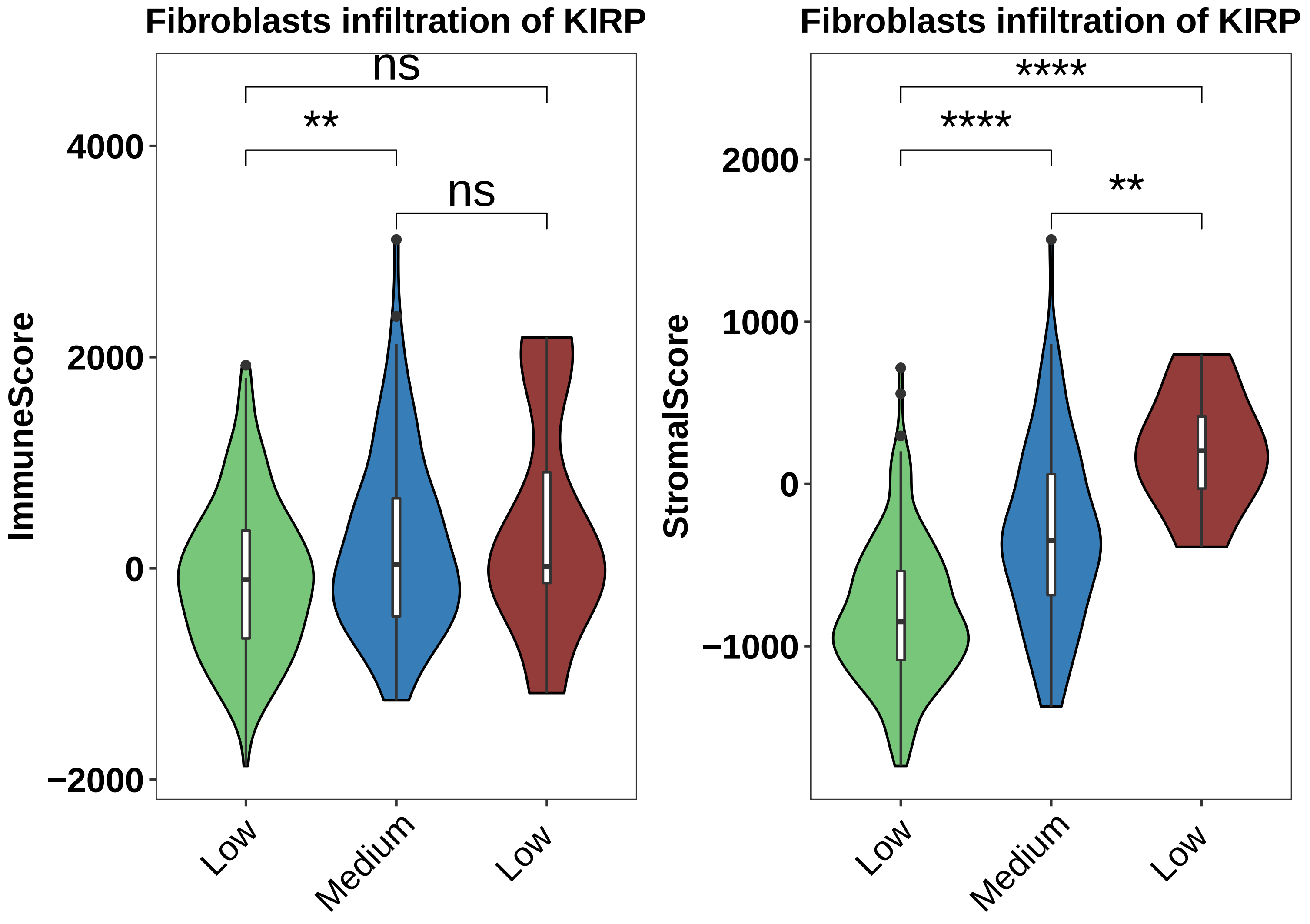

Fibroblasts infiltration of KIRP
ImmuneScore
4000
2000
0
−2000
ns
**
ns
Low
Medium
Low
Fibroblasts infiltration of KIRP
StromalScore
2000
1000
0
−1000
****
****
**
Low
Medium
Low

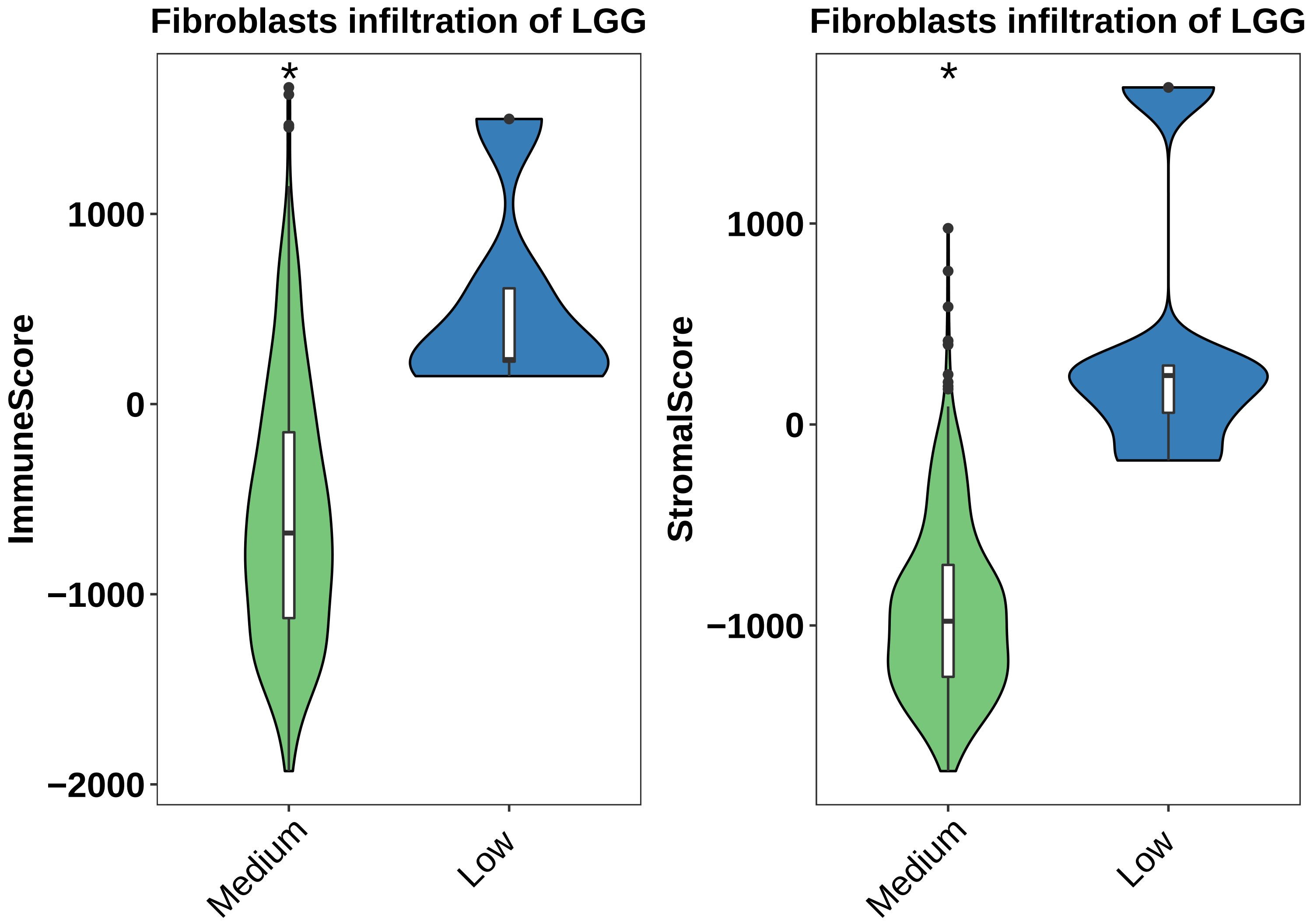
Fibroblasts infiltration of LGG
ImmuneScore
1000
0
−1000
−2000
*
Medium
Low
Fibroblasts infiltration of LGG
StromalScore
1000
0
−1000
*
Medium
Low

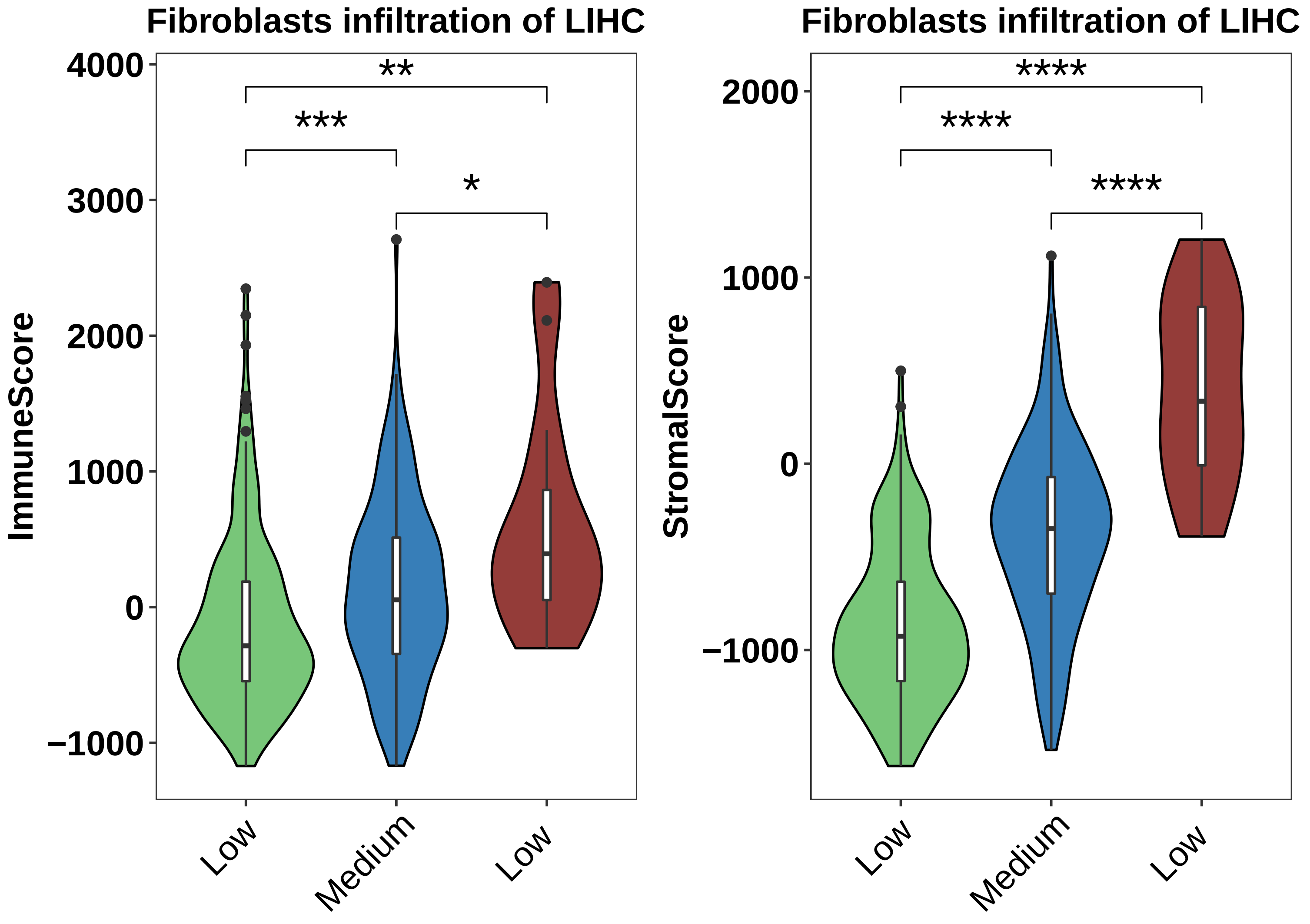

Fibroblasts infiltration of LIHC
ImmuneScore
4000
3000
2000
1000
0
−1000
**
***
*
Low
Medium
Low
Fibroblasts infiltration of LIHC
StromalScore
2000
1000
0
−1000
****
****
****
Low
Medium
Low

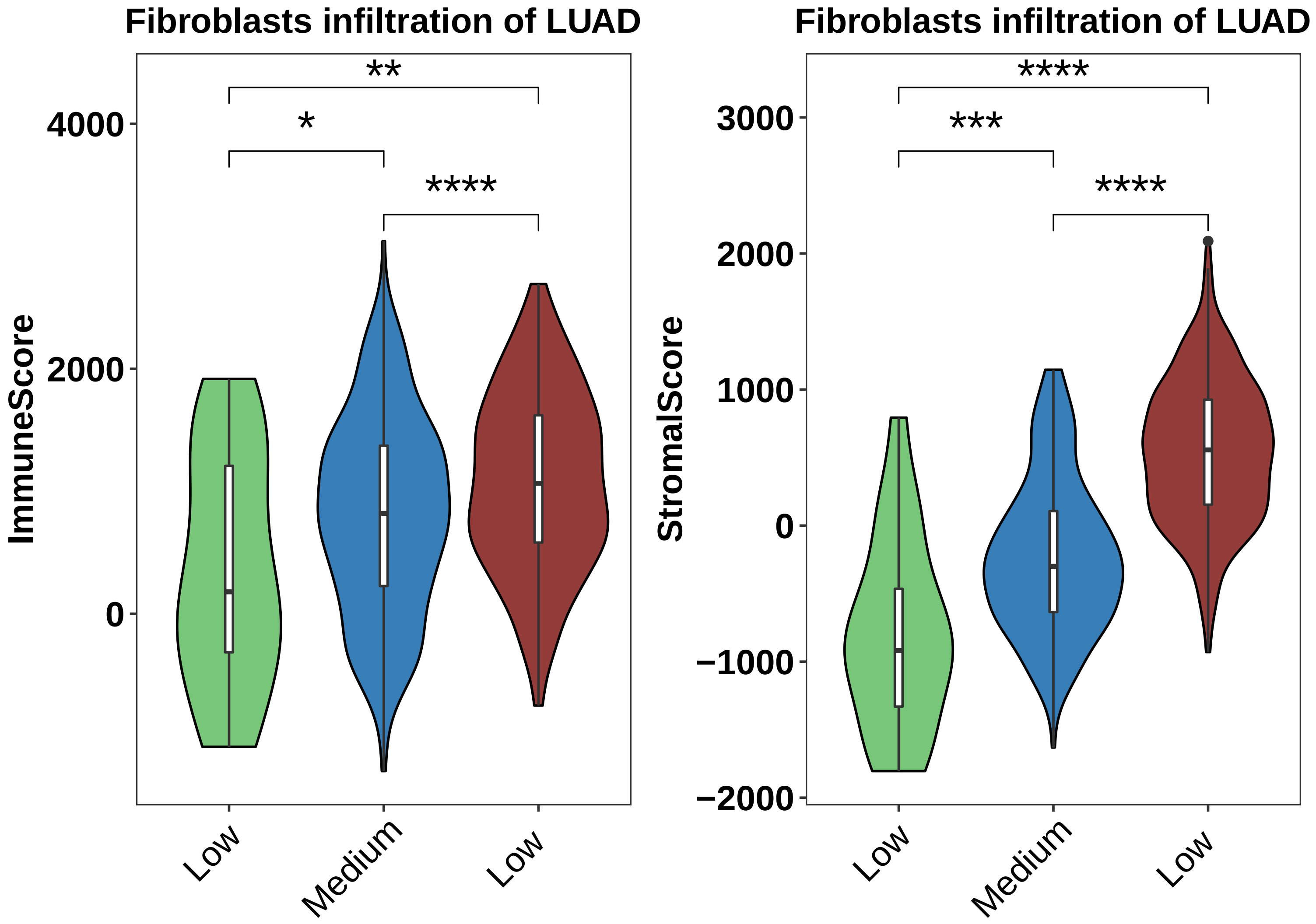
Fibroblasts infiltration of LUAD
ImmuneScore
4000
2000
0
**
*
****
Low
Medium
Low
Fibroblasts infiltration of LUAD
StromalScore
3000
2000
1000
0
−1000
−2000
****
***
****
Low
Medium
Low

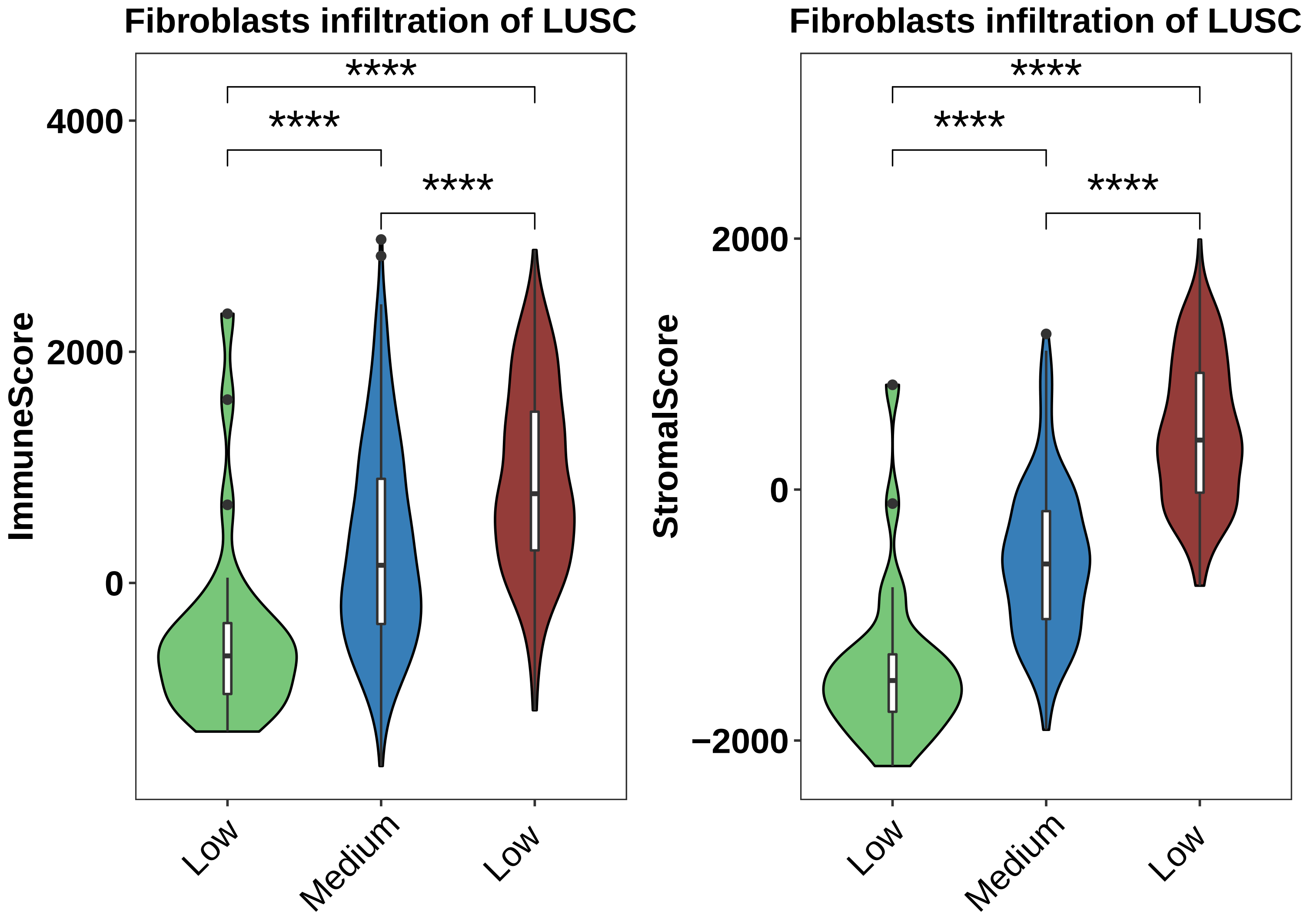

Fibroblasts infiltration of LUSC
ImmuneScore
4000
2000
0
****
****
****
Low
Medium
Low
Fibroblasts infiltration of LUSC
StromalScore
2000
0
−2000
****
****
****
Low
Medium
Low

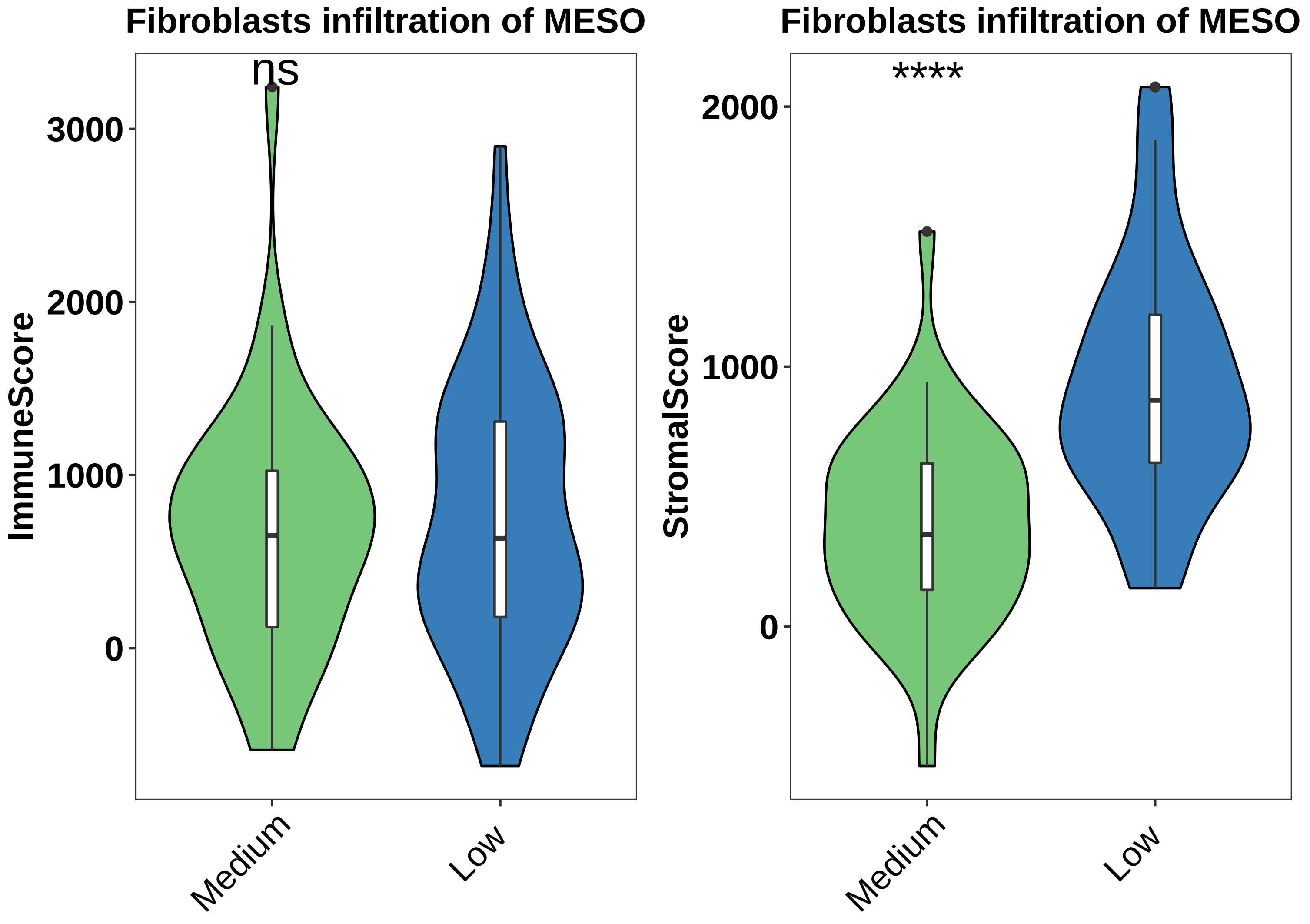

Fibroblasts infiltration of MESO
ns
ImmuneScore
3000
2000
1000
0
Medium
Low
Fibroblasts infiltration of MESO
****
StromalScore
2000
1000
0
Medium
Low

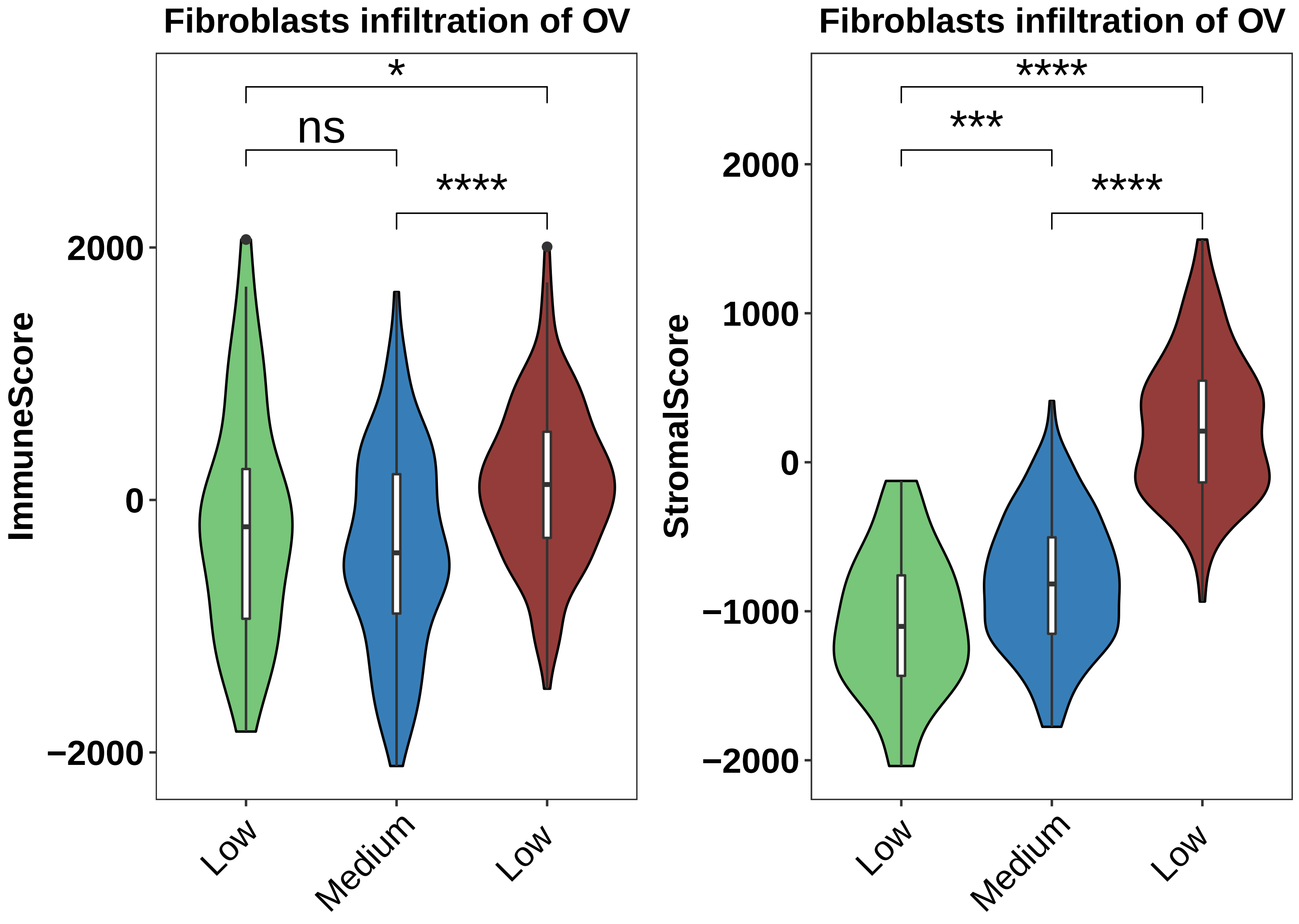

Fibroblasts infiltration of OV
ImmuneScore
2000
0
−2000
*
ns
****
Low
Medium
Low
Fibroblasts infiltration of OV
StromalScore
2000
1000
0
−1000
−2000
****
***
****
Low
Medium
Low

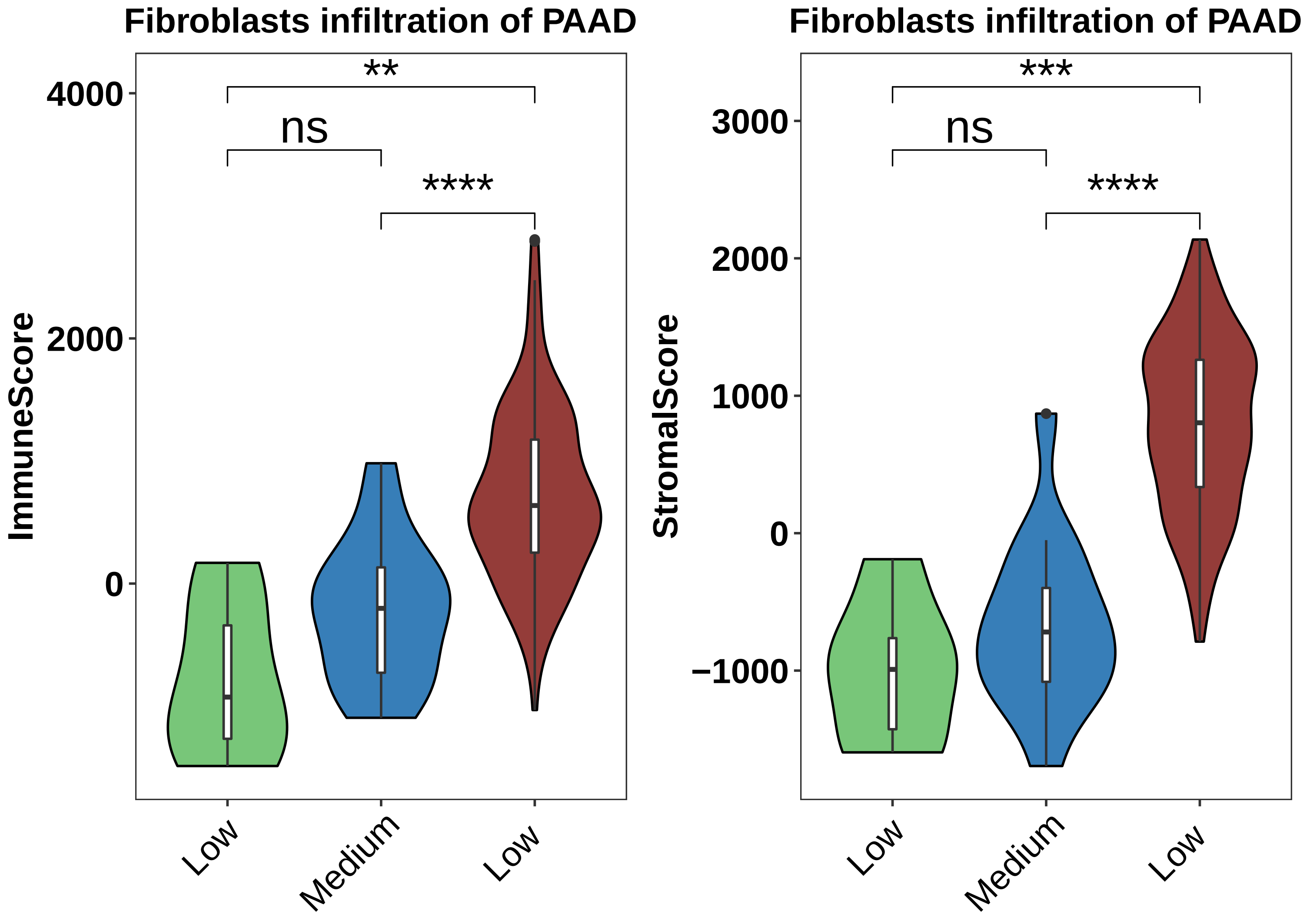

Fibroblasts infiltration of PAAD
ImmuneScore
4000
2000
0
**
ns
****
Low
Medium
Low
Fibroblasts infiltration of PAAD
StromalScore
3000
2000
1000
0
−1000
***
ns
****
Low
Medium
Low

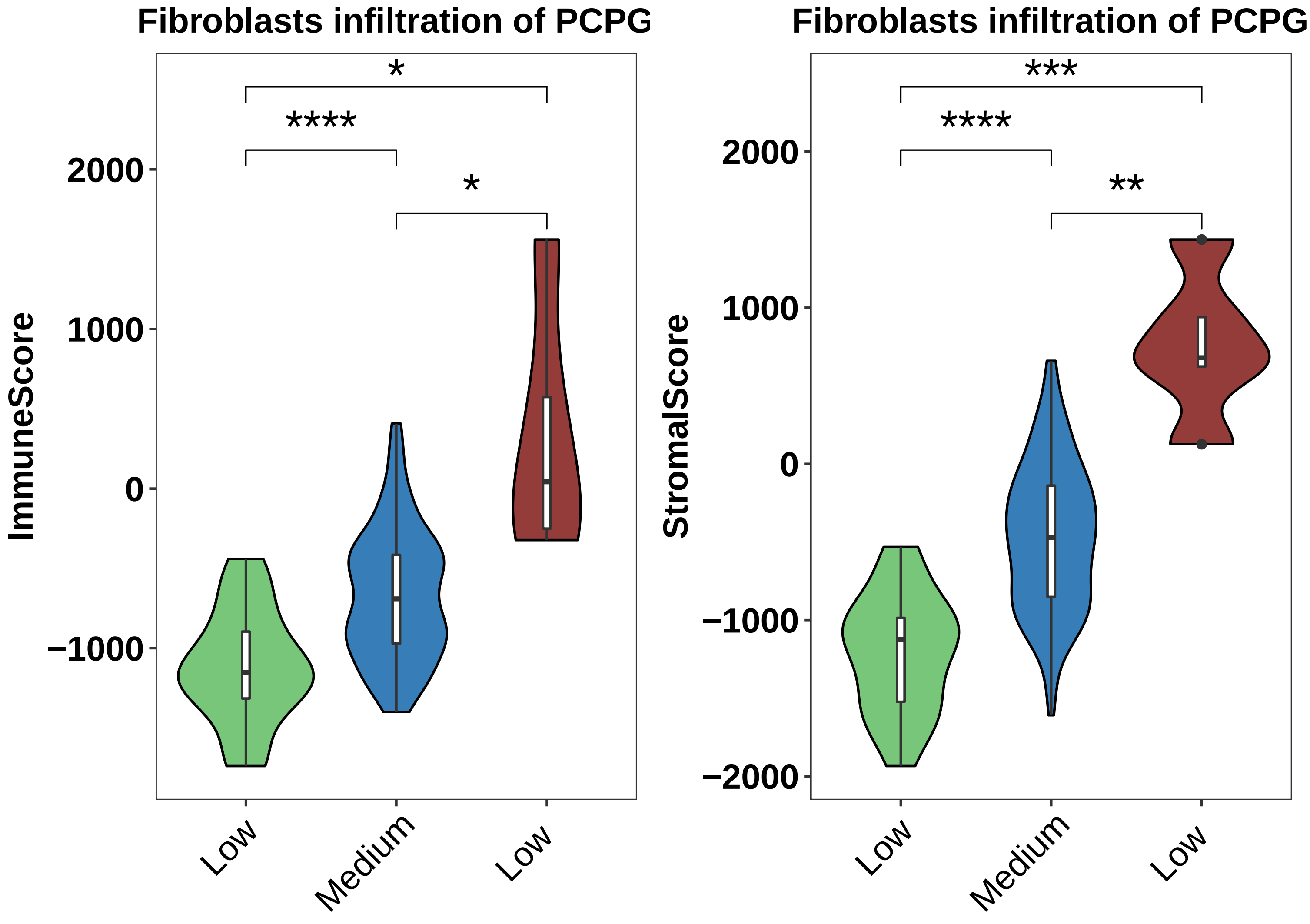

Fibroblasts infiltration of PCPG
ImmuneScore
2000
1000
0
−1000
*
****
*
Low
Medium
Low
Fibroblasts infiltration of PCPG
StromalScore
2000
1000
0
−1000
−2000
***
****
**
Low
Medium
Low

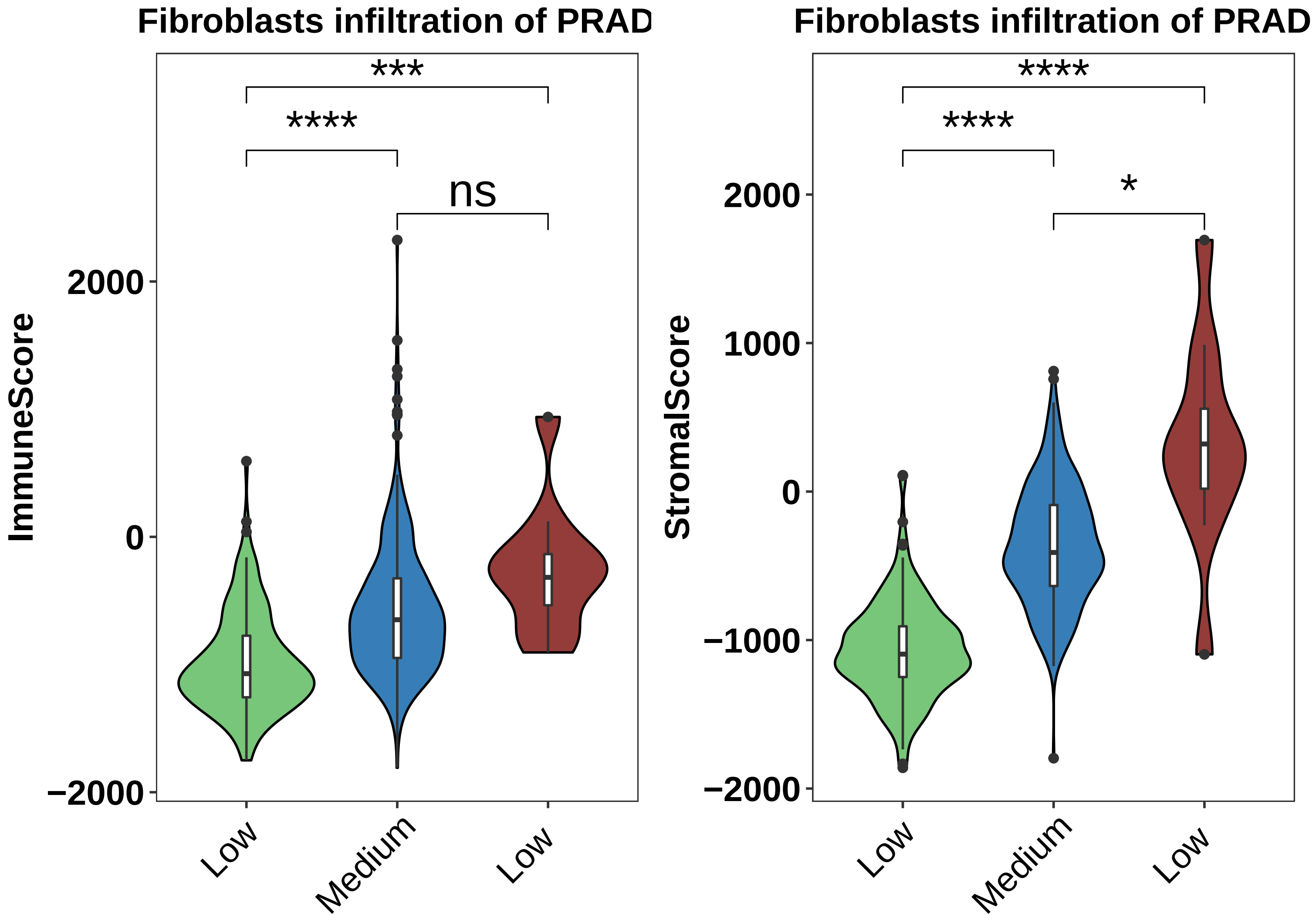

Fibroblasts infiltration of PRAD
ImmuneScore
***
****
ns
2000
0
−2000
Low
Medium
Low
Fibroblasts infiltration of PRAD
StromalScore
****
****
*
2000
1000
0
−1000
−2000
Low
Medium
Low

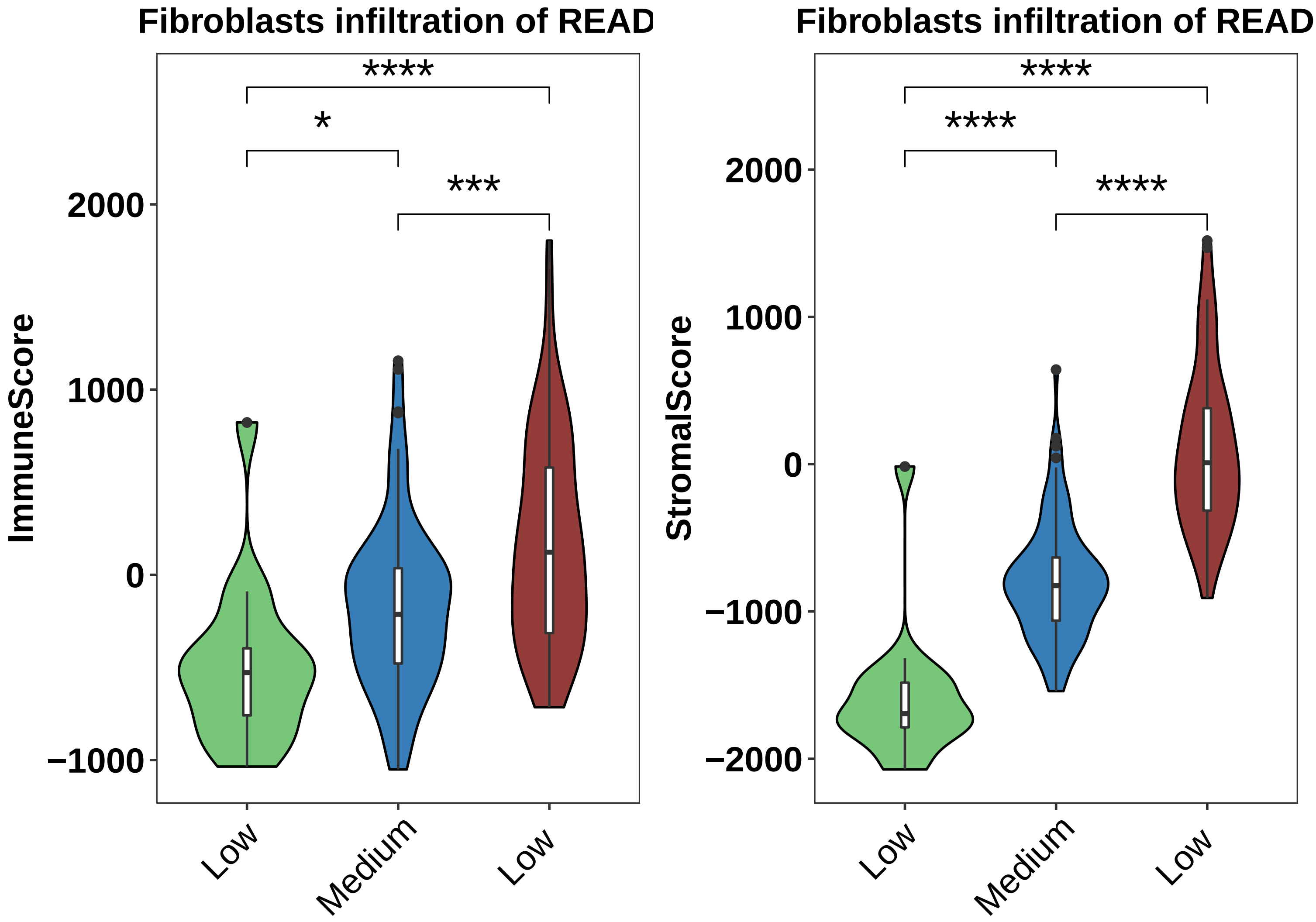

Fibroblasts infiltration of READ
ImmuneScore
2000
1000
0
−1000
****
*
***
Low
Medium
Low
Fibroblasts infiltration of READ
StromalScore
2000
1000
0
−1000
−2000
****
****
****
Low
Medium
Low

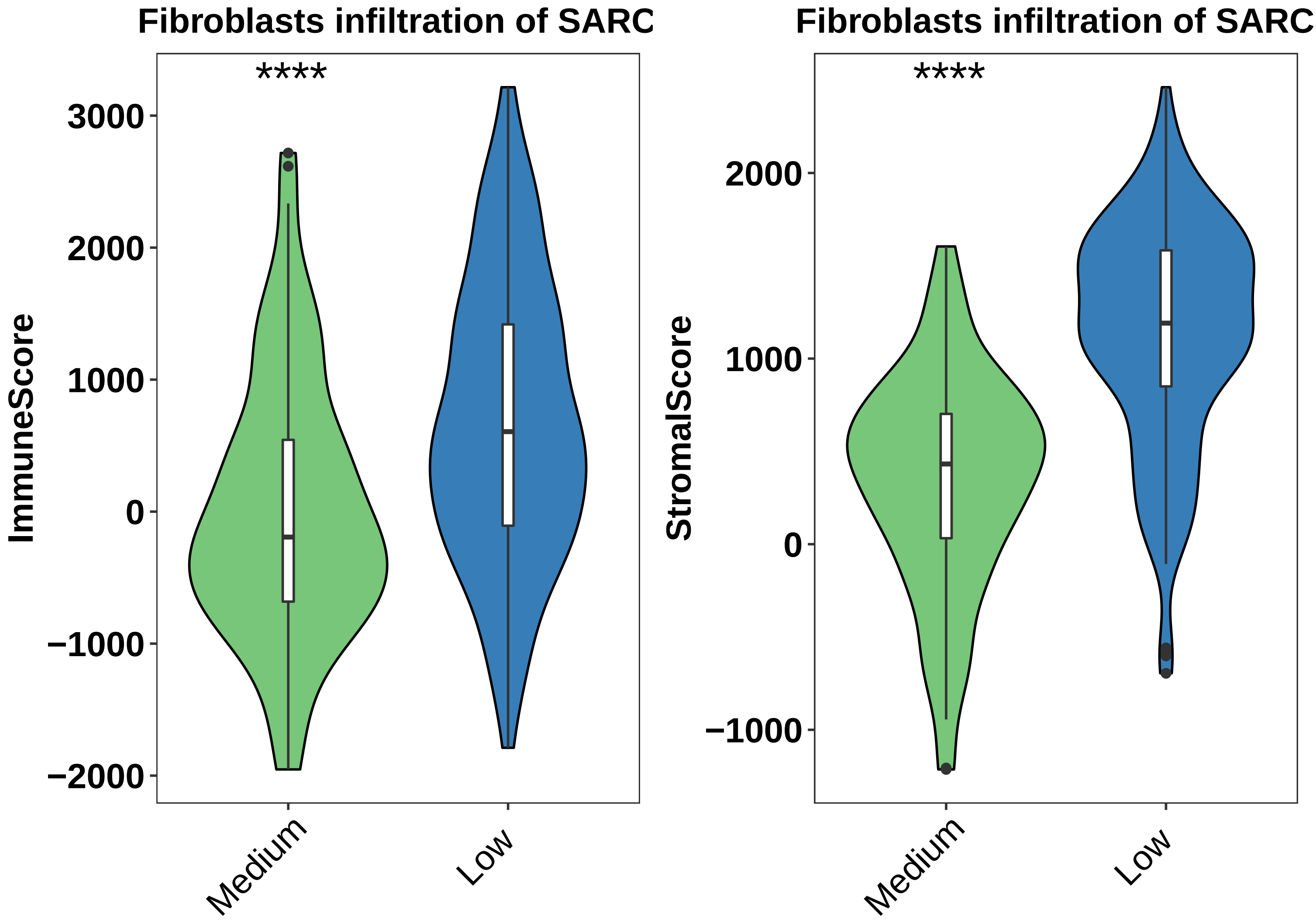

Fibroblasts infiltration of SARC
****
ImmuneScore
3000
2000
1000
0
−1000
−2000
Medium
Low
Fibroblasts infiltration of SARC
****
StromalScore
2000
1000
0
−1000
Medium
Low

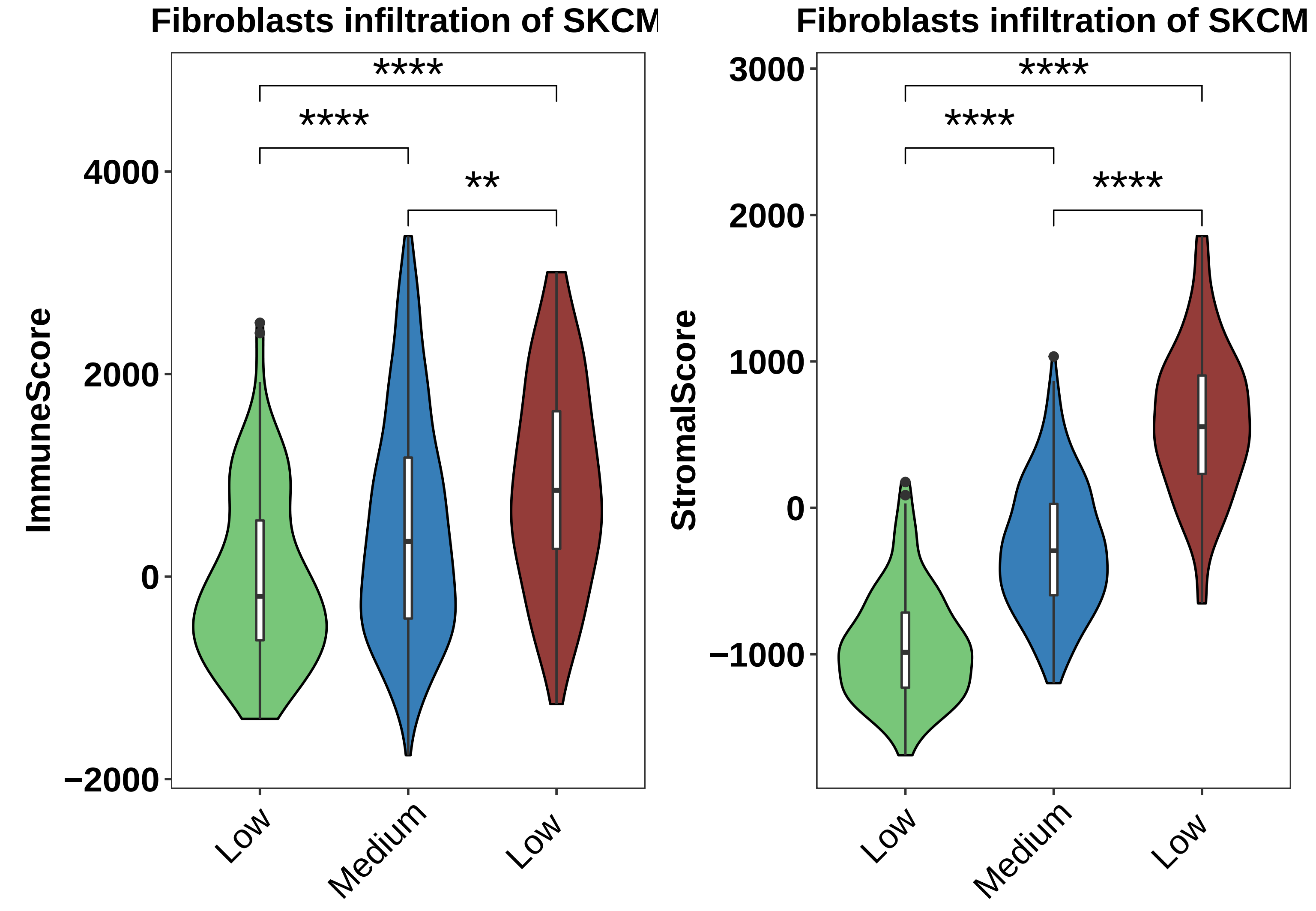

Fibroblasts infiltration of SKCM
ImmuneScore
4000
2000
0
−2000
****
****
**
Low
Medium
Low
Fibroblasts infiltration of SKCM
StromalScore
3000
2000
1000
0
−1000
****
****
****
Low
Medium
Low

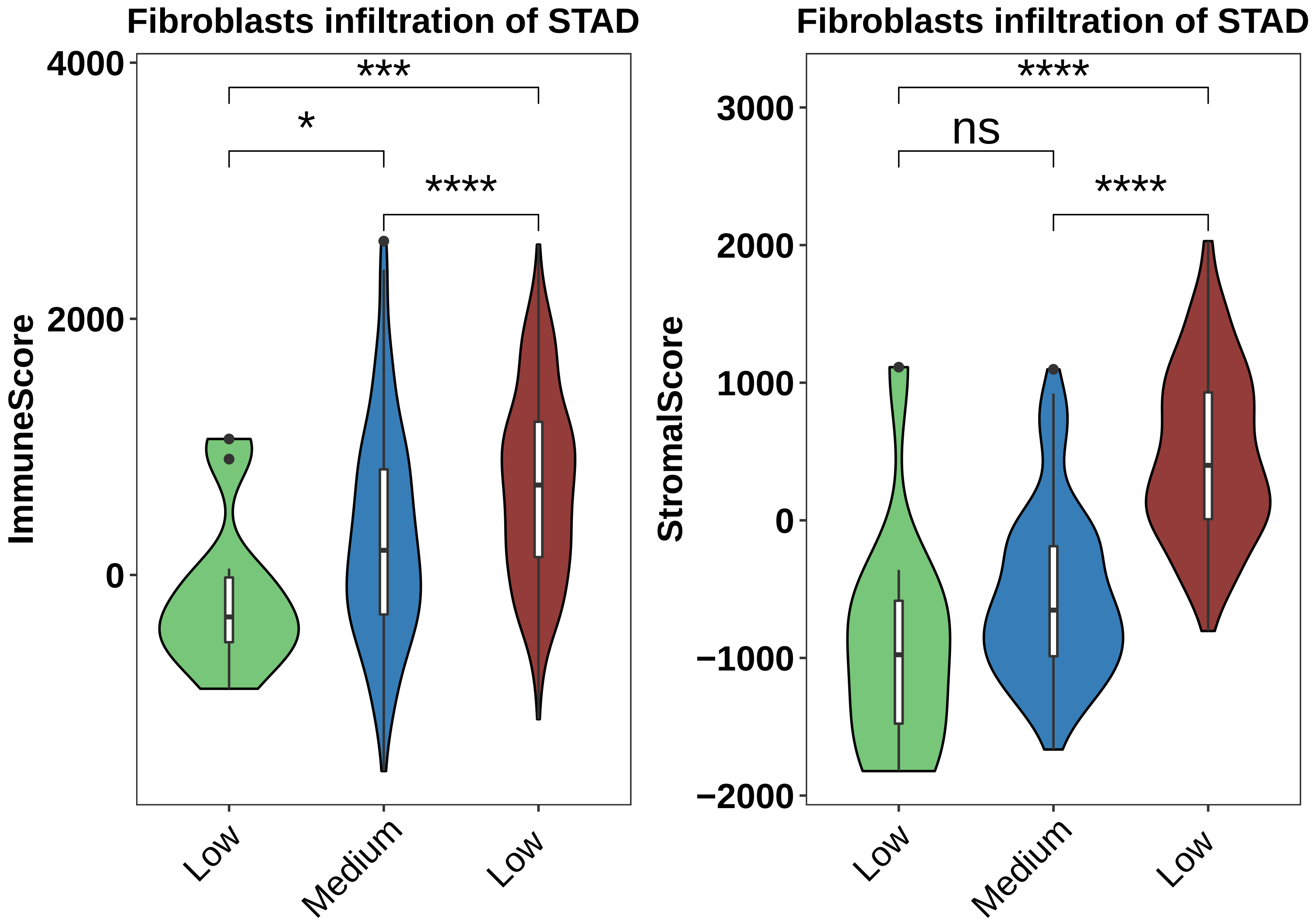

Fibroblasts infiltration of STAD
ImmuneScore
4000
2000
0
***
*
****
Low
Medium
Low
Fibroblasts infiltration of STAD
StromalScore
3000
2000
1000
0
−1000
−2000
****
ns
****
Low
Medium
Low

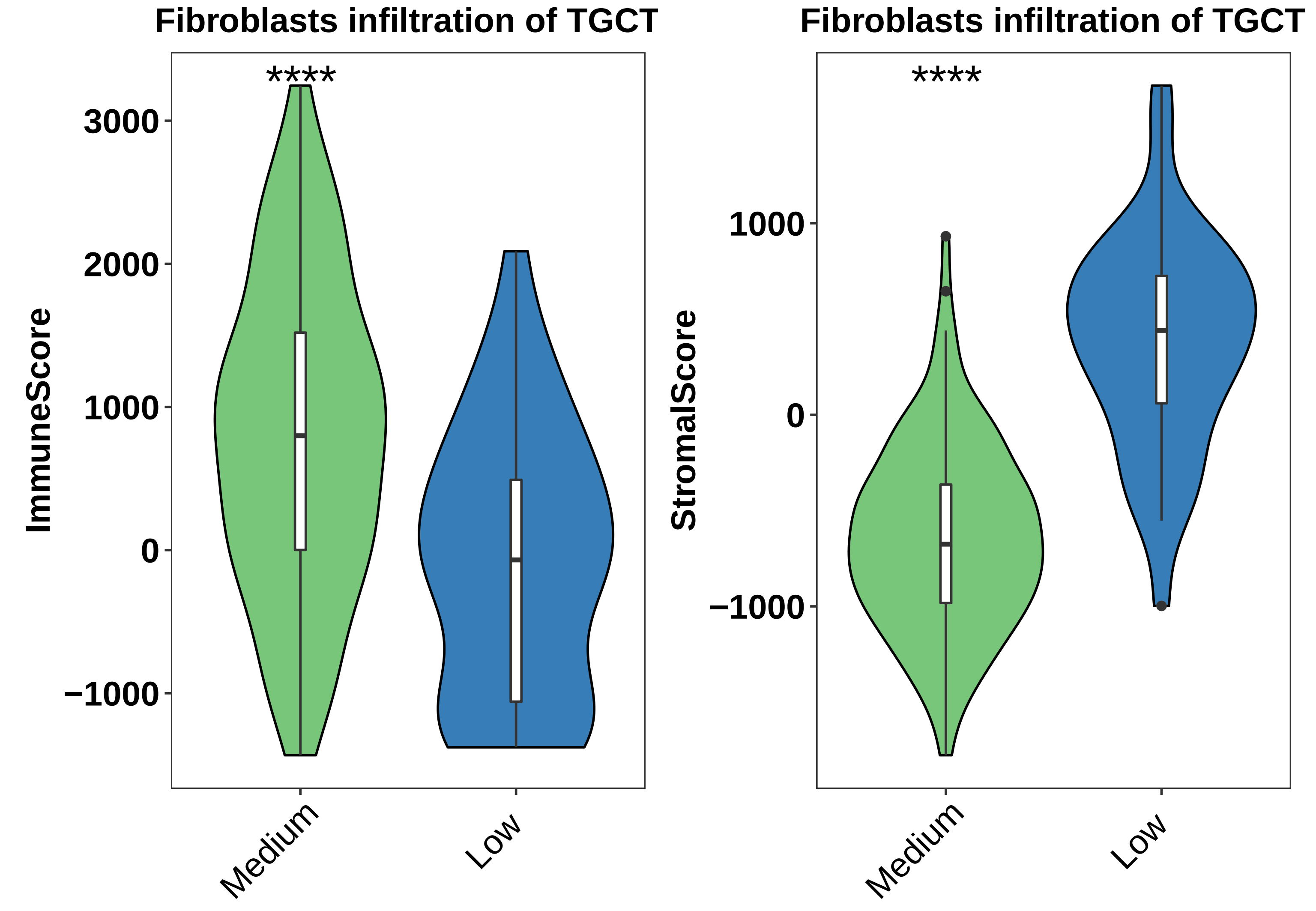
Fibroblasts infiltration of TGCT
****
ImmuneScore
3000
2000
1000
0
−1000
Medium
Low
Fibroblasts infiltration of TGCT
****
StromalScore
1000
0
−1000
Medium
Low

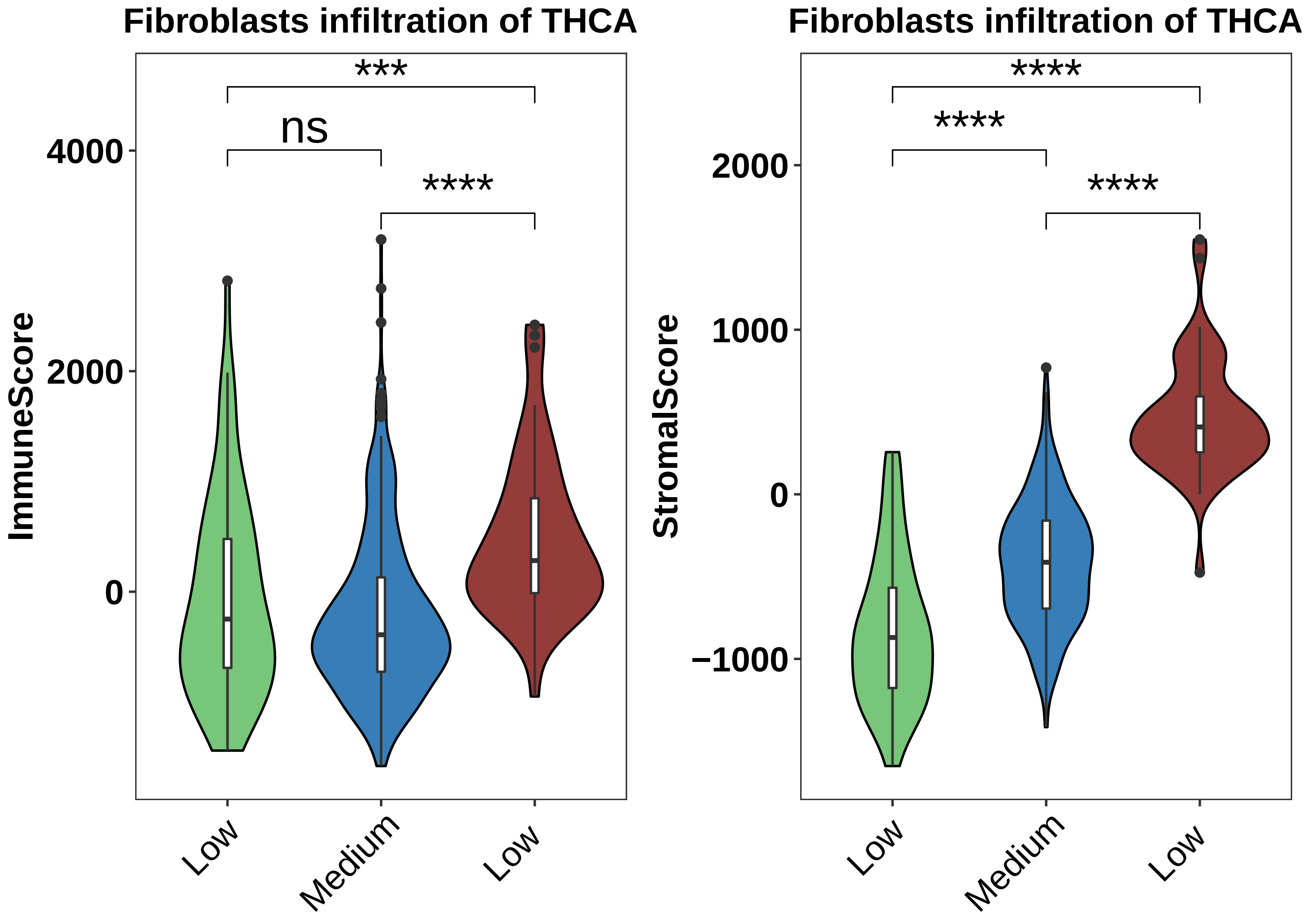
Fibroblasts infiltration of THCA
ImmuneScore
4000
2000
0
***
ns
****
Low
Medium
Low
Fibroblasts infiltration of THCA
StromalScore
2000
1000
0
−1000
****
****
****
Low
Medium
Low

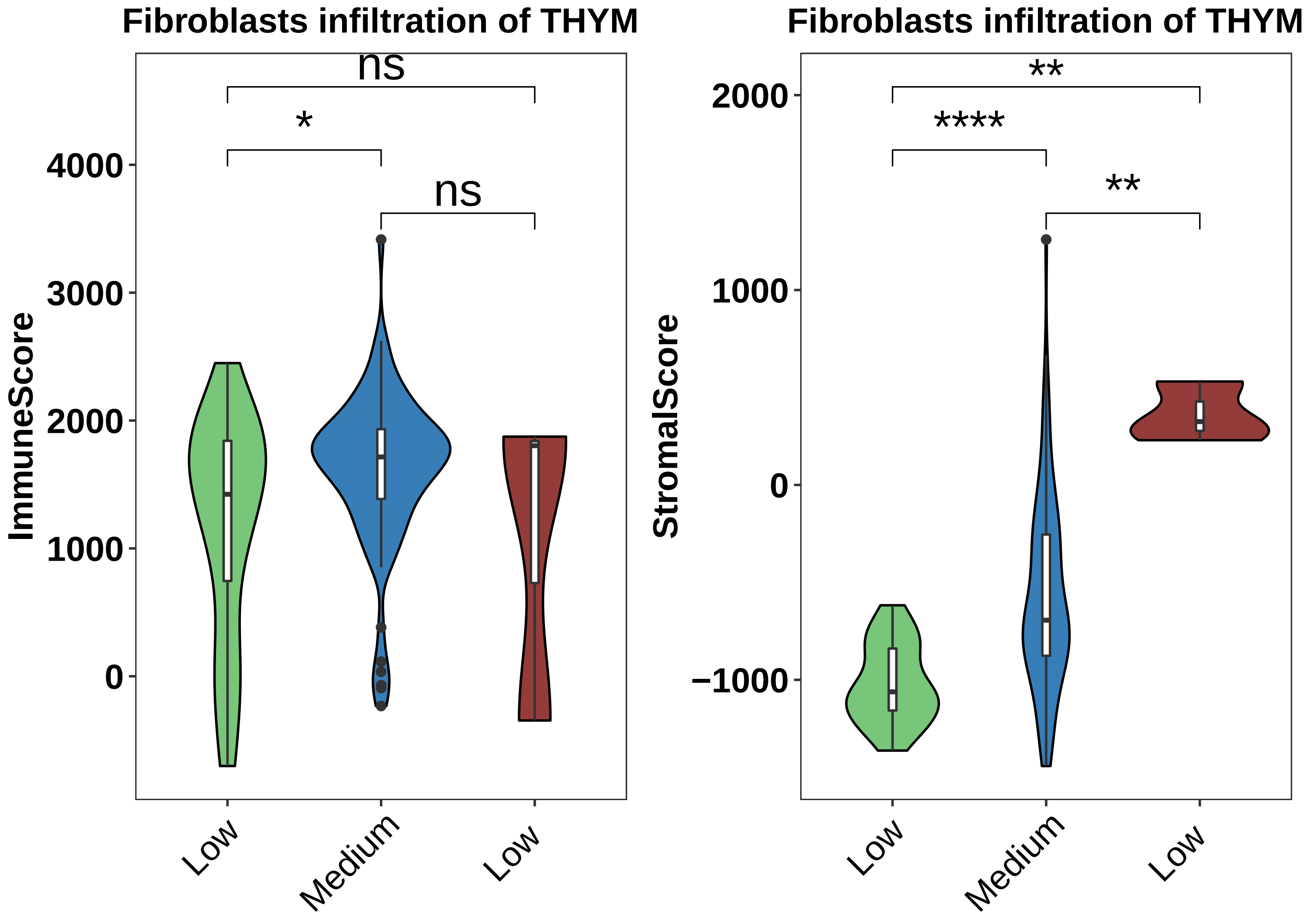
Fibroblasts infiltration of THYM
ImmuneScore
ns
*
ns
4000
3000
2000
1000
0
Low
Medium
Low
Fibroblasts infiltration of THYM
StromalScore
**
****
**
2000
1000
0
−1000
Low
Medium
Low

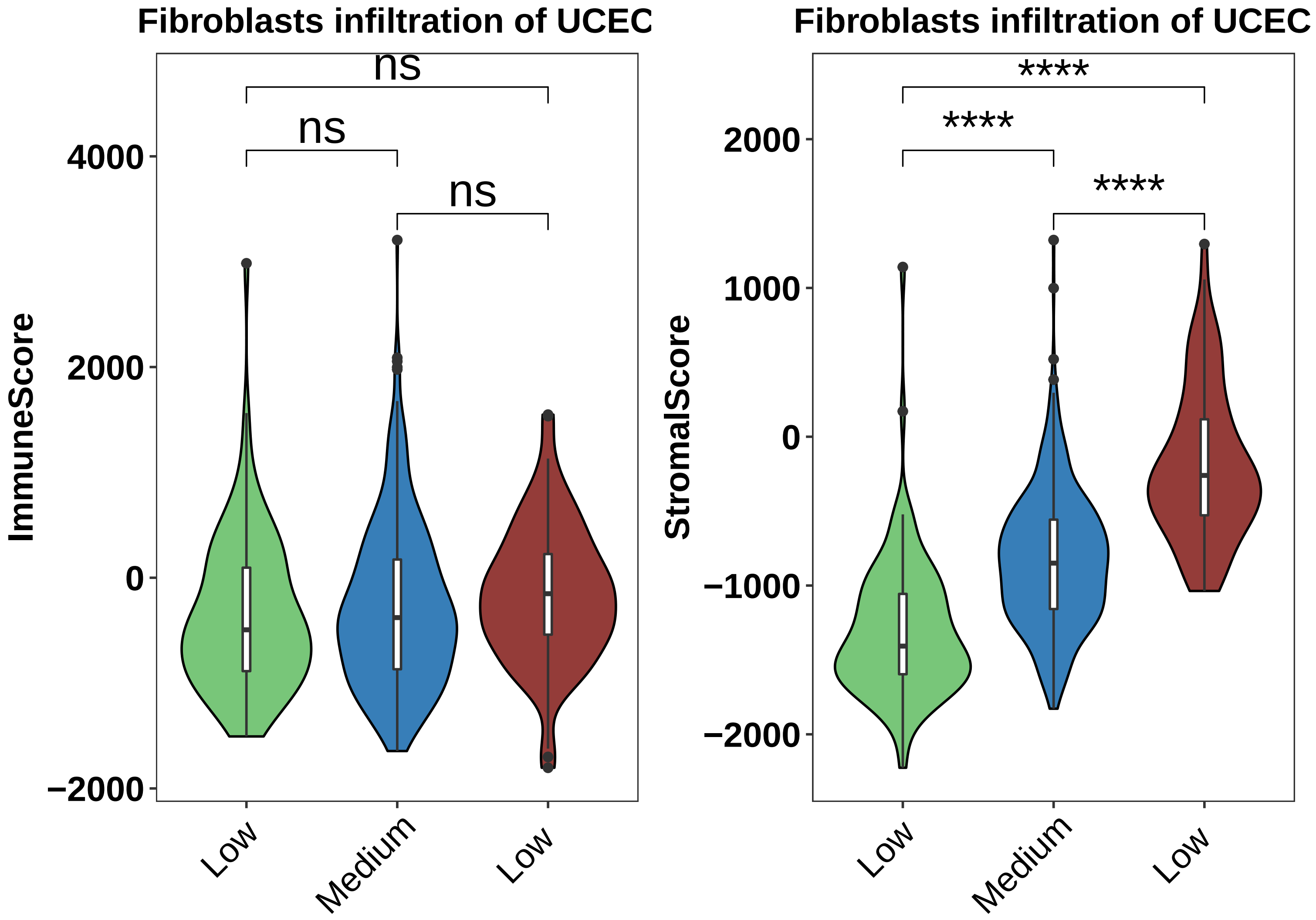

Fibroblasts infiltration of UCEC
ImmuneScore
ns
ns
ns
4000
2000
0
−2000
Low
Medium
Low
Fibroblasts infiltration of UCEC
StromalScore
****
****
****
2000
1000
0
−1000
−2000
Low
Medium
Low

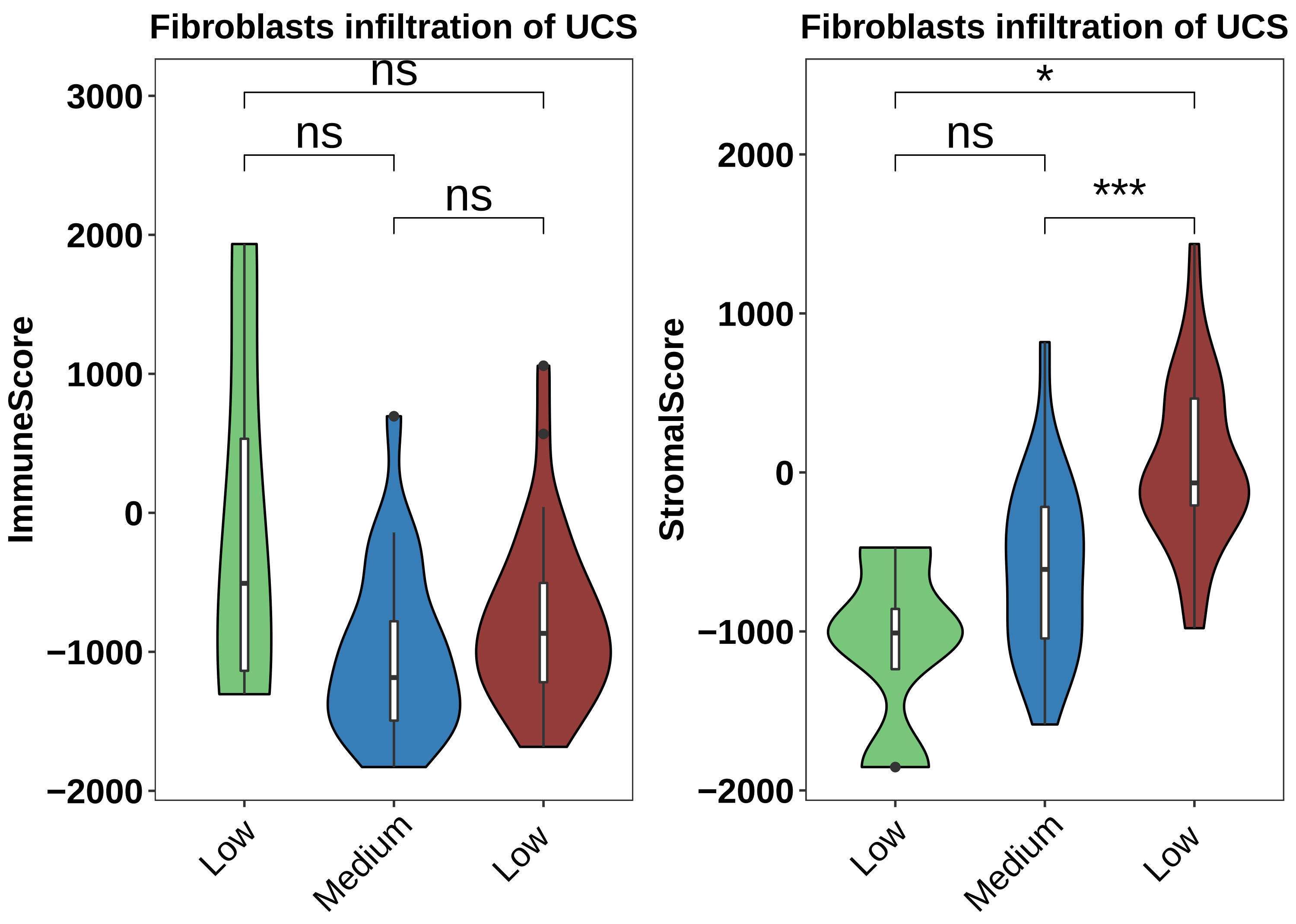

Fibroblasts infiltration of UCS
ImmuneScore
3000
2000
1000
0
−1000
−2000
ns
ns
ns
Low
Medium
Low
Fibroblasts infiltration of UCS
StromalScore
2000
1000
0
−1000
−2000
*
ns
***
Low
Medium
Low

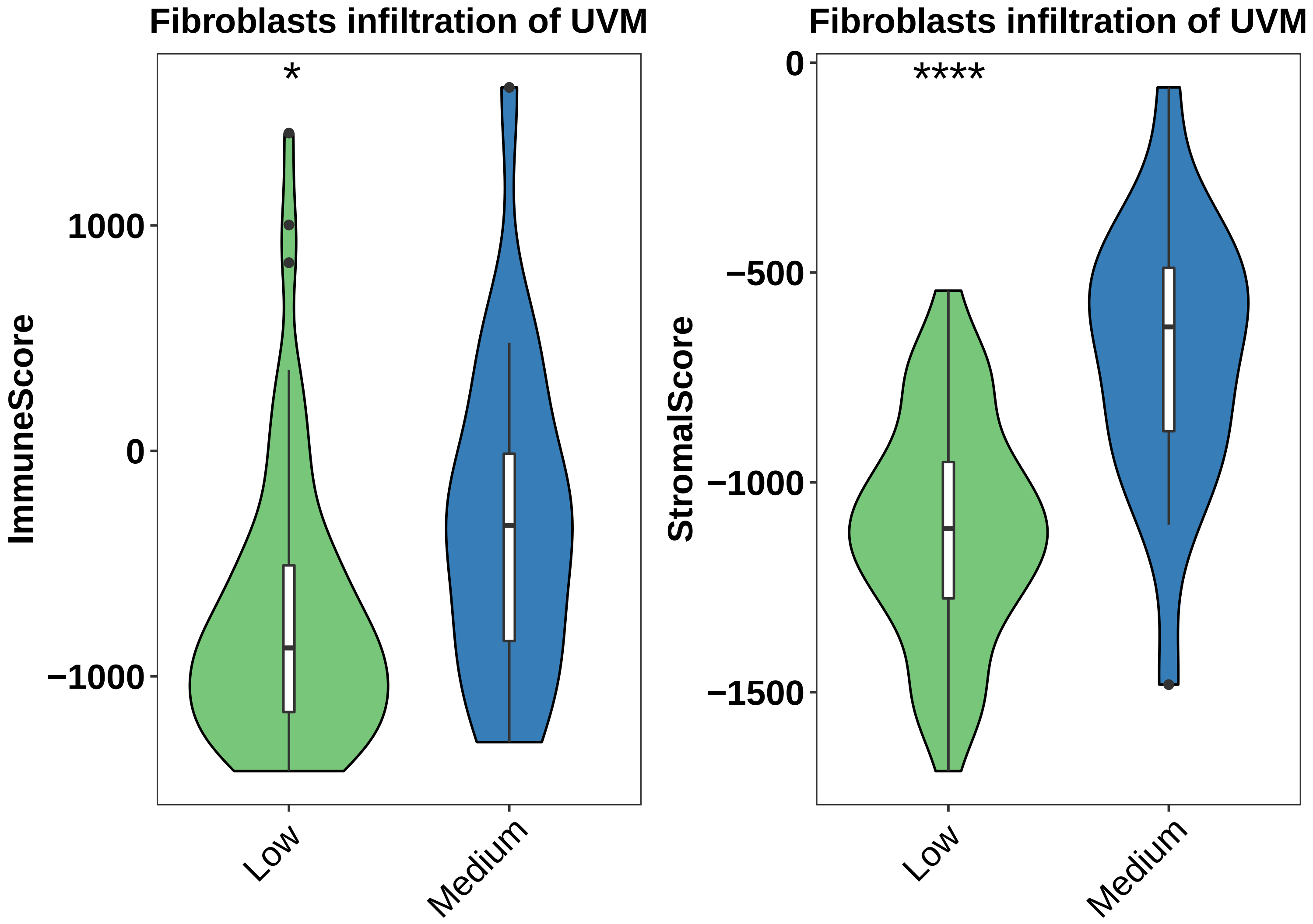
Fibroblasts infiltration of UVM
*
1000
0
−1000
ImmuneScore
Low
Medium
Fibroblasts infiltration of UVM
****
0
−500
−1000
−1500
StromalScore
Low
Medium
