# Supplementary material for: Dissecting the role of cancer‐associated fibroblast‐derived biglycan as a potential therapeutic target in immunotherapy resistance: A tumor bulk and single‐cell transcriptomic study
Source: Clin Transl Med. 2023 Feb 11;13(2):e1189. doi: 10.1002/ctm2.1189 (PMC9920016; doi:10.1002/ctm2.1189)
Supplement: Supplementary file 3 — Supporting Information [file CTM2-13-e1189-s001.pdf]

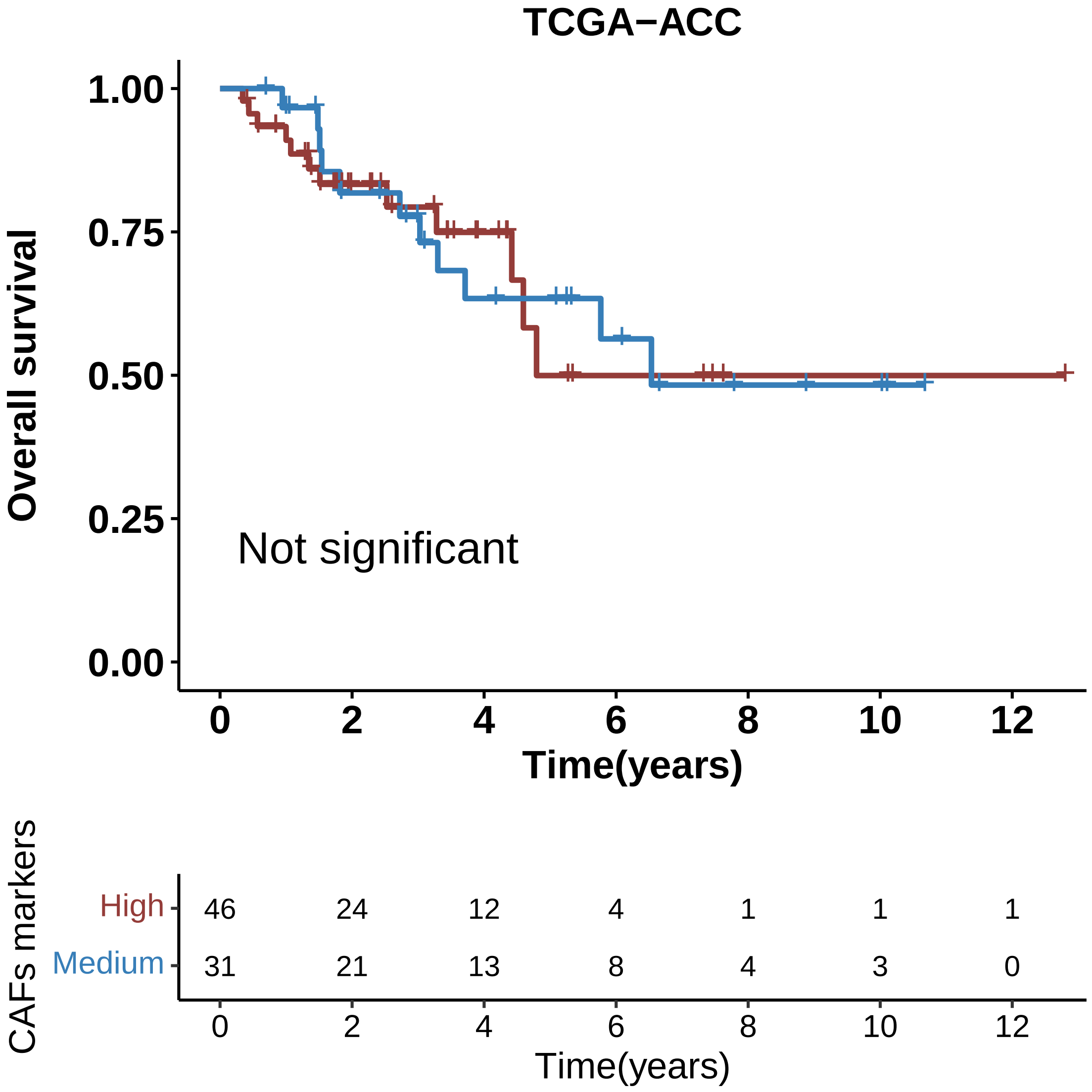
TCGA−ACC
Overall survival
1.00
0.75
0.50
0.25
0.00
Not significant
0
2
4
6
8
10
12
Time(years)
CAFs markers
High
Medium
46 24 12 4 1 1 1
31 21 13 8 4 3 0
Time(years)

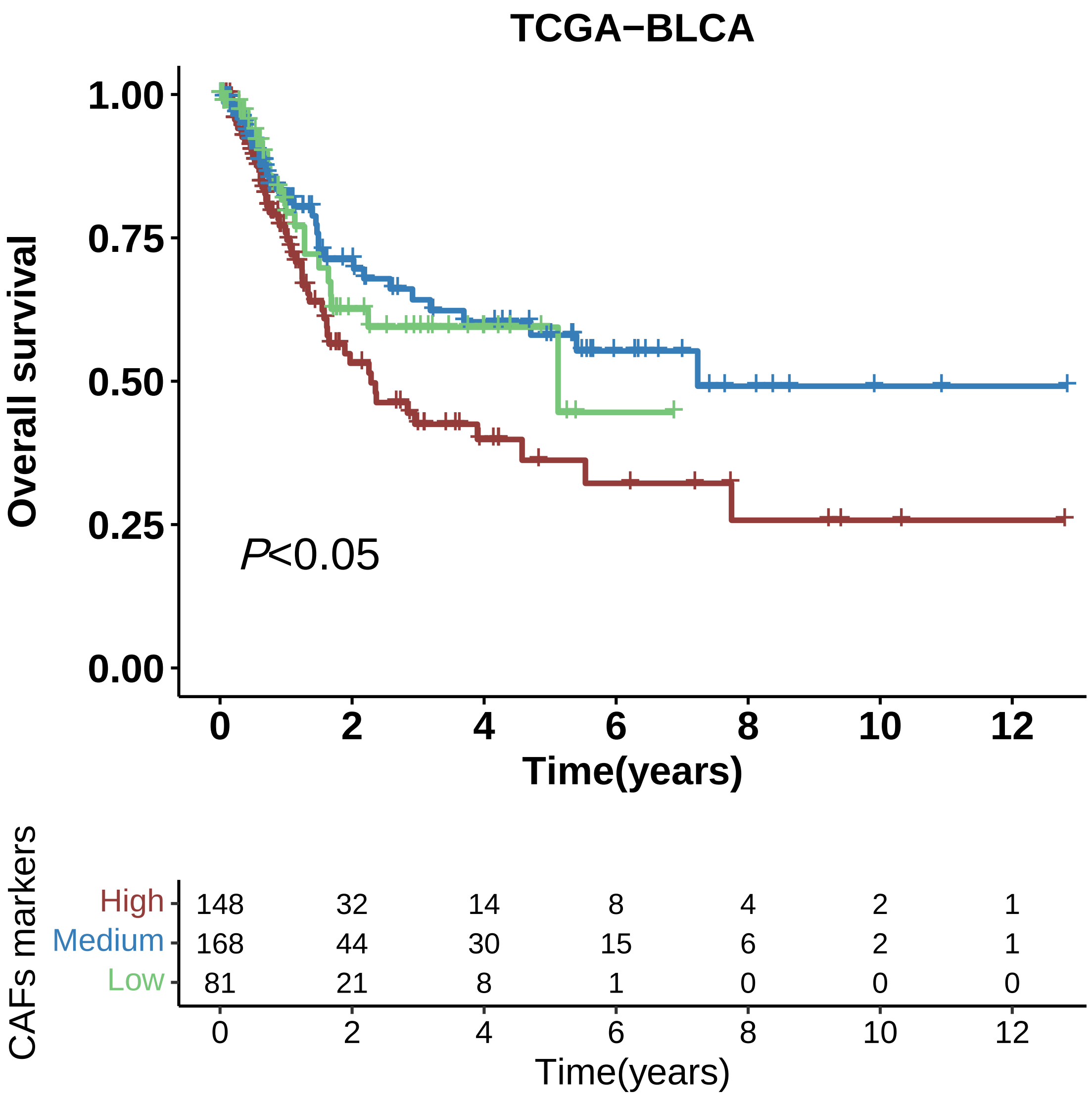

TCGA−BLCA
Overall survival
1.00
0.75
0.50
0.25
0.00
P<0.05
0
2
4
6
8
10
12
Time(years)
CAFs markers
High
Medium
Low
148 32 14 8 4 2 1
168 44 30 15 6 2 1
81 21 8 1 0 0 0
Time(years)

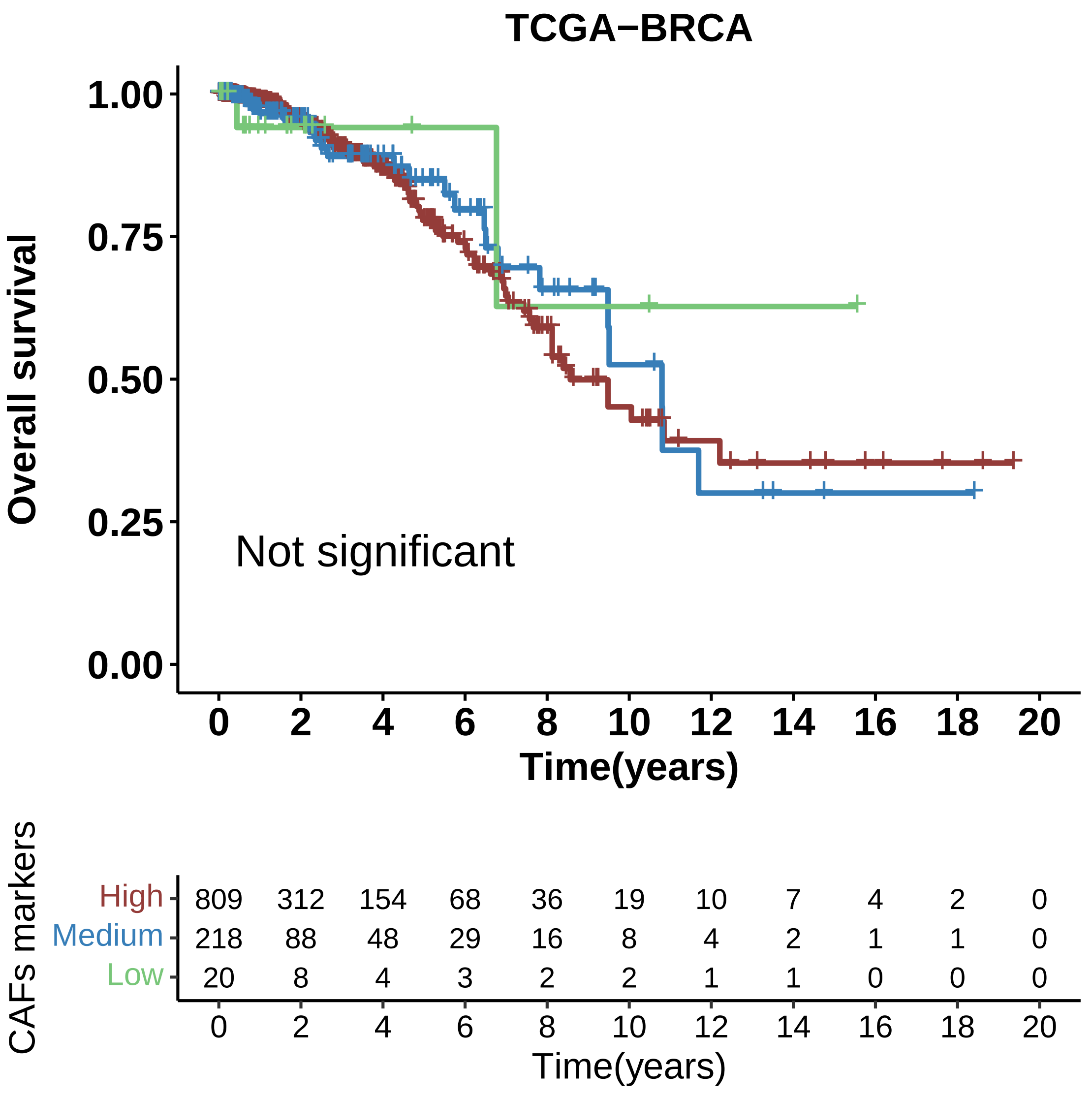
TCGA−BRCA
Overall survival
1.00
0.75
0.50
0.25
0.00
Not significant
0 2 4 6 8 10 12 14 16 18 20
Time(years)
CAFs markers
High 809 312 154 68 36 19 10 7 4 2 0
Medium 218 88 48 29 16 8 4 2 1 1 0
Low 20 8 4 3 2 2 1 1 0 0 0
0 2 4 6 8 10 12 14 16 18 20
Time(years)

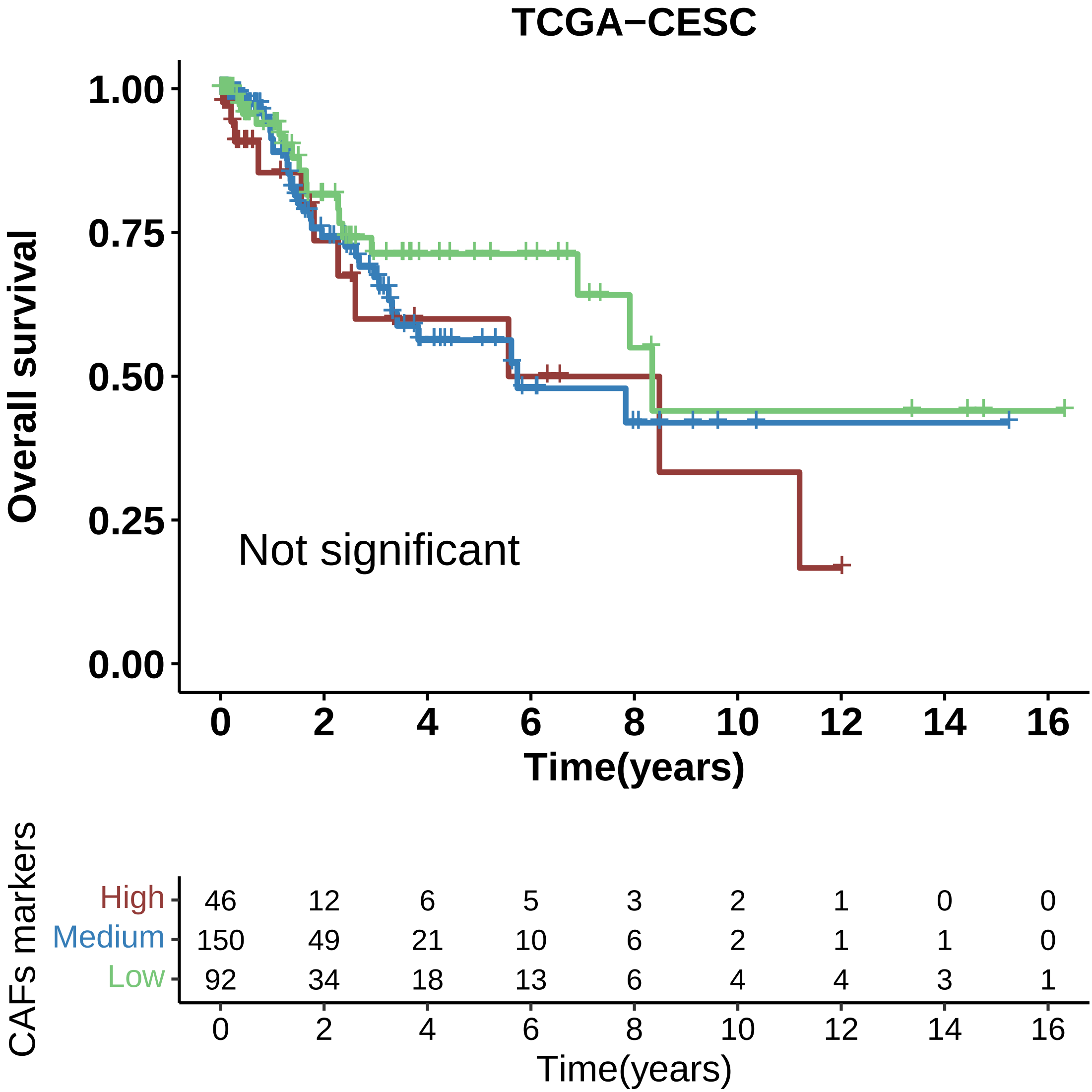
TCGA−CESC
Overall survival
1.00
0.75
0.50
0.25
0.00
Not significant
0 2 4 6 8 10 12 14 16
Time(years)
CAFs markers
High 46 12 6 5 3 2 1 0 0
Medium 150 49 21 10 6 2 1 1 0
Low 92 34 18 13 6 4 4 3 1
0 2 4 6 8 10 12 14 16
Time(years)

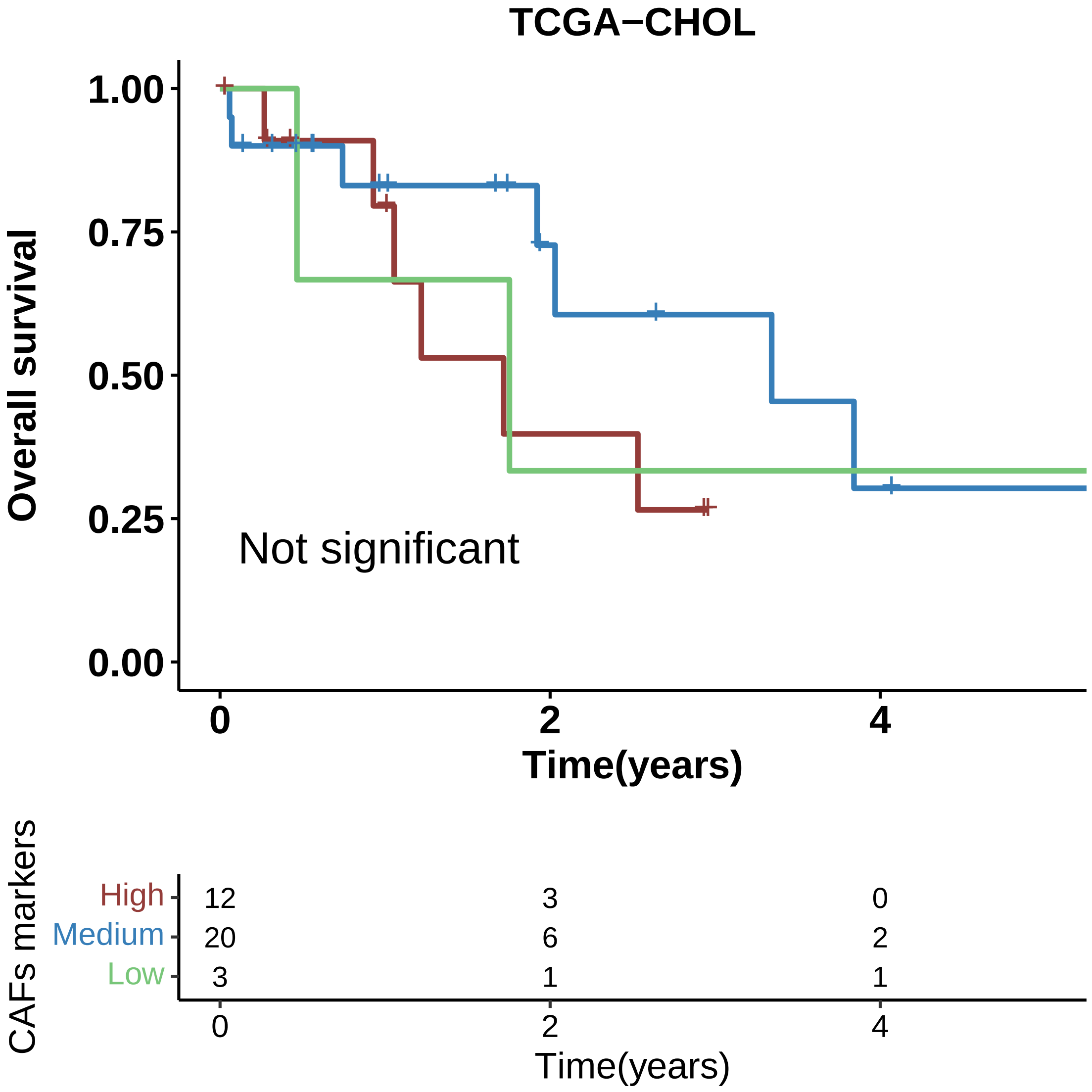
TCGA−CHOL
Overall survival
1.00
0.75
0.50
0.25
0.00
Not significant
0
2
4
Time(years)
CAFs markers
High
Medium
Low
12
3
0
20
6
2
3
1
1
0
2
4
Time(years)

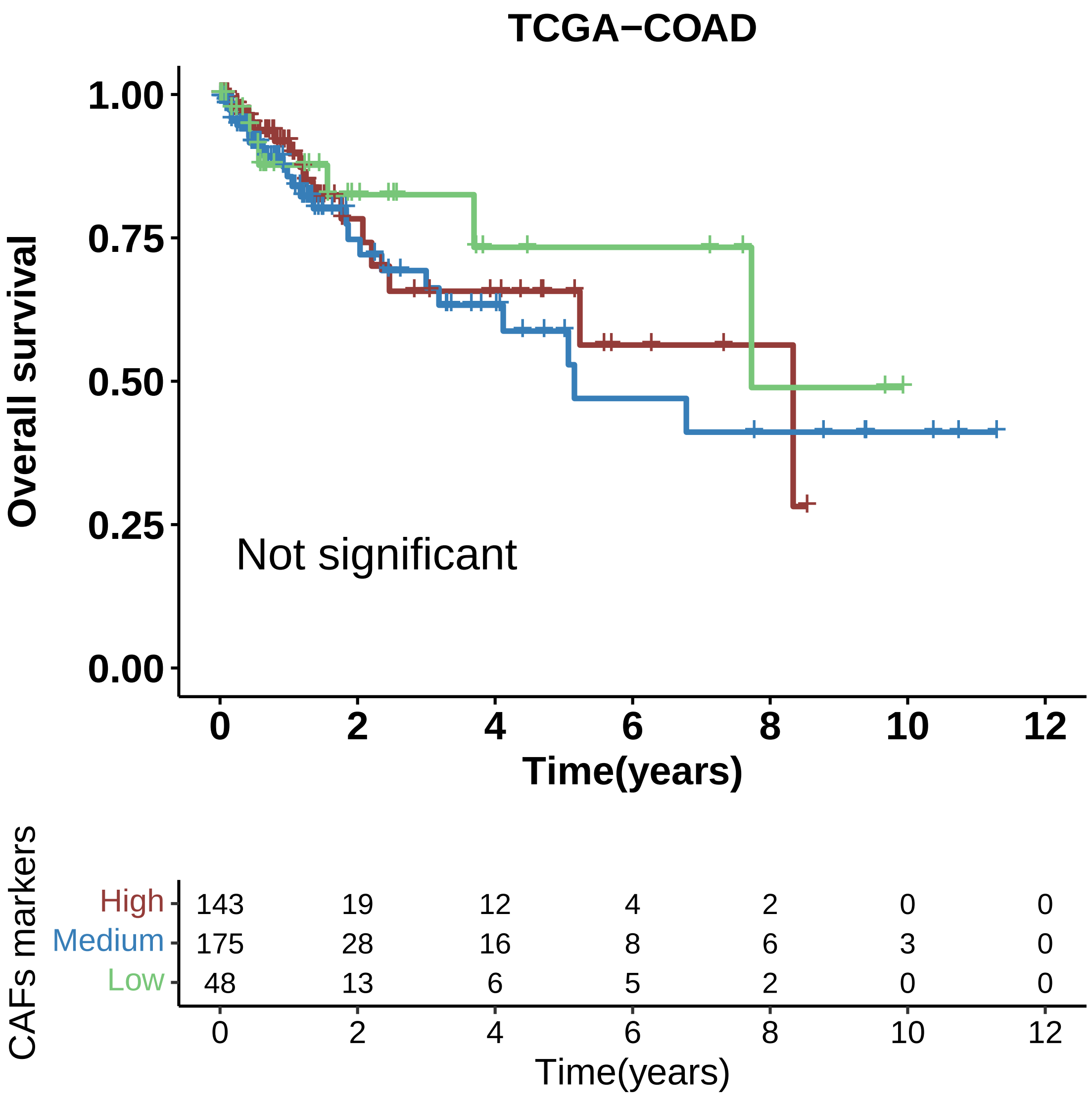

TCGA−COAD
Overall survival
1.00
0.75
0.50
0.25
0.00
Not significant
0
2
4
6
8
10
12
Time(years)
CAFs markers
High
Medium
Low
143 19 12 4 2 0 0
175 28 16 8 6 3 0
48 13 6 5 2 0 0
0
2
4
6
8
10
12
Time(years)

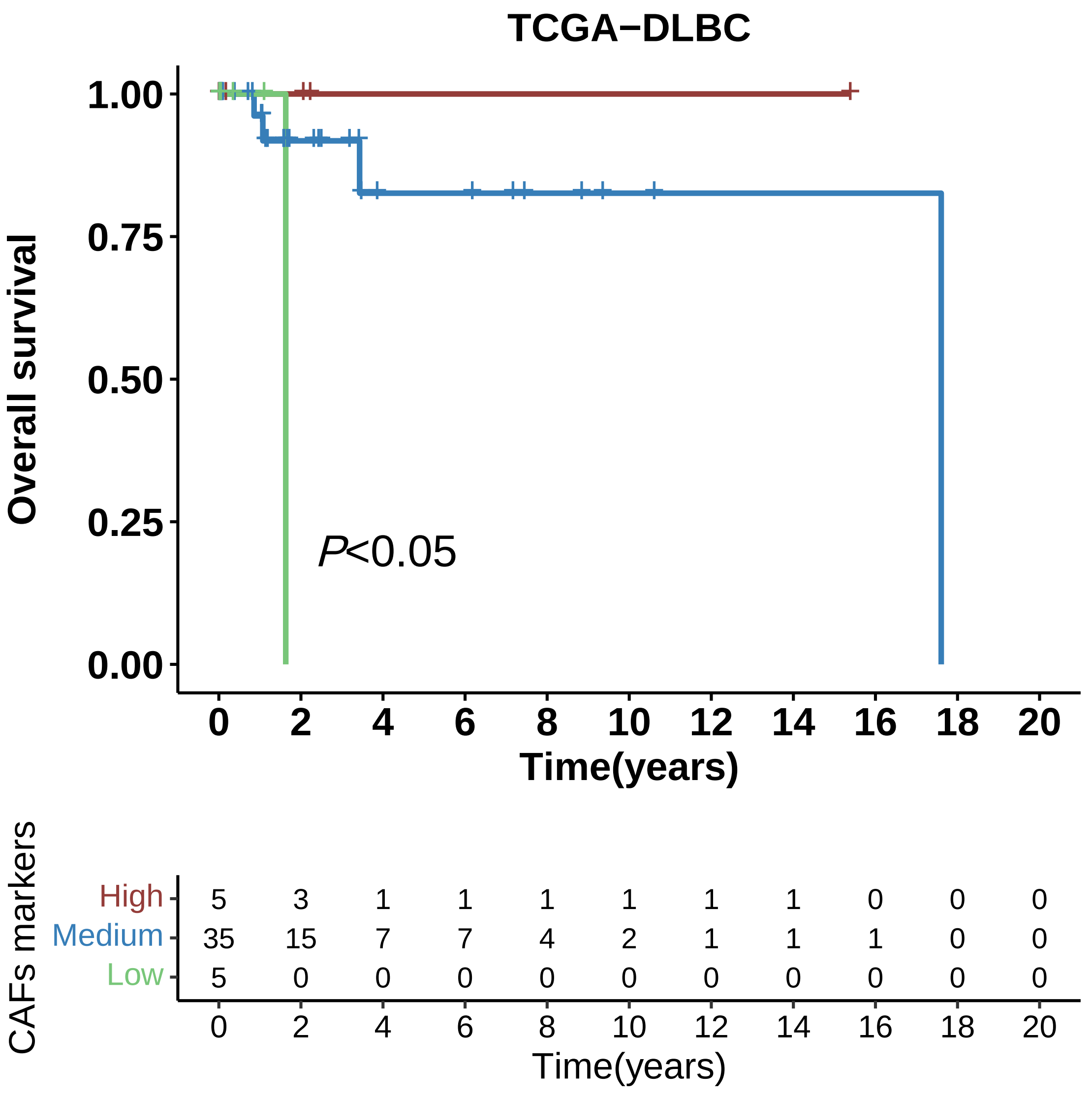
TCGA−DLBC
Overall survival
1.00
0.75
0.50
0.25
0.00
P<0.05
0 2 4 6 8 10 12 14 16 18 20
Time(years)
CAFs markers
High 5 3 1 1 1 1 1 1 0 0 0
Medium 35 15 7 7 4 2 1 1 1 0 0
Low 5 0 0 0 0 0 0 0 0 0 0
0 2 4 6 8 10 12 14 16 18 20
Time(years)

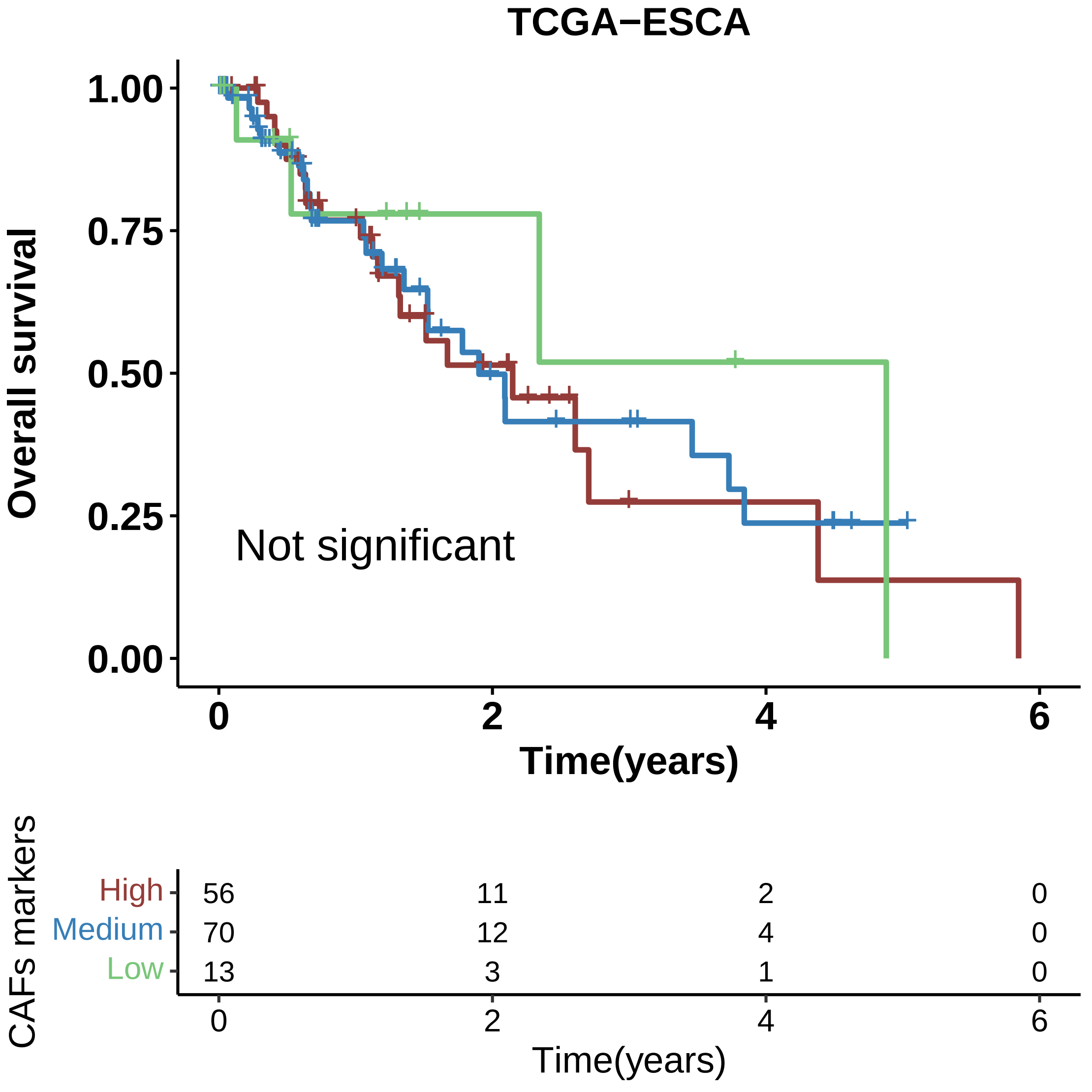
TCGA−ESCA
Overall survival
1.00
0.75
0.50
0.25
0.00
Not significant
0
2
4
6
Time(years)
CAFs markers
High
Medium
Low
56
11
2
0
70
12
4
0
13
3
1
0
0
2
4
6
Time(years)

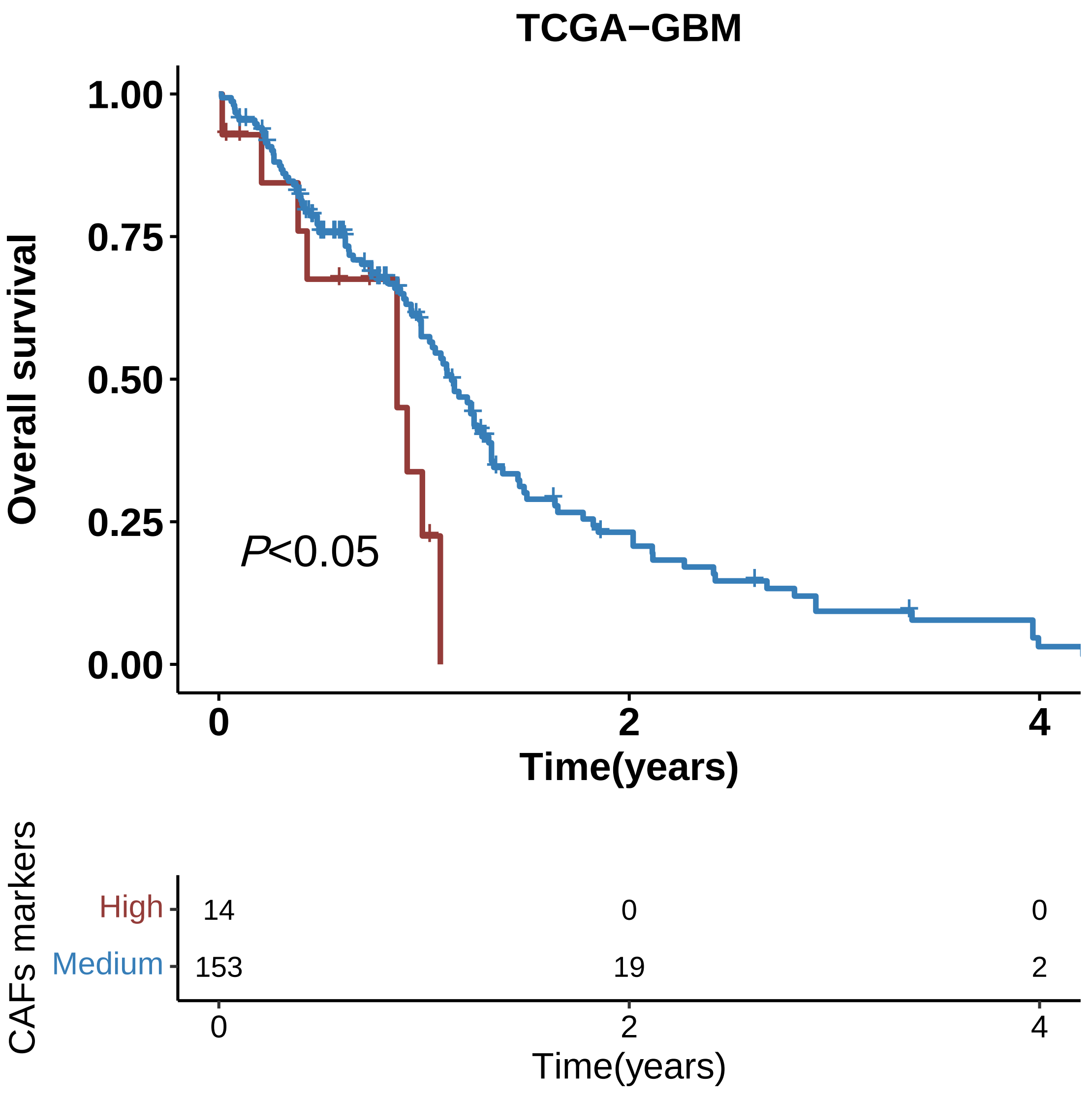
TCGA−GBM
Overall survival
1.00
0.75
0.50
0.25
0.00
P<0.05
0
2
4
Time(years)
CAFs markers
High
Medium
14
0
0
153
19
2
0
2
4
Time(years)

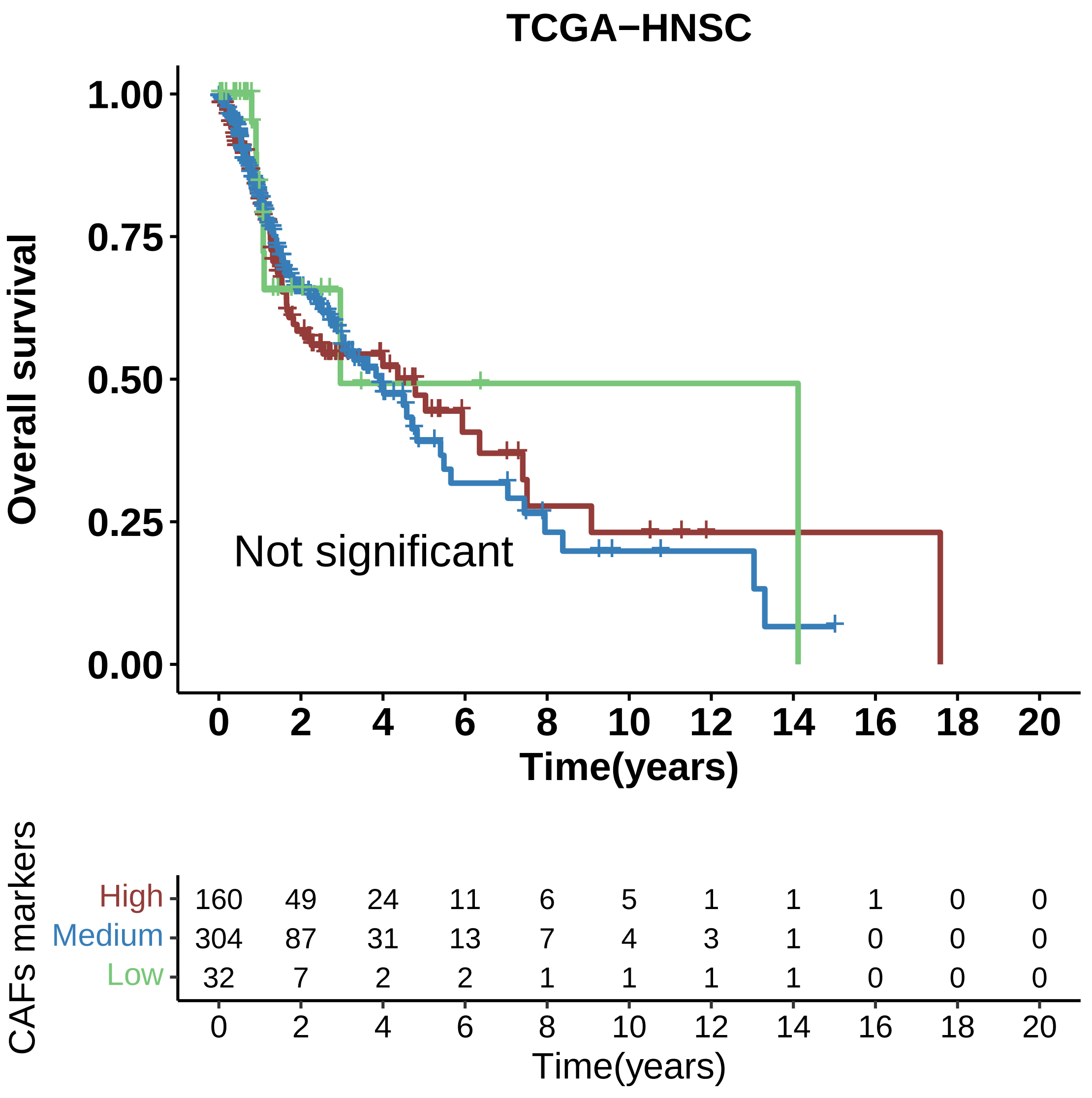
TCGA−HNSC
Overall survival
1.00
0.75
0.50
0.25
0.00
Not significant
0
2
4
6
8
10
12
14
16
18
20
Time(years)
CAFs markers
High
Medium
Low
160 49 24 11 6 5 1 1 1 0 0
304 87 31 13 7 4 3 1 0 0 0
32 7 2 2 1 1 1 1 0 0 0
Time(years)

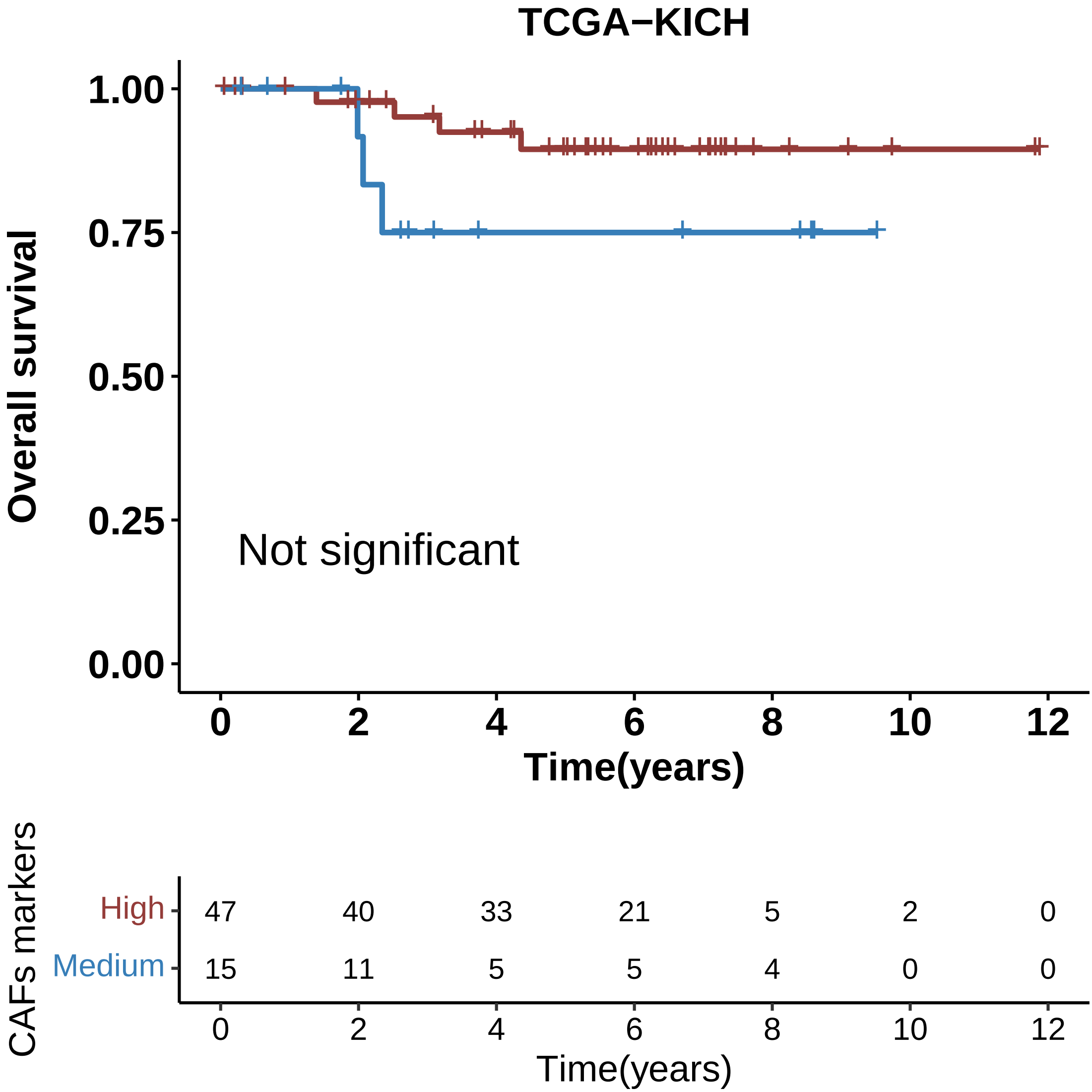
TCGA−KICH
Overall survival
1.00
0.75
0.50
0.25
0.00
Not significant
0
2
4
6
8
10
12
Time(years)
CAFs markers
High
Medium
47 40 33 21 5 2 0
15 11 5 5 4 0 0
0
2
4
6
8
10
12
Time(years)

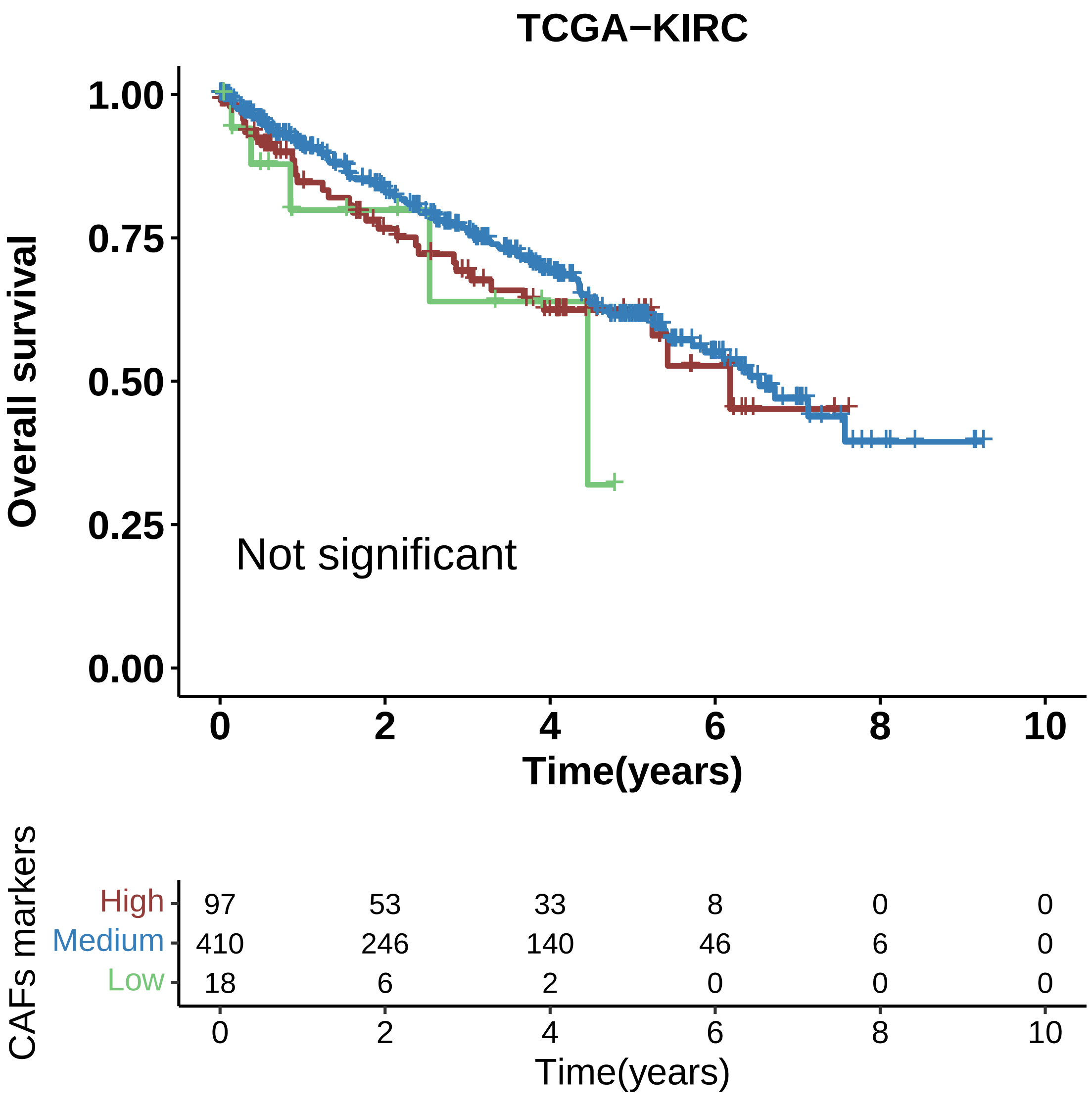

TCGA−KIRC
Overall survival
1.00
0.75
0.50
0.25
0.00
Not significant
0
2
4
6
8
10
Time(years)
CAFs markers
High
Medium
Low
97 53 33 8 0 0
410 246 140 46 6 0
18 6 2 0 0 0
Time(years)

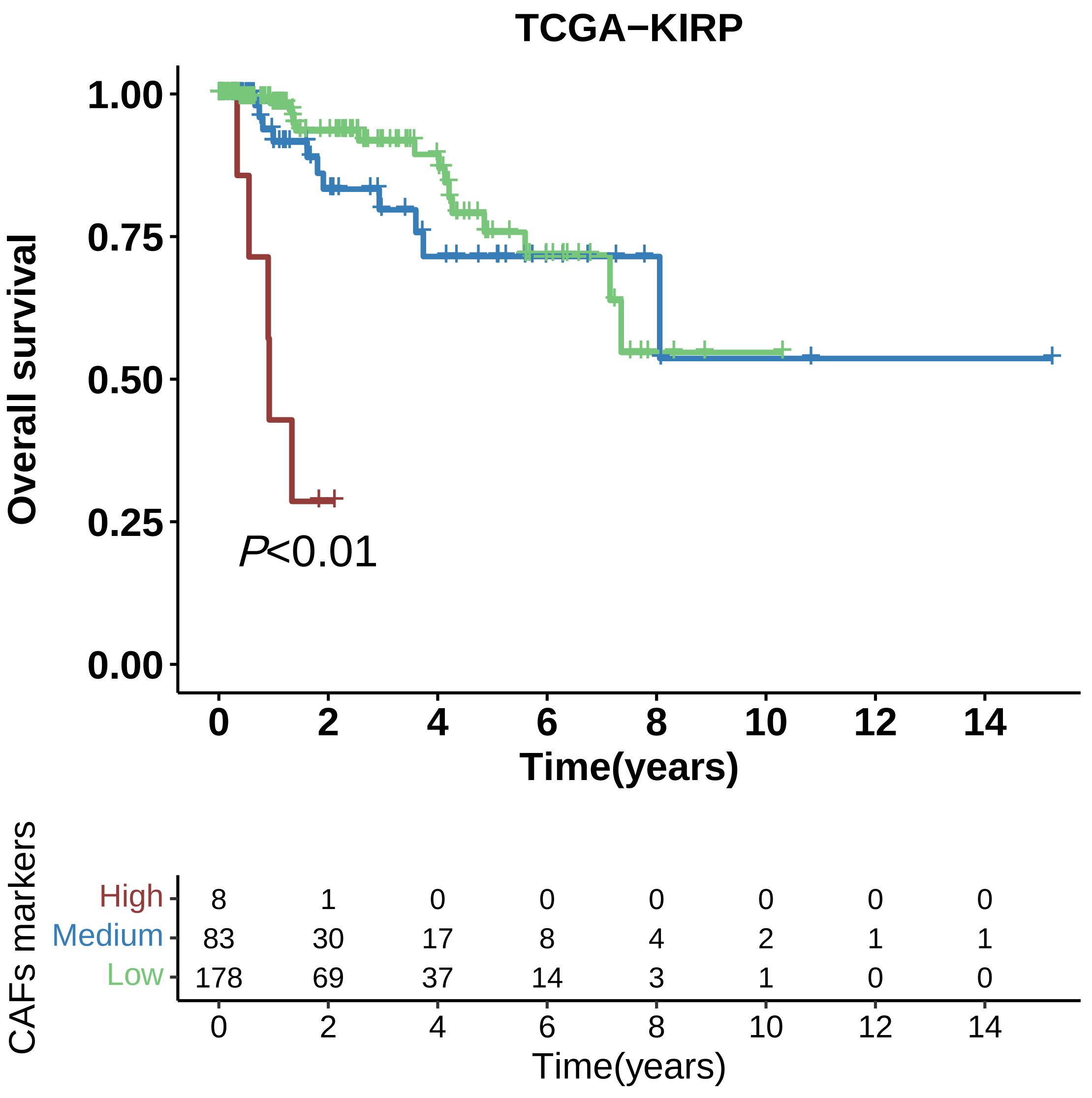
TCGA−KIRP
Overall survival
1.00
0.75
0.50
0.25
0.00
P<0.01
0
2
4
6
8
10
12
14
Time(years)
CAFs markers
High
Medium
Low
8 1 0 0 0 0 0 0
83 30 17 8 4 2 1 1
178 69 37 14 3 1 0 0
Time(years)

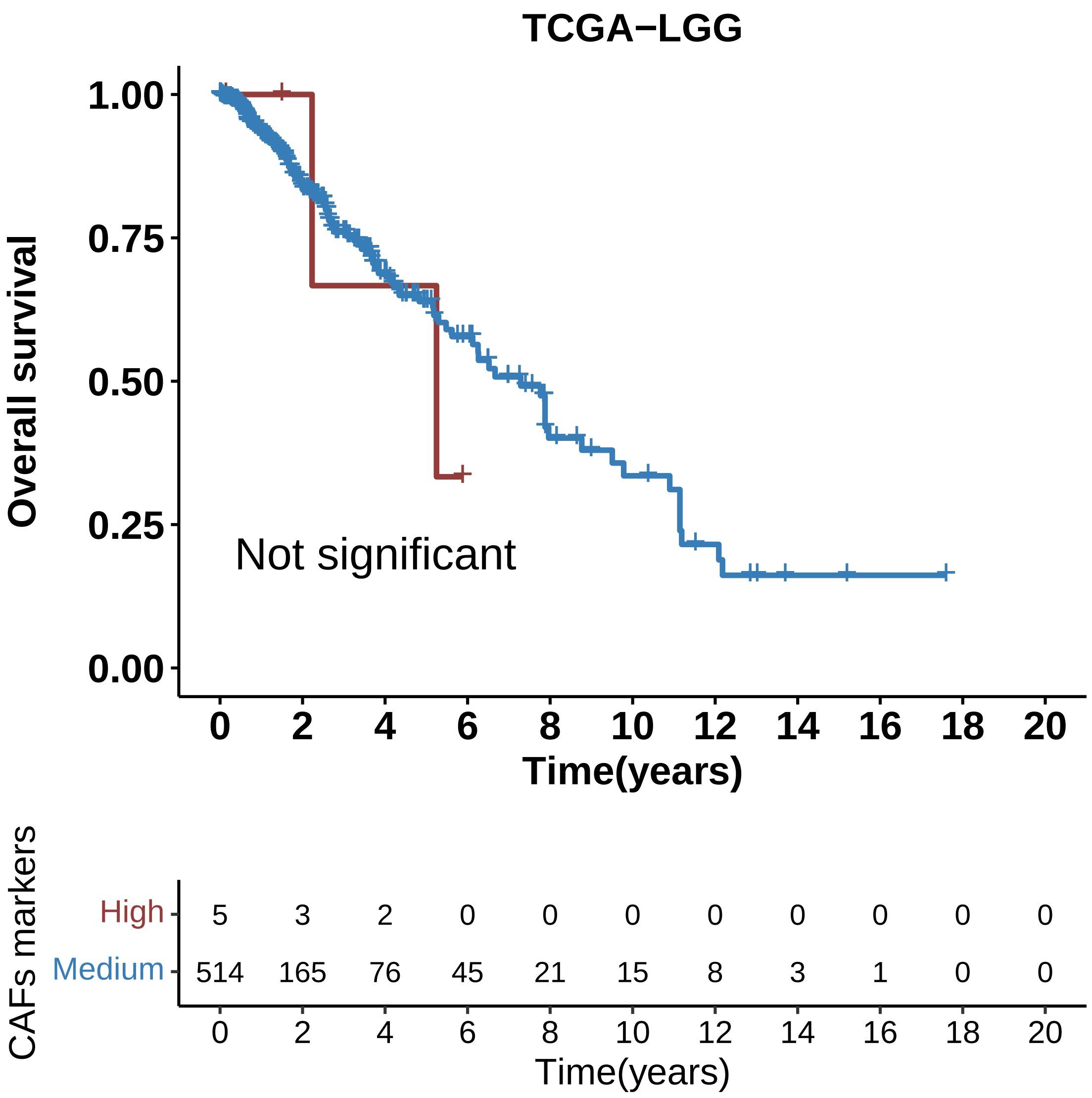

TCGA−LGG
Overall survival
1.00
0.75
0.50
0.25
0.00
Not significant
0 2 4 6 8 10 12 14 16 18 20
Time(years)
CAFs markers
High
Medium
5 3 2 0 0 0 0 0 0 0 0
514 165 76 45 21 15 8 3 1 0 0
0 2 4 6 8 10 12 14 16 18 20
Time(years)

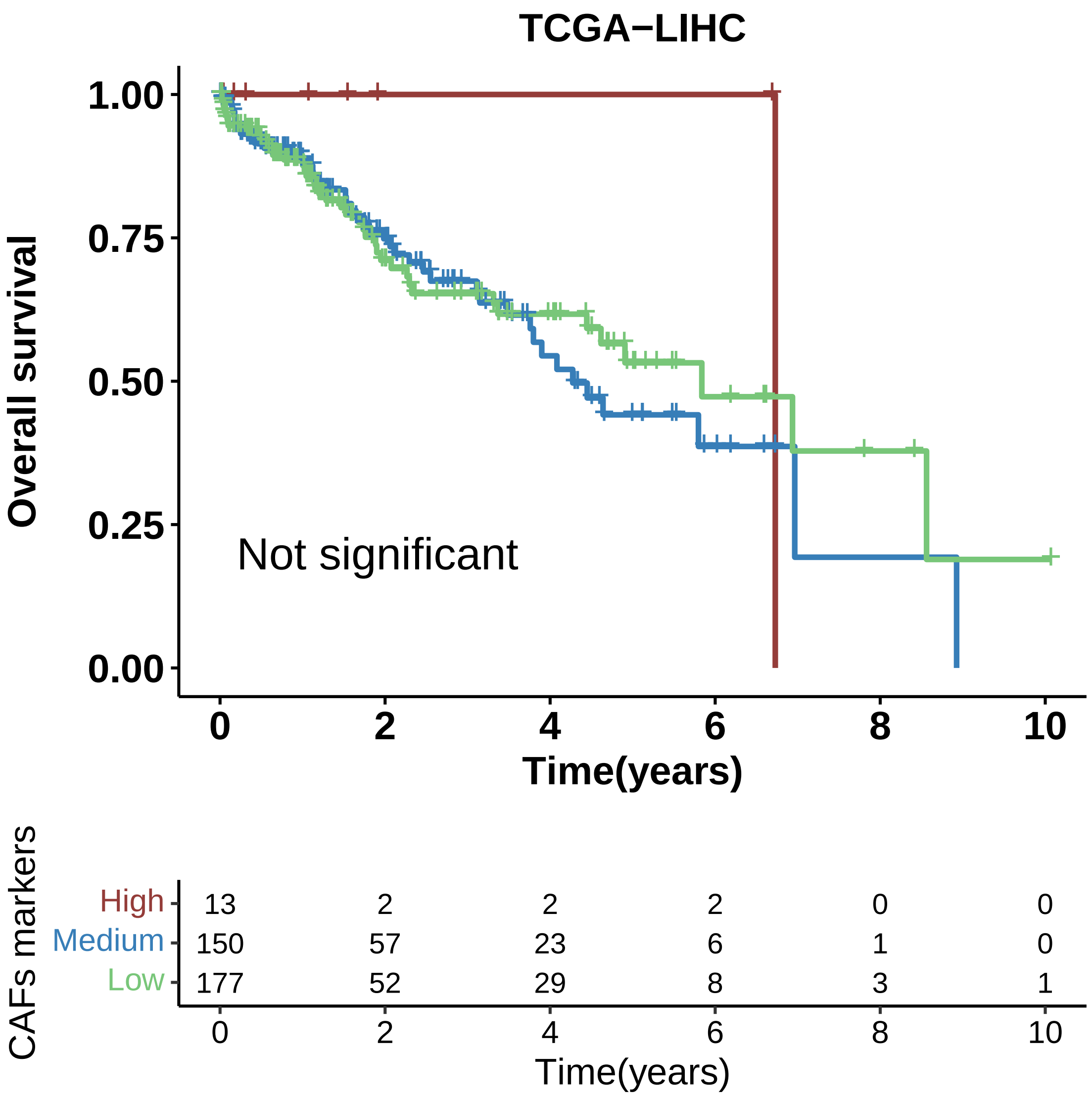

TCGA−LIHC
Overall survival
1.00
0.75
0.50
0.25
0.00
Not significant
0
2
4
6
8
10
Time(years)
CAFs markers
High
Medium
Low
13 2 2 2 0 0
150 57 23 6 1 0
177 52 29 8 3 1
Time(years)

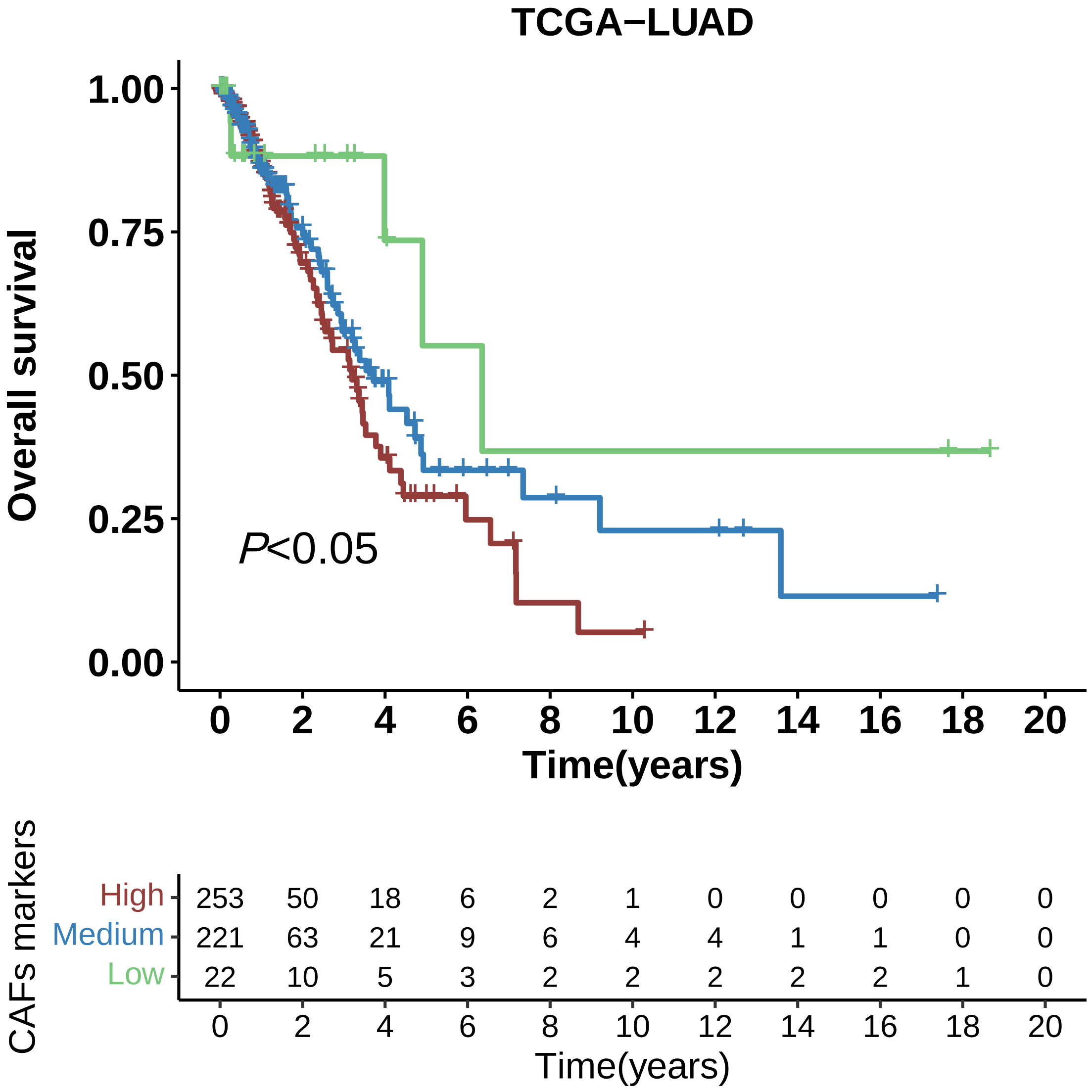
TCGA−LUAD
Overall survival
1.00
0.75
0.50
0.25
0.00
P<0.05
0 2 4 6 8 10 12 14 16 18 20
Time(years)
CAFs markers
High 253 50 18 6 2 1 0 0 0 0 0
Medium 221 63 21 9 6 4 4 1 1 0 0
Low 22 10 5 3 2 2 2 2 2 1 0
0 2 4 6 8 10 12 14 16 18 20
Time(years)

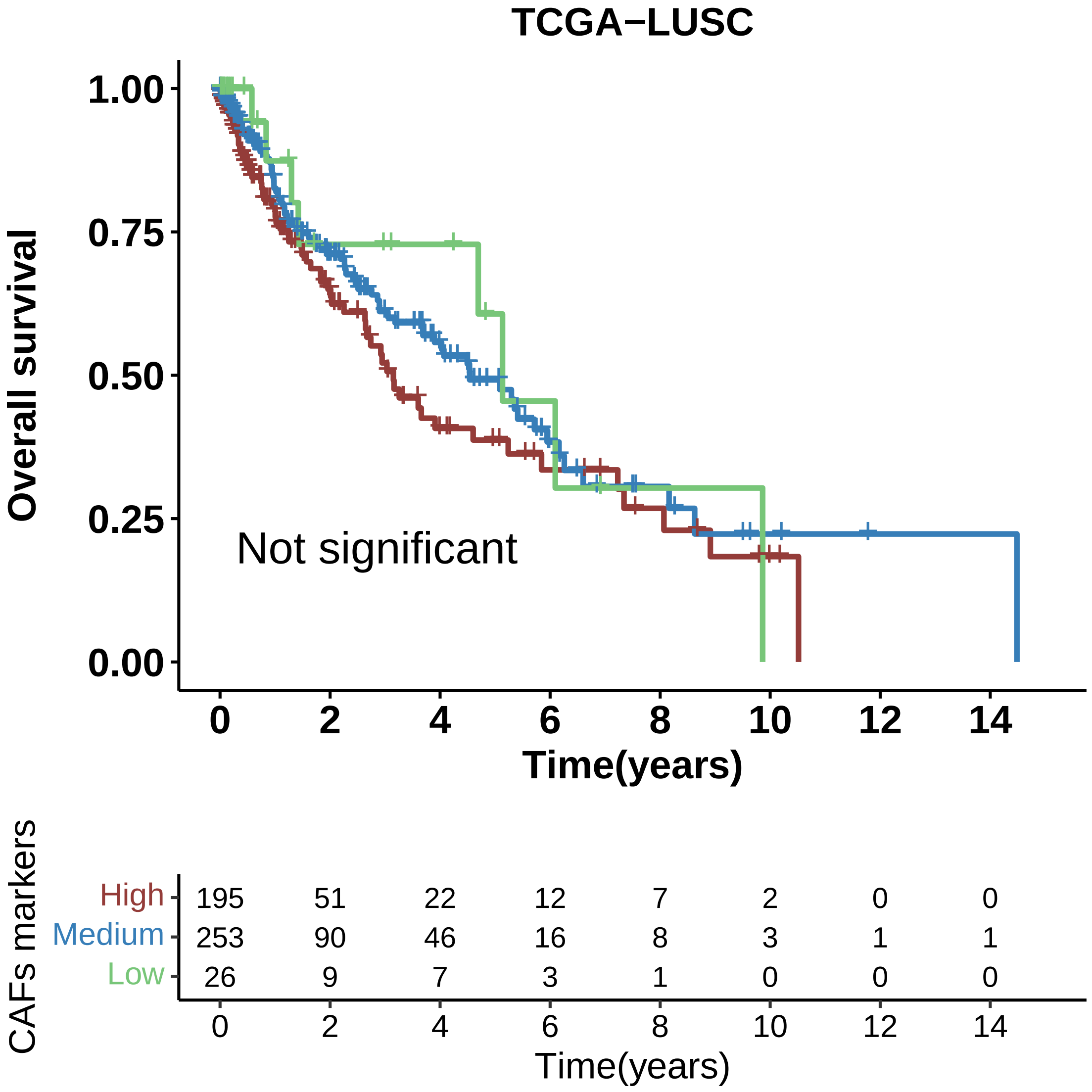
TCGA−LUSC
Overall survival
1.00
0.75
0.50
0.25
0.00
Not significant
0
2
4
6
8
10
12
14
Time(years)
CAFs markers
High 195 51 22 12 7 2 0 0
Medium 253 90 46 16 8 3 1 1
Low 26 9 7 3 1 0 0 0
Time(years)

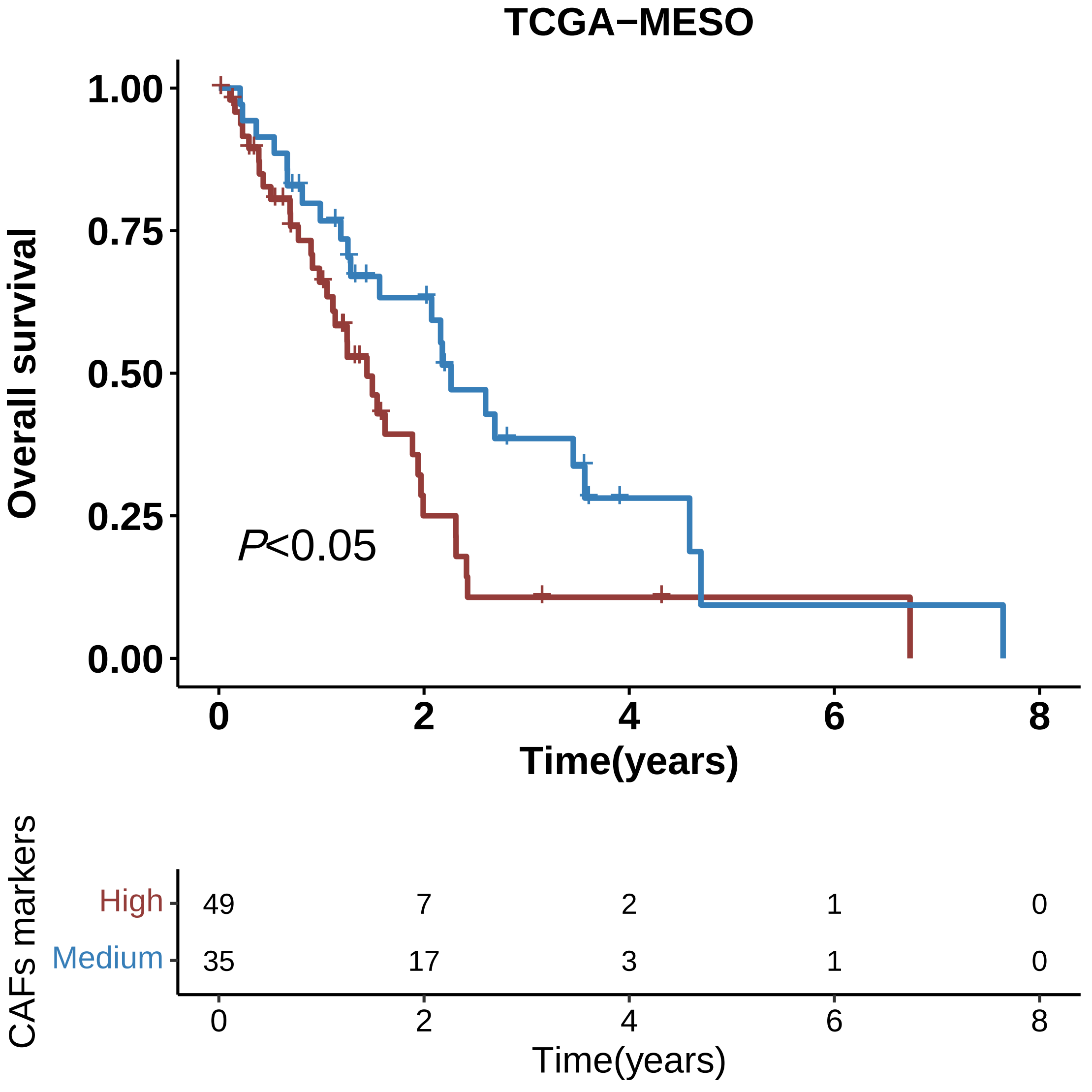

TCGA−MESO
Overall survival
1.00
0.75
0.50
0.25
0.00
P<0.05
0
2
4
6
8
Time(years)
CAFs markers
High
Medium
49
7
2
1
0
35
17
3
1
0
0
2
4
6
8
Time(years)

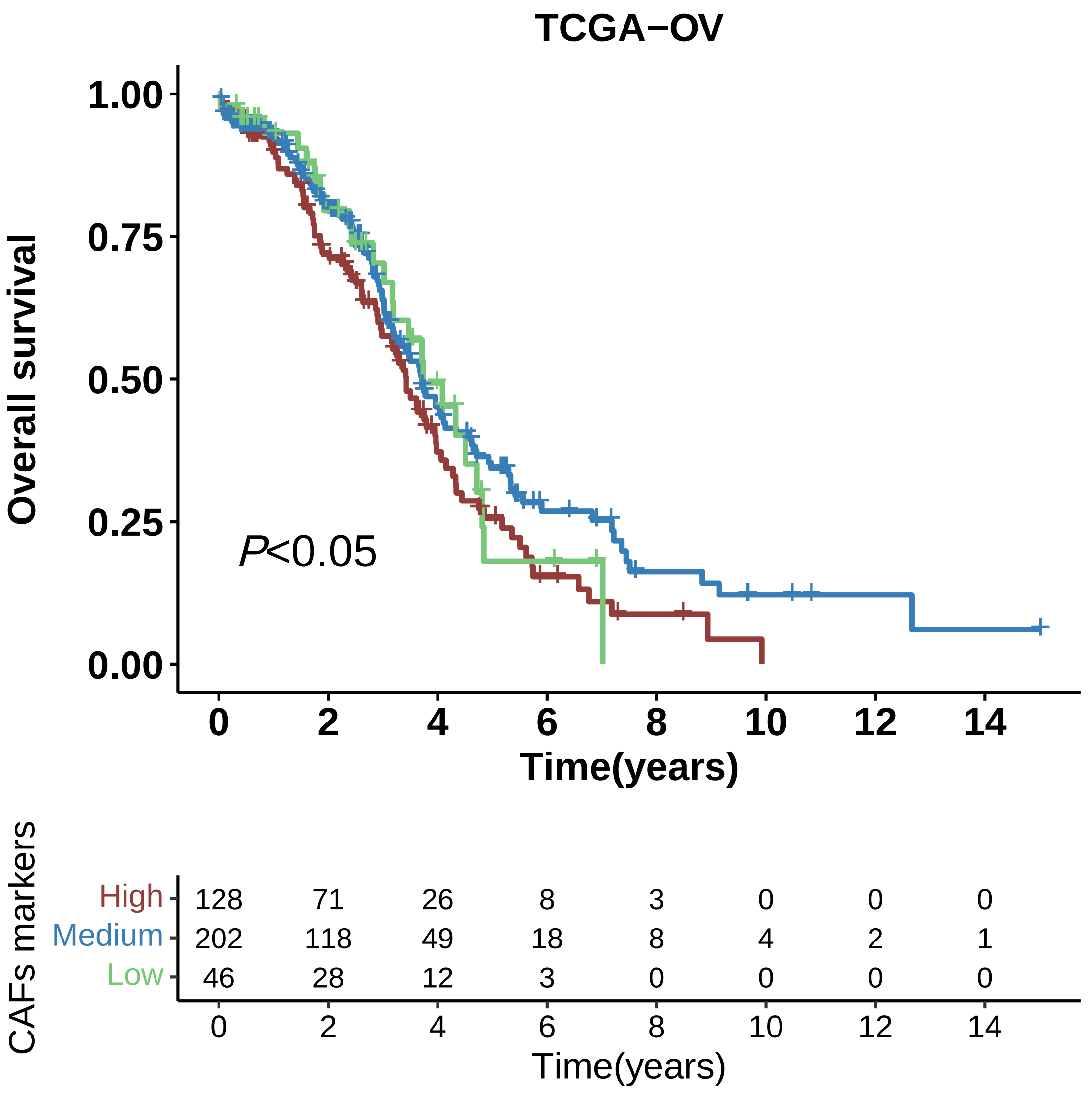
TCGA−OV
Overall survival
1.00
0.75
0.50
0.25
0.00
P<0.05
0 2 4 6 8 10 12 14
Time(years)
CAFs markers
High 128 71 26 8 3 0 0 0
Medium 202 118 49 18 8 4 2 1
Low 46 28 12 3 0 0 0 0
0 2 4 6 8 10 12 14
Time(years)

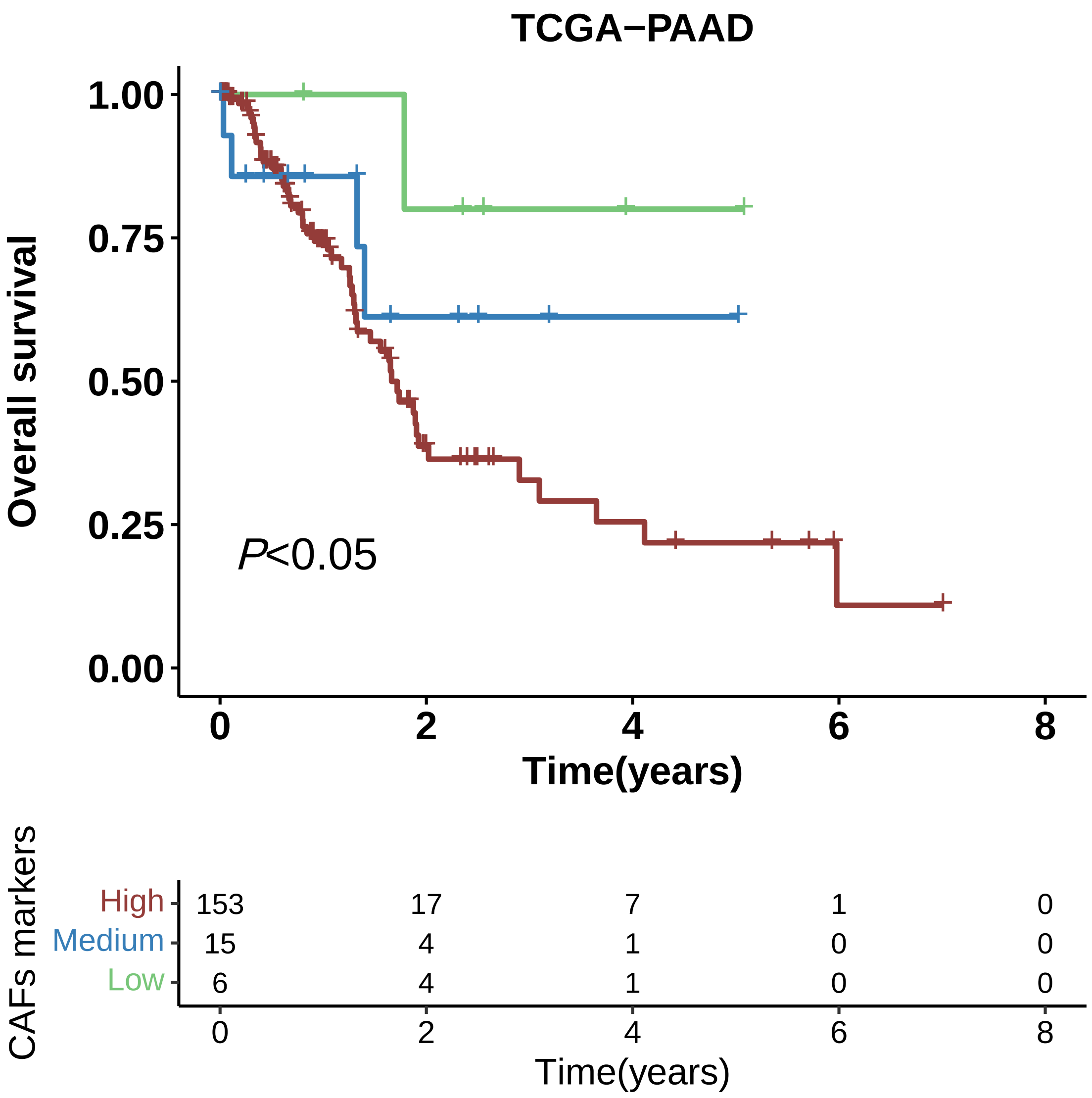
TCGA−PAAD
Overall survival
1.00
0.75
0.50
0.25
0.00
P<0.05
0
2
4
6
8
Time(years)
CAFs markers
High
Medium
Low
153
17
7
1
0
15
4
1
0
0
6
4
1
0
0
0
2
4
6
8
Time(years)

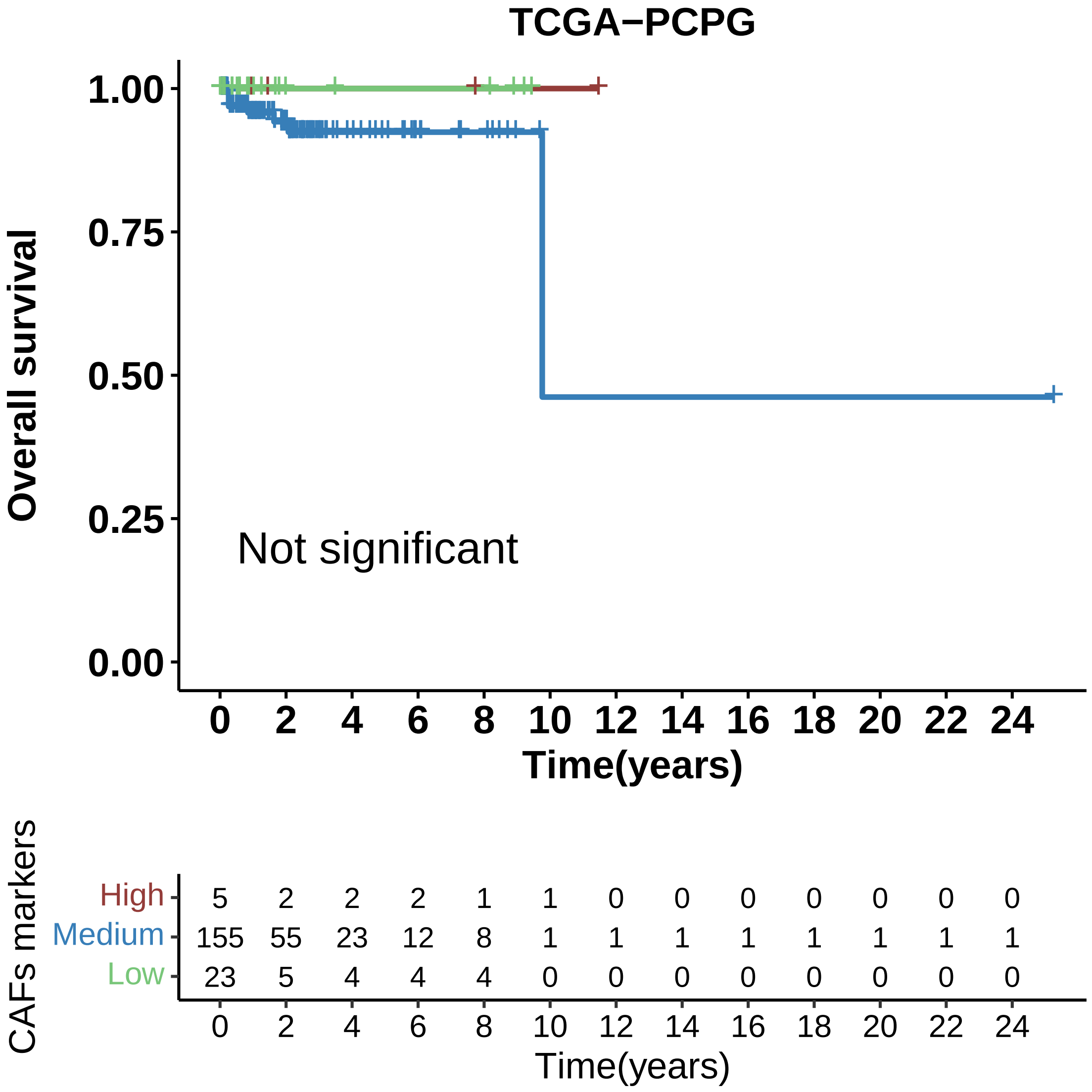

TCGA−PCPG
Overall survival
1.00
0.75
0.50
0.25
0.00
Not significant
0 2 4 6 8 10 12 14 16 18 20 22 24
Time(years)
CAFs markers
High 5 2 2 2 1 1 0 0 0 0 0 0 0
Medium 155 55 23 12 8 1 1 1 1 1 1 1 1
Low 23 5 4 4 4 0 0 0 0 0 0 0 0
0 2 4 6 8 10 12 14 16 18 20 22 24
Time(years)

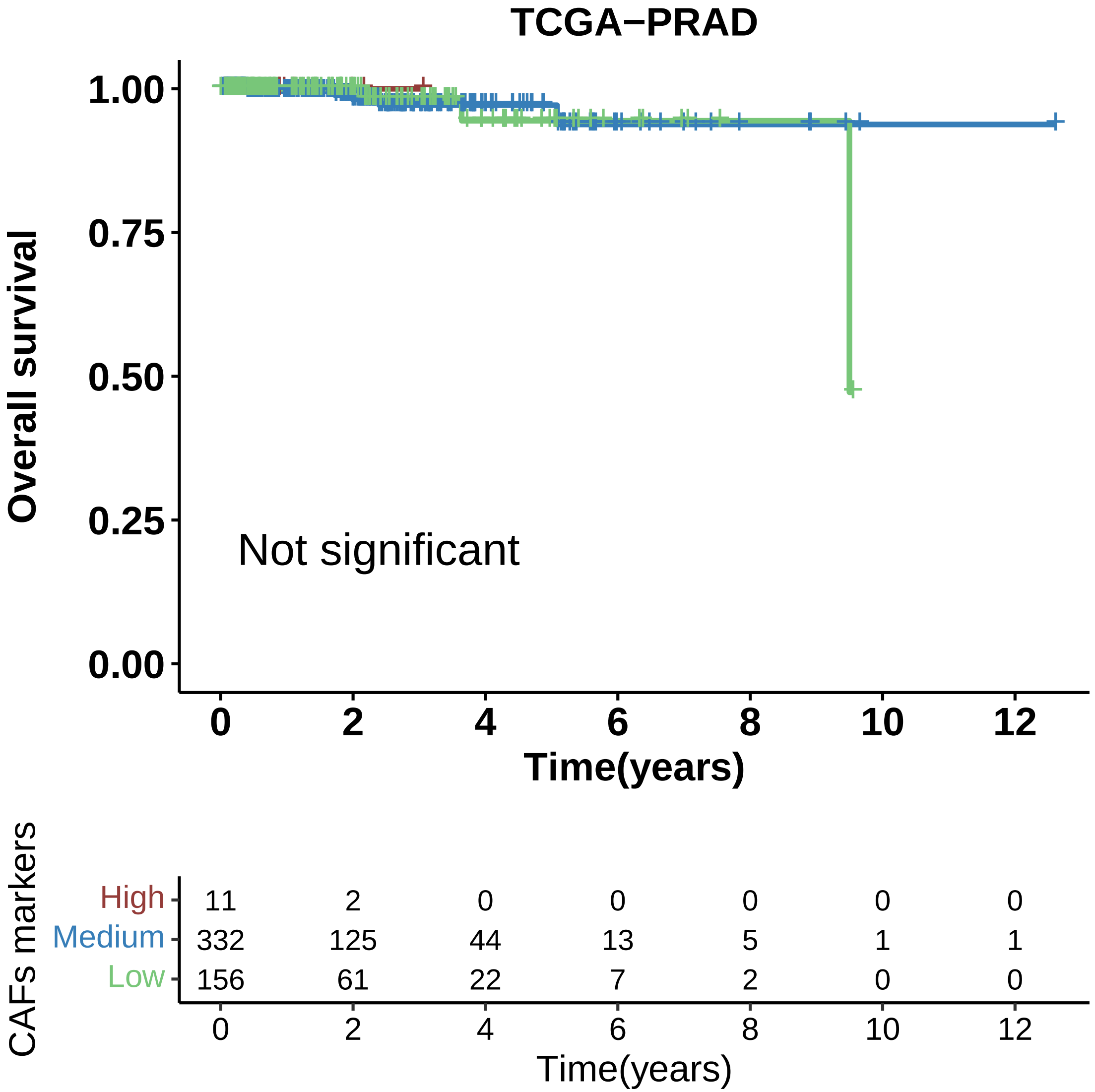

TCGA−PRAD
Overall survival
1.00
0.75
0.50
0.25
0.00
Not significant
0
2
4
6
8
10
12
Time(years)
CAFs markers
High
Medium
Low
11 2 0 0 0 0 0
332 125 44 13 5 1 1
156 61 22 7 2 0 0
0
2
4
6
8
10
12
Time(years)

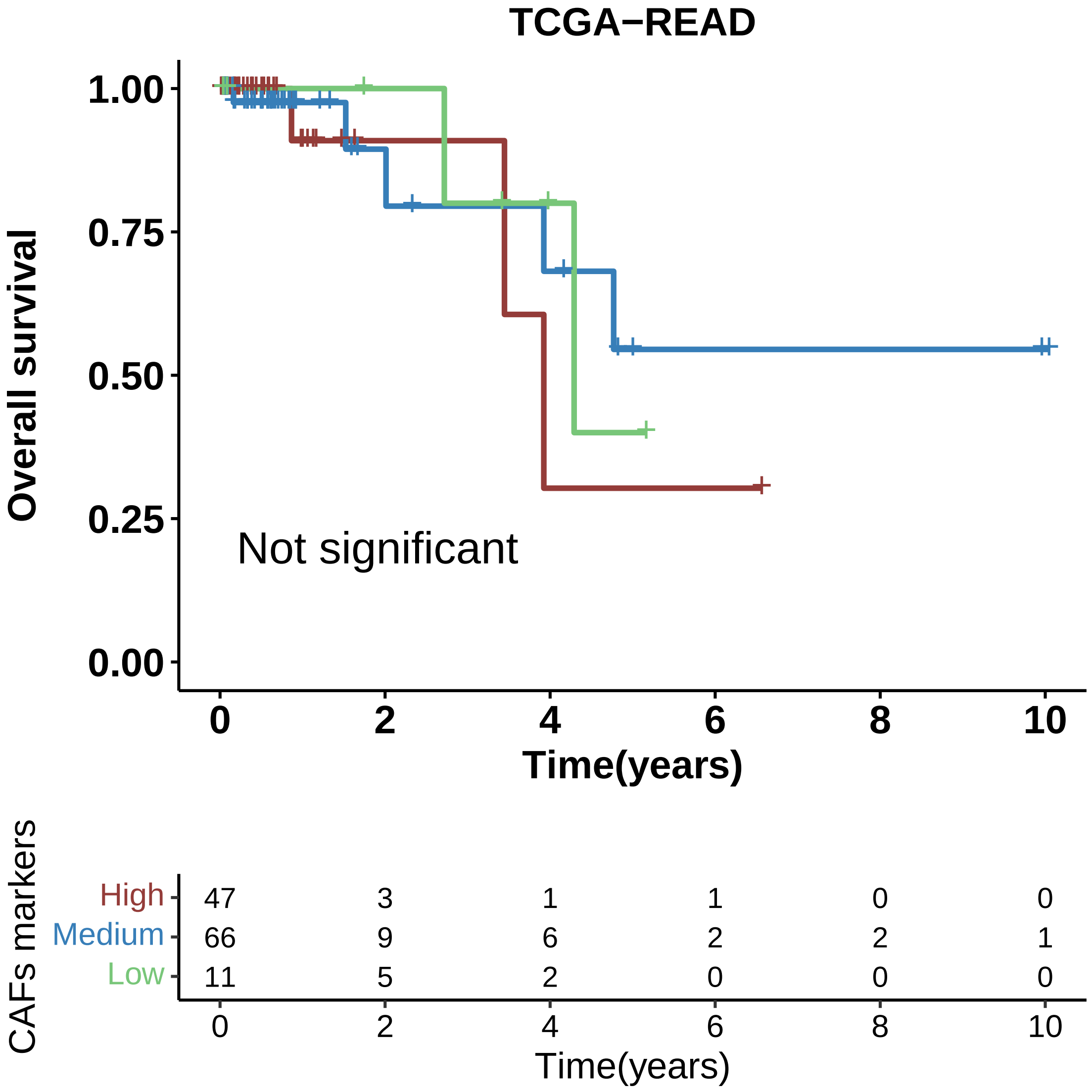

TCGA−READ
Overall survival
1.00
0.75
0.50
0.25
0.00
Not significant
0
2
4
6
8
10
Time(years)
CAFs markers
High 47 3 1 1 0 0
Medium 66 9 6 2 2 1
Low 11 5 2 0 0 0
Time(years)

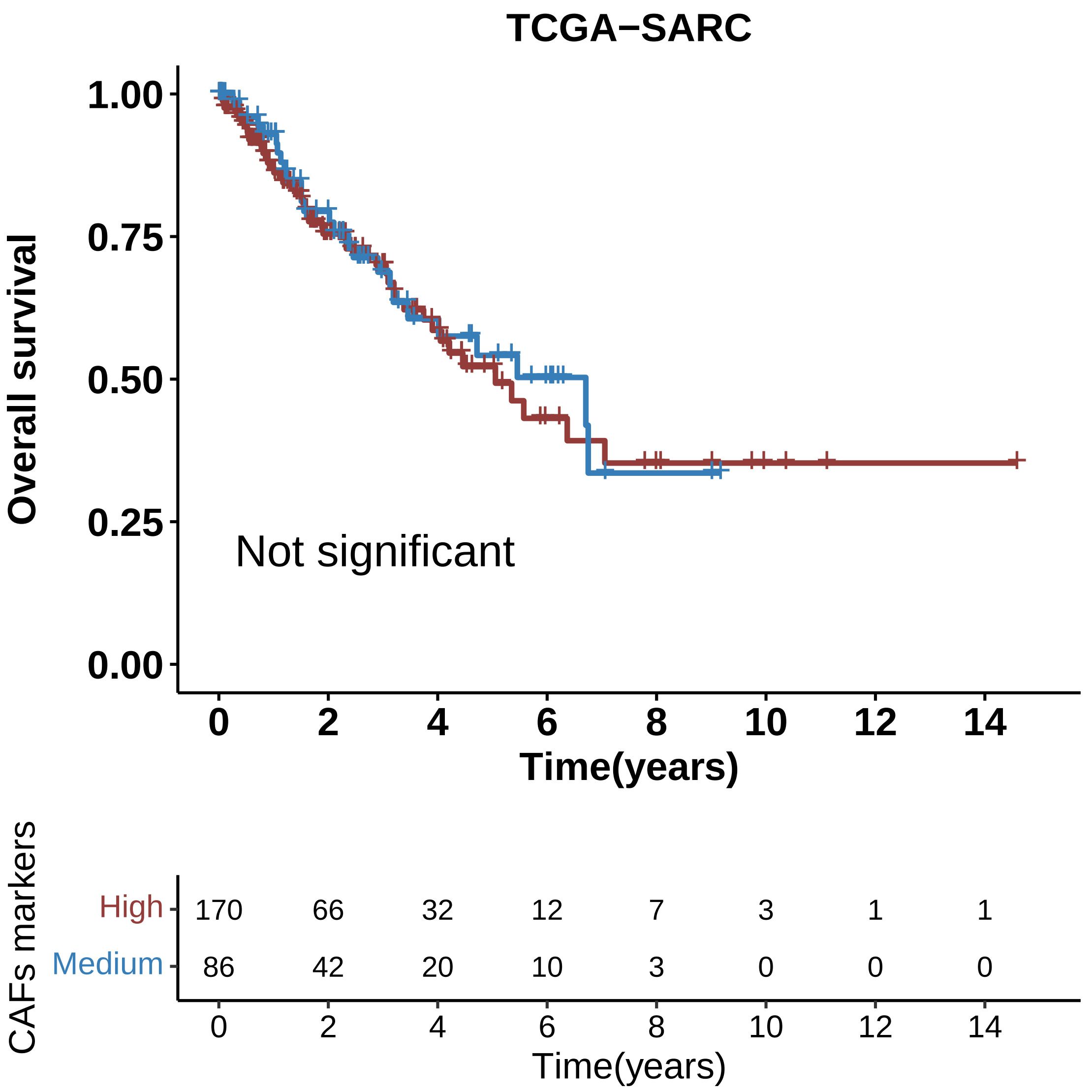

TCGA−SARC
Overall survival
1.00
0.75
0.50
0.25
0.00
Not significant
0
2
4
6
8
10
12
14
Time(years)
CAFs markers
High
Medium
170 66 32 12 7 3 1 1
86 42 20 10 3 0 0 0
Time(years)

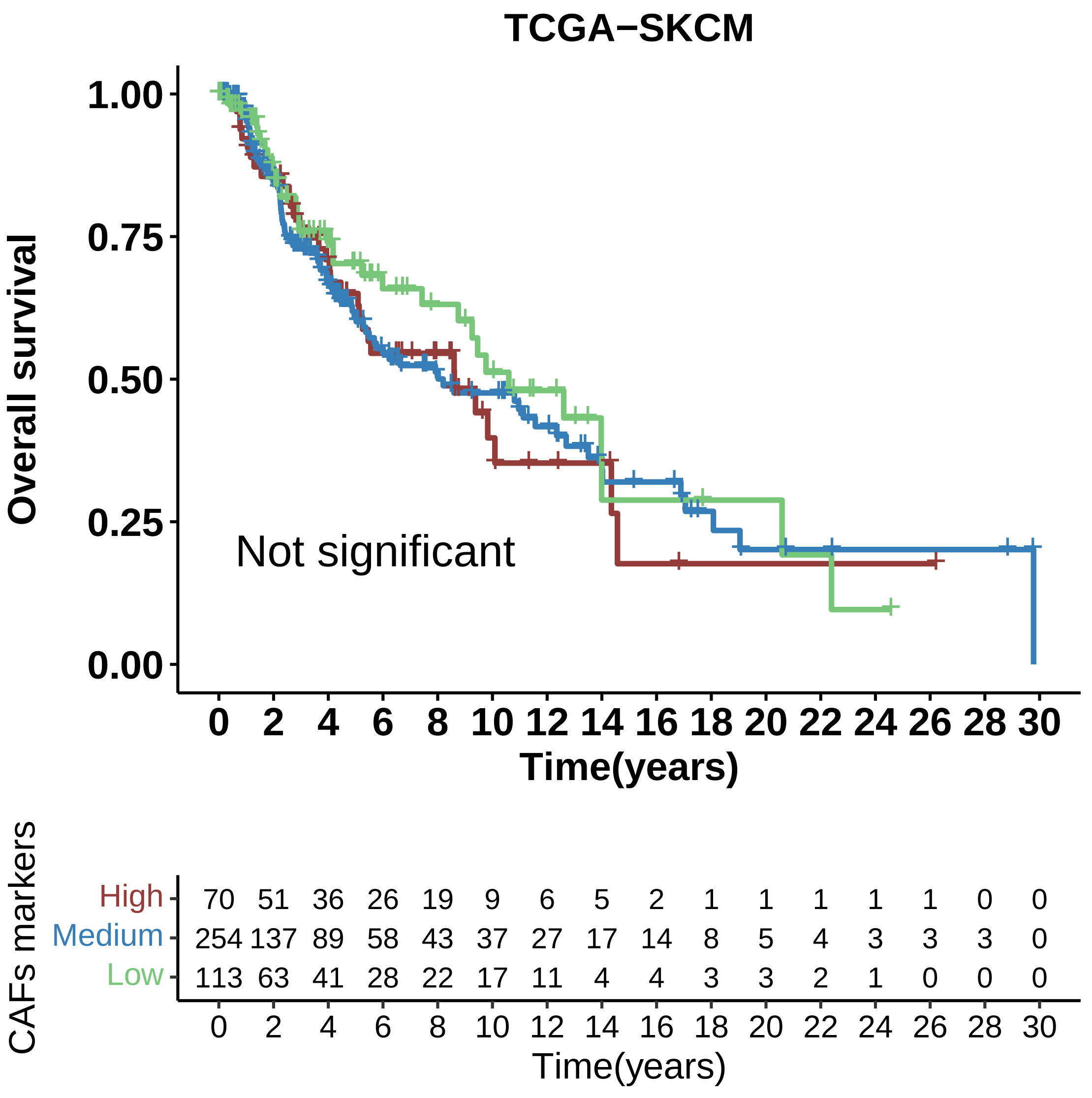

TCGA−SKCM
Overall survival
1.00
0.75
0.50
0.25
0.00
Not significant
0 2 4 6 8 10 12 14 16 18 20 22 24 26 28 30
Time(years)
CAFs markers
High 70 51 36 26 19 9 6 5 2 1 1 1 1 1 0 0
Medium 254 137 89 58 43 37 27 17 14 8 5 4 3 3 3 0
Low 113 63 41 28 22 17 11 4 4 3 3 2 1 0 0 0
0 2 4 6 8 10 12 14 16 18 20 22 24 26 28 30
Time(years)

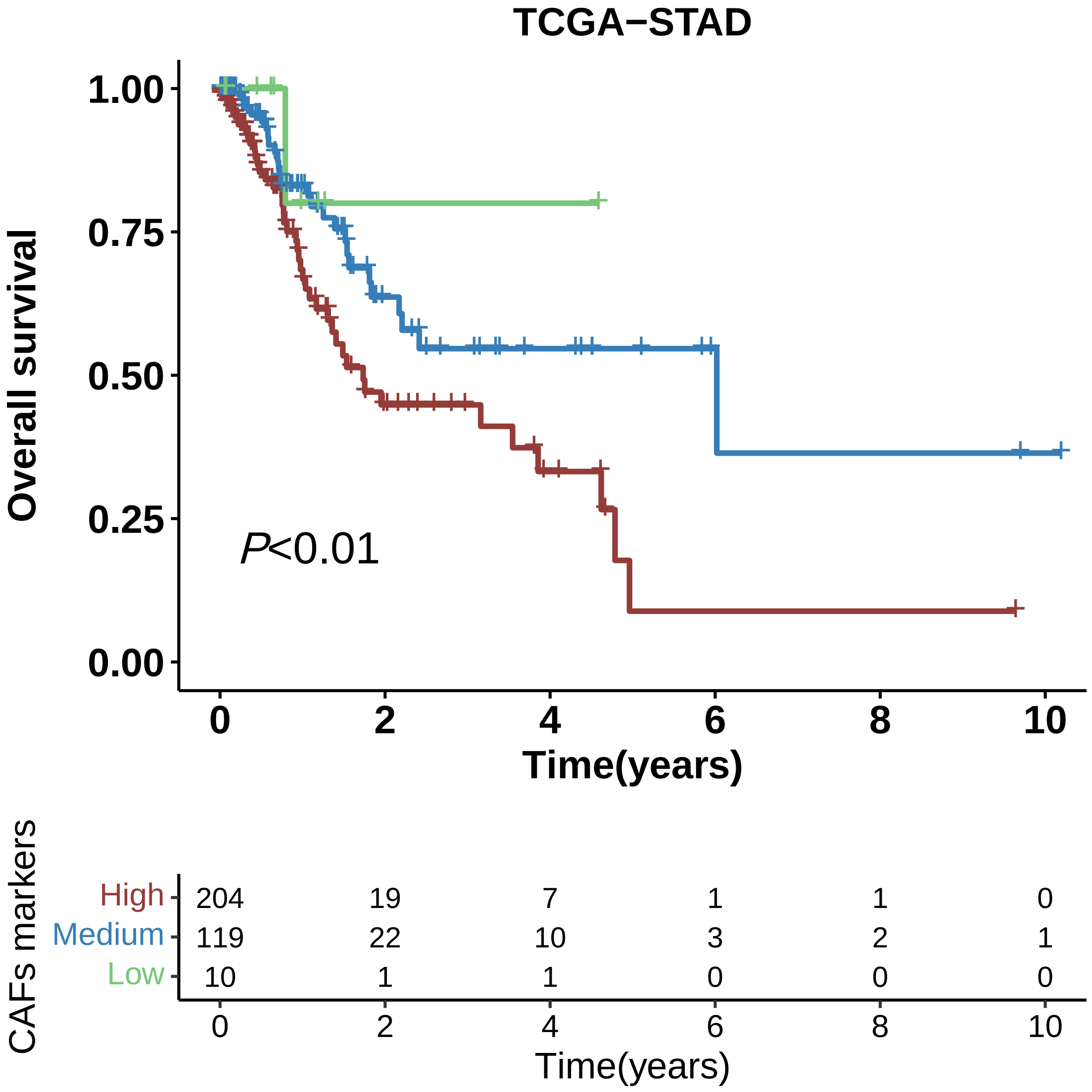
TCGA−STAD
Overall survival
1.00
0.75
0.50
0.25
0.00
P<0.01
0
2
4
6
8
10
Time(years)
CAFs markers
High
Medium
Low
204 19 7 1 1 0
119 22 10 3 2 1
10 1 1 0 0 0
Time(years)

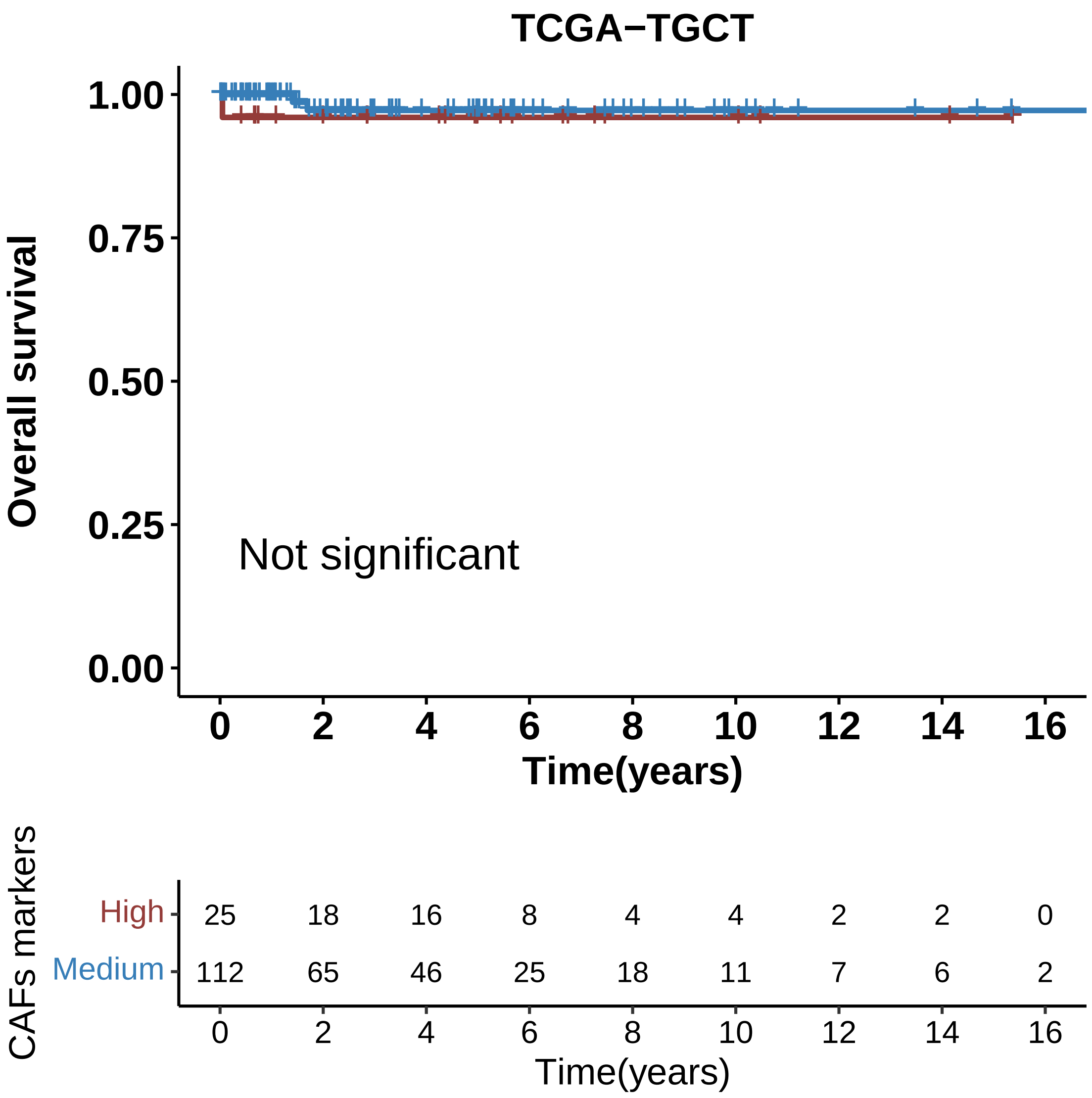

TCGA−TGCT
Overall survival
1.00
0.75
0.50
0.25
0.00
Not significant
0
2
4
6
8
10
12
14
16
Time(years)
CAFs markers
High
Medium
25 18 16 8 4 4 2 2 0
112 65 46 25 18 11 7 6 2
0
2
4
6
8
10
12
14
16
Time(years)

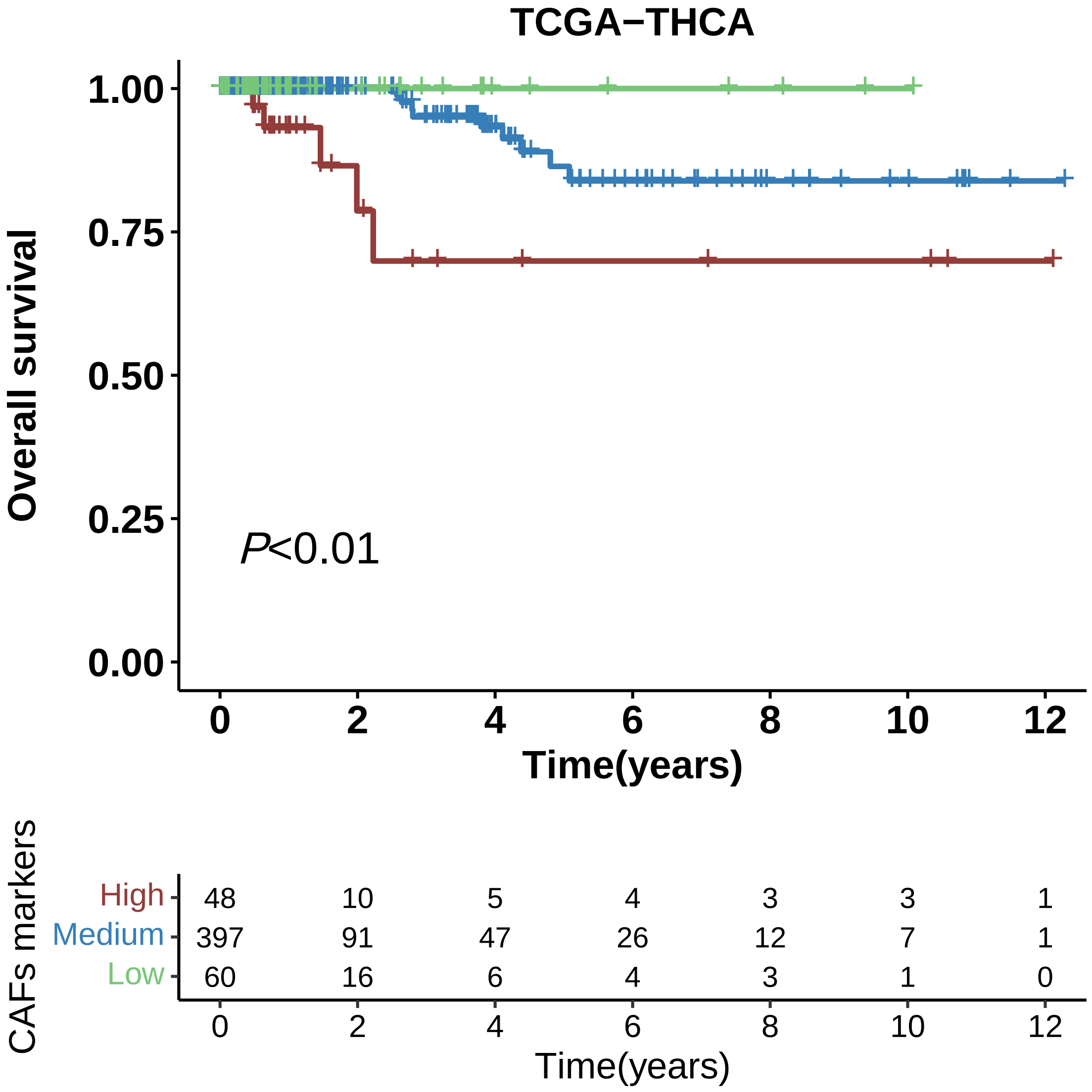
TCGA−THCA
Overall survival
1.00
0.75
0.50
0.25
0.00
P<0.01
0
2
4
6
8
10
12
Time(years)
CAFs markers
High
Medium
Low
48 10 5 4 3 3 1
397 91 47 26 12 7 1
60 16 6 4 3 1 0
Time(years)

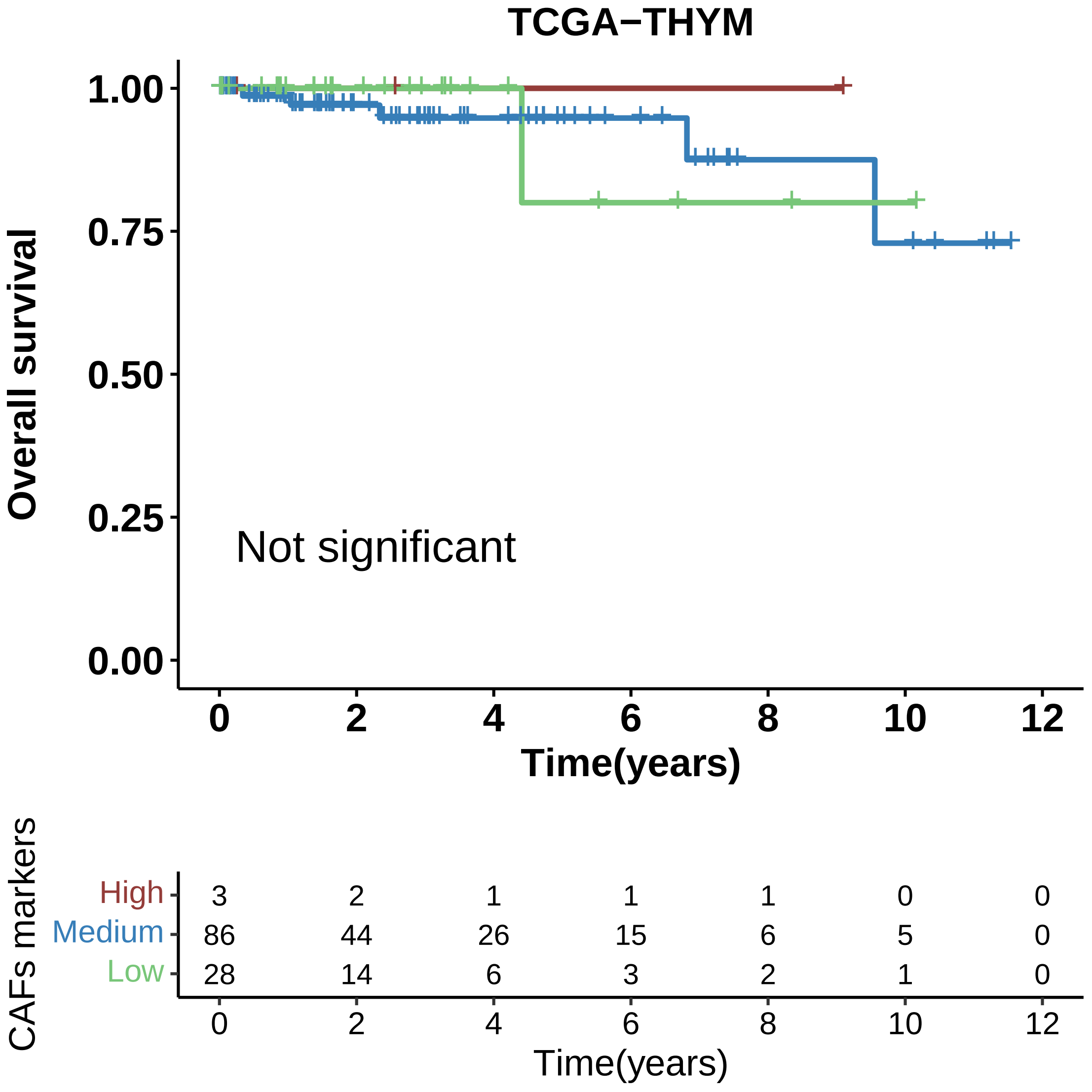

TCGA−THYM
Overall survival
1.00
0.75
0.50
0.25
0.00
Not significant
0
2
4
6
8
10
12
Time(years)
CAFs markers
High
Medium
Low
3 2 1 1 1 0 0
86 44 26 15 6 5 0
28 14 6 3 2 1 0
0
2
4
6
8
10
12
Time(years)

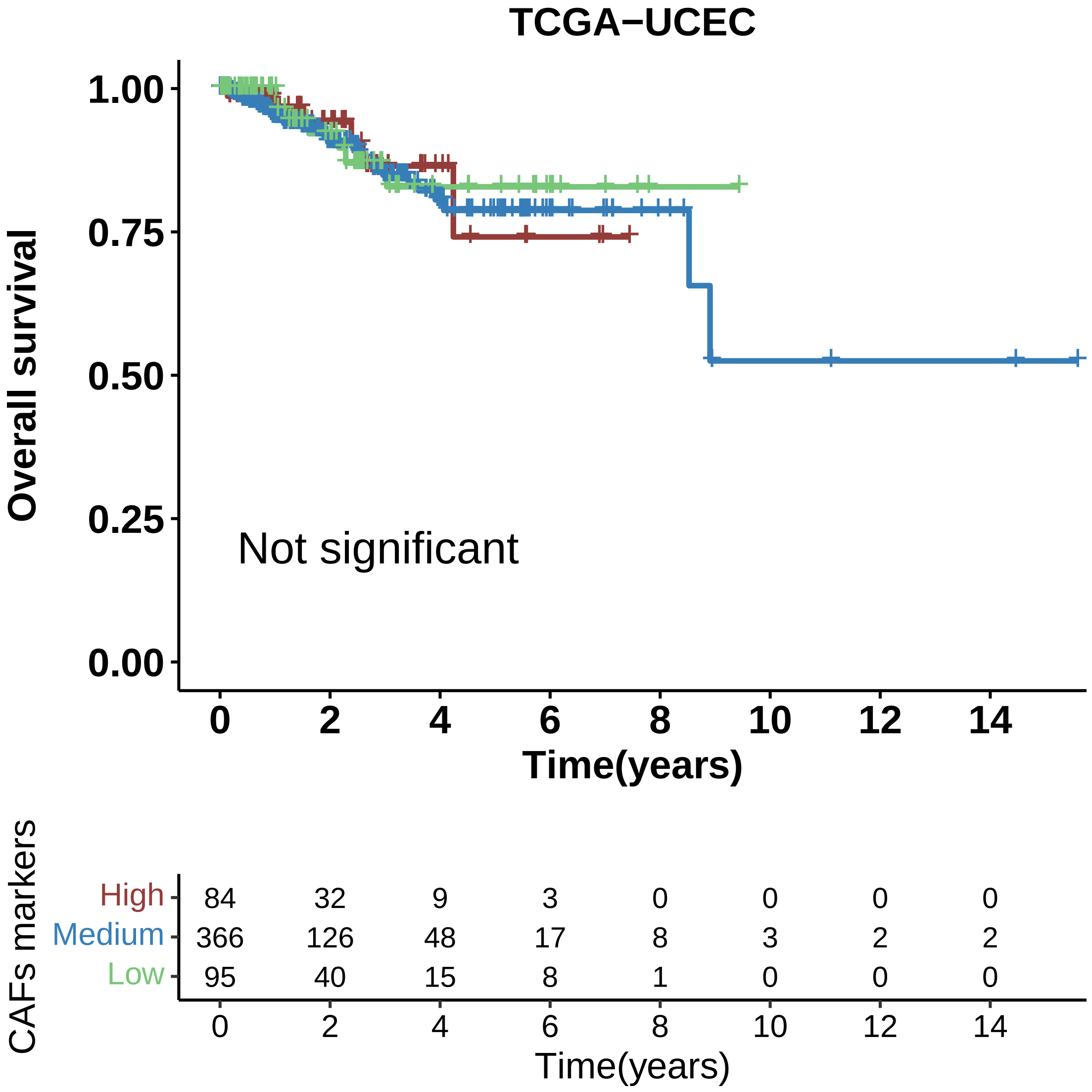
TCGA−UCEC
Overall survival
1.00
0.75
0.50
0.25
0.00
Not significant
0
2
4
6
8
10
12
14
Time(years)
CAFs markers
High
Medium
Low
84 32 9 3 0 0 0 0
366 126 48 17 8 3 2 2
95 40 15 8 1 0 0 0
Time(years)

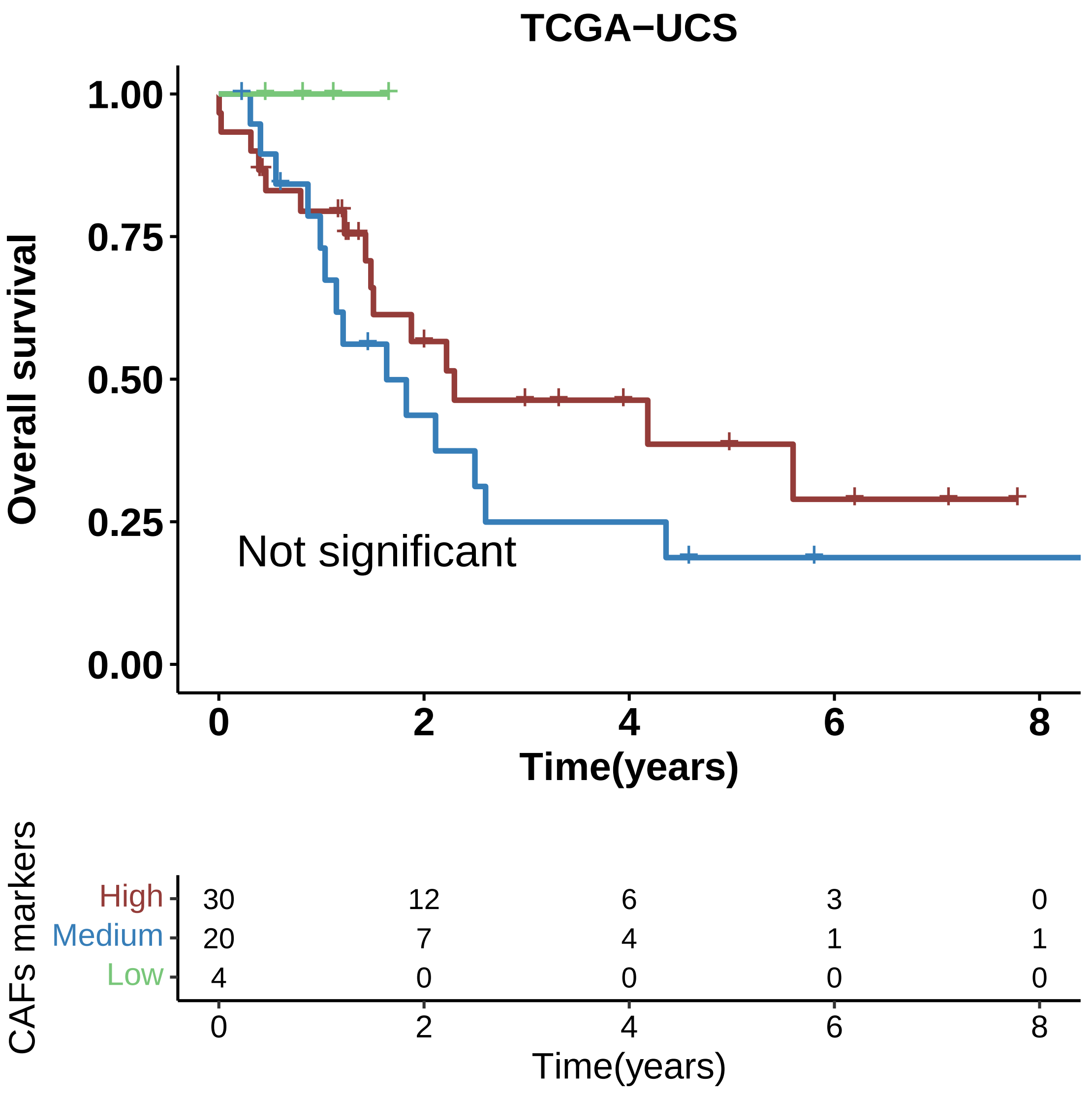
TCGA−UCS
Overall survival
1.00
0.75
0.50
0.25
0.00
Not significant
0
2
4
6
8
Time(years)
CAFs markers
High
Medium
Low
30
12
6
3
0
20
7
4
1
1
4
0
0
0
0
0
2
4
6
8
Time(years)

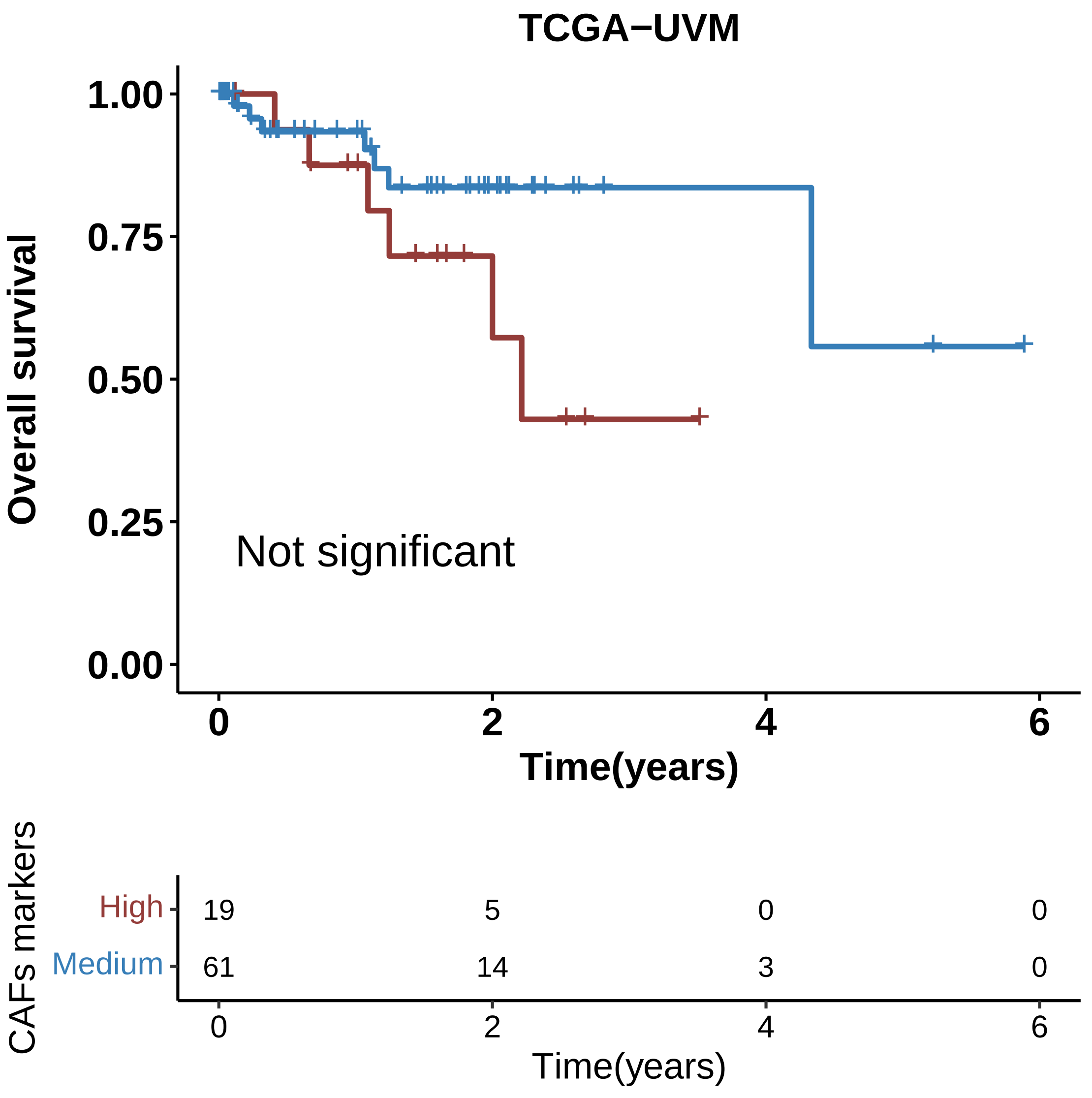

TCGA−UVM
Overall survival
1.00
0.75
0.50
0.25
0.00
Not significant
0
2
4
6
Time(years)
CAFs markers
High
Medium
19
5
0
0
61
14
3
0
0
2
4
6
Time(years)
