# Supplementary figures and images for: Dissecting the role of cancer‐associated fibroblast‐derived biglycan as a potential therapeutic target in immunotherapy resistance: A tumor bulk and single‐cell transcriptomic study
Source: Clin Transl Med. 2023 Feb 11;13(2):e1189. doi: 10.1002/ctm2.1189 (PMC9920016; doi:10.1002/ctm2.1189)

# NSCLC

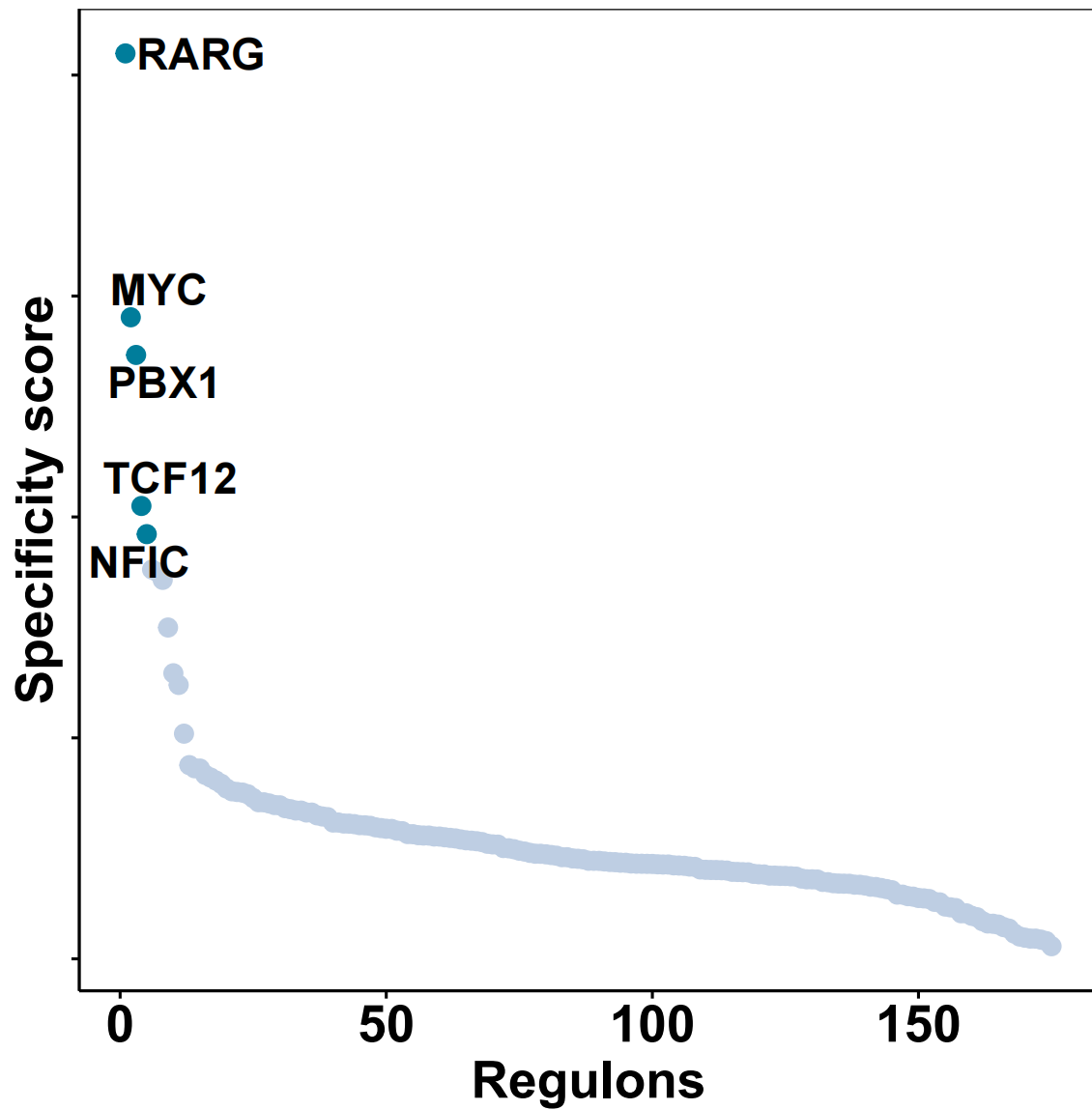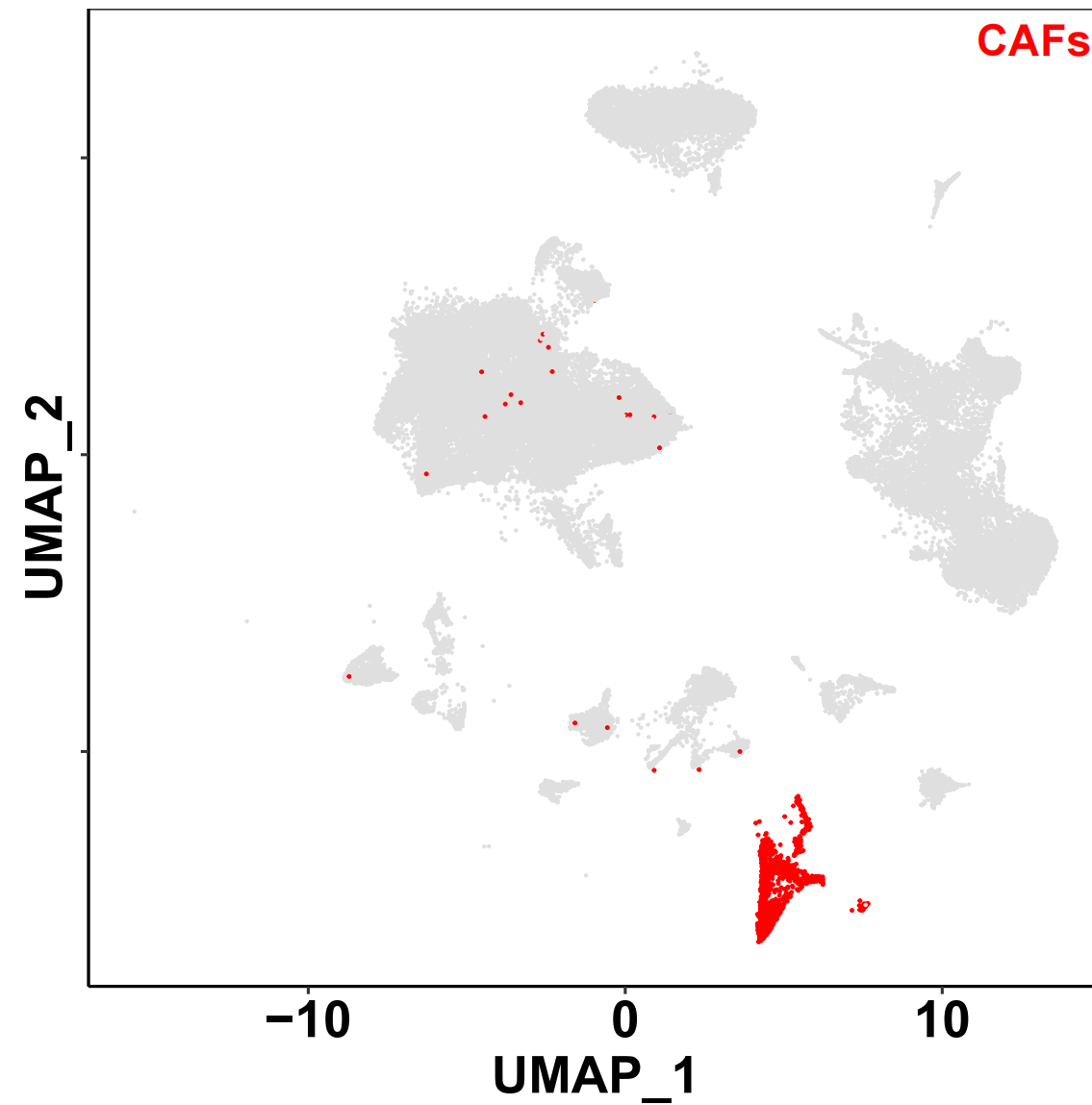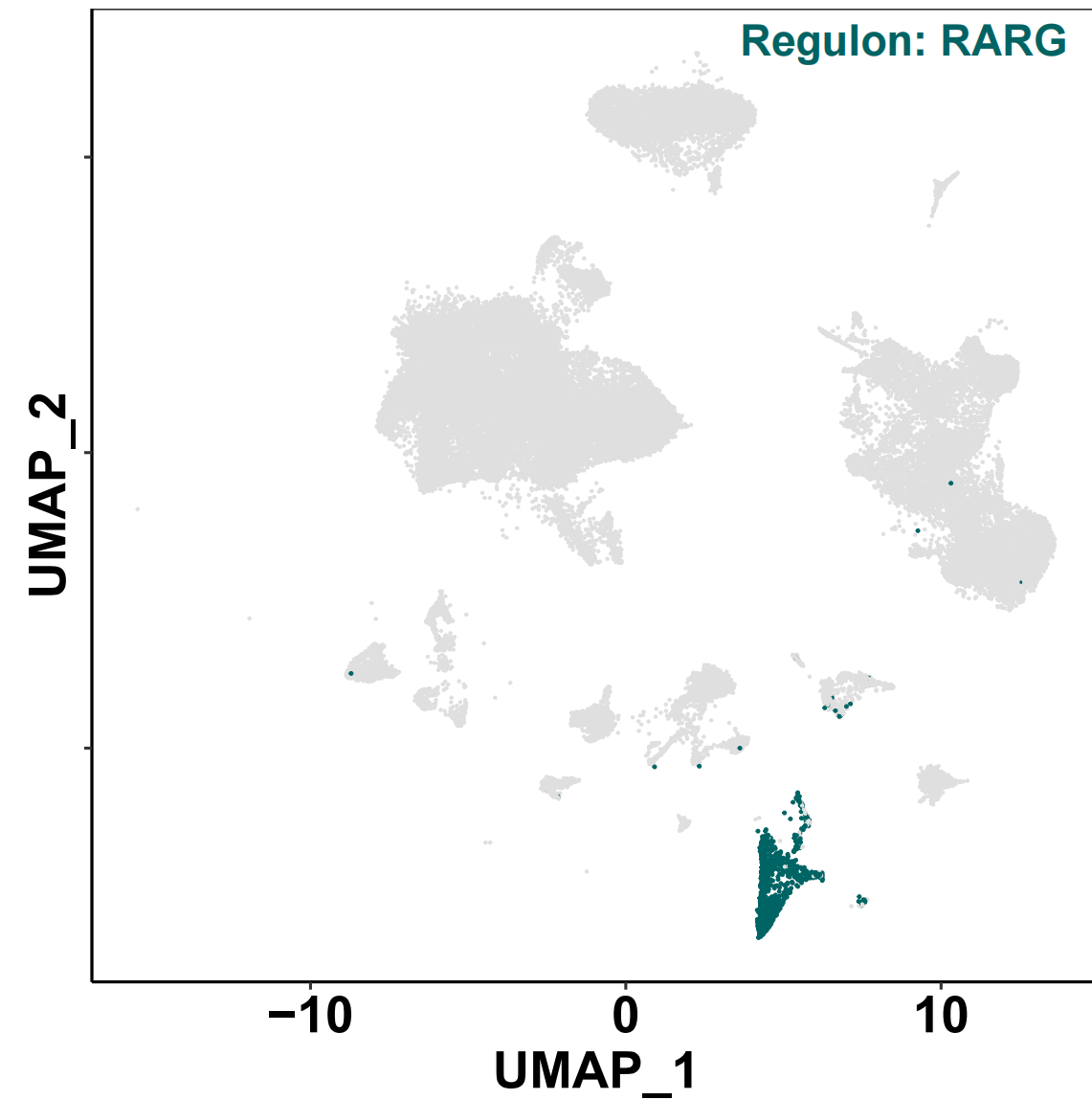

# BCC

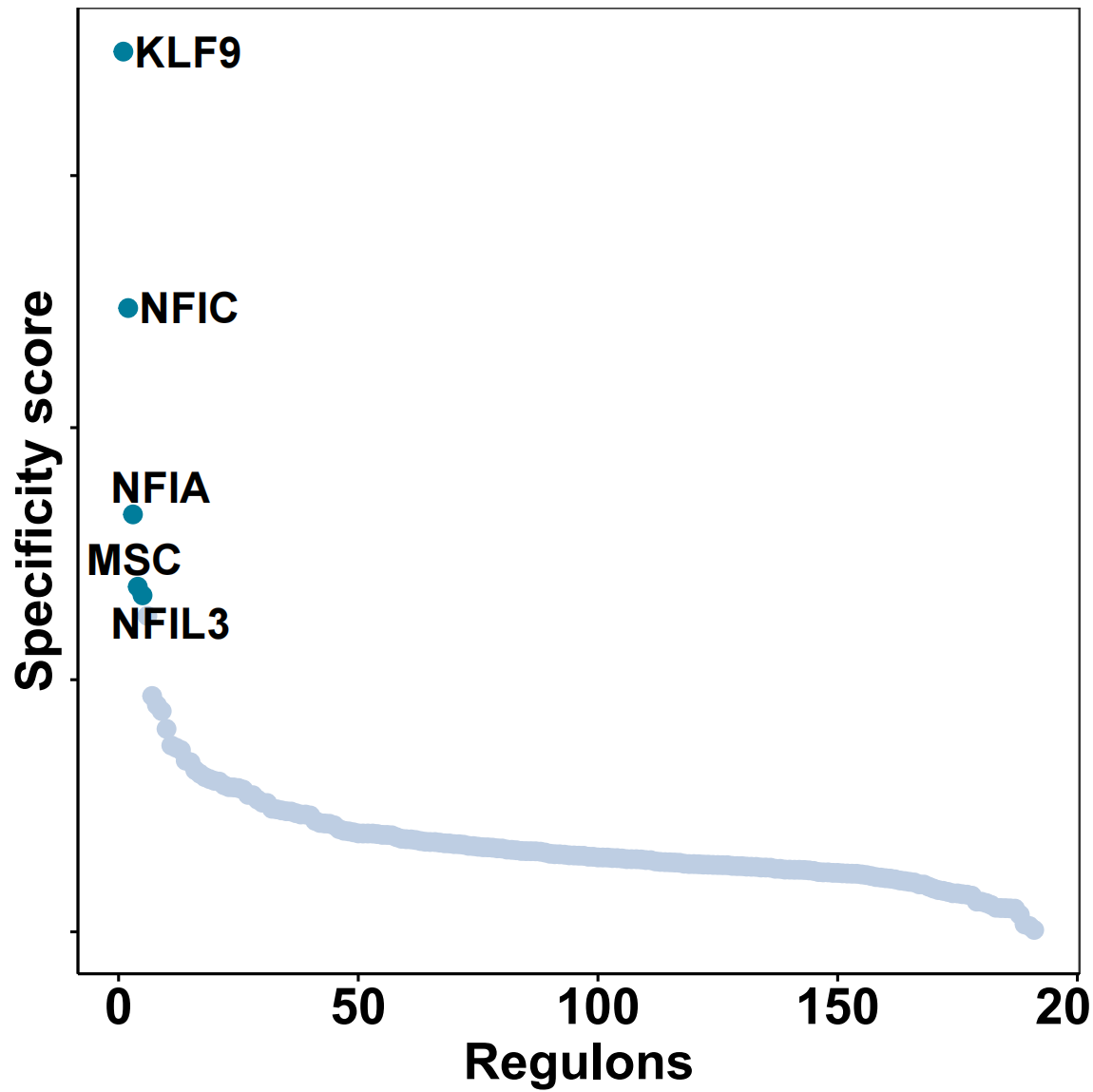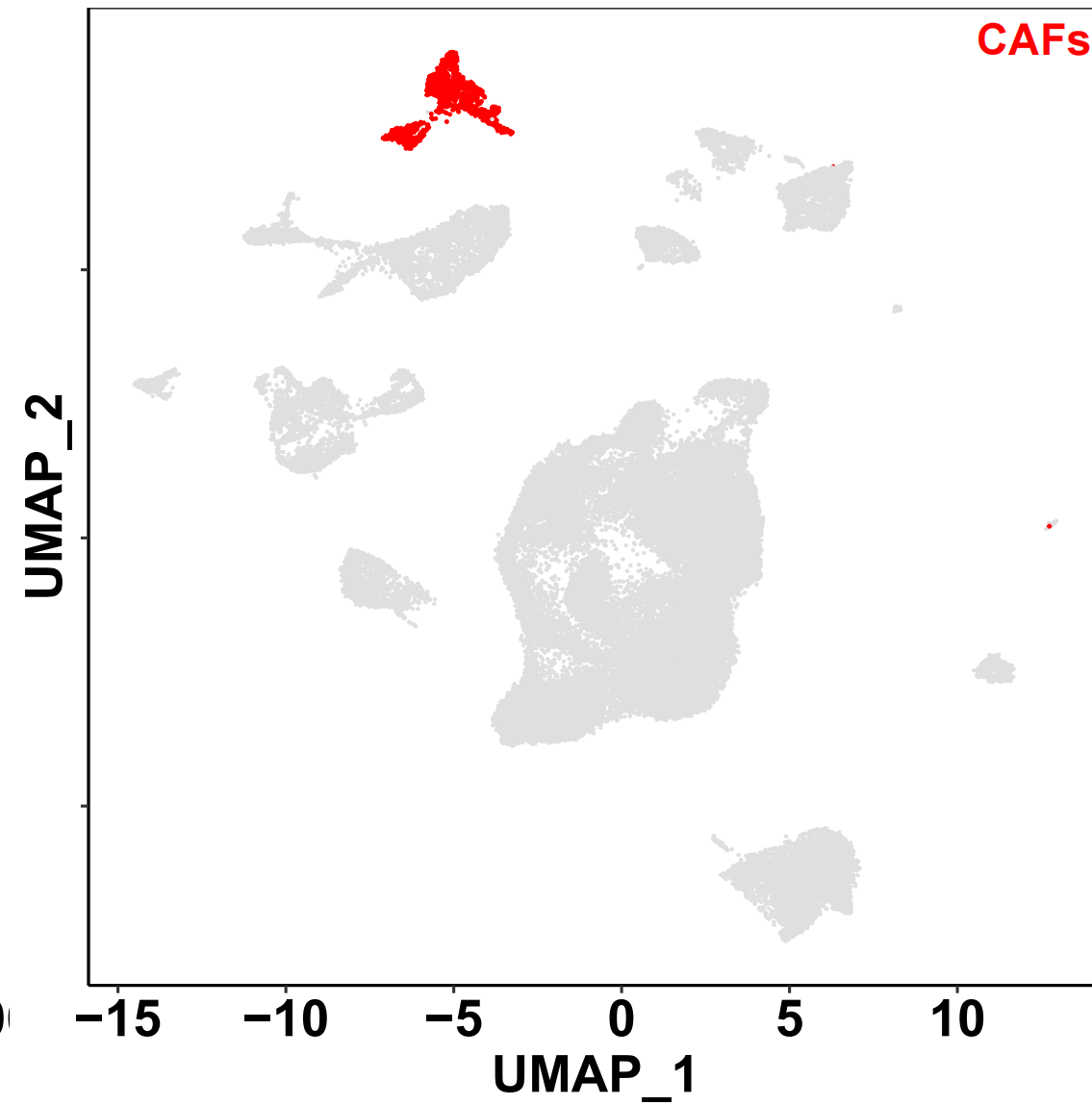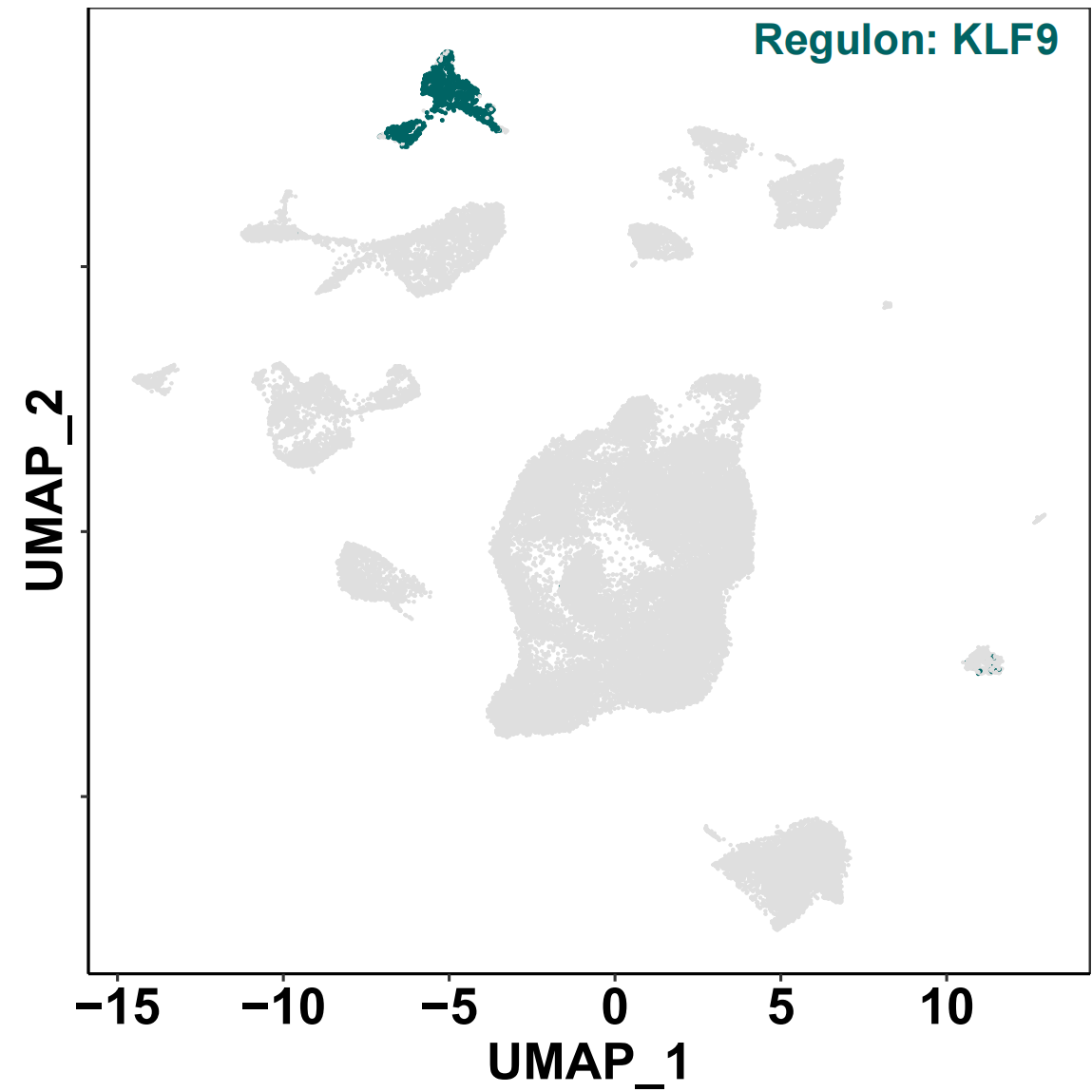

# BLCA

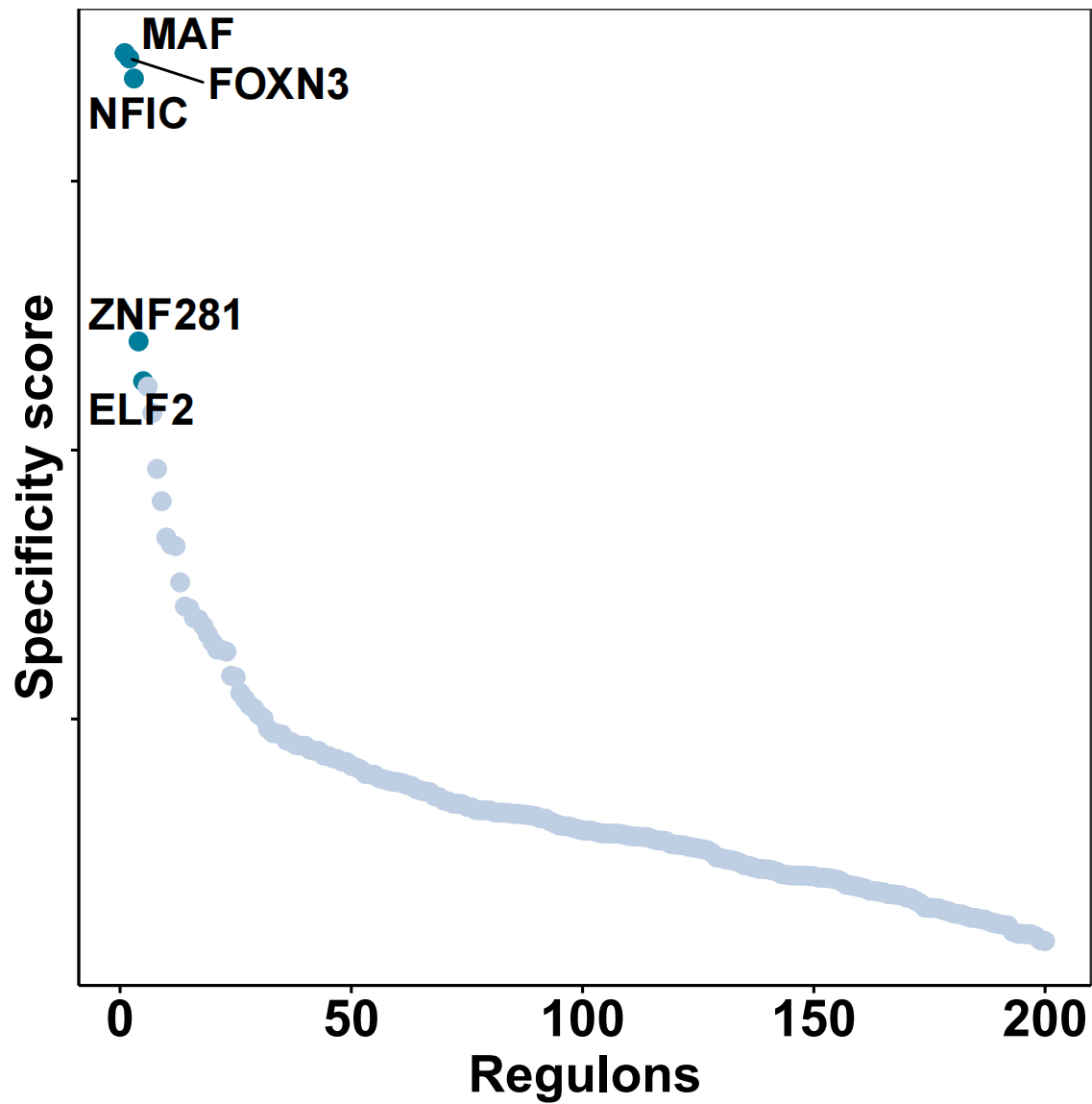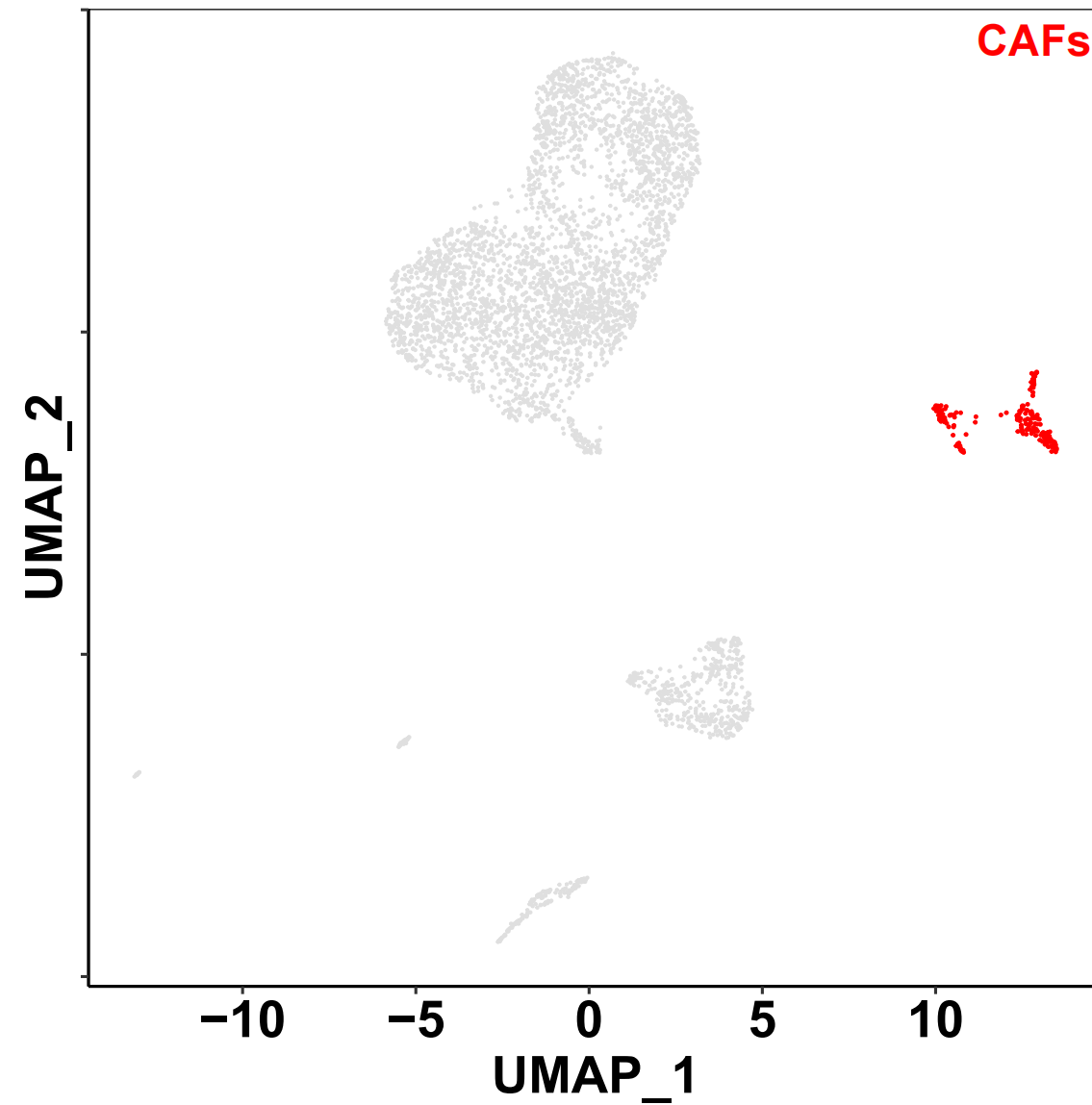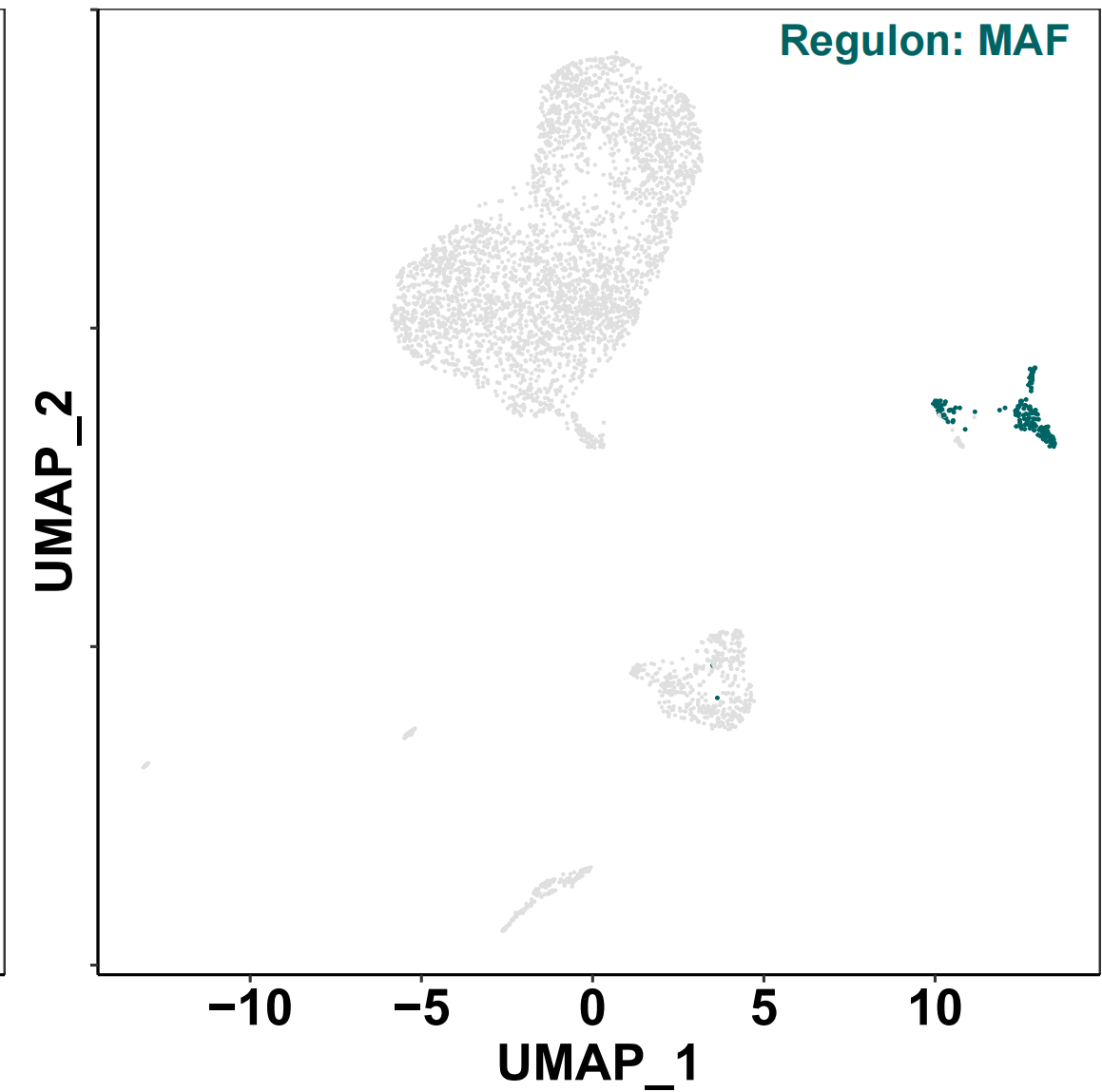

# BRCA

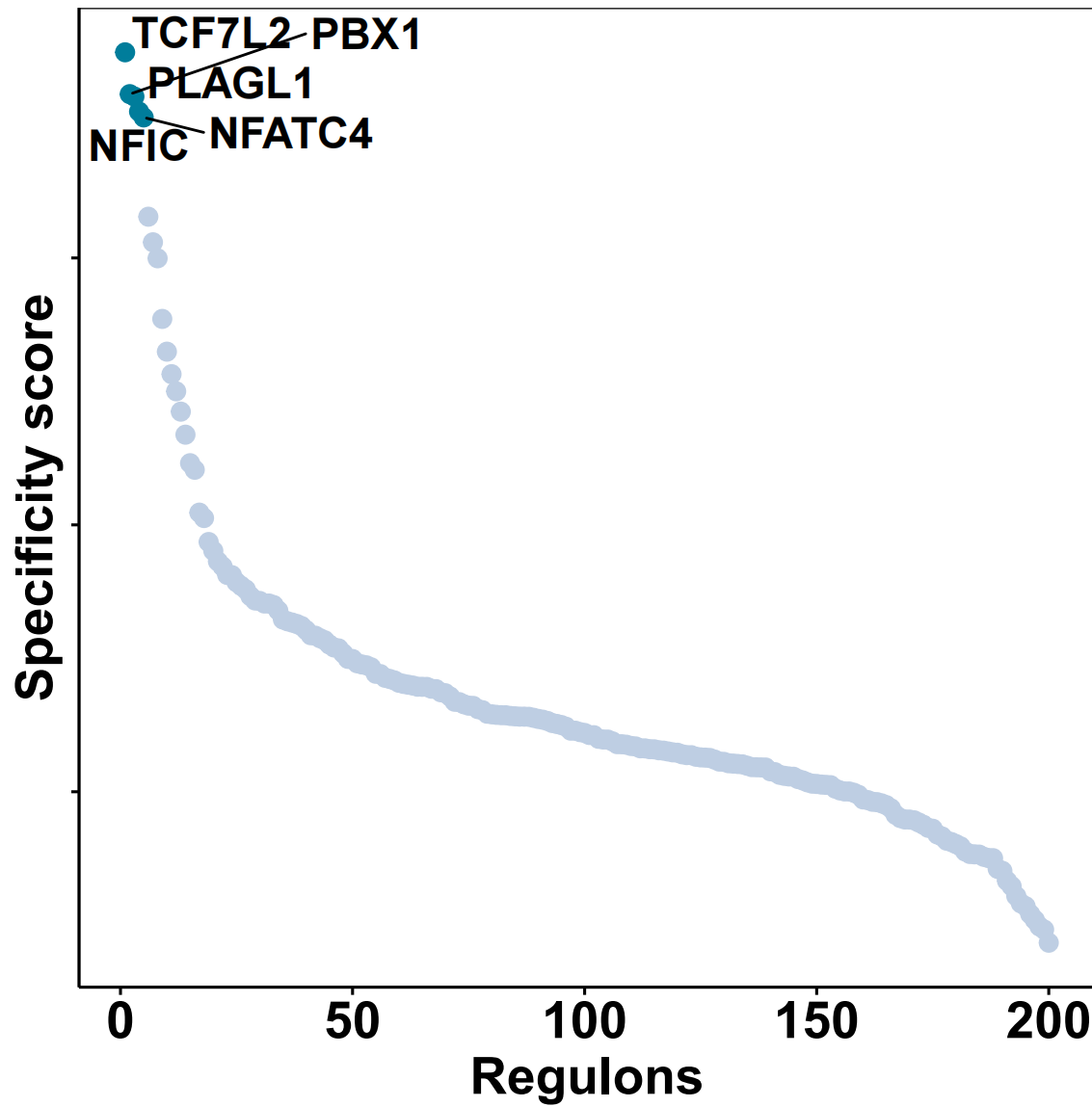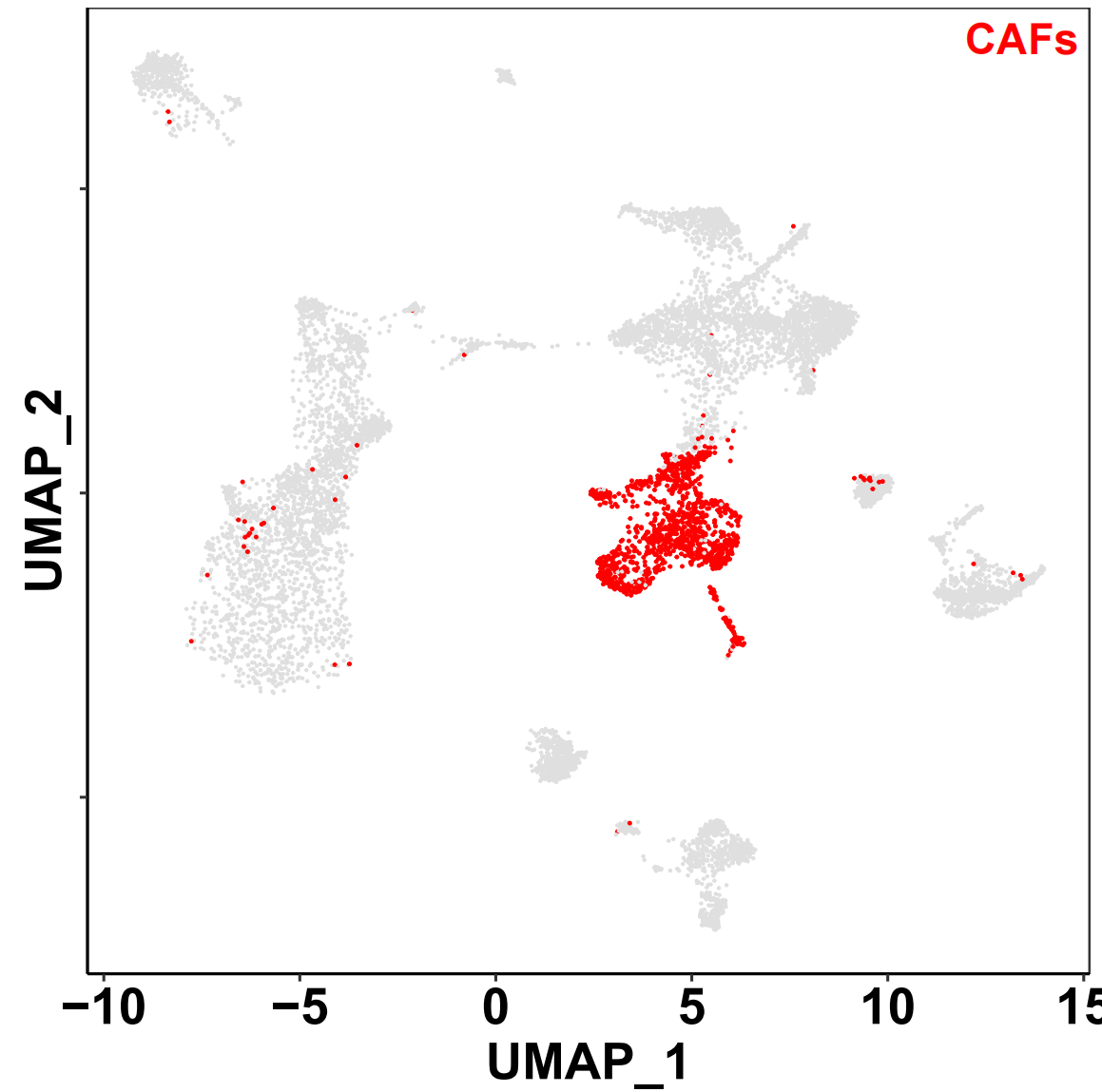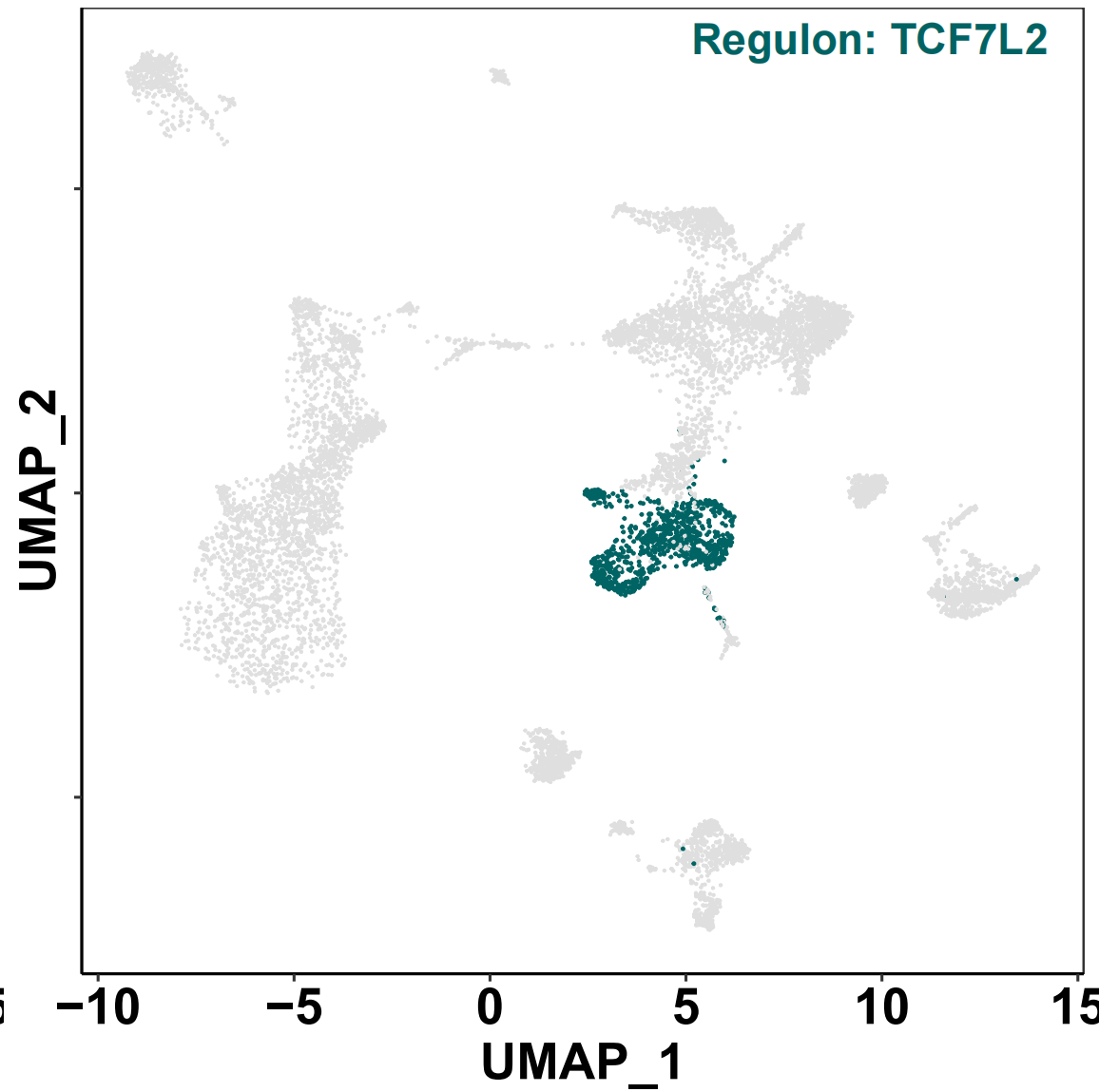

# CHOL

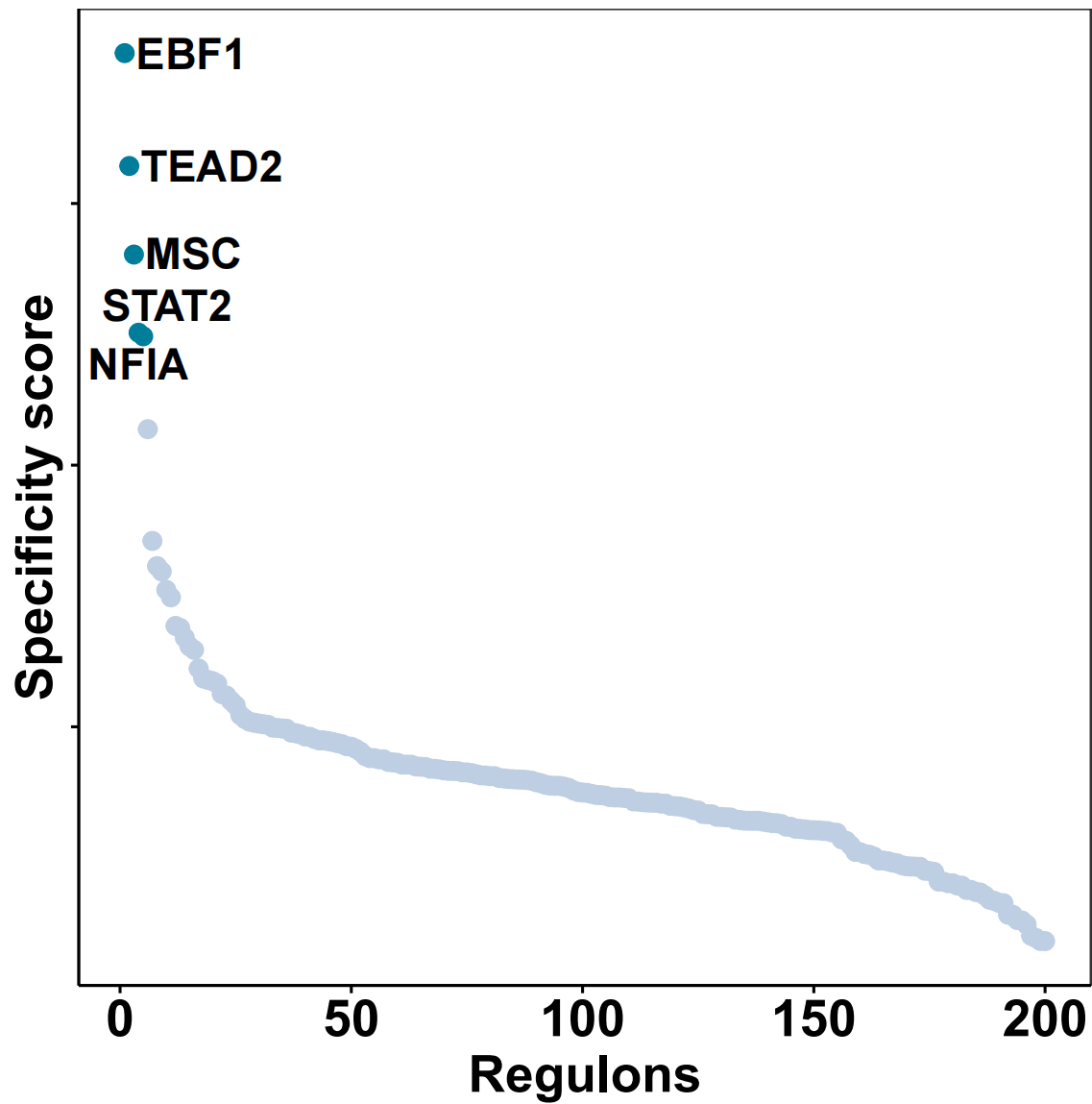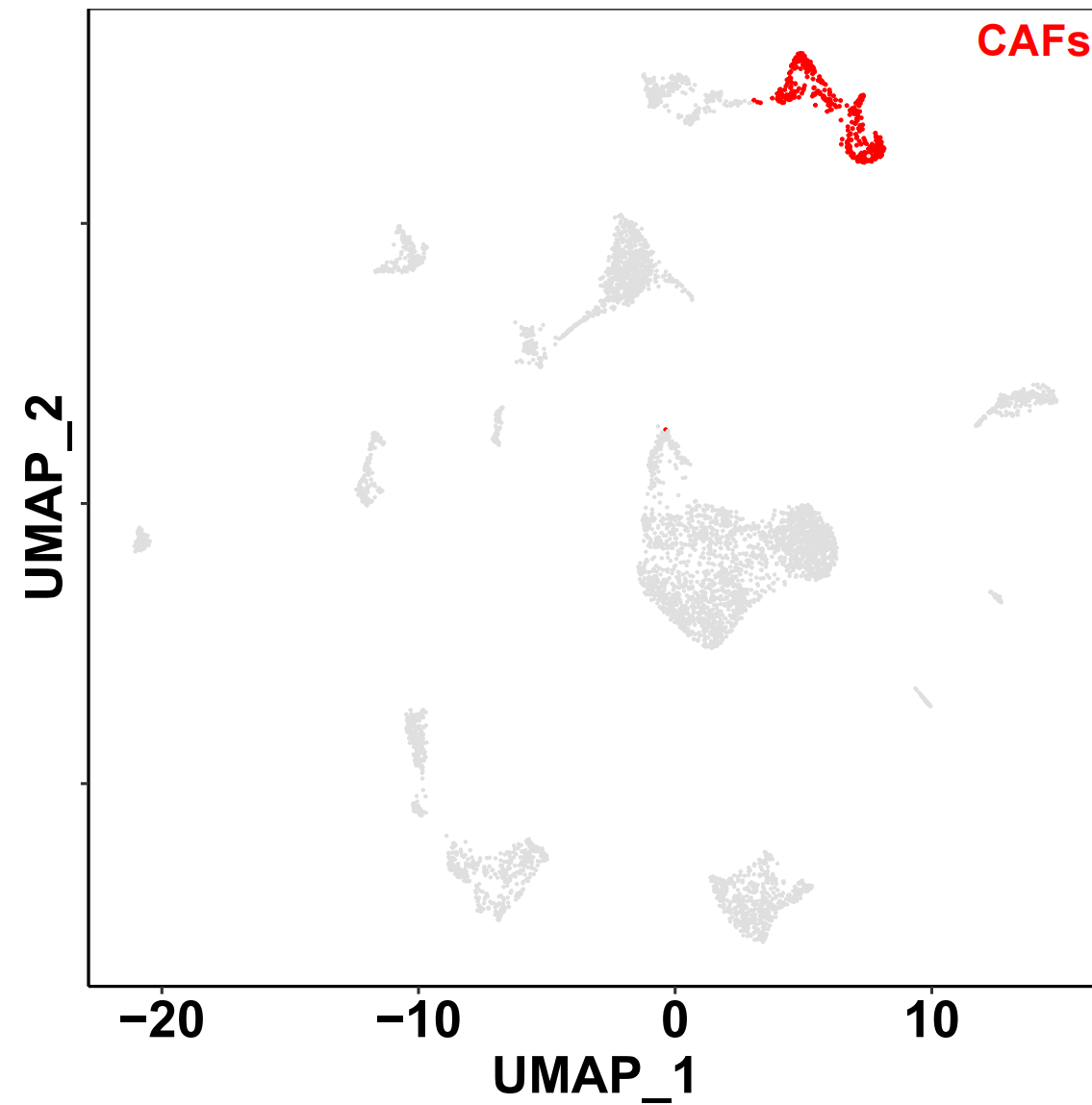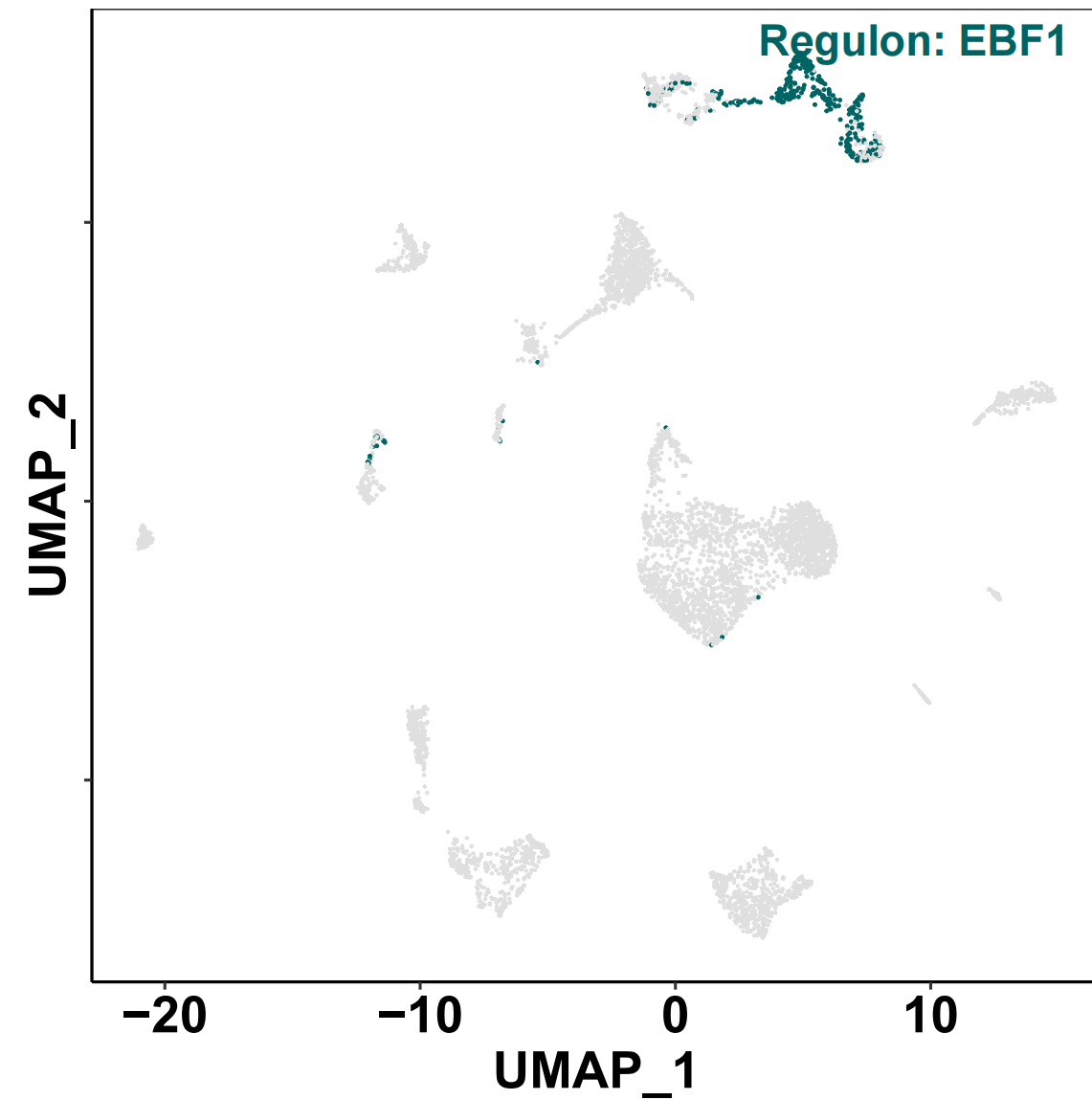

# CRC

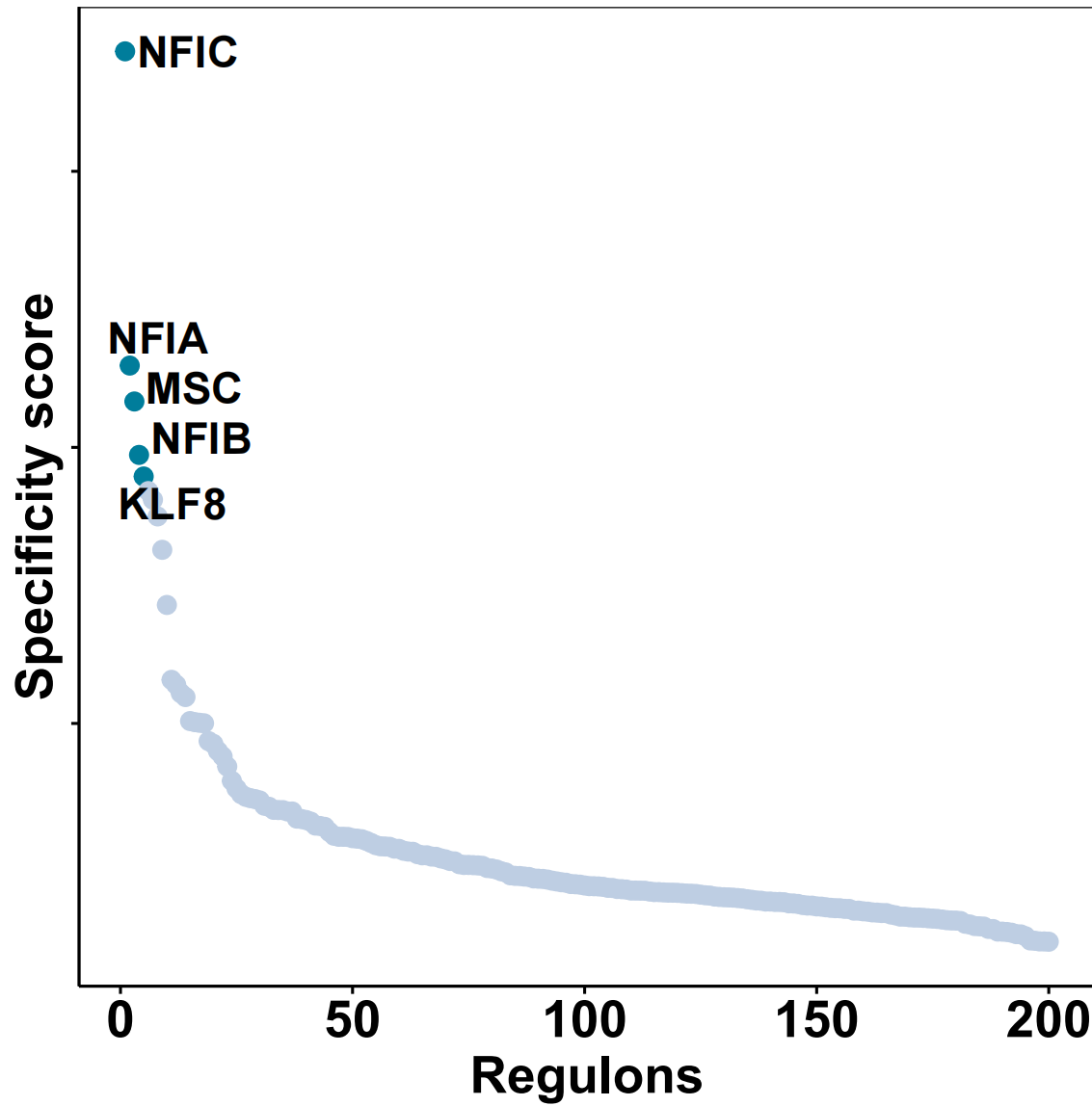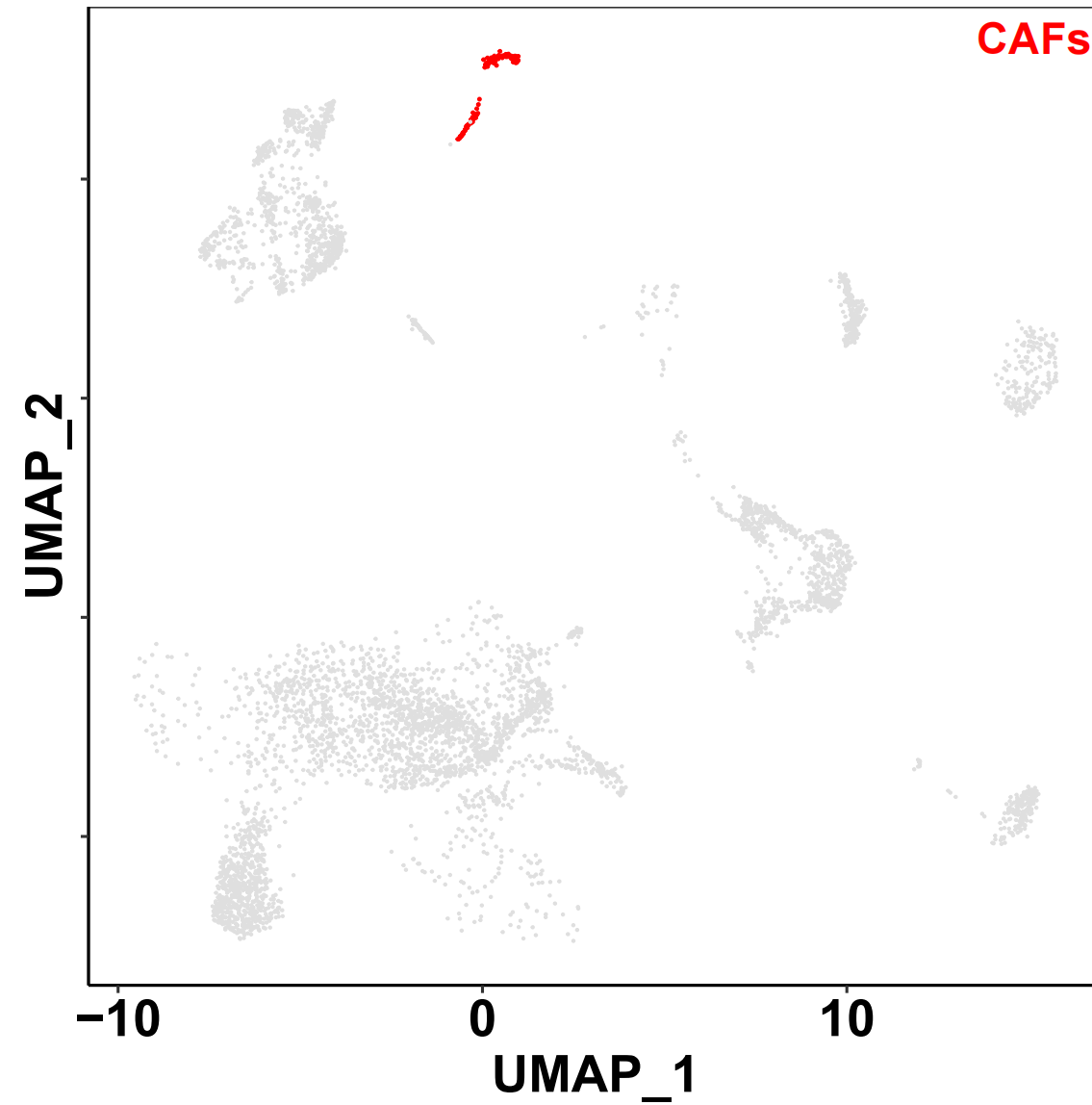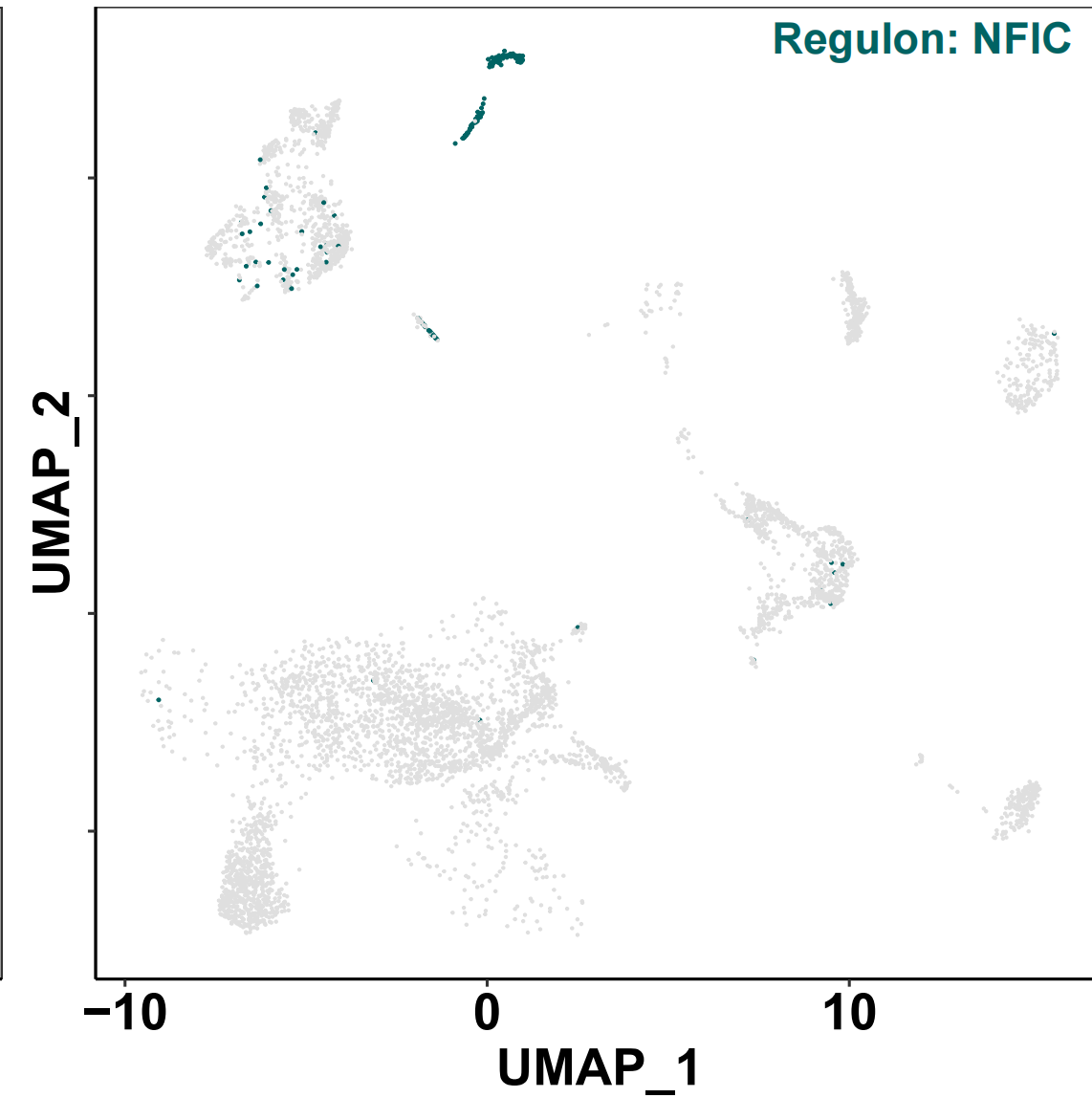

# HNSC

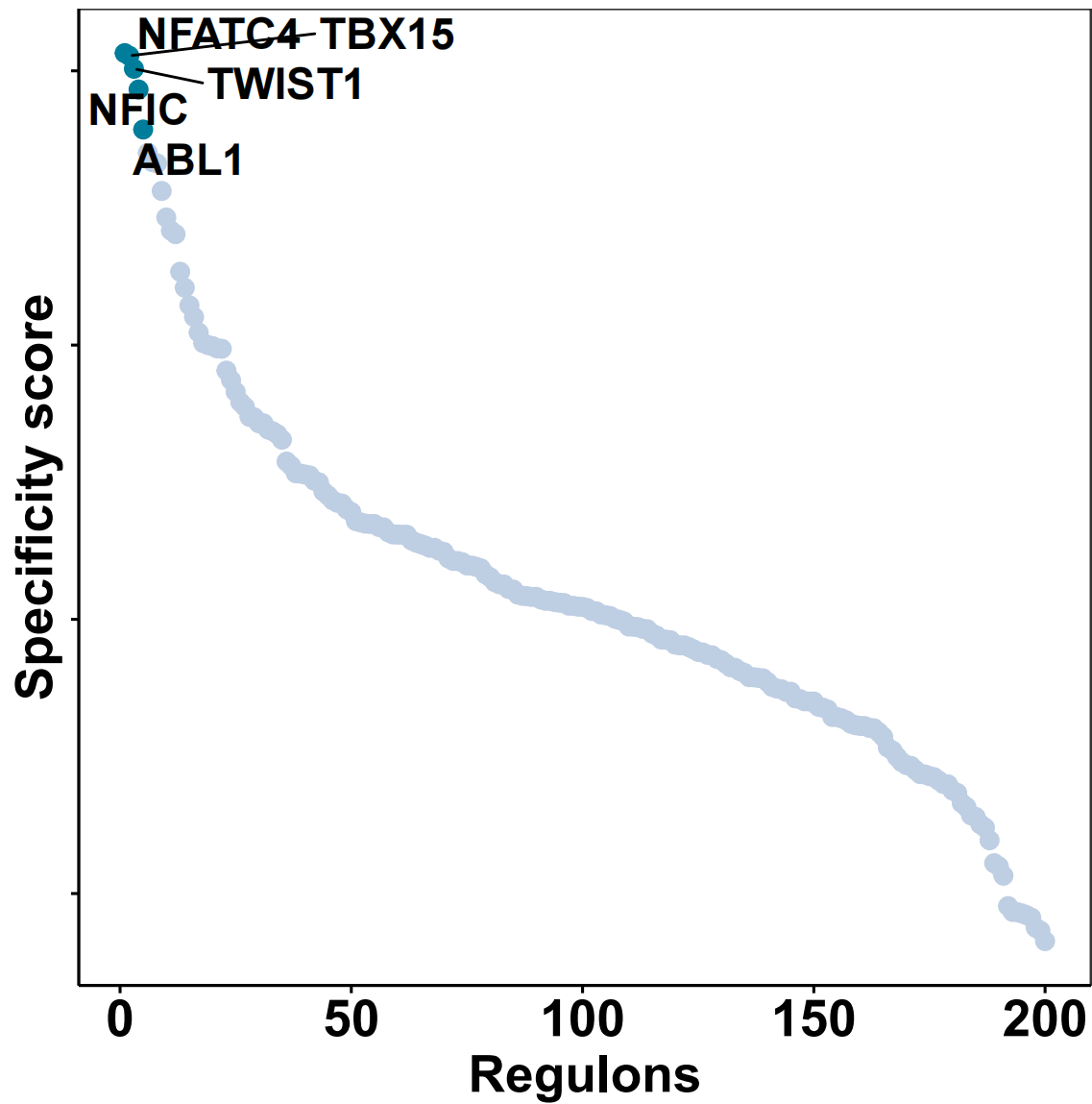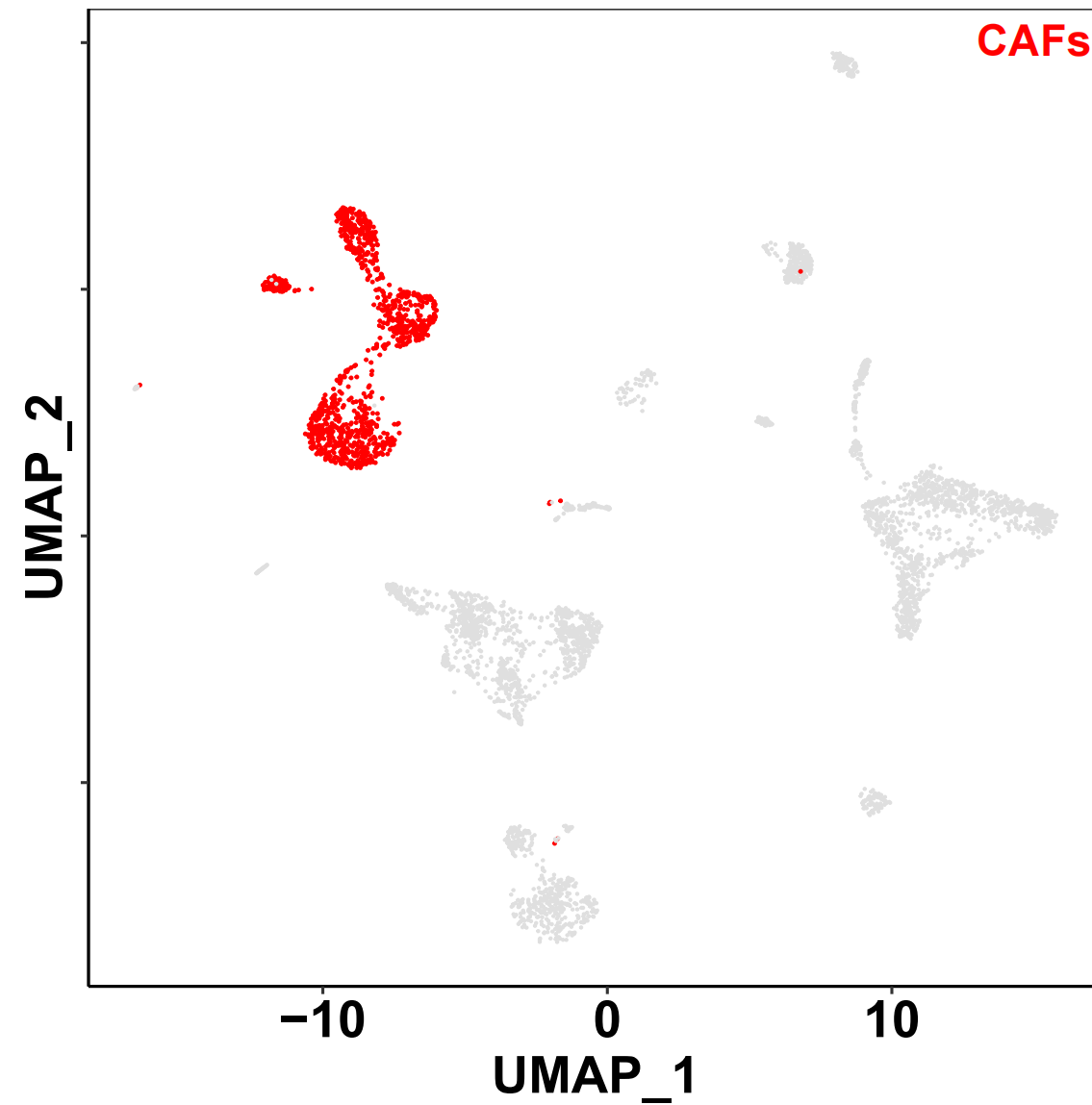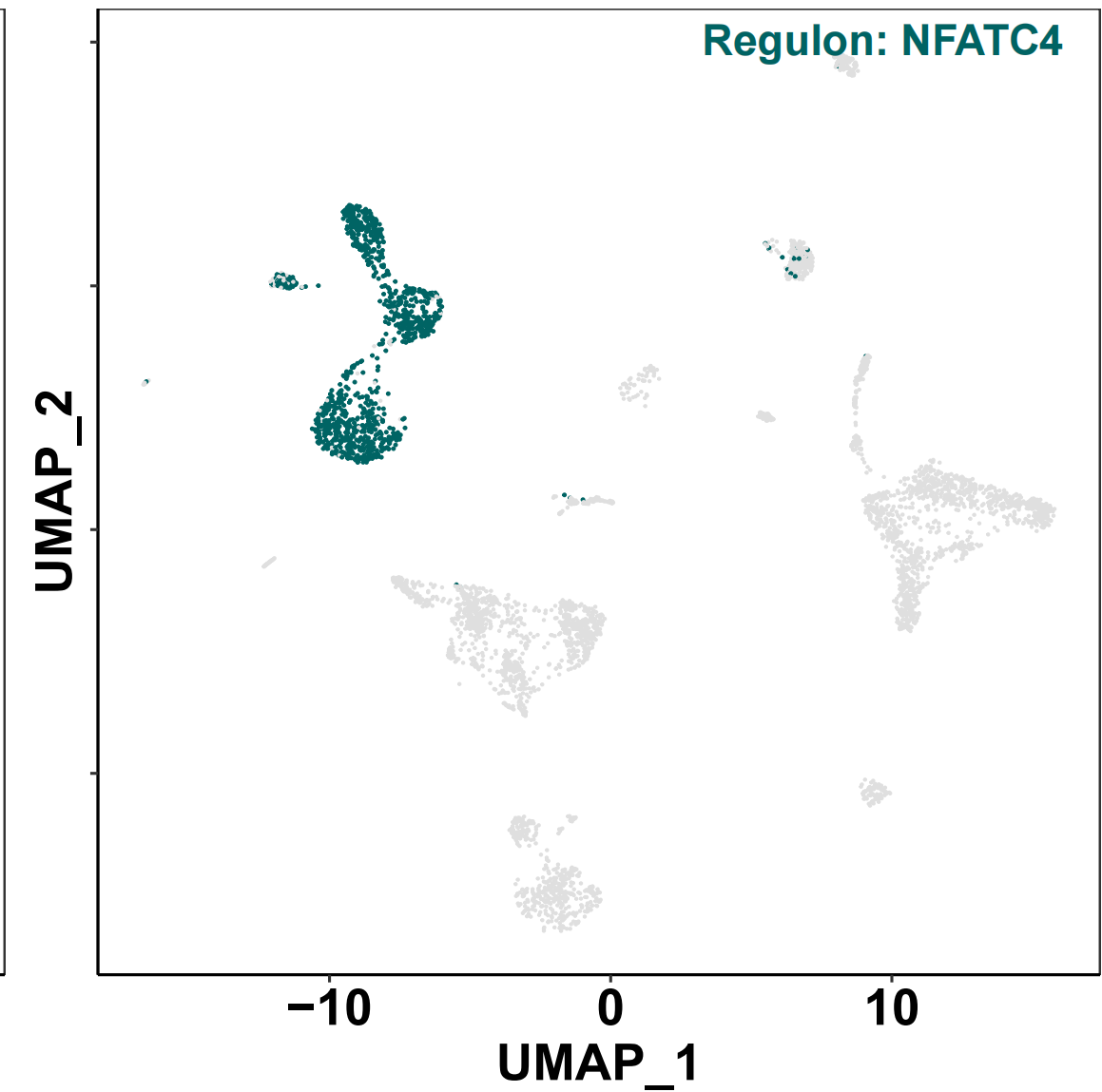

# LIHC

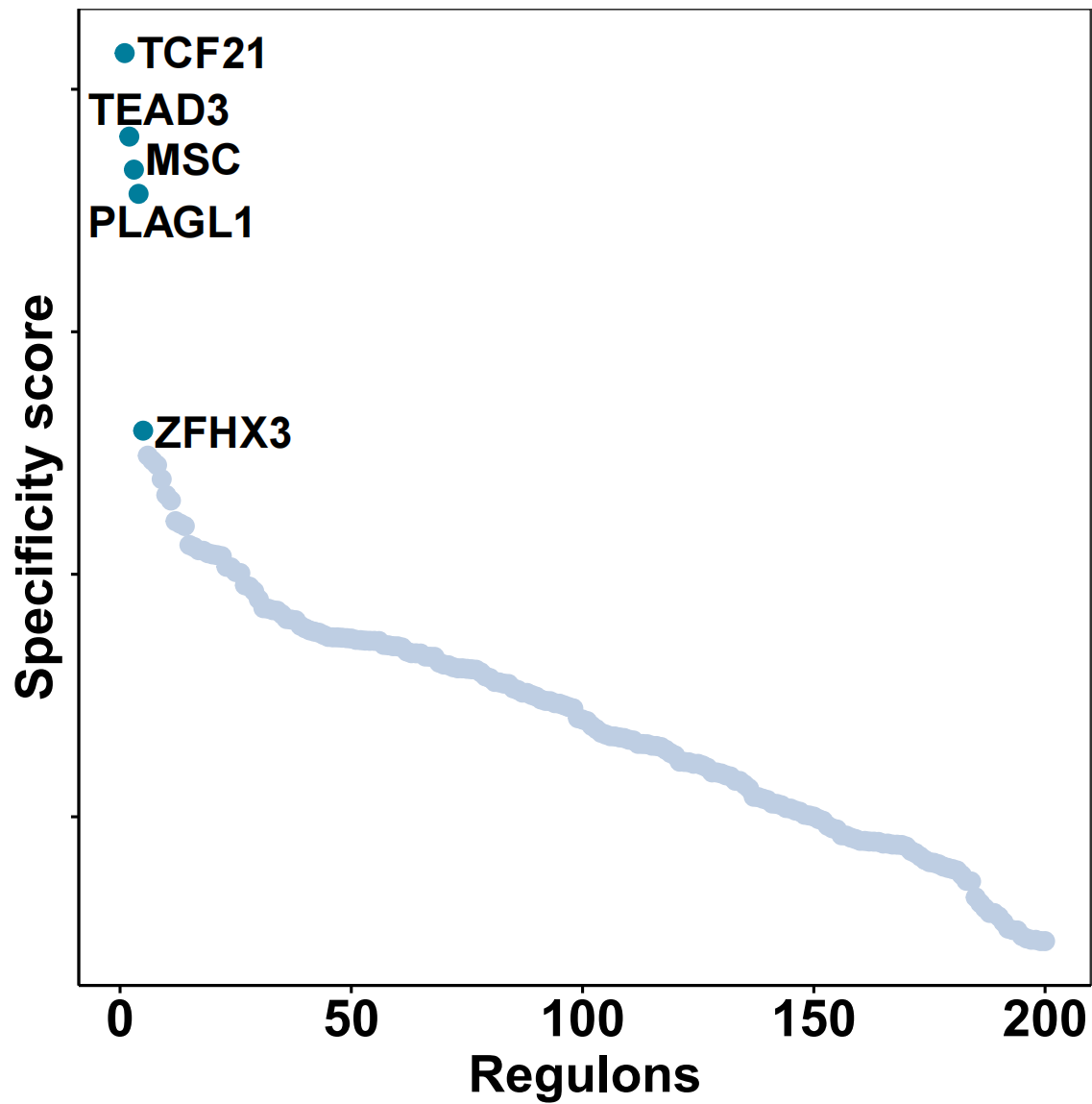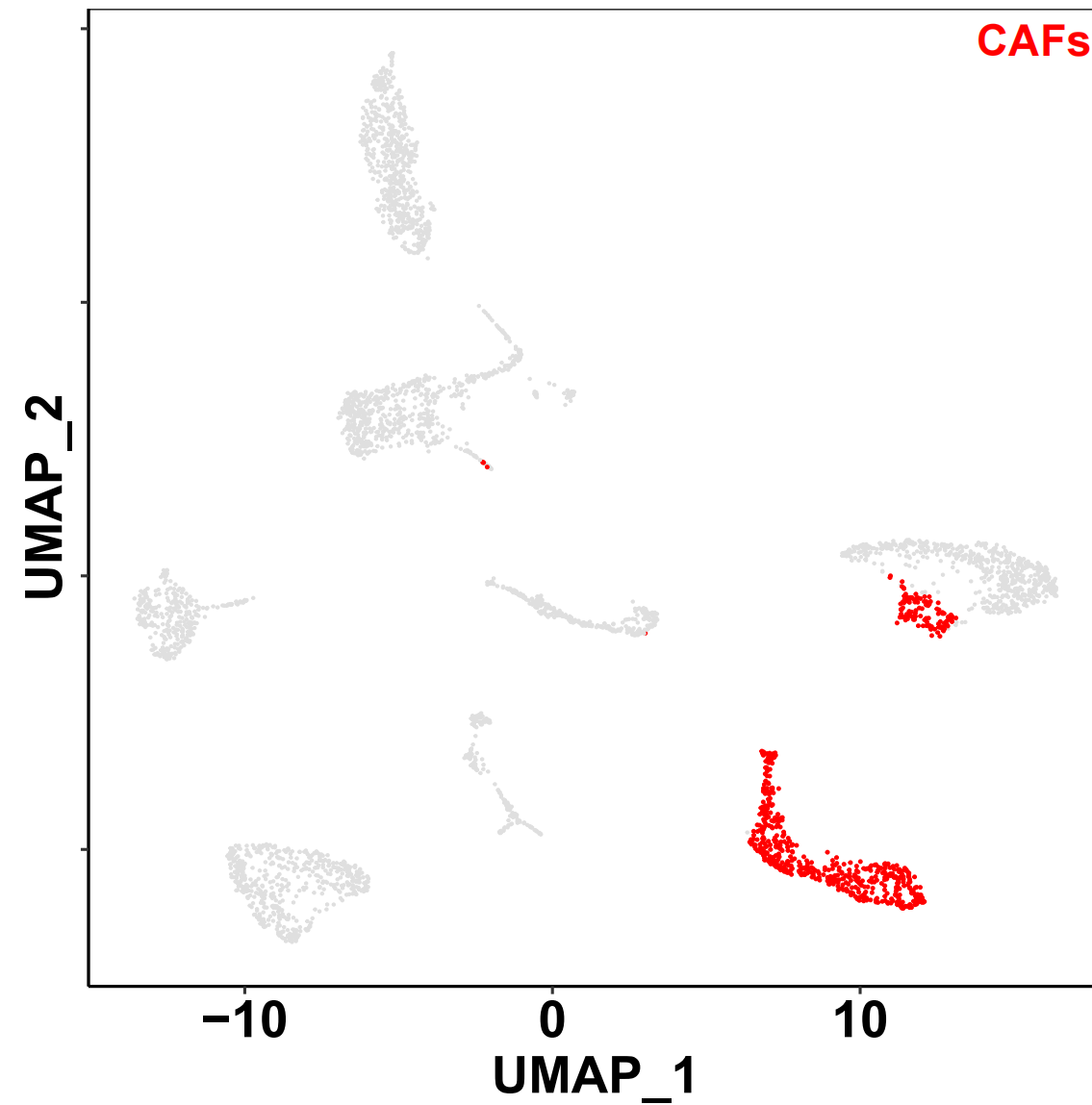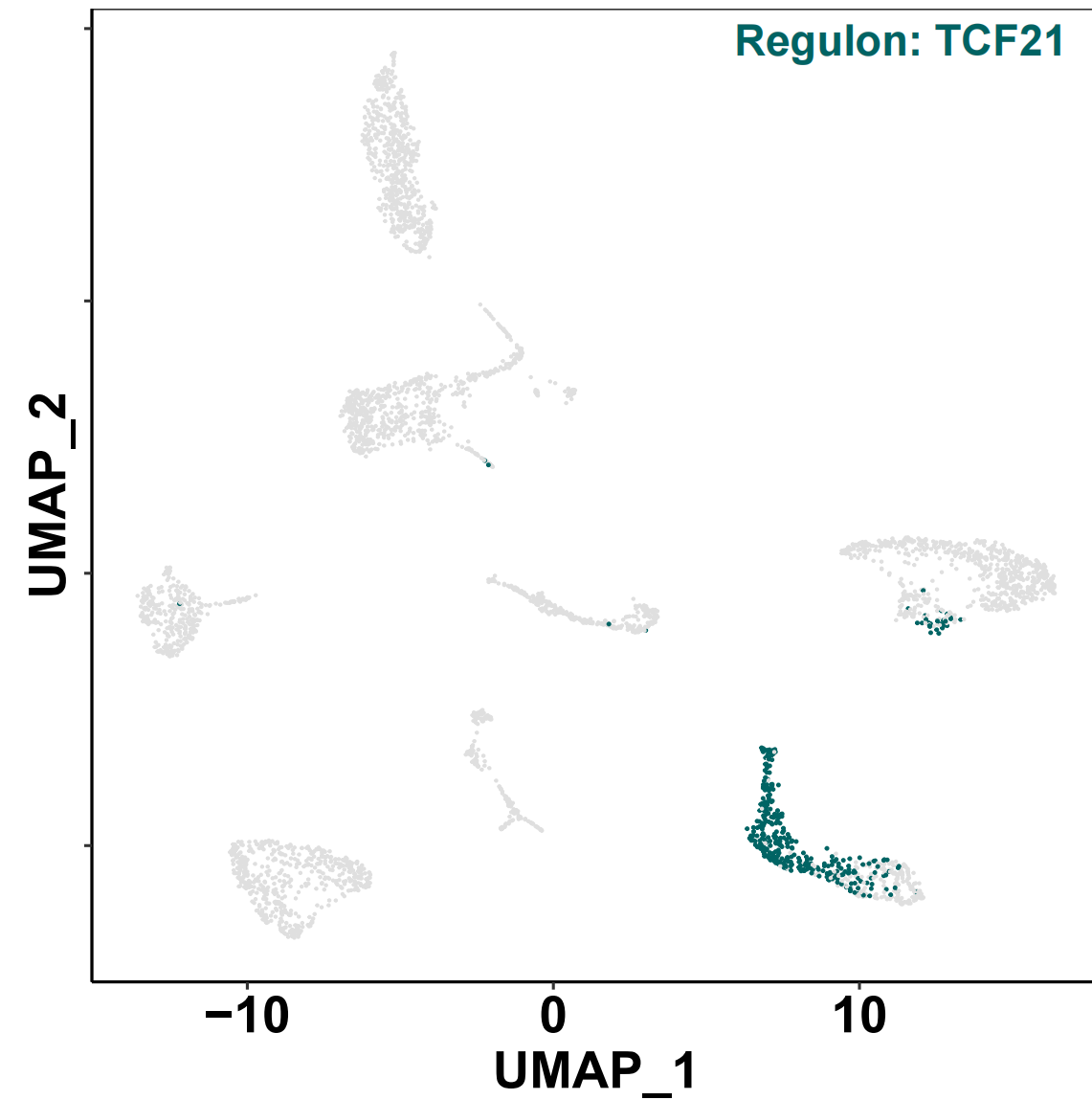

# NET

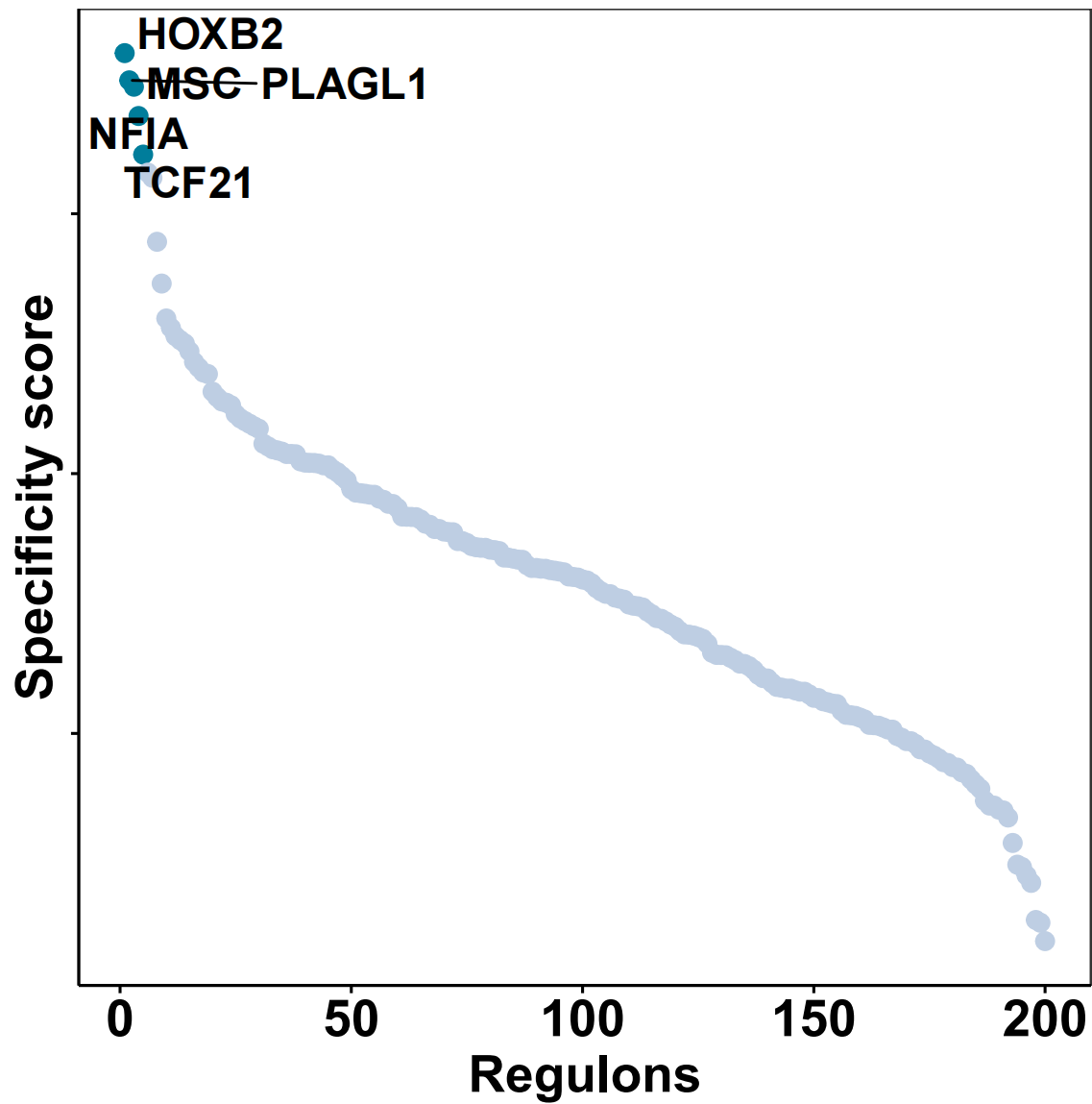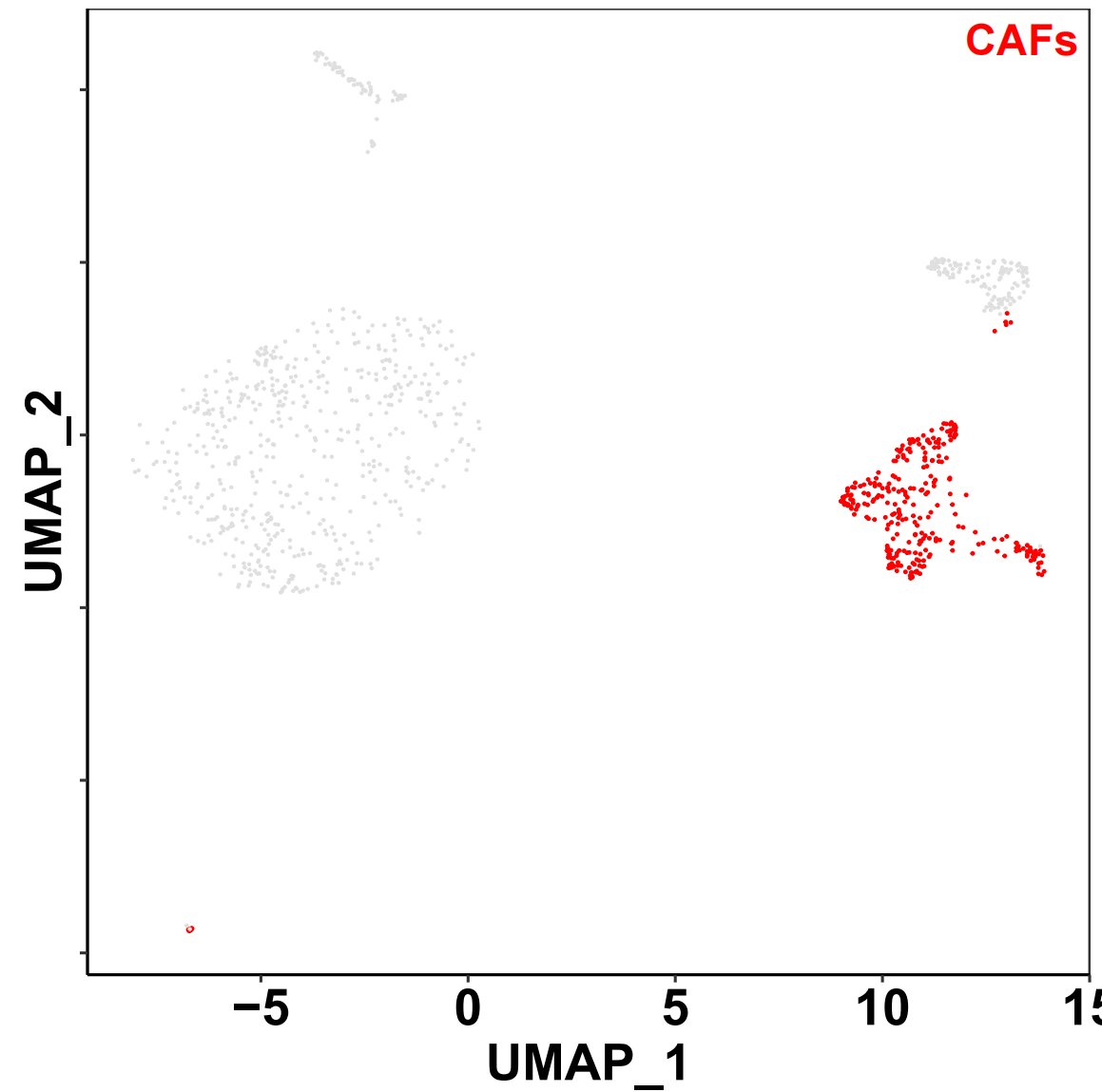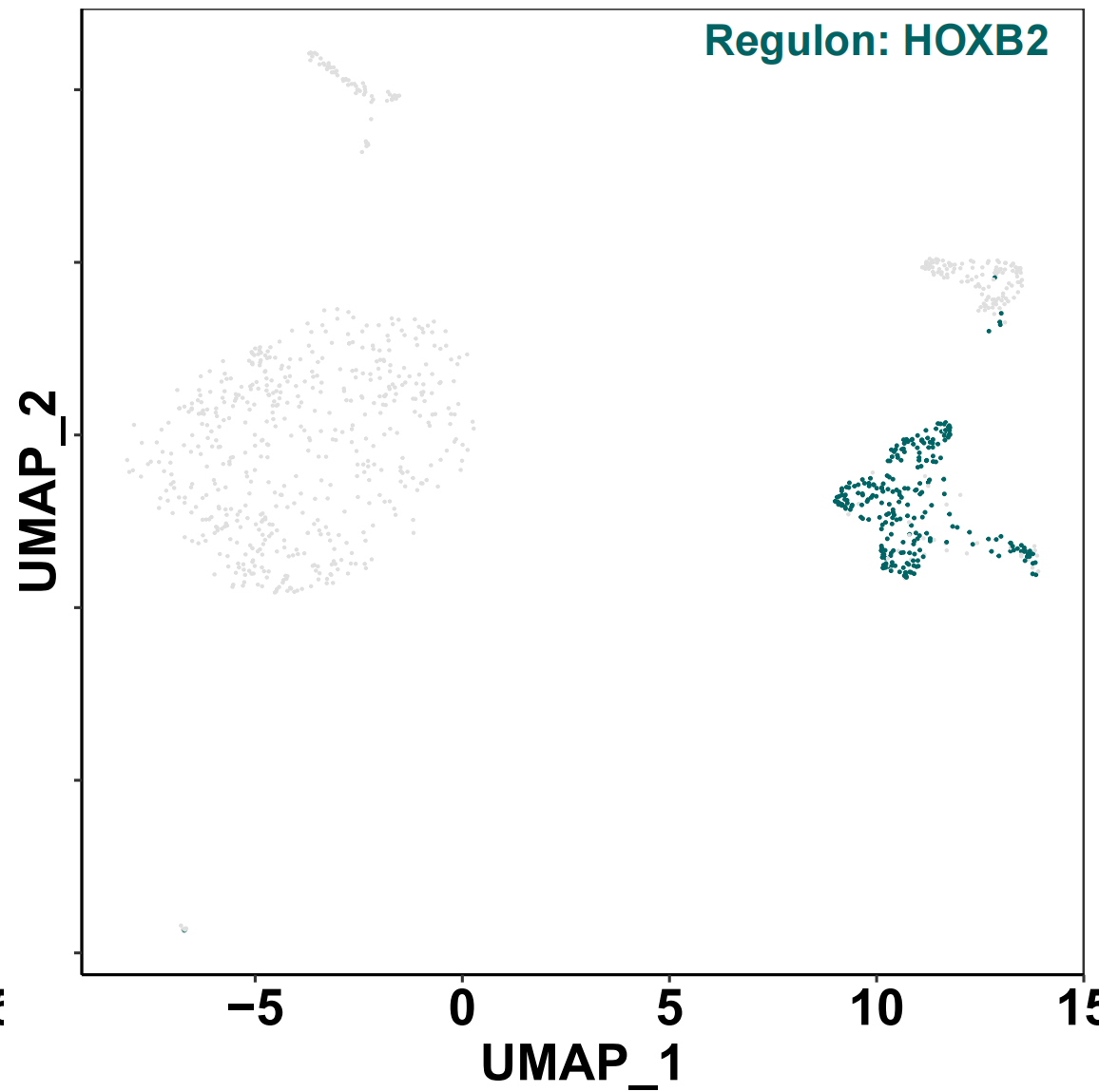

# OV

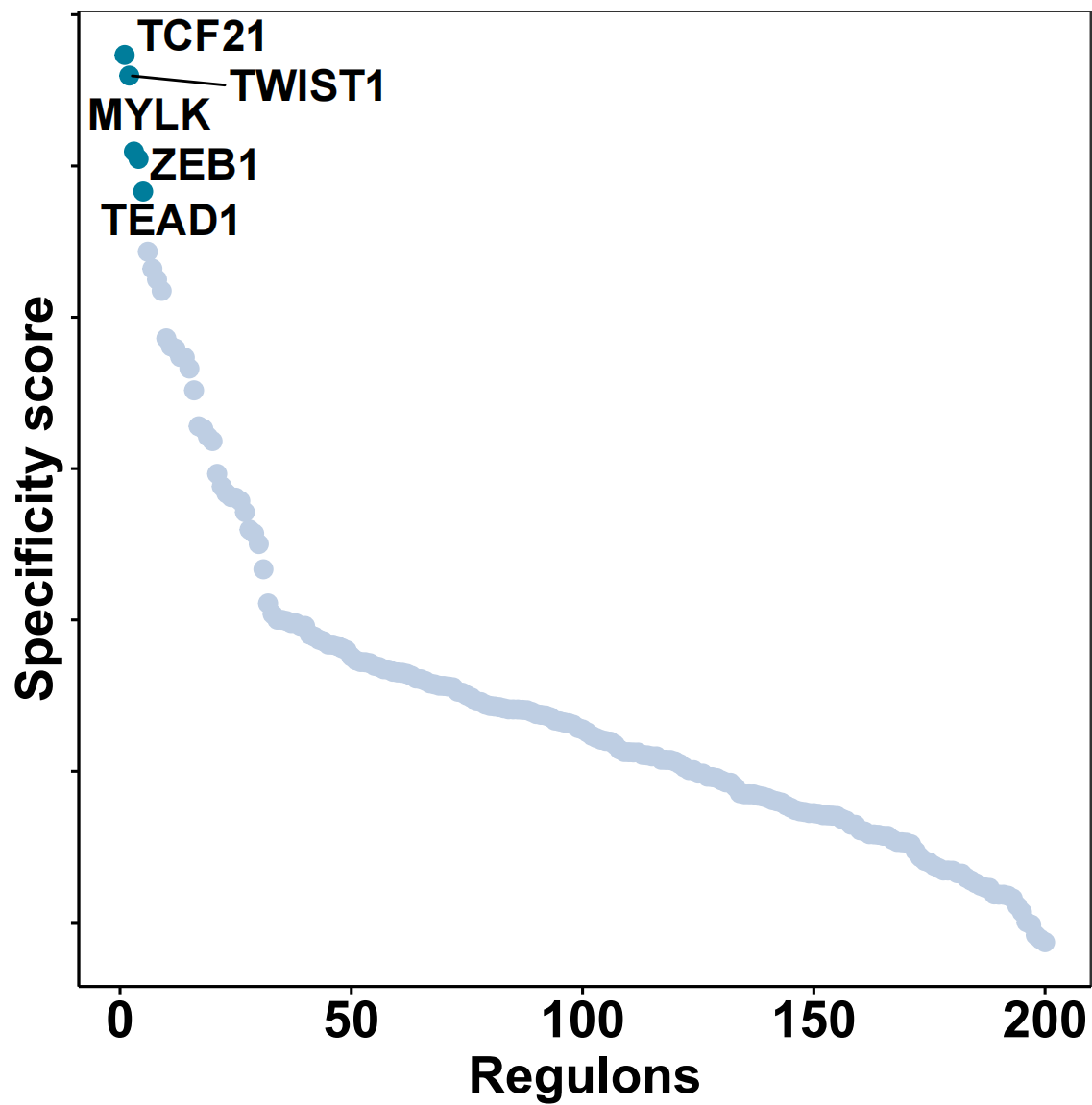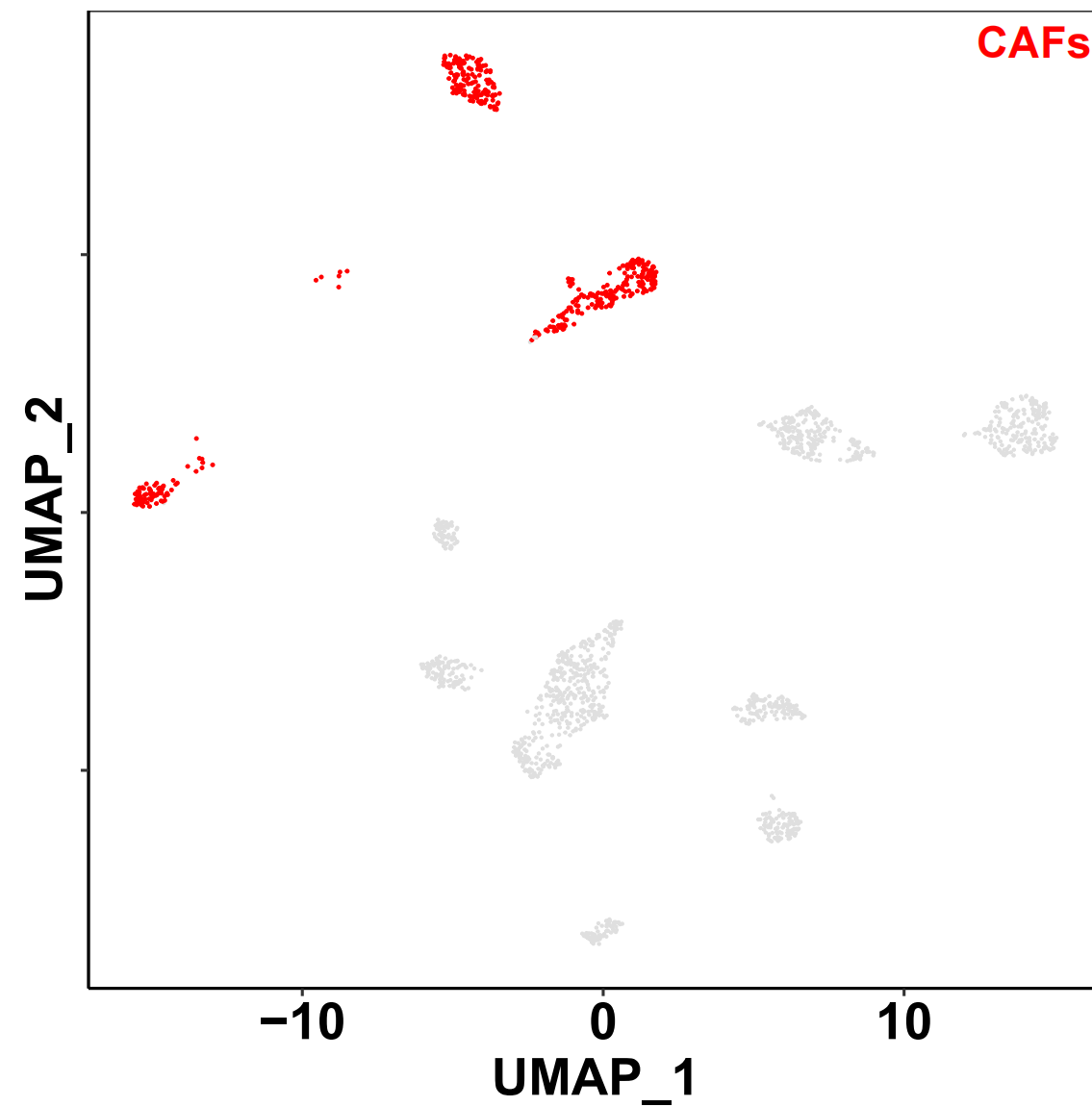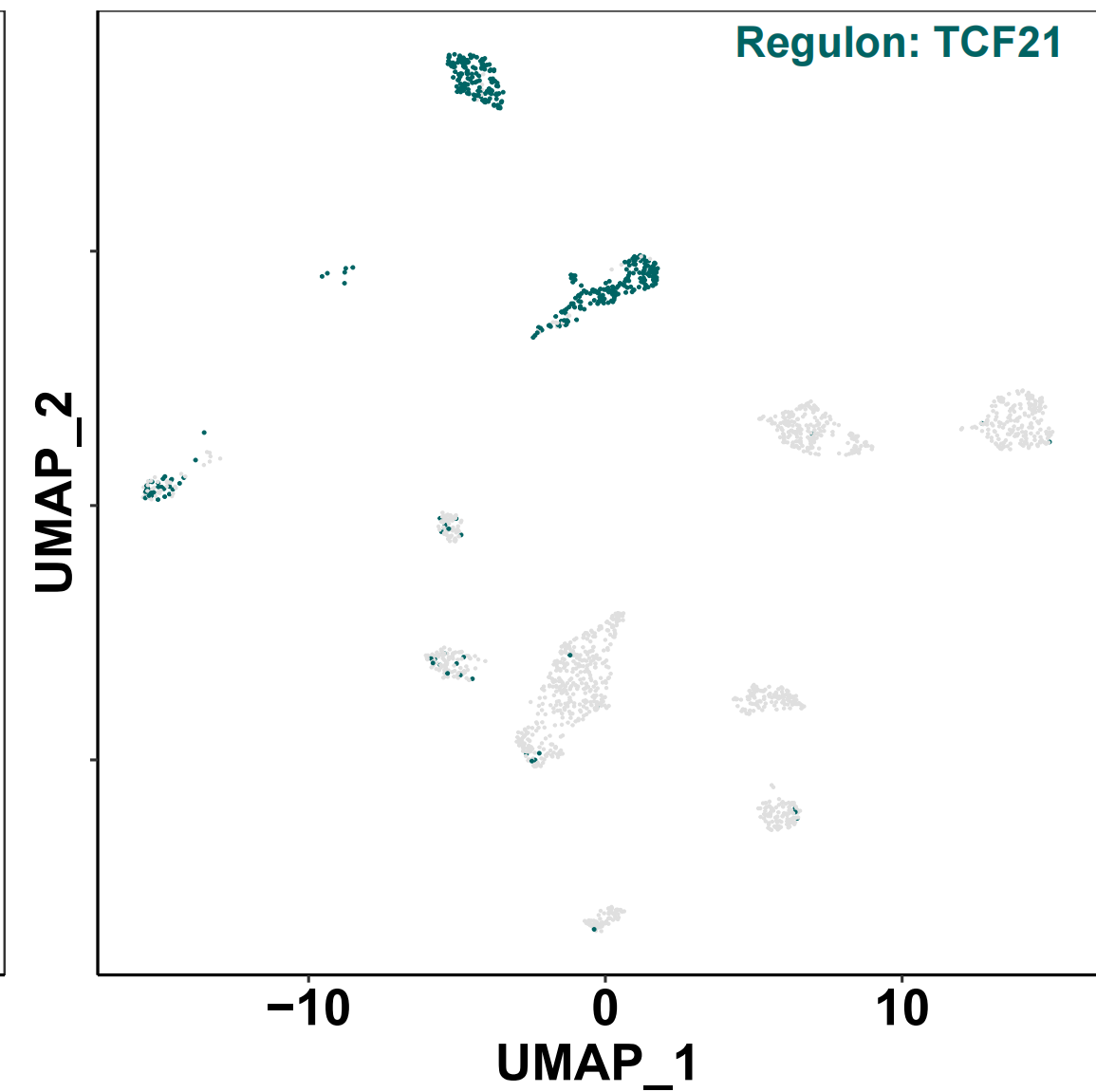

# PAAD

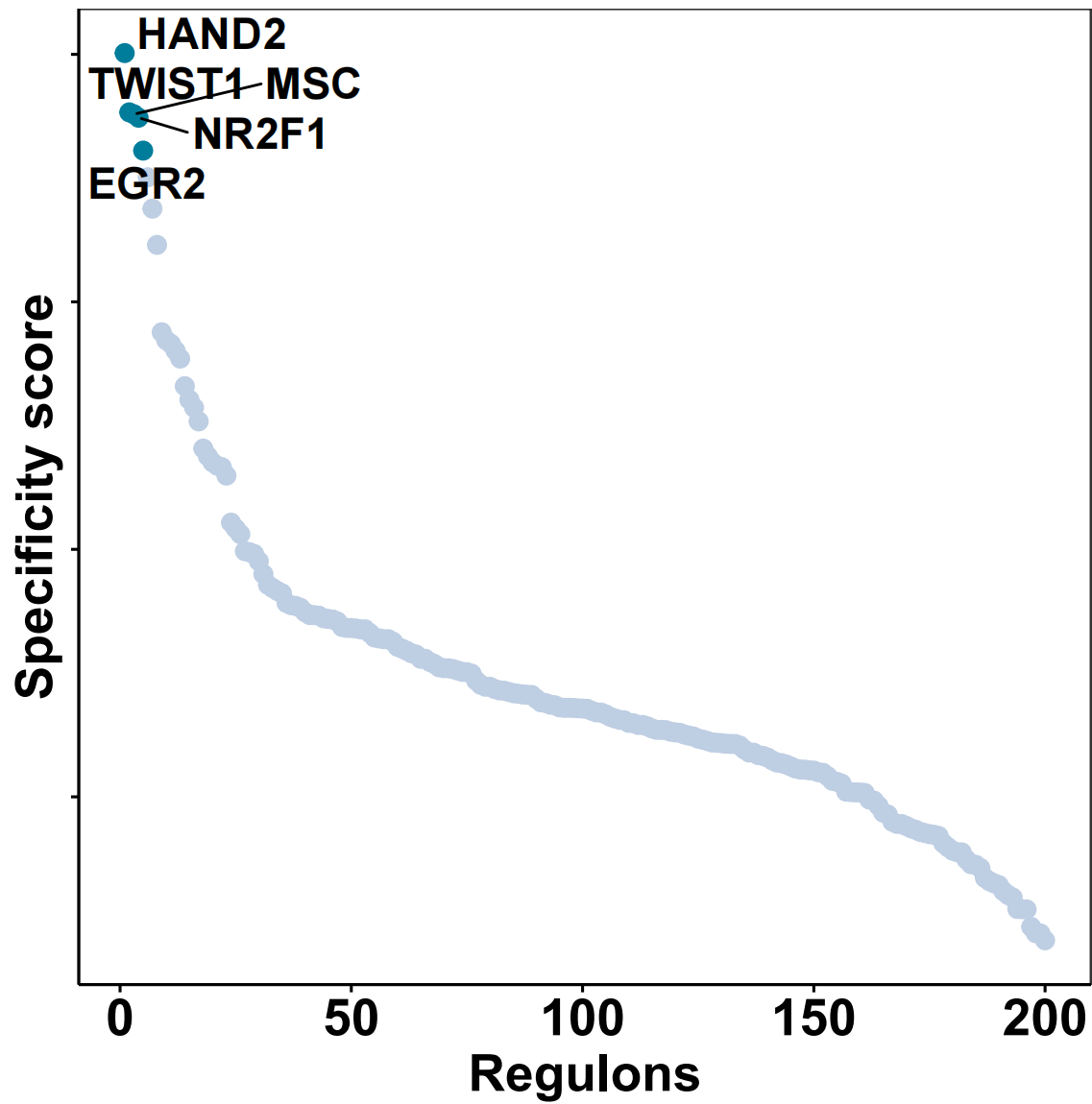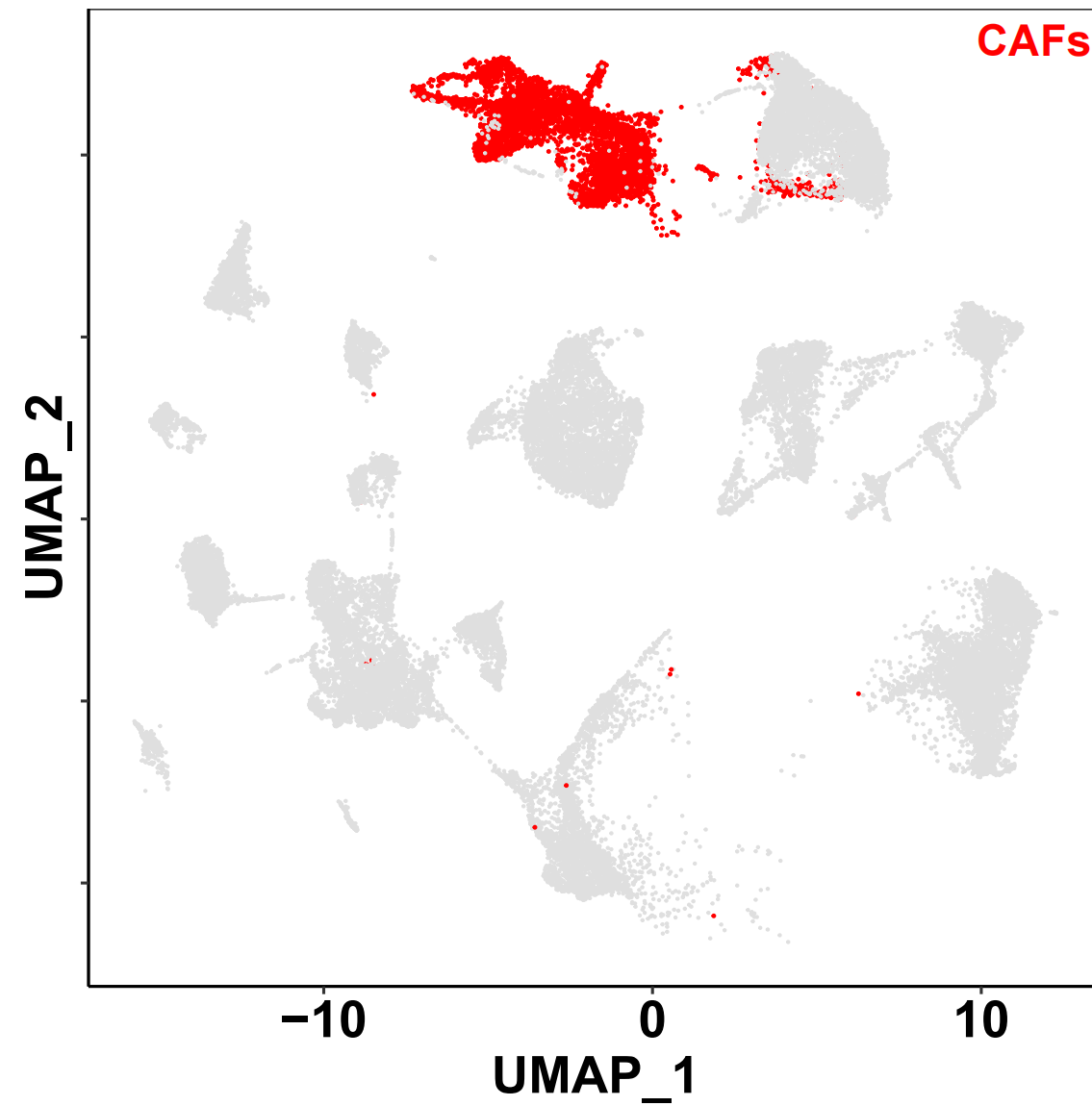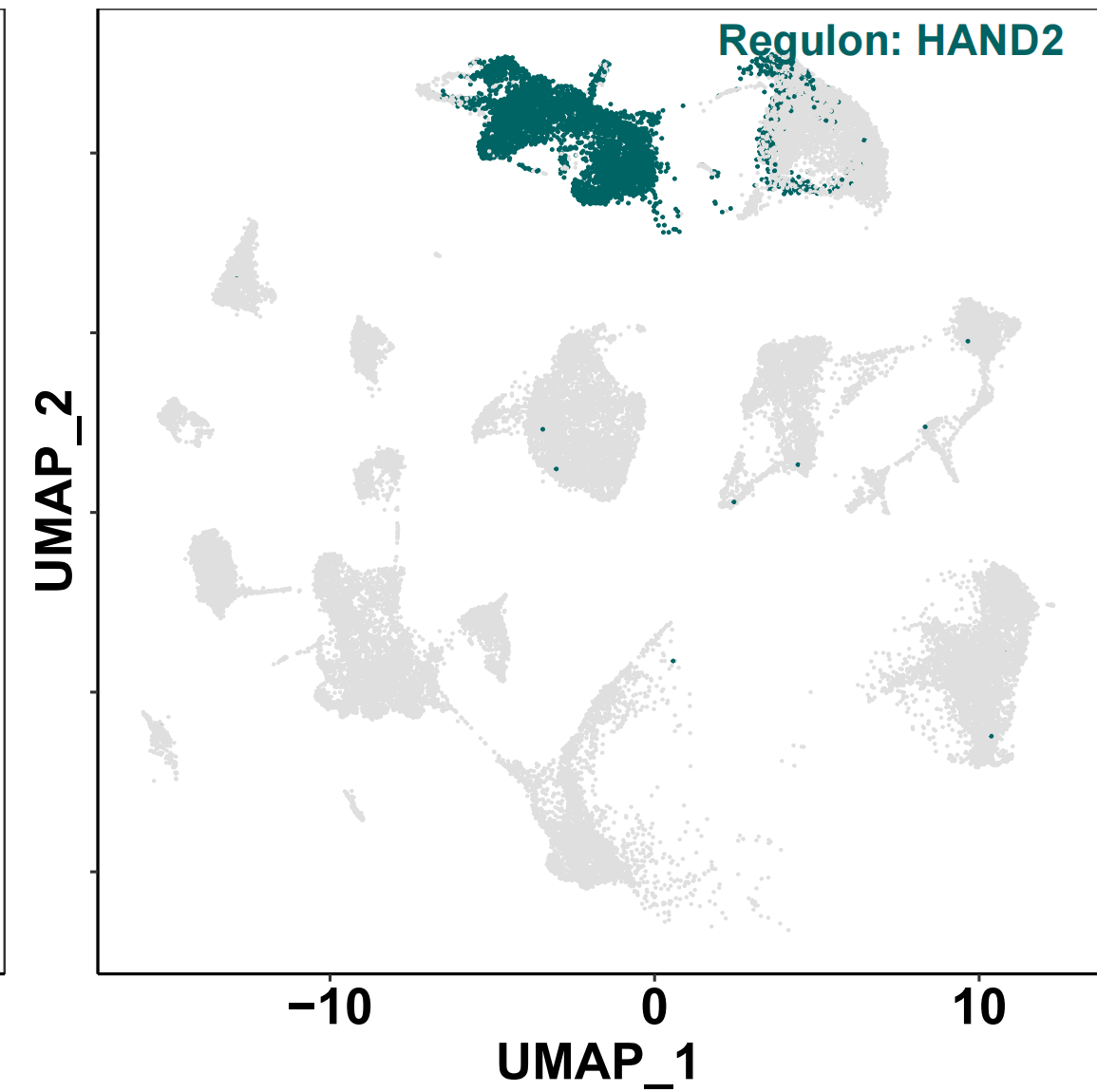

# STAD

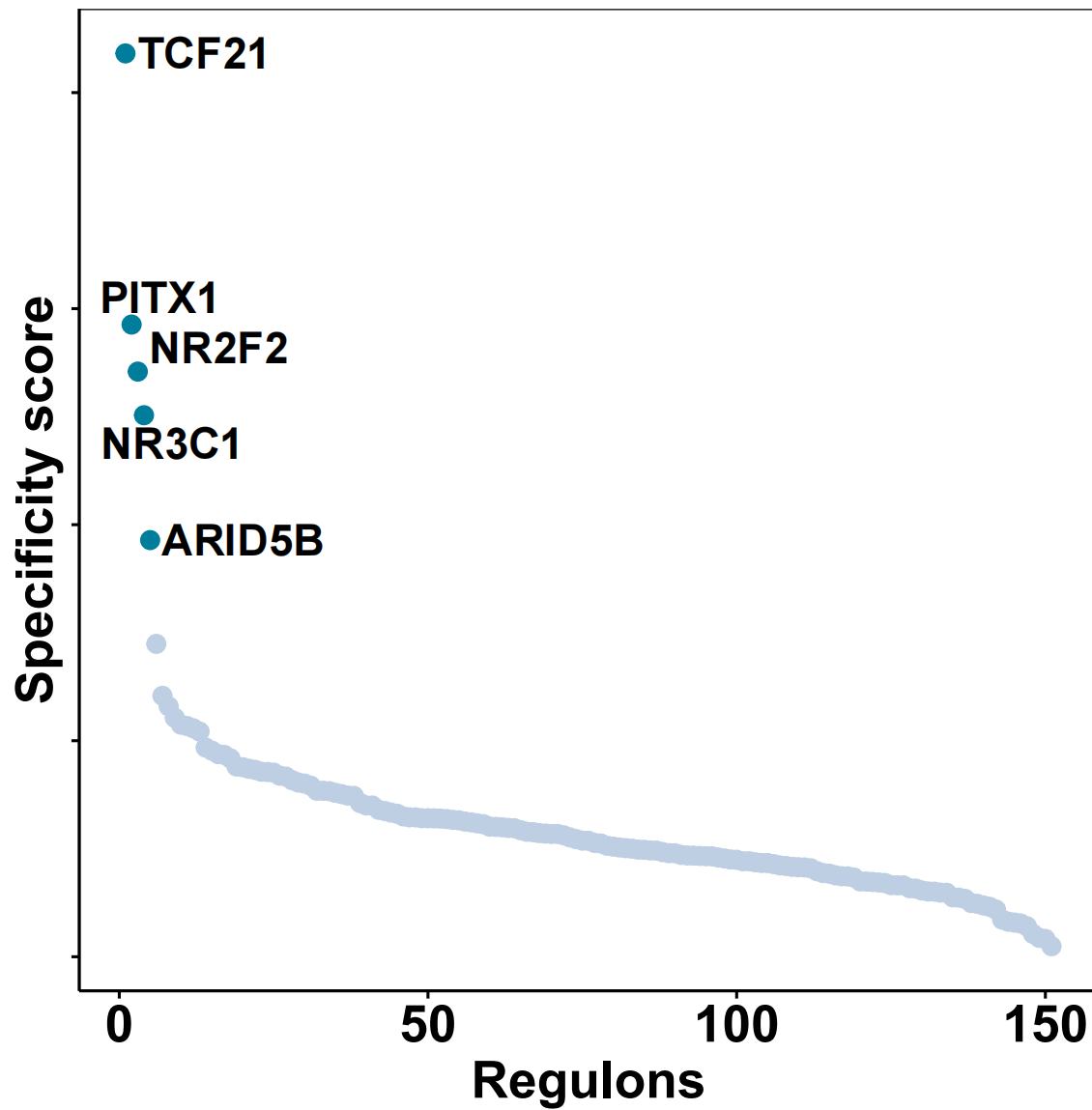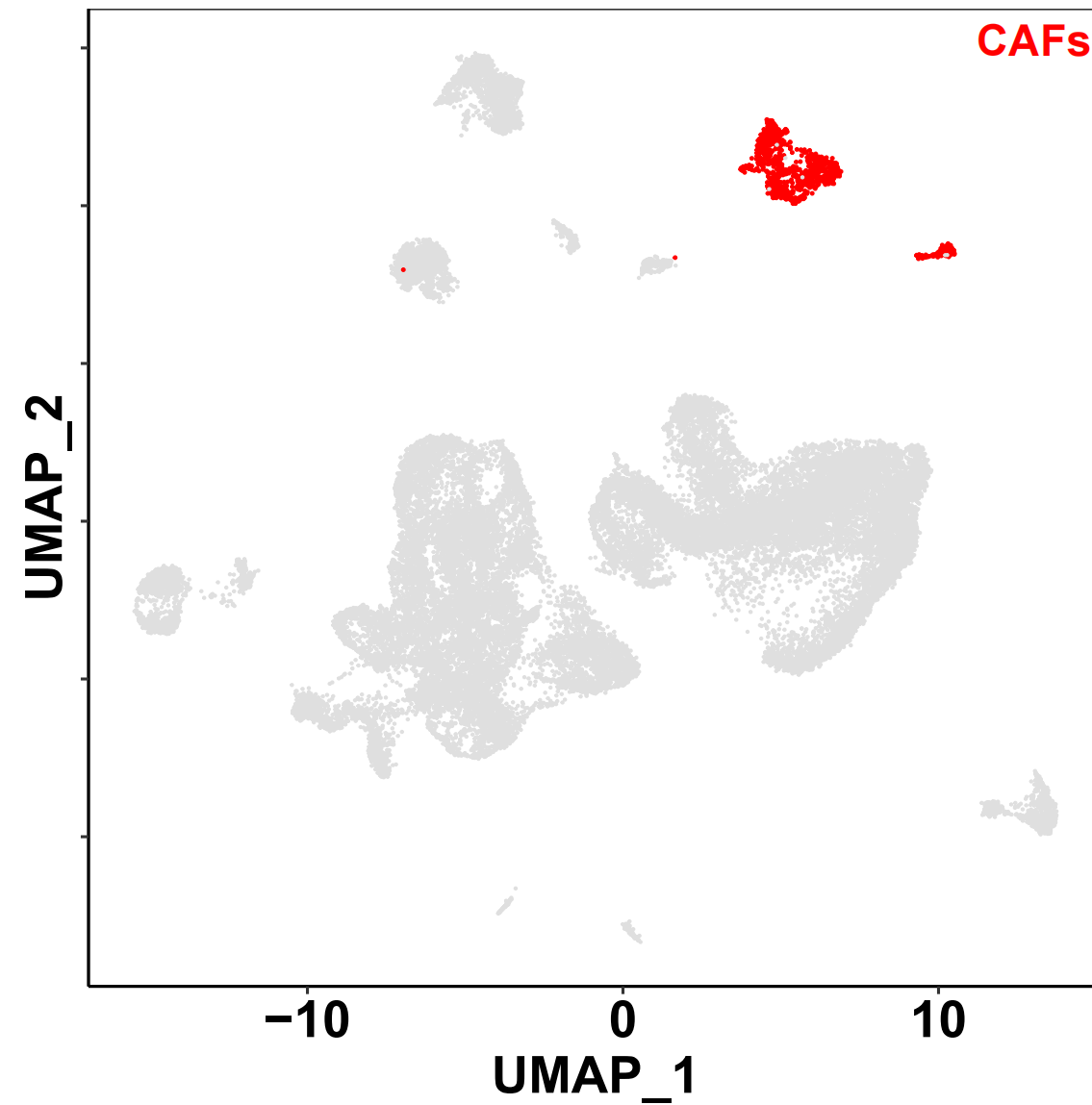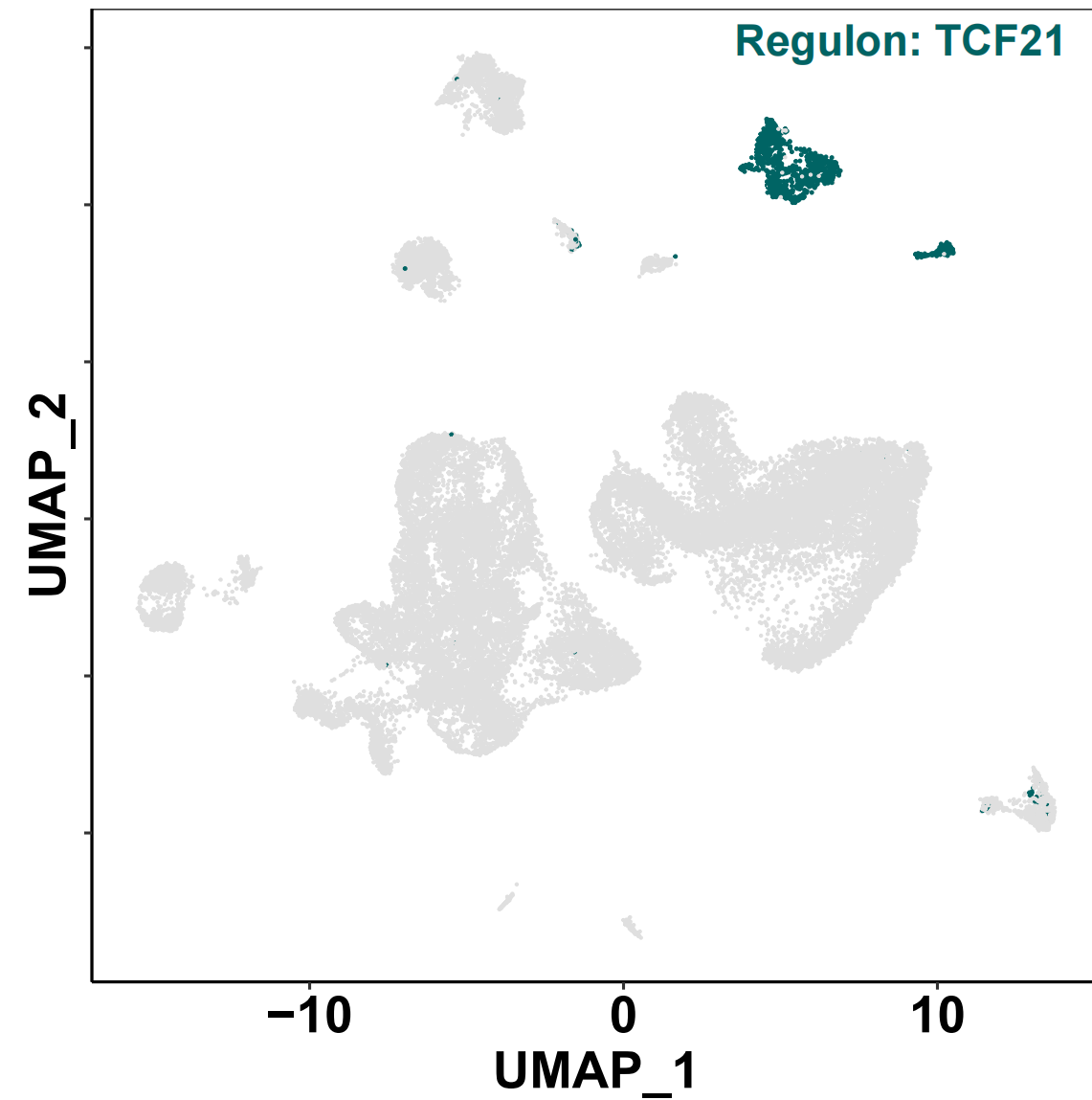

# SKCM

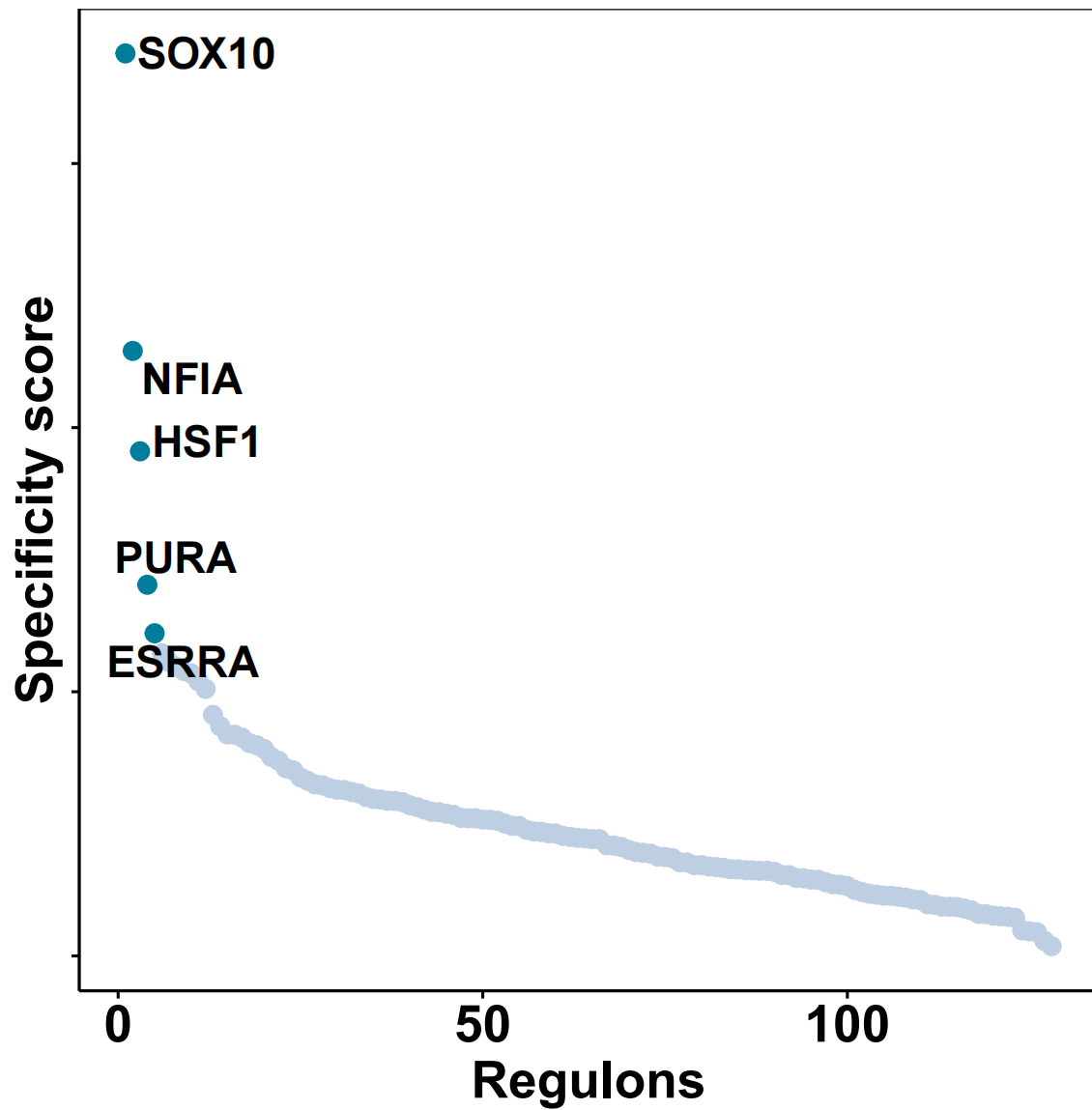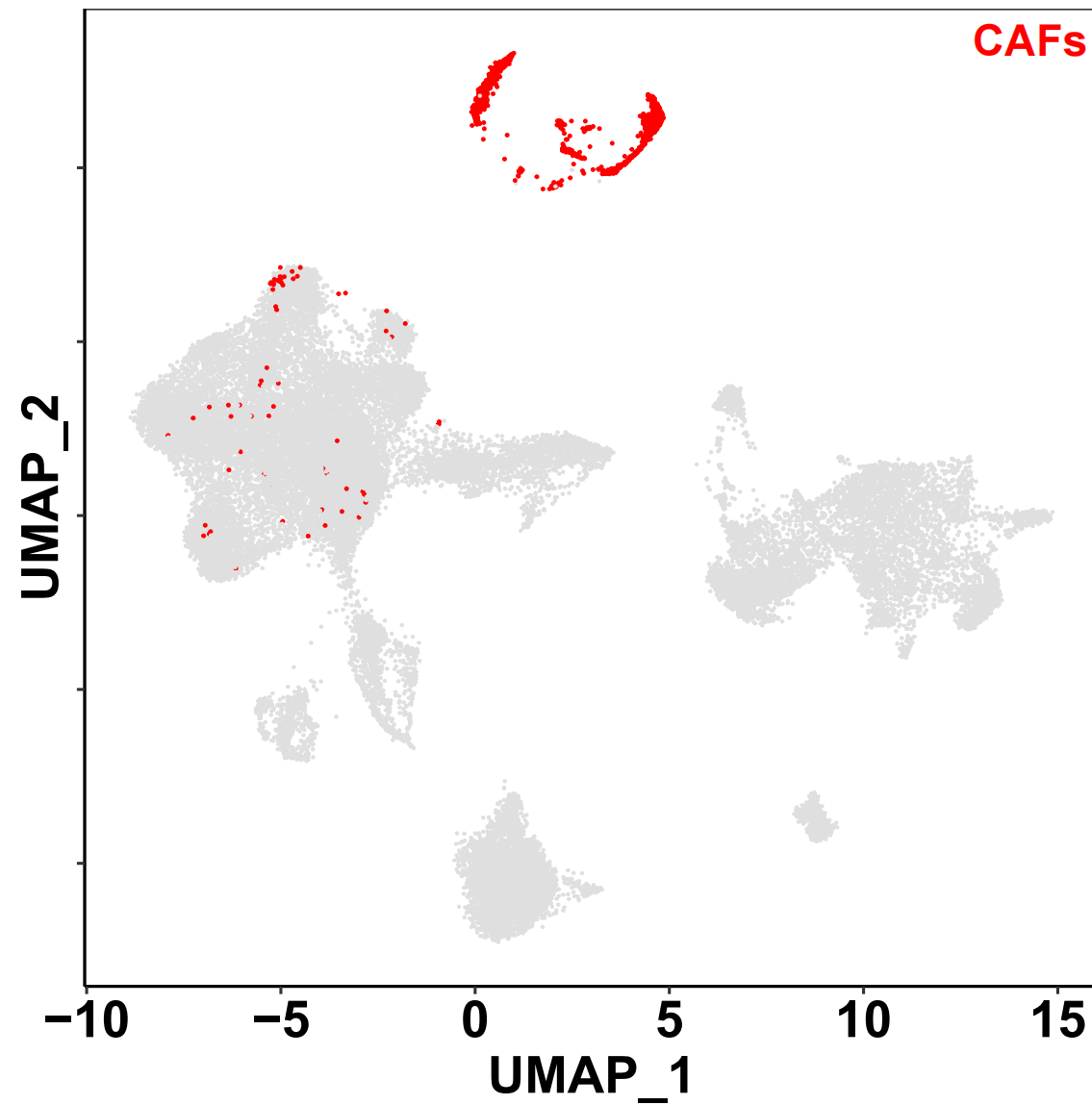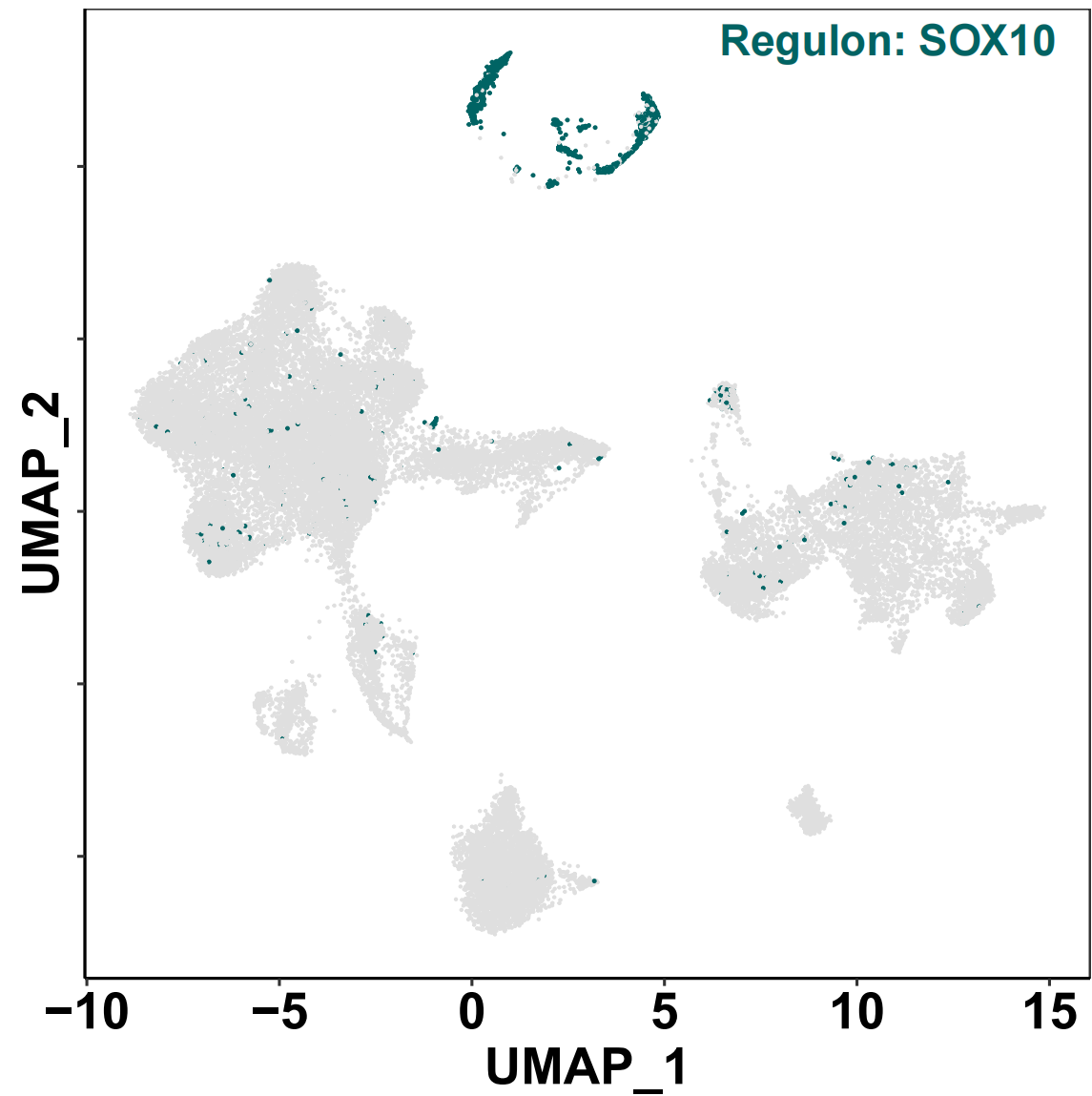

# KIRC

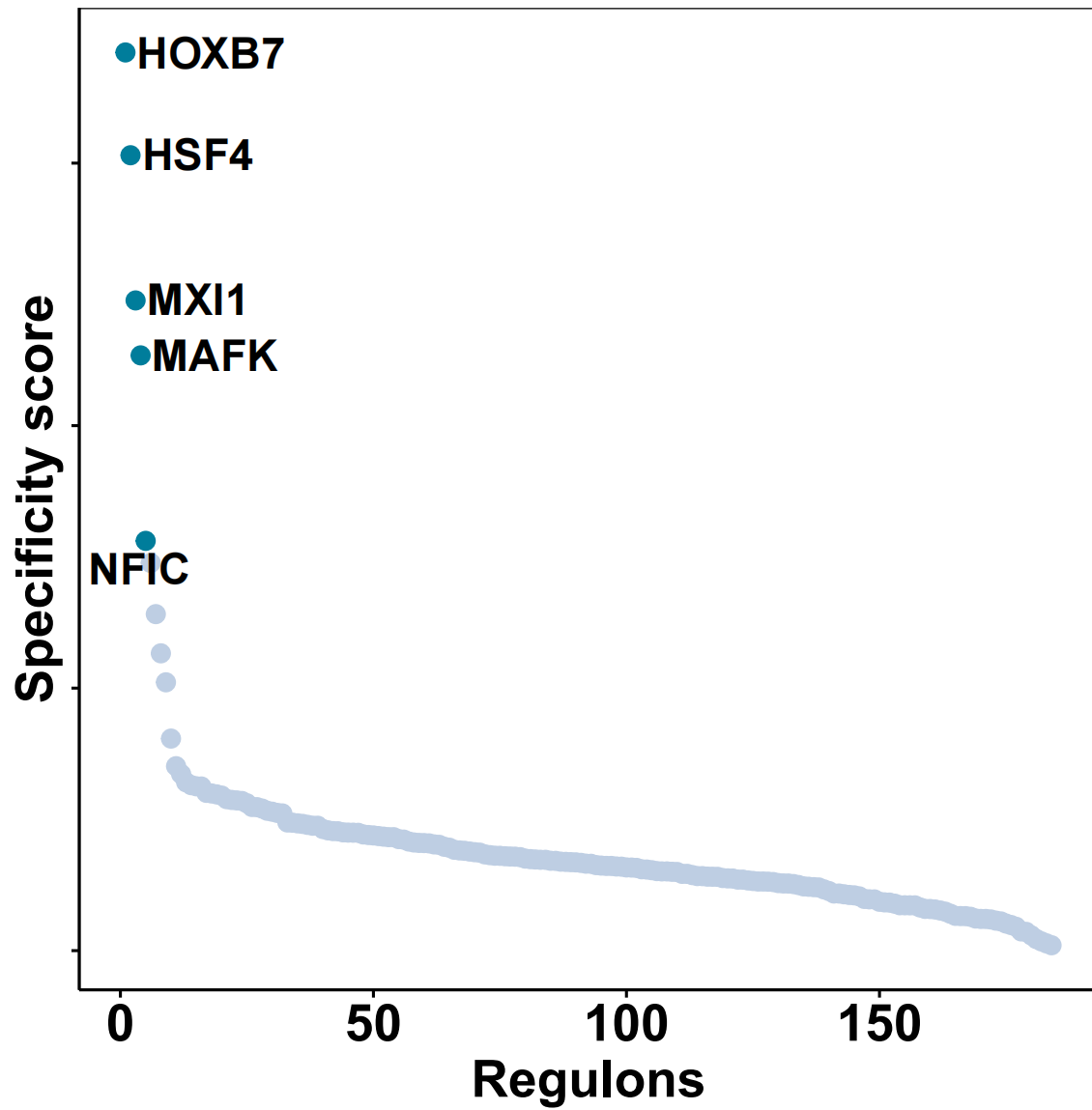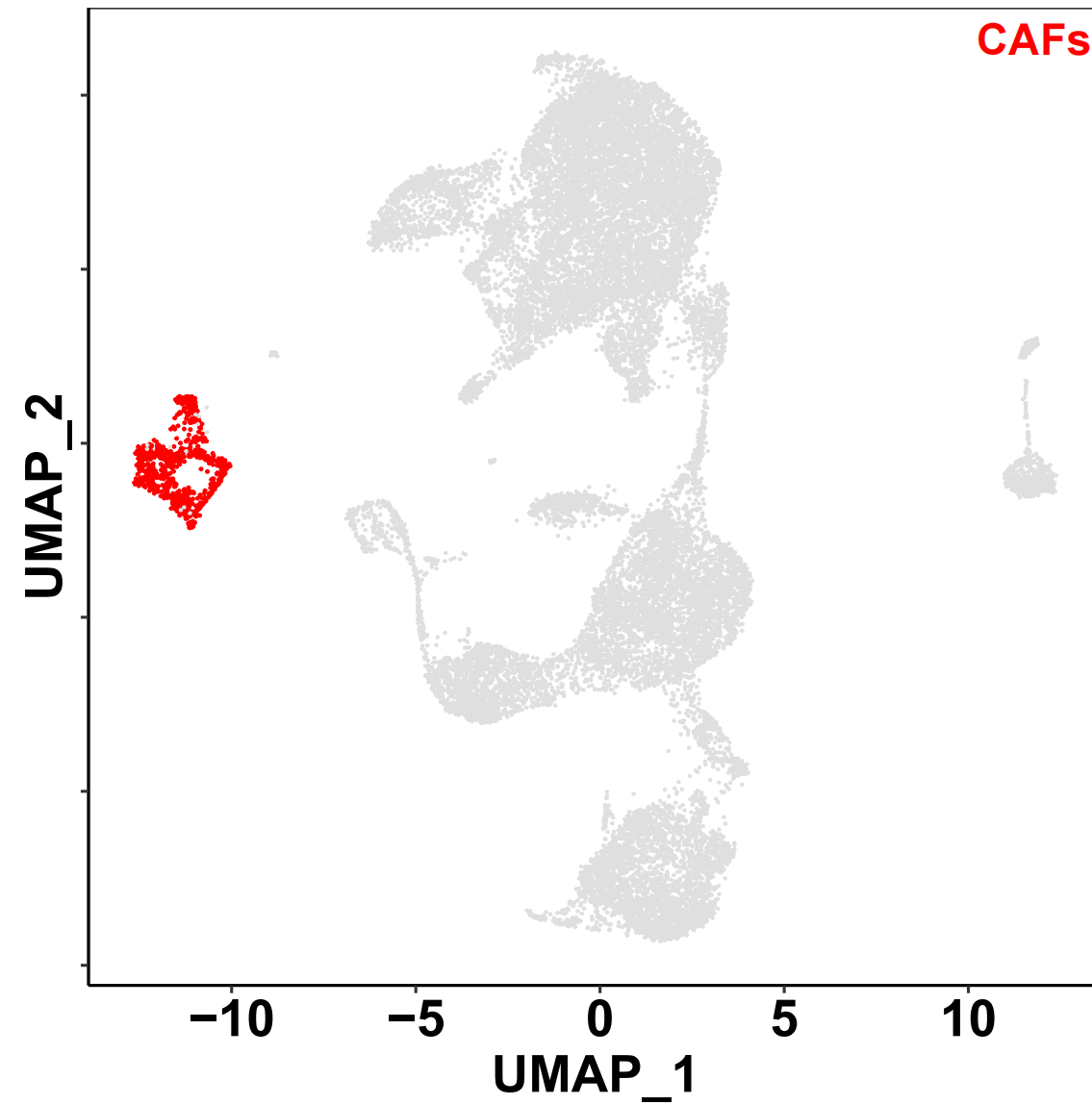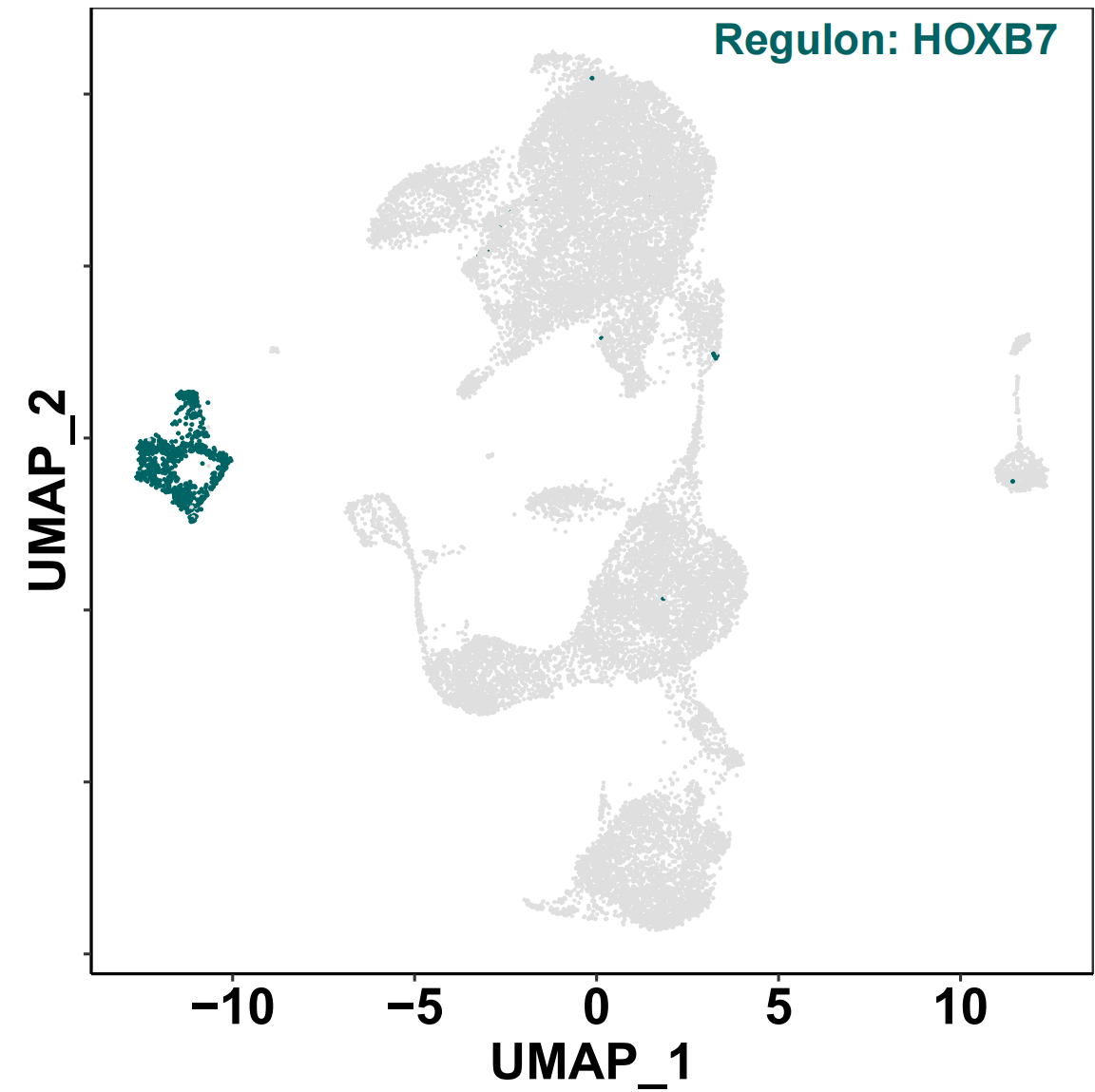

Supplement: Supplementary file 4 — Supporting Information [file CTM2-13-e1189-s007.pdf]

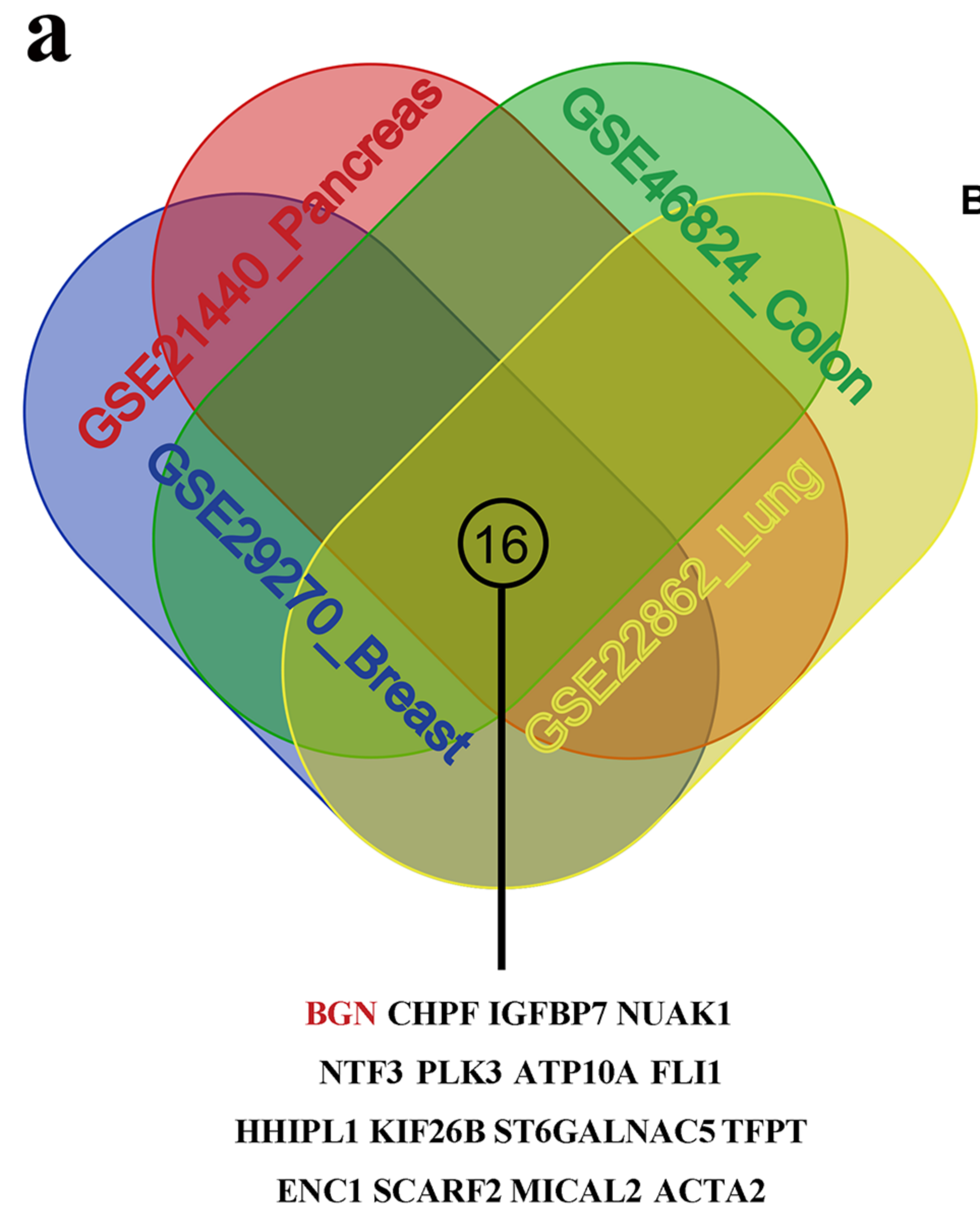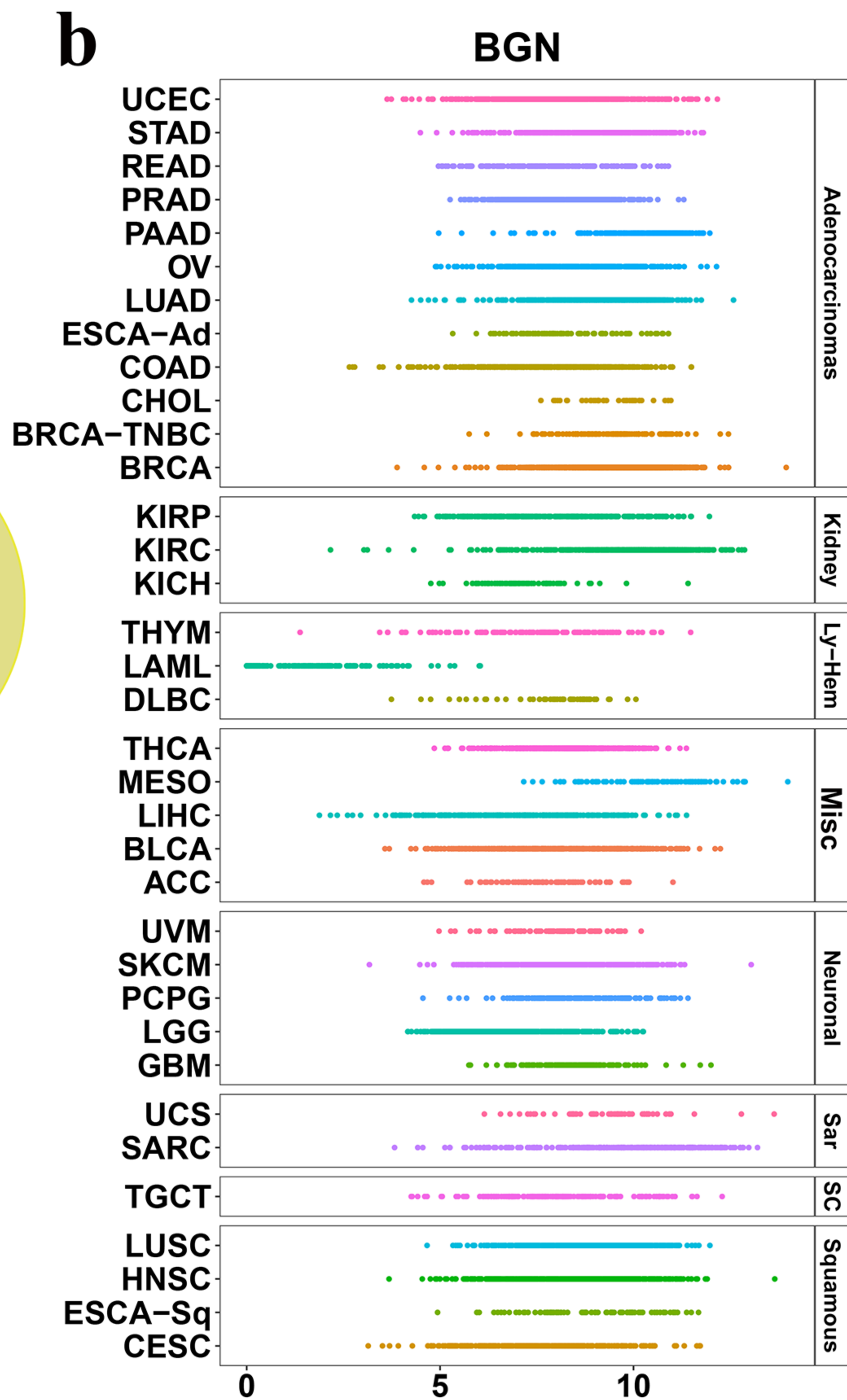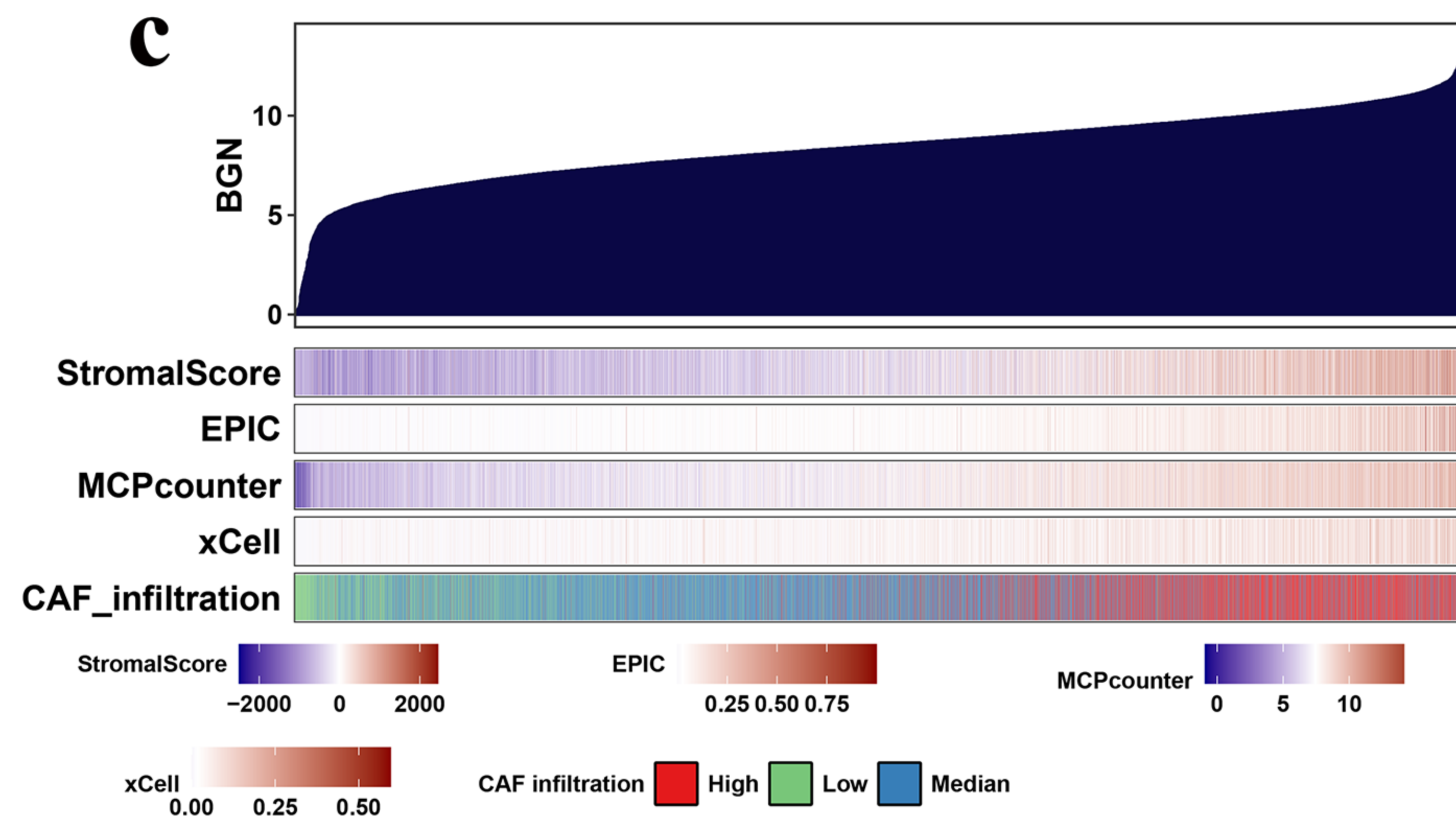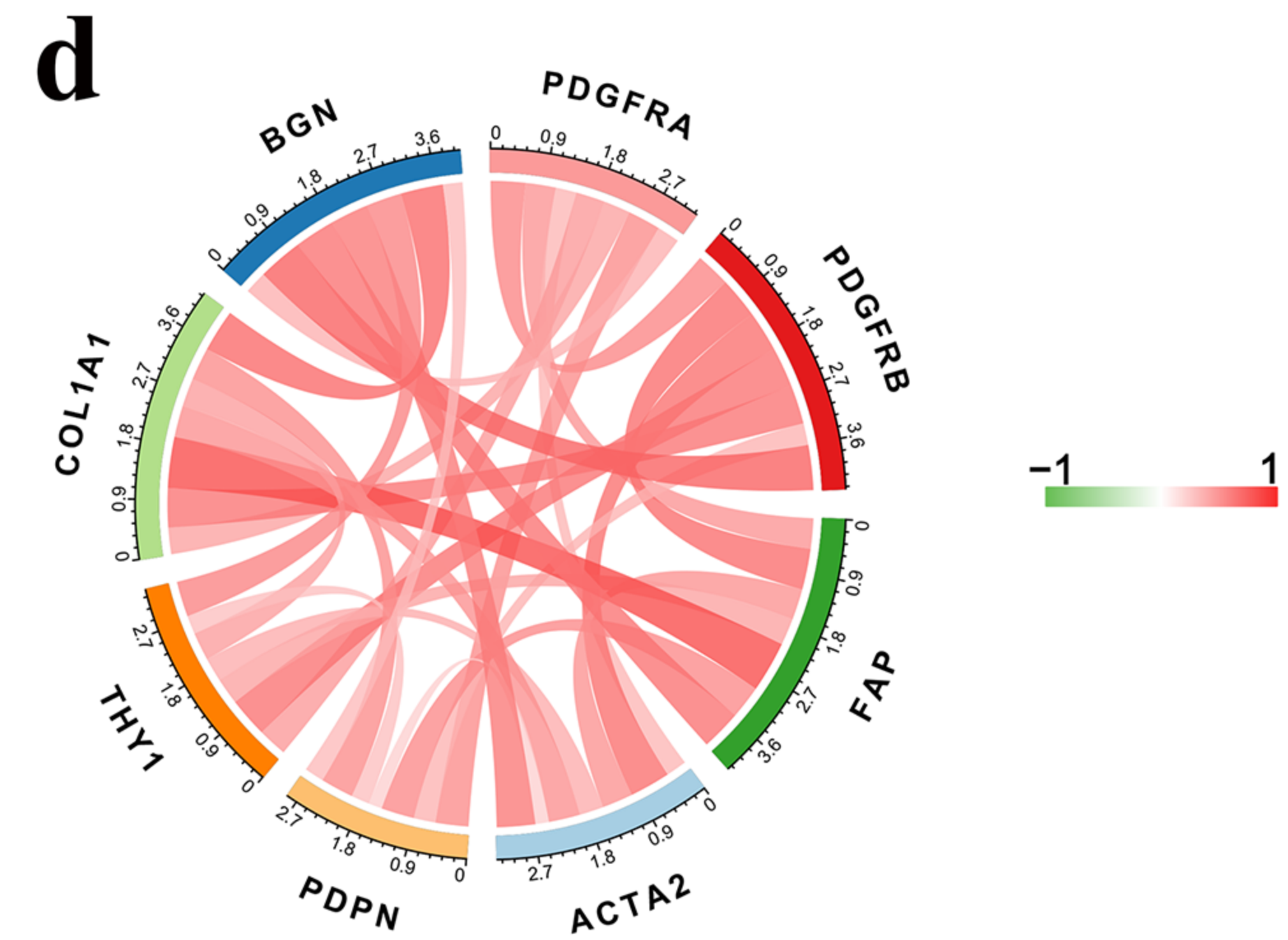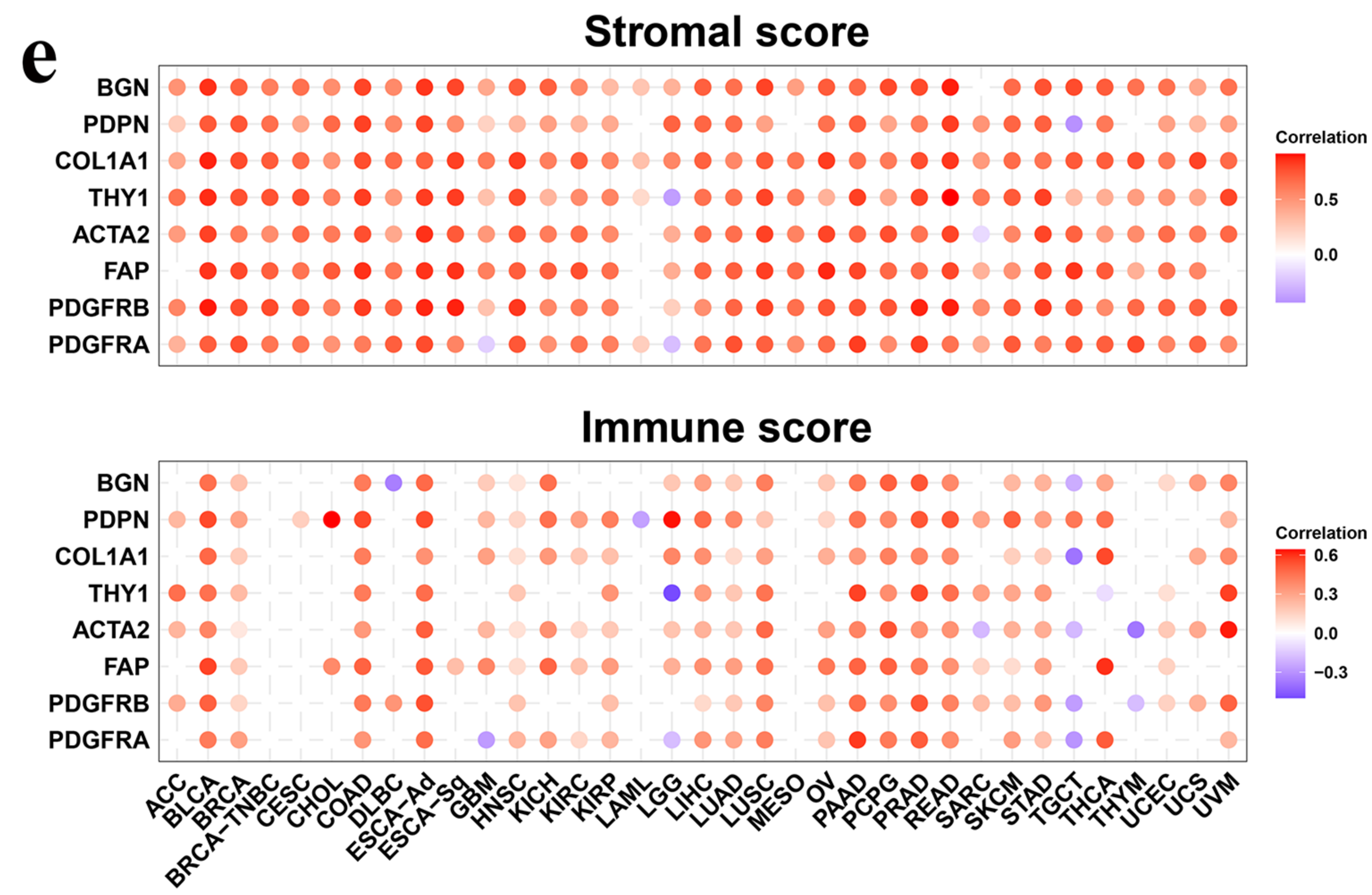

Supplement: Supplementary file 6 — Supporting Information [file CTM2-13-e1189-s014.pdf]

**a**

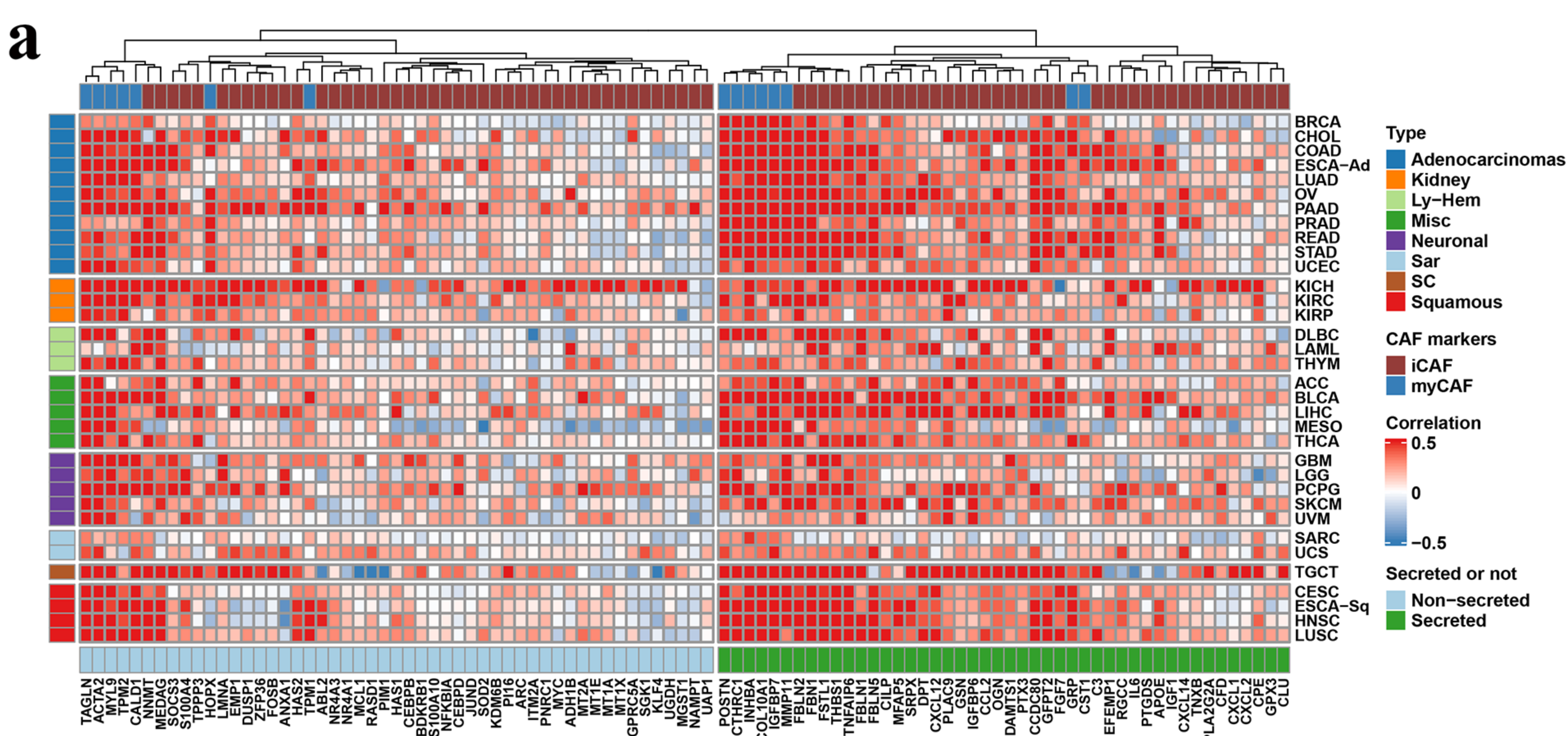**b**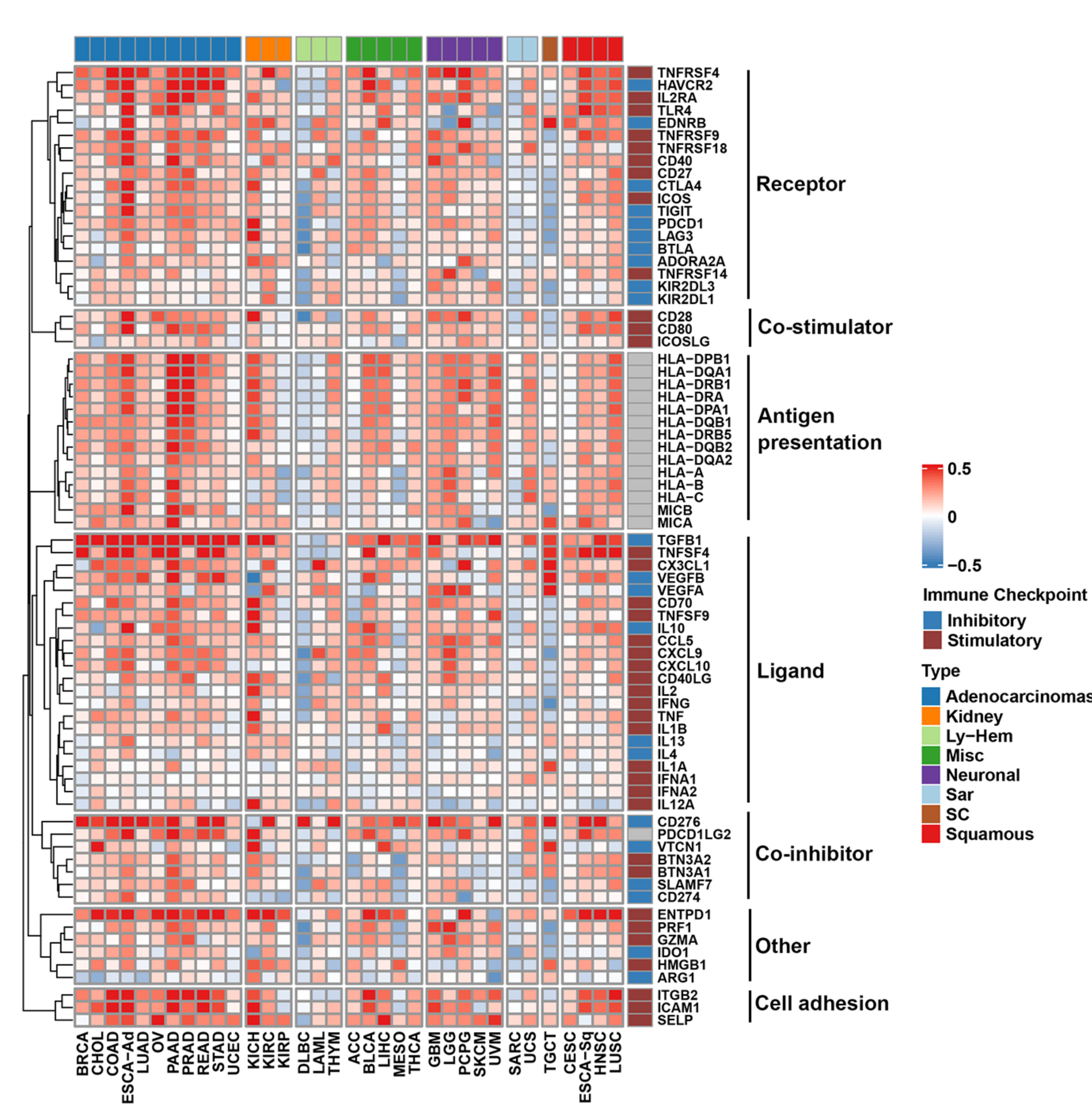

c

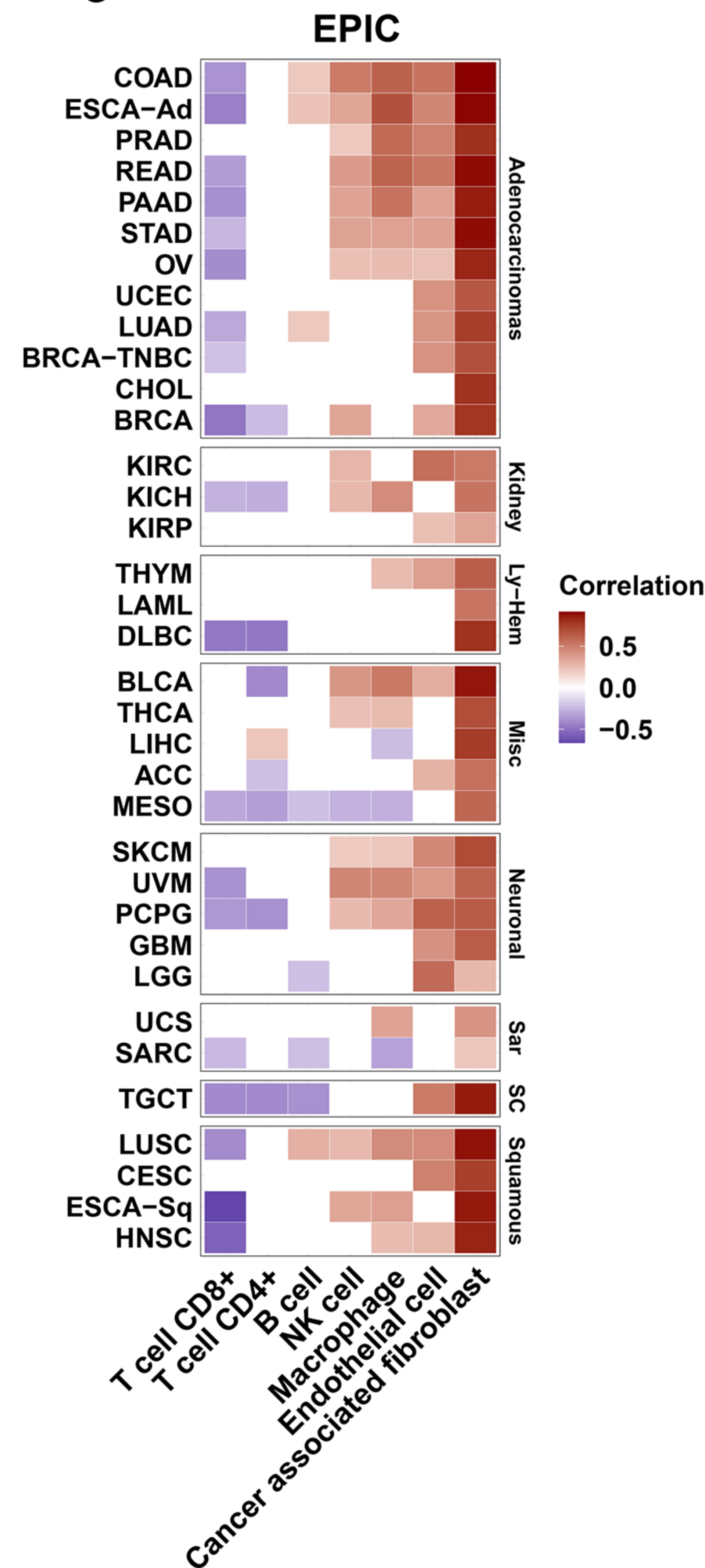

d

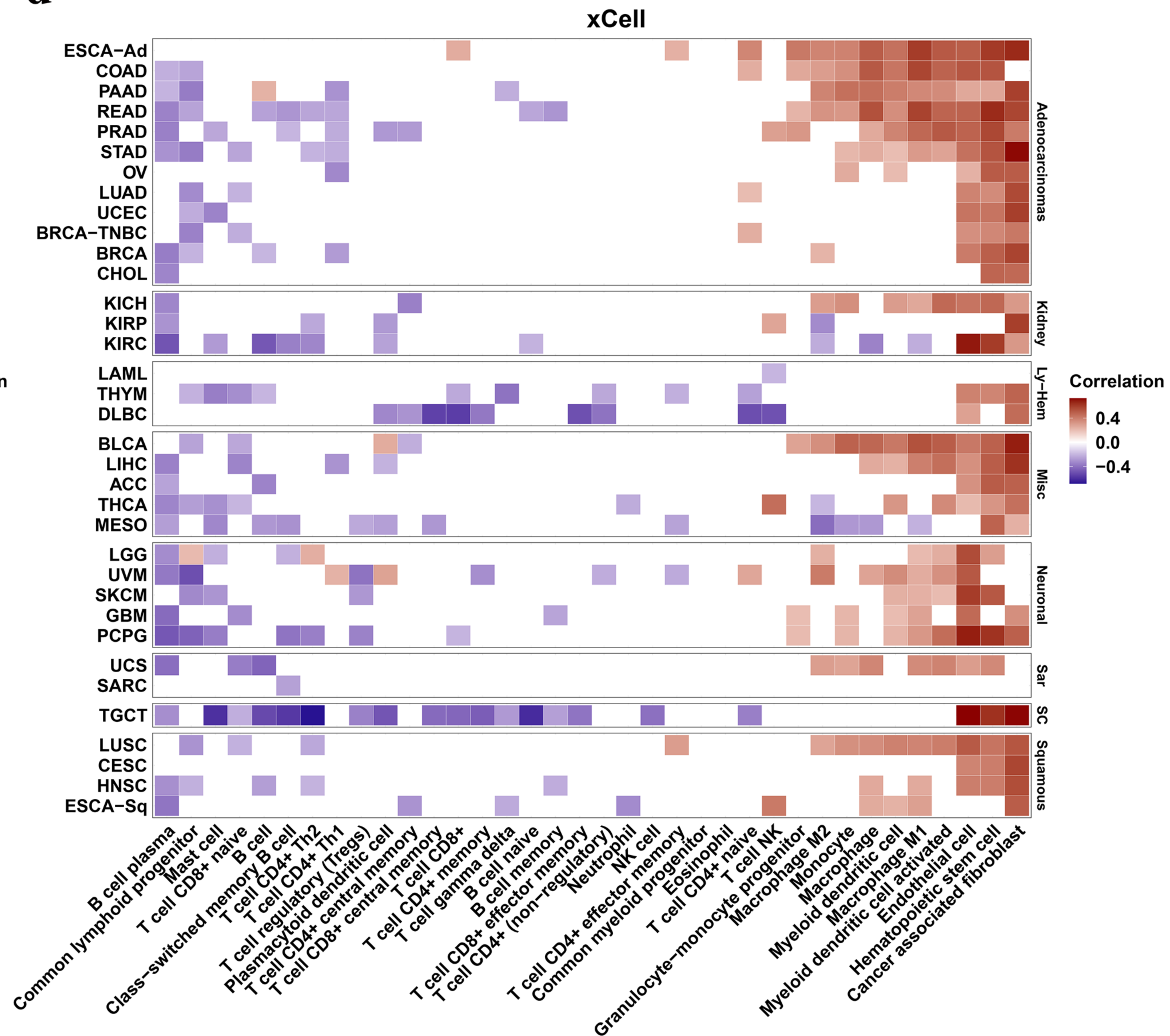

Supplement: Supplementary file 9 — Supporting Information [file CTM2-13-e1189-s009.pdf]
