# Supplementary material for: Dissecting the role of cancer‐associated fibroblast‐derived biglycan as a potential therapeutic target in immunotherapy resistance: A tumor bulk and single‐cell transcriptomic study
Source: Clin Transl Med. 2023 Feb 11;13(2):e1189. doi: 10.1002/ctm2.1189 (PMC9920016; doi:10.1002/ctm2.1189)
Supplement: Supplementary file 5 — Supporting Information [file CTM2-13-e1189-s016.pdf]

BLCA

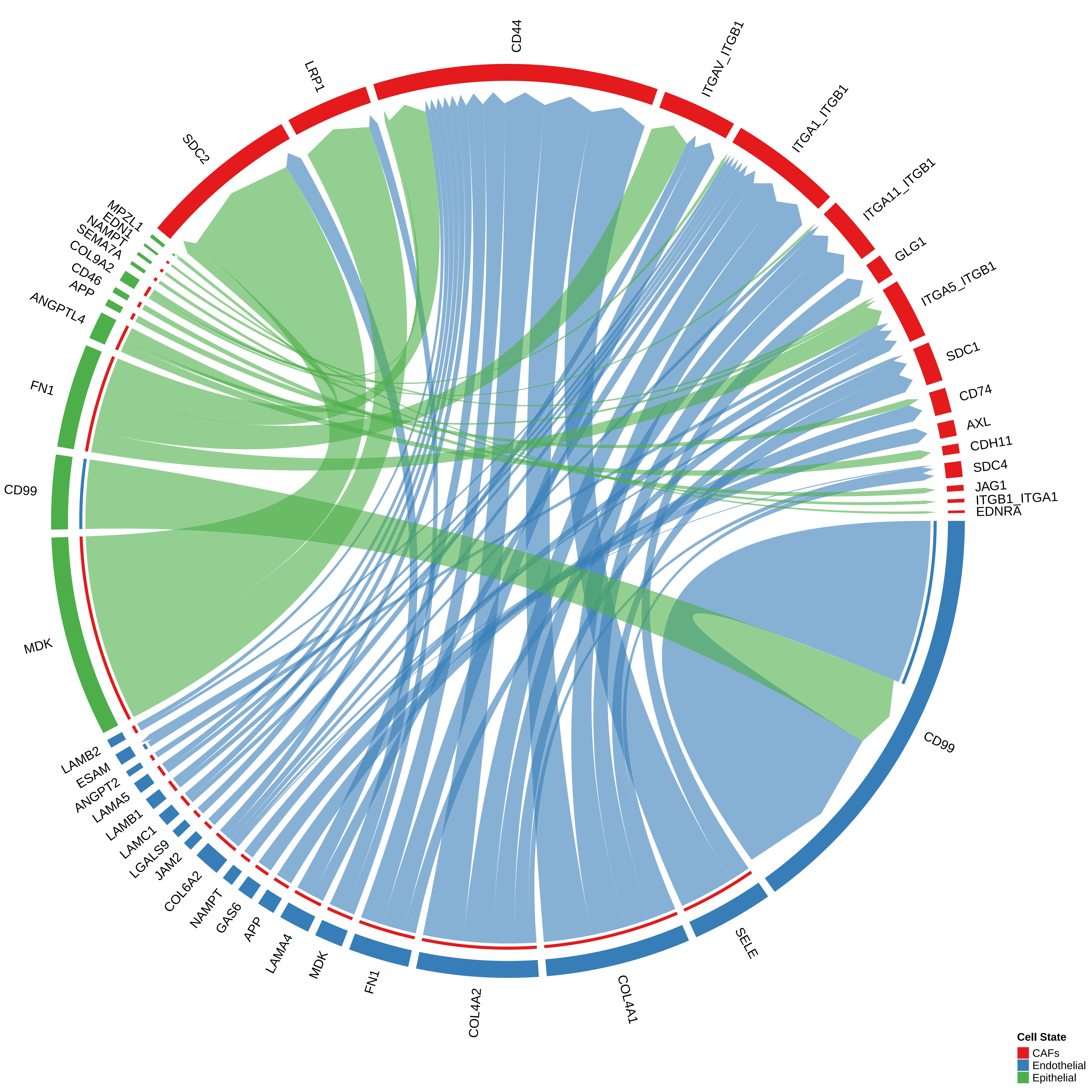

CD44
ITGAV_ITGB1
ITGA1_ITGB1
ITGA11_ITGB1
GLG1
ITGA5_ITGB1
SDC1
CD74
AXL
CDH11
SDC4
JAG1
ITGB1_ITGA1
EDNRA
CD99
SELE
COL4A1
COL4A2
FN1
MDK
LAMA4
APP
GAS6
NAMPT
COL6A2
JAM2
LGALS9
LAMC1
LAMB1
LAMA5
ANGPT2
ESAM
LAMB2
MDK
CD99
FN1
ANGPTL4
APP
CD46
COL9A2
SEMA7A
NAMPT
EDN1
MPZL1
SDC2
LRP1
Cell State
CAFs
Endothelial
Epithelial

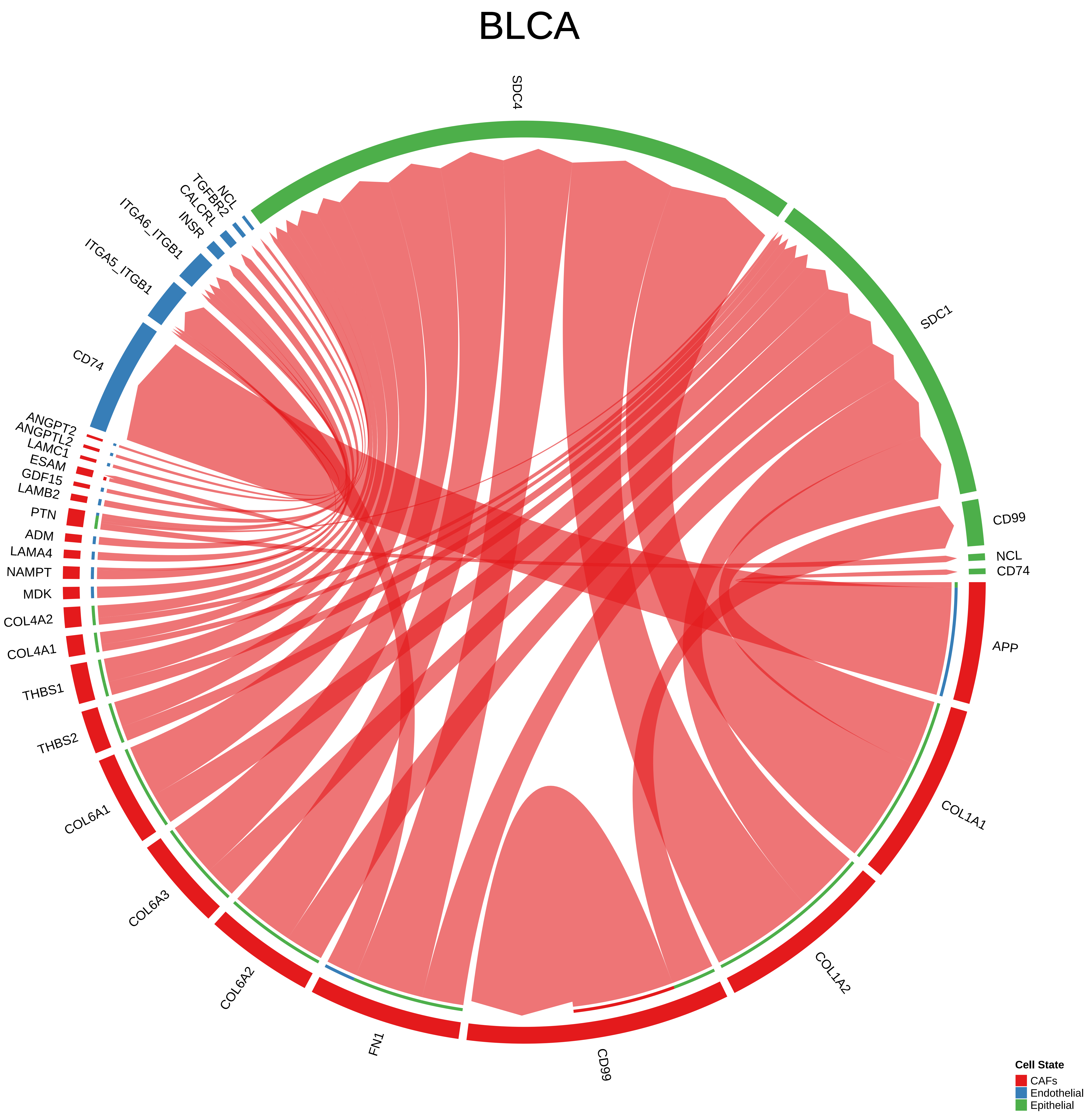

BLCA
SDC4
SDC1
CD99
NCL
CD74
APP
COL1A1
COL1A2
CD99
FN1
COL6A2
COL6A3
COL6A1
THBS2
THBS1
COL4A1
COL4A2
MDK
NAMPT
LAMA4
ADM
PTN
LAMB2
GDF15
ESAM
LAMC1
ANGPTL2
ANGPT2
CD74
ITGA5_ITGB1
ITGA6_ITGB1
INSR
CALCRL
TGFBR2
NCL
Cell State
CAFs
Endothelial
Epithelial

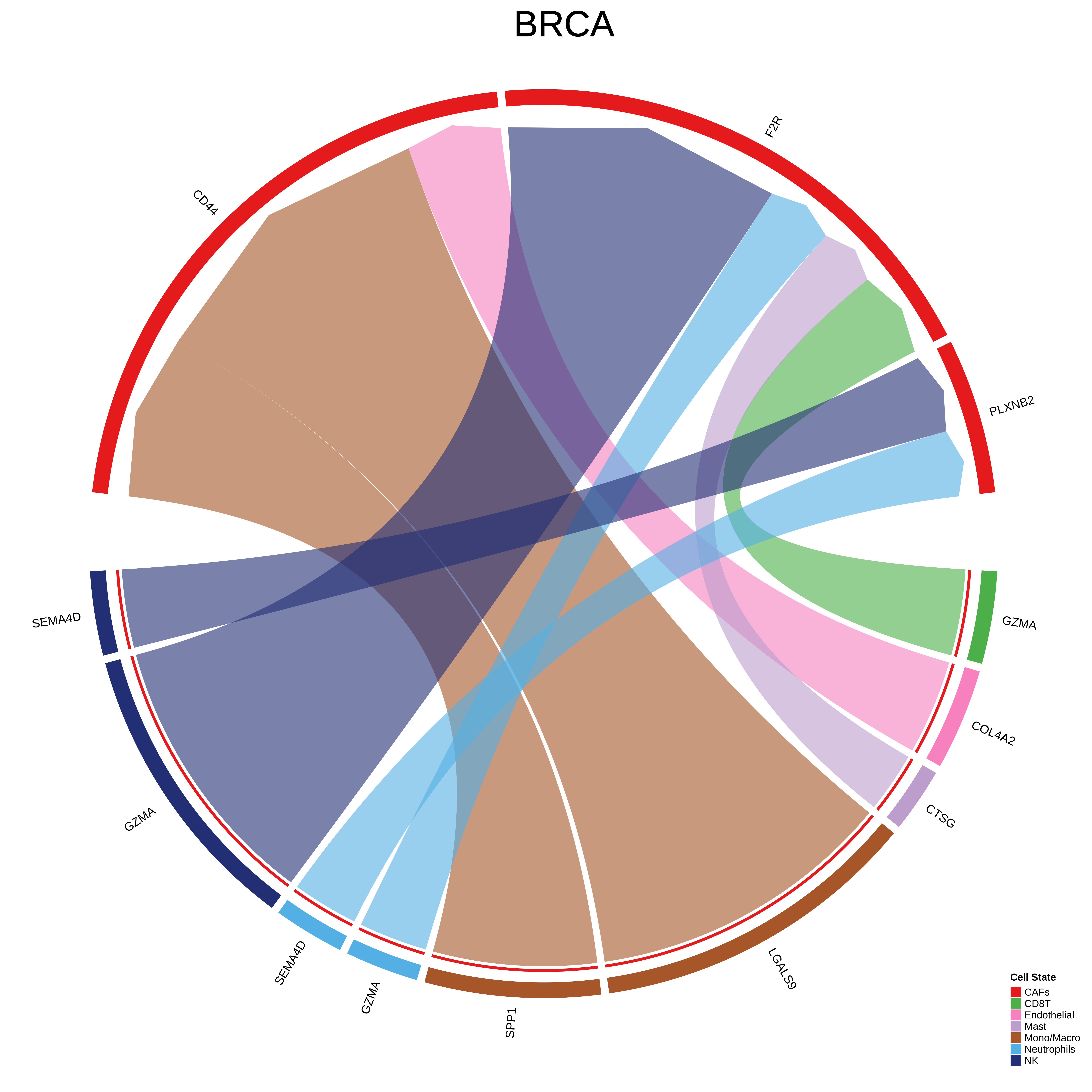

BRCA
CD44
F2R
PLXNB2
GZMA
COL4A2
CTSG
LGALS9
SPP1
GZMA
SEMA4D
GZMA
SEMA4D
Cell State
CAFs
CD8T
Endothelial
Mast
Mono/Macro
Neutrophils
NK

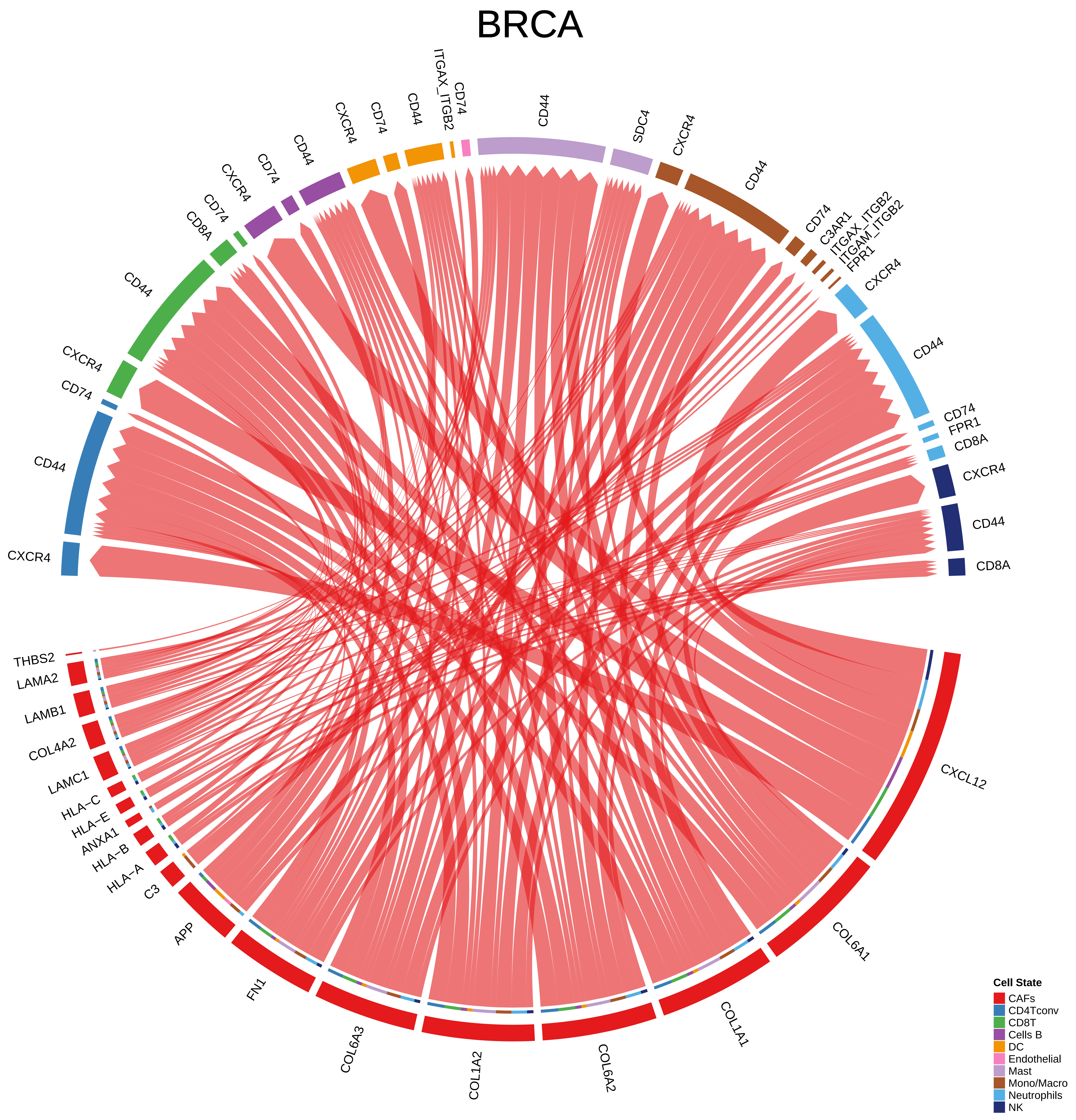
BRCA
CD44
SDC4
CXCR4
CD44
CD74
C3AR1
ITGAX_ITGB2
ITGAM_ITGB2
FPR1
CXCR4
CD44
CD74
FPR1
CD8A
CXCR4
CD44
CD8A
CXCL12
COL6A1
COL1A1
COL6A2
COL1A2
COL6A3
FN1
APP
C3
HLA−A
HLA−B
ANXA1
HLA−E
HLA−C
LAMC1
COL4A2
LAMB1
LAMA2
THBS2
CXCR4
CD44
CD74
CXCR4
CD44
CD8A
CD74
CXCR4
CD74
CD44
CXCR4
CD74
CD44
ITGAX_ITGB2
CD74
Cell State
CAFs
CD4Tconv
CD8T
Cells B
DC
Endothelial
Mast
Mono/Macro
Neutrophils
NK

CHOL

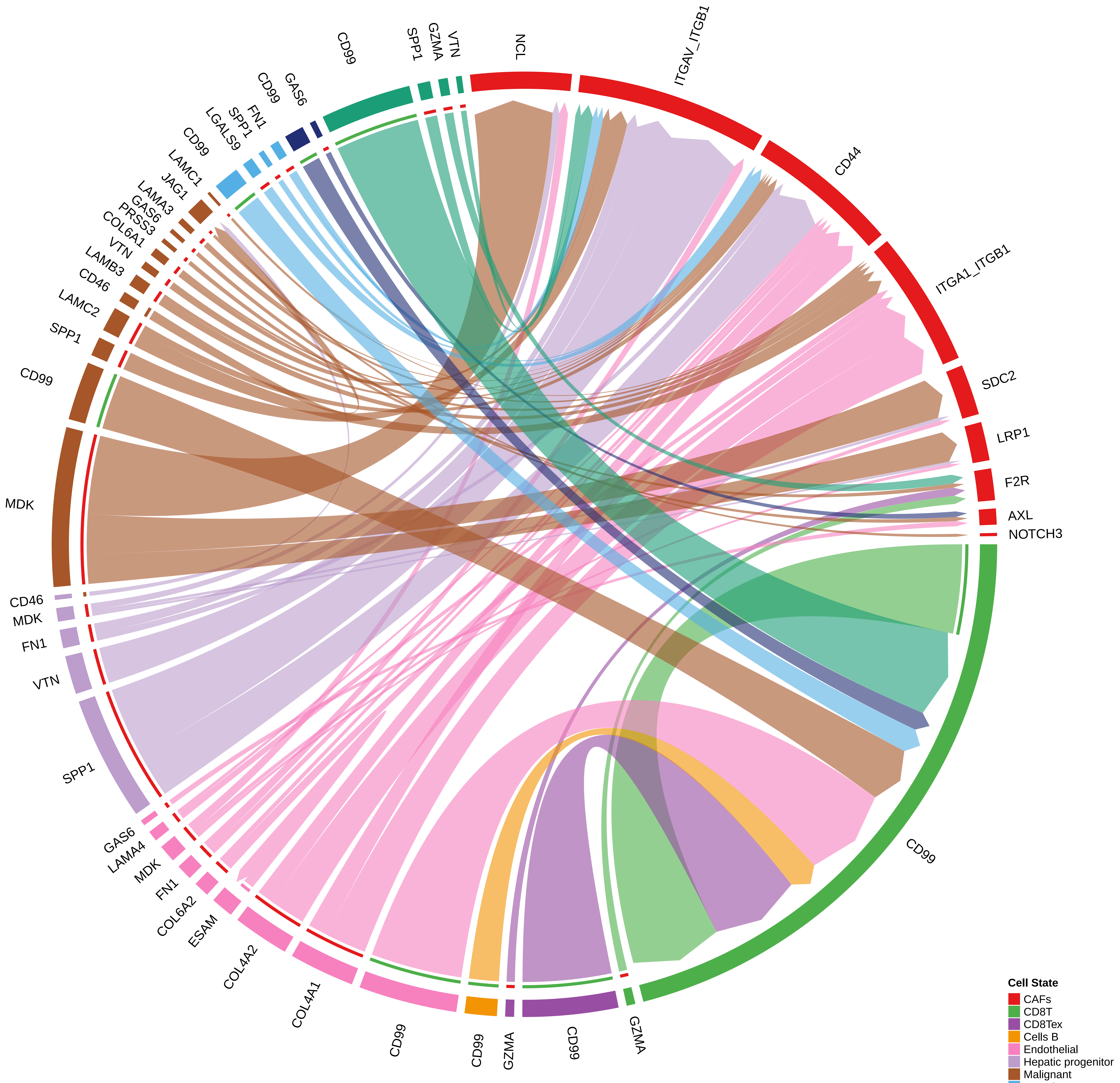

NCL
ITGAV_ITGB1
CD44
ITGA1_ITGB1
SDC2
LRP1
F2R
AXL
NOTCH3
CD99
GZMA
CD99
GZMA
CD99
CD99
COL4A1
COL4A2
ESAM
COL6A2
FN1
MDK
LAMA4
GAS6
SPP1
VTN
FN1
MDK
CD46
MDK
CD99
SPP1
LAMC2
CD46
LAMB3
VTN
COL6A1
PRSS3
GAS6
LAMA3
JAG1
LAMC1
CD99
LGALS9
SPP1
FN1
CD99
GAS6
CD99
SPP1
GZMA
VTN
Cell State
CAFs
CD8T
CD8Tex
Cells B
Endothelial
Hepatic progenitor
Malignant
Mono/Macro
Plasma
Tprolif

CHOL

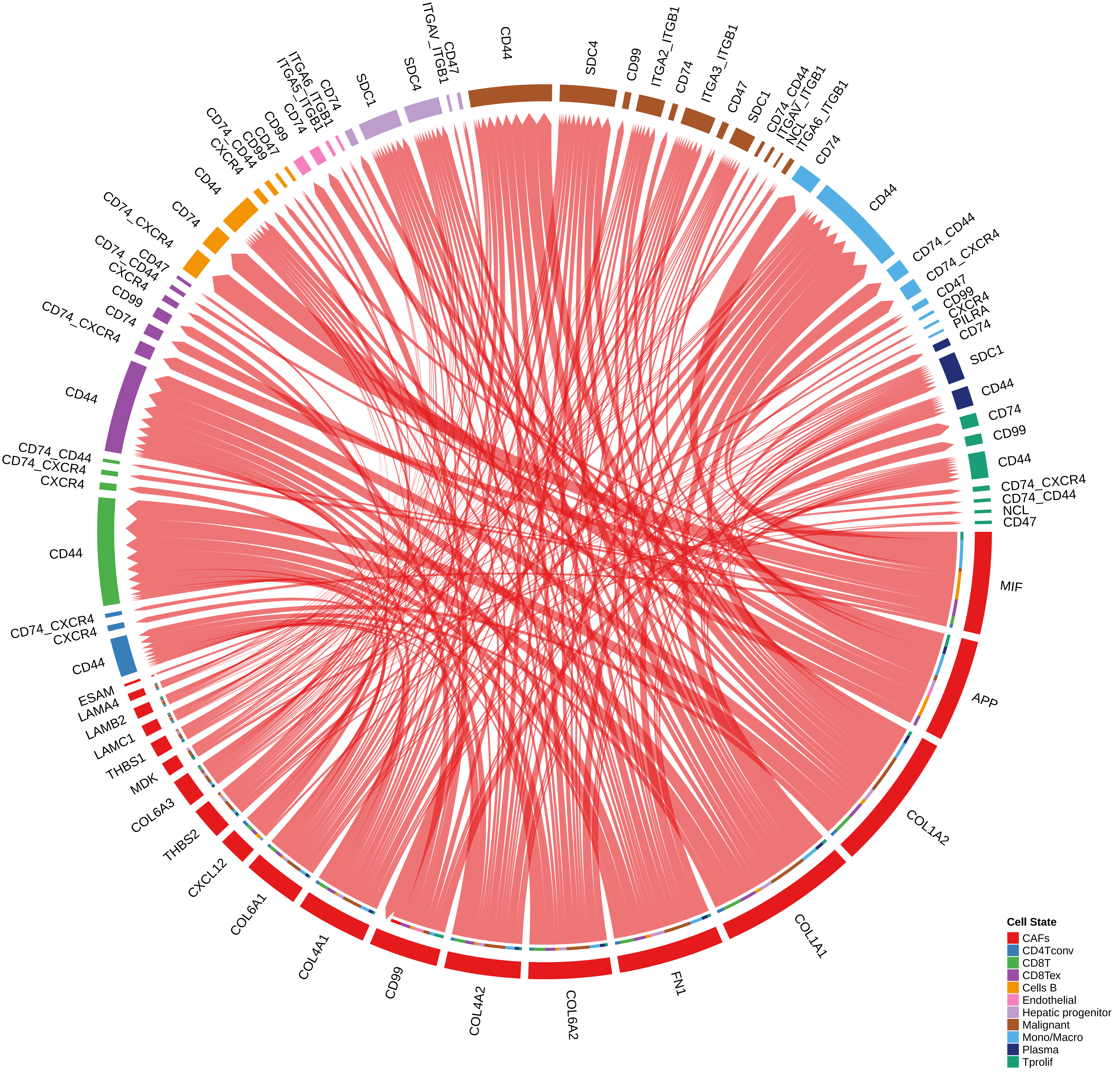

CD44
SDC4
CD99
ITGA2_ITGB1
CD74
ITGA3_ITGB1
CD47
SDC1
CD74_CD44
ITGAV_ITGB1
NCL
ITGA6_ITGB1
CD74
CD44
CD74_CD44
CD74_CXCR4
CD47
CD99
CXCR4
PILRA
CD74
SDC1
CD44
CD74
CD99
CD44
CD74_CXCR4
CD74_CD44
NCL
CD47
MIF
APP
COL1A2
COL1A1
FN1
COL6A2
COL4A2
CD99
COL4A1
COL6A1
CXCL12
THBS2
COL6A3
MDK
THBS1
LAMC1
LAMB2
LAMA4
ESAM
CD44
CXCR4
CD74_CXCR4
CD44
CXCR4
CD74_CXCR4
CD74_CD44
CD44
CD74_CXCR4
CD74
CD99
CXCR4
CD74_CD44
CD47
CD74_CXCR4
CD74
CD44
CXCR4
CD74_CD44
CD47
CD99
CD74
ITGA5_ITGB1
ITGA6_ITGB1
CD74
SDC1
SDC4
ITGAV_ITGB1
CD47
Cell State
CAFs
CD4Tconv
CD8T
CD8Tex
Cells B
Endothelial
Hepatic progenitor
Malignant
Mono/Macro
Plasma
Tprolif

CRC

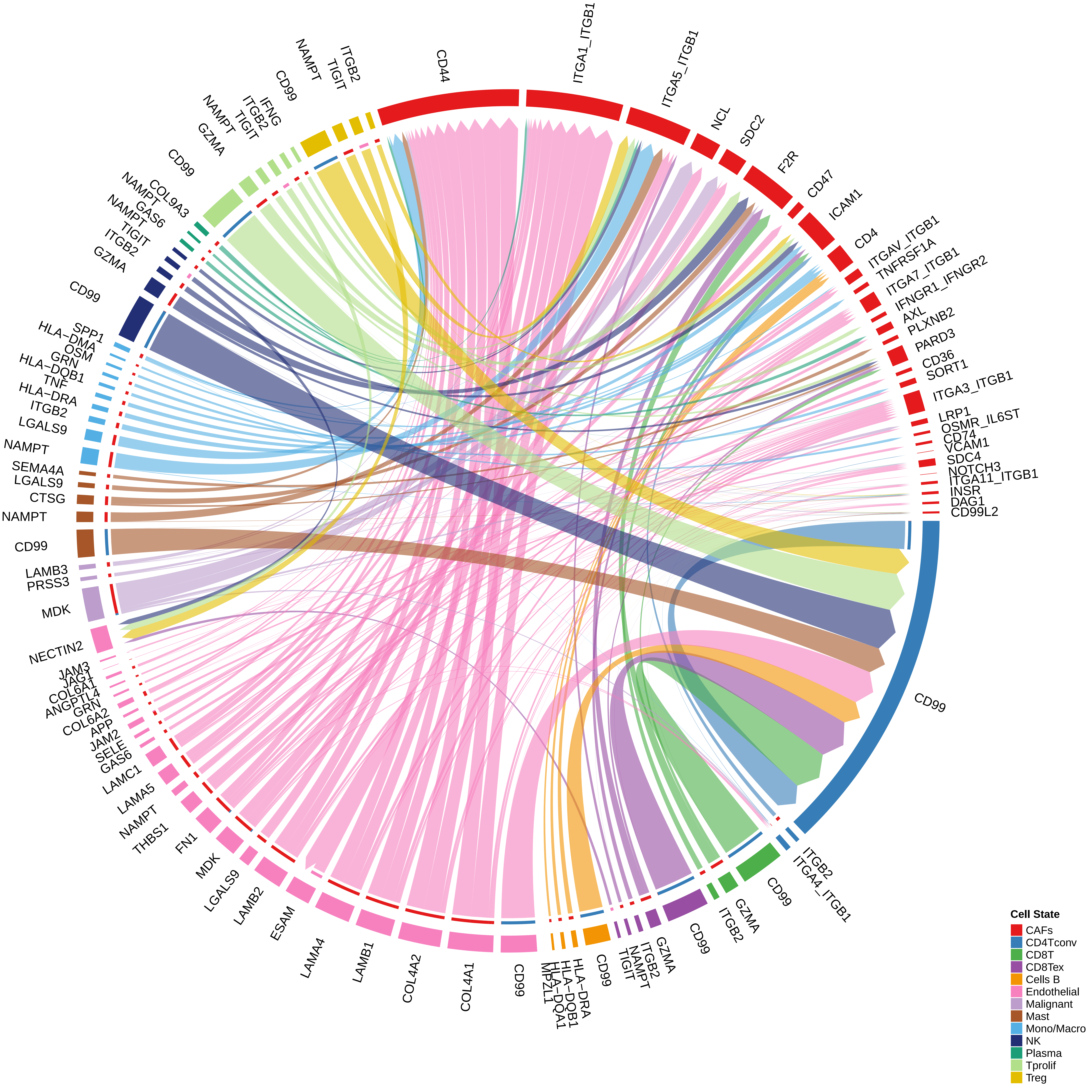
CD44
ITGA1_ITGB1
ITGA5_ITGB1
NCL
SDC2
F2R
CD47
ICAM1
CD4
ITGAV_ITGB1
TNFRSF1A
ITGA7_ITGB1
IFNGR1_IFNGR2
AXL
PLXNB2
PARD3
CD36
SORT1
ITGA3_ITGB1
LRP1
OSMR_IL6ST
CD74
VCAM1
SDC4
NOTCH3
ITGA11_ITGB1
INSR
DAG1
CD99L2
CD99
ITGB2
ITGA4_ITGB1
CD99
GZMA
ITGB2
CD99
GZMA
ITGB2
NAMPT
TIGIT
CD99
HLA-DRA
HLA-DQB1
HLA-DQA1
MPZL1
CD99
COL4A1
COL4A2
LAMB1
LAMA4
ESAM
LAMB2
LGALS9
MDK
FN1
THBS1
NAMPT
LAMA5
LAMC1
GAS6
SELE
JAM2
APP
COL6A2
GRN
ANGPTL4
COL6A1
JAG1
JAM3
NECTIN2
MDK
PRSS3
LAMB3
CD99
NAMPT
CTSG
LGALS9
SEMA4A
NAMPT
LGALS9
ITGB2
HLA-DRA
TNF
HLA-DQB1
GRN
OSM
HLA-DMA
SPP1
CD99
GZMA
ITGB2
TIGIT
NAMPT
GAS6
NAMPT
COL9A3
CD99
GZMA
NAMPT
TIGIT
ITGB2
IFNG
CD99
NAMPT
TIGIT
ITGB2
Cell State
CAFs
CD4Tconv
CD8T
CD8Tex
Cells B
Endothelial
Malignant
Mast
Mono/Macro
NK
Plasma
Tprolif
Treg

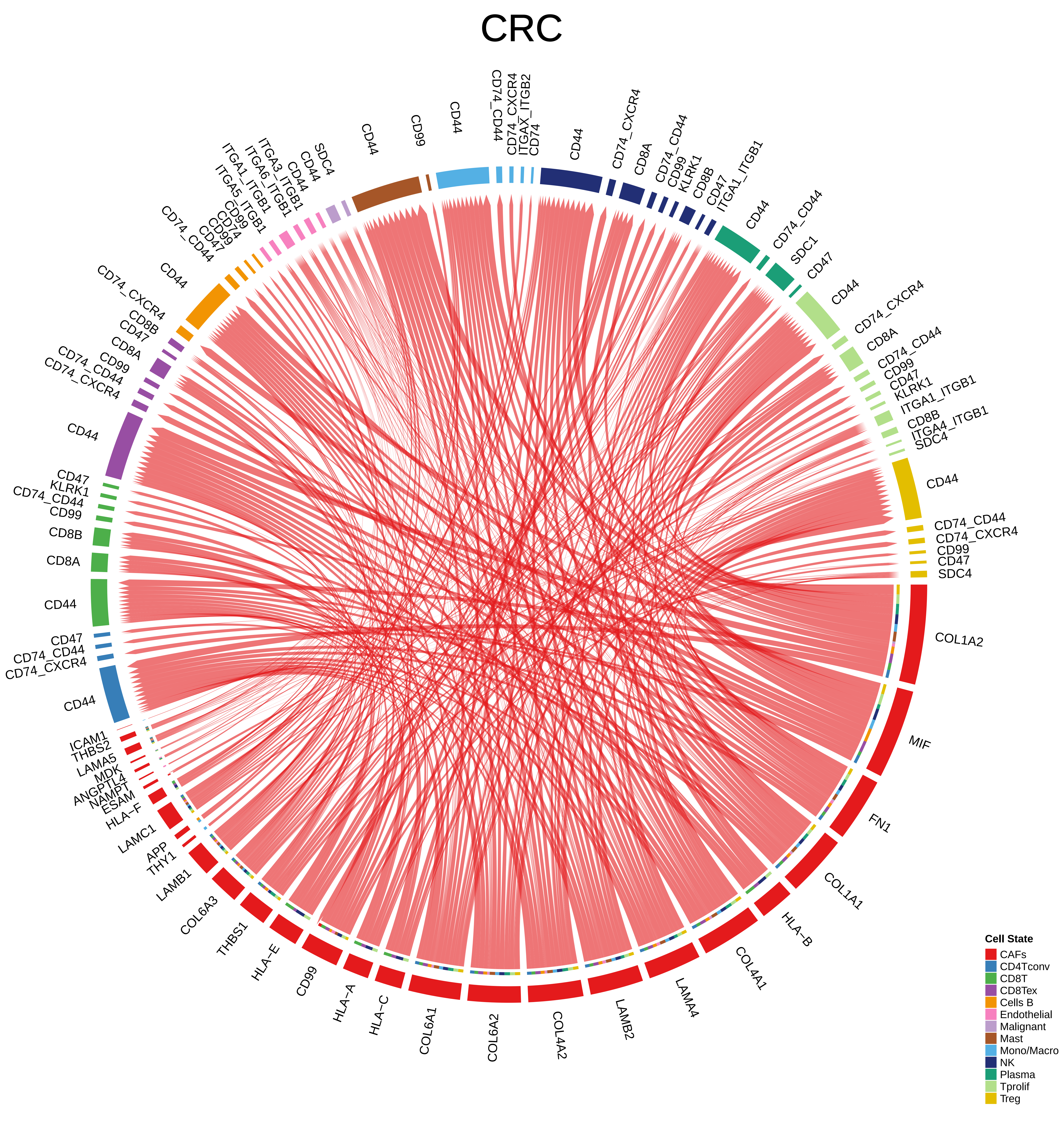
CRC
Cell State
CAFs
CD4Tconv
CD8T
CD8Tex
Cells B
Endothelial
Malignant
Mast
Mono/Macro
NK
Plasma
Tprolif
Treg
COL1A2
MIF
FN1
COL1A1
HLA−B
COL4A1
LAMA4
LAMB2
COL4A2
COL6A2
COL6A1
HLA−C
HLA−A
CD99
HLA−E
THBS1
COL6A3
LAMB1
THY1
APP
LAMC1
HLA−F
ESAM
NAMPT
ANGPTL4
MDK
LAMA5
THBS2
ICAM1
CD44
CD74_CXCR4
CD74_CD44
CD47
CD8A
CD8B
CD99
KLRK1
ITGA1_ITGB1
ITGA5_ITGB1
ITGA6_ITGB1
ITGA3_ITGB1
CD74
SDC4
ITGAX_ITGB2
SDC1
ITGA4_ITGB1

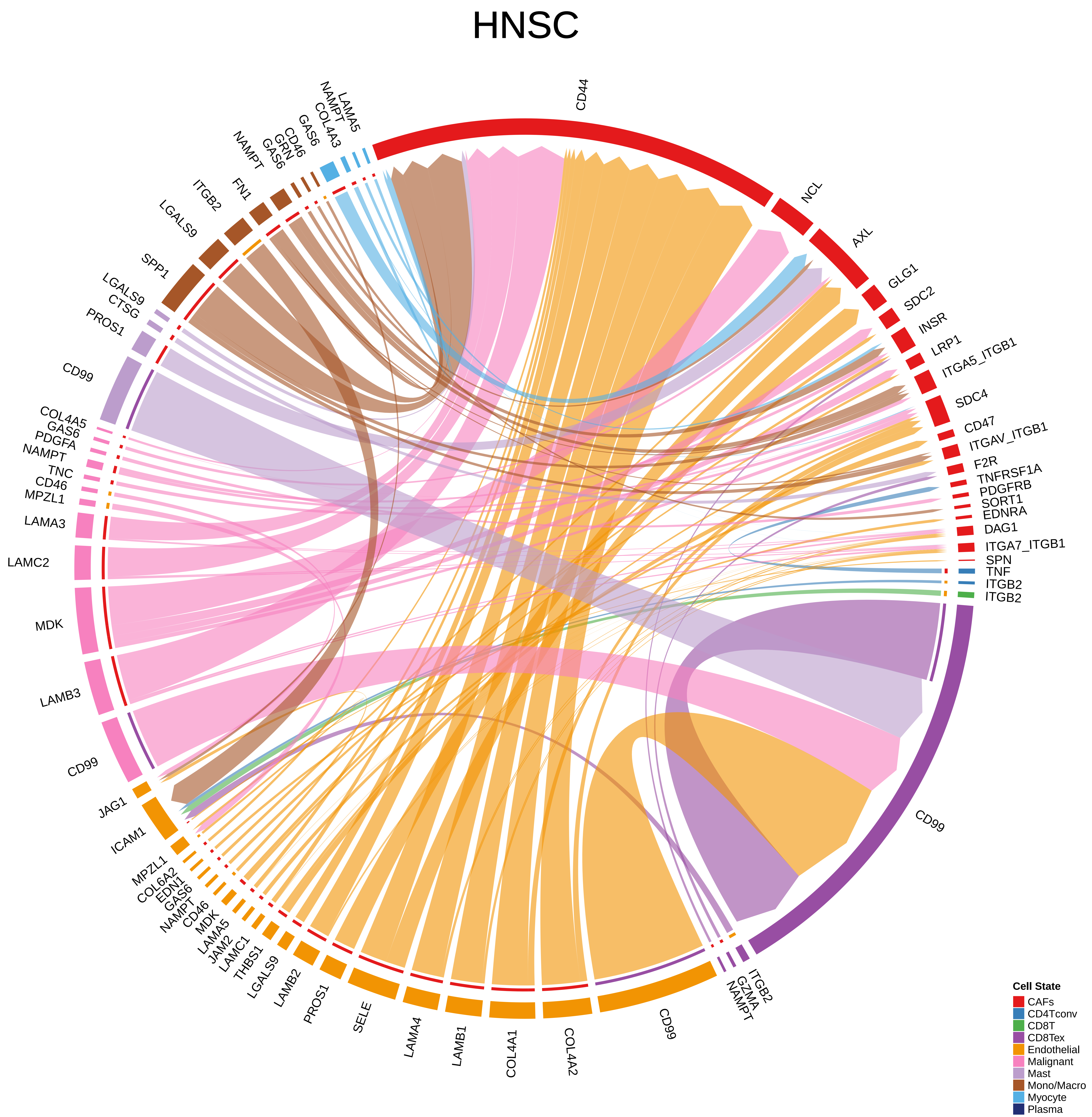
HNSC
CD44
NCL
AXL
GLG1
SDC2
INSR
LRP1
ITGA5_ITGB1
SDC4
CD47
ITGAV_ITGB1
F2R
TNFRSF1A
PDGFRB
SORT1
EDNRA
DAG1
ITGA7_ITGB1
SPN
TNF
ITGB2
ITGB2
CD99
ITGB2
GZMA
NAMPT
CD99
COL4A2
COL4A1
LAMB1
LAMA4
SELE
PROS1
LAMB2
LGALS9
THBS1
LAMC1
JAM2
LAMA5
MDK
CD46
NAMPT
GAS6
EDN1
COL6A2
MPZL1
ICAM1
JAG1
CD99
LAMB3
MDK
LAMC2
LAMA3
MPZL1
CD46
TNC
NAMPT
PDGFA
GAS6
COL4A5
CD99
PROS1
CTSG
LGALS9
SPP1
LGALS9
ITGB2
FN1
NAMPT
GAS6
GRN
CD46
GAS6
COL4A3
NAMPT
LAMA5
Cell State
CAFs
CD4Tconv
CD8T
CD8Tex
Endothelial
Malignant
Mast
Mono/Macro
Myocyte
Plasma

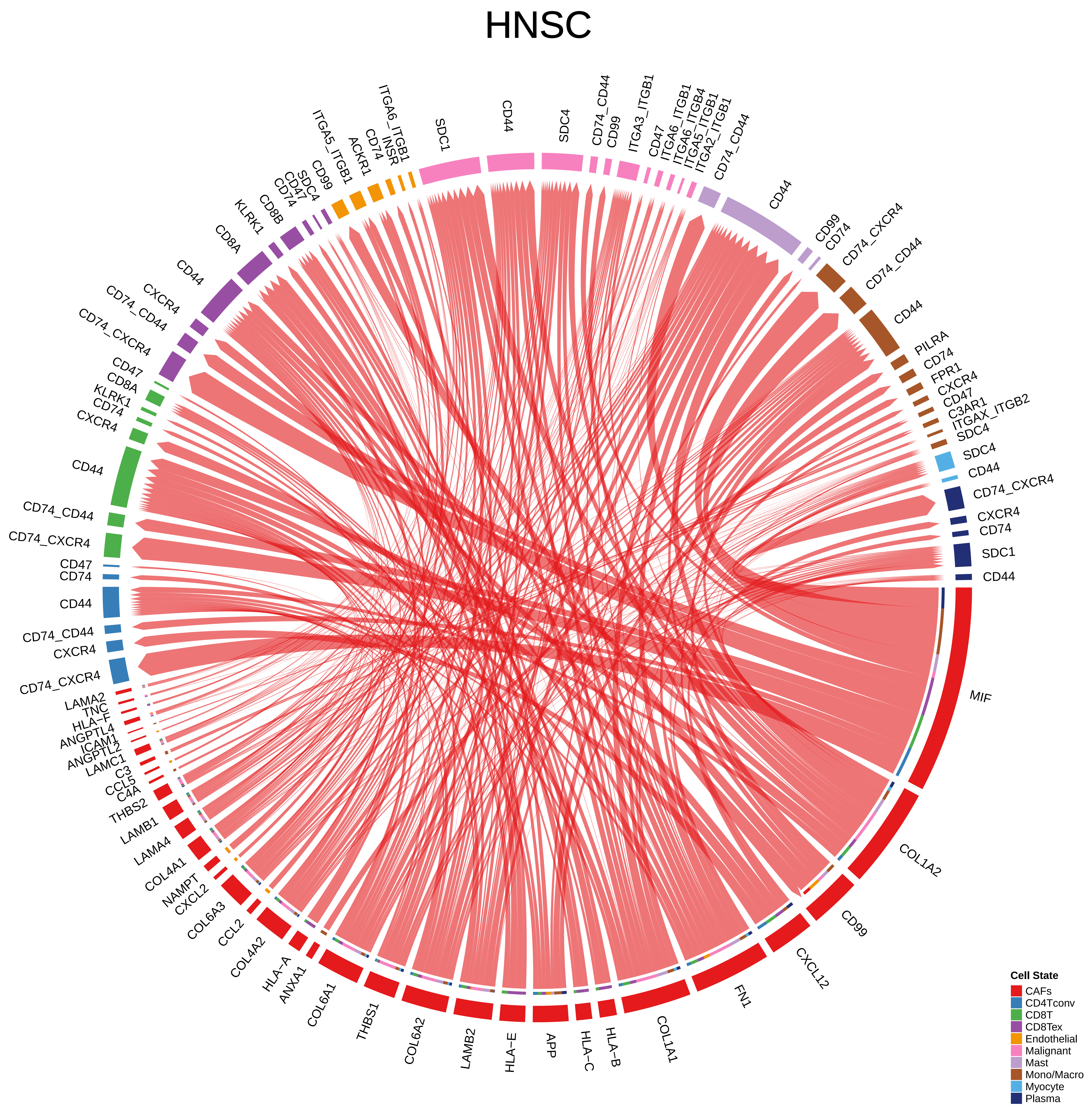
HNSC
SDC1
CD44
SDC4
CD74_CD44
CD99
ITGA3_ITGB1
CD47
ITGA6_ITGB1
ITGA6_ITGB4
ITGA5_ITGB1
ITGA2_ITGB1
CD74_CD44
CD44
CD99
CD74
CD74_CXCR4
CD74_CD44
CD44
PILRA
CD74
FPR1
CXCR4
CD47
C3AR1
ITGAX_ITGB2
SDC4
SDC4
CD44
CD74_CXCR4
CXCR4
CD74
SDC1
CD44
MIF
COL1A2
CD99
CXCL12
FN1
COL1A1
HLA−B
HLA−C
APP
HLA−E
LAMB2
COL6A2
THBS1
COL6A1
ANXA1
HLA−A
COL4A2
CCL2
COL6A3
CXCL2
NAMPT
COL4A1
LAMA4
LAMB1
THBS2
C4A
CCL5
C3
LAMC1
ANGPTL2
ICAM1
ANGPTL4
HLA−F
TNC
LAMA2
CD74_CXCR4
CXCR4
CD74_CD44
CD44
CD74
CD47
CD74_CXCR4
CD74_CD44
CD44
CXCR4
CD74
KLRK1
CD8A
CD47
CD74_CXCR4
CD74_CD44
CXCR4
CD44
CD8A
KLRK1
CD8B
CD74
CD47
SDC4
CD99
ITGA5_ITGB1
ACKR1
CD74
INSR
ITGA6_ITGB1
Cell State
CAFs
CD4Tconv
CD8T
CD8Tex
Endothelial
Malignant
Mast
Mono/Macro
Myocyte
Plasma

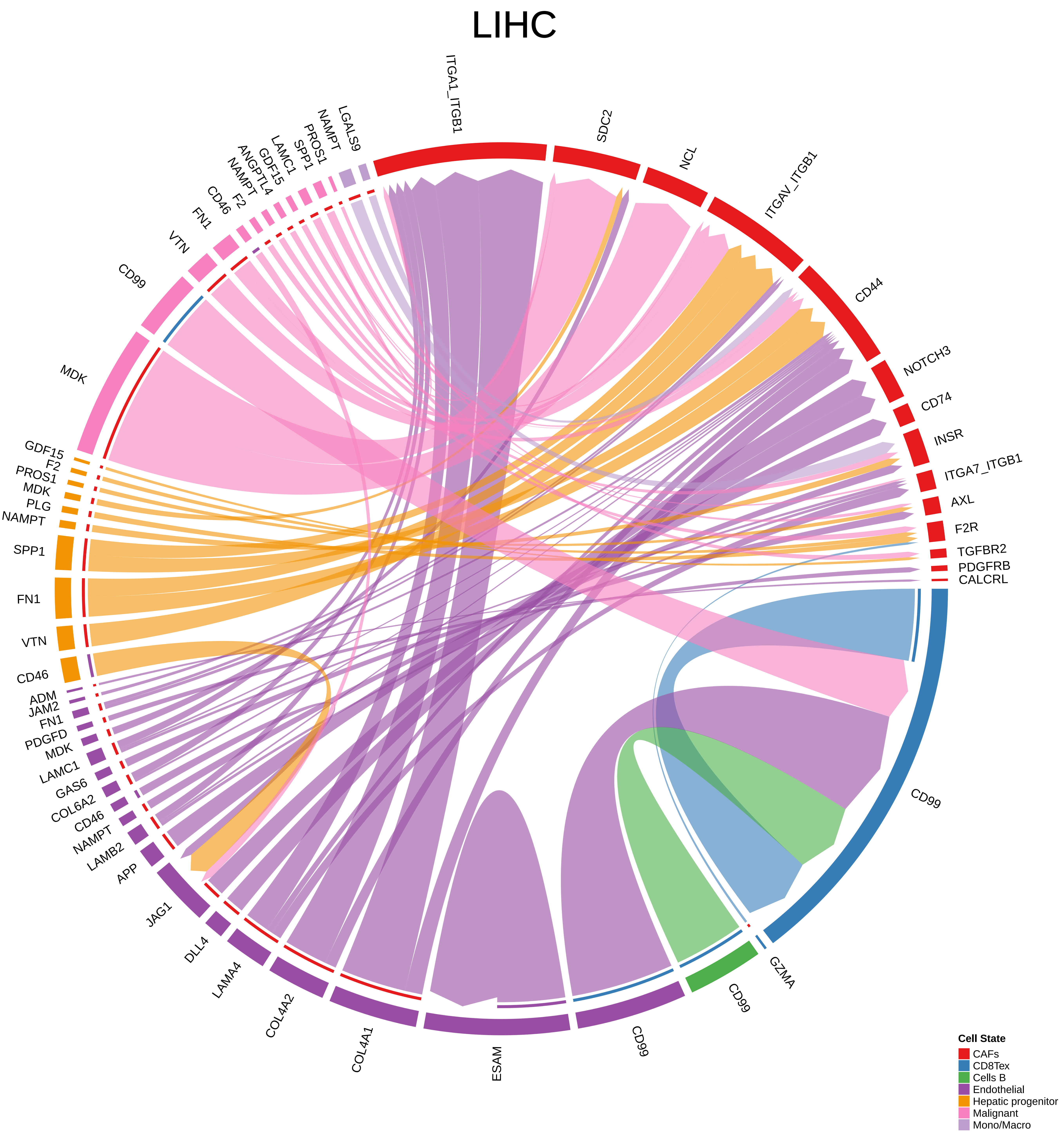

LIHC
ITGA1_ITGB1
SDC2
NCL
ITGAV_ITGB1
CD44
NOTCH3
CD74
INSR
ITGA7_ITGB1
AXL
F2R
TGFBR2
PDGFRB
CALCRL
CD99
GZMA
CD99
CD99
ESAM
COL4A1
COL4A2
LAMA4
DLL4
JAG1
APP
LAMB2
NAMPT
CD46
COL6A2
GAS6
LAMC1
MDK
PDGFD
FN1
JAM2
ADM
CD46
VTN
FN1
SPP1
NAMPT
PLG
MDK
PROS1
F2
GDF15
MDK
CD99
VTN
FN1
CD46
F2
NAMPT
ANGPTL4
GDF15
LAMC1
SPP1
PROS1
NAMPT
LGALS9
Cell State
CAFs
CD8Tex
Cells B
Endothelial
Hepatic progenitor
Malignant
Mono/Macro

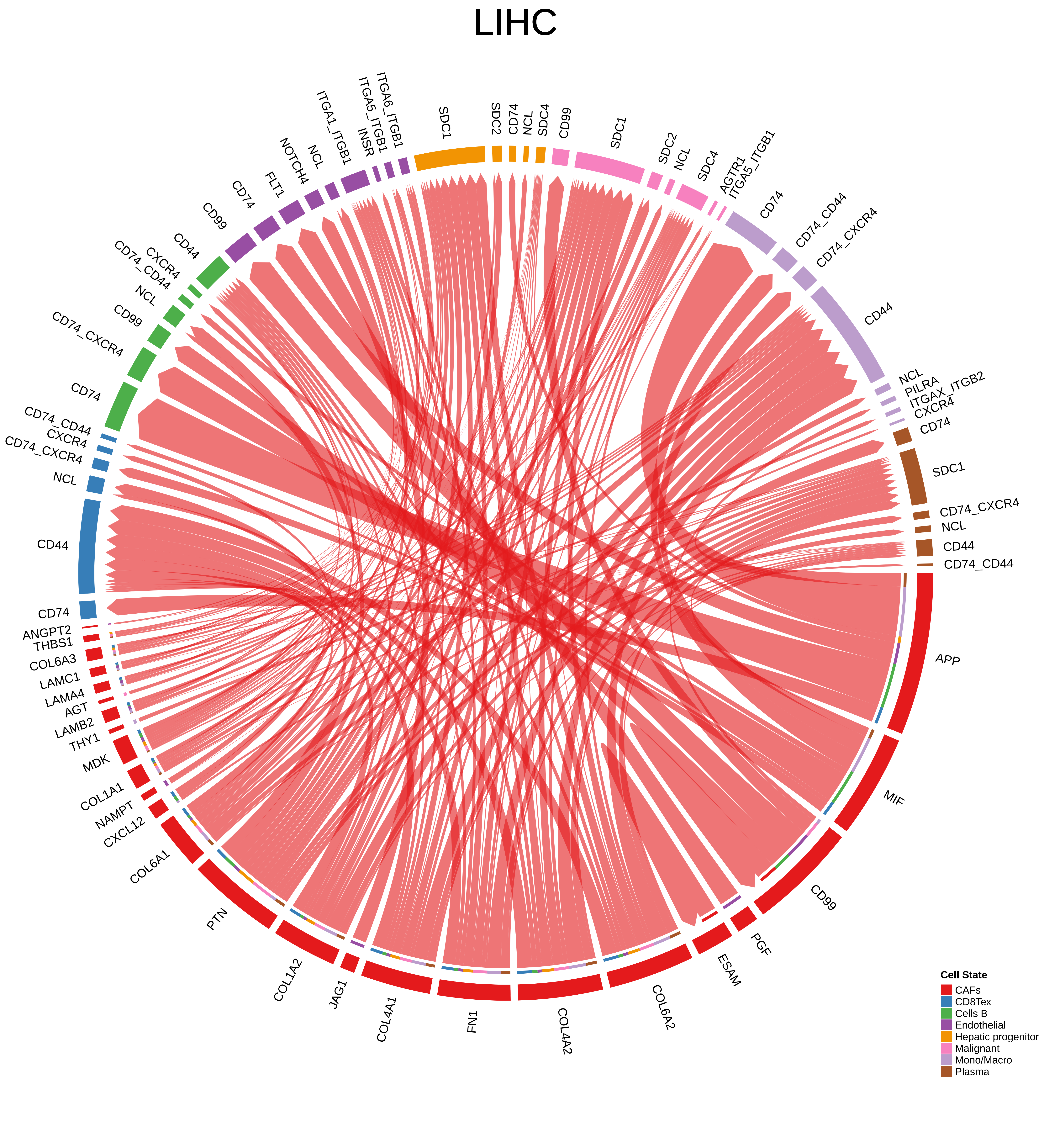
LIHC
SDC1
SDC2
CD74
NCL
SDC4
CD99
SDC1
SDC2
NCL
SDC4
AGTR1
ITGA5_ITGB1
CD74
CD74_CD44
CD74_CXCR4
CD44
NCL
PILRA
ITGAX_ITGB2
CXCR4
CD74
SDC1
CD74_CXCR4
NCL
CD44
CD74_CD44
APP
MIF
CD99
PGF
ESAM
COL6A2
COL4A2
FN1
COL4A1
JAG1
COL1A2
PTN
COL6A1
CXCL12
NAMPT
COL1A1
MDK
THY1
LAMB2
AGT
LAMA4
LAMC1
COL6A3
THBS1
ANGPT2
CD74
CD44
NCL
CD74_CXCR4
CXCR4
CD74_CD44
CD74
CD74_CXCR4
CD99
NCL
CD74_CD44
CXCR4
CD44
CD99
CD74
FLT1
NOTCH4
NCL
ITGA1_ITGB1
INSR
ITGA5_ITGB1
ITGA6_ITGB1
Cell State
CAFs
CD8Tex
Cells B
Endothelial
Hepatic progenitor
Malignant
Mono/Macro
Plasma

NET

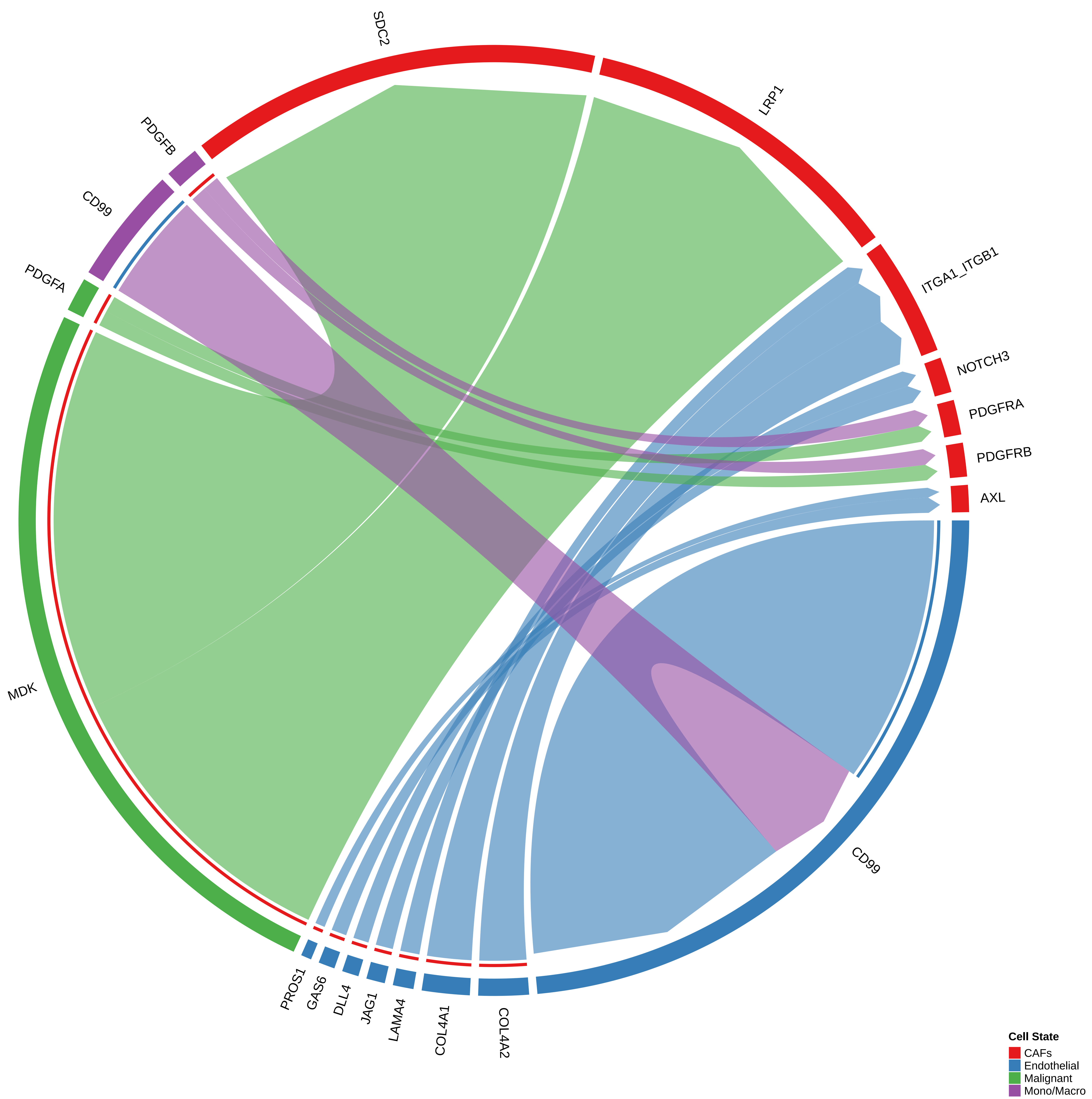
SDC2
LRP1
ITGA1_ITGB1
NOTCH3
PDGFRA
PDGFRB
AXL
CD99
COL4A2
COL4A1
LAMA4
JAG1
DLL4
GAS6
PROS1
MDK
PDGFA
CD99
PDGFB
Cell State
CAFs
Endothelial
Malignant
Mono/Macro

NET

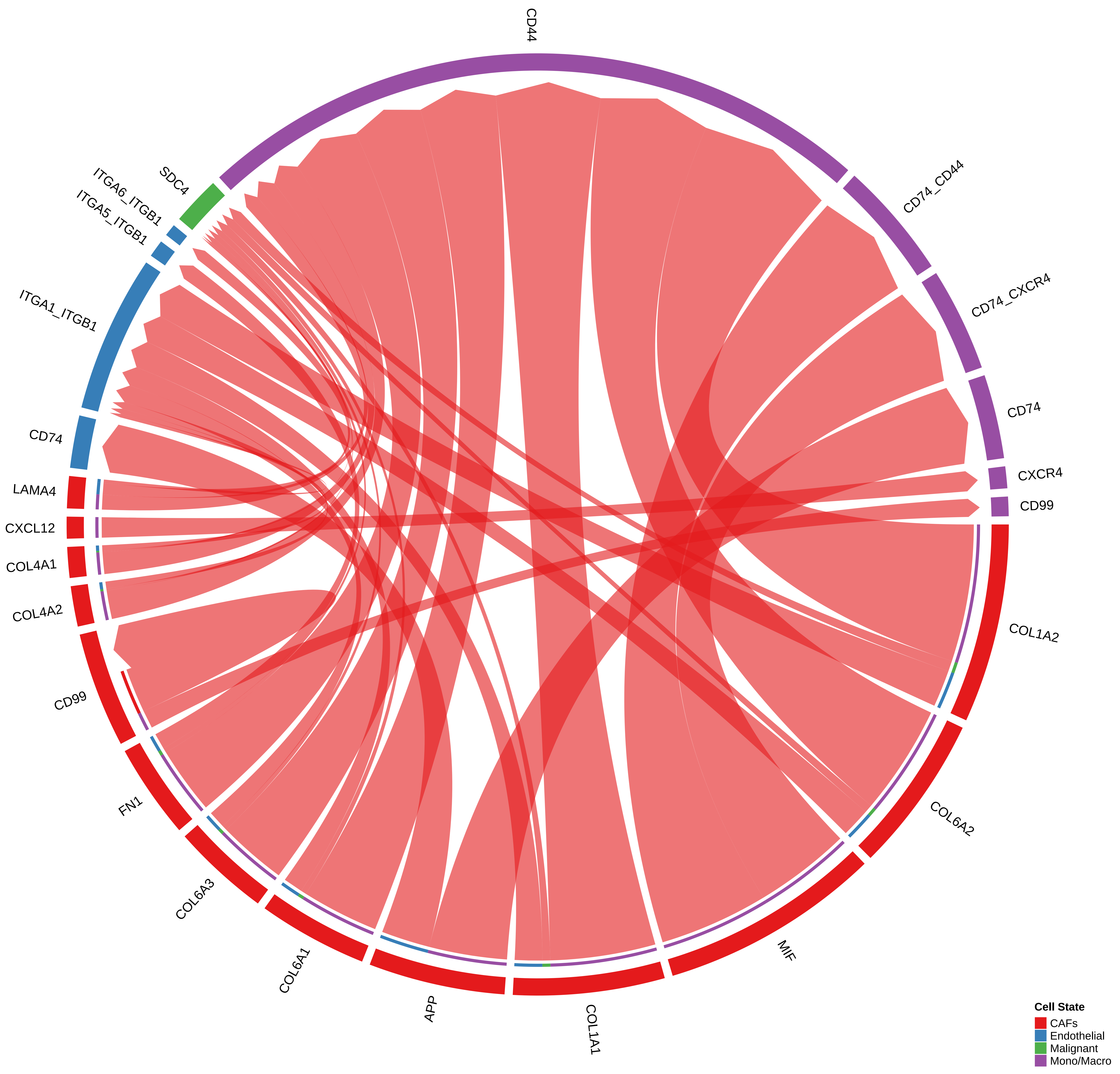
CD44
CD74_CD44
CD74_CXCR4
CD74
CXCR4
CD99
COL1A2
COL6A2
MIF
COL1A1
APP
COL6A1
COL6A3
FN1
CD99
COL4A2
COL4A1
CXCL12
LAMA4
CD74
ITGA1_ITGB1
ITGA5_ITGB1
ITGA6_ITGB1
SDC4
Cell State
CAFs
Endothelial
Malignant
Mono/Macro

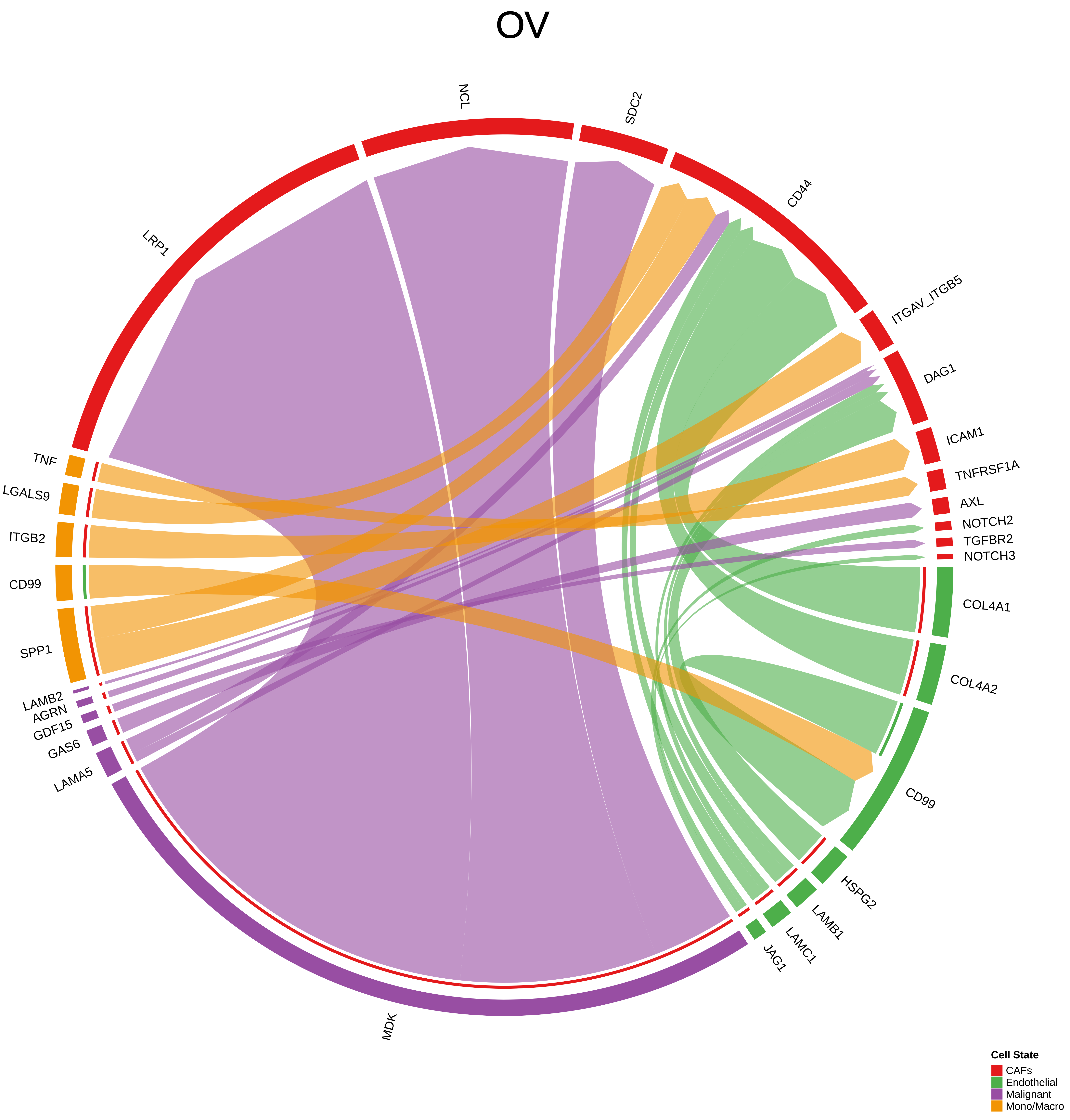

OV
NCL
SDC2
CD44
ITGAV_ITGB5
DAG1
ICAM1
TNFRSF1A
AXL
NOTCH2
TGFBR2
NOTCH3
COL4A1
COL4A2
CD99
HSPG2
LAMB1
LAMC1
JAG1
MDK
LAMA5
GAS6
GDF15
AGRN
LAMB2
SPP1
CD99
ITGB2
LGALS9
TNF
LRP1
Cell State
CAFs
Endothelial
Malignant
Mono/Macro

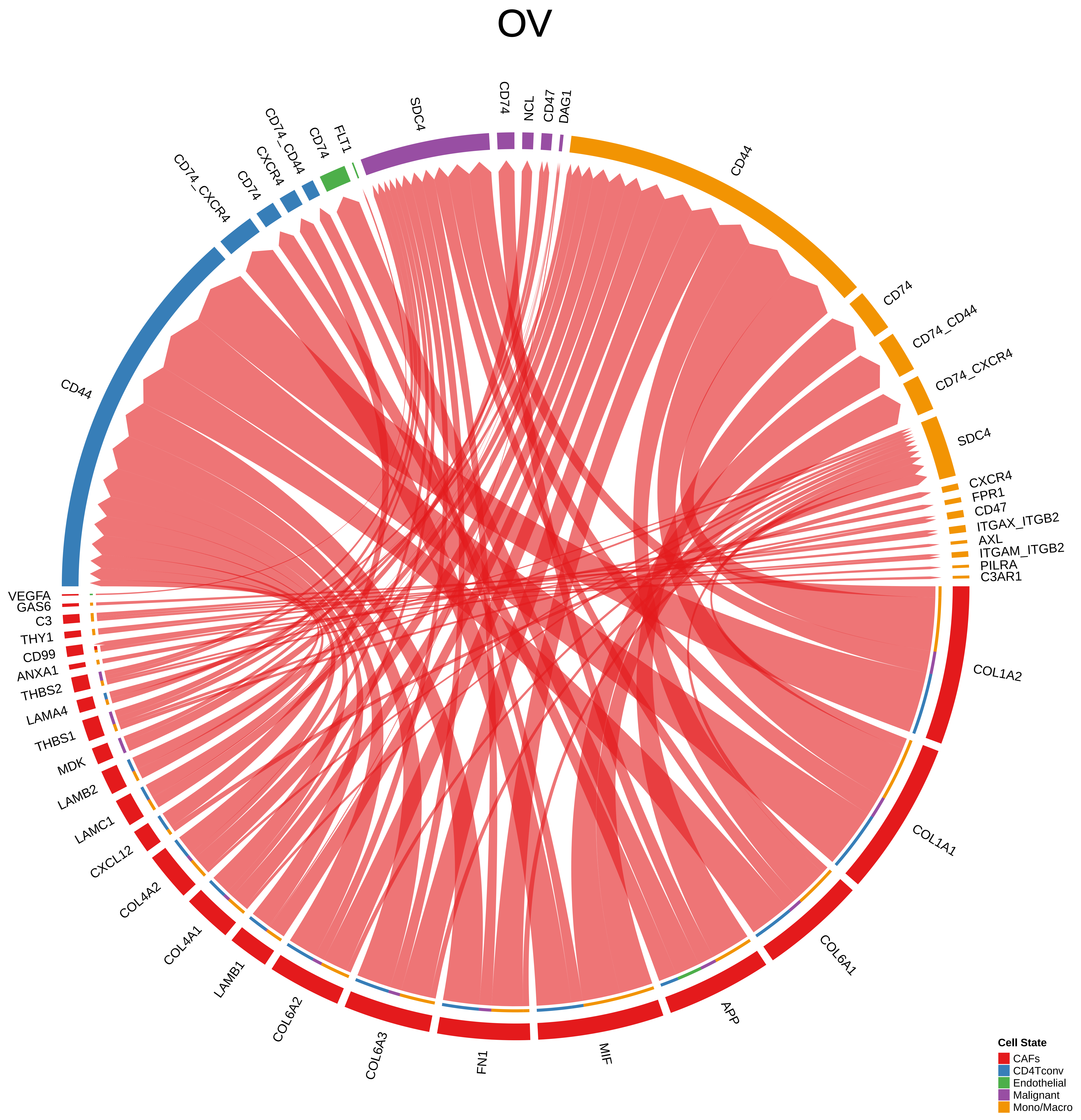
OV
CD44
CD74_CXCR4
CD74
CXCR4
CD74_CD44
CD74
FLT1
SDC4
CD74
NCL
CD47
DAG1
CD44
CD74
CD74_CD44
CD74_CXCR4
SDC4
CXCR4
FPR1
CD47
ITGAX_ITGB2
AXL
ITGAM_ITGB2
PILRA
C3AR1
COL1A2
COL1A1
COL6A1
APP
MIF
FN1
COL6A3
COL6A2
LAMB1
COL4A1
COL4A2
CXCL12
LAMC1
LAMB2
MDK
THBS1
LAMA4
THBS2
ANXA1
CD99
THY1
C3
GAS6
VEGFA
Cell State
CAFs
CD4Tconv
Endothelial
Malignant
Mono/Macro

PAAD

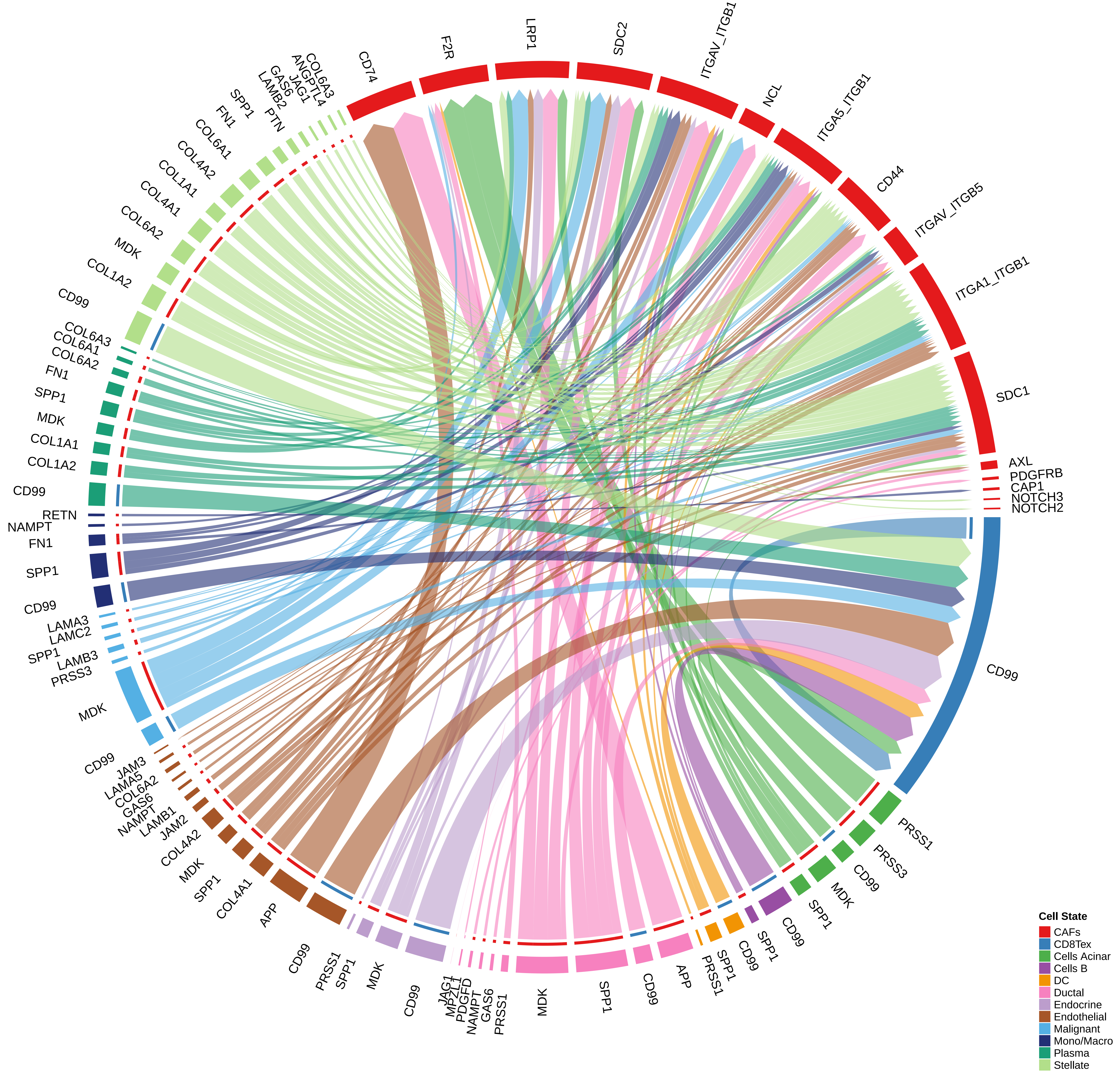

CD74
F2R
LRP1
SDC2
ITGAV_ITGB1
NCL
ITGA5_ITGB1
CD44
ITGAV_ITGB5
ITGA1_ITGB1
SDC1
AXL
PDGFRB
CAP1
NOTCH3
NOTCH2
CD99
PRSS1
PRSS3
CD99
MDK
SPP1
CD99
SPP1
CD99
SPP1
PRSS1
APP
CD99
SPP1
MDK
PRSS1
GAS6
NAMPT
PDGFD
MPZL1
JAG1
CD99
MDK
SPP1
PRSS1
CD99
APP
COL4A1
SPP1
MDK
COL4A2
JAM2
LAMB1
NAMPT
GAS6
COL6A2
LAMA5
JAM3
CD99
MDK
PRSS3
LAMB3
SPP1
LAMC2
LAMA3
CD99
SPP1
FN1
NAMPT
RETN
CD99
COL1A2
COL1A1
MDK
SPP1
FN1
COL6A2
COL6A1
COL6A3
CD99
COL1A2
MDK
COL6A2
COL4A1
COL1A1
COL4A2
COL6A1
FN1
SPP1
PTN
LAMB2
GAS6
JAG1
ANGPTL4
COL6A3
Cell State
CAFs
CD8Tex
Cells Acinar
Cells B
DC
Ductal
Endocrine
Endothelial
Malignant
Mono/Macro
Plasma
Stellate

PAAD

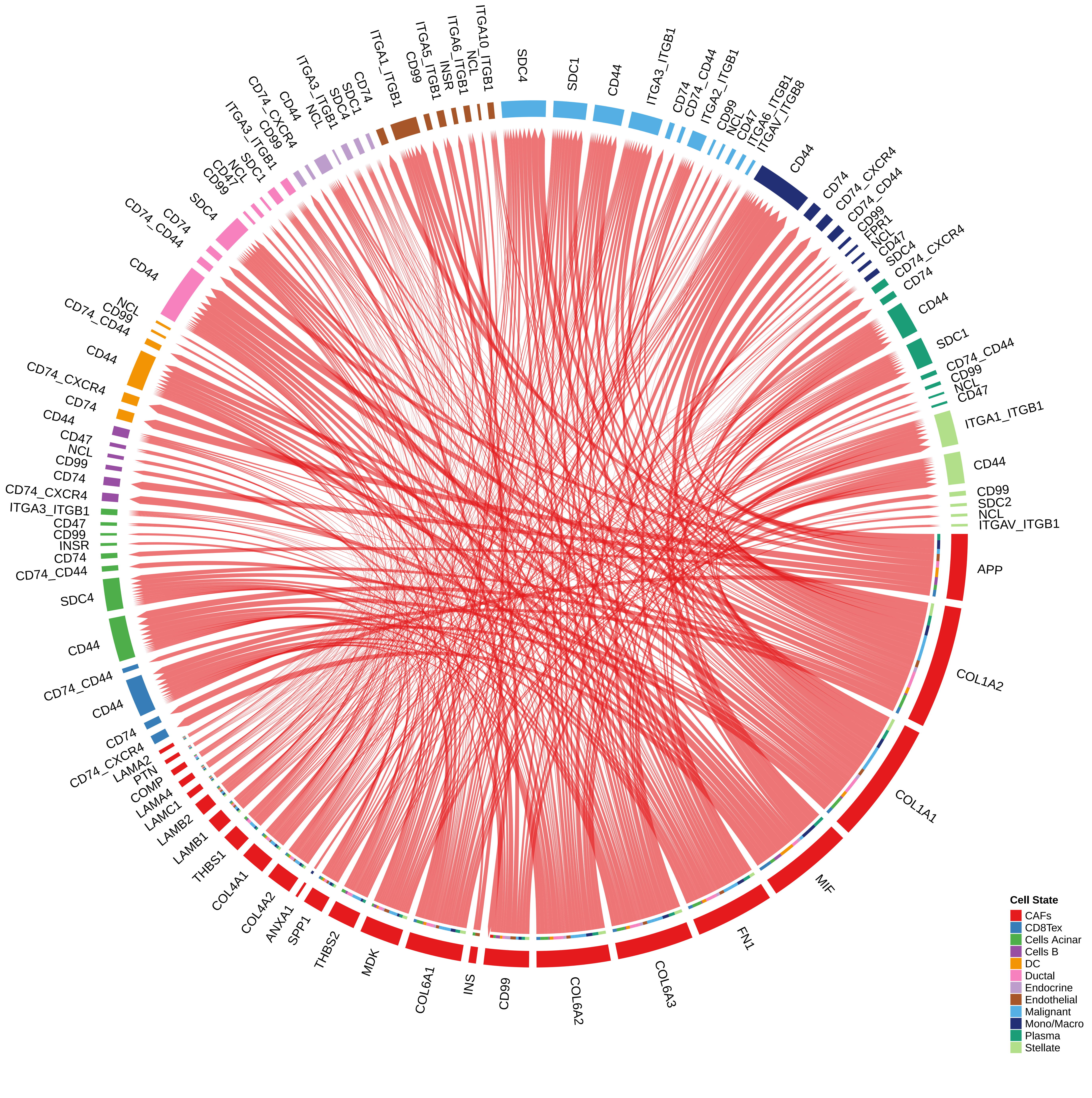

SDC4
SDC1
CD44
ITGA3_ITGB1
CD74
CD74_CD44
ITGA2_ITGB1
CD99
NCL
CD47
ITGA6_ITGB1
ITGAV_ITGB8
CD44
CD74
CD74_CXCR4
CD74_CD44
CD99
FPR1
NCL
CD47
SDC4
CD74_CXCR4
CD74
CD44
SDC1
CD74_CD44
CD99
NCL
CD47
ITGA1_ITGB1
CD44
CD99
SDC2
NCL
ITGAV_ITGB1
APP
COL1A2
COL1A1
MIF
FN1
COL6A3
COL6A2
CD99
INS
COL6A1
MDK
THBS2
SPP1
ANXA1
COL4A2
COL4A1
THBS1
LAMB1
LAMB2
LAMC1
LAMA4
COMP
PTN
LAMA2
CD74_CXCR4
CD74
CD44
CD74_CD44
CD44
SDC4
CD74_CD44
CD74
INSR
CD99
CD47
ITGA3_ITGB1
CD74_CXCR4
CD74
CD99
NCL
CD47
CD44
CD74
CD74_CXCR4
CD44
CD74_CD44
CD99
NCL
CD44
CD74_CD44
CD74
SDC4
CD99
CD47
NCL
SDC1
ITGA3_ITGB1
CD99
CD74_CXCR4
CD44
NCL
ITGA3_ITGB1
SDC4
SDC1
CD74
ITGA1_ITGB1
CD99
ITGA5_ITGB1
INSR
ITGA6_ITGB1
NCL
ITGA10_ITGB1
Cell State
CAFs
CD8Tex
Cells Acinar
Cells B
DC
Ductal
Endocrine
Endothelial
Malignant
Mono/Macro
Plasma
Stellate

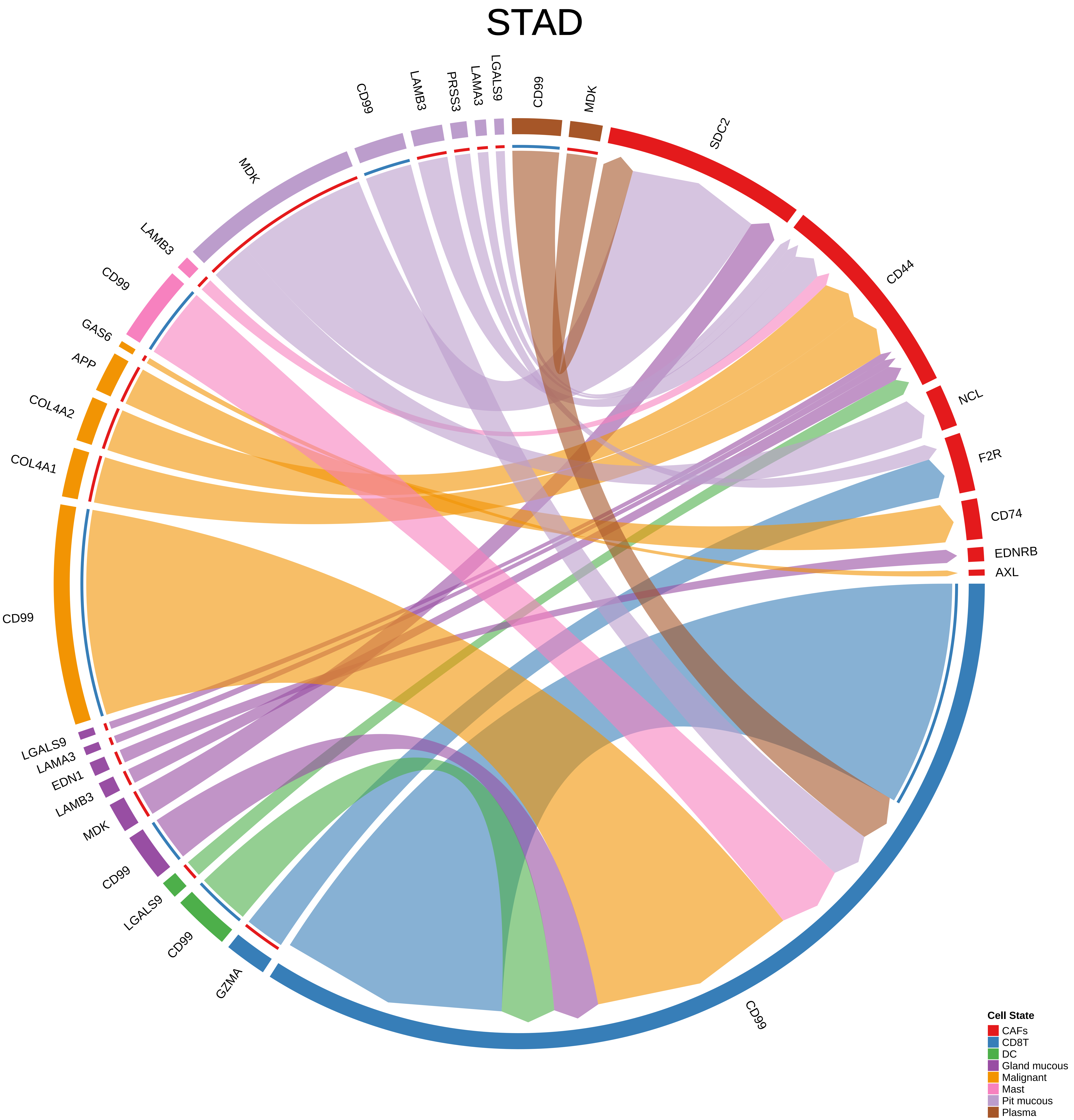
STAD
CD99
LAMB3
PRSS3
LAMA3
LGALS9
CD99
MDK
SDC2
MDK
CD44
LAMB3
CD99
GAS6
APP
NCL
COL4A2
F2R
COL4A1
CD74
EDNRB
AXL
CD99
LGALS9
LAMA3
EDN1
LAMB3
MDK
CD99
LGALS9
CD99
GZMA
CD99
Cell State
CAFs
CD8T
DC
Gland mucous
Malignant
Mast
Pit mucous
Plasma

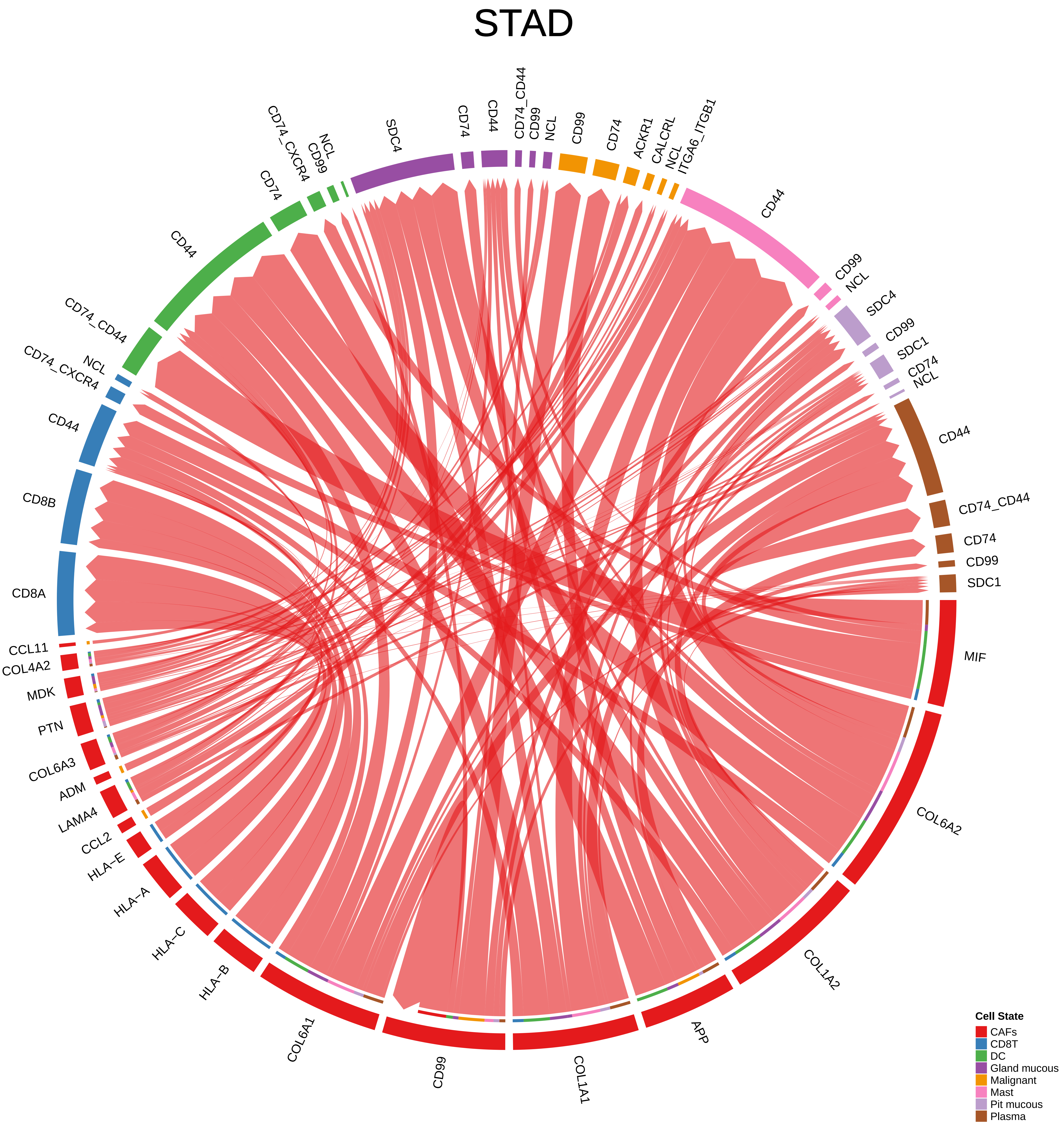
STAD
CD74_CD44
CD99
NCL
CD99
CD74
ACKR1
CALCRL
NCL
ITGA6_ITGB1
CD44
CD74
SDC4
NCL
CD99
CD74_CXCR4
CD74
CD44
CD74_CD44
NCL
CD74_CXCR4
CD44
CD8B
CD8A
CCL11
COL4A2
MDK
PTN
COL6A3
ADM
LAMA4
CCL2
HLA−E
HLA−A
HLA−C
HLA−B
COL6A1
CD99
COL1A1
APP
COL1A2
COL6A2
MIF
SDC1
CD99
CD74
CD74_CD44
CD44
NCL
CD74
SDC1
CD99
SDC4
NCL
CD99
CD44
Cell State
CAFs
CD8T
DC
Gland mucous
Malignant
Mast
Pit mucous
Plasma

SKCM

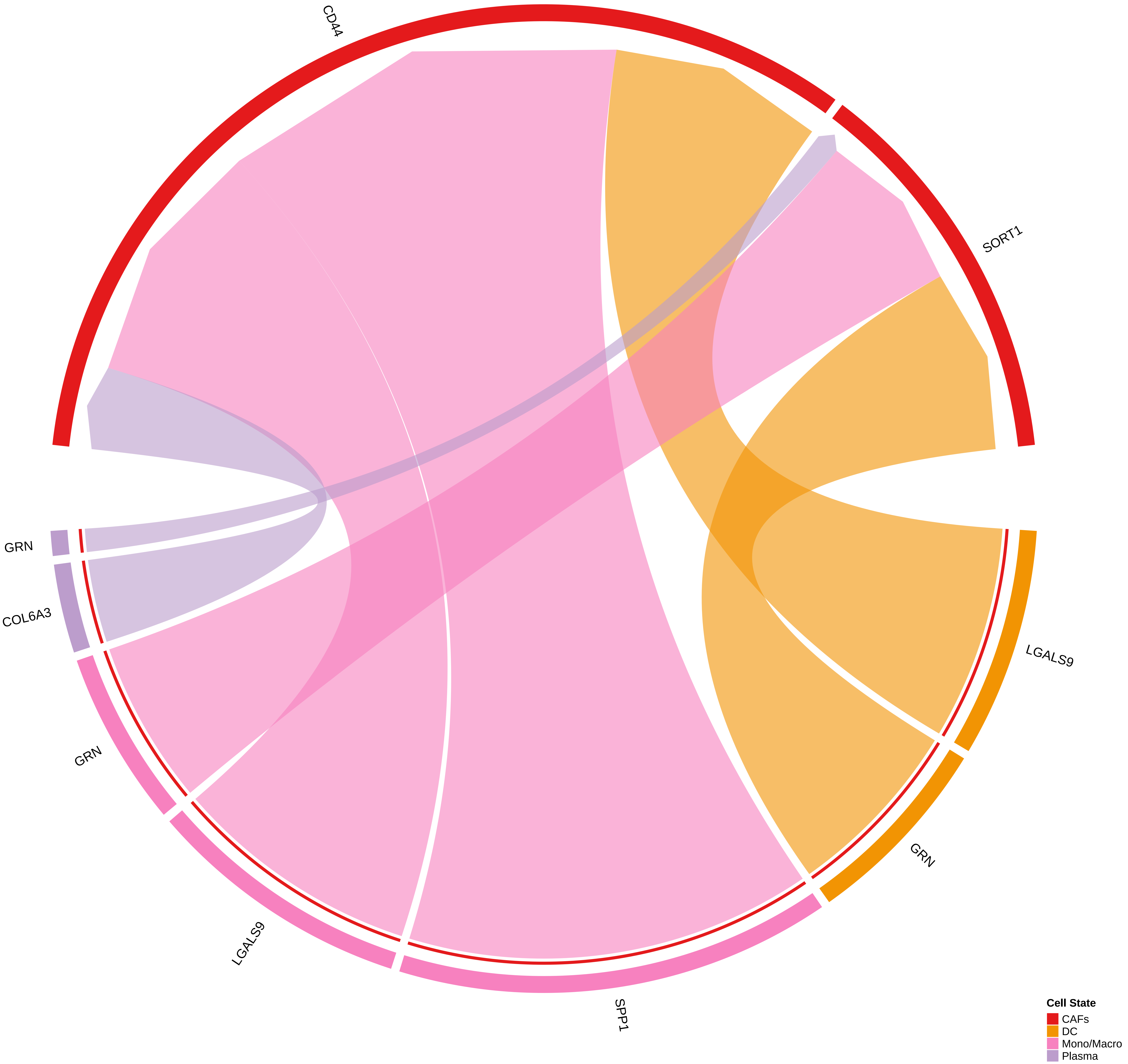
CD44
SORT1
LGALS9
GRN
SPP1
LGALS9
GRN
COL6A3
GRN
Cell State
CAFs
DC
Mono/Macro
Plasma

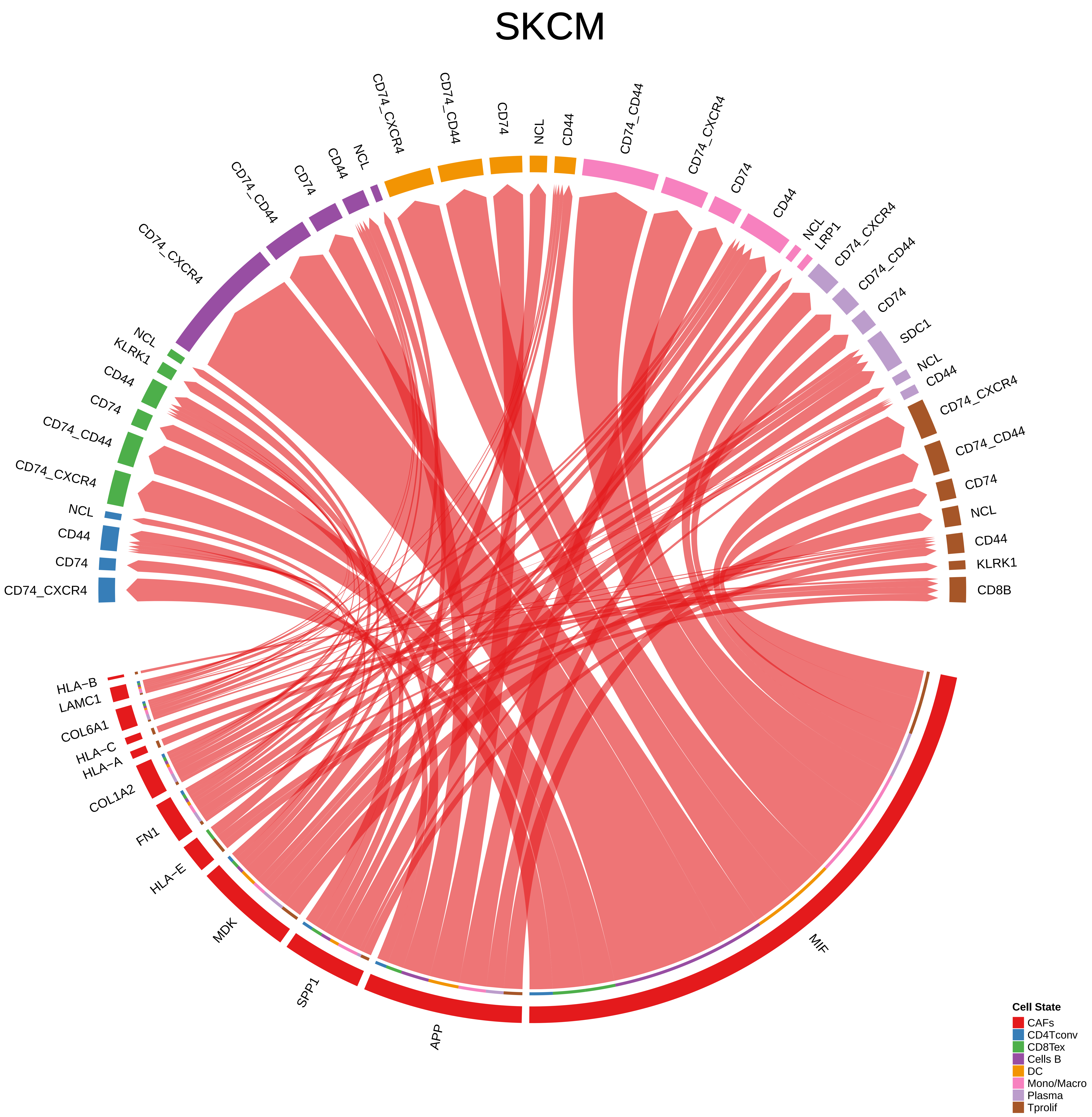
SKCM
CD74_CXCR4
CD74
CD44
NCL
CD74_CXCR4
CD74_CD44
CD74
CD44
KLRK1
NCL
CD74_CXCR4
CD74_CD44
CD74
CD44
NCL
CD74_CXCR4
CD74_CD44
CD74
NCL
CD44
CD74_CD44
CD74_CXCR4
CD74
CD44
NCL
LRP1
CD74_CXCR4
CD74_CD44
CD74
SDC1
NCL
CD44
CD74_CXCR4
CD74_CD44
CD74
NCL
CD44
KLRK1
CD8B
HLA−B
LAMC1
COL6A1
HLA−C
HLA−A
COL1A2
FN1
HLA−E
MDK
SPP1
APP
MIF
Cell State
CAFs
CD4Tconv
CD8Tex
Cells B
DC
Mono/Macro
Plasma
Tprolif

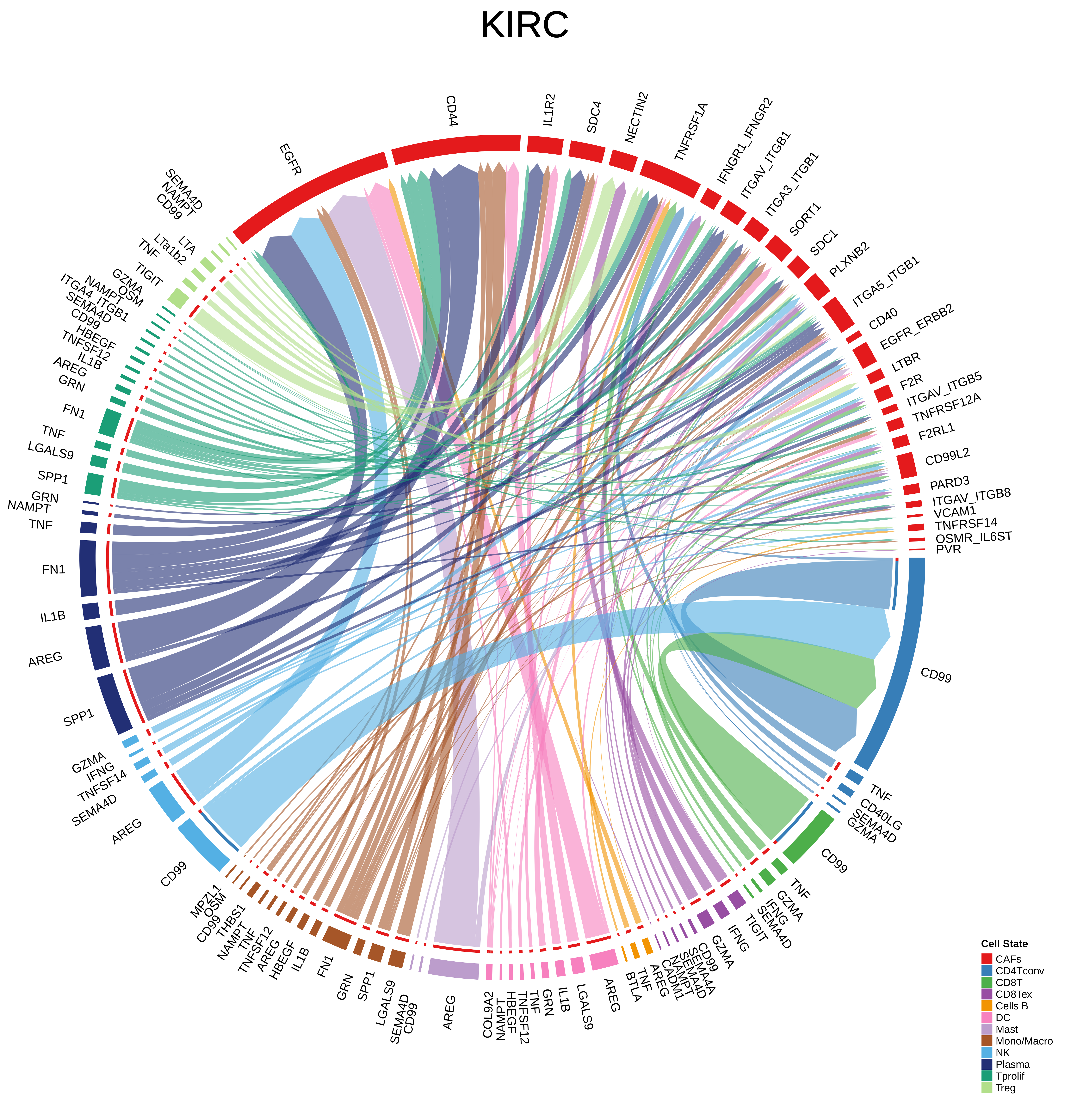
KIRC
CD44
IL1R2
SDC4
NECTIN2
TNFRSF1A
IFNGR1_IFNGR2
ITGAV_ITGB1
ITGA3_ITGB1
SORT1
SDC1
PLXNB2
ITGA5_ITGB1
CD40
EGFR_ERBB2
LTBR
F2R
ITGAV_ITGB5
TNFRSF12A
F2RL1
CD99L2
PARD3
ITGAV_ITGB8
VCAM1
TNFRSF14
OSMR_IL6ST
PVR
CD99
TNF
CD40LG
SEMA4D
GZMA
CD99
TNF
GZMA
IFNG
SEMA4D
TIGIT
IFNG
GZMA
CD99
SEMA4A
SEMA4D
NAMPT
CADM1
AREG
TNF
BTLA
AREG
LGALS9
IL1B
GRN
TNF
TNFSF12
HBEGF
NAMPT
COL9A2
AREG
CD99
SEMA4D
LGALS9
SPP1
GRN
FN1
IL1B
HBEGF
AREG
TNFSF12
TNF
NAMPT
THBS1
CD99
OSM
MPZL1
CD99
AREG
SEMA4D
TNFSF14
IFNG
GZMA
SPP1
AREG
IL1B
FN1
TNF
NAMPT
GRN
SPP1
LGALS9
TNF
FN1
GRN
AREG
IL1B
TNFSF12
HBEGF
CD99
SEMA4D
ITGA4_ITGB1
NAMPT
OSM
GZMA
TIGIT
TNF
LTa1b2
LTA
CD99
NAMPT
SEMA4D
EGFR
Cell State
CAFs
CD4Tconv
CD8T
CD8Tex
Cells B
DC
Mast
Mono/Macro
NK
Plasma
Tprolif
Treg

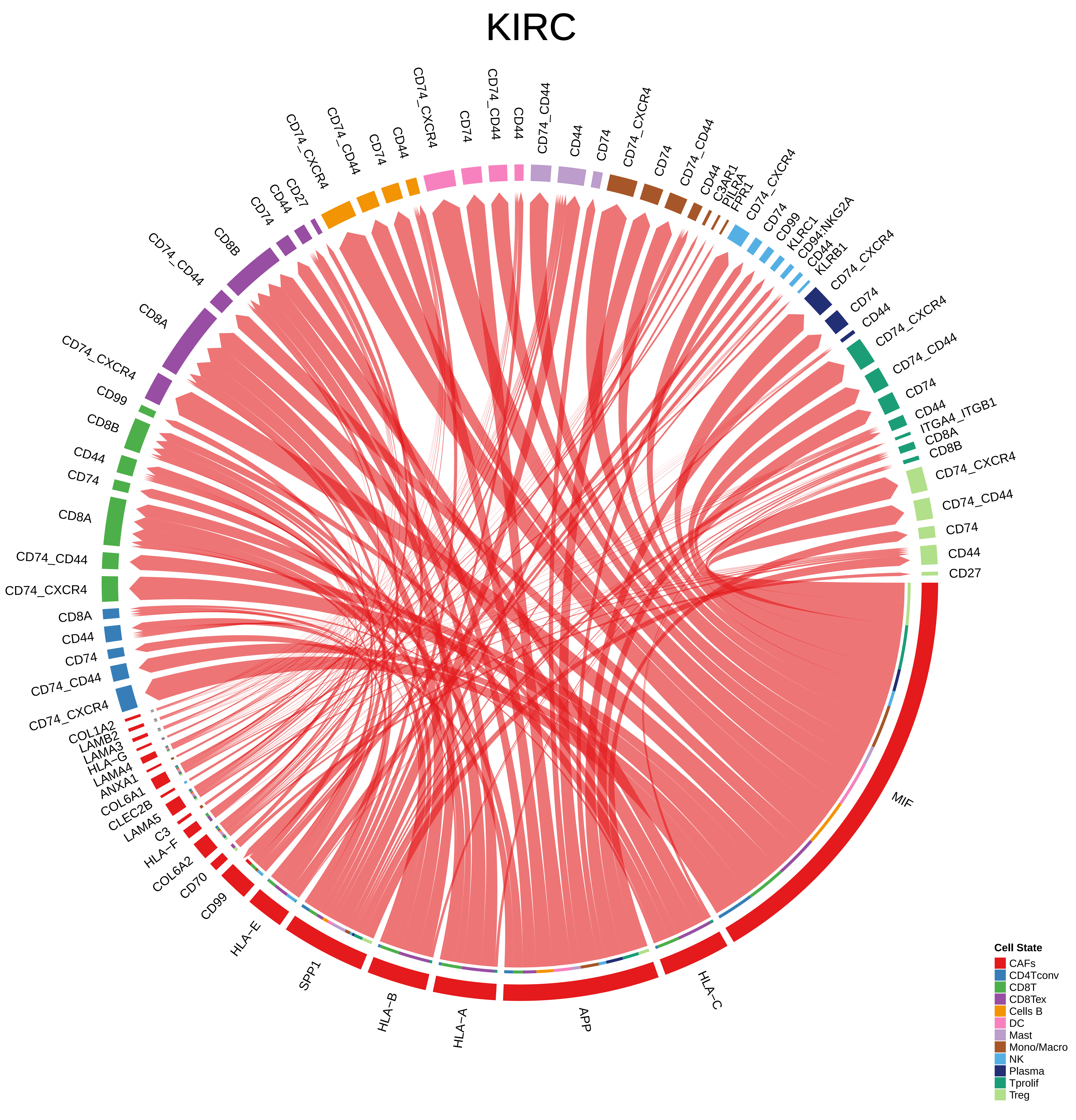

KIRC
MIF
APP
HLA-C
HLA-A
HLA-B
SPP1
HLA-E
CD99
CD70
COL6A2
HLA-F
C3
LAMA5
CLEC2B
COL6A1
ANXA1
LAMA4
HLA-G
LAMA3
LAMB2
COL1A2
CD74_CXCR4
CD74_CD44
CD74
CD44
CD8A
CD8B
CD99
CD27
C3AR1
PILRA
FPR1
KLRC1
CD94:NKG2A
KLRB1
ITGA4_ITGB1
Cell State
CAFs
CD4Tconv
CD8T
CD8Tex
Cells B
DC
Mast
Mono/Macro
NK
Plasma
Tprolif
Treg

NSCLC

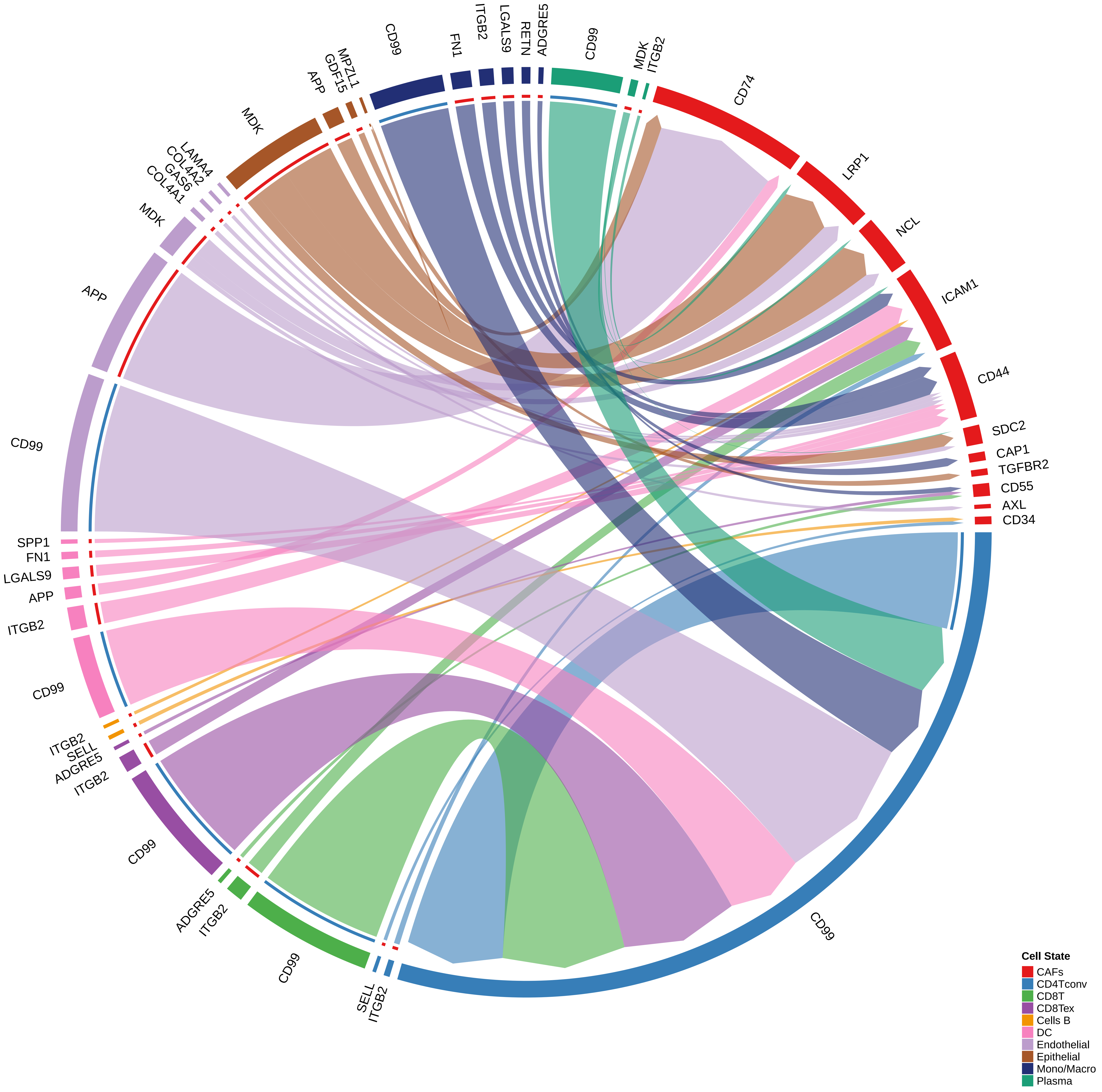
CD74
LRP1
NCL
ICAM1
CD44
SDC2
CAP1
TGFBR2
CD55
AXL
CD34
CD99
ITGB2
SELL
CD99
ITGB2
ADGRE5
CD99
ITGB2
ADGRE5
SELL
ITGB2
CD99
ITGB2
APP
LGALS9
FN1
SPP1
CD99
APP
MDK
COL4A1
GAS6
COL4A2
LAMA4
MDK
APP
GDF15
MPZL1
CD99
FN1
ITGB2
LGALS9
RETN
ADGRE5
CD99
MDK
ITGB2
Cell State
CAFs
CD4Tconv
CD8T
CD8Tex
Cells B
DC
Endothelial
Epithelial
Mono/Macro
Plasma

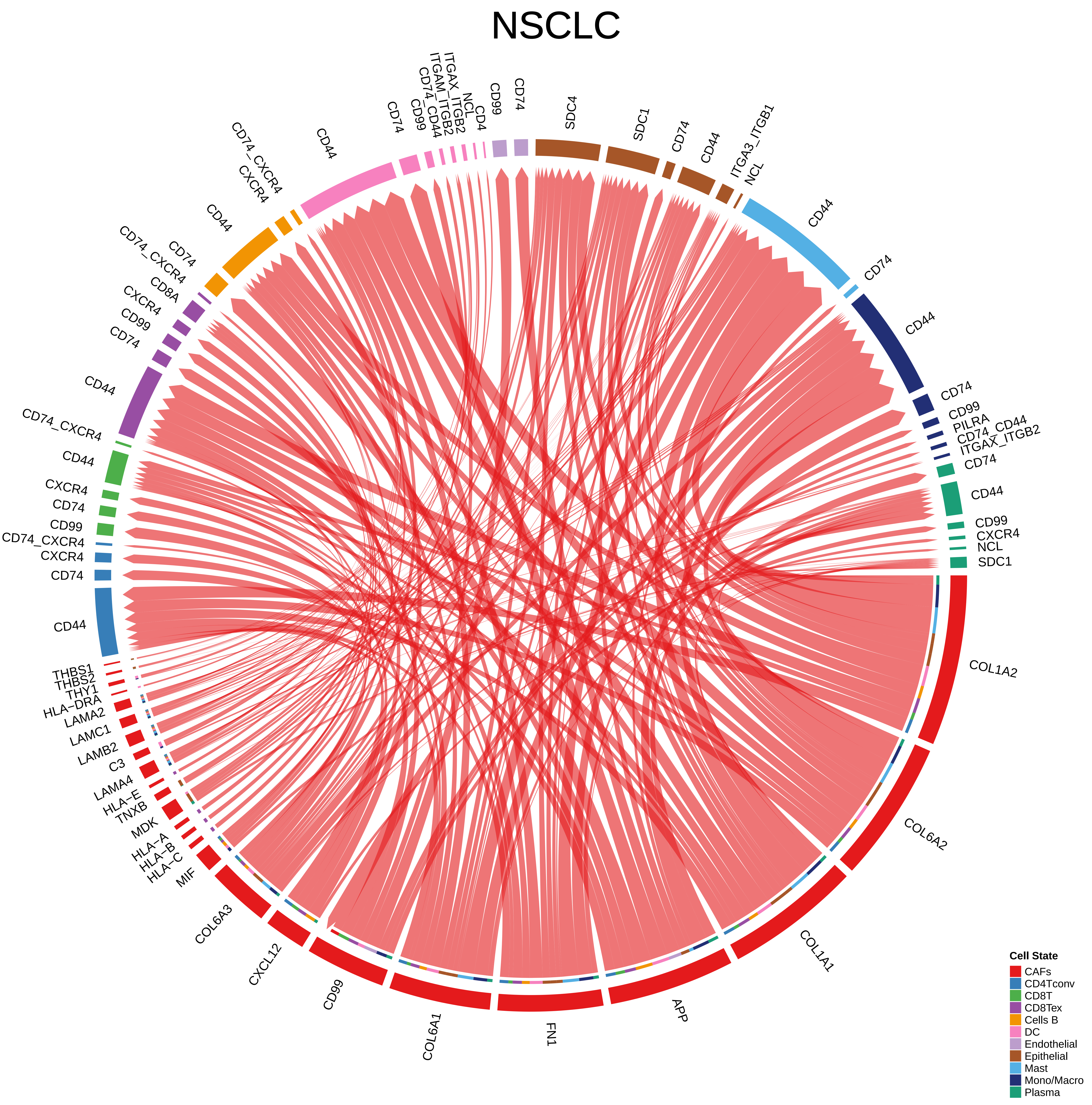
NSCLC
SDC4
SDC1
CD74
CD44
ITGA3_ITGB1
NCL
CD44
CD74
CD44
CD74
CD99
PILRA
CD74_CD44
ITGAX_ITGB2
CD74
CD44
CD99
CXCR4
NCL
SDC1
COL1A2
COL6A2
COL1A1
APP
FN1
COL6A1
CD99
CXCL12
COL6A3
MIF
HLA−C
HLA−B
HLA−A
MDK
TNXB
HLA−E
LAMA4
C3
LAMB2
LAMC1
LAMA2
HLA−DRA
THY1
THBS2
THBS1
CD44
CD74
CXCR4
CD74_CXCR4
CD99
CD74
CXCR4
CD44
CD74_CXCR4
CD44
CD74
CD99
CXCR4
CD8A
CD74_CXCR4
CD74
CD44
CXCR4
CD74_CXCR4
CD44
CD74
CD99
CD74_CD44
ITGAM_ITGB2
ITGAX_ITGB2
NCL
CD4
CD99
CD74
Cell State
CAFs
CD4Tconv
CD8T
CD8Tex
Cells B
DC
Endothelial
Epithelial
Mast
Mono/Macro
Plasma

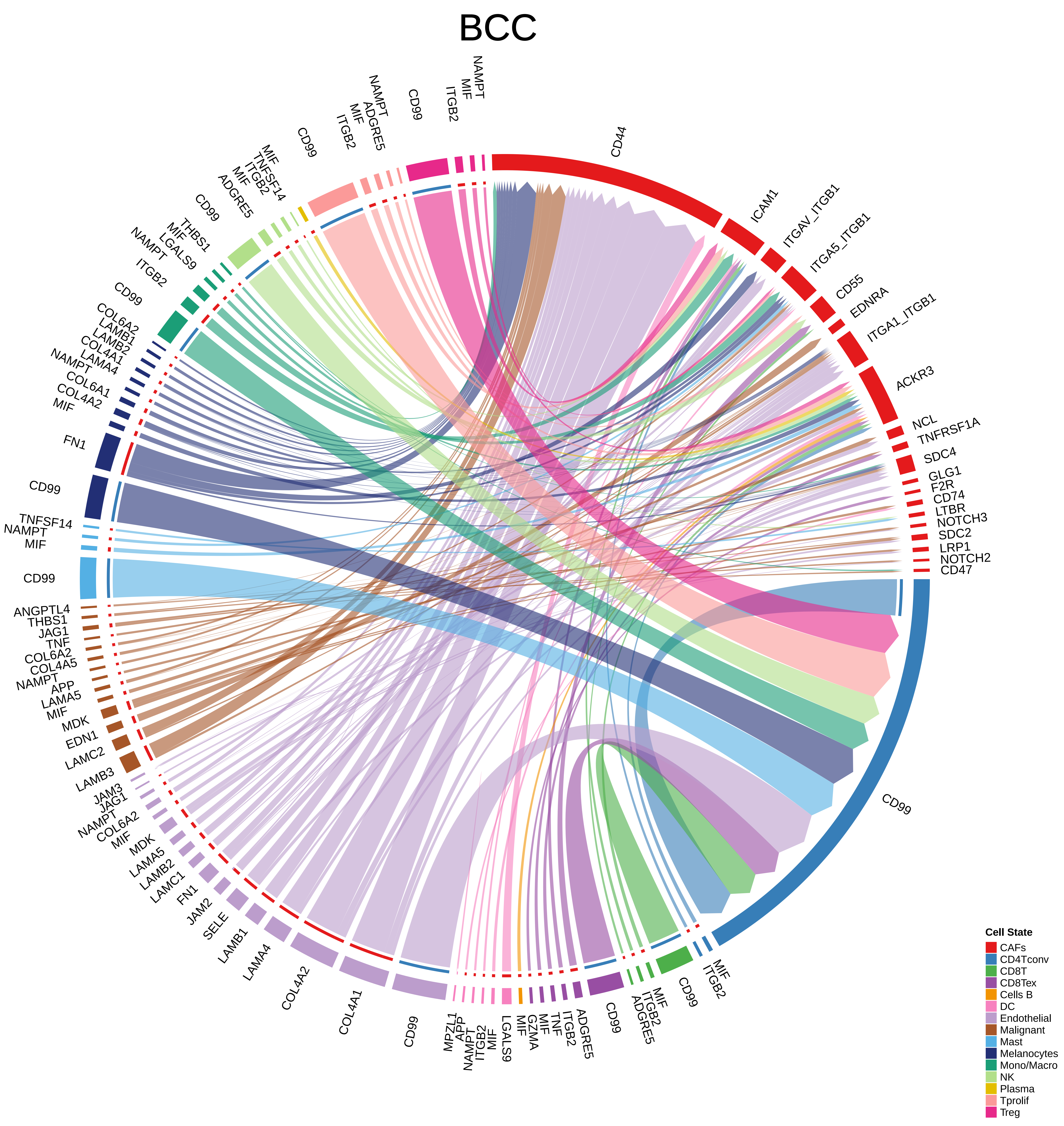
BCC
NAMPT
MIF
ITGB2
CD99
NAMPT
ADGRE5
MIF
ITGB2
CD99
MIF
TNFSF14
ITGB2
MIF
ADGRE5
CD99
THBS1
MIF
LGALS9
NAMPT
ITGB2
CD99
COL6A2
LAMB1
LAMB2
COL4A1
LAMA4
NAMPT
COL6A1
COL4A2
MIF
FN1
CD99
TNFSF14
NAMPT
MIF
CD99
ANGPTL4
THBS1
JAG1
TNF
COL6A2
COL4A5
NAMPT
APP
LAMA5
MIF
MDK
EDN1
LAMC2
LAMB3
JAM3
JAG1
NAMPT
COL6A2
MIF
MDK
LAMA5
LAMB2
LAMC1
FN1
JAM2
SELE
LAMB1
LAMA4
COL4A2
COL4A1
CD99
MPZL1
APP
NAMPT
ITGB2
MIF
LGALS9
MIF
GZMA
MIF
TNF
ITGB2
ADGRE5
CD99
ADGRE5
ITGB2
MIF
CD99
ITGB2
MIF
CD99
CD47
NOTCH2
LRP1
SDC2
NOTCH3
LTBR
CD74
F2R
GLG1
SDC4
TNFRSF1A
NCL
ACKR3
ITGA1_ITGB1
EDNRA
CD55
ITGA5_ITGB1
ITGAV_ITGB1
ICAM1
CD44
Cell State
CAFs
CD4Tconv
CD8T
CD8Tex
Cells B
DC
Endothelial
Malignant
Mast
Melanocytes
Mono/Macro
NK
Plasma
Tprolif
Treg

BCC

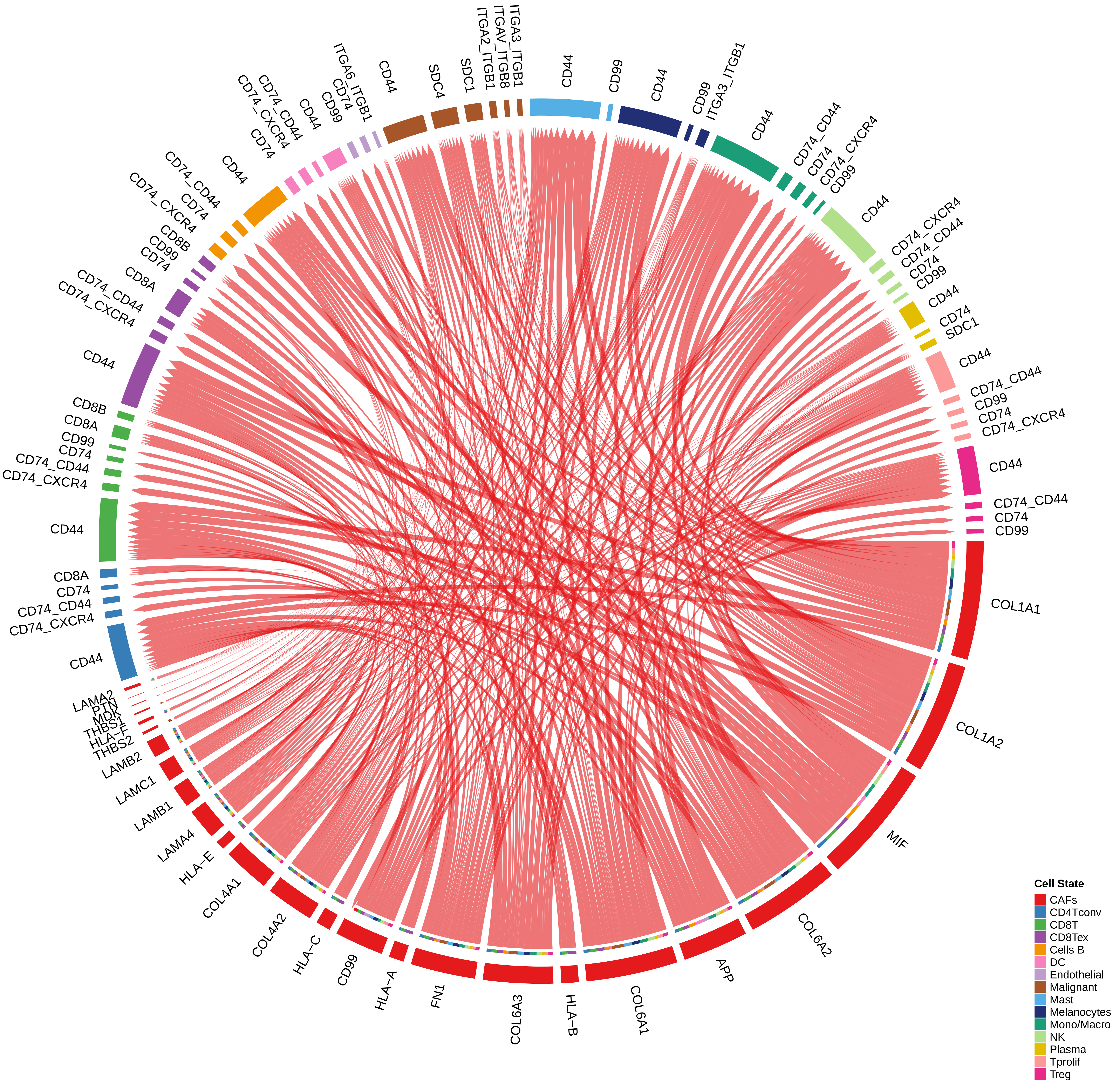

CD44
CD99
CD44
CD99
ITGA3_ITGB1
CD44
CD74_CD44
CD74
CD74_CXCR4
CD99
CD44
CD74_CXCR4
CD74_CD44
CD74
CD99
CD44
CD74
SDC1
CD44
CD74_CD44
CD99
CD74
CD74_CXCR4
CD44
CD74_CD44
CD74
CD99
COL1A1
COL1A2
MIF
COL6A2
APP
COL6A1
HLA−B
COL6A3
FN1
HLA−A
CD99
HLA−C
COL4A2
COL4A1
HLA−E
LAMA4
LAMB1
LAMC1
LAMB2
THBS2
HLA−F
THBS1
MDK
PTN
LAMA2
CD44
CD74_CXCR4
CD74_CD44
CD74
CD8A
CD44
CD74_CXCR4
CD74_CD44
CD74
CD99
CD8A
CD8B
CD44
CD74_CXCR4
CD74_CD44
CD8A
CD74
CD99
CD8B
CD74_CXCR4
CD74
CD74_CD44
CD44
CD74
CD74_CXCR4
CD74_CD44
CD44
CD99
CD74
ITGA6_ITGB1
CD44
SDC4
SDC1
ITGA2_ITGB1
ITGAV_ITGB8
ITGA3_ITGB1
Cell State
CAFs
CD4Tconv
CD8T
CD8Tex
Cells B
DC
Endothelial
Malignant
Mast
Melanocytes
Mono/Macro
NK
Plasma
Tprolif
Treg

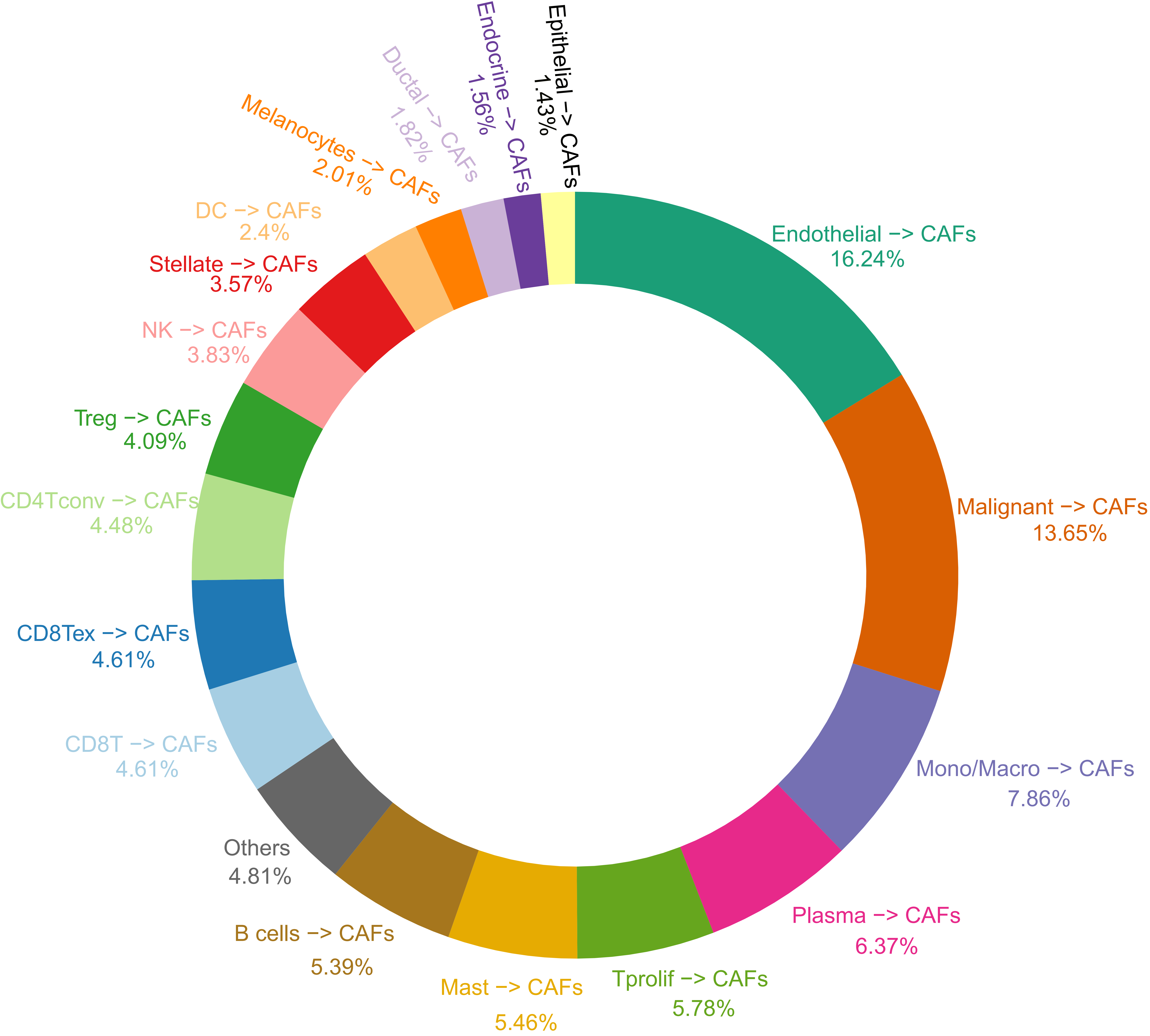

Endothelial -> CAFs
16.24%
Malignant -> CAFs
13.65%
Mono/Macro -> CAFs
7.86%
Plasma -> CAFs
6.37%
Tprolif -> CAFs
5.78%
Mast -> CAFs
5.46%
B cells -> CAFs
5.39%
Others
4.81%
CD8T -> CAFs
4.61%
CD8Tex -> CAFs
4.61%
CD4Tconv -> CAFs
4.48%
Treg -> CAFs
4.09%
NK -> CAFs
3.83%
Stellate -> CAFs
3.57%
DC -> CAFs
2.4%
Melanocytes -> CAFs
2.01%
Ductal -> CAFs
1.82%
Endocrine -> CAFs
1.56%
Epithelial -> CAFs
1.43%

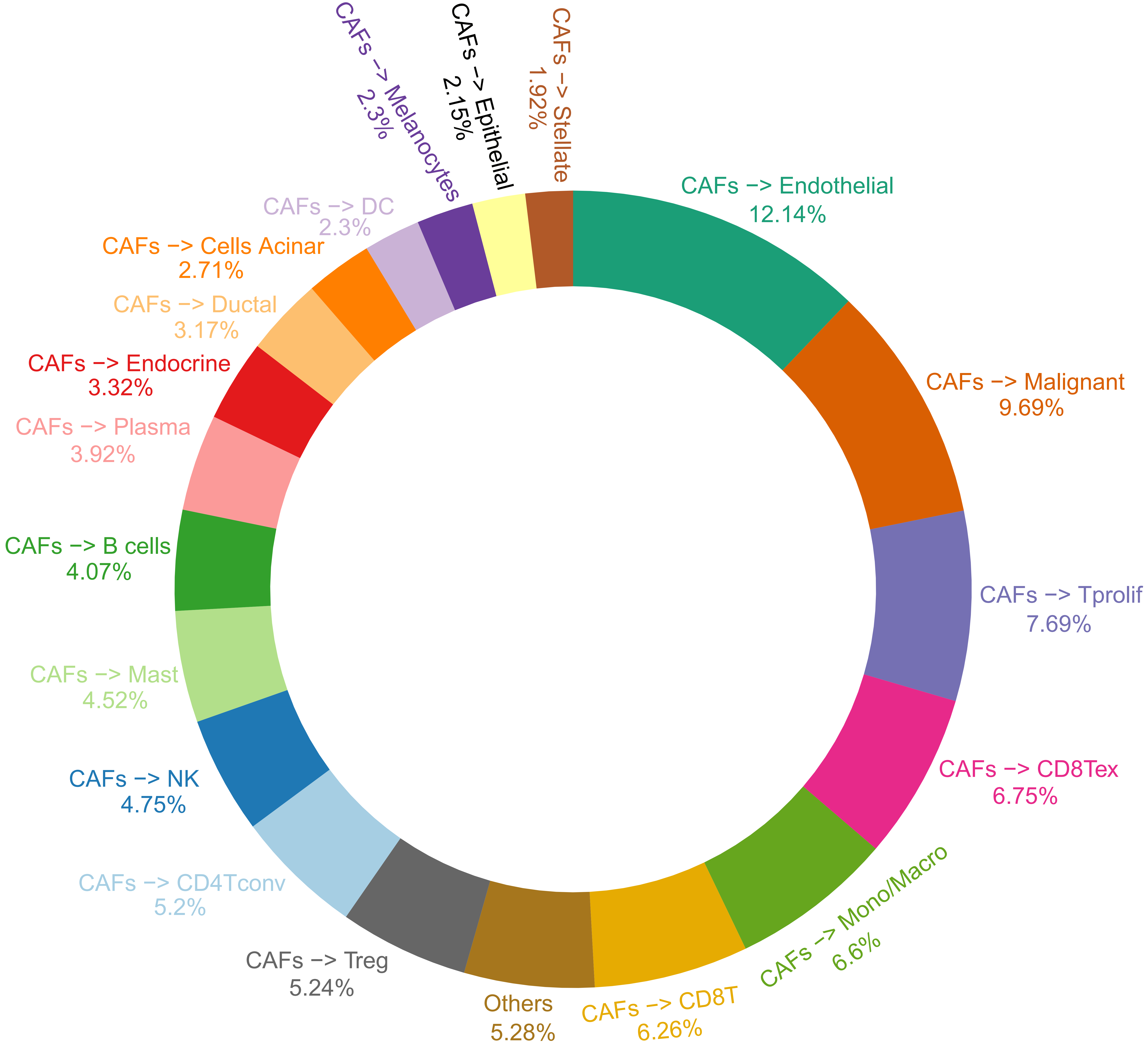
CAFs -> Endothelial
12.14%
CAFs -> Malignant
9.69%
CAFs -> Tprolif
7.69%
CAFs -> CD8Tex
6.75%
CAFs -> Mono/Macro
6.6%
CAFs -> CD8T
6.26%
Others
5.28%
CAFs -> Treg
5.24%
CAFs -> CD4Tconv
5.2%
CAFs -> NK
4.75%
CAFs -> Mast
4.52%
CAFs -> B cells
4.07%
CAFs -> Plasma
3.92%
CAFs -> Endocrine
3.32%
CAFs -> Ductal
3.17%
CAFs -> Cells Acinar
2.71%
CAFs -> DC
2.3%
CAFs -> Melanocytes
2.3%
CAFs -> Epithelial
2.15%
CAFs -> Stellate
1.92%
