# Supplementary material for: Dissecting the role of cancer‐associated fibroblast‐derived biglycan as a potential therapeutic target in immunotherapy resistance: A tumor bulk and single‐cell transcriptomic study
Source: Clin Transl Med. 2023 Feb 11;13(2):e1189. doi: 10.1002/ctm2.1189 (PMC9920016; doi:10.1002/ctm2.1189)
Supplement: Supplementary file 7 — Supporting Information [file CTM2-13-e1189-s008.pdf]

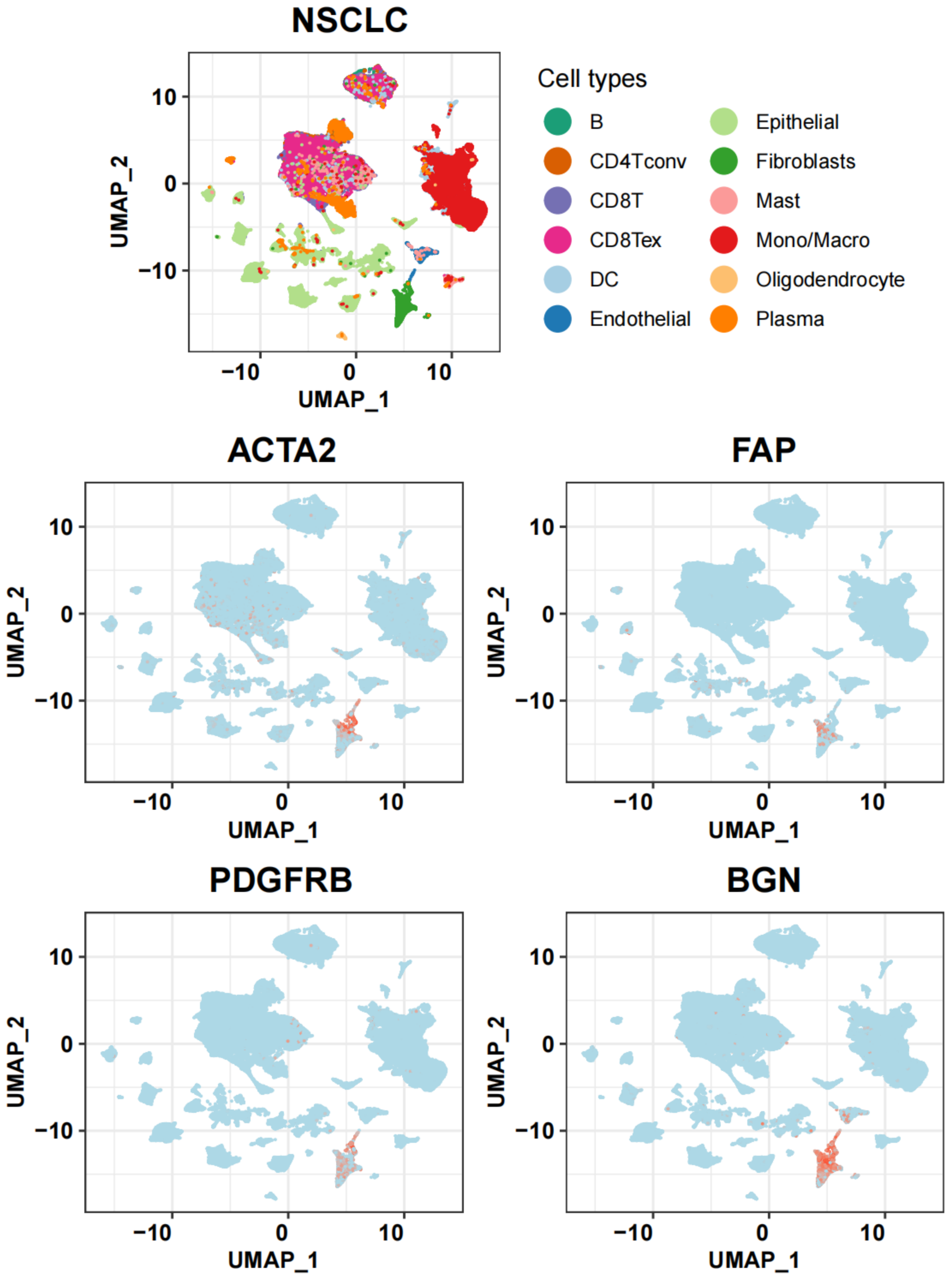

NSCLC
Cell types
B
CD4Tconv
CD8T
CD8Tex
DC
Endothelial
Epithelial
Fibroblasts
Mast
Mono/Macro
Oligodendrocyte
Plasma
UMAP_1
UMAP_2
ACTA2
FAP
PDGFRB
BGN
10
0
−10

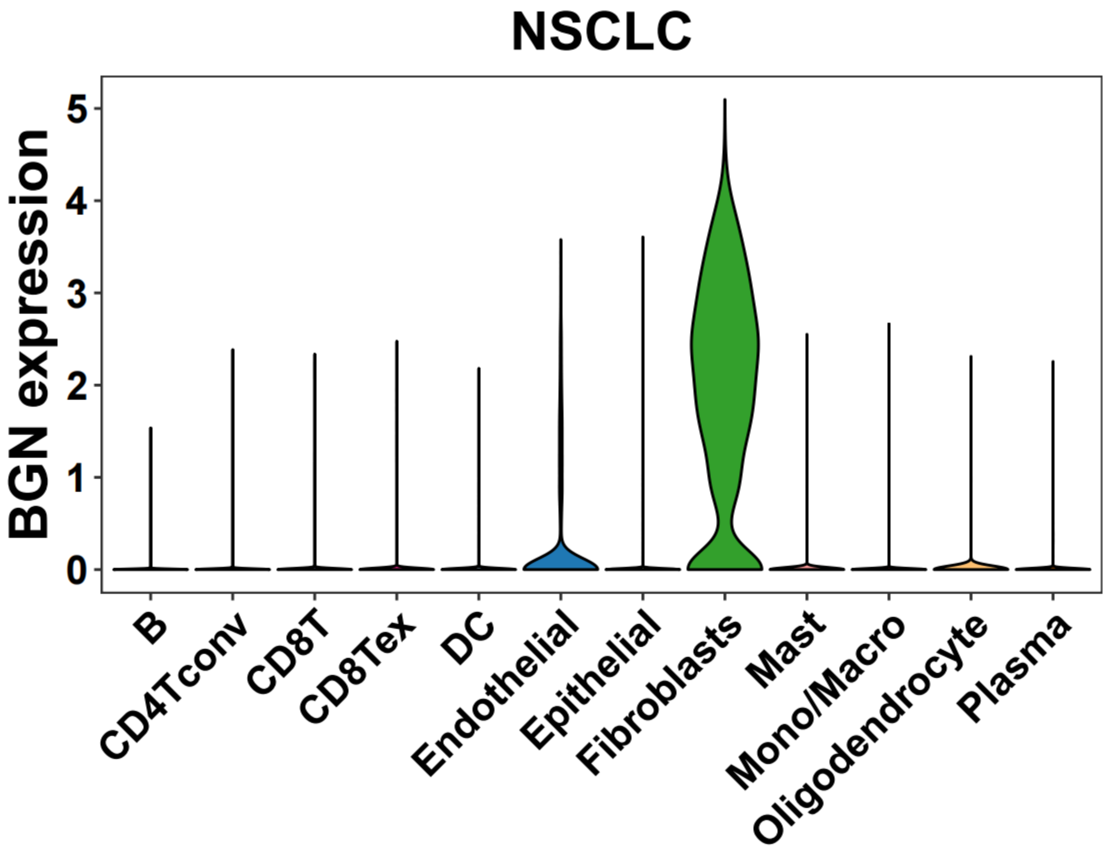

NSCLC
BGN expression
0
1
2
3
4
5
B
CD4Tconv
CD8T
CD8Tex
DC
Endothelial
Epithelial
Fibroblasts
Mast
Mono/Macro
Oligodendrocyte
Plasma

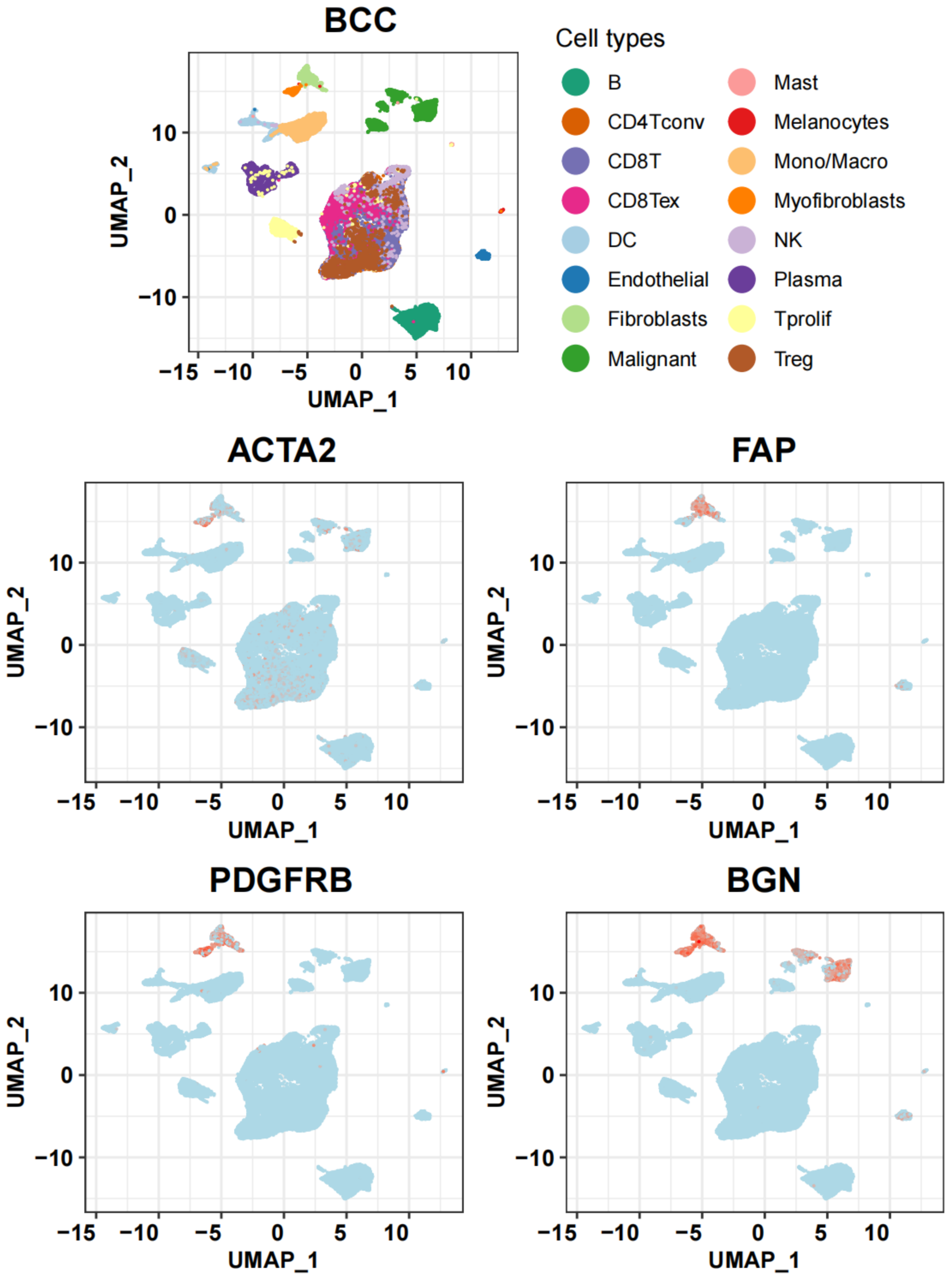
BCC
Cell types
B
CD4Tconv
CD8T
CD8Tex
DC
Endothelial
Fibroblasts
Malignant
Mast
Melanocytes
Mono/Macro
Myofibroblasts
NK
Plasma
Tprolif
Treg
UMAP_1
UMAP_2
ACTA2
FAP
PDGFRB
BGN

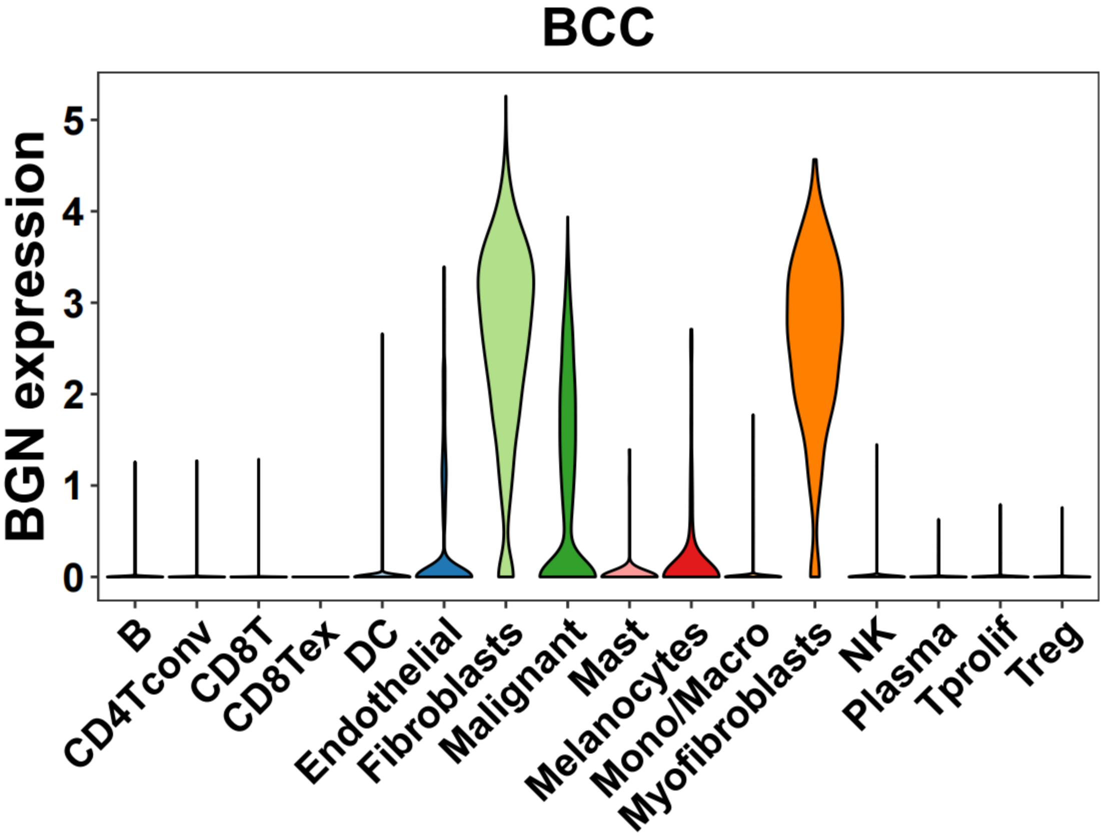
BCC
BGN expression
0
1
2
3
4
5
B
CD4Tconv
CD8T
CD8Tex
DC
Endothelial
Fibroblasts
Malignant
Mast
Melanocytes
Mono/Macro
Myofibroblasts
NK
Plasma
Tprolif
Treg

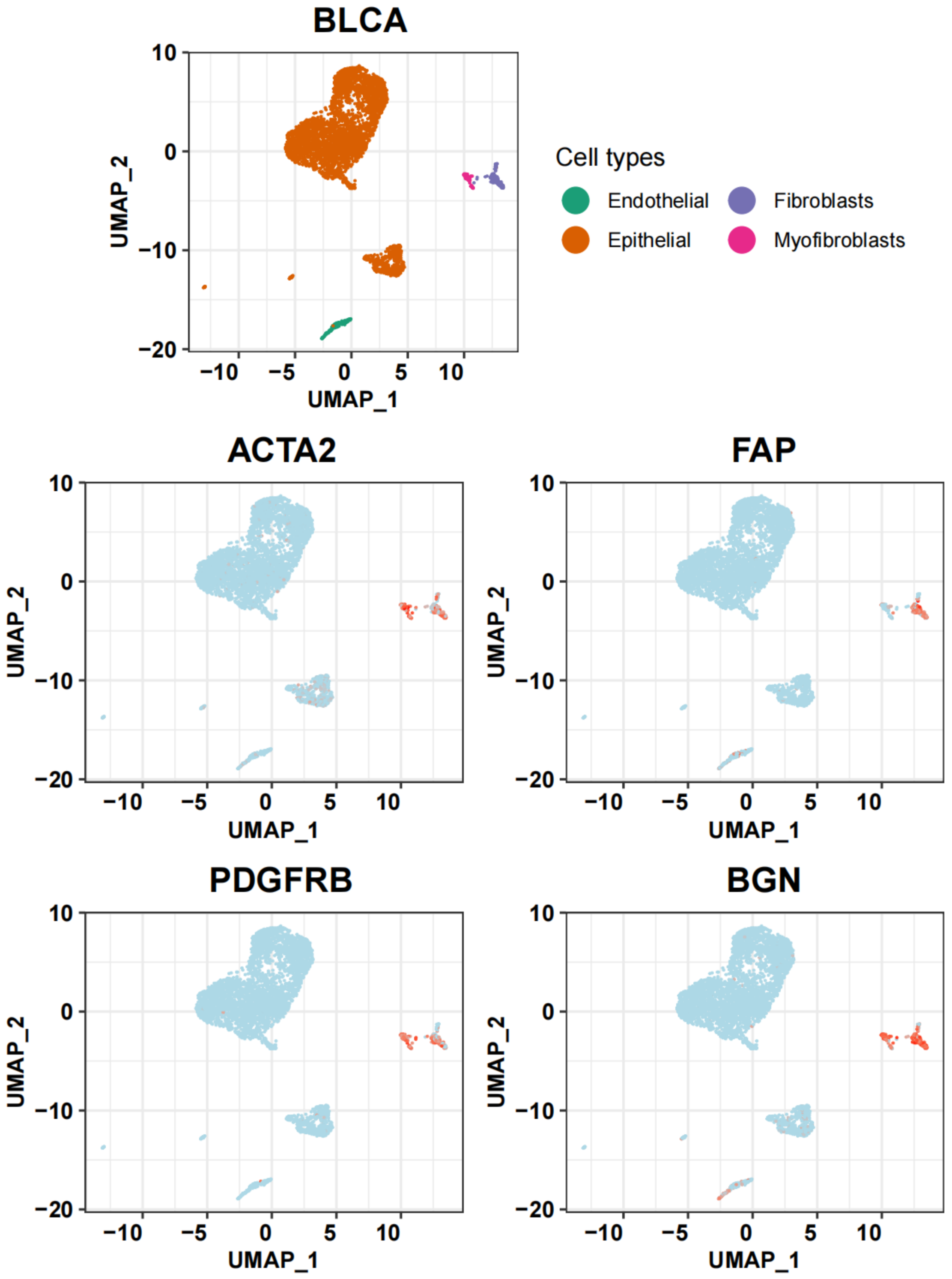
BLCA
Cell types
Endothelial
Epithelial
Fibroblasts
Myofibroblasts
ACTA2
FAP
PDGFRB
BGN
UMAP_1
UMAP_2

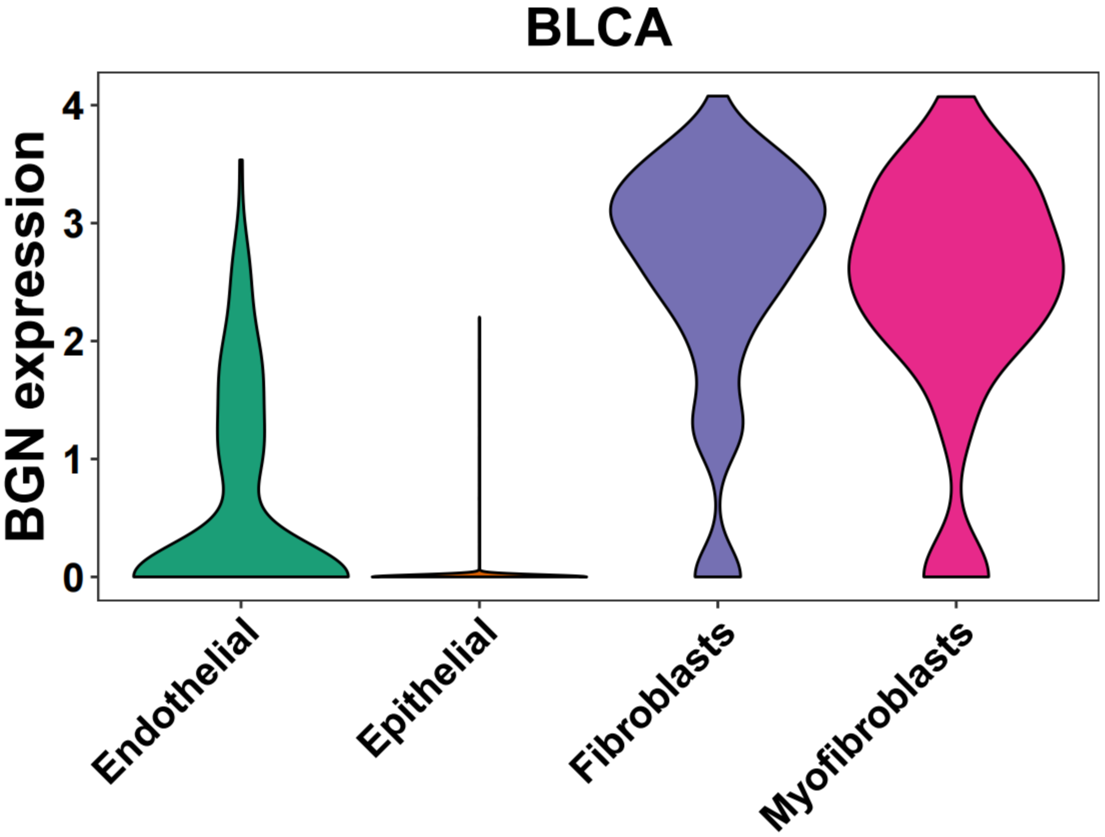

BLCA
BGN expression
0
1
2
3
4
Endothelial
Epithelial
Fibroblasts
Myofibroblasts

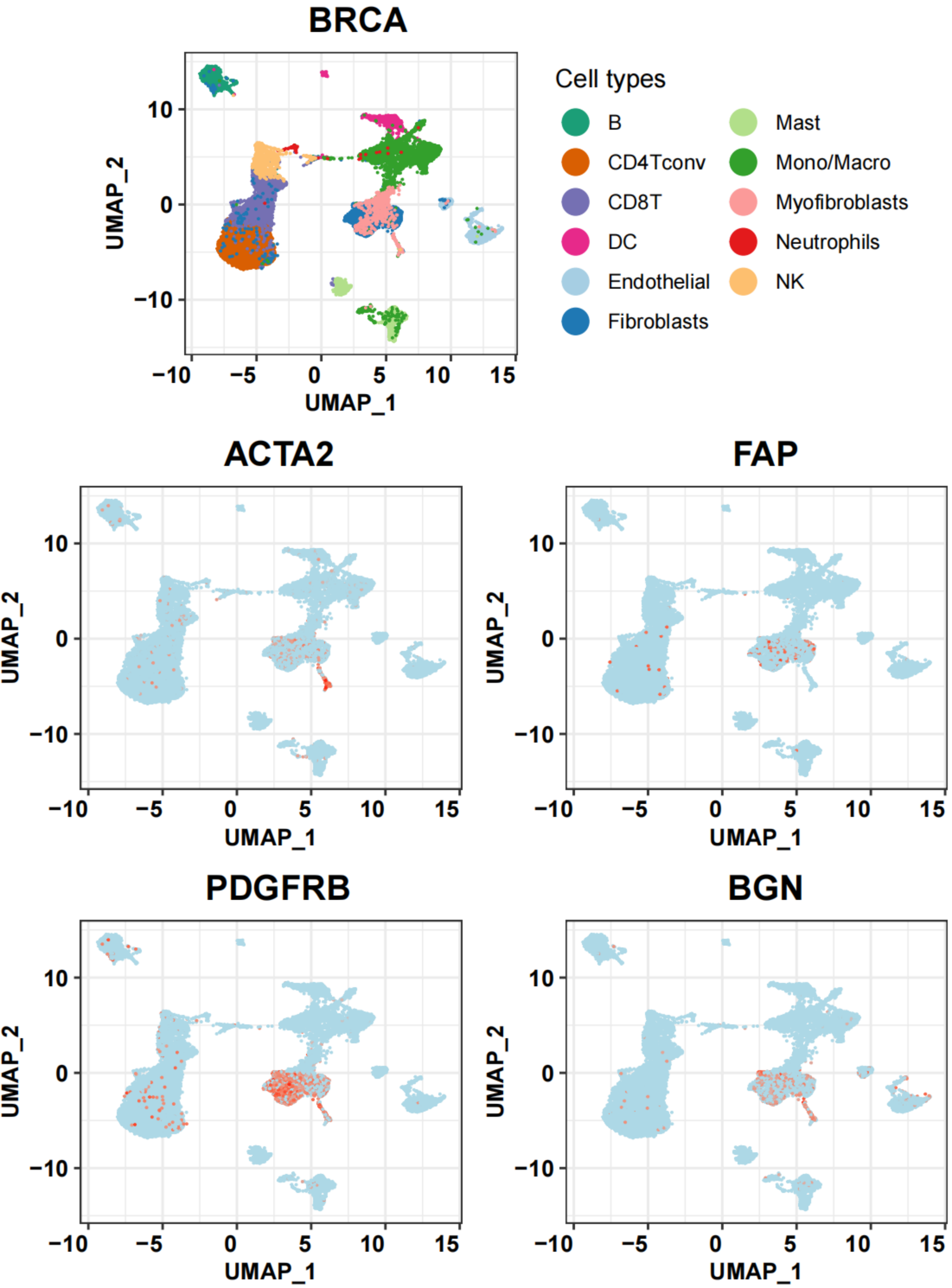
BRCA
Cell types
B
CD4Tconv
CD8T
DC
Endothelial
Fibroblasts
Mast
Mono/Macro
Myofibroblasts
Neutrophils
NK
UMAP_1
UMAP_2
ACTA2
FAP
PDGFRB
BGN

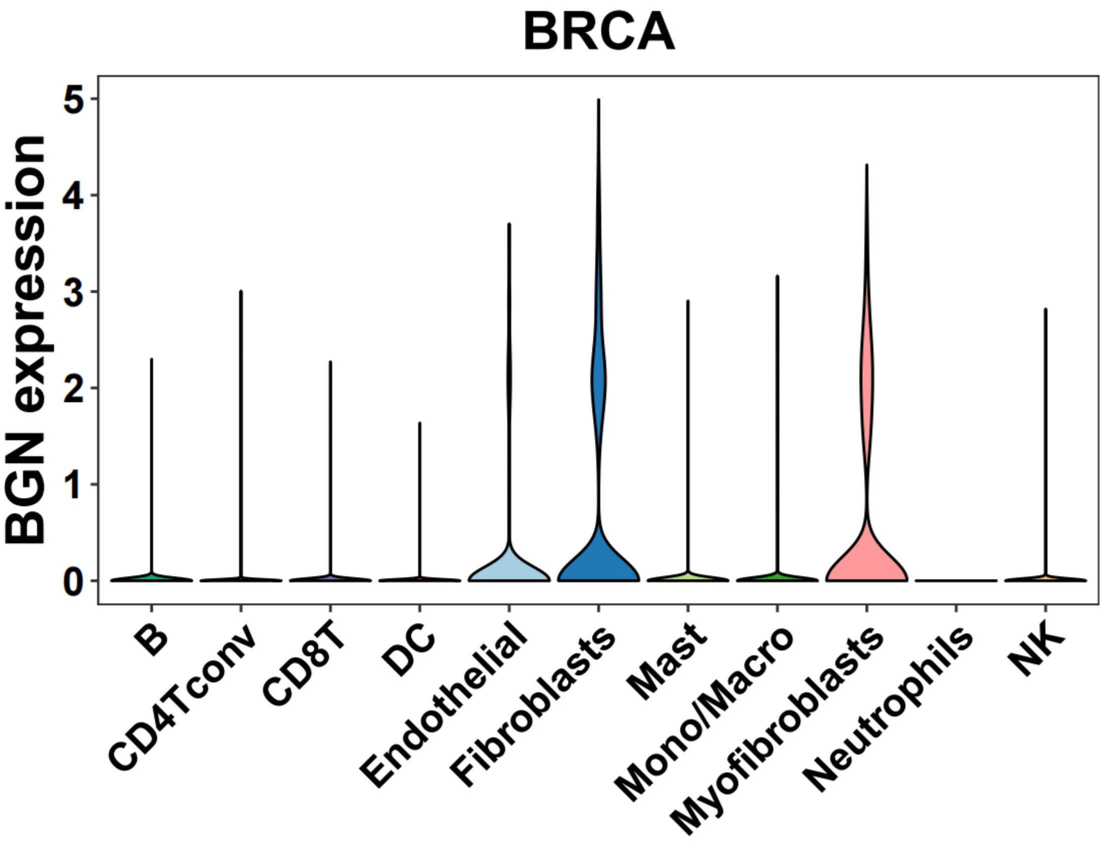
BRCA
BGN expression
0
1
2
3
4
5
B
CD4Tconv
CD8T
DC
Endothelial
Fibroblasts
Mast
Mono/Macro
Myofibroblasts
Neutrophils
NK

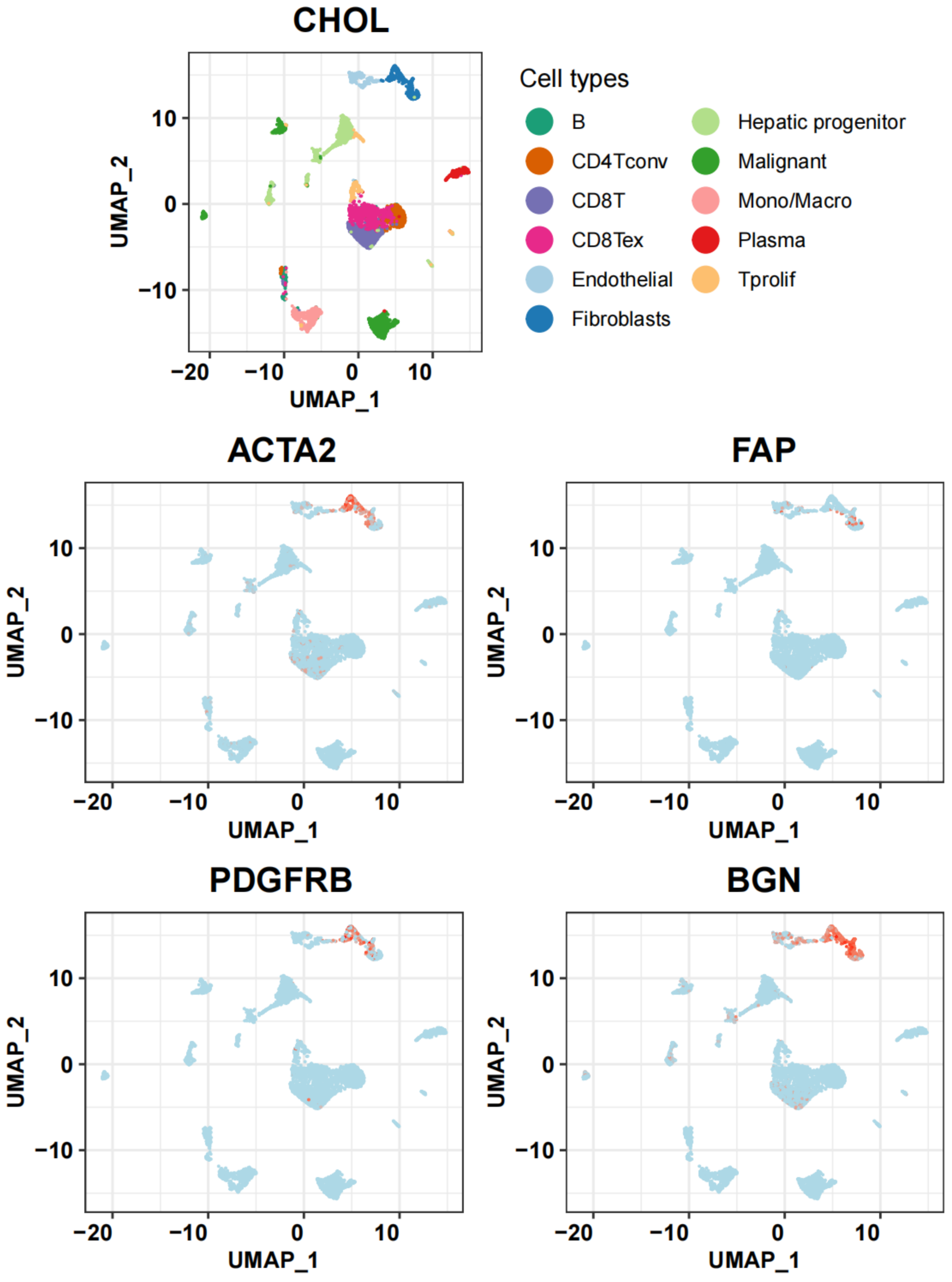

CHOL
Cell types
B
CD4Tconv
CD8T
CD8Tex
Endothelial
Fibroblasts
Hepatic progenitor
Malignant
Mono/Macro
Plasma
Tprolif
ACTA2
FAP
PDGFRB
BGN
UMAP_1
UMAP_2

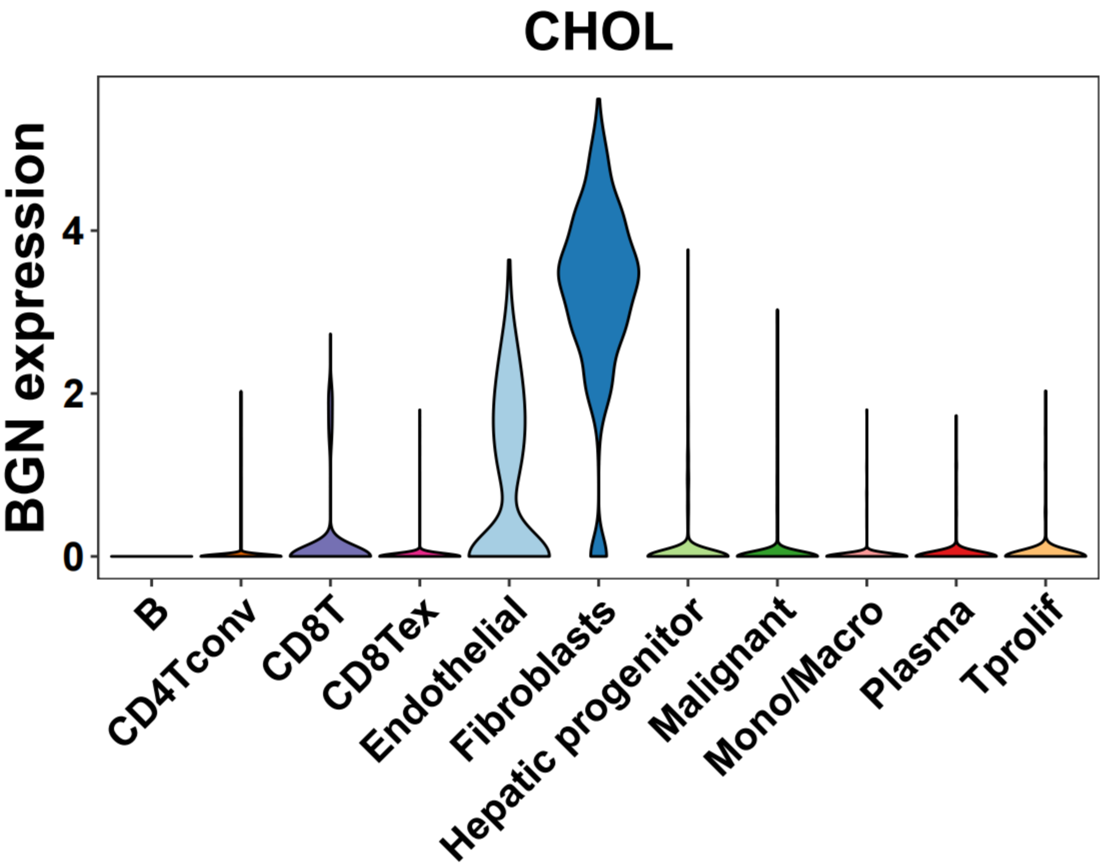

CHOL
BGN expression
0
2
4
B
CD4Tconv
CD8T
CD8Tex
Endothelial
Fibroblasts
Hepatic progenitor
Malignant
Mono/Macro
Plasma
Tprolif

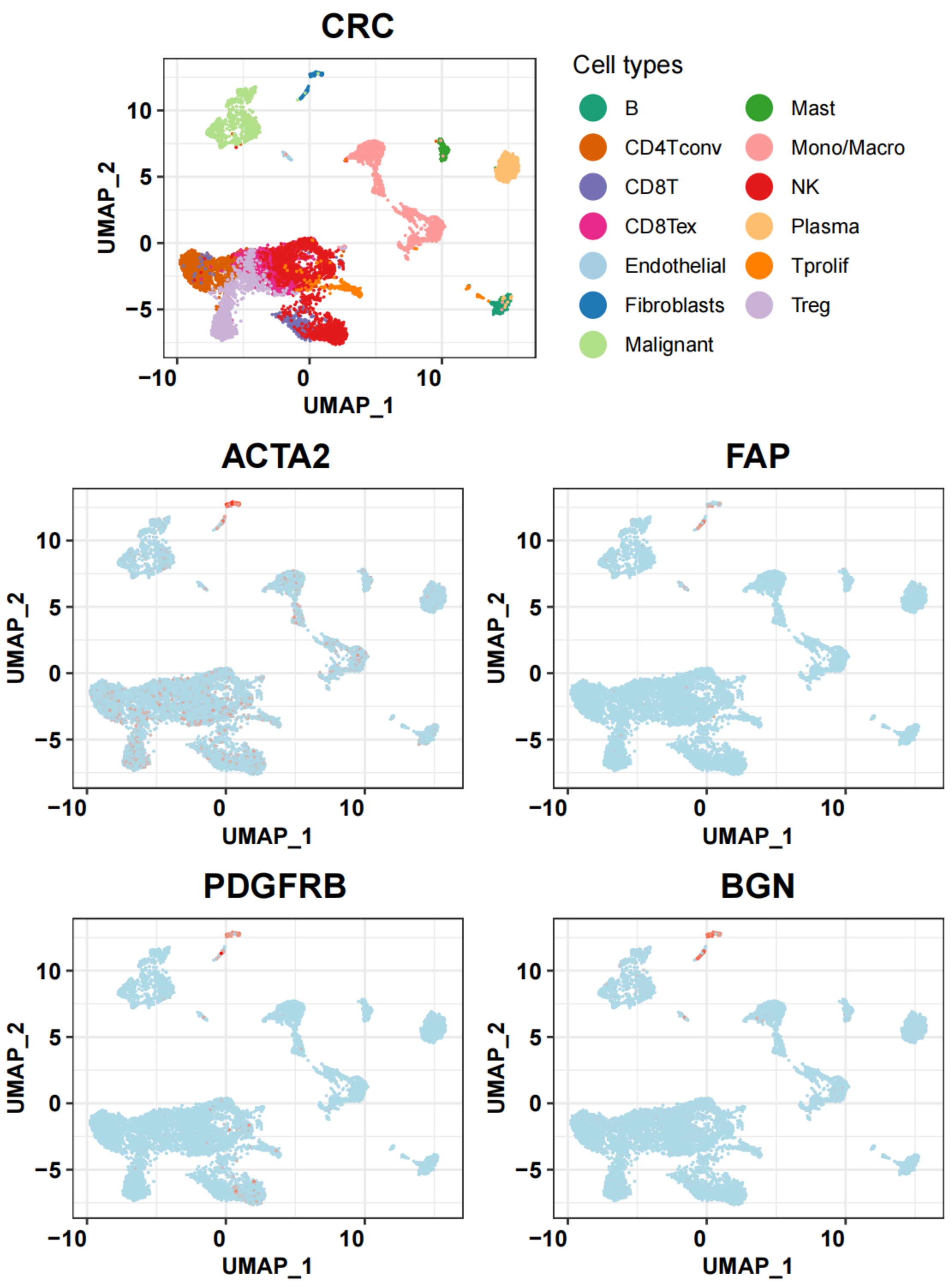
CRC
Cell types
B
CD4Tconv
CD8T
CD8Tex
Endothelial
Fibroblasts
Malignant
Mast
Mono/Macro
NK
Plasma
Tprolif
Treg
UMAP_1
UMAP_2
ACTA2
FAP
PDGFRB
BGN

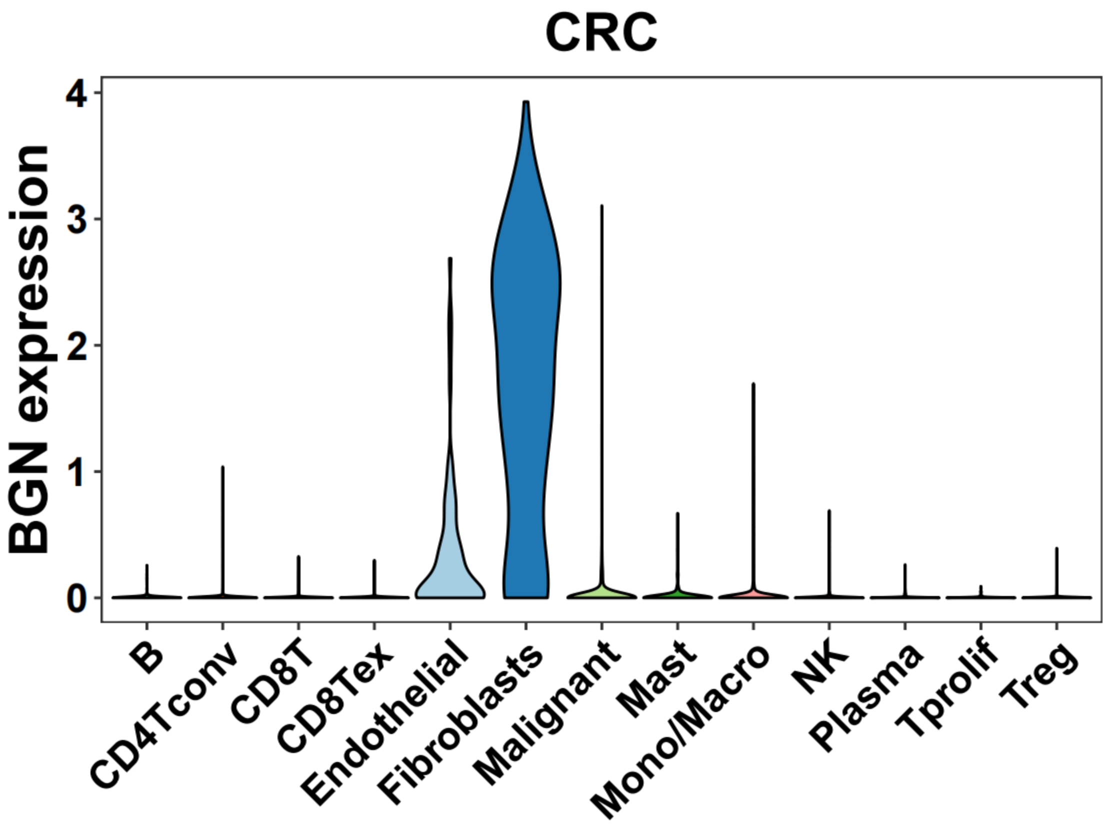

CRC
BGN expression
4
3
2
1
0
B
CD4Tconv
CD8T
CD8Tex
Endothelial
Fibroblasts
Malignant
Mast
Mono/Macro
NK
Plasma
Tprolif
Treg

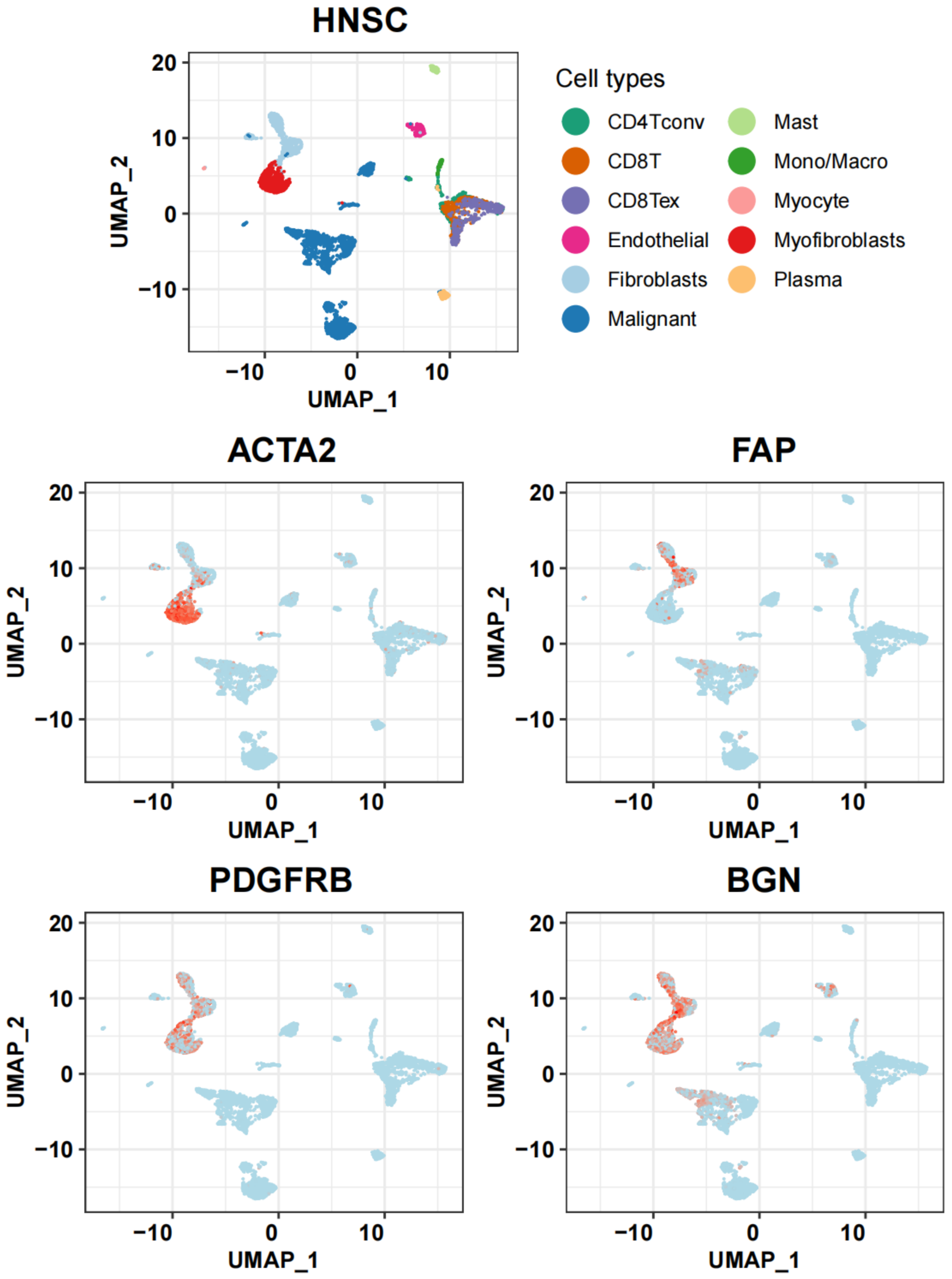
HNSC
Cell types
CD4Tconv
CD8T
CD8Tex
Endothelial
Fibroblasts
Malignant
Mast
Mono/Macro
Myocyte
Myofibroblasts
Plasma
ACTA2
FAP
PDGFRB
BGN
UMAP_1
UMAP_2

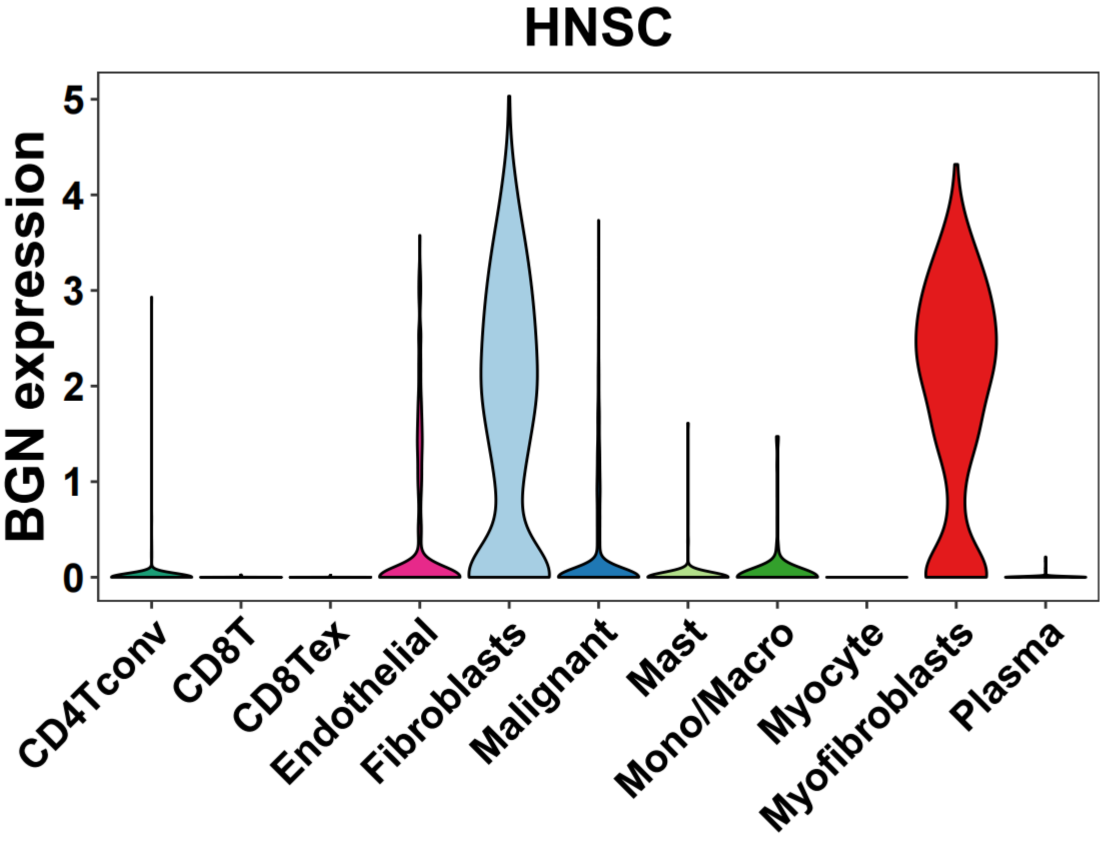

HNSC
BGN expression
0
1
2
3
4
5
CD4Tconv
CD8T
CD8Tex
Endothelial
Fibroblasts
Malignant
Mast
Mono/Macro
Myocyte
Myofibroblasts
Plasma

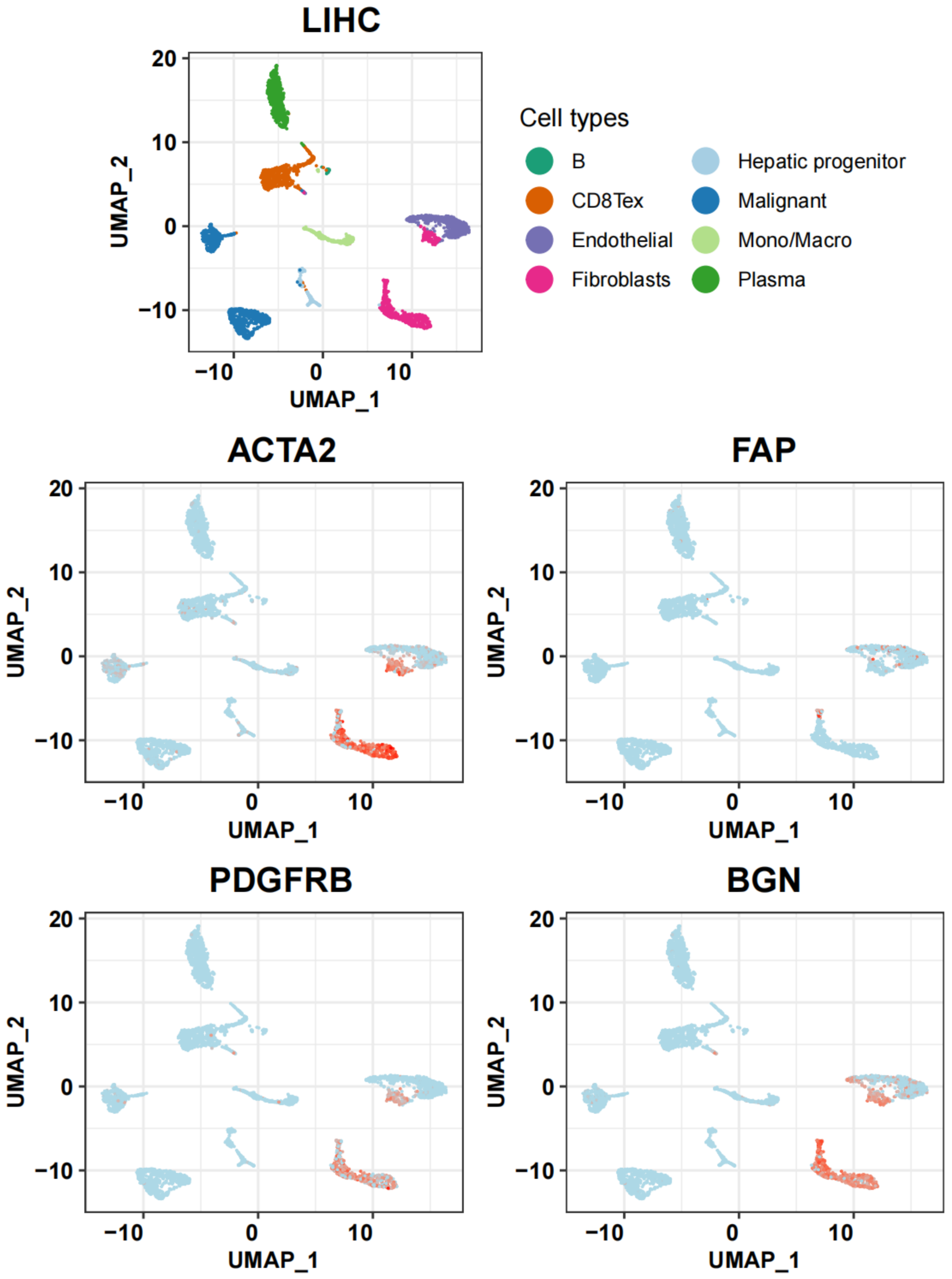

LIHC
Cell types
B
CD8Tex
Endothelial
Fibroblasts
Hepatic progenitor
Malignant
Mono/Macro
Plasma
UMAP_1
UMAP_2
ACTA2
FAP
PDGFRB
BGN

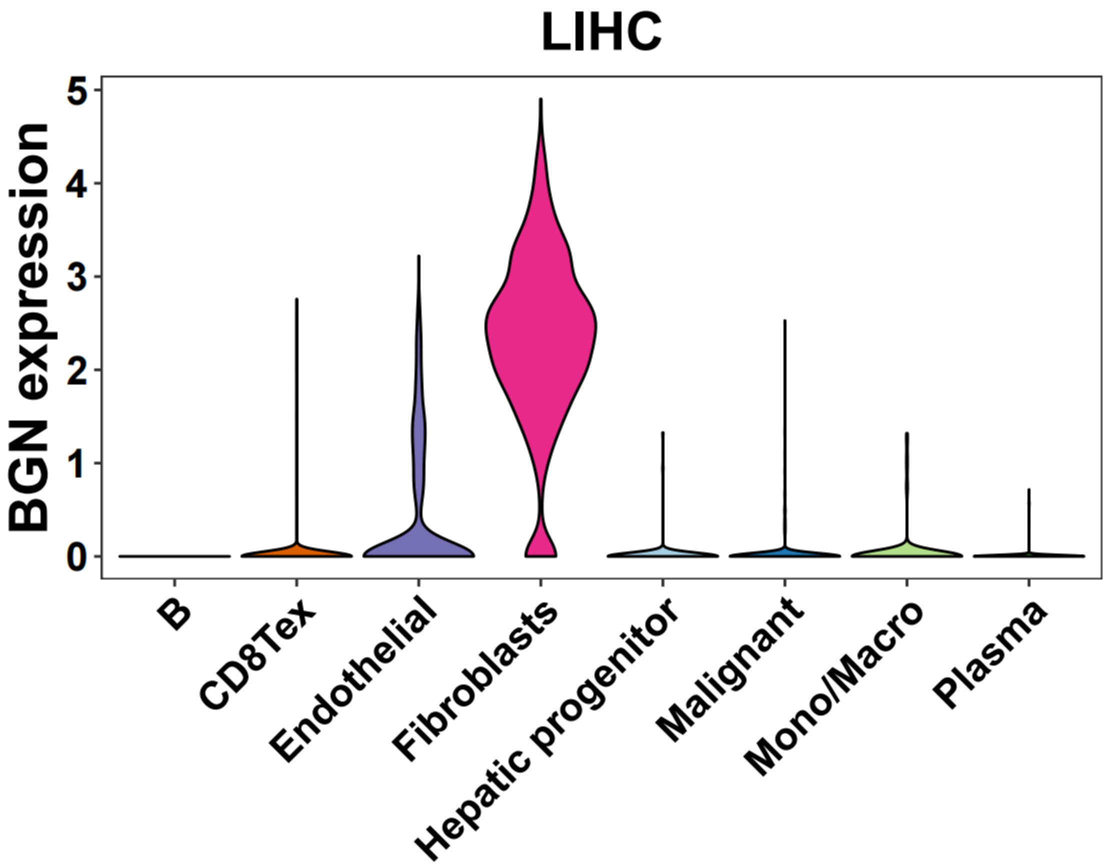
LIHC
BGN expression
0
1
2
3
4
5
B
CD8Tex
Endothelial
Fibroblasts
Hepatic progenitor
Malignant
Mono/Macro
Plasma

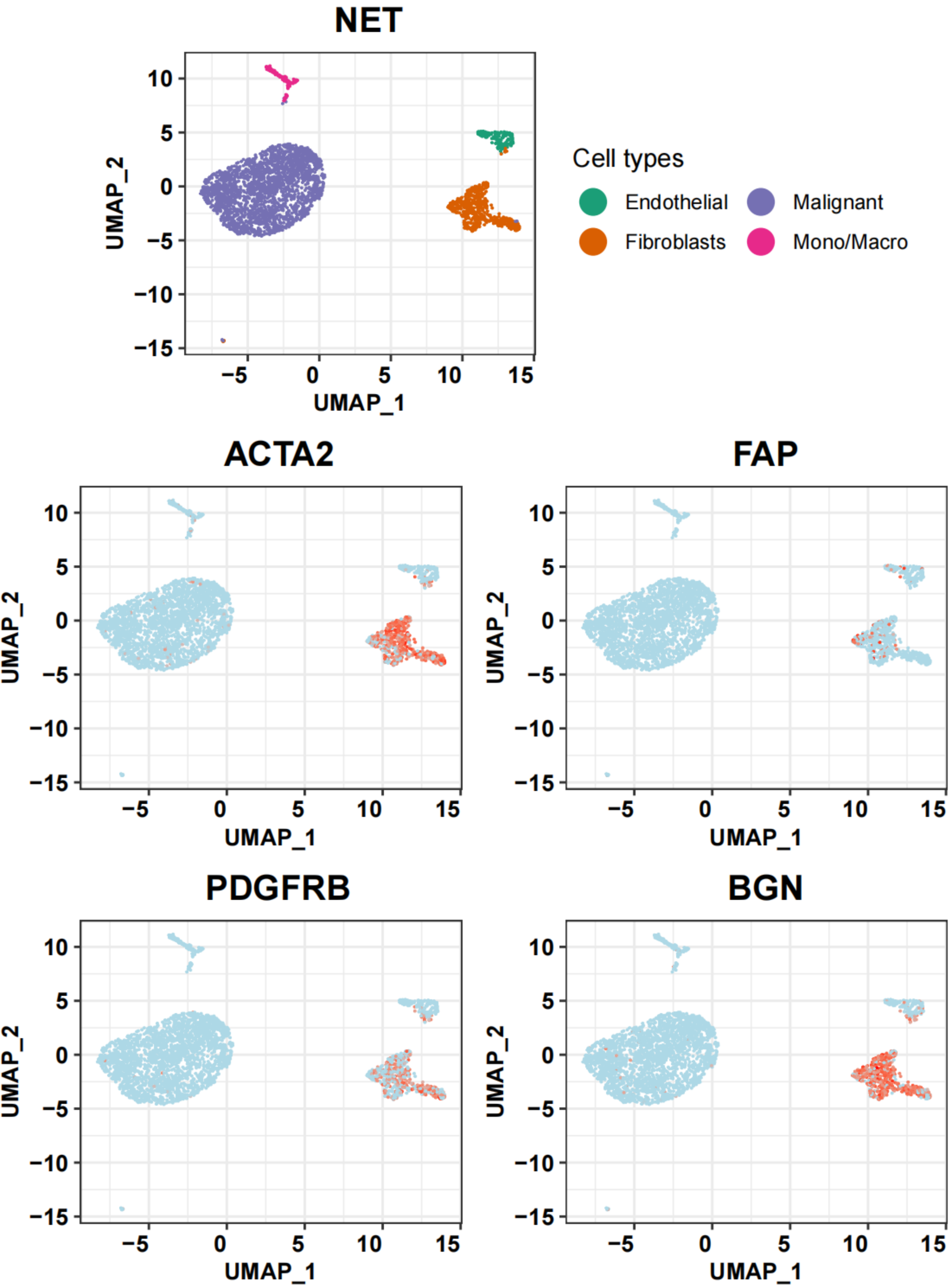

NET
Cell types
Endothelial
Malignant
Fibroblasts
Mono/Macro
ACTA2
FAP
PDGFRB
BGN
UMAP_1
UMAP_2

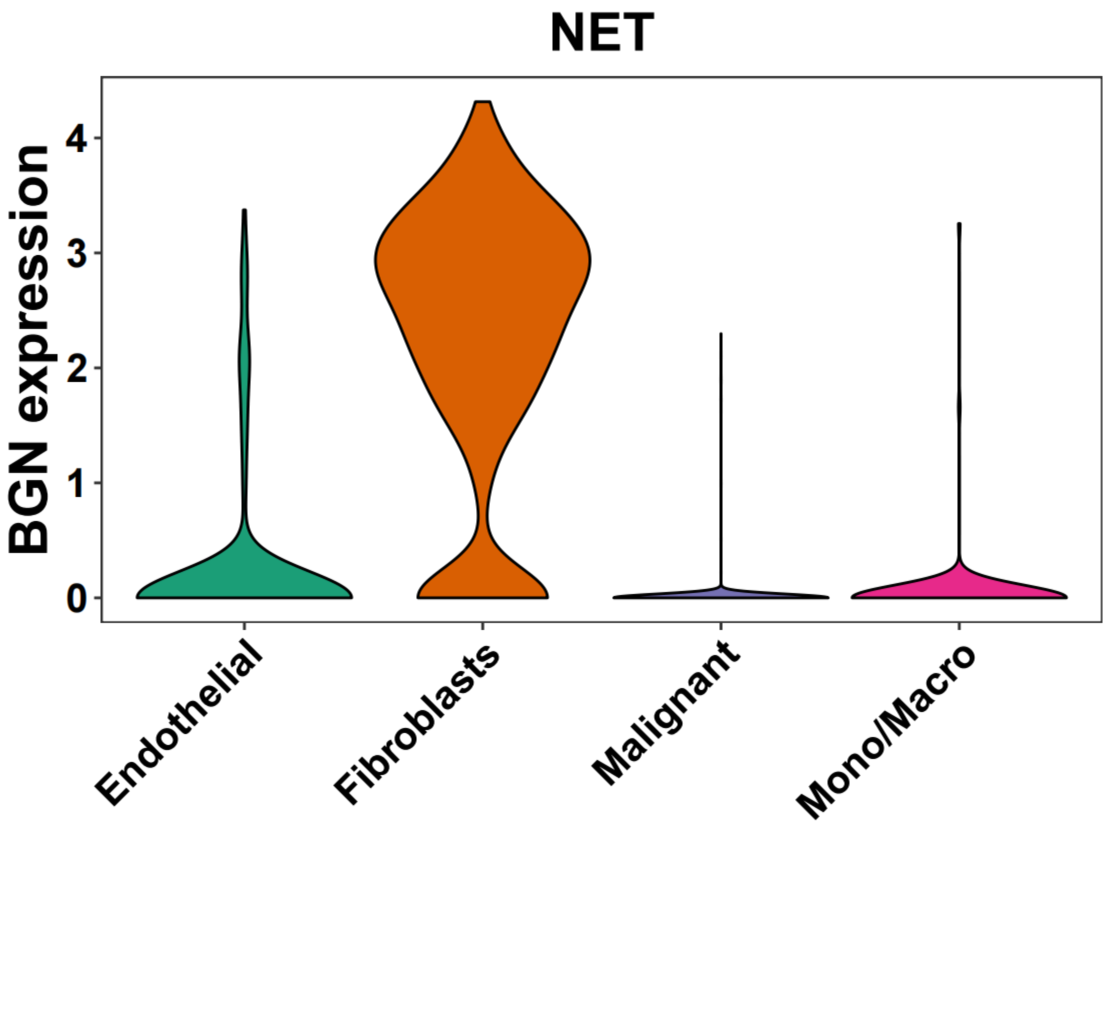
NET
BGN expression
0
1
2
3
4
Endothelial
Fibroblasts
Malignant
Mono/Macro

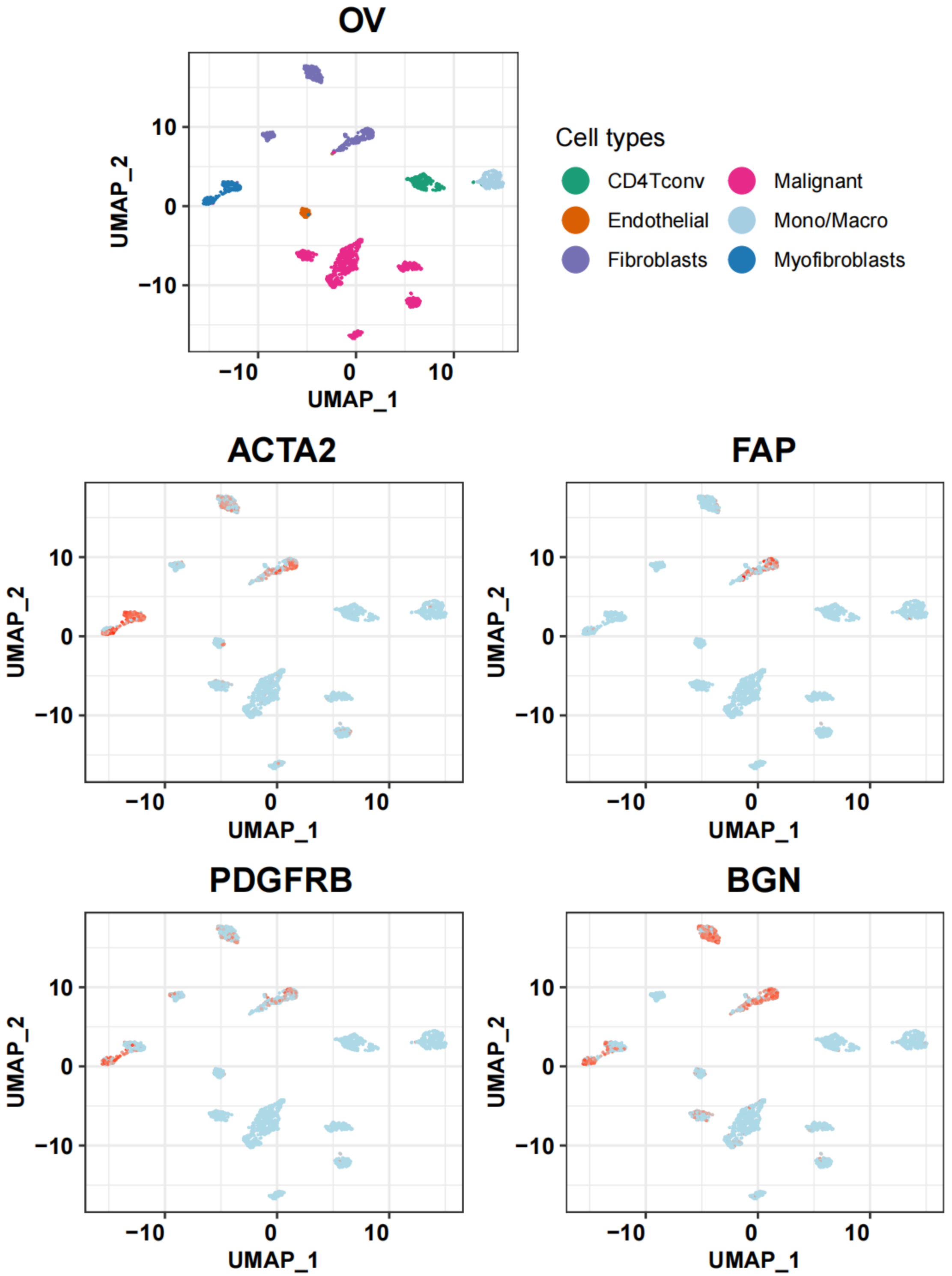
OV
Cell types
CD4Tconv
Endothelial
Fibroblasts
Malignant
Mono/Macro
Myofibroblasts
ACTA2
FAP
PDGFRB
BGN
UMAP_1
UMAP_2
−10
0
10

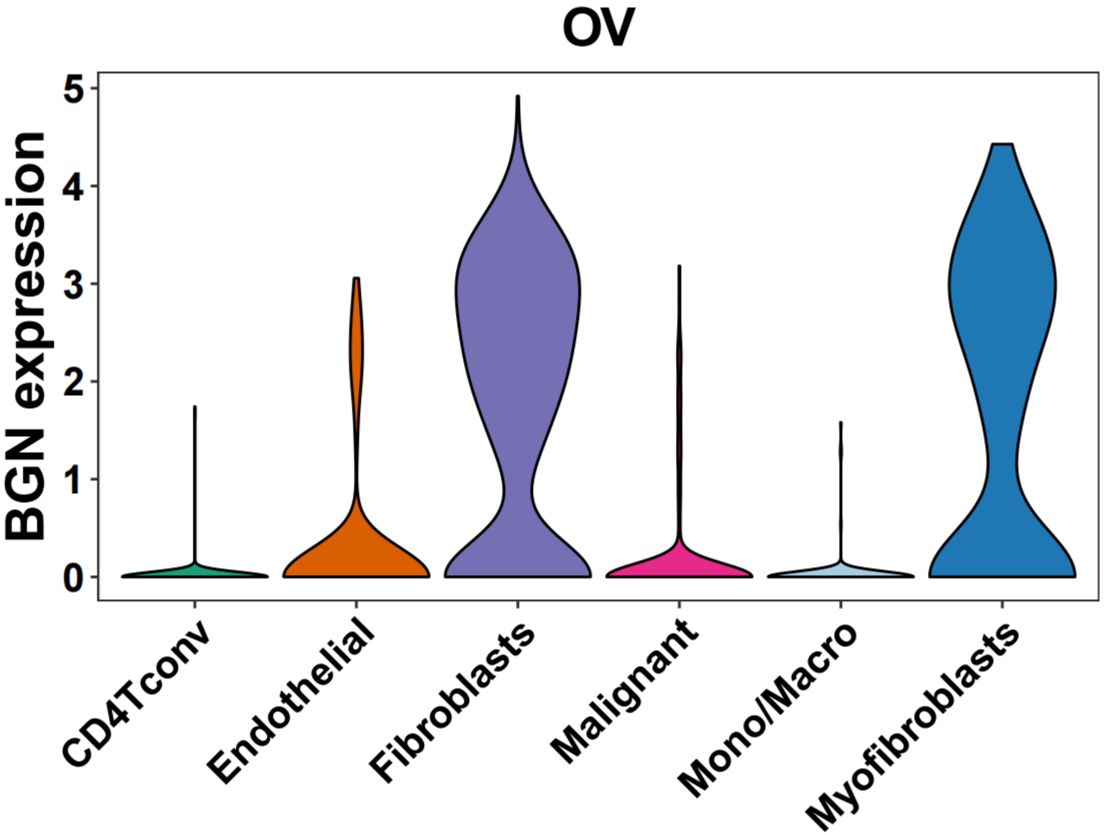
OV
BGN expression
0
1
2
3
4
5
CD4Tconv
Endothelial
Fibroblasts
Malignant
Mono/Macro
Myofibroblasts

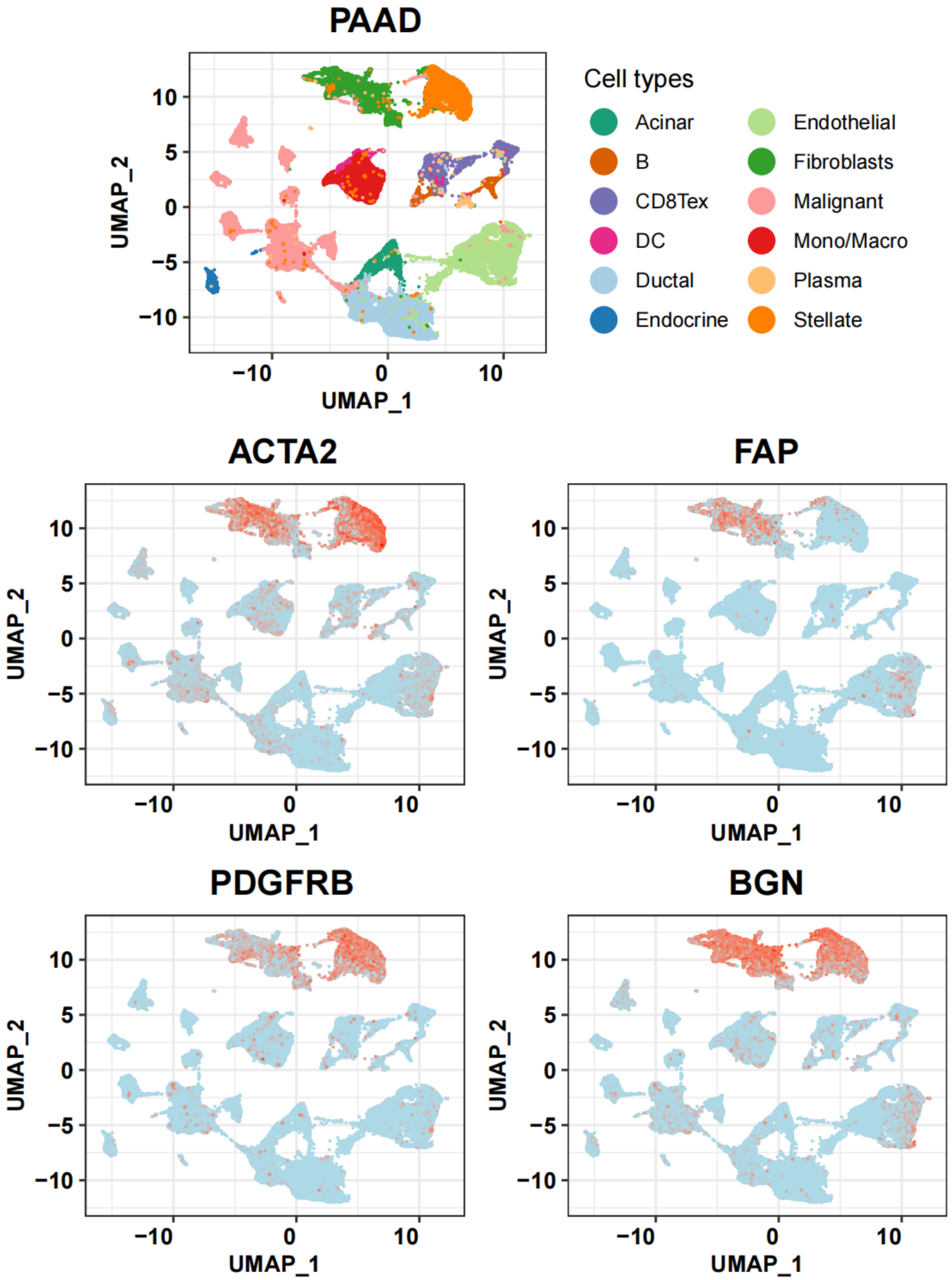

PAAD
Cell types
Acinar
B
CD8Tex
DC
Ductal
Endocrine
Endothelial
Fibroblasts
Malignant
Mono/Macro
Plasma
Stellate
UMAP_1
UMAP_2
ACTA2
FAP
PDGFRB
BGN

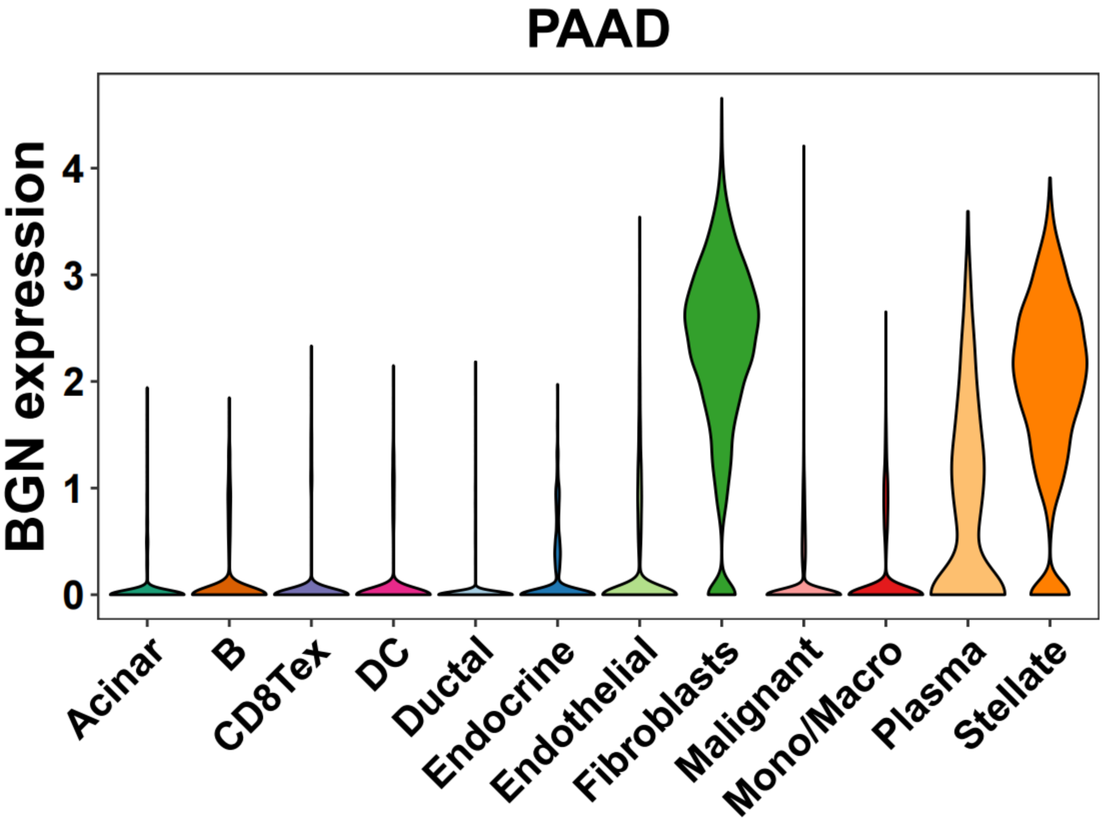
PAAD
BGN expression
0
1
2
3
4
Acinar
B
CD8Tex
DC
Ductal
Endocrine
Endothelial
Fibroblasts
Malignant
Mono/Macro
Plasma
Stellate

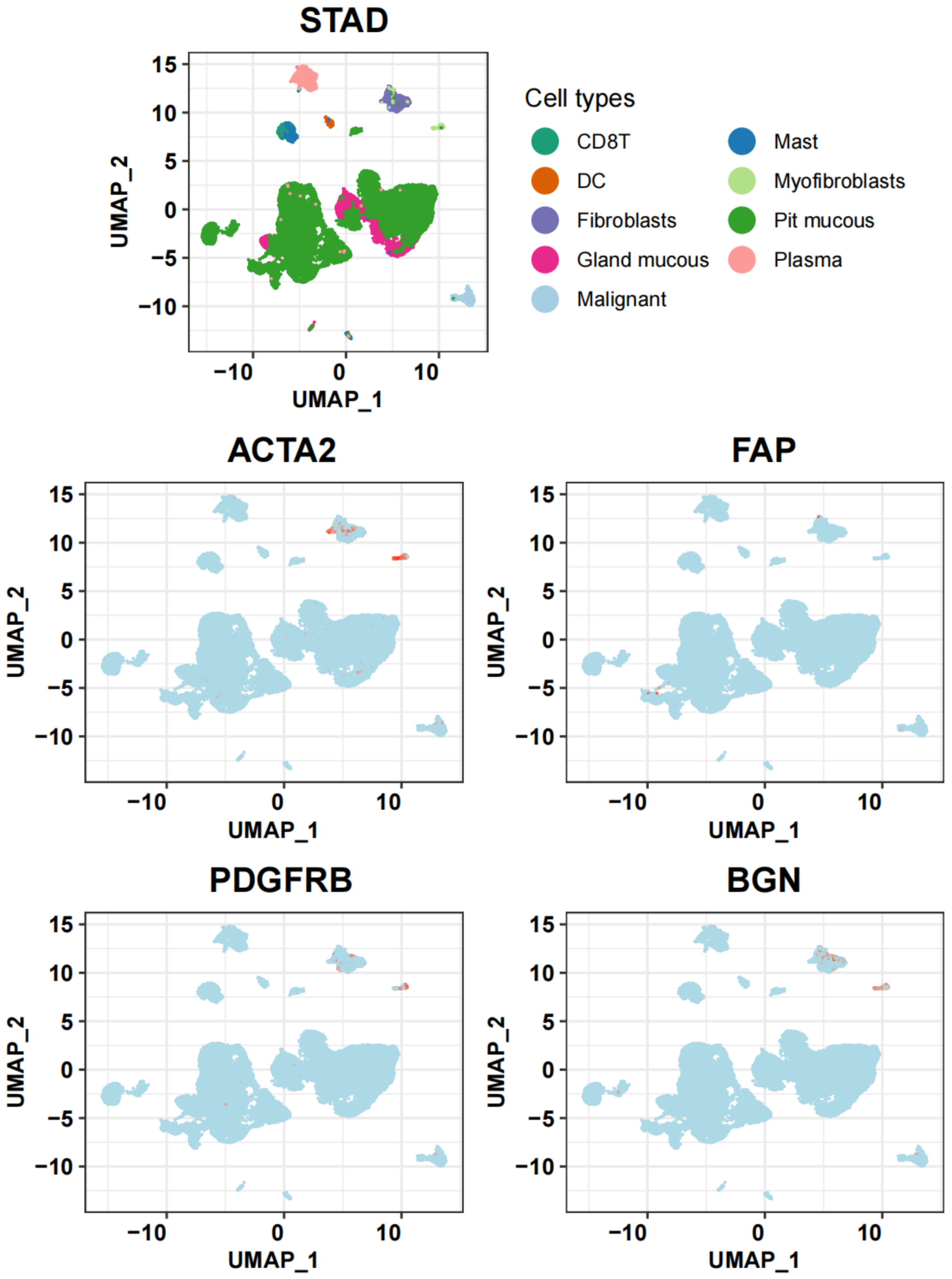
STAD
UMAP_1
UMAP_2
Cell types
CD8T
DC
Fibroblasts
Gland mucous
Malignant
Mast
Myofibroblasts
Pit mucous
Plasma
ACTA2
FAP
PDGFRB
BGN

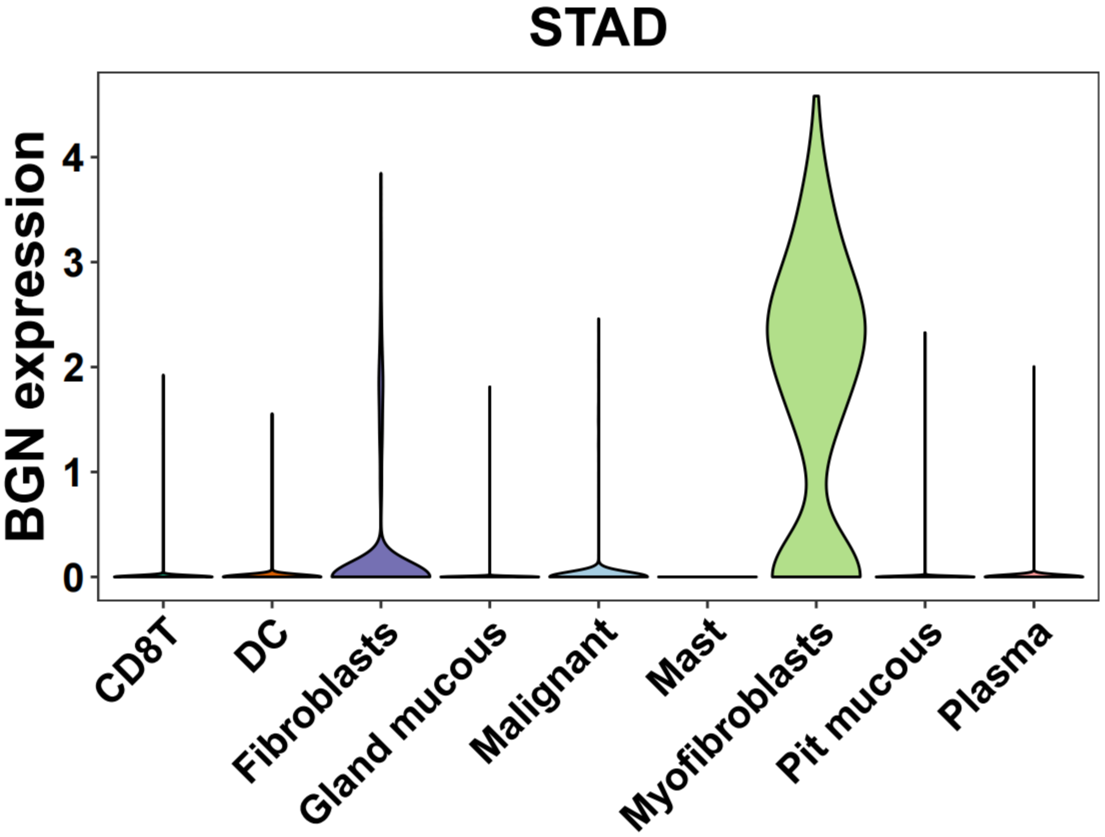

STAD
BGN expression
0
1
2
3
4
CD8T
DC
Fibroblasts
Gland mucous
Malignant
Mast
Myofibroblasts
Pit mucous
Plasma

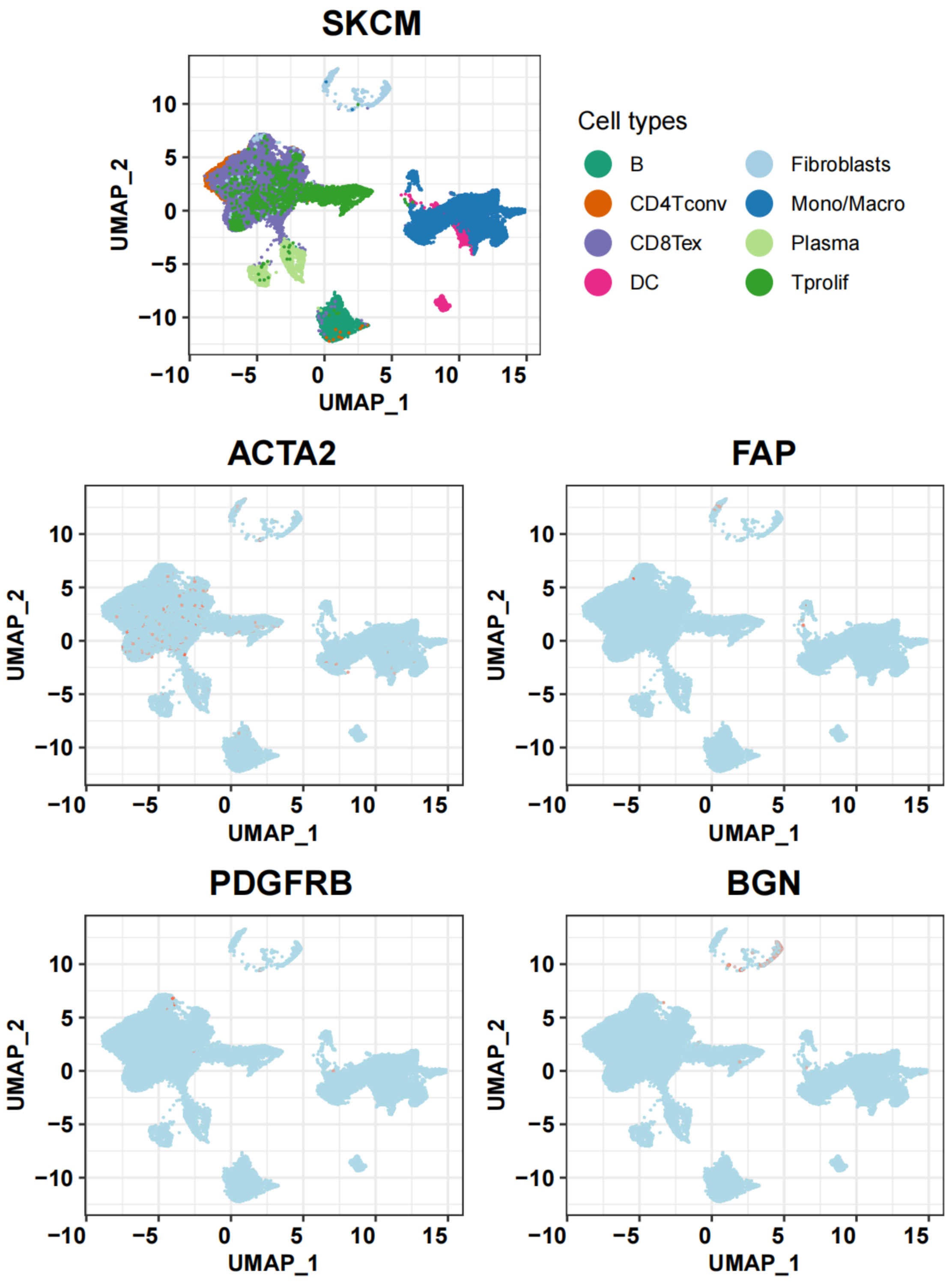
SKCM
Cell types
B
CD4Tconv
CD8Tex
DC
Fibroblasts
Mono/Macro
Plasma
Tprolif
ACTA2
FAP
PDGFRB
BGN
UMAP_1
UMAP_2

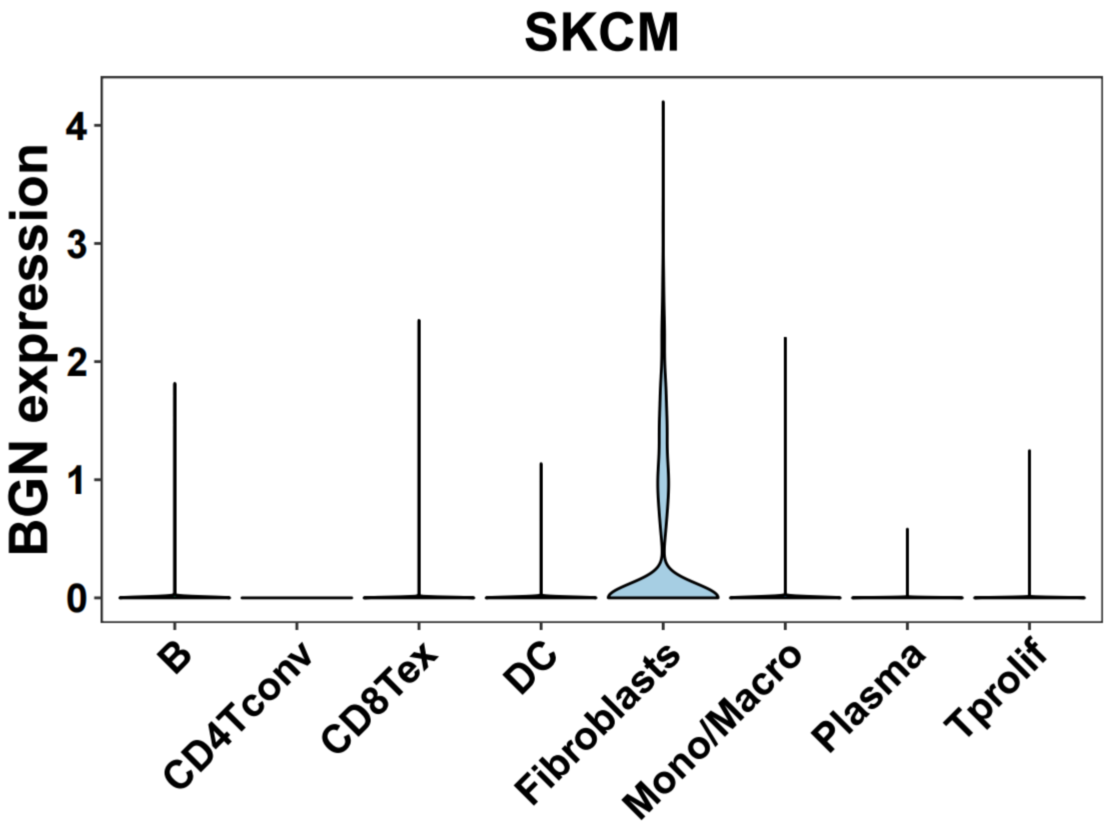
SKCM
BGN expression
0
1
2
3
4
B
CD4Tconv
CD8Tex
DC
Fibroblasts
Mono/Macro
Plasma
Tprolif
