# Supplementary material for: Dissecting the role of cancer‐associated fibroblast‐derived biglycan as a potential therapeutic target in immunotherapy resistance: A tumor bulk and single‐cell transcriptomic study
Source: Clin Transl Med. 2023 Feb 11;13(2):e1189. doi: 10.1002/ctm2.1189 (PMC9920016; doi:10.1002/ctm2.1189)
Supplement: Supplementary file 8 — Supporting Information [file CTM2-13-e1189-s005.pdf]

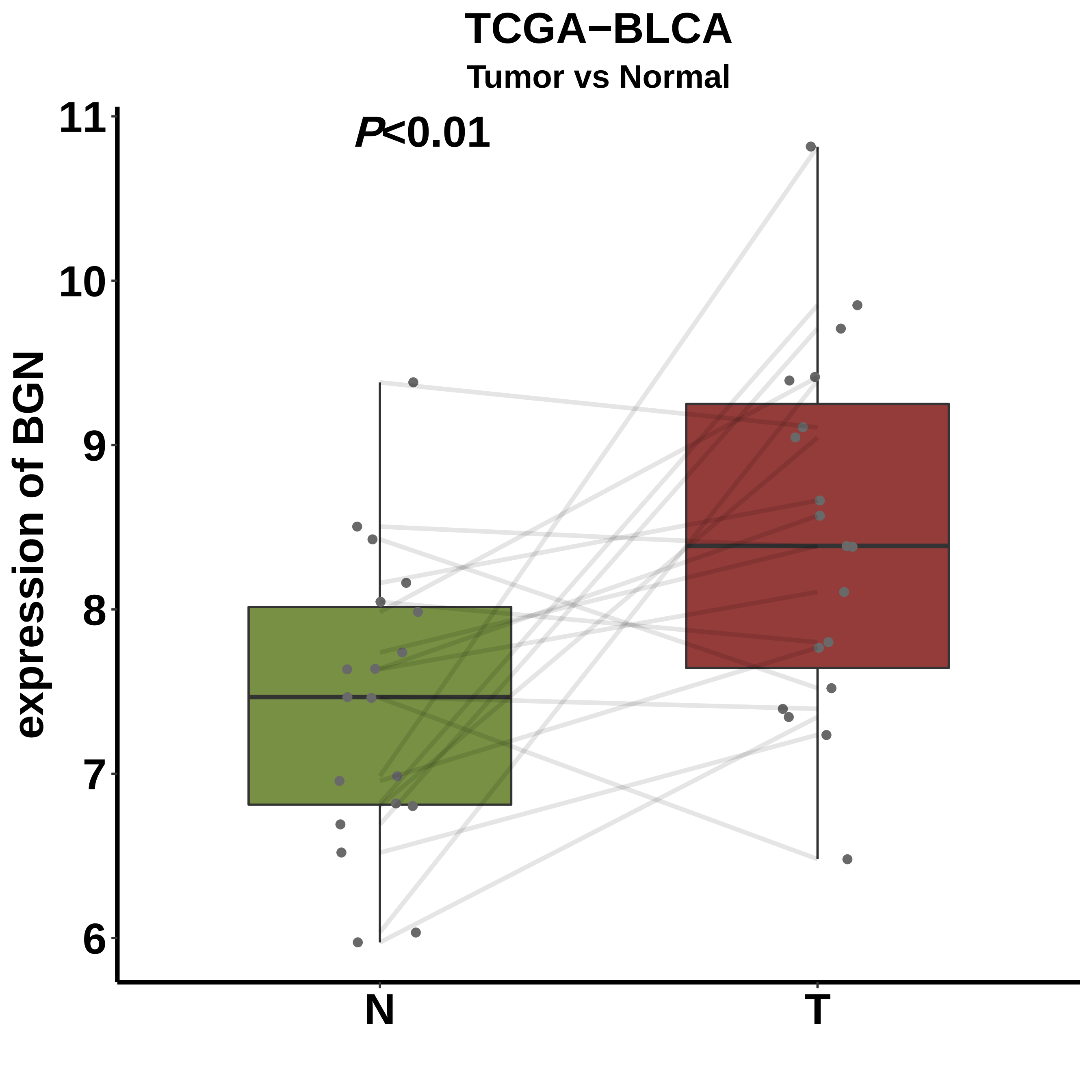
TCGA−BLCA
Tumor vs Normal
P<0.01
expression of BGN
11
10
9
8
7
6
N
T

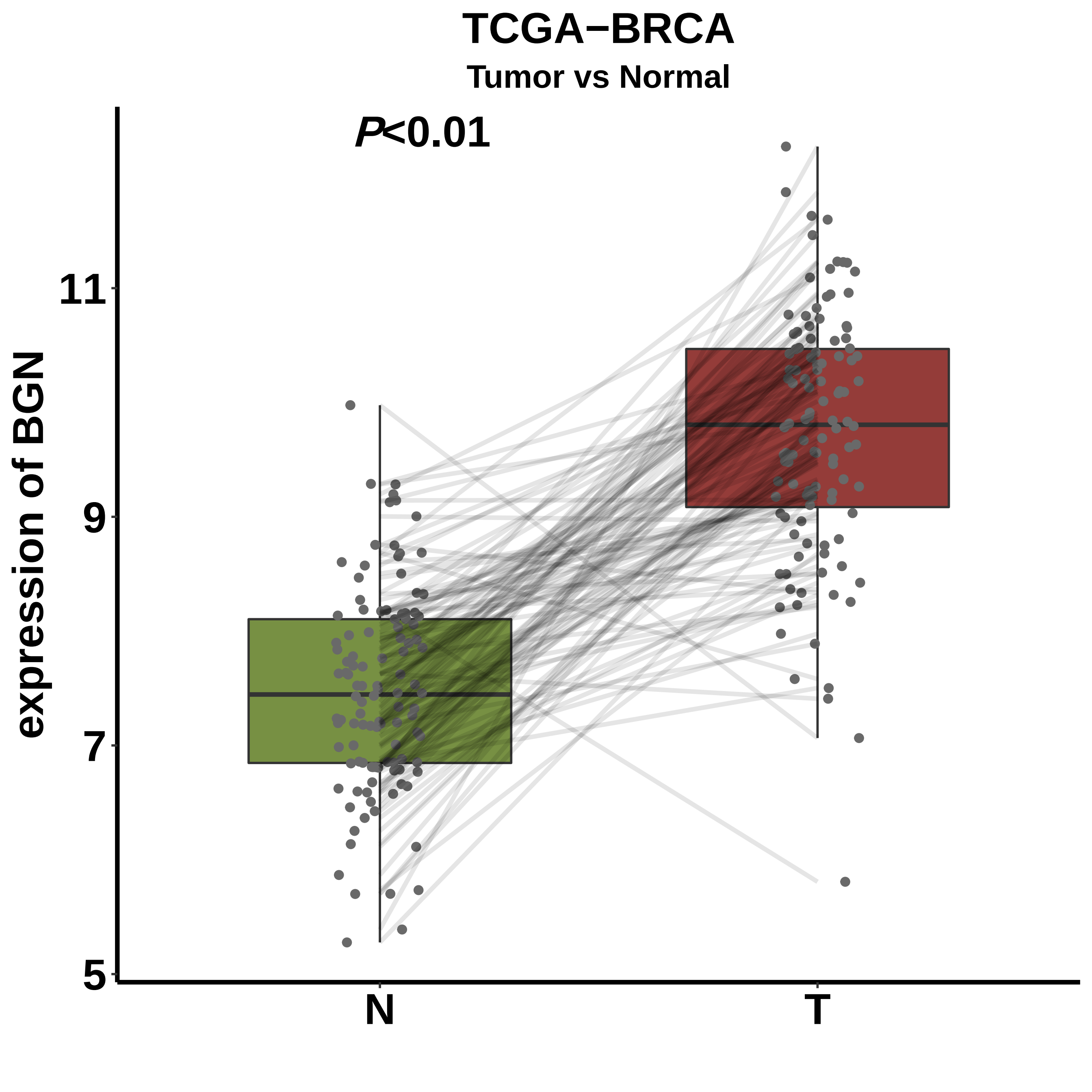

TCGA−BRCA
Tumor vs Normal
P<0.01
expression of BGN
11
9
7
5
N
T

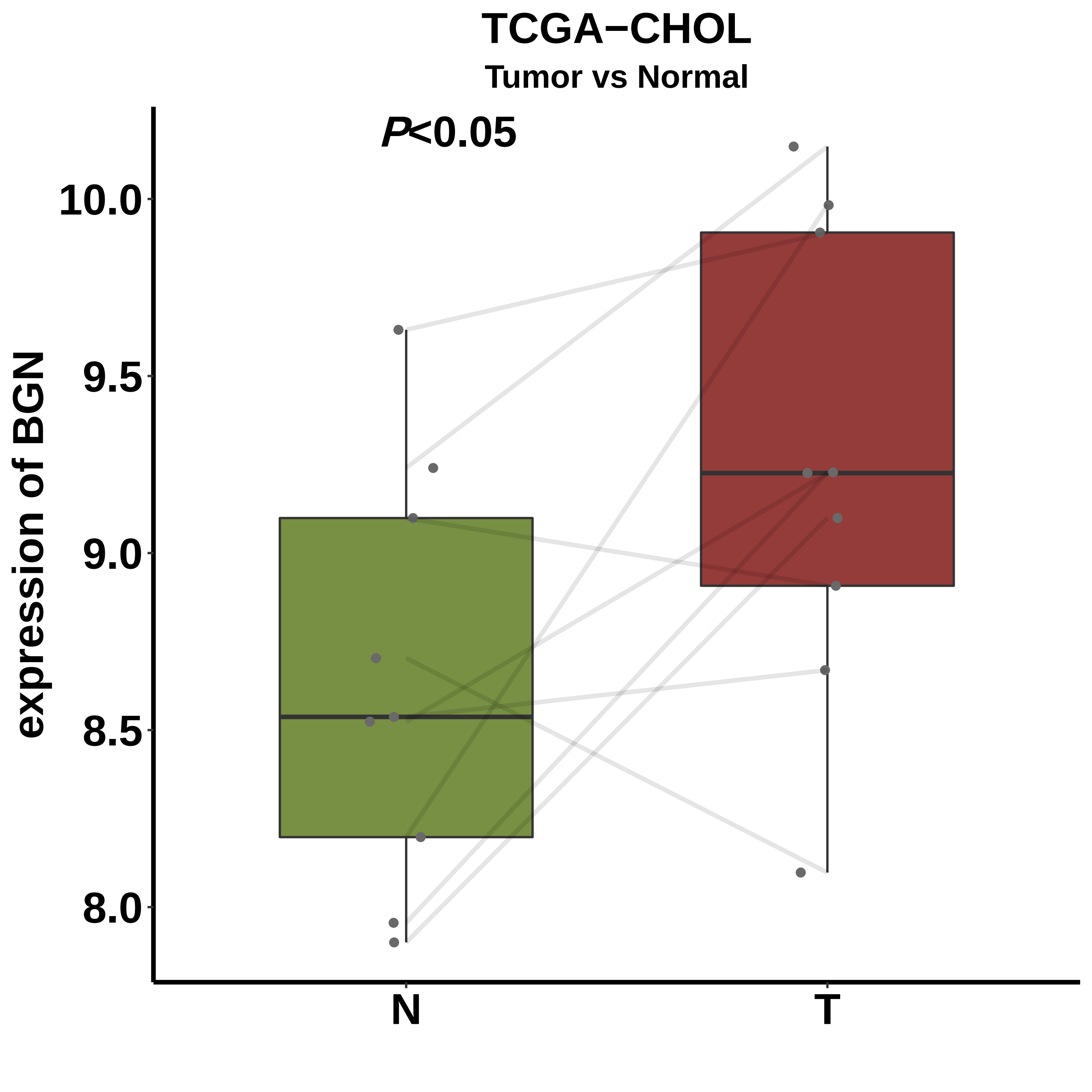
TCGA−CHOL
Tumor vs Normal
P<0.05
expression of BGN
10.0
9.5
9.0
8.5
8.0
N
T

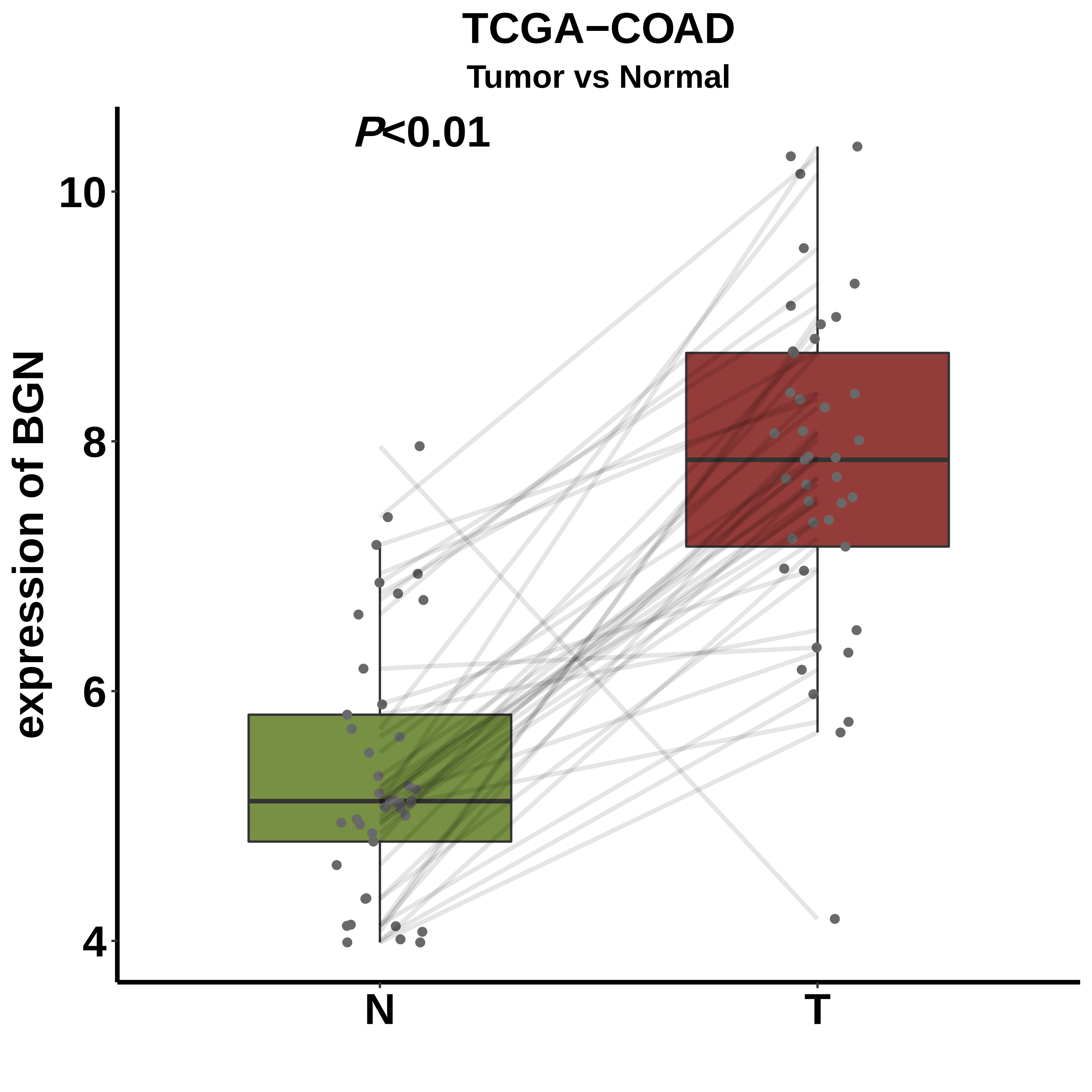
TCGA−COAD
Tumor vs Normal
P<0.01
expression of BGN
10
8
6
4
N
T

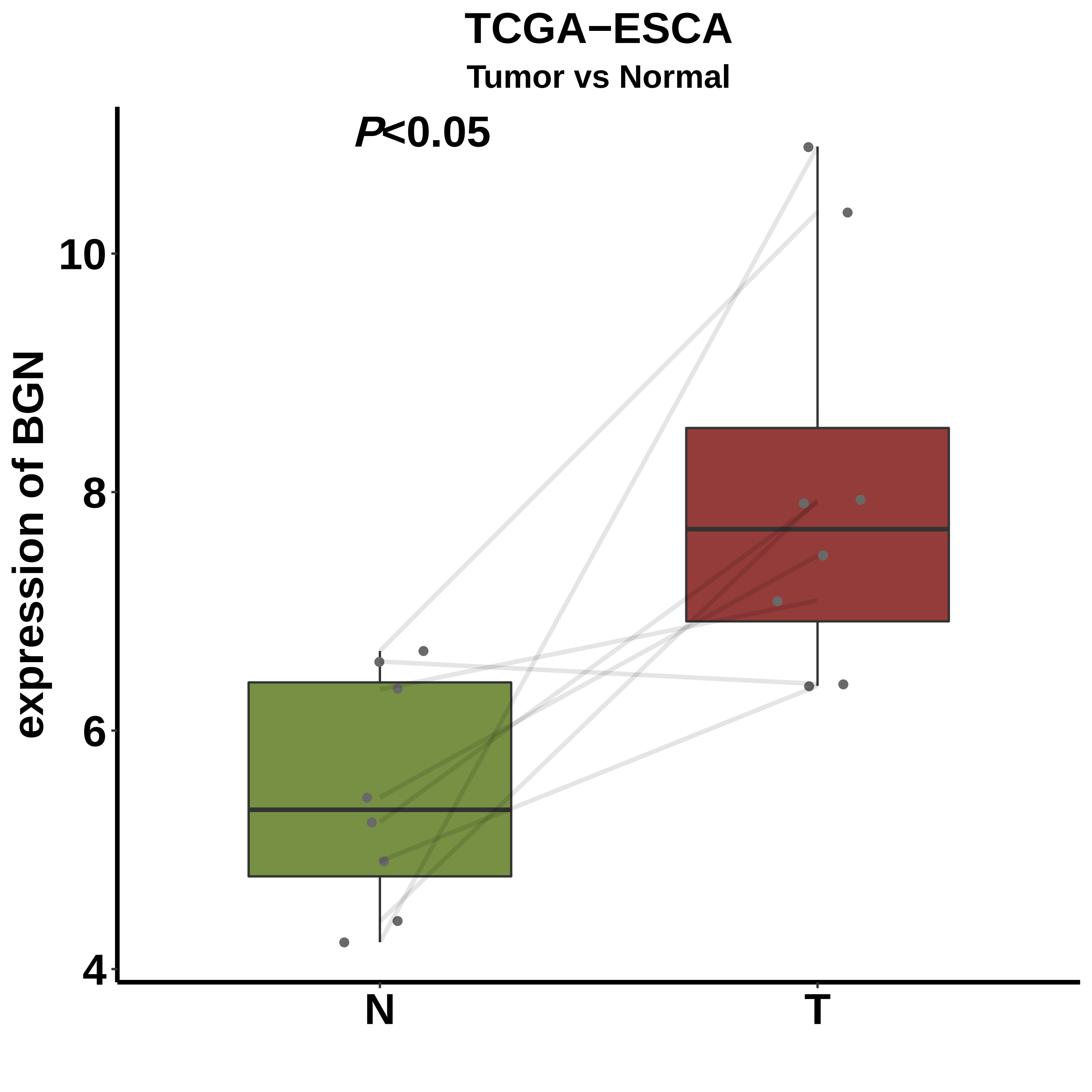
TCGA−ESCA
Tumor vs Normal
P<0.05
expression of BGN
10
8
6
4
N
T

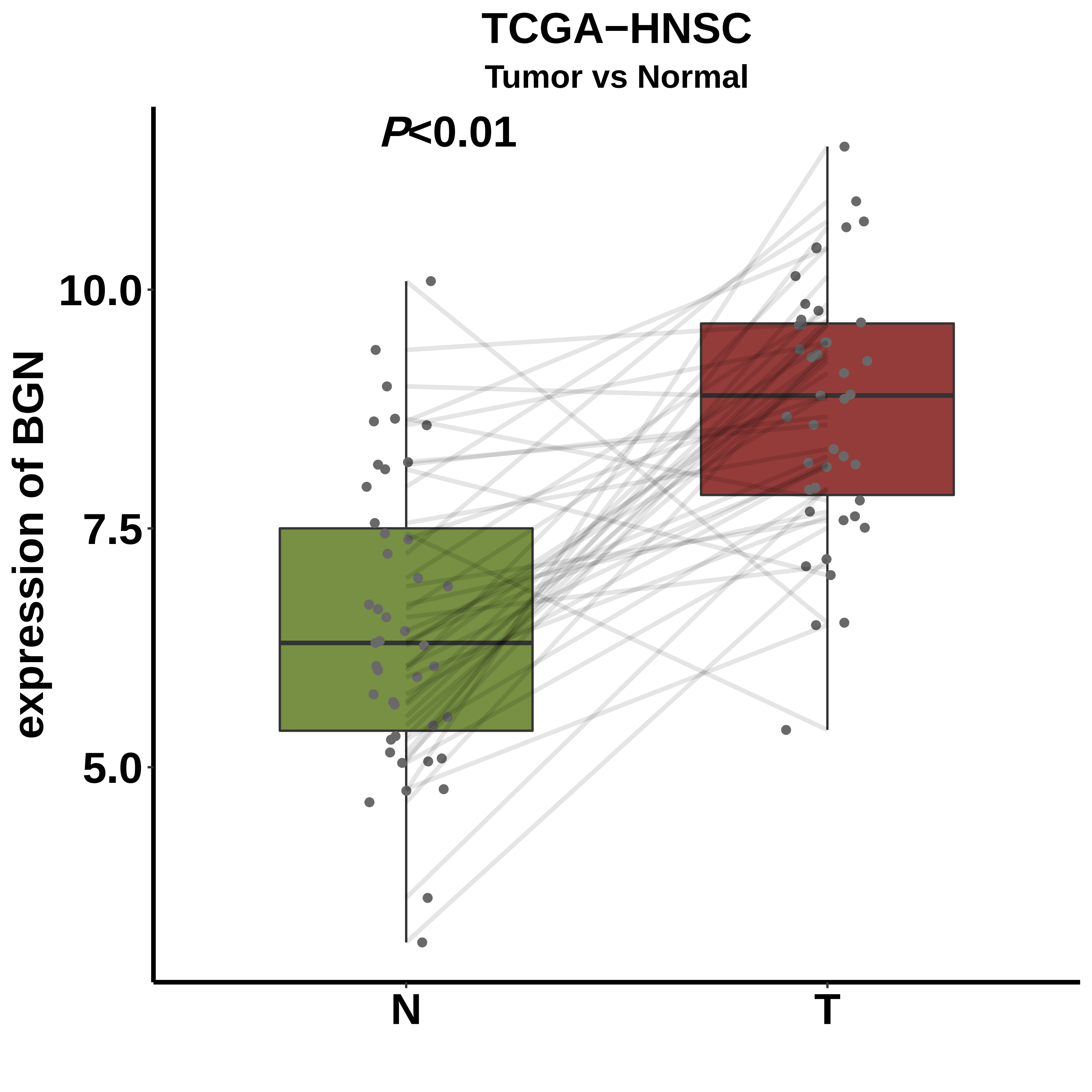

TCGA−HNSC
Tumor vs Normal
P<0.01
expression of BGN
10.0
7.5
5.0
N
T

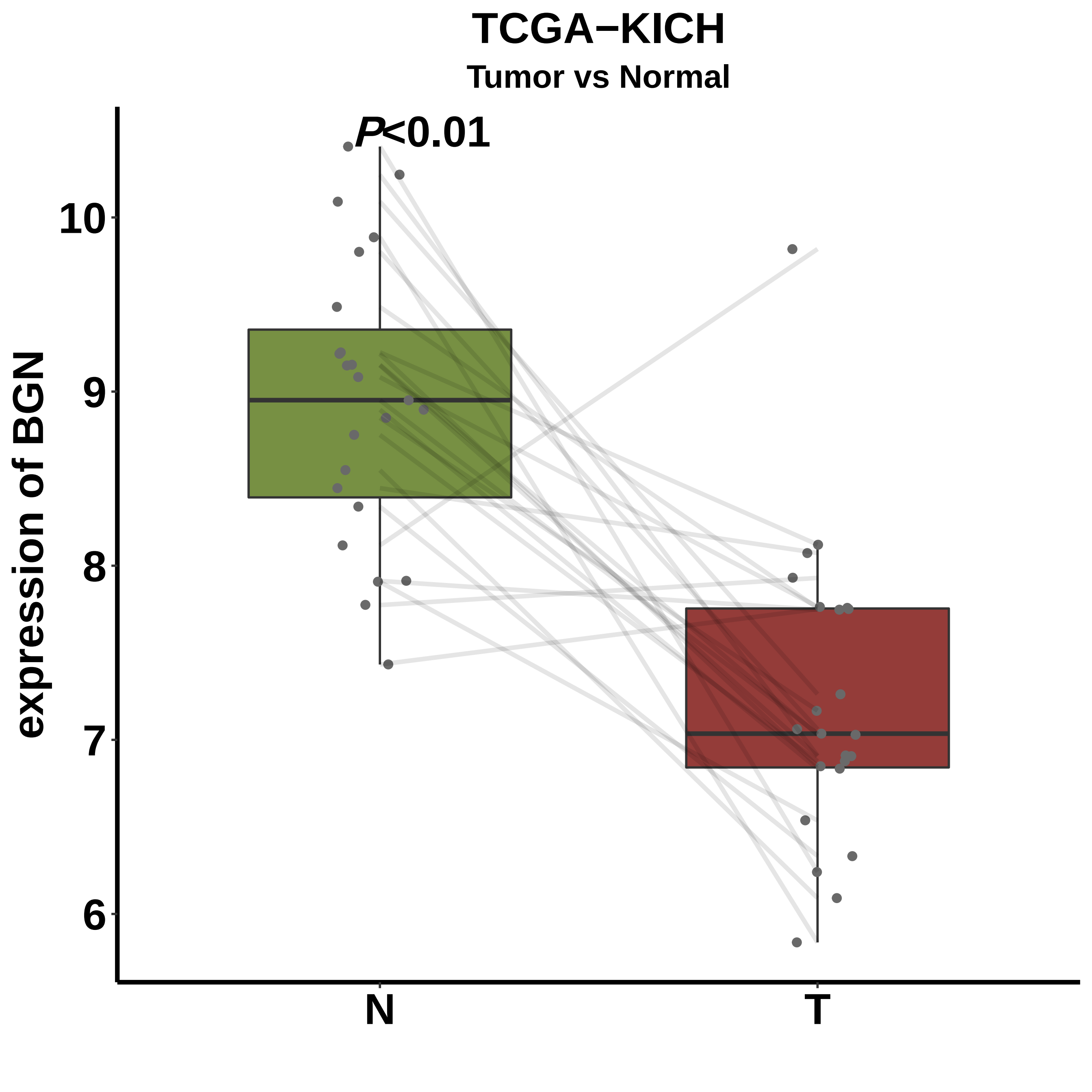

TCGA−KICH
Tumor vs Normal
P<0.01
expression of BGN
10
9
8
7
6
N
T

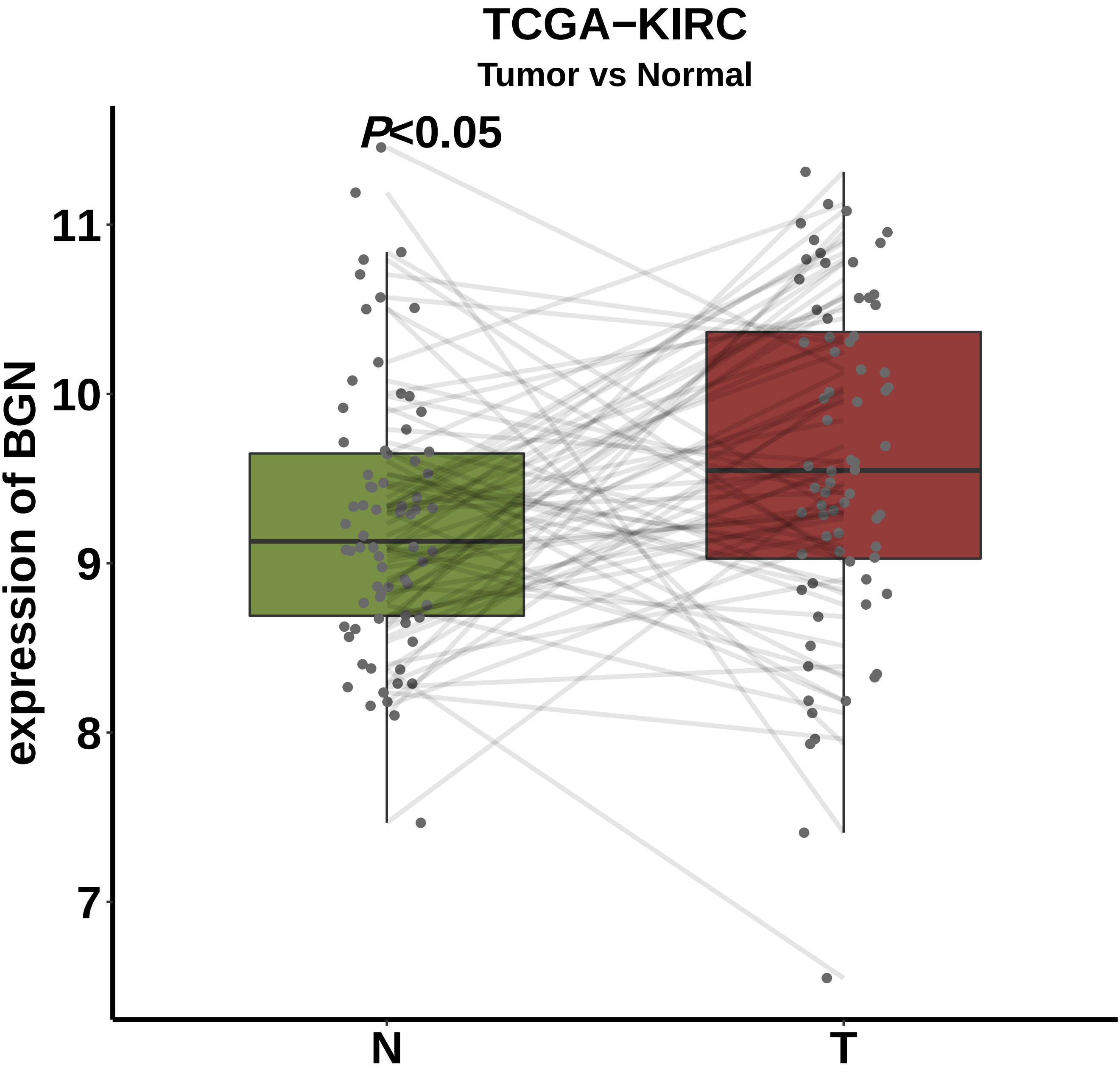

TCGA−KIRC
Tumor vs Normal
P<0.05
expression of BGN
11
10
9
8
7
N
T

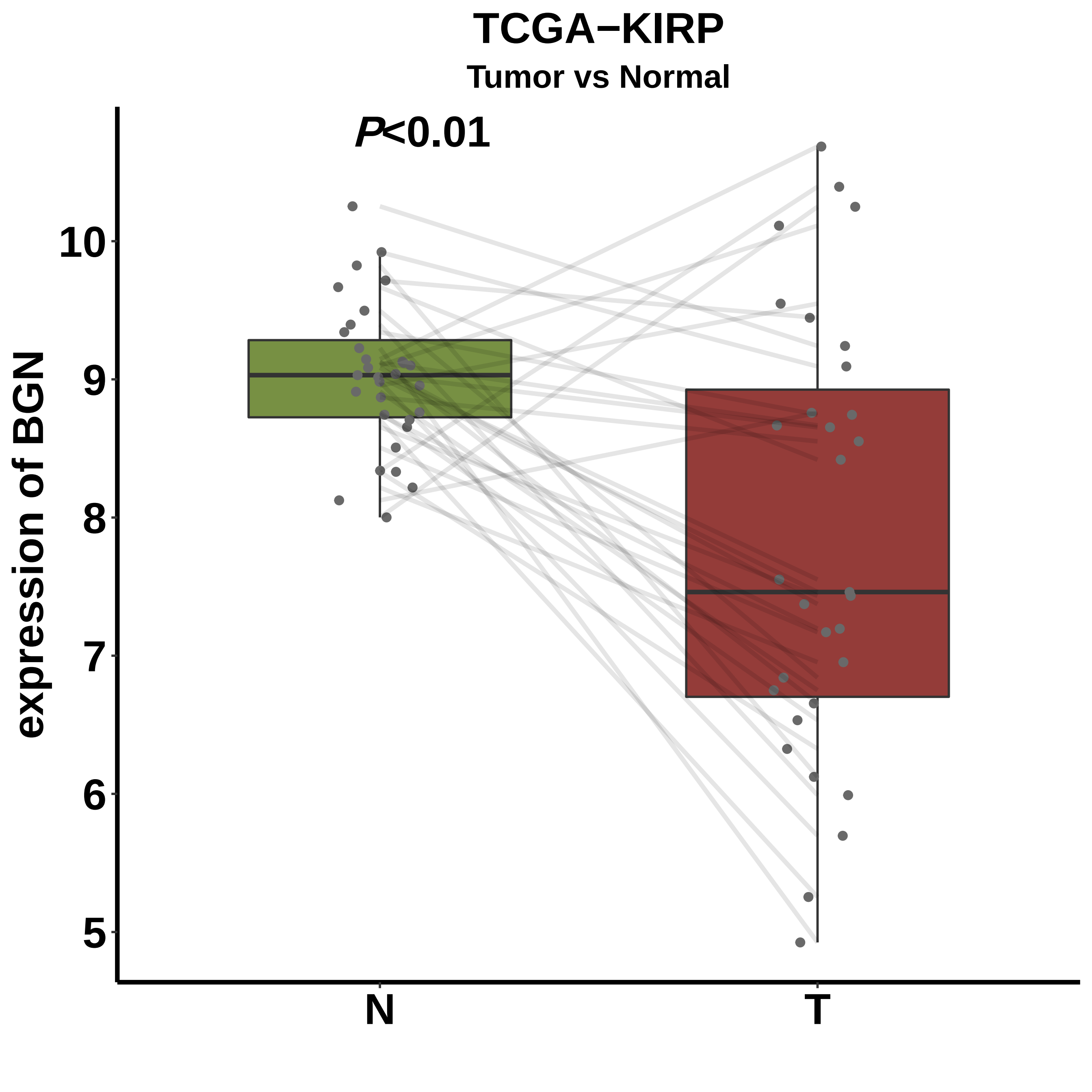
TCGA−KIRP
Tumor vs Normal
P<0.01
expression of BGN
10
9
8
7
6
5
N
T

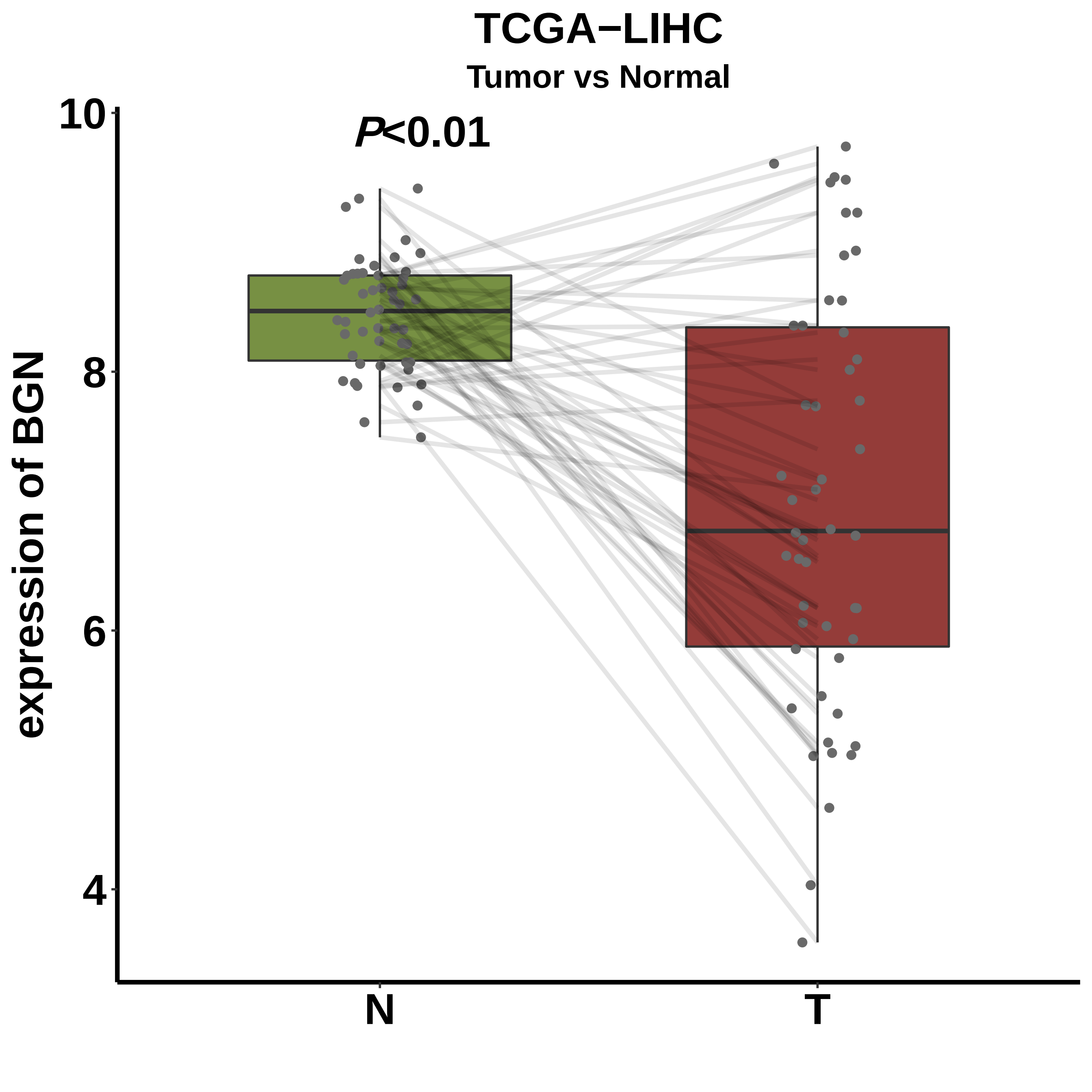
TCGA−LIHC
Tumor vs Normal
P<0.01
expression of BGN
10
8
6
4
N
T

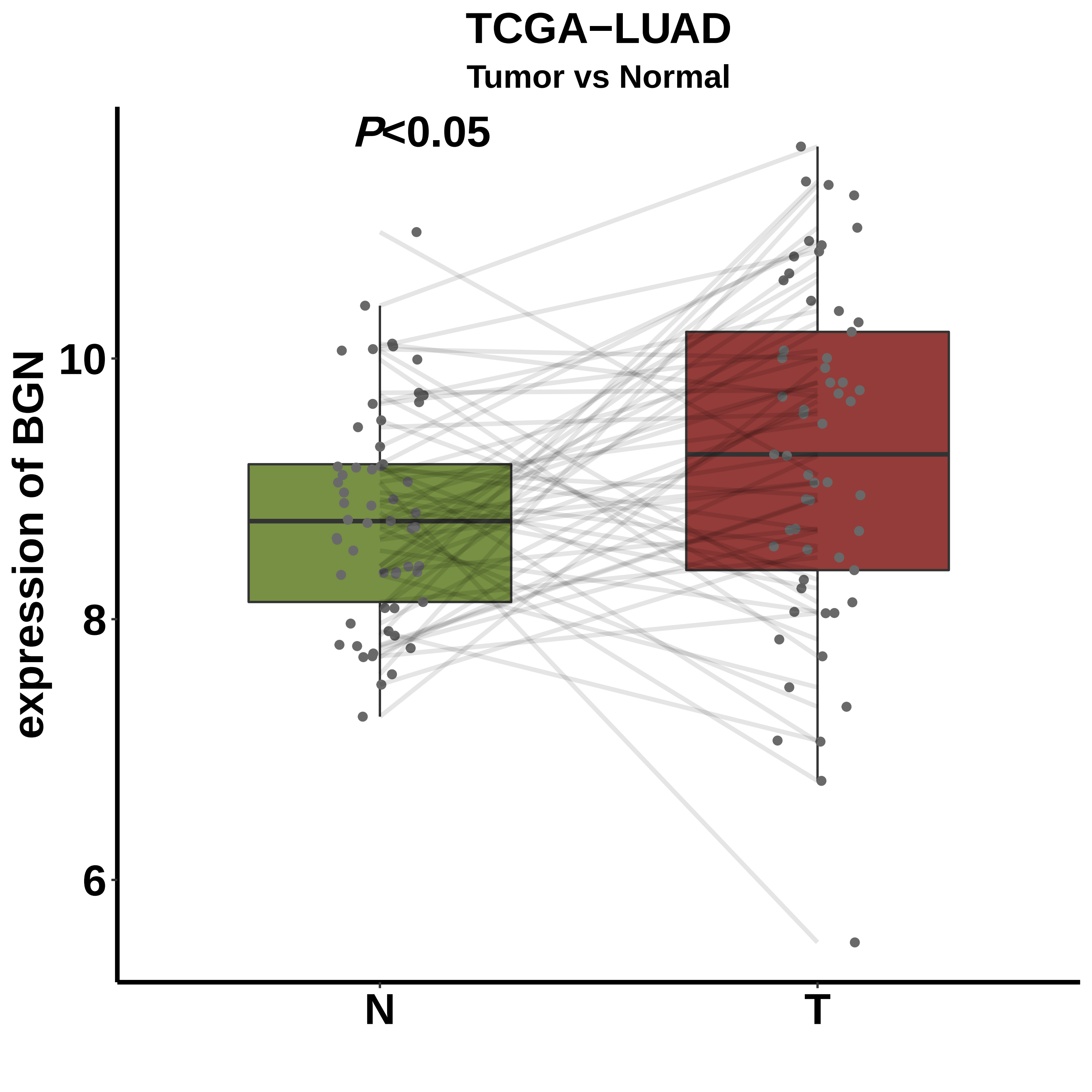
TCGA−LUAD
Tumor vs Normal
P<0.05
expression of BGN
10
8
6
N
T

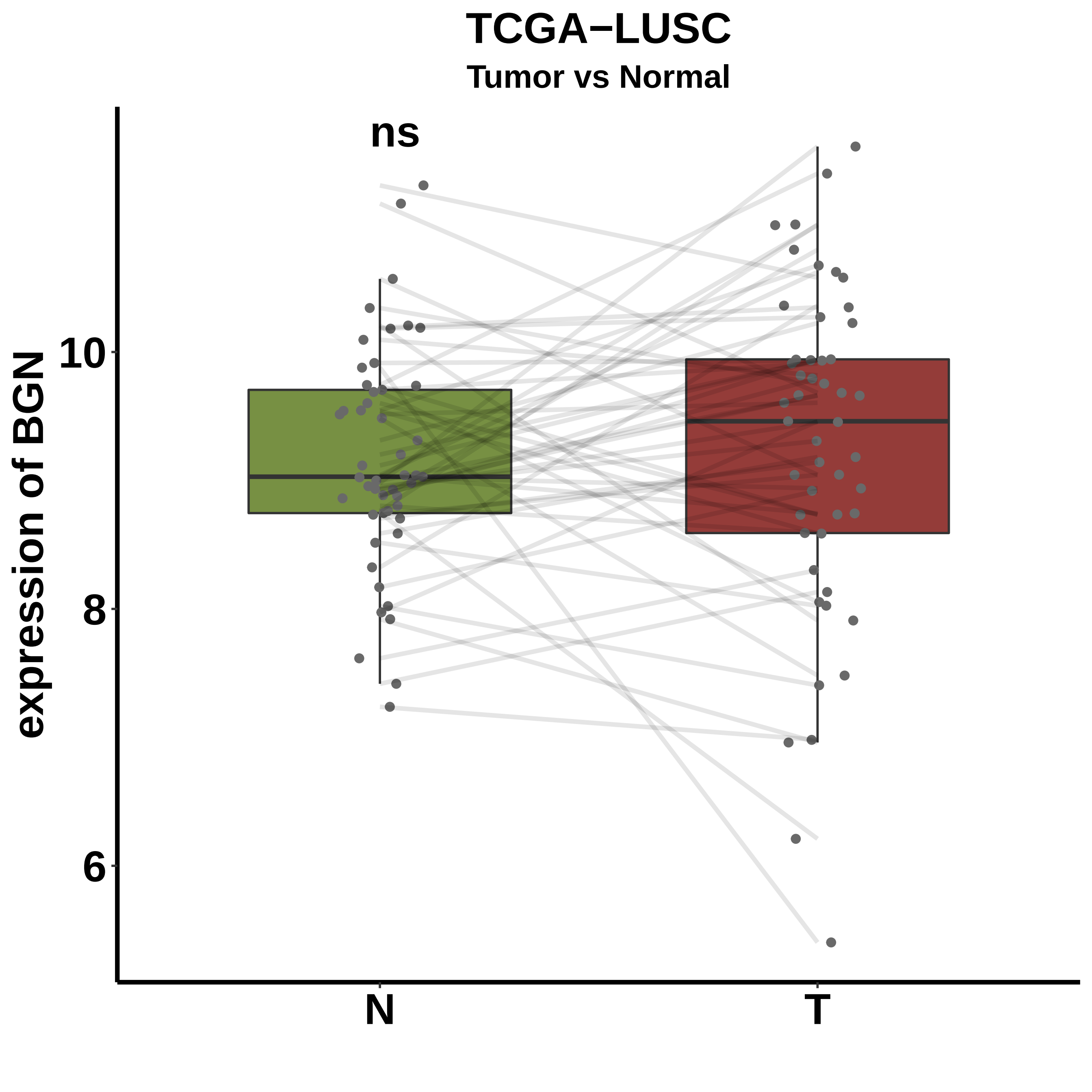

TCGA−LUSC
Tumor vs Normal
ns
expression of BGN
10
8
6
N
T

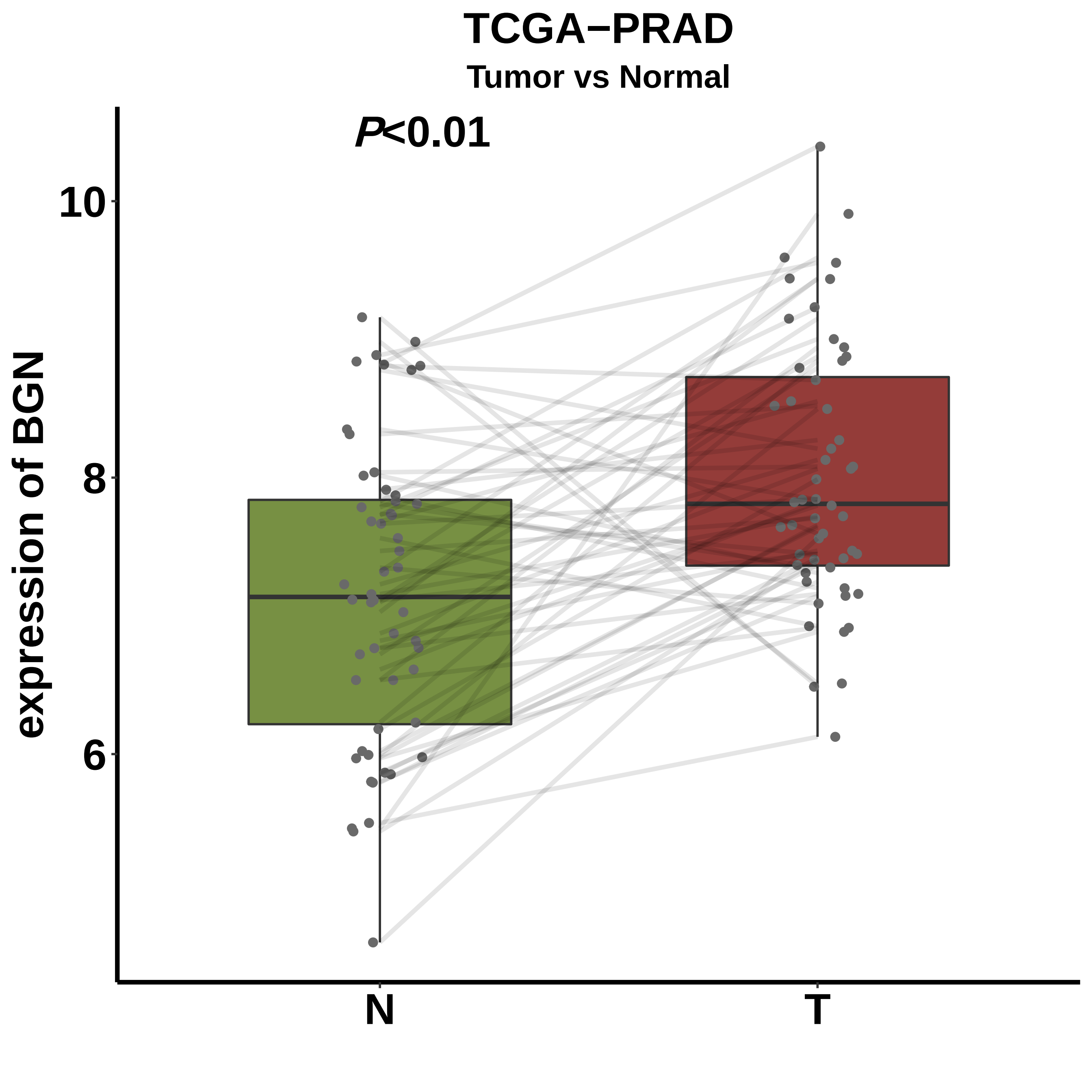
TCGA−PRAD
Tumor vs Normal
P<0.01
expression of BGN
10
8
6
N
T

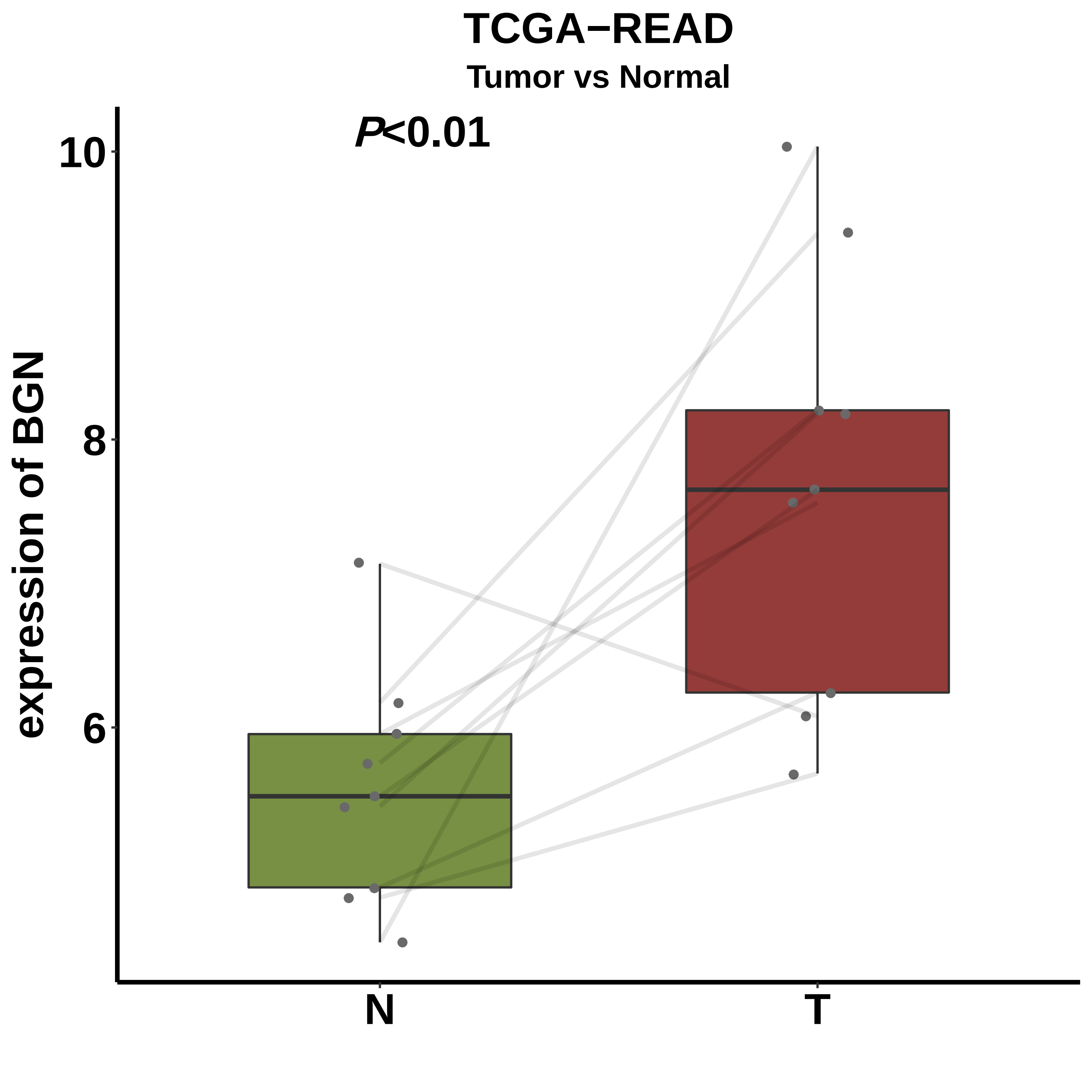

TCGA−READ
Tumor vs Normal
P<0.01
10
8
6
expression of BGN
N
T

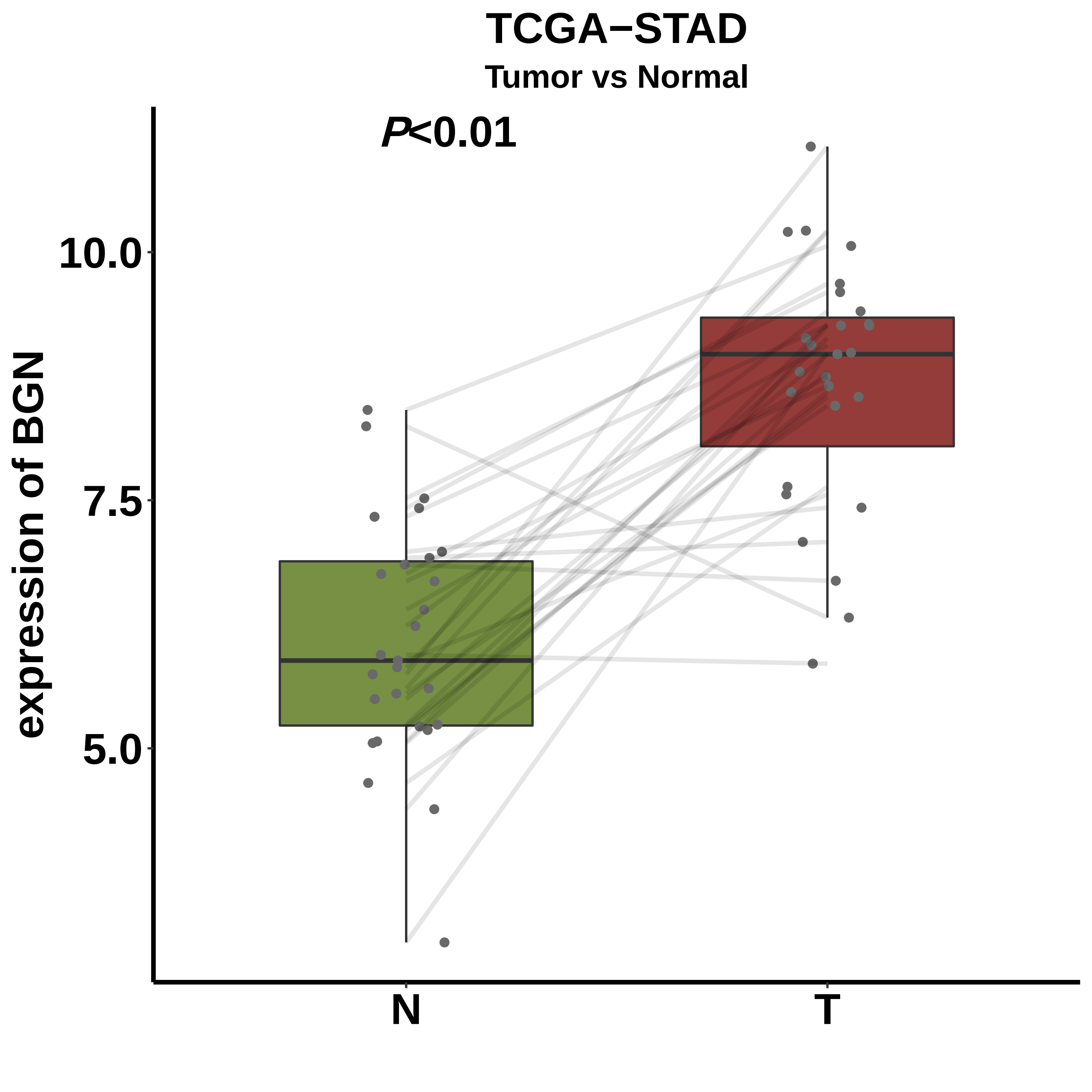
TCGA−STAD
Tumor vs Normal
P<0.01
10.0
7.5
5.0
expression of BGN
N
T

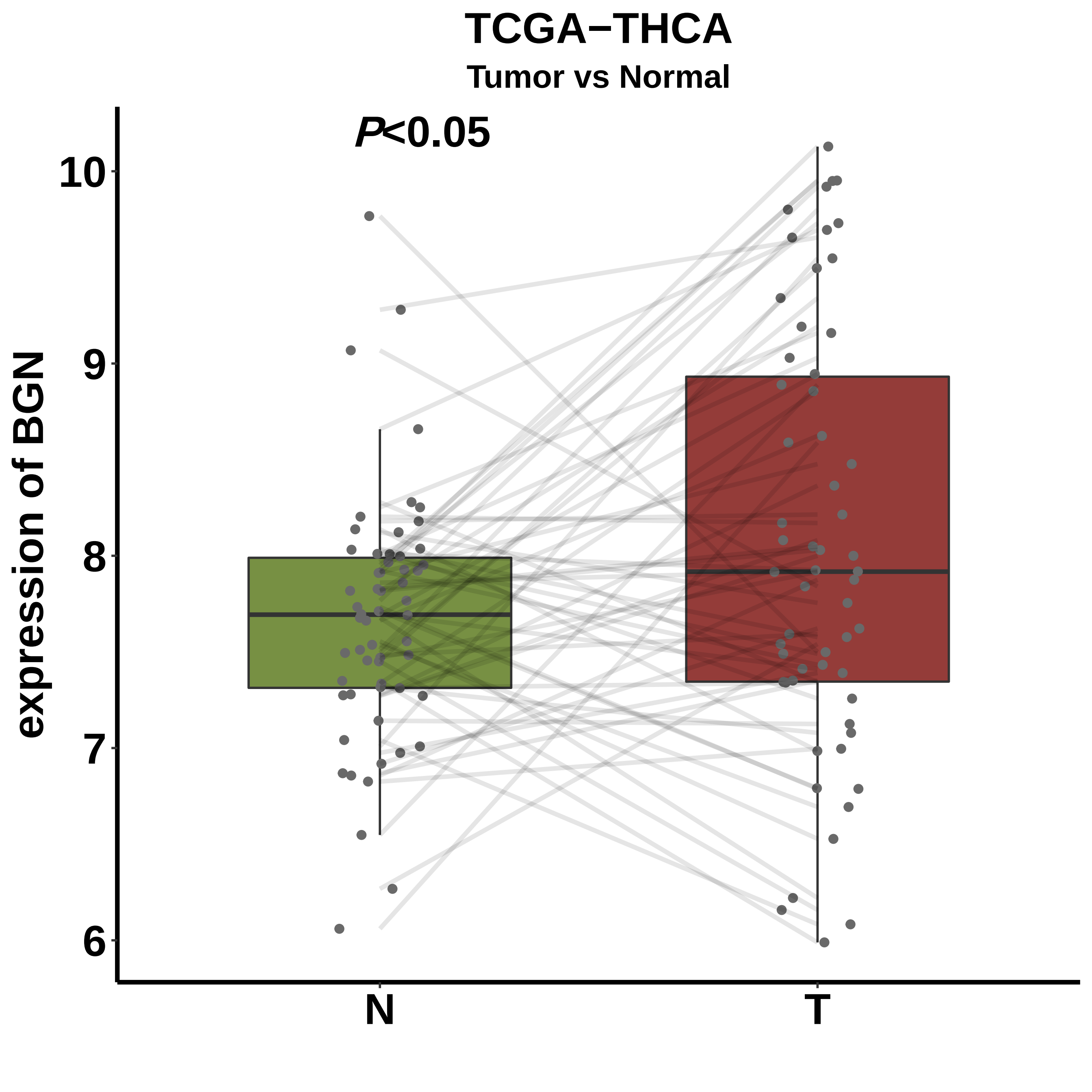
TCGA−THCA
Tumor vs Normal
P<0.05
expression of BGN
10
9
8
7
6
N
T

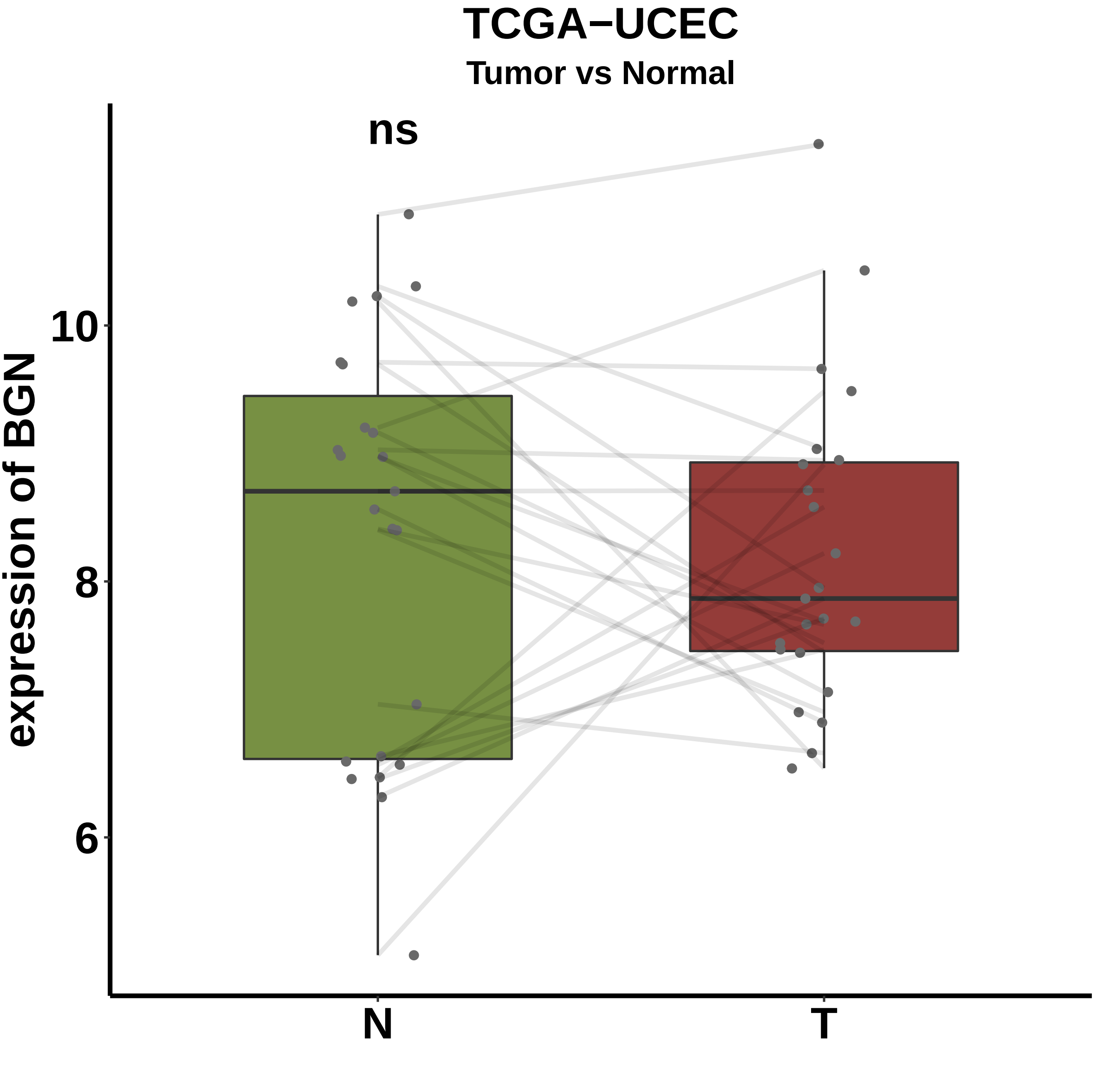
TCGA−UCEC
Tumor vs Normal
ns
expression of BGN
10
8
6
N
T
