# Supplementary material for: Dissecting the role of cancer‐associated fibroblast‐derived biglycan as a potential therapeutic target in immunotherapy resistance: A tumor bulk and single‐cell transcriptomic study
Source: Clin Transl Med. 2023 Feb 11;13(2):e1189. doi: 10.1002/ctm2.1189 (PMC9920016; doi:10.1002/ctm2.1189)
Supplement: Supplementary file 10 — Supporting Information [file CTM2-13-e1189-s017.pdf]

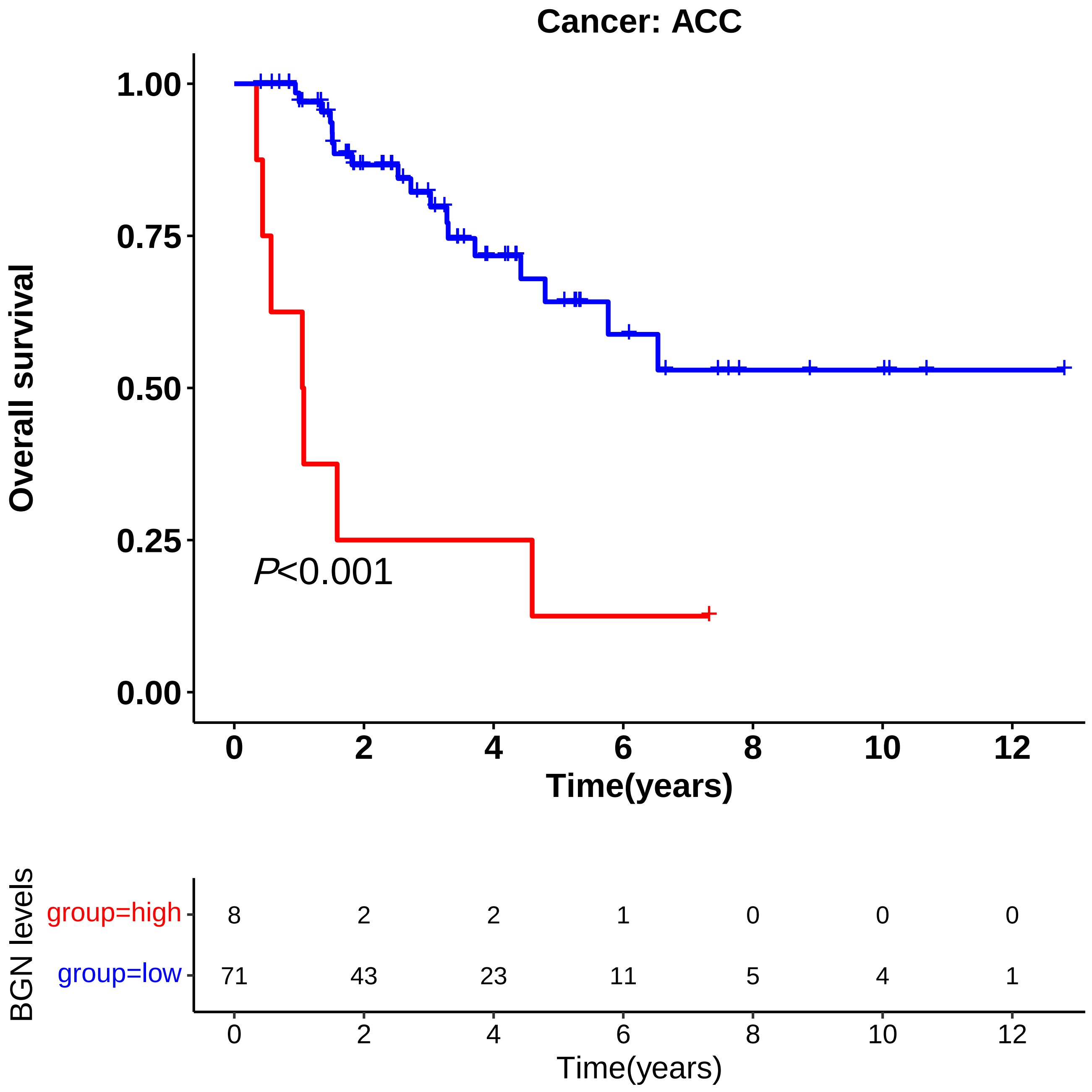

Cancer: ACC
1.00
0.75
0.50
0.25
0.00
Overall survival
P<0.001
0
2
4
6
8
10
12
Time(years)
BGN levels
group=high
group=low
8
2
2
1
0
0
0
71
43
23
11
5
4
1
0
2
4
6
8
10
12
Time(years)

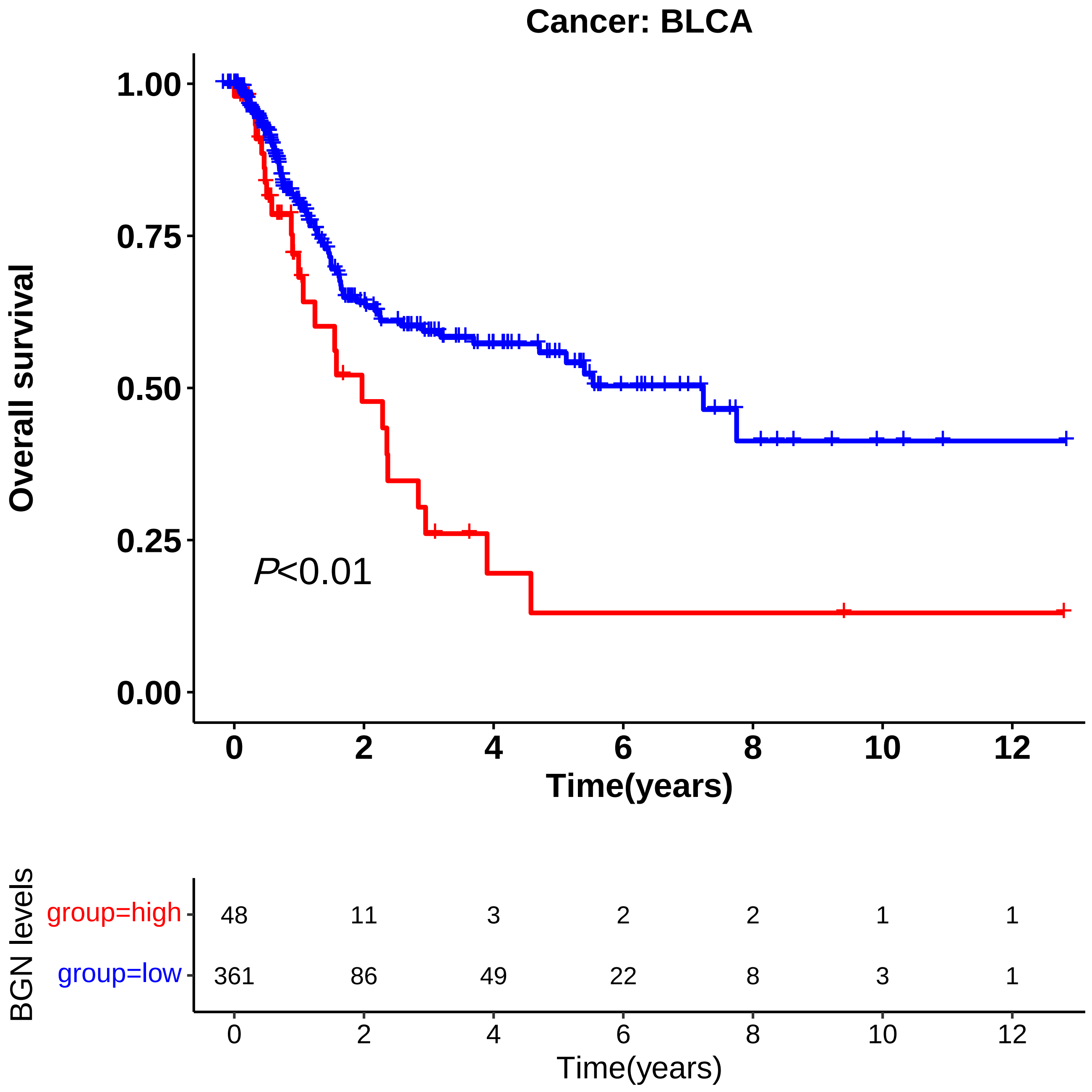

Cancer: BLCA
Overall survival
1.00
0.75
0.50
0.25
0.00
P<0.01
0
2
4
6
8
10
12
Time(years)
BGN levels
group=high
48 11 3 2 2 1 1
group=low
361 86 49 22 8 3 1
Time(years)

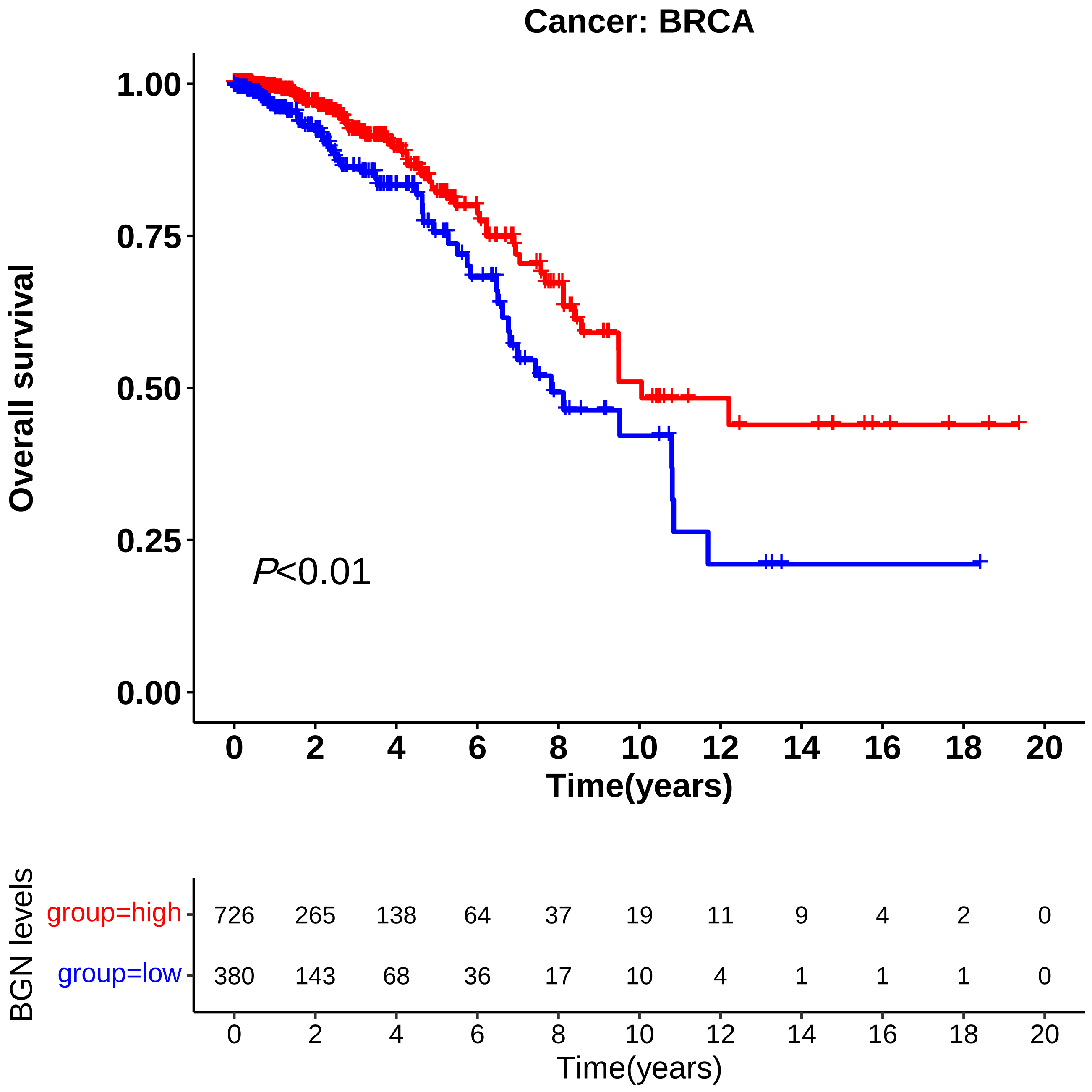

Cancer: BRCA
Overall survival
1.00
0.75
0.50
0.25
0.00
P<0.01
0
2
4
6
8
10
12
14
16
18
20
Time(years)
BGN levels
group=high
group=low
726 265 138 64 37 19 11 9 4 2 0
380 143 68 36 17 10 4 1 1 1 0
Time(years)

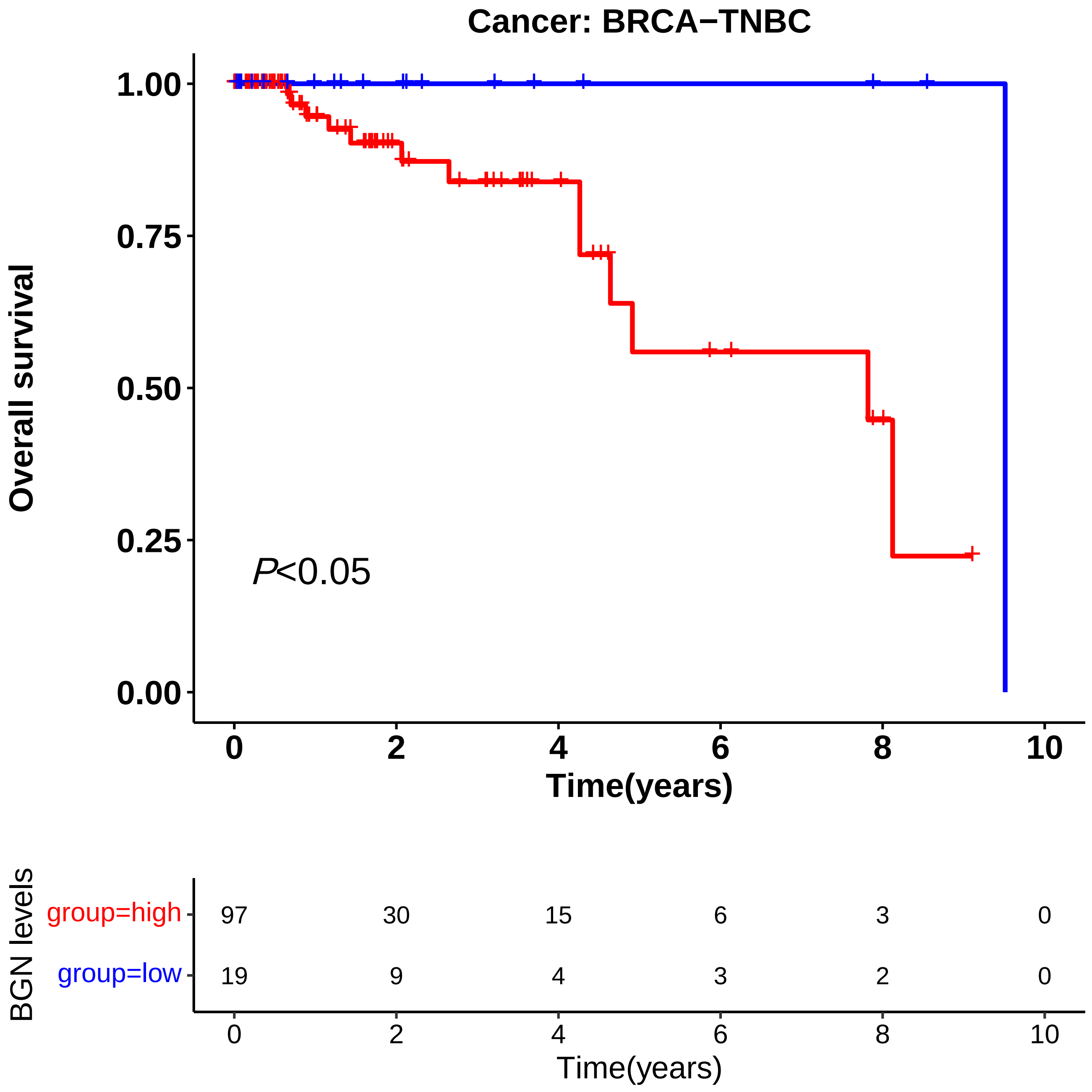
Cancer: BRCA−TNBC
Overall survival
1.00
0.75
0.50
0.25
0.00
P<0.05
0
2
4
6
8
10
Time(years)
BGN levels
group=high
group=low
97 30 15 6 3 0
19 9 4 3 2 0
0
2
4
6
8
10
Time(years)

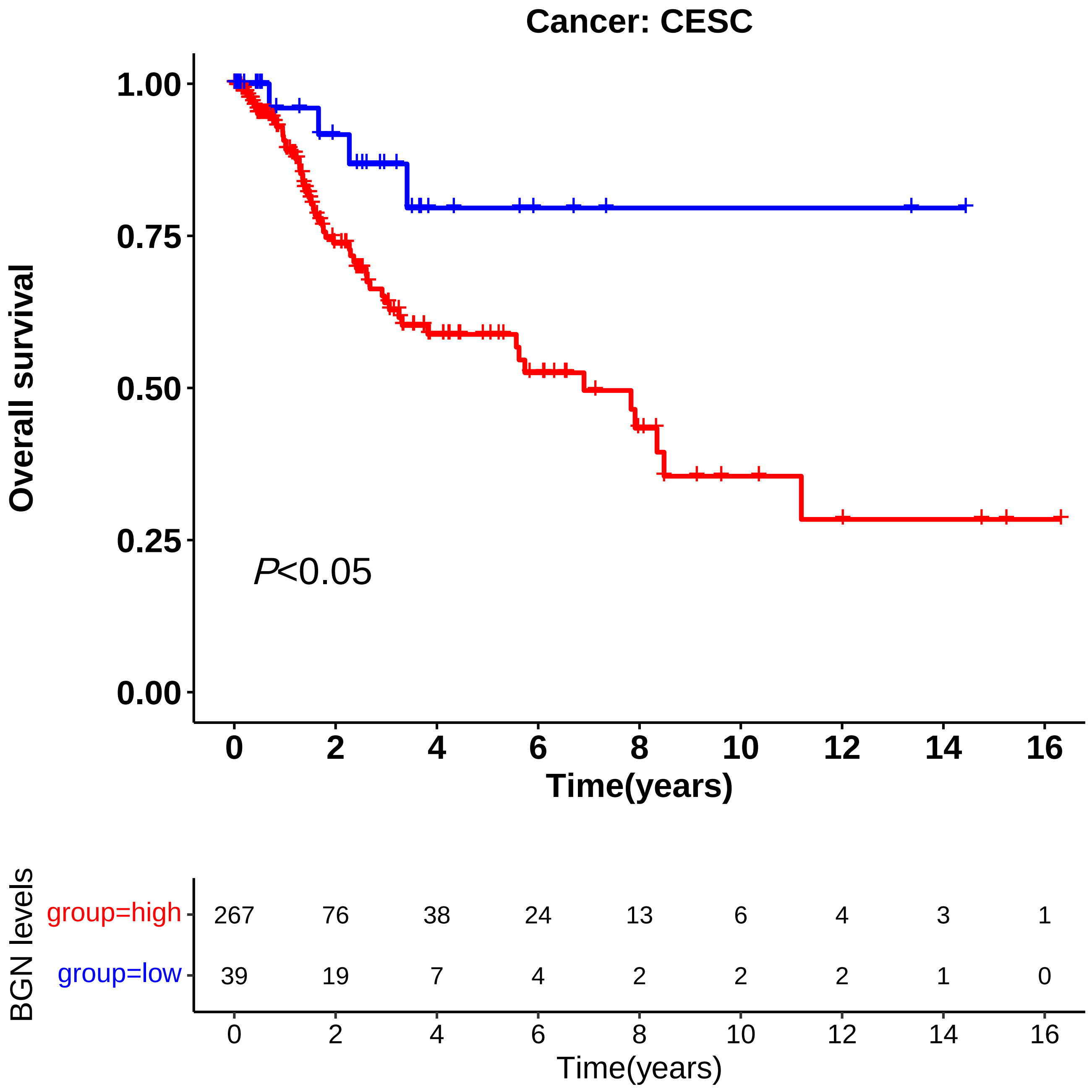
Cancer: CESC
Overall survival
1.00
0.75
0.50
0.25
0.00
P<0.05
0 2 4 6 8 10 12 14 16
Time(years)
BGN levels
group=high 267 76 38 24 13 6 4 3 1
group=low 39 19 7 4 2 2 2 1 0
0 2 4 6 8 10 12 14 16
Time(years)

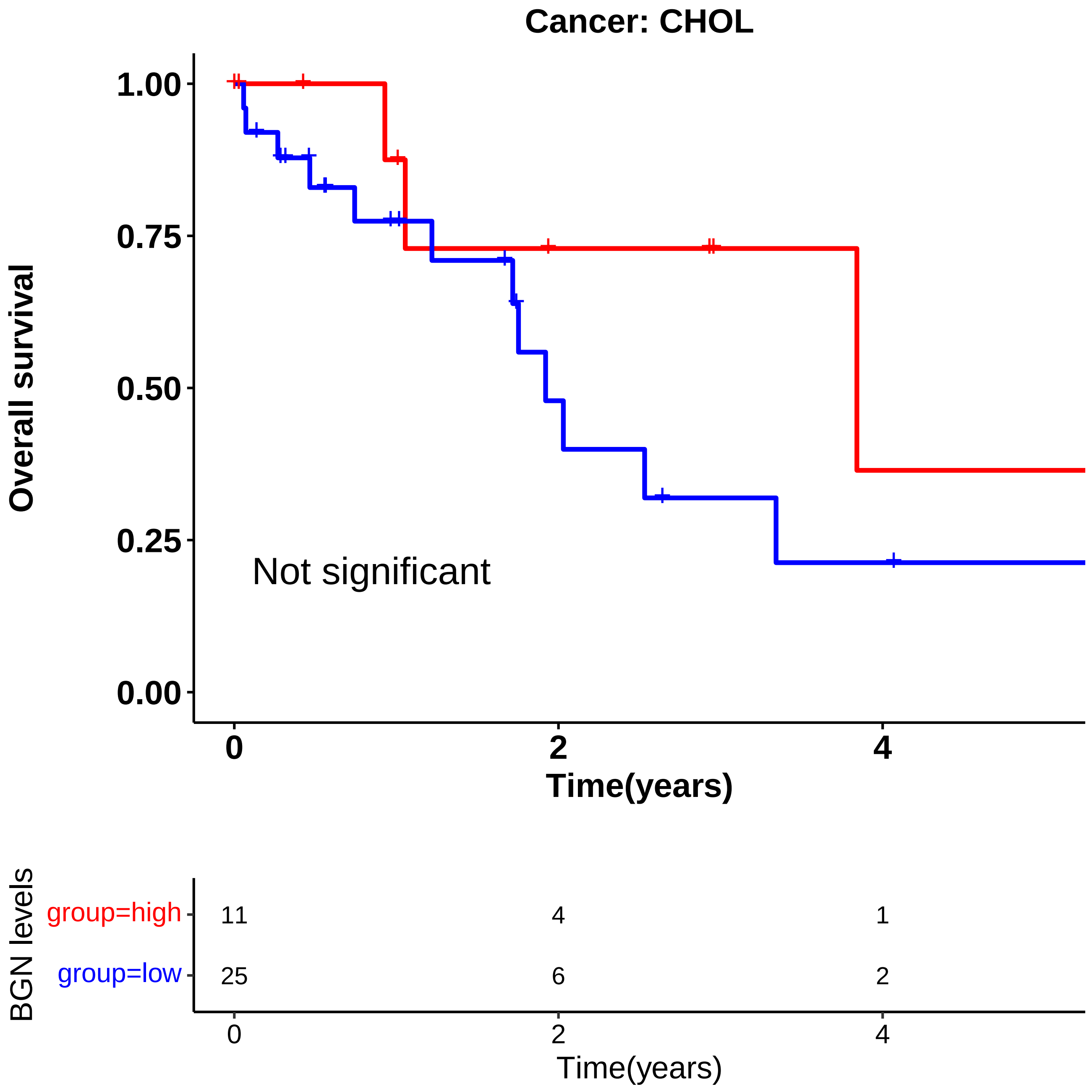
Cancer: CHOL
Overall survival
1.00
0.75
0.50
0.25
0.00
Not significant
0
2
4
Time(years)
BGN levels
group=high
group=low
11
4
1
25
6
2
0
2
4
Time(years)

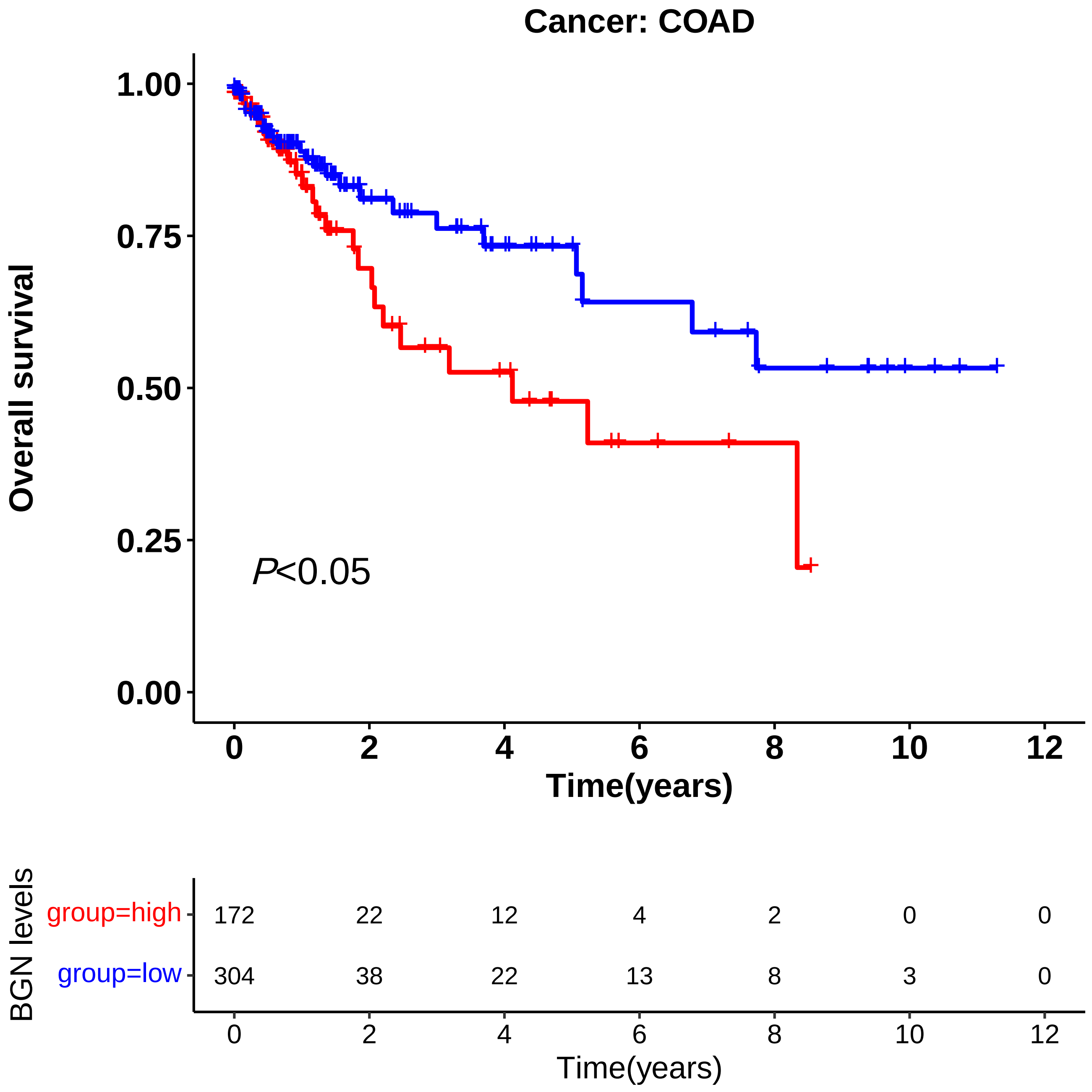
Cancer: COAD
Overall survival
1.00
0.75
0.50
0.25
0.00
P<0.05
0
2
4
6
8
10
12
Time(years)
BGN levels
group=high
group=low
172 22 12 4 2 0 0
304 38 22 13 8 3 0
Time(years)

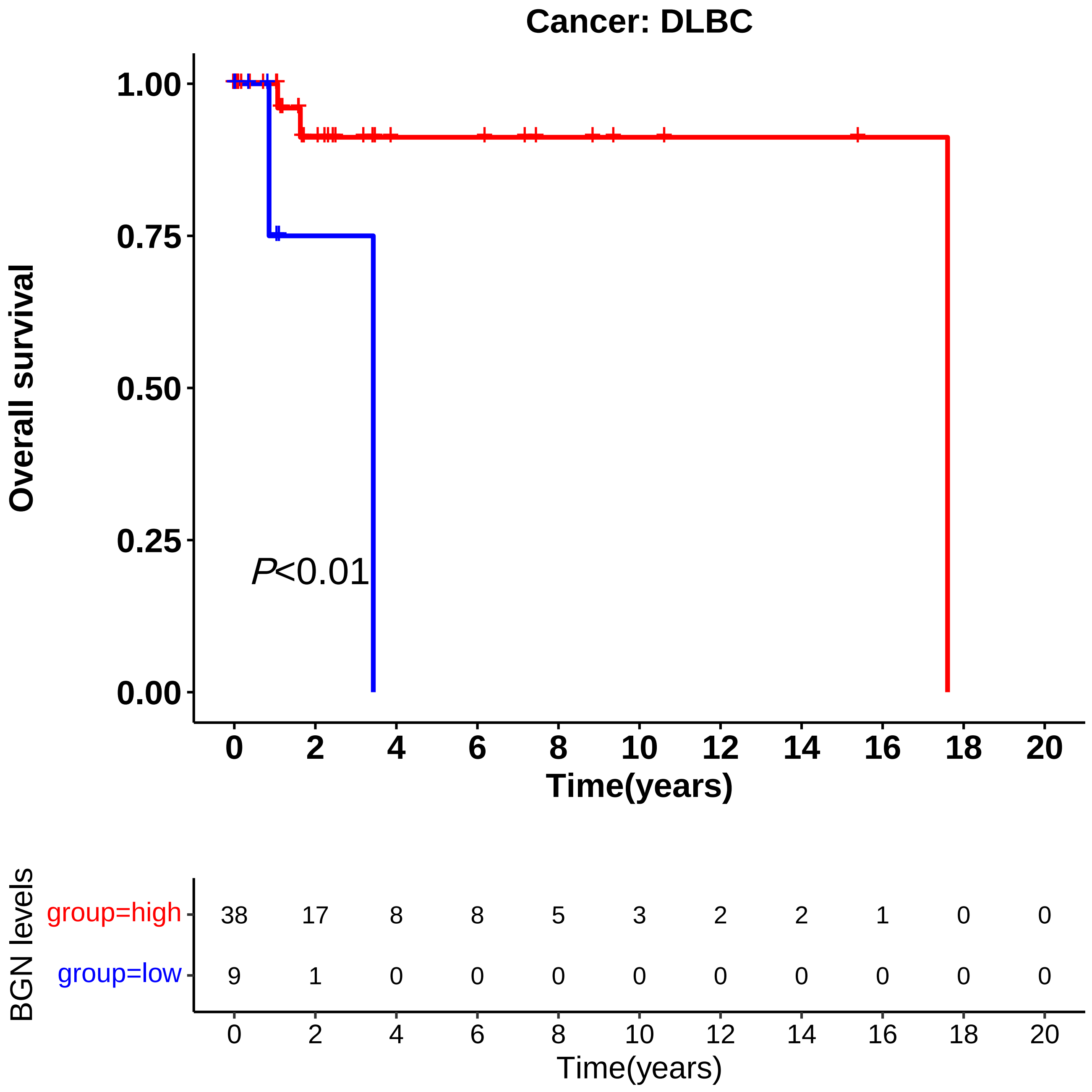

Cancer: DLBC
Overall survival
1.00
0.75
0.50
0.25
0.00
P<0.01
0
2
4
6
8
10
12
14
16
18
20
Time(years)
BGN levels
group=high
group=low
38 17 8 8 5 3 2 2 1 0 0
9 1 0 0 0 0 0 0 0 0 0
Time(years)

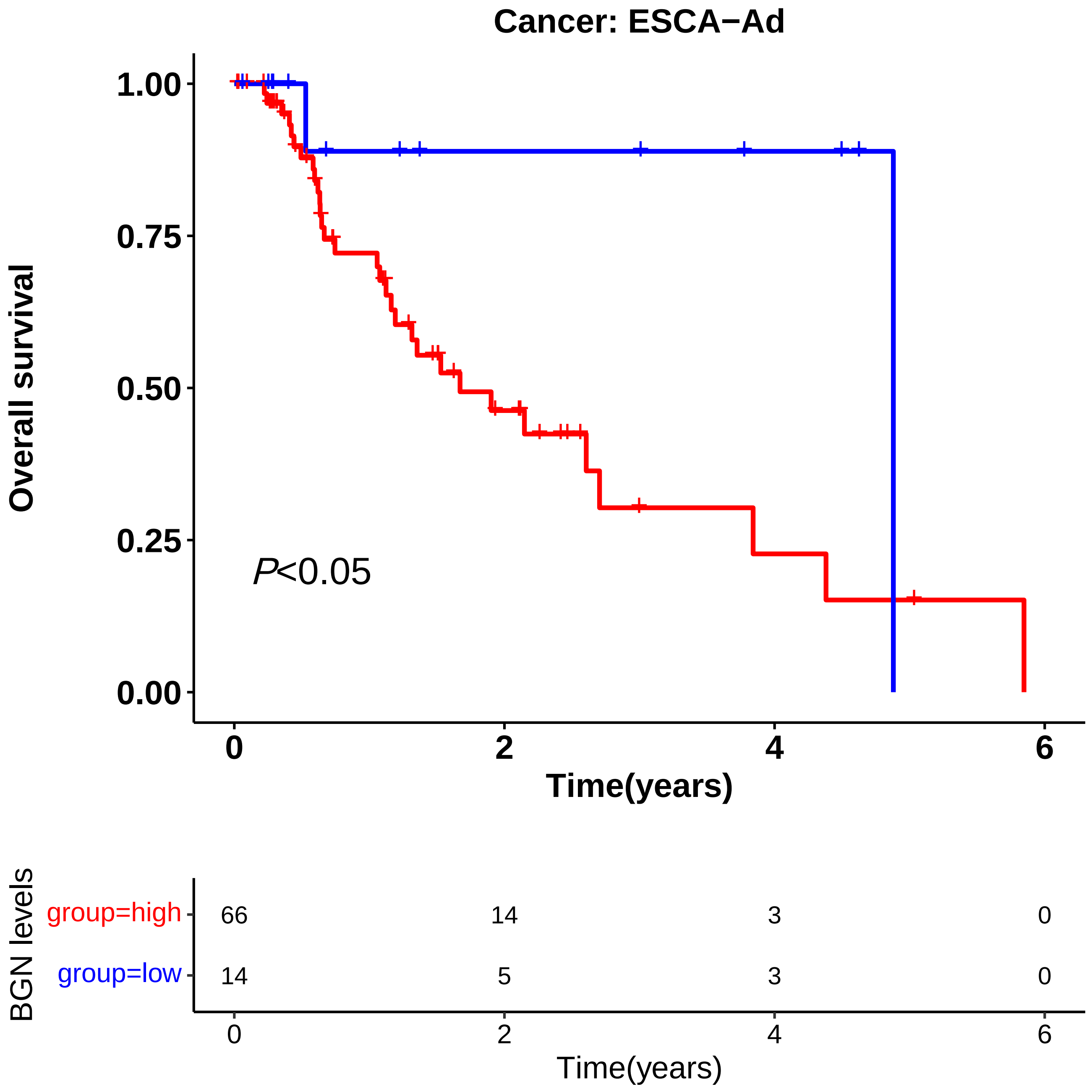

Cancer: ESCA−Ad
Overall survival
1.00
0.75
0.50
0.25
0.00
P<0.05
0
2
4
6
Time(years)
BGN levels
group=high
group=low
66
14
3
0
14
5
3
0
0
2
4
6
Time(years)

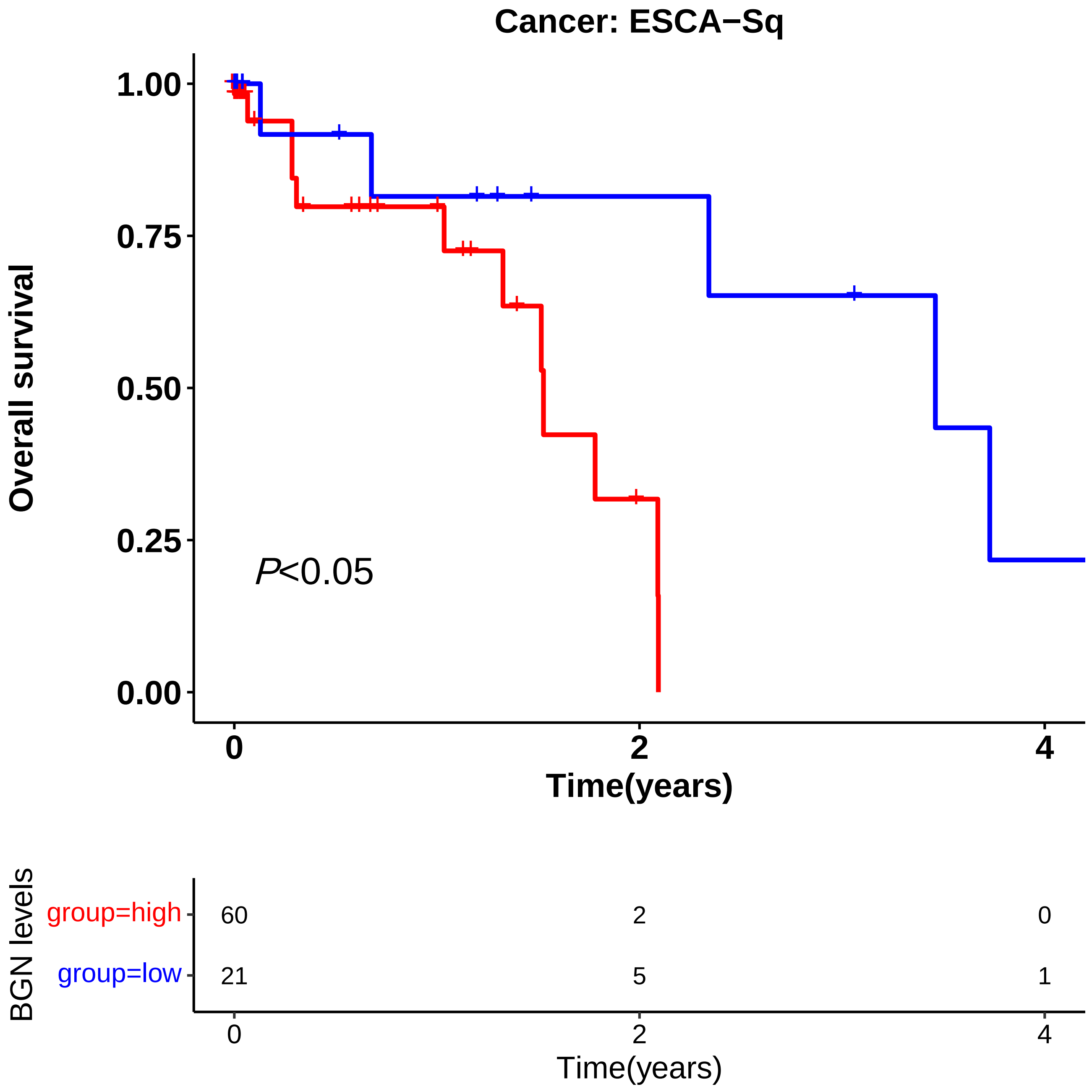
Cancer: ESCA−Sq
Overall survival
1.00
0.75
0.50
0.25
0.00
P<0.05
0
2
4
Time(years)
BGN levels
group=high
group=low
60
2
0
21
5
1
0
2
4
Time(years)

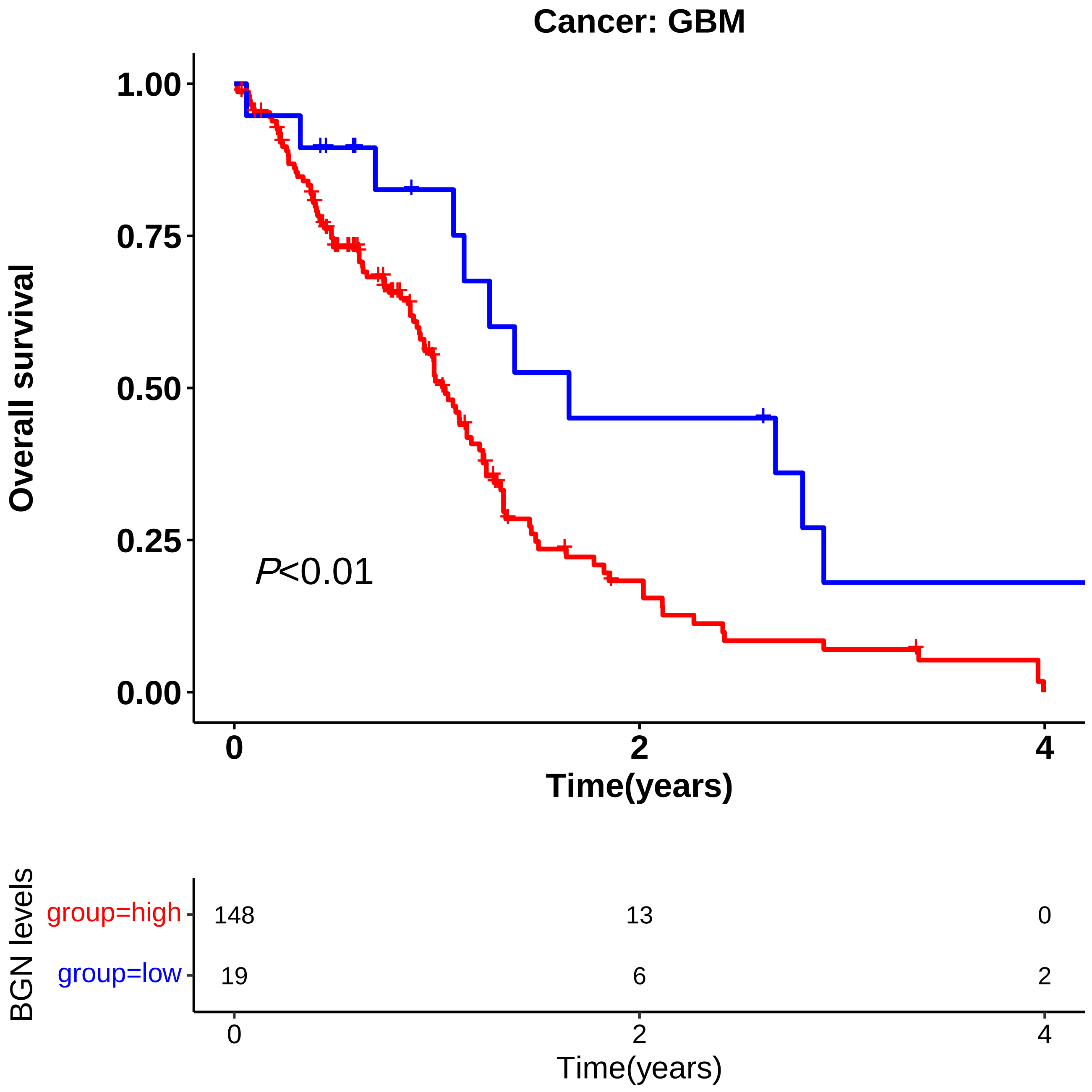

Cancer: GBM
Overall survival
1.00
0.75
0.50
0.25
0.00
P<0.01
0
2
4
Time(years)
BGN levels
group=high
group=low
148
13
0
19
6
2
0
2
4
Time(years)

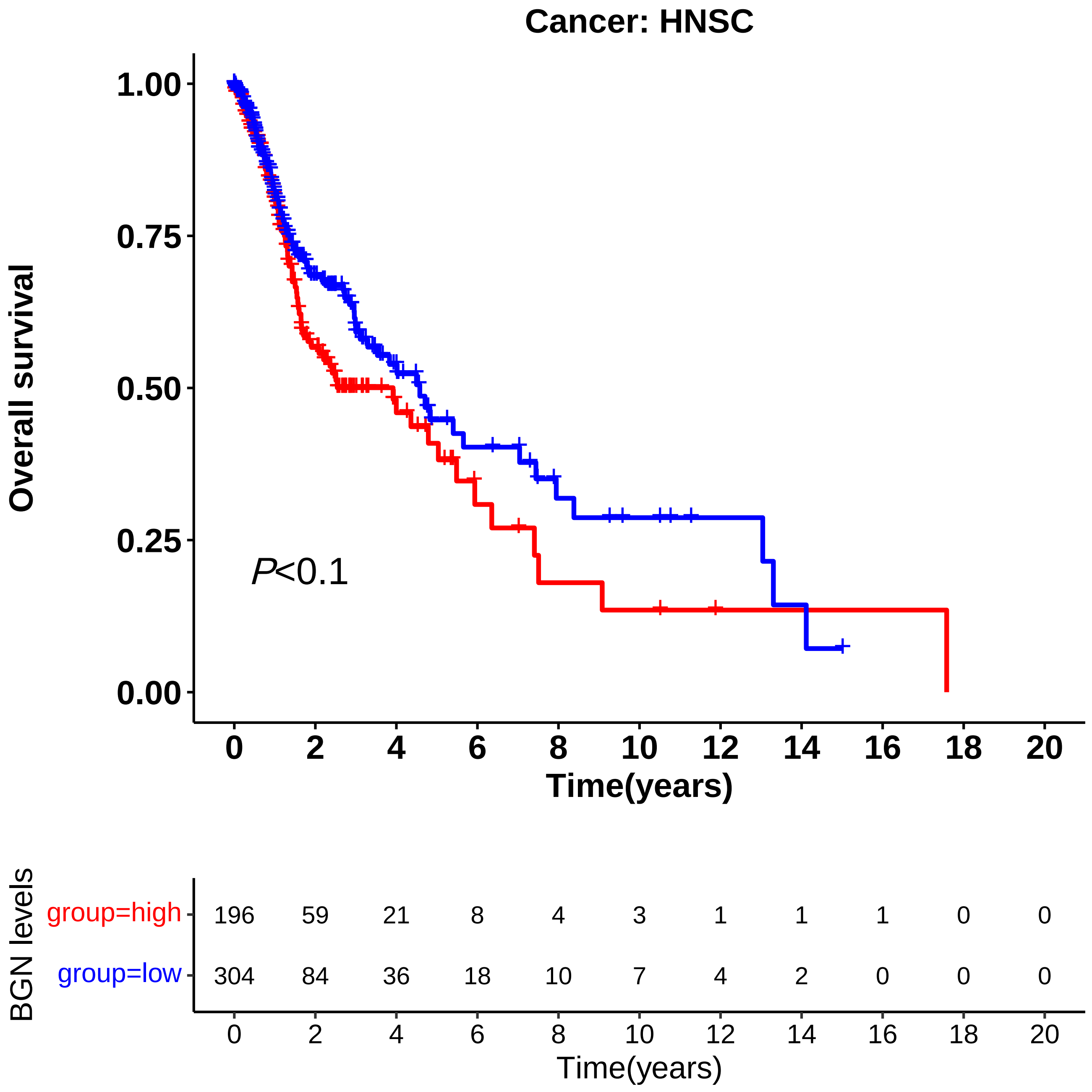
Cancer: HNSC
Overall survival
1.00
0.75
0.50
0.25
0.00
P<0.1
0 2 4 6 8 10 12 14 16 18 20
Time(years)
BGN levels
group=high 196 59 21 8 4 3 1 1 1 0 0
group=low 304 84 36 18 10 7 4 2 0 0 0
0 2 4 6 8 10 12 14 16 18 20
Time(years)

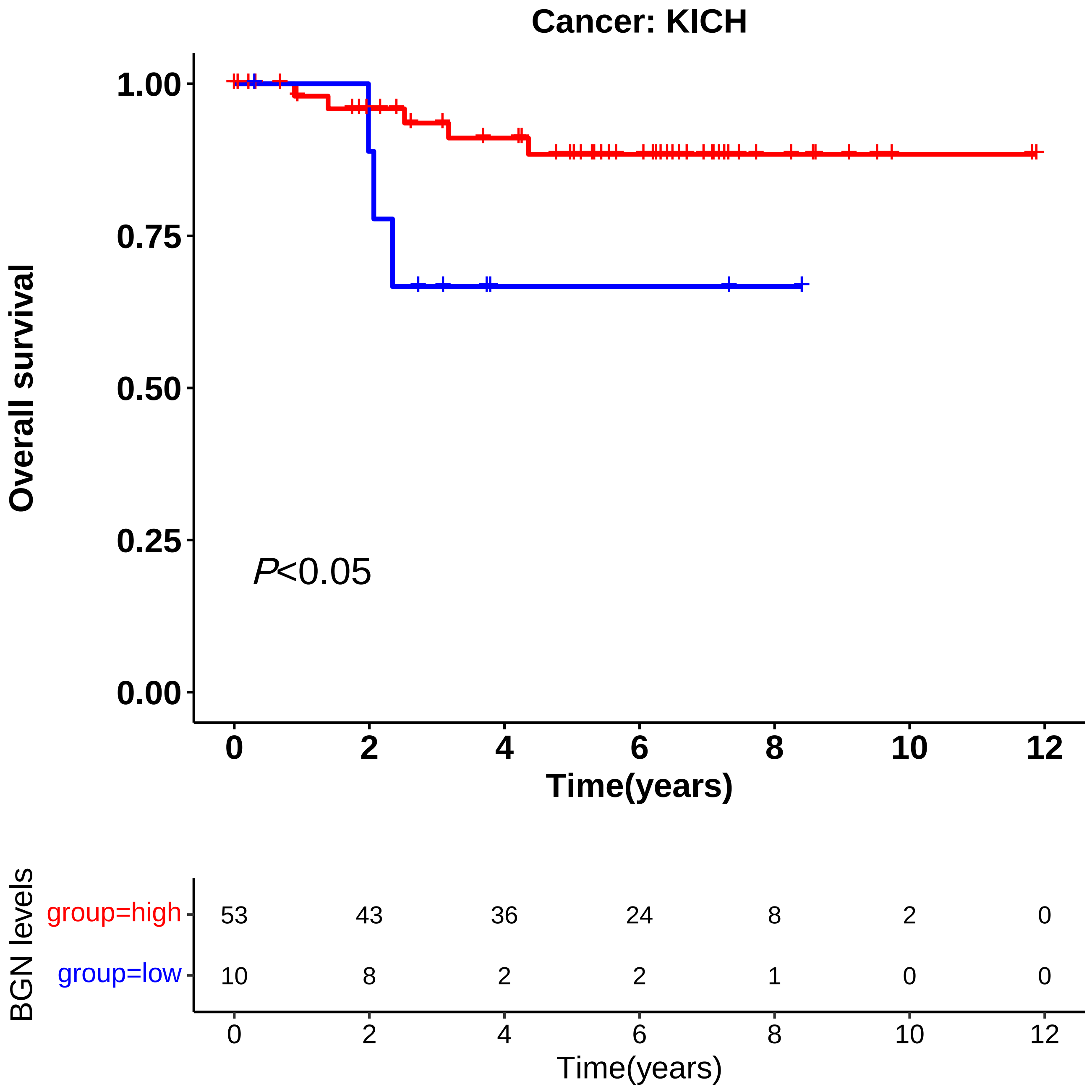

Cancer: KICH
Overall survival
1.00
0.75
0.50
0.25
0.00
P<0.05
0
2
4
6
8
10
12
Time(years)
BGN levels
group=high
group=low
53 43 36 24 8 2 0
10 8 2 2 1 0 0
Time(years)

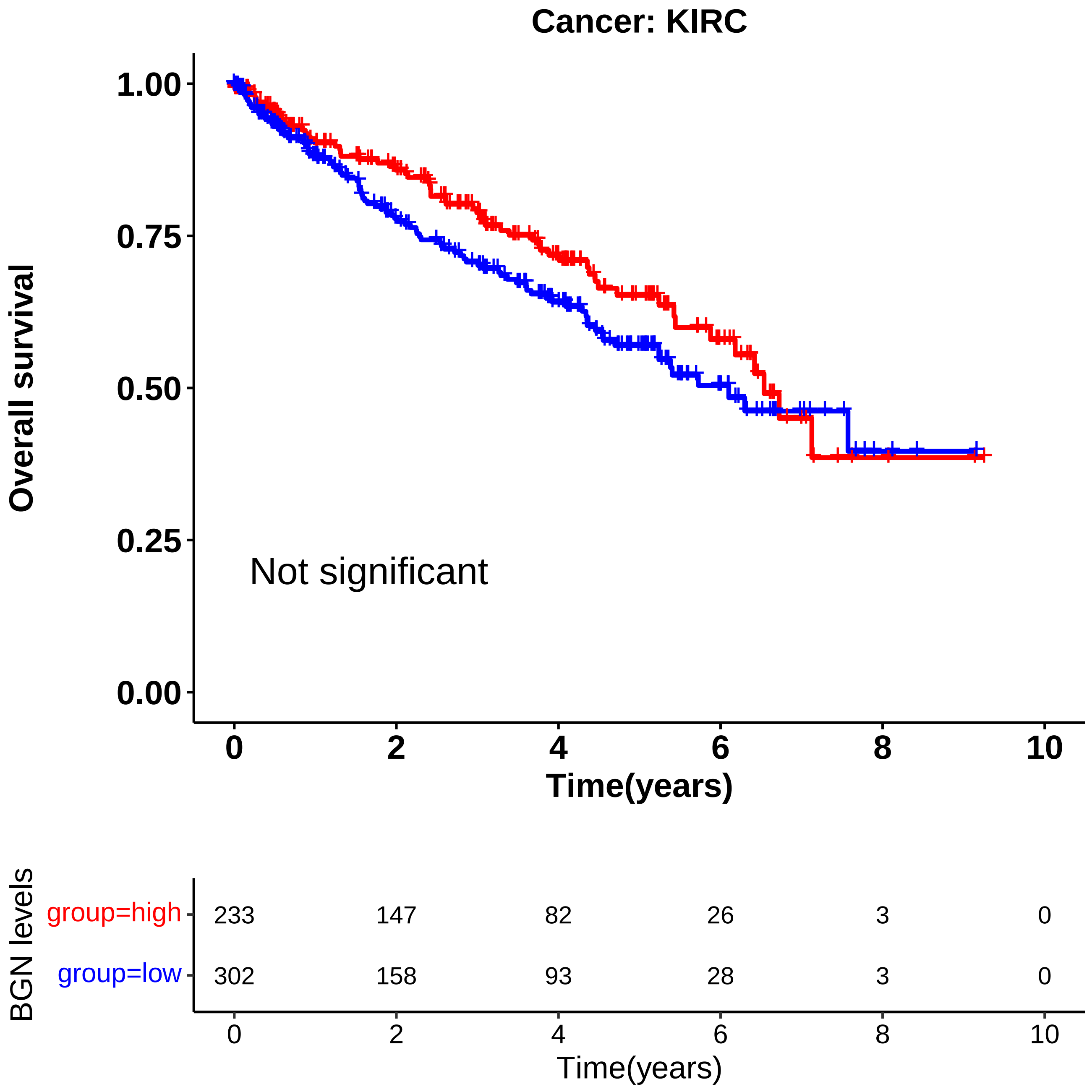

Cancer: KIRC
Overall survival
1.00
0.75
0.50
0.25
0.00
Not significant
0
2
4
6
8
10
Time(years)
BGN levels
group=high
233
147
82
26
3
0
group=low
302
158
93
28
3
0
0
2
4
6
8
10
Time(years)

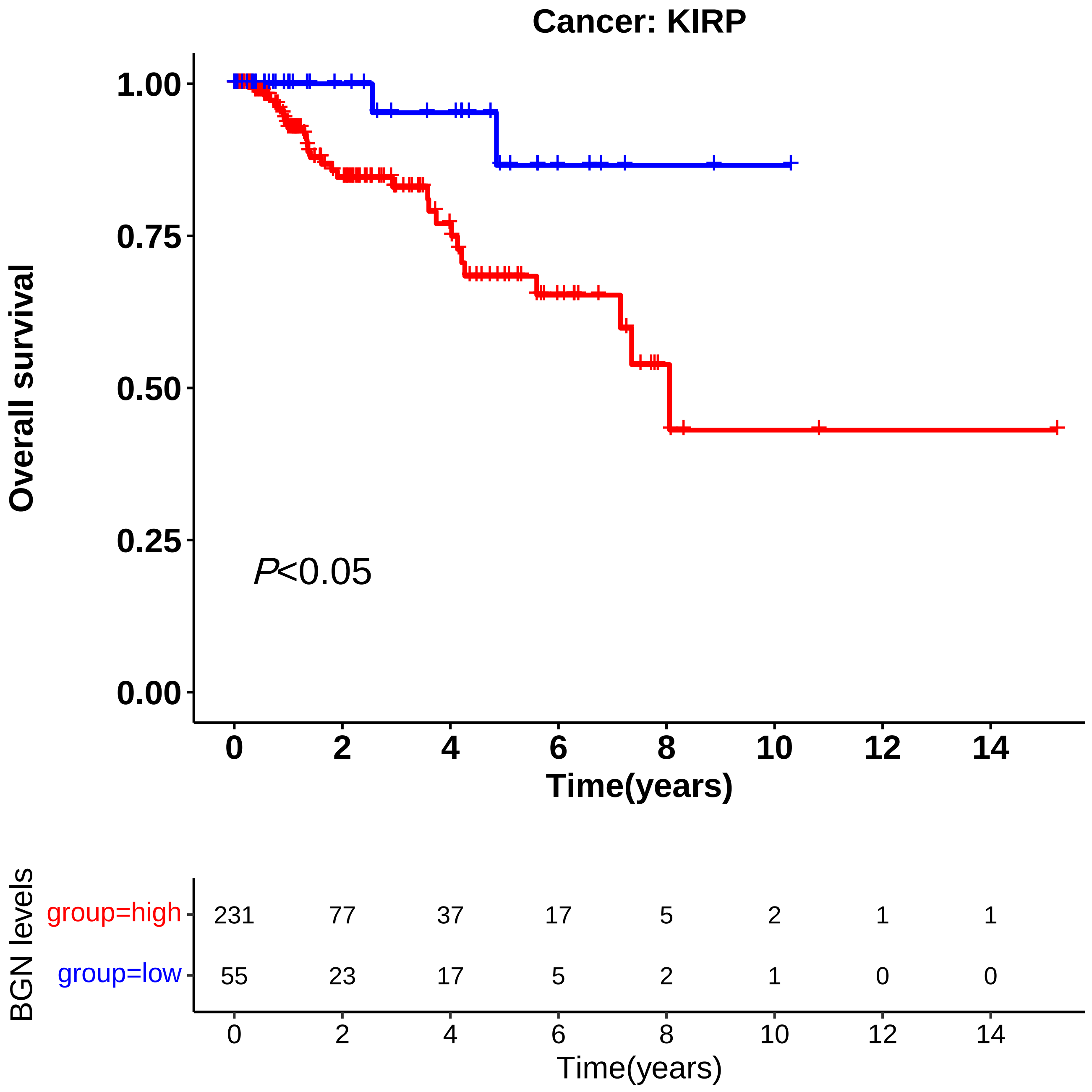
Cancer: KIRP
Overall survival
1.00
0.75
0.50
0.25
0.00
P<0.05
0
2
4
6
8
10
12
14
Time(years)
BGN levels
group=high
group=low
231 77 37 17 5 2 1 1
55 23 17 5 2 1 0 0
Time(years)

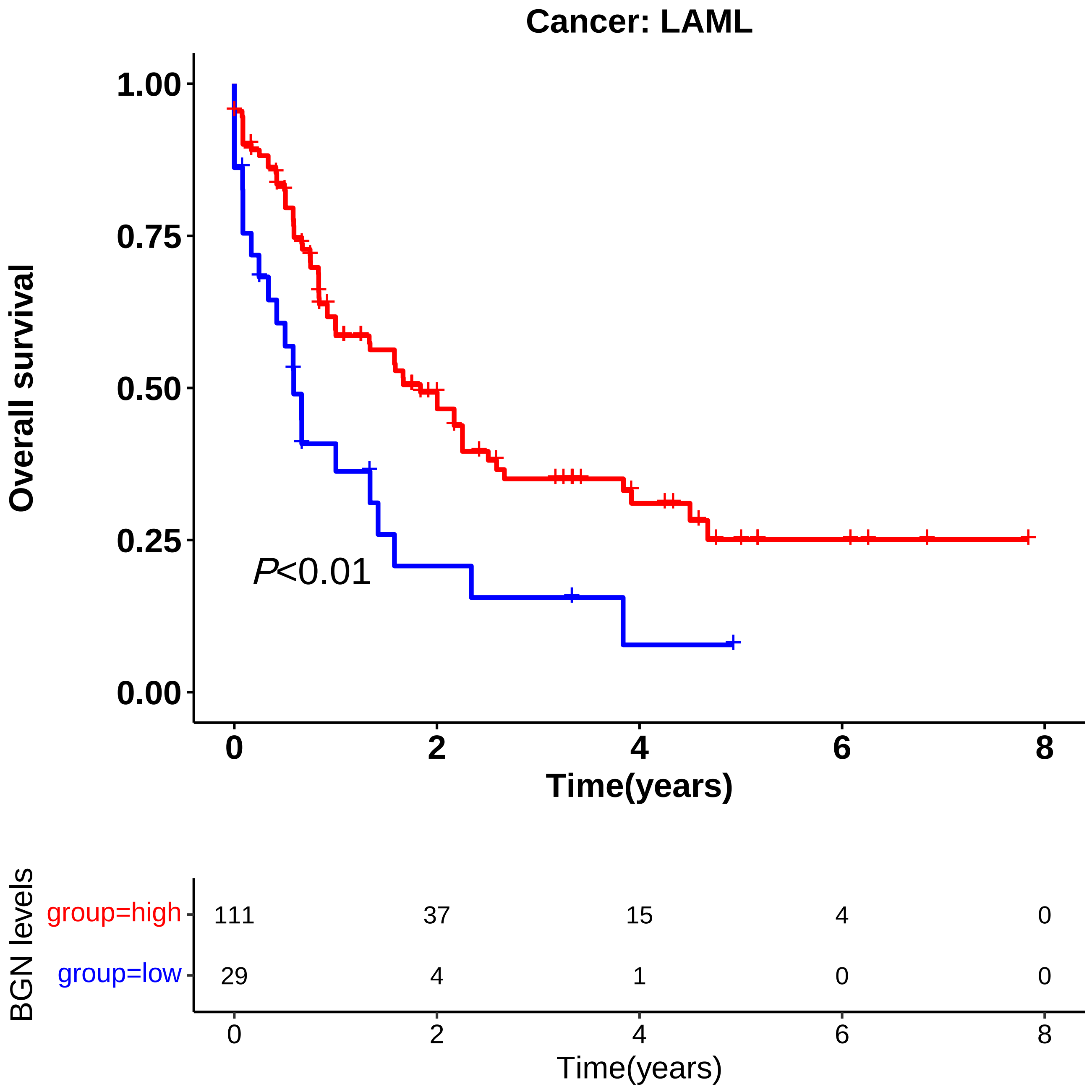

Cancer: LAML
Overall survival
1.00
0.75
0.50
0.25
0.00
P<0.01
0
2
4
6
8
Time(years)
BGN levels
group=high
group=low
111
37
15
4
0
29
4
1
0
0
0
2
4
6
8
Time(years)

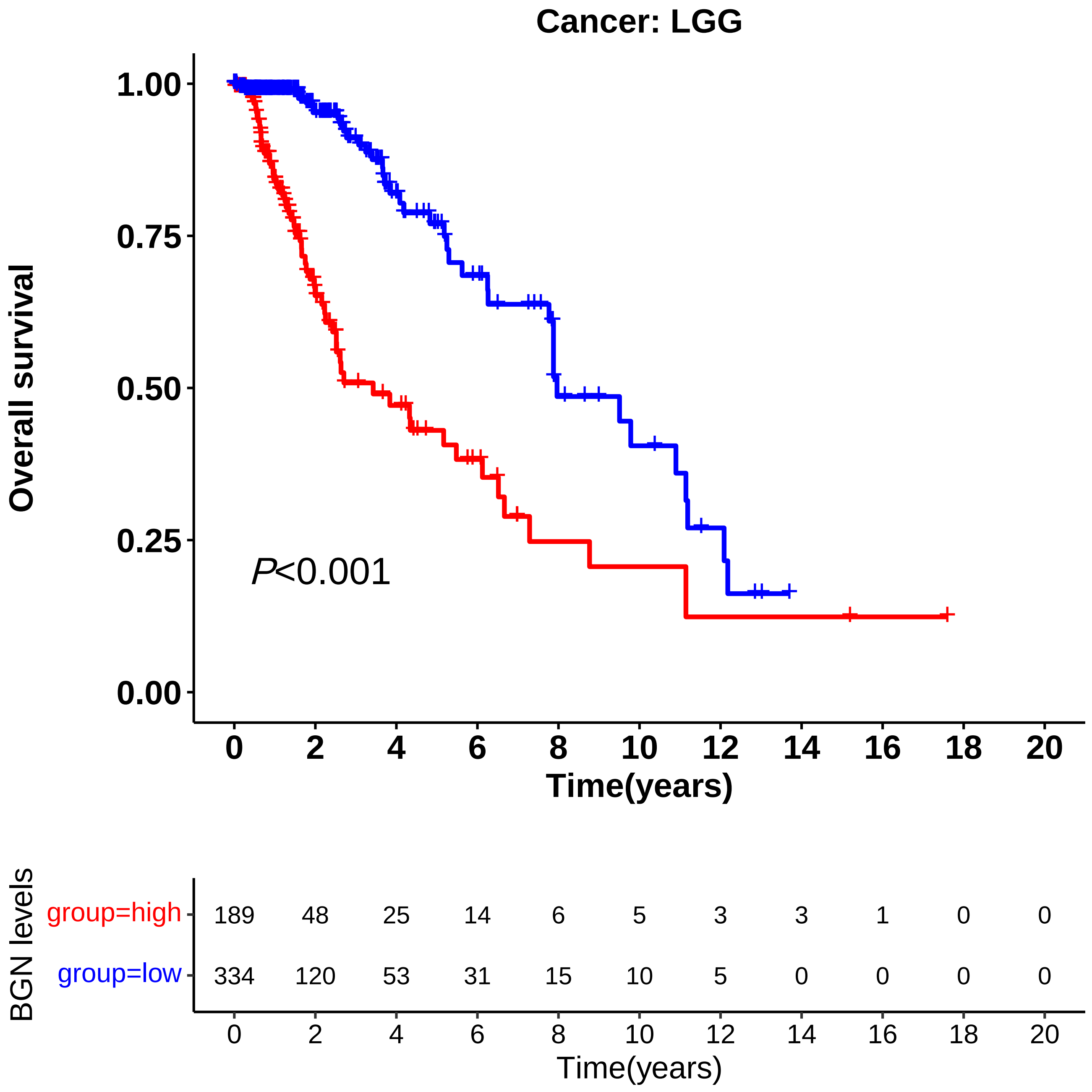

Cancer: LGG
Overall survival
1.00
0.75
0.50
0.25
0.00
P<0.001
0
2
4
6
8
10
12
14
16
18
20
Time(years)
BGN levels
group=high
group=low
189 48 25 14 6 5 3 3 1 0 0
334 120 53 31 15 10 5 0 0 0 0
Time(years)

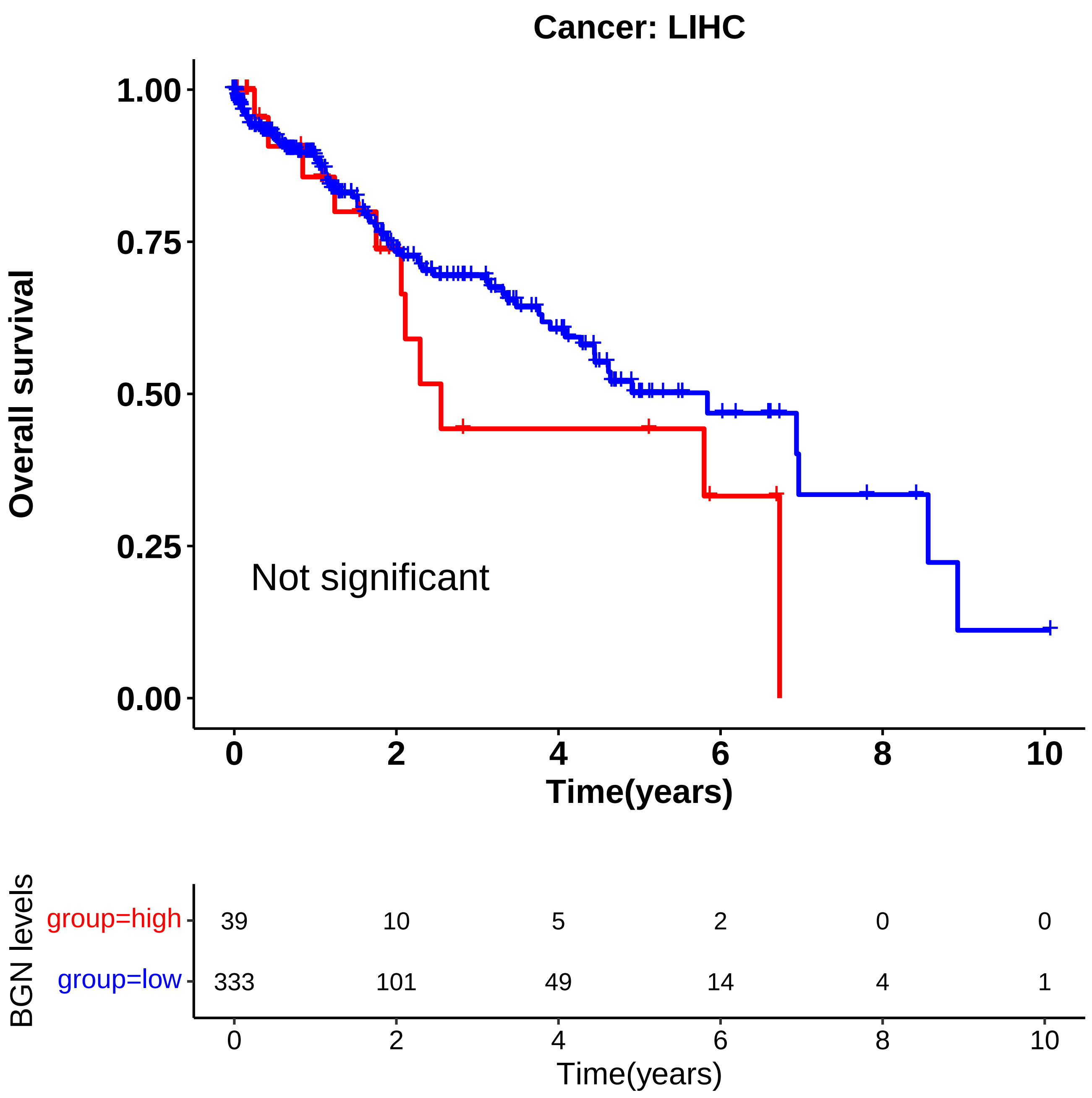

Cancer: LIHC
1.00
0.75
0.50
0.25
0.00
Overall survival
Not significant
0
2
4
6
8
10
Time(years)
BGN levels
group=high
group=low
39
10
5
2
0
0
333
101
49
14
4
1
0
2
4
6
8
10
Time(years)

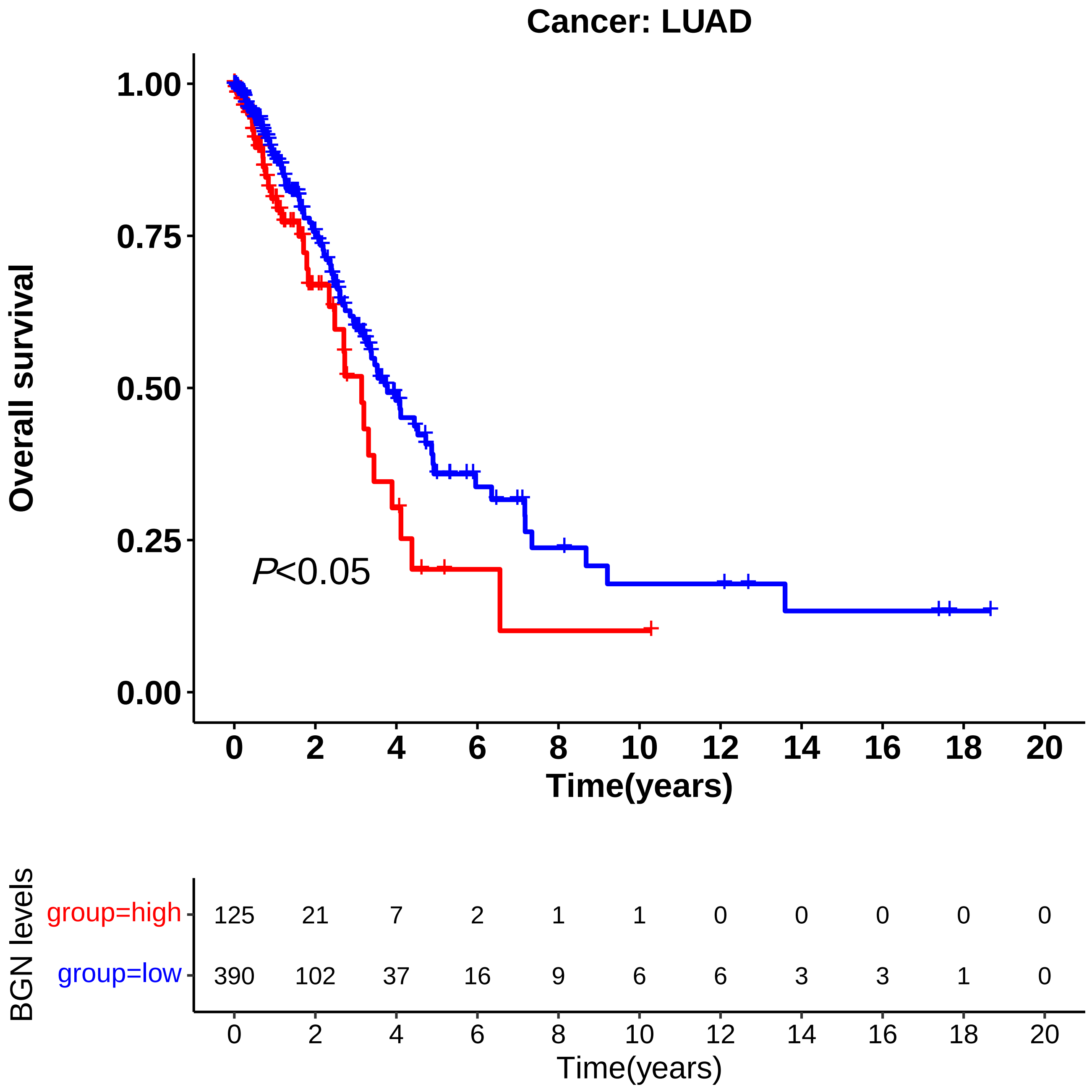

Cancer: LUAD
Overall survival
1.00
0.75
0.50
0.25
0.00
P<0.05
0 2 4 6 8 10 12 14 16 18 20
Time(years)
BGN levels
group=high 125 21 7 2 1 1 0 0 0 0 0
group=low 390 102 37 16 9 6 6 3 3 1 0
0 2 4 6 8 10 12 14 16 18 20
Time(years)

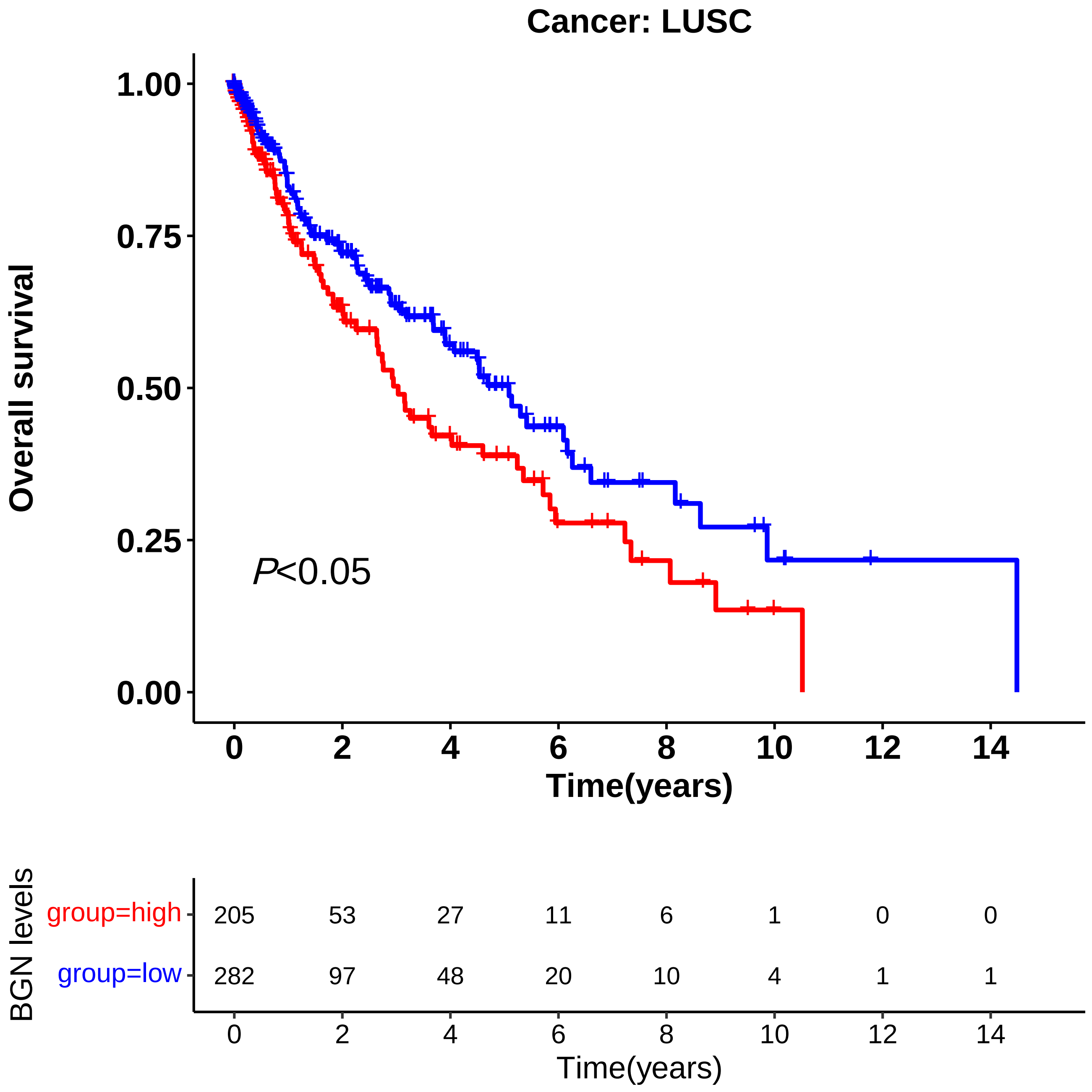

Cancer: LUSC
Overall survival
1.00
0.75
0.50
0.25
0.00
P<0.05
0
2
4
6
8
10
12
14
Time(years)
BGN levels
group=high
205 53 27 11 6 1 0 0
group=low
282 97 48 20 10 4 1 1
0
2
4
6
8
10
12
14
Time(years)

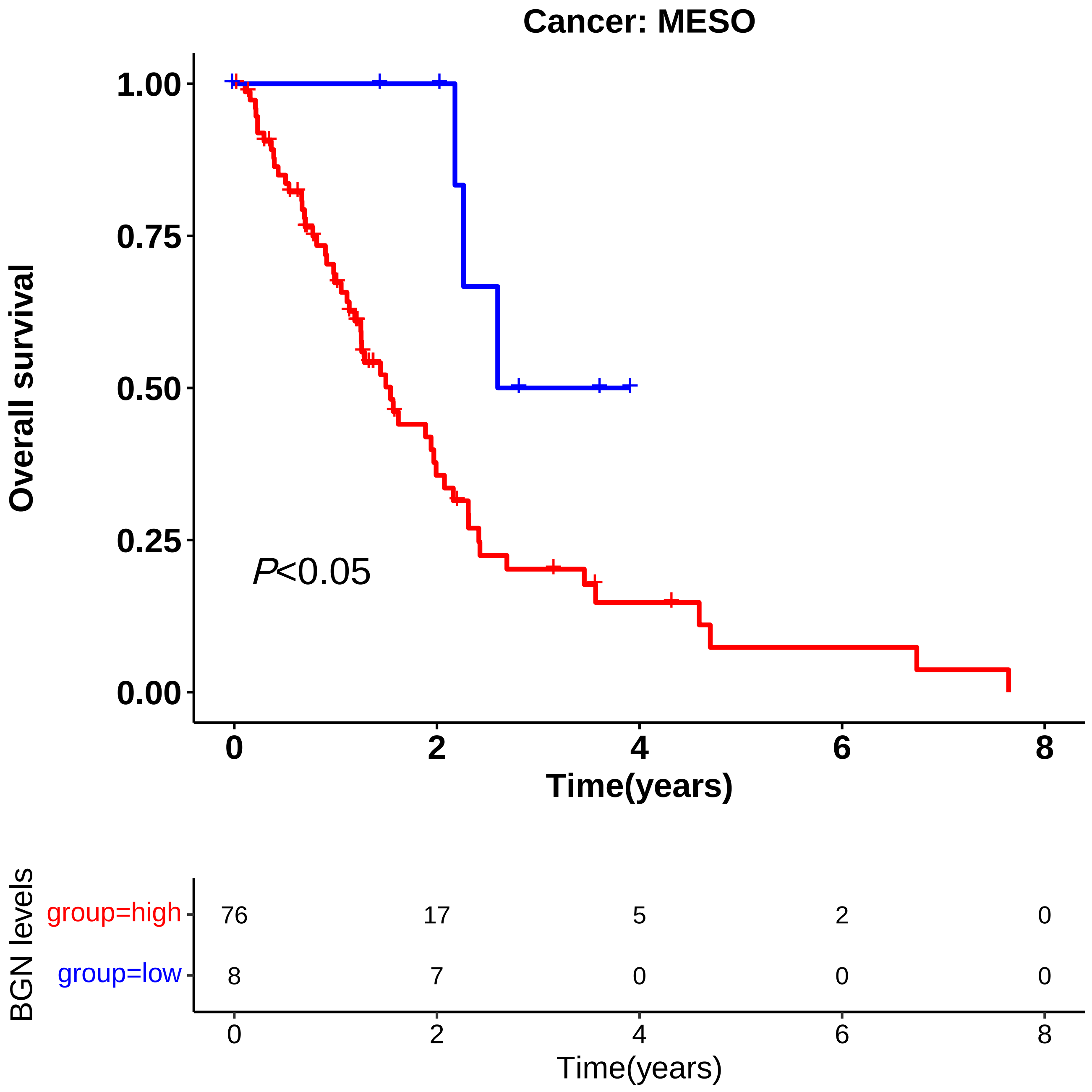

Cancer: MESO
Overall survival
1.00
0.75
0.50
0.25
0.00
P<0.05
0
2
4
6
8
Time(years)
BGN levels
group=high
76
17
5
2
0
group=low
8
7
0
0
0
0
2
4
6
8
Time(years)

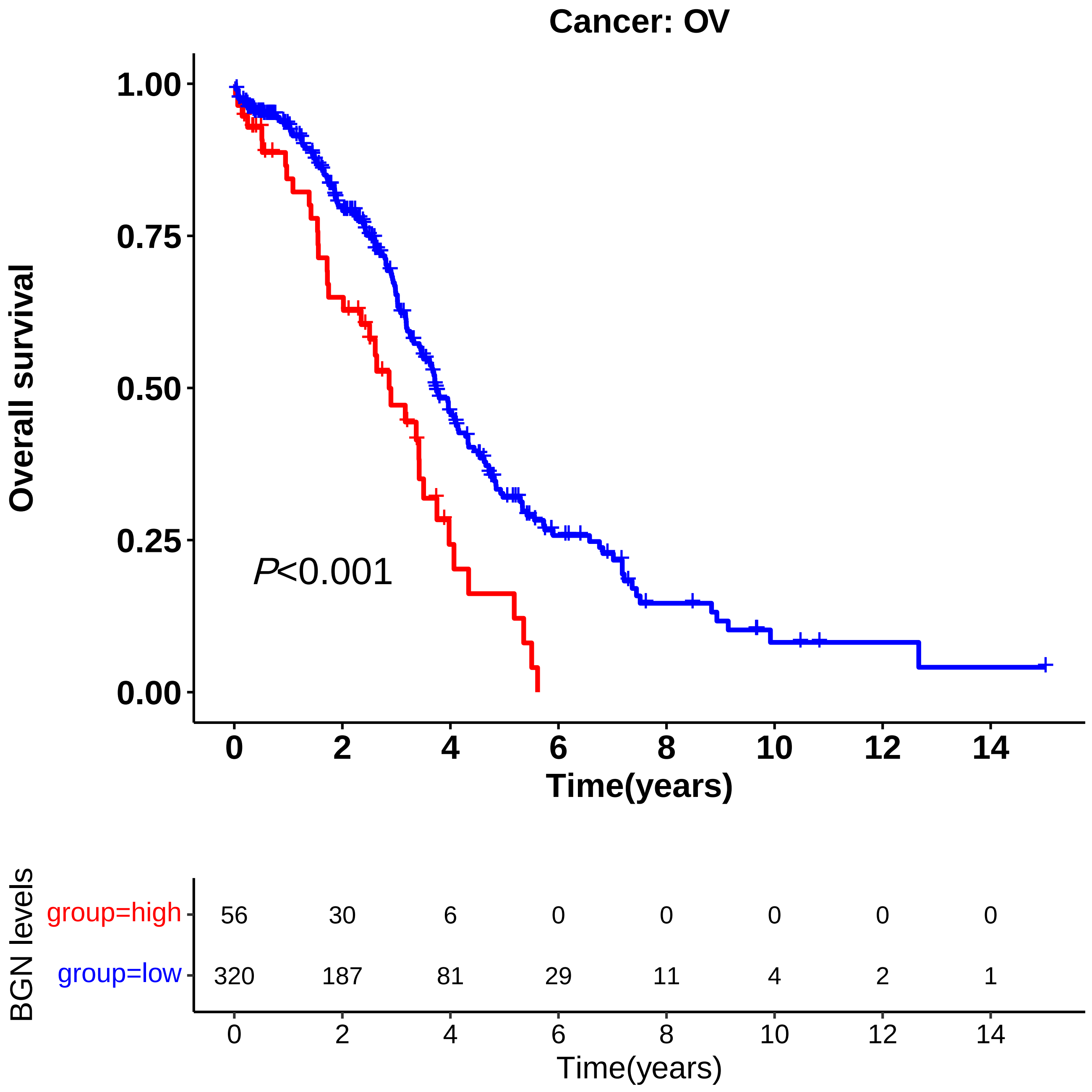

Cancer: OV
Overall survival
1.00
0.75
0.50
0.25
0.00
P<0.001
0
2
4
6
8
10
12
14
Time(years)
BGN levels
group=high
group=low
56 30 6 0 0 0 0 0
320 187 81 29 11 4 2 1
Time(years)

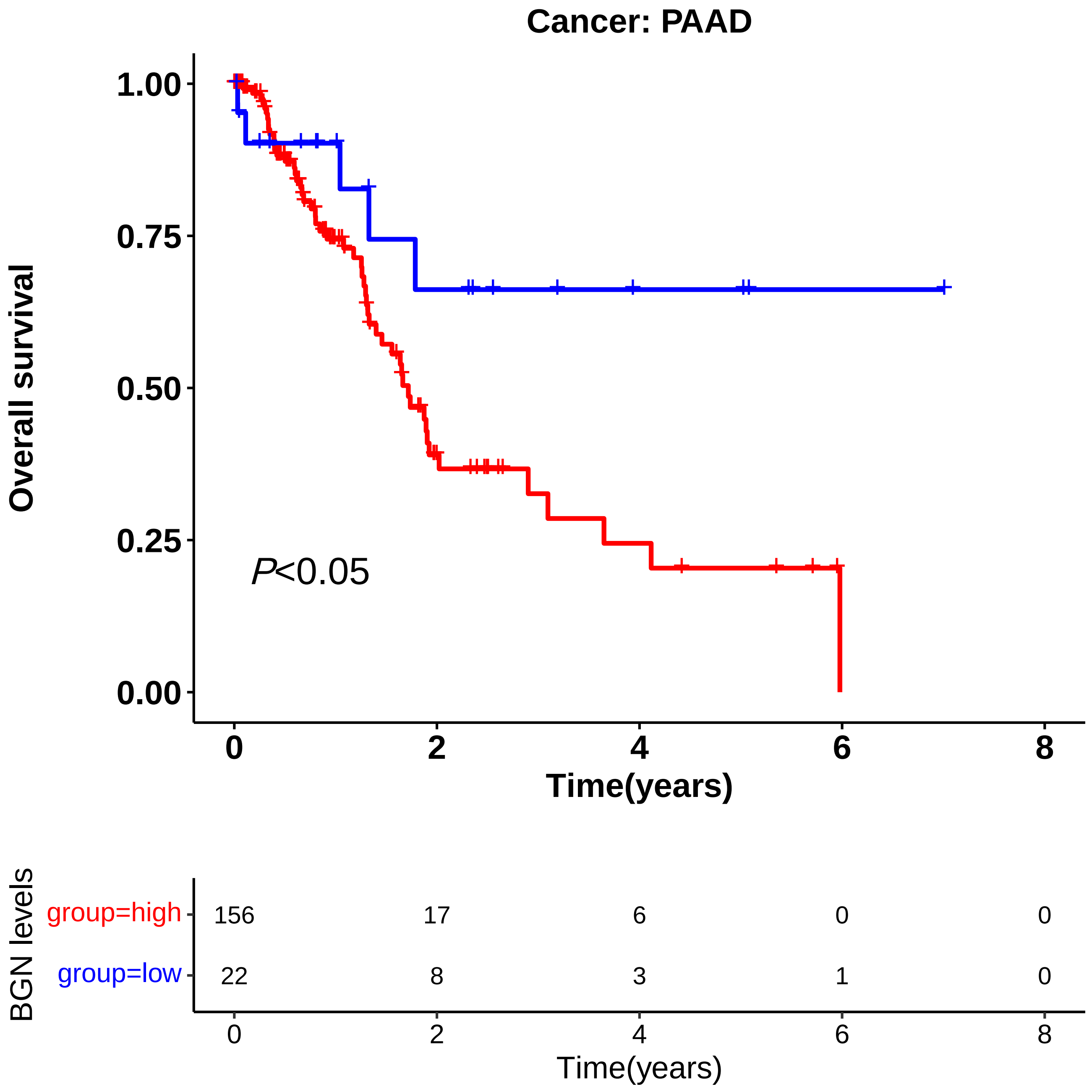

Cancer: PAAD
Overall survival
1.00
0.75
0.50
0.25
0.00
P<0.05
0
2
4
6
8
Time(years)
BGN levels
group=high
group=low
156
17
6
0
0
22
8
3
1
0
0
2
4
6
8
Time(years)

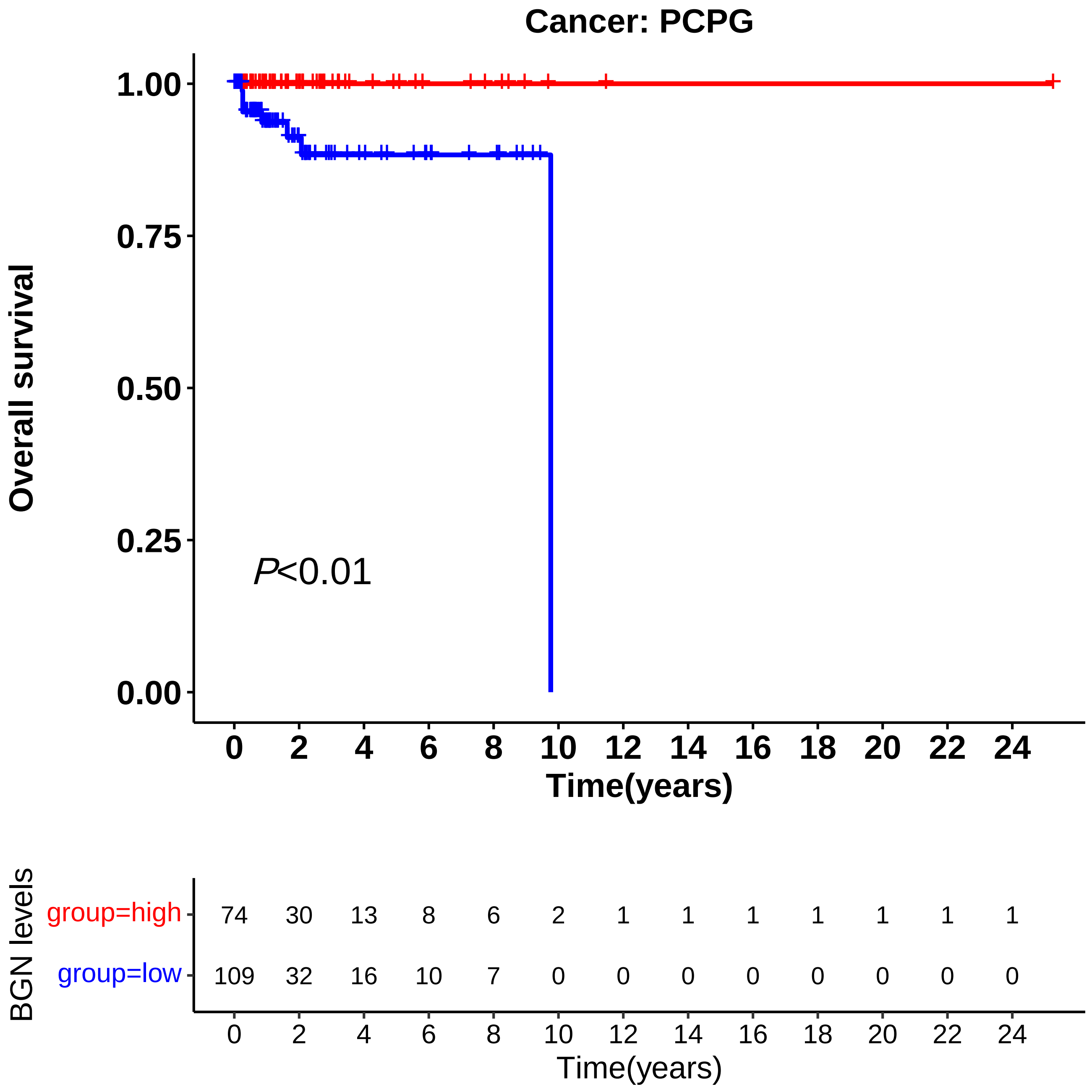
Cancer: PCPG
Overall survival
1.00
0.75
0.50
0.25
0.00
P<0.01
0 2 4 6 8 10 12 14 16 18 20 22 24
Time(years)
BGN levels
group=high 74 30 13 8 6 2 1 1 1 1 1 1 1
group=low 109 32 16 10 7 0 0 0 0 0 0 0 0
0 2 4 6 8 10 12 14 16 18 20 22 24
Time(years)

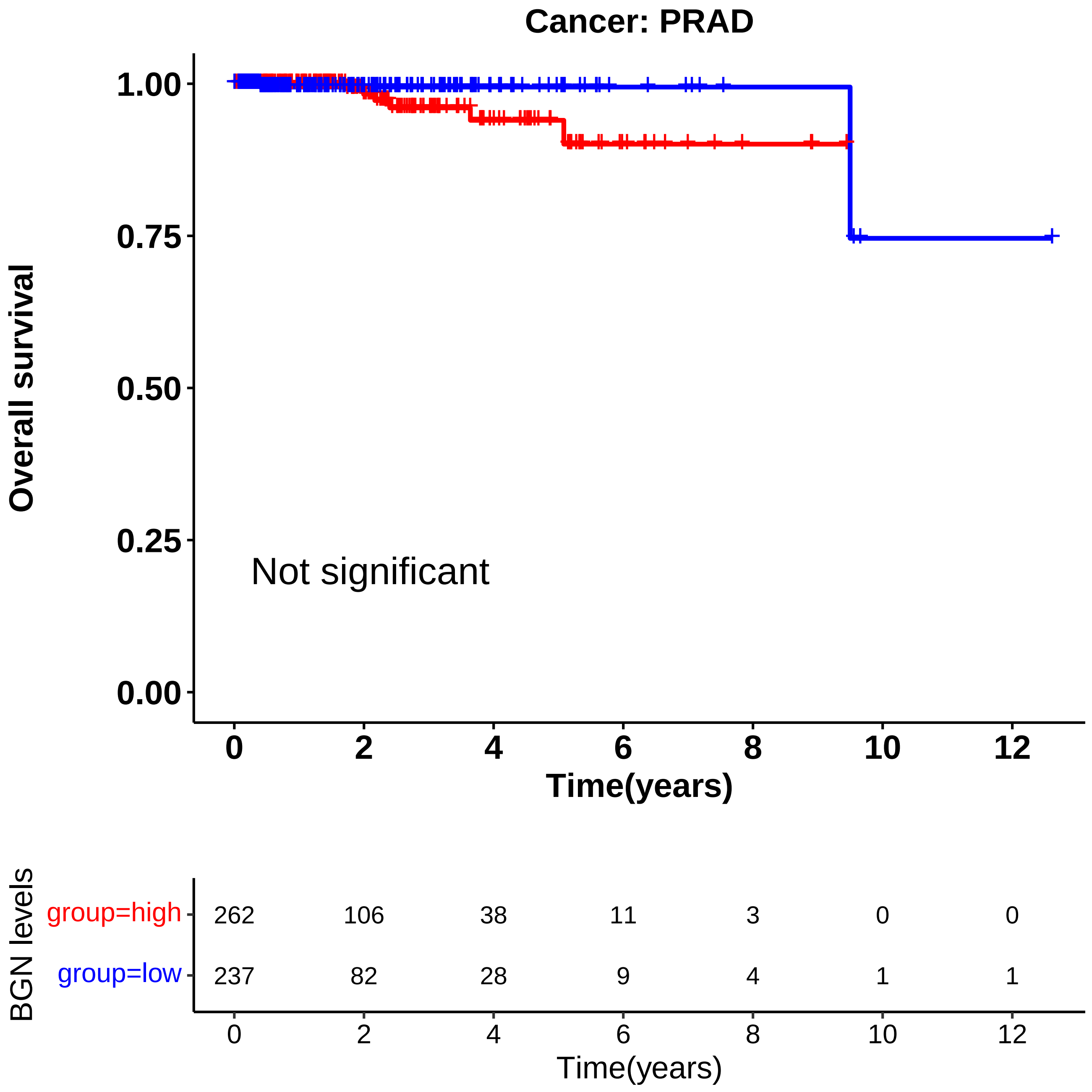

Cancer: PRAD
Overall survival
1.00
0.75
0.50
0.25
0.00
Not significant
0
2
4
6
8
10
12
Time(years)
BGN levels
group=high
262 106 38 11 3 0 0
group=low
237 82 28 9 4 1 1
0
2
4
6
8
10
12
Time(years)

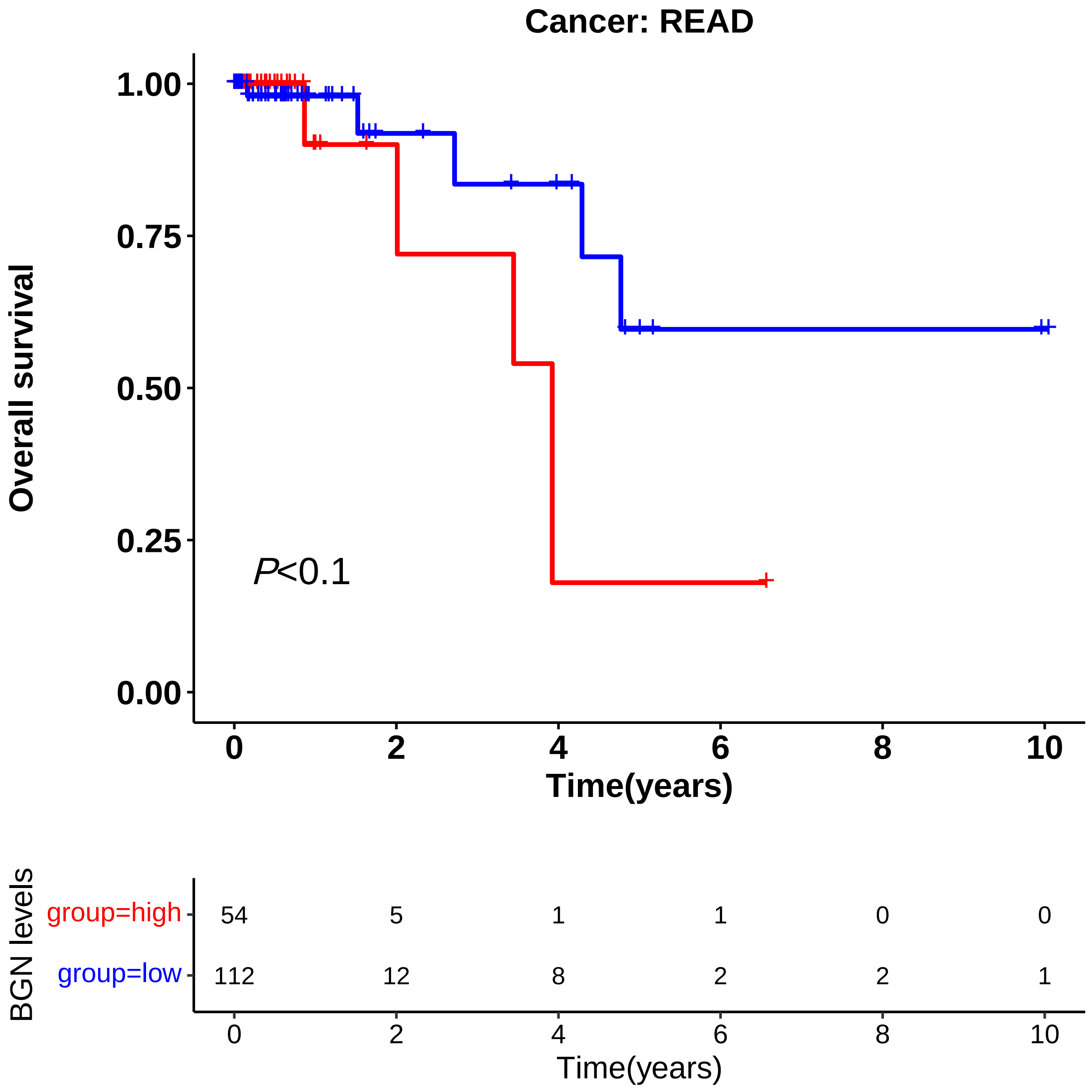

Cancer: READ
Overall survival
1.00
0.75
0.50
0.25
0.00
P<0.1
0
2
4
6
8
10
Time(years)
BGN levels
group=high
group=low
54
5
1
1
0
0
112
12
8
2
2
1
0
2
4
6
8
10
Time(years)

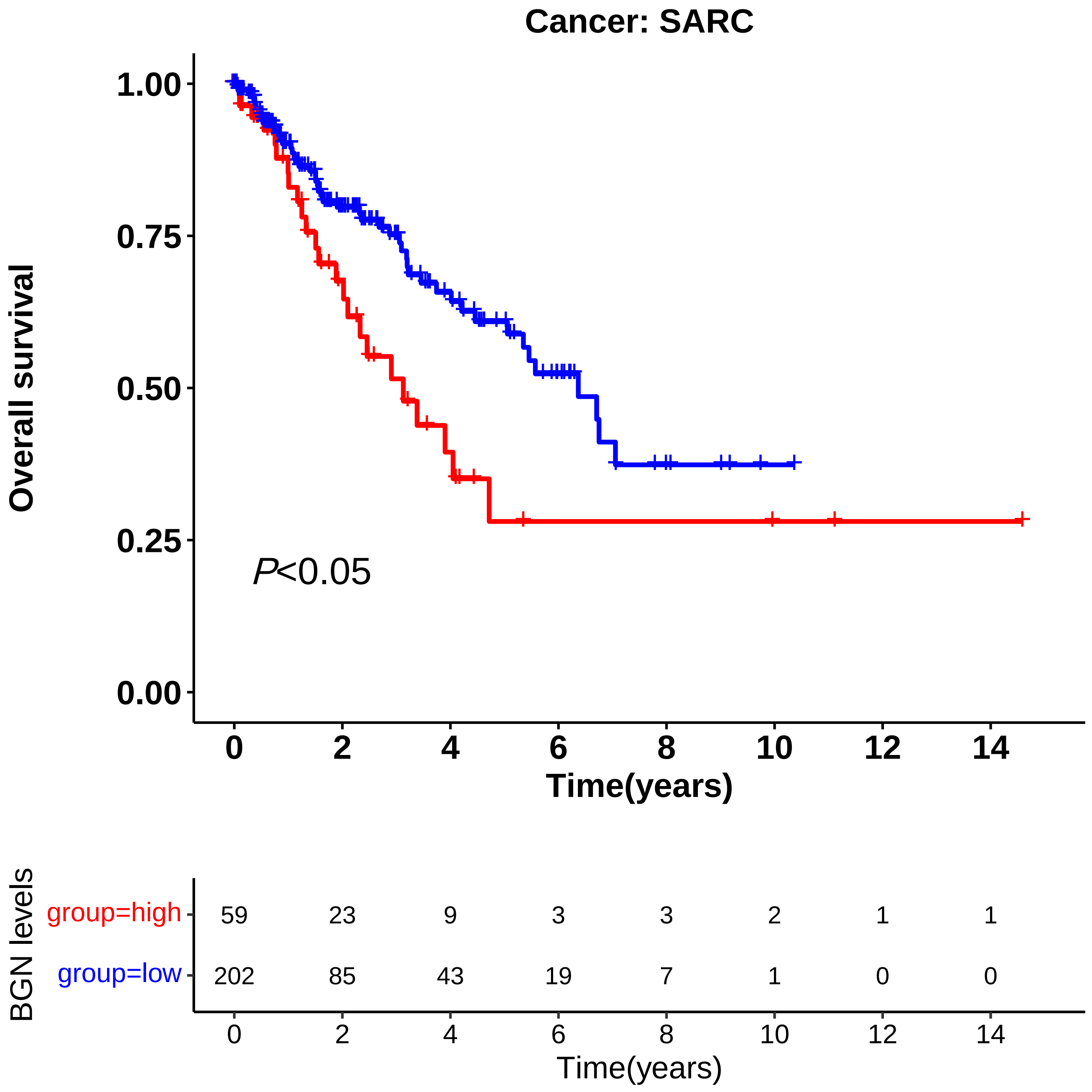

Cancer: SARC
Overall survival
1.00
0.75
0.50
0.25
0.00
P<0.05
0
2
4
6
8
10
12
14
Time(years)
BGN levels
group=high
59 23 9 3 3 2 1 1
group=low
202 85 43 19 7 1 0 0
0 2 4 6 8 10 12 14
Time(years)

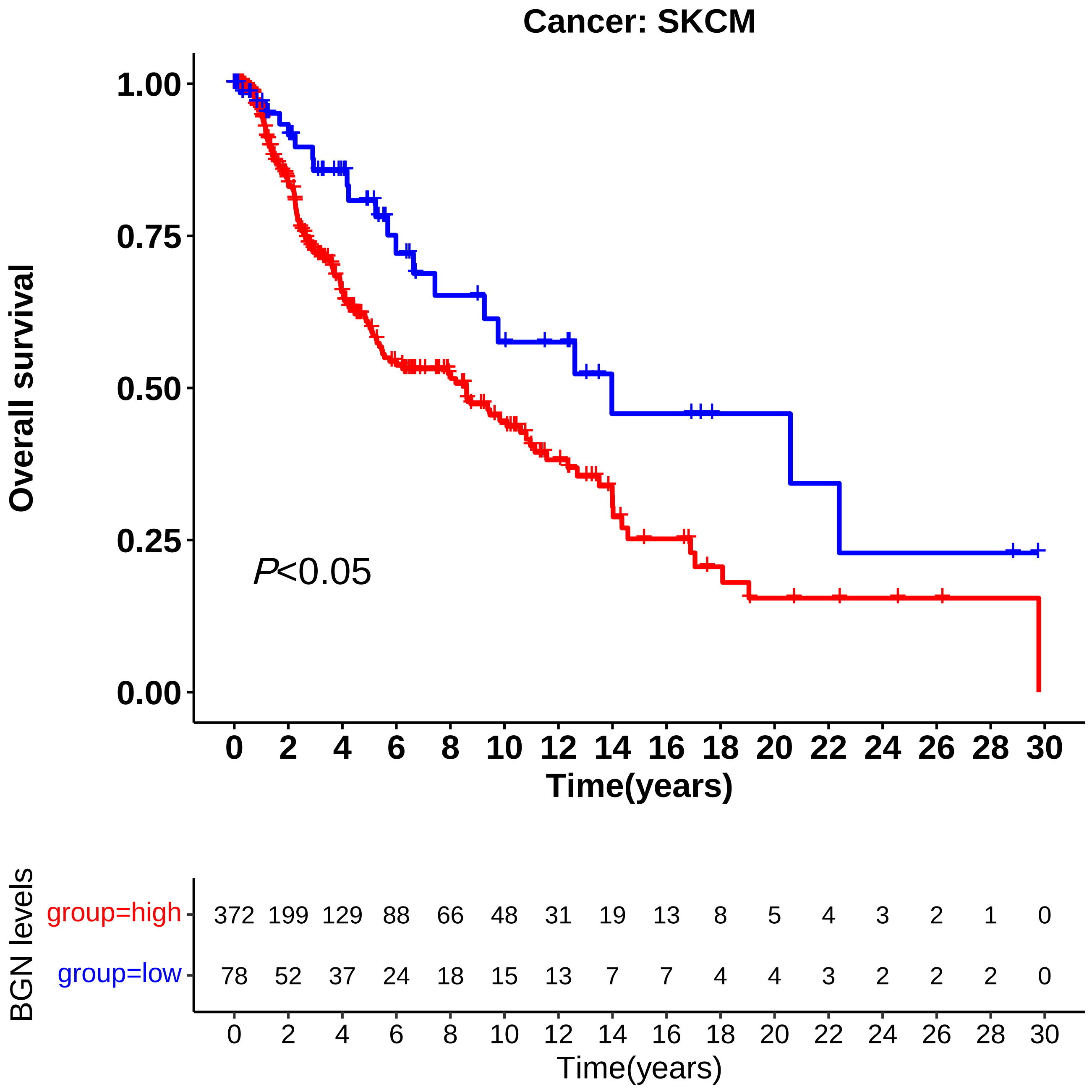

Cancer: SKCM
Overall survival
1.00
0.75
0.50
0.25
0.00
P<0.05
0 2 4 6 8 10 12 14 16 18 20 22 24 26 28 30
Time(years)
BGN levels
group=high
group=low
372 199 129 88 66 48 31 19 13 8 5 4 3 2 1 0
78 52 37 24 18 15 13 7 7 4 4 3 2 2 2 0
0 2 4 6 8 10 12 14 16 18 20 22 24 26 28 30
Time(years)

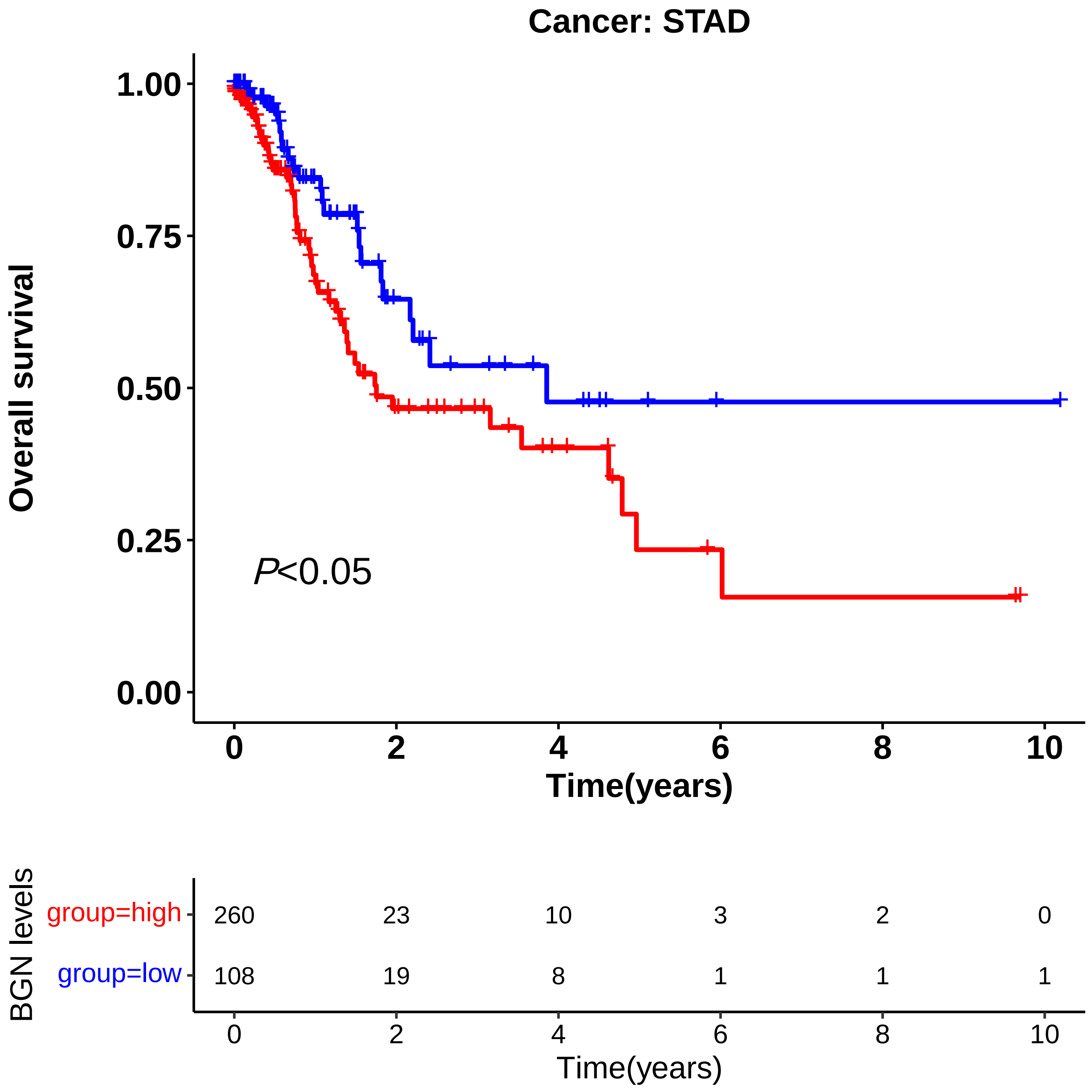

Cancer: STAD
Overall survival
1.00
0.75
0.50
0.25
0.00
P<0.05
0
2
4
6
8
10
Time(years)
BGN levels
group=high
260
23
10
3
2
0
group=low
108
19
8
1
1
1
0
2
4
6
8
10
Time(years)

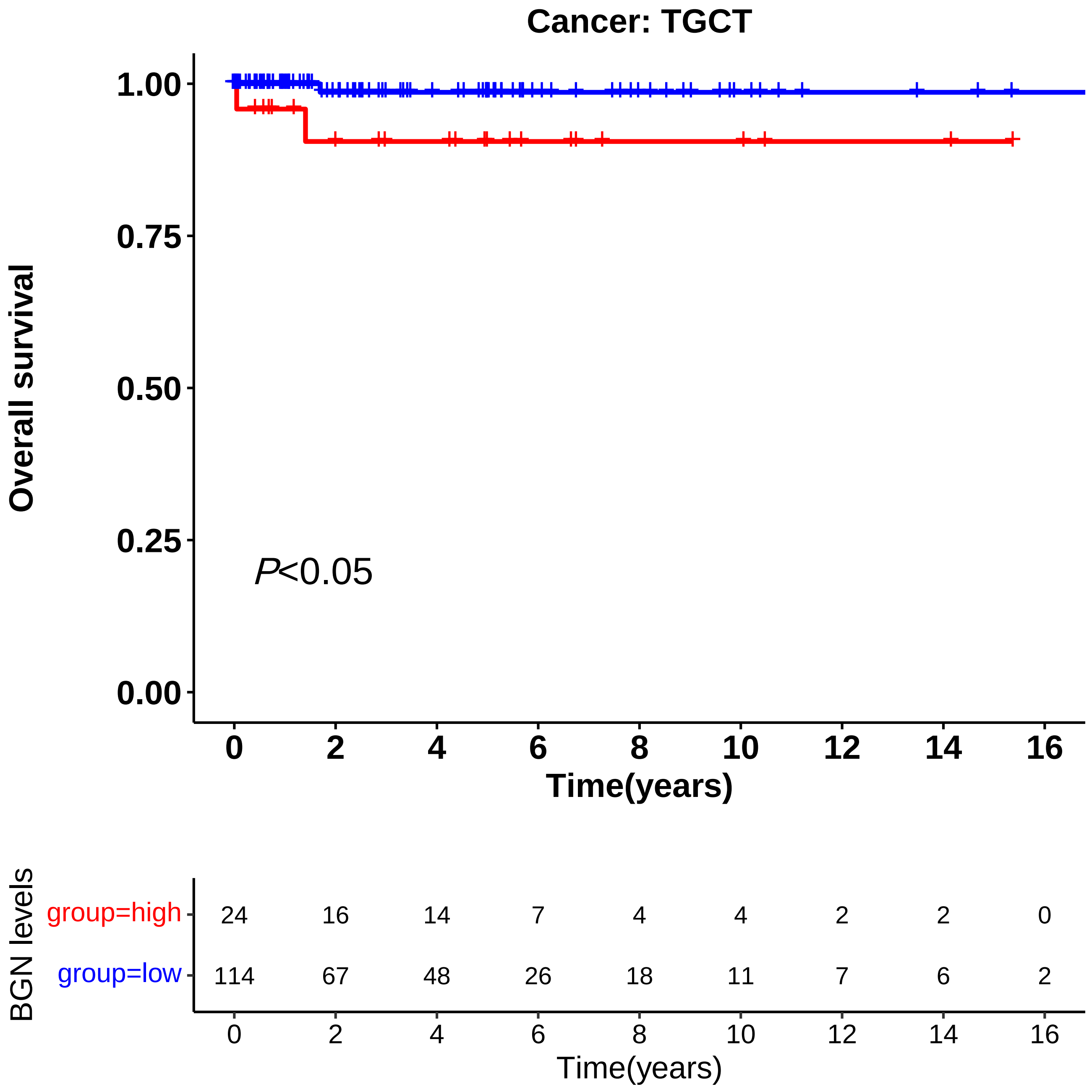

Cancer: TGCT
Overall survival
1.00
0.75
0.50
0.25
0.00
P<0.05
0
2
4
6
8
10
12
14
16
Time(years)
BGN levels
group=high
group=low
24 16 14 7 4 4 2 2 0
114 67 48 26 18 11 7 6 2
0
2
4
6
8
10
12
14
16
Time(years)

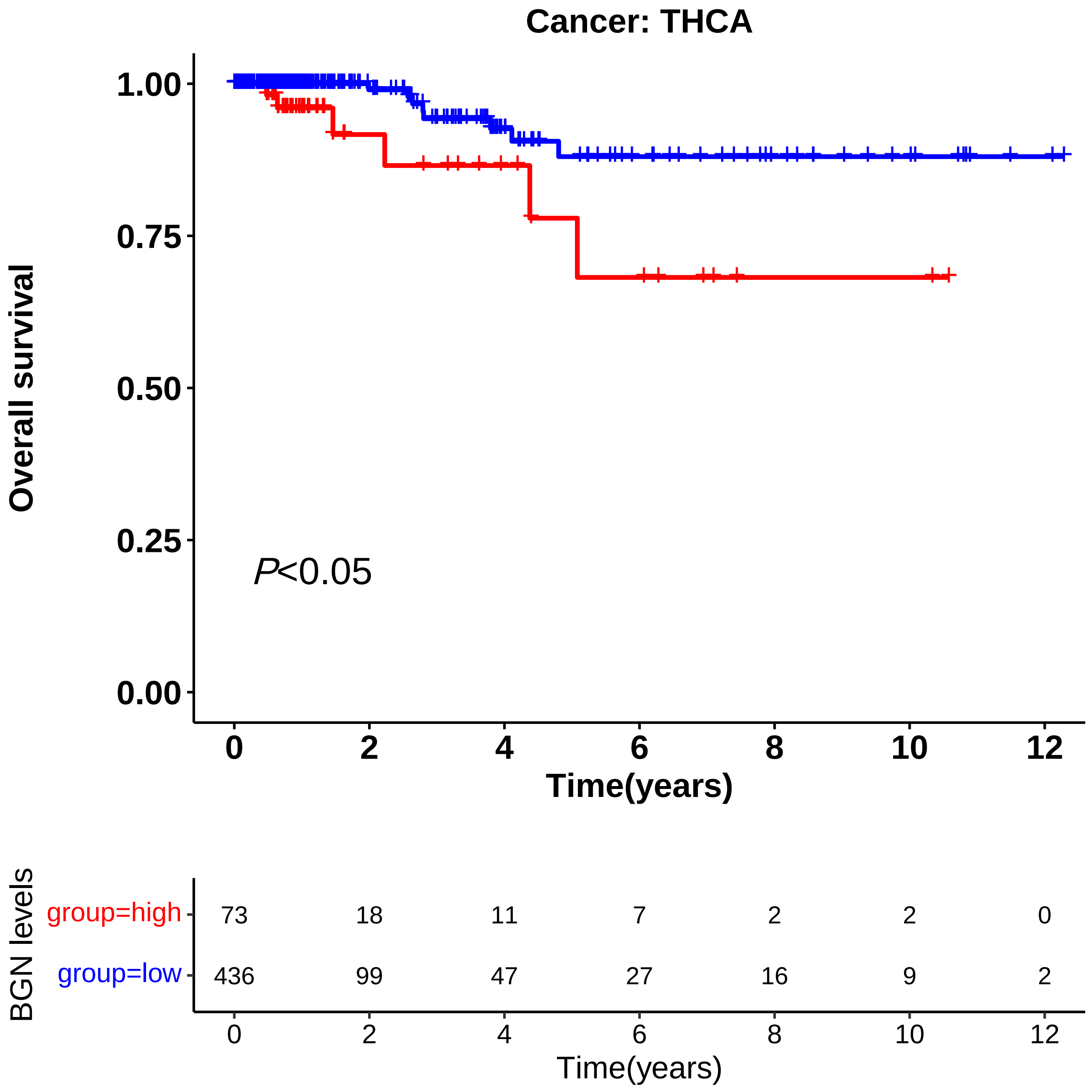

Cancer: THCA
Overall survival
1.00
0.75
0.50
0.25
0.00
P<0.05
0
2
4
6
8
10
12
Time(years)
BGN levels
group=high
group=low
73 18 11 7 2 2 0
436 99 47 27 16 9 2
0
2
4
6
8
10
12
Time(years)

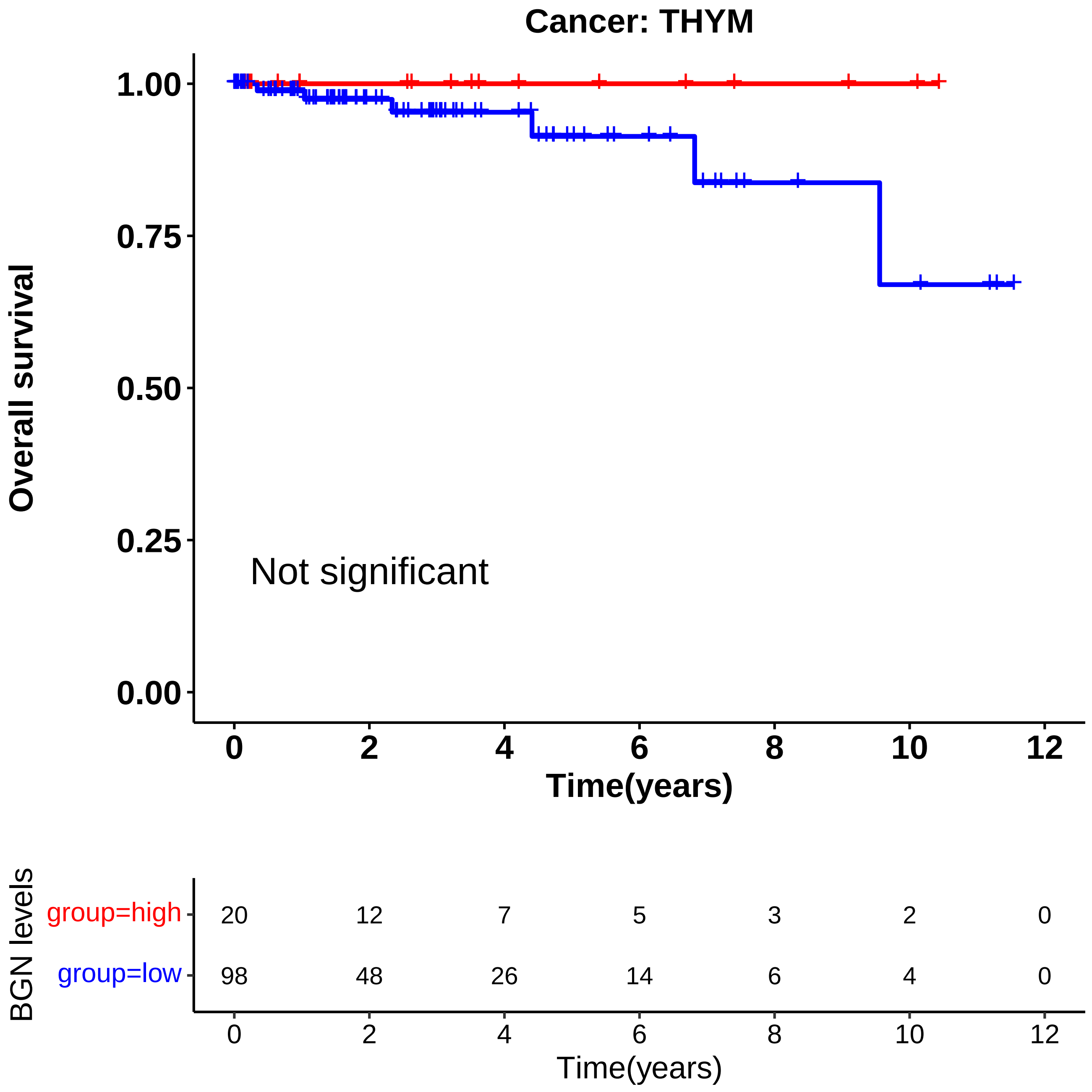
Cancer: THYM
Overall survival
1.00
0.75
0.50
0.25
0.00
Not significant
0
2
4
6
8
10
12
Time(years)
BGN levels
group=high
group=low
20
12
7
5
3
2
0
98
48
26
14
6
4
0
0
2
4
6
8
10
12
Time(years)

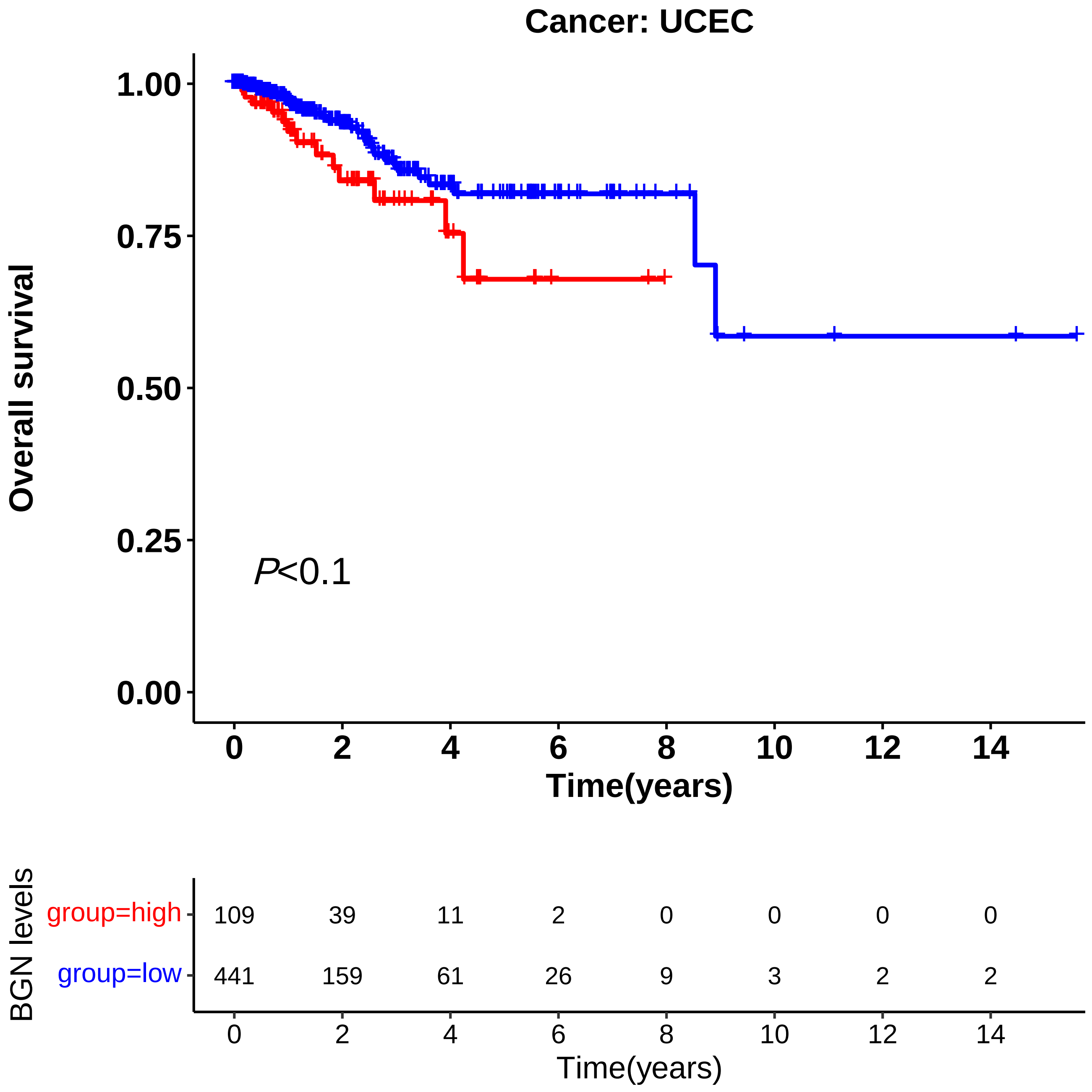

Cancer: UCEC
Overall survival
1.00
0.75
0.50
0.25
0.00
P<0.1
0
2
4
6
8
10
12
14
Time(years)
BGN levels
group=high
group=low
109 39 11 2 0 0 0 0
441 159 61 26 9 3 2 2
Time(years)

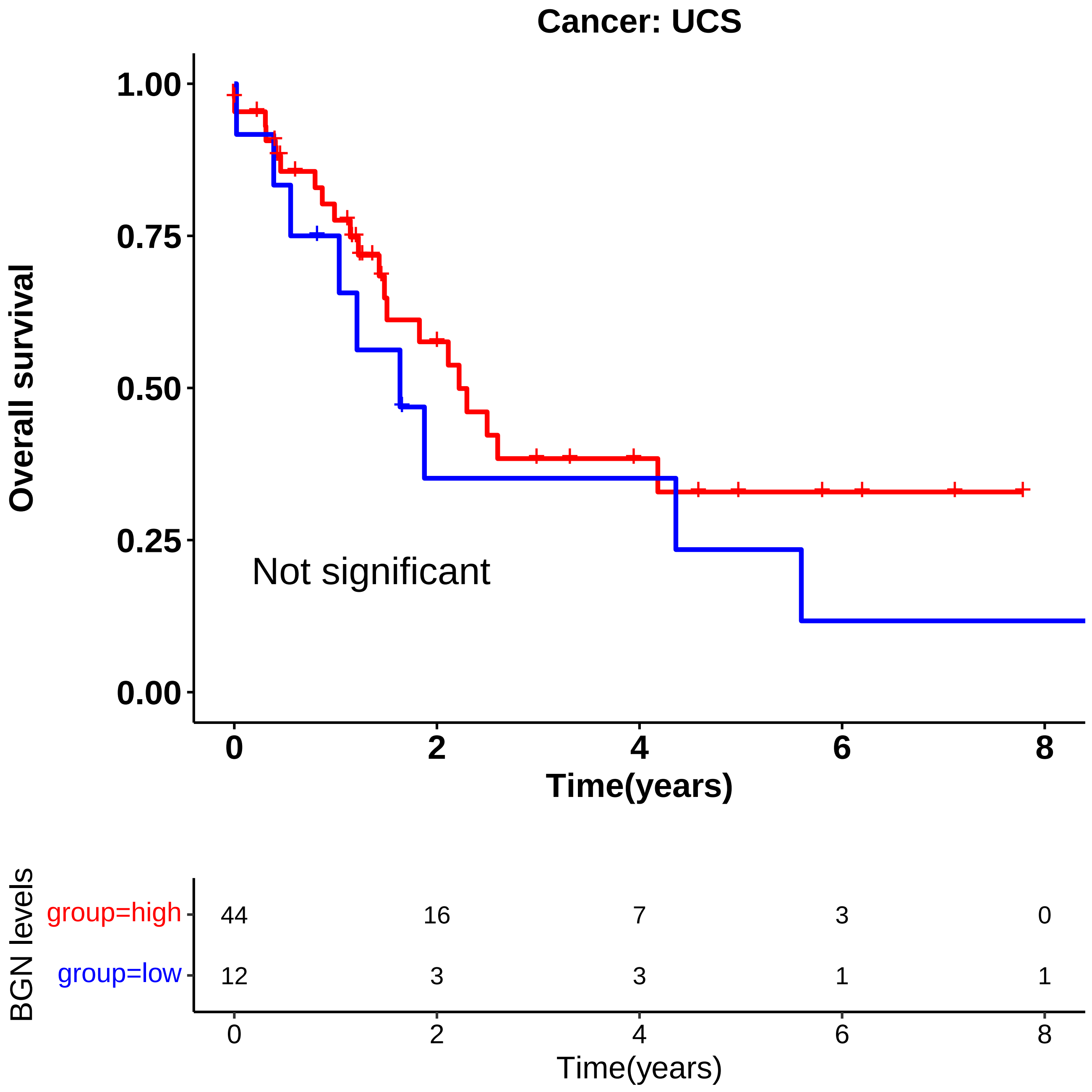

Cancer: UCS
Overall survival
1.00
0.75
0.50
0.25
0.00
Not significant
0
2
4
6
8
Time(years)
BGN levels
group=high
group=low
44
16
7
3
0
12
3
3
1
1
0
2
4
6
8
Time(years)

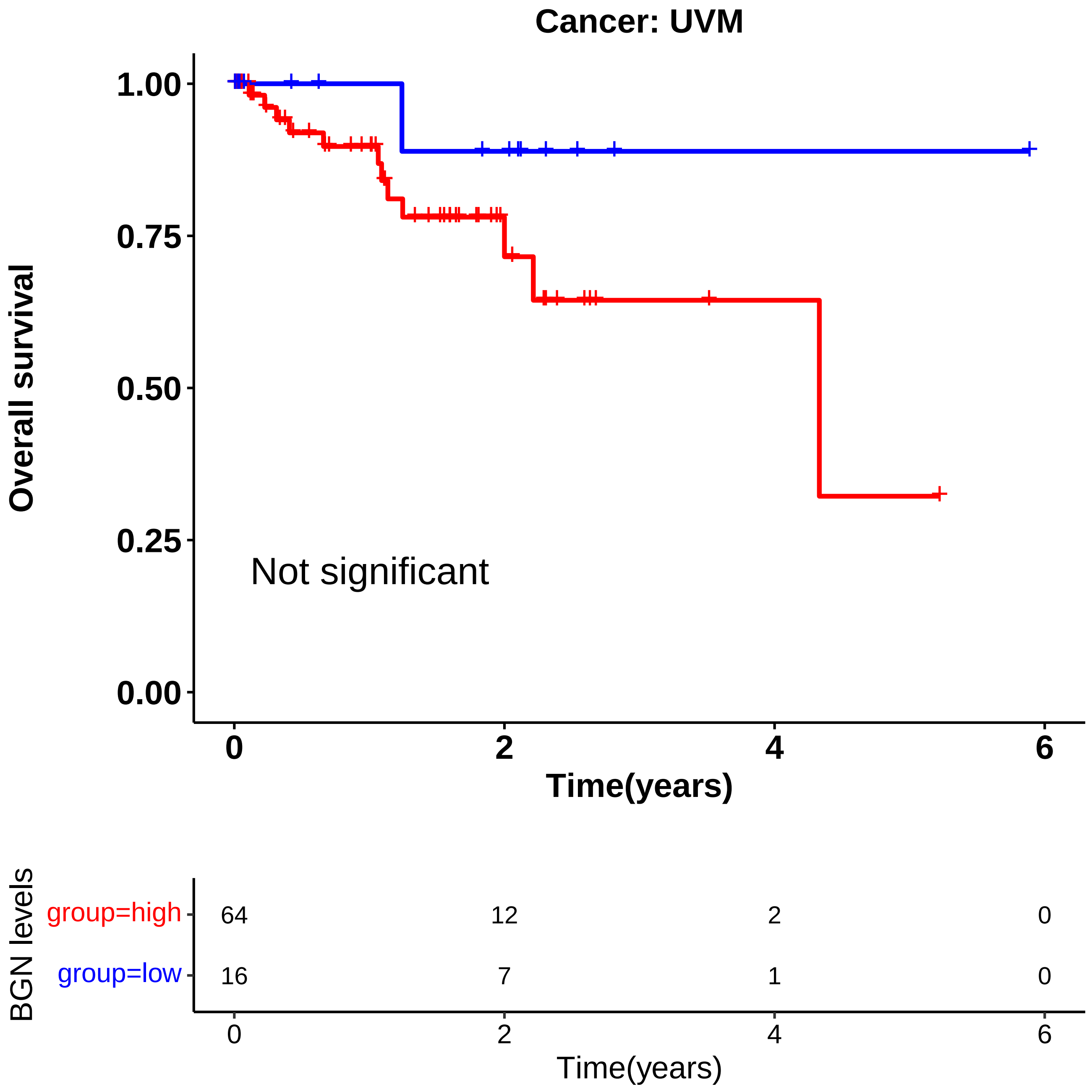
Cancer: UVM
Overall survival
1.00
0.75
0.50
0.25
0.00
Not significant
0
2
4
6
Time(years)
BGN levels
group=high
group=low
64
12
2
0
16
7
1
0
0
2
4
6
Time(years)
