# Supplementary material for: Dissecting the role of cancer‐associated fibroblast‐derived biglycan as a potential therapeutic target in immunotherapy resistance: A tumor bulk and single‐cell transcriptomic study
Source: Clin Transl Med. 2023 Feb 11;13(2):e1189. doi: 10.1002/ctm2.1189 (PMC9920016; doi:10.1002/ctm2.1189)
Supplement: Supplementary file 13 — Supporting Information [file CTM2-13-e1189-s011.pdf]

Cancer-associated fibroblasts infiltration of ACC

	Low (n=31)	Medium(n=46)	High (n=2)	Total (n=79)	*P* Value
**T stage**					
T1-2	22 (71.0%)	29 (65.9%)	0 (0.0%)	51 (66.2%)	>0.1
T3-4	9 (29.0%)	15 (34.1%)	2 (100.0%)	26 (33.8%)	
**LN metastasis**					
N+	5 (16.1%)	4 (9.1%)	0 (0.0%)	9 (11.7%)	>0.1
N0	26 (83.9%)	40 (90.9%)	2 (100.0%)	68 (88.3%)	
**Pathological stage**					
Stage I-II	18 (58.1%)	28 (63.6%)	0 (0.0%)	46 (59.7%)	>0.1
Stage III-IV	13 (41.9%)	16 (36.4%)	2 (100.0%)	31 (40.3%)	
**TP53**					
Wildtype	25 (80.6%)	37 (80.4%)	2 (100.0%)	64 (81.0%)	>0.1
Mutant	6 (19.4%)	9 (19.6%)	0 (0.0%)	15 (19.0%)	
**BRAF**					
Wildtype	30 (96.8%)	46 (100.0%)	2 (100.0%)	78 (98.7%)	>0.1
Mutant	1 (3.2%)	0 (0.0%)	0 (0.0%)	1 (1.3%)	
**EGFR**					
Wildtype	30 (96.8%)	46 (100.0%)	2 (100.0%)	78 (98.7%)	>0.1
Mutant	1 (3.2%)	0 (0.0%)	0 (0.0%)	1 (1.3%)	
**Gender**					
Female	20 (64.5%)	27 (58.7%)	1 (50.0%)	48 (60.8%)	>0.1
Male	11 (35.5%)	19 (41.3%)	1 (50.0%)	31 (39.2%)	

* Statistically significant

Cancer-associated fibroblasts infiltration of BLCA

	Low (n=89)	Medium(n=174)	High (n=151)	Total (n=414)	*P* Value
**T stage**					
T1-2	64 (71.9%)	88 (50.6%)	52 (34.4%)	204 (49.3%)	<0.01*
T3-4	25 (28.1%)	86 (49.4%)	99 (65.6%)	210 (50.7%)	
**LN metastasis**					
N+	34 (39.5%)	61 (35.7%)	74 (49.0%)	169 (41.4%)	<0.05*
N0	52 (60.5%)	110 (64.3%)	77 (51.0%)	239 (58.6%)	
**Pathological stage**					
Stage I-II	53 (60.9%)	59 (33.9%)	22 (14.6%)	134 (32.5%)	<0.01*
Stage III-IV	34 (39.1%)	115 (66.1%)	129 (85.4%)	278 (67.5%)	
**TP53**					
Wildtype	52 (58.4%)	88 (50.6%)	79 (52.3%)	219 (52.9%)	>0.1
Mutant	37 (41.6%)	86 (49.4%)	72 (47.7%)	195 (47.1%)	
**BRAF**					
Wildtype	87 (97.8%)	169 (97.1%)	150 (99.3%)	406 (98.1%)	>0.1
Mutant	2 (2.2%)	5 (2.9%)	1 (0.7%)	8 (1.9%)	
**EGFR**					
Wildtype	87 (97.8%)	170 (97.7%)	151 (100.0%)	408 (98.6%)	>0.1
Mutant	2 (2.2%)	4 (2.3%)	0 (0.0%)	6 (1.4%)	
**KRAS**					
Wildtype	84 (94.4%)	169 (97.1%)	145 (96.0%)	398 (96.1%)	>0.1
Mutant	5 (5.6%)	5 (2.9%)	6 (4.0%)	16 (3.9%)	
**Age**					
<=50	8 (10.1%)	12 (7.2%)	4 (2.8%)	24 (6.2%)	<0.1
>50	71 (89.9%)	155 (92.8%)	139 (97.2%)	365 (93.8%)	
**Gender**					
Female	17 (19.1%)	43 (24.7%)	49 (32.5%)	109 (26.3%)	<0.1
Male	72 (80.9%)	131 (75.3%)	102 (67.5%)	305 (73.7%)	

* Statistically significant

Cancer-associated fibroblasts infiltration of BRCA

	Low (n=26)	Medium(n=231)	High (n=852)	Total (n=1109)	*P* Value
**T stage**					
T1-2	20 (76.9%)	193 (83.5%)	713 (83.8%)	926 (83.6%)	>0.1
T3-4	6 (23.1%)	38 (16.5%)	138 (16.2%)	182 (16.4%)	
**LN metastasis**					
N+	12 (46.2%)	103 (44.6%)	472 (55.5%)	587 (53.0%)	<0.05*
N0	14 (53.8%)	128 (55.4%)	379 (44.5%)	521 (47.0%)	
**Pathological stage**					
Stage I-II	18 (75.0%)	189 (82.5%)	607 (73.0%)	814 (75.0%)	<0.05*
Stage III-IV	6 (25.0%)	40 (17.5%)	225 (27.0%)	271 (25.0%)	
**TP53**					
Wildtype	9 (42.9%)	122 (57.8%)	524 (68.5%)	655 (65.7%)	<0.01*
Mutant	12 (57.1%)	89 (42.2%)	241 (31.5%)	342 (34.3%)	
**BRAF**					
Wildtype	21 (100.0%)	211 (100.0%)	759 (99.2%)	991 (99.4%)	>0.1
Mutant	0 (0.0%)	0 (0.0%)	6 (0.8%)	6 (0.6%)	
**EGFR**					
Wildtype	21 (100.0%)	210 (99.5%)	756 (98.8%)	987 (99.0%)	>0.1
Mutant	0 (0.0%)	1 (0.5%)	9 (1.2%)	10 (1.0%)	
**KRAS**					
Wildtype	21 (100.0%)	211 (100.0%)	759 (99.2%)	991 (99.4%)	>0.1
Mutant	0 (0.0%)	0 (0.0%)	6 (0.8%)	6 (0.6%)	
**Age**					
<=50	5 (19.2%)	63 (27.3%)	271 (31.8%)	339 (30.6%)	>0.1
>50	21 (80.8%)	168 (72.7%)	580 (68.2%)	769 (69.4%)	
**Gender**					
Female	26 (100.0%)	228 (98.7%)	842 (98.9%)	1096 (98.9%)	>0.1
Male	0 (0.0%)	3 (1.3%)	9 (1.1%)	12 (1.1%)	

* Statistically significant

Cancer-associated fibroblasts infiltration of CESC

	Low (n=96)	Medium(n=160)	High (n=50)	Total (n=306)	*P* Value
**T stage**					
T1-2	69 (85.2%)	113 (81.3%)	30 (73.2%)	212 (81.2%)	>0.1
T3-4	12 (14.8%)	26 (18.7%)	11 (26.8%)	49 (18.8%)	
**LN metastasis**					
N+	38 (46.9%)	70 (50.4%)	19 (46.3%)	127 (48.7%)	>0.1
N0	43 (53.1%)	69 (49.6%)	22 (53.7%)	134 (51.3%)	
**TP53**					
Wildtype	80 (88.9%)	141 (91.6%)	44 (100.0%)	265 (92.0%)	<0.1
Mutant	10 (11.1%)	13 (8.4%)	0 (0.0%)	23 (8.0%)	
**BRAF**					
Wildtype	89 (98.9%)	152 (98.7%)	44 (100.0%)	285 (99.0%)	>0.1
Mutant	1 (1.1%)	2 (1.3%)	0 (0.0%)	3 (1.0%)	
**EGFR**					
Wildtype	87 (96.7%)	151 (98.1%)	44 (100.0%)	282 (97.9%)	>0.1
Mutant	3 (3.3%)	3 (1.9%)	0 (0.0%)	6 (2.1%)	
**KRAS**					
Wildtype	82 (91.1%)	149 (96.8%)	41 (93.2%)	272 (94.4%)	>0.1
Mutant	8 (8.9%)	5 (3.2%)	3 (6.8%)	16 (5.6%)	
**Age**					
<=50	53 (55.2%)	102 (63.7%)	33 (66.0%)	188 (61.4%)	>0.1
>50	43 (44.8%)	58 (36.2%)	17 (34.0%)	118 (38.6%)	

* Statistically significant

Cancer-associated fibroblasts infiltration of CHOL

	Low (n=3)	Medium(n=20)	High (n=13)	Total (n=36)	*P* Value
**T stage**					
T1-2	3 (100.0%)	17 (85.0%)	11 (84.6%)	31 (86.1%)	>0.1
T3-4	0 (0.0%)	3 (15.0%)	2 (15.4%)	5 (13.9%)	
**LN metastasis**					
N0	3 (100.0%)	15 (75.0%)	8 (61.5%)	26 (72.2%)	>0.1
N+	0 (0.0%)	5 (25.0%)	5 (38.5%)	10 (27.8%)	
**Pathological stage**					
Stage I-II	3 (100.0%)	15 (75.0%)	10 (76.9%)	28 (77.8%)	>0.1
Stage III-IV	0 (0.0%)	5 (25.0%)	3 (23.1%)	8 (22.2%)	
**TP53**					
Wildtype	1 (33.3%)	19 (95.0%)	12 (92.3%)	32 (88.9%)	<0.05*
Mutant	2 (66.7%)	1 (5.0%)	1 (7.7%)	4 (11.1%)	
**BRAF**					
Mutant	0 (0.0%)	0 (0.0%)	1 (7.7%)	1 (2.8%)	>0.1
Wildtype	3 (100.0%)	20 (100.0%)	12 (92.3%)	35 (97.2%)	
**KRAS**					
Mutant	0 (0.0%)	0 (0.0%)	2 (15.4%)	2 (5.6%)	>0.1
Wildtype	3 (100.0%)	20 (100.0%)	11 (84.6%)	34 (94.4%)	
**Gender**					
Male	3 (100.0%)	7 (35.0%)	6 (46.2%)	16 (44.4%)	>0.1
Female	0 (0.0%)	13 (65.0%)	7 (53.8%)	20 (55.6%)	

* Statistically significant

Cancer-associated fibroblasts infiltration of COAD

	Low (n=52)	Medium(n=253)	High (n=175)	Total (n=480)	*P* Value
**T stage**					
T1-2	14 (26.9%)	54 (21.5%)	26 (14.9%)	94 (19.7%)	<0.1
T3-4	38 (73.1%)	197 (78.5%)	148 (85.1%)	383 (80.3%)	
**LN metastasis**					
N+	13 (25.0%)	96 (38.2%)	85 (48.9%)	194 (40.7%)	<0.01*
N0	39 (75.0%)	155 (61.8%)	89 (51.1%)	283 (59.3%)	
**Pathological stage**					
Stage I-II	36 (70.6%)	143 (58.4%)	88 (51.8%)	267 (57.3%)	<0.1
Stage III-IV	15 (29.4%)	102 (41.6%)	82 (48.2%)	199 (42.7%)	
**TP53**					
Wildtype	23 (48.9%)	100 (48.1%)	66 (40.7%)	189 (45.3%)	>0.1
Mutant	24 (51.1%)	108 (51.9%)	96 (59.3%)	228 (54.7%)	
**BRAF**					
Wildtype	41 (87.2%)	177 (85.1%)	136 (84.0%)	354 (84.9%)	>0.1
Mutant	6 (12.8%)	31 (14.9%)	26 (16.0%)	63 (15.1%)	
**EGFR**					
Wildtype	46 (97.9%)	201 (96.6%)	158 (97.5%)	405 (97.1%)	>0.1
Mutant	1 (2.1%)	7 (3.4%)	4 (2.5%)	12 (2.9%)	
**KRAS**					
Wildtype	22 (46.8%)	125 (60.1%)	102 (63.0%)	249 (59.7%)	>0.1
Mutant	25 (53.2%)	83 (39.9%)	60 (37.0%)	168 (40.3%)	
**Age**					
<=50	4 (7.7%)	30 (12.0%)	28 (16.1%)	62 (13.0%)	>0.1
>50	48 (92.3%)	221 (88.0%)	146 (83.9%)	415 (87.0%)	
**Gender**					
Female	23 (44.2%)	121 (48.2%)	82 (47.1%)	226 (47.4%)	>0.1
Male	29 (55.8%)	130 (51.8%)	92 (52.9%)	251 (52.6%)	

* Statistically significant

Cancer-associated fibroblasts infiltration of DLBC

	Low (n=6)	Medium(n=37)	High (n=5)	Total (n=48)	*P* Value
**TP53**					
Wildtype	4 (80.0%)	25 (89.3%)	3 (75.0%)	32 (86.5%)	>0.1
Mutant	1 (20.0%)	3 (10.7%)	1 (25.0%)	5 (13.5%)	
**BRAF**					
Wildtype	5 (100.0%)	27 (96.4%)	4 (100.0%)	36 (97.3%)	>0.1
Mutant	0 (0.0%)	1 (3.6%)	0 (0.0%)	1 (2.7%)	
**KRAS**					
Wildtype	5 (100.0%)	26 (92.9%)	4 (100.0%)	35 (94.6%)	>0.1
Mutant	0 (0.0%)	2 (7.1%)	0 (0.0%)	2 (5.4%)	
**Age**					
<=50	1 (16.7%)	11 (29.7%)	2 (40.0%)	14 (29.2%)	>0.1
>50	5 (83.3%)	26 (70.3%)	3 (60.0%)	34 (70.8%)	
**Gender**					
Female	5 (83.3%)	18 (48.6%)	3 (60.0%)	26 (54.2%)	>0.1
Male	1 (16.7%)	19 (51.4%)	2 (40.0%)	22 (45.8%)	

* Statistically significant

Cancer-associated fibroblasts infiltration of ESCA

	Low (n=13)	Medium(n=77)	High (n=72)	Total (n=162)	*P* Value
**T stage**					
T1-2	7 (53.8%)	35 (45.5%)	24 (33.3%)	66 (40.7%)	>0.1
T3-4	6 (46.2%)	42 (54.5%)	48 (66.7%)	96 (59.3%)	
**LN metastasis**					
N+	9 (69.2%)	47 (61.0%)	41 (56.9%)	97 (59.9%)	>0.1
N0	4 (30.8%)	30 (39.0%)	31 (43.1%)	65 (40.1%)	
**Pathological stage**					
Stage I-II	8 (72.7%)	42 (64.6%)	35 (53.0%)	85 (59.9%)	>0.1
Stage III-IV	3 (27.3%)	23 (35.4%)	31 (47.0%)	57 (40.1%)	
**TP53**					
Wildtype	4 (30.8%)	16 (20.8%)	7 (9.9%)	27 (16.8%)	<0.1
Mutant	9 (69.2%)	61 (79.2%)	64 (90.1%)	134 (83.2%)	
**BRAF**					
Wildtype	13 (100.0%)	77 (100.0%)	70 (98.6%)	160 (99.4%)	>0.1
Mutant	0 (0.0%)	0 (0.0%)	1 (1.4%)	1 (0.6%)	
**EGFR**					
Wildtype	13 (100.0%)	75 (97.4%)	68 (95.8%)	156 (96.9%)	>0.1
Mutant	0 (0.0%)	2 (2.6%)	3 (4.2%)	5 (3.1%)	
**KRAS**					
Wildtype	13 (100.0%)	76 (98.7%)	70 (98.6%)	159 (98.8%)	>0.1
Mutant	0 (0.0%)	1 (1.3%)	1 (1.4%)	2 (1.2%)	
**Gender**					
Female	2 (15.4%)	13 (16.9%)	8 (11.1%)	23 (14.2%)	>0.1
Male	11 (84.6%)	64 (83.1%)	64 (88.9%)	139 (85.8%)	
**Cancer type**					
Adenocarcinomas	6 (46.2%)	39 (50.6%)	35 (48.6%)	80 (49.4%)	>0.1
Squamous	7 (53.8%)	38 (49.4%)	37 (51.4%)	82 (50.6%)	

* Statistically significant

Cancer-associated fibroblasts infiltration of GBM

	Medium(n=155)	High (n=14)	Total (n=169)	*P* Value
**TP53**				
Wildtype	101 (68.7%)	5 (38.5%)	106 (66.2%)	<0.1
Mutant	46 (31.3%)	8 (61.5%)	54 (33.8%)	
**BRAF**				
Wildtype	146 (99.3%)	12 (92.3%)	158 (98.8%)	>0.1
Mutant	1 (0.7%)	1 (7.7%)	2 (1.2%)	
**EGFR**				
Wildtype	110 (74.8%)	13 (100.0%)	123 (76.9%)	<0.1
Mutant	37 (25.2%)	0 (0.0%)	37 (23.1%)	
**KRAS**				
Wildtype	146 (99.3%)	13 (100.0%)	159 (99.4%)	>0.1
Mutant	1 (0.7%)	0 (0.0%)	1 (0.6%)	
**Age**				
<=50	39 (25.5%)	3 (21.4%)	42 (25.1%)	>0.1
>50	114 (74.5%)	11 (78.6%)	125 (74.9%)	
**Gender**				
Female	55 (35.9%)	4 (28.6%)	59 (35.3%)	>0.1
Male	98 (64.1%)	10 (71.4%)	108 (64.7%)	

* Statistically significant

Cancer-associated fibroblasts infiltration of HNSC

	Low (n=33)	Medium(n=307)	High (n=162)	Total (n=502)	*P* Value
**T stage**					
T1-2	10 (30.3%)	111 (36.2%)	57 (35.2%)	178 (35.5%)	>0.1
T3-4	23 (69.7%)	196 (63.8%)	105 (64.8%)	324 (64.5%)	
**LN metastasis**					
N+	18 (54.5%)	158 (51.5%)	85 (52.5%)	261 (52.0%)	>0.1
N0	15 (45.5%)	149 (48.5%)	77 (47.5%)	241 (48.0%)	
**Pathological stage**					
Stage I-II	5 (16.7%)	60 (23.2%)	30 (20.7%)	95 (21.9%)	>0.1
Stage III-IV	25 (83.3%)	199 (76.8%)	115 (79.3%)	339 (78.1%)	
**TP53**					
Wildtype	20 (60.6%)	106 (35.0%)	42 (26.6%)	168 (34.0%)	<0.01 *
Mutant	13 (39.4%)	197 (65.0%)	116 (73.4%)	326 (66.0%)	
**BRAF**					
Wildtype	32 (97.0%)	299 (98.7%)	157 (99.4%)	488 (98.8%)	>0.1
Mutant	1 (3.0%)	4 (1.3%)	1 (0.6%)	6 (1.2%)	
**EGFR**					
Wildtype	33 (100.0%)	297 (98.0%)	155 (98.1%)	485 (98.2%)	>0.1
Mutant	0 (0.0%)	6 (2.0%)	3 (1.9%)	9 (1.8%)	
**Age**					
<=50	4 (12.1%)	52 (17.0%)	32 (19.8%)	88 (17.6%)	>0.1
>50	29 (87.9%)	254 (83.0%)	130 (80.2%)	413 (82.4%)	
**Gender**					
Female	8 (24.2%)	85 (27.7%)	41 (25.3%)	134 (26.7%)	>0.1
Male	25 (75.8%)	222 (72.3%)	121 (74.7%)	368 (73.3%)	

* Statistically significant

Cancer-associated fibroblasts infiltration of KICH

	Low (n=15)	Medium(n=49)	High (n=1)	Total (n=65)	*P* Value
**T stage**					
T1-2	11 (73.3%)	34 (69.4%)	0 (0.0%)	45 (69.2%)	>0.1
T3-4	4 (26.7%)	15 (30.6%)	1 (100.0%)	20 (30.8%)	
**LN metastasis**					
N+	9 (60.0%)	16 (32.7%)	1 (100.0%)	26 (40.0%)	<0.05*
N0	6 (40.0%)	33 (67.3%)	0 (0.0%)	39 (60.0%)	
**Pathological stage**					
Stage I-II	11 (73.3%)	34 (69.4%)	0 (0.0%)	45 (69.2%)	>0.1
Stage III-IV	4 (26.7%)	15 (30.6%)	1 (100.0%)	20 (30.8%)	
**TP53**					
Wildtype	11 (73.3%)	35 (71.4%)	0 (0.0%)	46 (70.8%)	>0.1
Mutant	4 (26.7%)	14 (28.6%)	1 (100.0%)	19 (29.2%)	
**Age**					
<=50	7 (46.7%)	26 (53.1%)	0 (0.0%)	33 (50.8%)	>0.1
>50	8 (53.3%)	23 (46.9%)	1 (100.0%)	32 (49.2%)	
**Gender**					
Female	3 (20.0%)	23 (46.9%)	0 (0.0%)	26 (40.0%)	<0.1
Male	12 (80.0%)	26 (53.1%)	1 (100.0%)	39 (60.0%)	

* Statistically significant

Cancer-associated fibroblasts infiltration of KIRC

	Low (n=18)	Medium(n=422)	High (n=99)	Total (n=539)	*P* Value
**T stage**					
T1-2	17 (94.4%)	279 (66.6%)	52 (52.5%)	348 (64.9%)	<0.01*
T3-4	1 (5.6%)	140 (33.4%)	47 (47.5%)	188 (35.1%)	
**LN metastasis**					
N+	11 (61.1%)	236 (56.3%)	51 (51.5%)	298 (55.6%)	>0.1
N0	7 (38.9%)	183 (43.7%)	48 (48.5%)	238 (44.4%)	
**Pathological stage**					
Stage I-II	15 (83.3%)	264 (63.0%)	51 (53.1%)	330 (61.9%)	<0.05*
Stage III-IV	3 (16.7%)	155 (37.0%)	45 (46.9%)	203 (38.1%)	
**TP53**					
Wildtype	12 (85.7%)	259 (98.9%)	59 (96.7%)	330 (97.9%)	<0.05*
Mutant	2 (14.3%)	3 (1.1%)	2 (3.3%)	7 (2.1%)	
**BRAF**					
Wildtype	14 (100.0%)	261 (99.6%)	61 (100.0%)	336 (99.7%)	>0.1
Mutant	0 (0.0%)	1 (0.4%)	0 (0.0%)	1 (0.3%)	
**EGFR**					
Wildtype	14 (100.0%)	260 (99.2%)	61 (100.0%)	335 (99.4%)	>0.1
Mutant	0 (0.0%)	2 (0.8%)	0 (0.0%)	2 (0.6%)	
**KRAS**					
Wildtype	14 (100.0%)	262 (100.0%)	60 (98.4%)	336 (99.7%)	>0.1
Mutant	0 (0.0%)	0 (0.0%)	1 (1.6%)	1 (0.3%)	
**Age**					
<=50	3 (16.7%)	87 (20.8%)	24 (24.2%)	114 (21.3%)	>0.1
>50	15 (83.3%)	332 (79.2%)	75 (75.8%)	422 (78.7%)	
**Gender**					
Female	4 (22.2%)	154 (36.8%)	27 (27.3%)	185 (34.5%)	>0.1
Male	14 (77.8%)	265 (63.2%)	72 (72.7%)	351 (65.5%)	

* Statistically significant

Cancer-associated fibroblasts infiltration of KIRP

	Low (n=195)	Medium(n=86)	High (n=8)	Total (n=289)	*P* Value
**T stage**					
T1-2	170 (87.2%)	64 (74.4%)	2 (25.0%)	236 (81.7%)	<0.01*
T3-4	25 (12.8%)	22 (25.6%)	6 (75.0%)	53 (18.3%)	
**LN metastasis**					
N+	94 (48.2%)	46 (53.5%)	6 (75.0%)	146 (50.5%)	>0.1
N0	101 (51.8%)	40 (46.5%)	2 (25.0%)	143 (49.5%)	
**Pathological stage**					
Stage I-II	138 (80.7%)	56 (69.1%)	0 (0.0%)	194 (74.6%)	<0.01*
Stage III-IV	33 (19.3%)	25 (30.9%)	8 (100.0%)	66 (25.4%)	
**TP53**					
Wildtype	183 (97.3%)	82 (98.8%)	8 (100.0%)	273 (97.8%)	>0.1
Mutant	5 (2.7%)	1 (1.2%)	0 (0.0%)	6 (2.2%)	
**BRAF**					
Wildtype	186 (98.9%)	82 (98.8%)	8 (100.0%)	276 (98.9%)	>0.1
Mutant	2 (1.1%)	1 (1.2%)	0 (0.0%)	3 (1.1%)	
**KRAS**					
Wildtype	184 (97.9%)	82 (98.8%)	8 (100.0%)	274 (98.2%)	>0.1
Mutant	4 (2.1%)	1 (1.2%)	0 (0.0%)	5 (1.8%)	
**Age**					
<=50	28 (14.6%)	18 (20.9%)	1 (12.5%)	47 (16.4%)	>0.1
>50	164 (85.4%)	68 (79.1%)	7 (87.5%)	239 (83.6%)	
**Gender**					
Female	40 (20.5%)	34 (39.5%)	3 (37.5%)	77 (26.6%)	<0.01*
Male	155 (79.5%)	52 (60.5%)	5 (62.5%)	212 (73.4%)	

* Statistically significant

Cancer-associated fibroblasts infiltration of LAML

	Low (n=148)	Medium(n=3)	Total (n=151)	*P* Value
**TP53**				
Wildtype	96 (93.2%)	2 (66.7%)	98 (92.5%)	>0.1
Mutant	7 (6.8%)	1 (33.3%)	8 (7.5%)	
**KRAS**				
Wildtype	99 (96.1%)	3 (100.0%)	102 (96.2%)	>0.1
Mutant	4 (3.9%)	0 (0.0%)	4 (3.8%)	
**Age**				
<=50	57 (38.5%)	0 (0.0%)	57 (37.7%)	>0.1
>50	91 (61.5%)	3 (100.0%)	94 (62.3%)	
**Gender**				
Female	67 (45.3%)	1 (33.3%)	68 (45.0%)	>0.1
Male	81 (54.7%)	2 (66.7%)	83 (55.0%)	

* Statistically significant

Cancer-associated fibroblasts infiltration of LGG

	Low (n=1)	Medium(n=523)	High (n=5)	Total (n=529)	*P* Value
**TP53**					
Wildtype	0 (0.0%)	276 (53.9%)	2 (40.0%)	278 (53.7%)	>0.1
Mutant	1 (100.0%)	236 (46.1%)	3 (60.0%)	240 (46.3%)	
**BRAF**					
Wildtype	1 (100.0%)	509 (99.4%)	5 (100.0%)	515 (99.4%)	>0.1
Mutant	0 (0.0%)	3 (0.6%)	0 (0.0%)	3 (0.6%)	
**EGFR**					
Wildtype	1 (100.0%)	482 (94.1%)	5 (100.0%)	488 (94.2%)	>0.1
Mutant	0 (0.0%)	30 (5.9%)	0 (0.0%)	30 (5.8%)	
**KRAS**					
Wildtype	1 (100.0%)	511 (99.8%)	5 (100.0%)	517 (99.8%)	>0.1
Mutant	0 (0.0%)	1 (0.2%)	0 (0.0%)	1 (0.2%)	
**Age**					
<=50	1 (100.0%)	369 (70.7%)	3 (60.0%)	373 (70.6%)	>0.1
>50	0 (0.0%)	153 (29.3%)	2 (40.0%)	155 (29.4%)	
**Gender**					
Female	1 (100.0%)	238 (45.6%)	0 (0.0%)	239 (45.3%)	<0.05*
Male	0 (0.0%)	284 (54.4%)	5 (100.0%)	289 (54.7%)	

* Statistically significant

Cancer-associated fibroblasts infiltration of LIHC

	Low (n=194)	Medium(n=165)	High (n=15)	Total (n=374)	*P* Value
**T stage**					
T1-2	150 (77.7%)	118 (72.0%)	10 (66.7%)	278 (74.7%)	>0.1
T3-4	43 (22.3%)	46 (28.0%)	5 (33.3%)	94 (25.3%)	
**LN metastasis**					
N+	62 (32.0%)	52 (31.7%)	5 (33.3%)	119 (31.9%)	>0.1
N0	132 (68.0%)	112 (68.3%)	10 (66.7%)	254 (68.1%)	
**Pathological stage**					
Stage I-II	144 (77.8%)	107 (71.3%)	9 (60.0%)	260 (74.3%)	>0.1
Stage III-IV	41 (22.2%)	43 (28.7%)	6 (40.0%)	90 (25.7%)	
**TP53**					
Wildtype	131 (68.9%)	114 (72.2%)	10 (71.4%)	255 (70.4%)	>0.1
Mutant	59 (31.1%)	44 (27.8%)	4 (28.6%)	107 (29.6%)	
**EGFR**					
Wildtype	186 (97.9%)	157 (99.4%)	14 (100.0%)	357 (98.6%)	>0.1
Mutant	4 (2.1%)	1 (0.6%)	0 (0.0%)	5 (1.4%)	
**KRAS**					
Wildtype	187 (98.4%)	157 (99.4%)	13 (92.9%)	357 (98.6%)	>0.1
Mutant	3 (1.6%)	1 (0.6%)	1 (7.1%)	5 (1.4%)	
**Age**					
<=50	35 (18.1%)	36 (21.8%)	7 (46.7%)	78 (20.9%)	<0.05*
>50	158 (81.9%)	129 (78.2%)	8 (53.3%)	295 (79.1%)	
**Gender**					
Female	48 (24.7%)	67 (40.6%)	6 (40.0%)	121 (32.4%)	<0.01*
Male	146 (75.3%)	98 (59.4%)	9 (60.0%)	253 (67.6%)	

* Statistically significant

Cancer-associated fibroblasts infiltration of LUAD

	Low (n=22)	Medium(n=246)	High (n=267)	Total (n=535)	*P* Value
**T stage**					
T1-2	20 (90.9%)	212 (86.2%)	232 (86.9%)	464 (86.7%)	>0.1
T3-4	2 (9.1%)	34 (13.8%)	35 (13.1%)	71 (13.3%)	
**LN metastasis**					
N+	6 (27.3%)	87 (35.5%)	93 (34.8%)	186 (34.8%)	>0.1
N0	16 (72.7%)	158 (64.5%)	174 (65.2%)	348 (65.2%)	
**Pathological stage**					
Stage I-II	16 (76.2%)	192 (78.7%)	209 (79.8%)	417 (79.1%)	>0.1
Stage III-IV	5 (23.8%)	52 (21.3%)	53 (20.2%)	110 (20.9%)	
**TP53**					
Wildtype	15 (71.4%)	123 (51.0%)	144 (54.8%)	282 (53.7%)	>0.1
Mutant	6 (28.6%)	118 (49.0%)	119 (45.2%)	243 (46.3%)	
**BRAF**					
Wildtype	18 (85.7%)	222 (92.1%)	248 (94.3%)	488 (93.0%)	>0.1
Mutant	3 (14.3%)	19 (7.9%)	15 (5.7%)	37 (7.0%)	
**EGFR**					
Wildtype	19 (90.5%)	212 (88.0%)	228 (86.7%)	459 (87.4%)	>0.1
Mutant	2 (9.5%)	29 (12.0%)	35 (13.3%)	66 (12.6%)	
**KRAS**					
Wildtype	16 (76.2%)	185 (76.8%)	199 (75.7%)	400 (76.2%)	>0.1
Mutant	5 (23.8%)	56 (23.2%)	64 (24.3%)	125 (23.8%)	
**Age**					
<=50	4 (18.2%)	24 (10.3%)	13 (5.0%)	41 (7.9%)	<0.05*
>50	18 (81.8%)	210 (89.7%)	247 (95.0%)	475 (92.1%)	
**Gender**					
Female	9 (40.9%)	121 (49.2%)	156 (58.4%)	286 (53.5%)	<0.1
Male	13 (59.1%)	125 (50.8%)	111 (41.6%)	249 (46.5%)	

* Statistically significant

Cancer-associated fibroblasts infiltration of LUSC

	Low (n=27)	Medium(n=267)	High (n=208)	Total (n=502)	*P* Value
**T stage**					
T1-2	24 (88.9%)	219 (82.0%)	165 (79.3%)	408 (81.3%)	>0.1
T3-4	3 (11.1%)	48 (18.0%)	43 (20.7%)	94 (18.7%)	
**LN metastasis**					
N+	9 (33.3%)	98 (36.7%)	75 (36.1%)	182 (36.3%)	>0.1
N0	18 (66.7%)	169 (63.3%)	133 (63.9%)	320 (63.7%)	
**Pathological stage**					
Stage I-II	23 (85.2%)	215 (81.1%)	169 (82.0%)	407 (81.7%)	>0.1
Stage III-IV	4 (14.8%)	50 (18.9%)	37 (18.0%)	91 (18.3%)	
**TP53**					
Wildtype	6 (22.2%)	54 (20.5%)	53 (26.6%)	113 (23.1%)	>0.1
Mutant	21 (77.8%)	209 (79.5%)	146 (73.4%)	376 (76.9%)	
**BRAF**					
Wildtype	27 (100.0%)	253 (96.2%)	198 (99.5%)	478 (97.8%)	<0.05*
Mutant	0 (0.0%)	10 (3.8%)	1 (0.5%)	11 (2.2%)	
**EGFR**					
Wildtype	27 (100.0%)	260 (98.9%)	195 (98.0%)	482 (98.6%)	>0.1
Mutant	0 (0.0%)	3 (1.1%)	4 (2.0%)	7 (1.4%)	
**KRAS**					
Wildtype	26 (96.3%)	261 (99.2%)	195 (98.0%)	482 (98.6%)	>0.1
Mutant	1 (3.7%)	2 (0.8%)	4 (2.0%)	7 (1.4%)	
**Age**					
>50	27 (100.0%)	254 (96.9%)	192 (94.1%)	473 (95.9%)	>0.1
<=50	0 (0.0%)	8 (3.1%)	12 (5.9%)	20 (4.1%)	
**Gender**					
Female	5 (18.5%)	74 (27.7%)	52 (25.0%)	131 (26.1%)	>0.1
Male	22 (81.5%)	193 (72.3%)	156 (75.0%)	371 (73.9%)	

* Statistically significant

Cancer-associated fibroblasts infiltration of MESO

	Medium(n=36)	High (n=50)	Total (n=86)	*P* Value
**T stage**				
T1-2	17 (47.2%)	23 (46.0%)	40 (46.5%)	>0.1
T3-4	19 (52.8%)	27 (54.0%)	46 (53.5%)	
**LN metastasis**				
N+	15 (41.7%)	28 (56.0%)	43 (50.0%)	>0.1
N0	21 (58.3%)	22 (44.0%)	43 (50.0%)	
**Pathological stage**				
Stage I-II	11 (30.6%)	15 (30.0%)	26 (30.2%)	>0.1
Stage III-IV	25 (69.4%)	35 (70.0%)	60 (69.8%)	
**TP53**				
Wildtype	29 (85.3%)	38 (84.4%)	67 (84.8%)	>0.1
Mutant	5 (14.7%)	7 (15.6%)	12 (15.2%)	
**KRAS**				
Wildtype	34 (100.0%)	44 (97.8%)	78 (98.7%)	>0.1
Mutant	0 (0.0%)	1 (2.2%)	1 (1.3%)	
**Gender**				
Female	7 (19.4%)	8 (16.0%)	15 (17.4%)	>0.1
Male	29 (80.6%)	42 (84.0%)	71 (82.6%)	

* Statistically significant

Cancer-associated fibroblasts infiltration of OV

	Low (n=46)	Medium(n=205)	High (n=128)	Total (n=379)	*P* Value
**TP53**					
Wildtype	4 (12.1%)	8 (5.2%)	10 (11.2%)	22 (8.0%)	>0.1
Mutant	29 (87.9%)	146 (94.8%)	79 (88.8%)	254 (92.0%)	
**BRAF**					
Wildtype	33 (100.0%)	152 (98.7%)	89 (100.0%)	274 (99.3%)	>0.1
Mutant	0 (0.0%)	2 (1.3%)	0 (0.0%)	2 (0.7%)	
**EGFR**					
Wildtype	33 (100.0%)	154 (100.0%)	85 (95.5%)	272 (98.6%)	<0.05*
Mutant	0 (0.0%)	0 (0.0%)	4 (4.5%)	4 (1.4%)	
**KRAS**					
Wildtype	32 (97.0%)	153 (99.4%)	87 (97.8%)	272 (98.6%)	>0.1
Mutant	1 (3.0%)	1 (0.6%)	2 (2.2%)	4 (1.4%)	
**Age**					
<=50	8 (17.4%)	48 (23.5%)	35 (27.3%)	91 (24.1%)	>0.1
>50	38 (82.6%)	156 (76.5%)	93 (72.7%)	287 (75.9%)	

* Statistically significant

Cancer-associated fibroblasts infiltration of PAAD

	Low (n=6)	Medium(n=15)	High (n=157)	Total (n=178)	*P* Value
**T stage**					
T1-2	2 (33.3%)	2 (13.3%)	27 (17.3%)	31 (17.5%)	>0.1
T3-4	4 (66.7%)	13 (86.7%)	129 (82.7%)	146 (82.5%)	
**LN metastasis**					
N+	4 (66.7%)	10 (66.7%)	113 (72.4%)	127 (71.8%)	>0.1
N0	2 (33.3%)	5 (33.3%)	43 (27.6%)	50 (28.2%)	
**TP53**					
Wildtype	5 (100.0%)	9 (64.3%)	56 (42.1%)	70 (46.1%)	<0.01*
Mutant	0 (0.0%)	5 (35.7%)	77 (57.9%)	82 (53.9%)	
**BRAF**					
Wildtype	5 (100.0%)	13 (92.9%)	133 (100.0%)	151 (99.3%)	>0.1
Mutant	0 (0.0%)	1 (7.1%)	0 (0.0%)	1 (0.7%)	
**EGFR**					
Wildtype	5 (100.0%)	14 (100.0%)	132 (99.2%)	151 (99.3%)	>0.1
Mutant	0 (0.0%)	0 (0.0%)	1 (0.8%)	1 (0.7%)	
**KRAS**					
Wildtype	4 (80.0%)	9 (64.3%)	58 (43.6%)	71 (46.7%)	>0.1
Mutant	1 (20.0%)	5 (35.7%)	75 (56.4%)	81 (53.3%)	
**Age**					
>50	6 (100.0%)	15 (100.0%)	134 (85.4%)	155 (87.1%)	>0.1
<=50	0 (0.0%)	0 (0.0%)	23 (14.6%)	23 (12.9%)	
**Gender**					
Male	6 (100.0%)	9 (60.0%)	83 (52.9%)	98 (55.1%)	<0.1
Female	0 (0.0%)	6 (40.0%)	74 (47.1%)	80 (44.9%)	

* Statistically significant

Cancer-associated fibroblasts infiltration of PCPG

	Low (n=23)	Medium(n=155)	High (n=5)	Total (n=183)	*P* Value
**BRAF**					
Wildtype	22 (100.0%)	154 (99.4%)	5 (100.0%)	181 (99.5%)	>0.1
Mutant	0 (0.0%)	1 (0.6%)	0 (0.0%)	1 (0.5%)	
**Gender**					
Female	16 (69.6%)	84 (54.2%)	2 (40.0%)	102 (55.7%)	>0.1
Male	7 (30.4%)	71 (45.8%)	3 (60.0%)	81 (44.3%)	

* Statistically significant

Cancer-associated fibroblasts infiltration of PRAD

	Low (n=156)	Medium(n=332)	High (n=11)	Total (n=499)	*P* Value
**T stage**					
T1-2	125 (80.6%)	252 (76.4%)	6 (54.5%)	383 (77.2%)	>0.1
T3-4	30 (19.4%)	78 (23.6%)	5 (45.5%)	113 (22.8%)	
**LN metastasis**					
N+	27 (20.3%)	51 (18.0%)	1 (11.1%)	79 (18.5%)	>0.1
N0	106 (79.7%)	233 (82.0%)	8 (88.9%)	347 (81.5%)	
**TP53**					
Wildtype	141 (90.4%)	285 (89.3%)	8 (72.7%)	434 (89.3%)	>0.1
Mutant	15 (9.6%)	34 (10.7%)	3 (27.3%)	52 (10.7%)	
**BRAF**					
Wildtype	154 (98.7%)	315 (98.7%)	11 (100.0%)	480 (98.8%)	>0.1
Mutant	2 (1.3%)	4 (1.3%)	0 (0.0%)	6 (1.2%)	
**EGFR**					
Wildtype	155 (99.4%)	317 (99.4%)	11 (100.0%)	483 (99.4%)	>0.1
Mutant	1 (0.6%)	2 (0.6%)	0 (0.0%)	3 (0.6%)	
**KRAS**					
Wildtype	155 (99.4%)	318 (99.7%)	11 (100.0%)	484 (99.6%)	>0.1
Mutant	1 (0.6%)	1 (0.3%)	0 (0.0%)	2 (0.4%)	
**Age**					
<=50	12 (7.7%)	22 (6.6%)	1 (9.1%)	35 (7.0%)	>0.1
>50	144 (92.3%)	310 (93.4%)	10 (90.9%)	464 (93.0%)	

* Statistically significant

Cancer-associated fibroblasts infiltration of READ

	Low (n=16)	Medium(n=95)	High (n=56)	Total (n=167)	*P* Value
**T stage**					
T1-2	6 (37.5%)	25 (26.6%)	6 (11.1%)	37 (22.6%)	<0.05*
T3-4	10 (62.5%)	69 (73.4%)	48 (88.9%)	127 (77.4%)	
**LN metastasis**					
N+	10 (62.5%)	42 (44.7%)	28 (51.9%)	80 (48.8%)	>0.1
N0	6 (37.5%)	52 (55.3%)	26 (48.1%)	84 (51.2%)	
**Pathological stage**					
Stage I-II	5 (35.7%)	51 (54.8%)	25 (51.0%)	81 (51.9%)	>0.1
Stage III-IV	9 (64.3%)	42 (45.2%)	24 (49.0%)	75 (48.1%)	
**TP53**					
Wildtype	6 (42.9%)	15 (21.1%)	10 (21.3%)	31 (23.5%)	>0.1
Mutant	8 (57.1%)	56 (78.9%)	37 (78.7%)	101 (76.5%)	
**BRAF**					
Wildtype	14 (100.0%)	68 (95.8%)	45 (95.7%)	127 (96.2%)	>0.1
Mutant	0 (0.0%)	3 (4.2%)	2 (4.3%)	5 (3.8%)	
**EGFR**					
Wildtype	14 (100.0%)	69 (97.2%)	47 (100.0%)	130 (98.5%)	>0.1
Mutant	0 (0.0%)	2 (2.8%)	0 (0.0%)	2 (1.5%)	
**KRAS**					
Wildtype	6 (42.9%)	39 (54.9%)	30 (63.8%)	75 (56.8%)	>0.1
Mutant	8 (57.1%)	32 (45.1%)	17 (36.2%)	57 (43.2%)	
**Age**					
<=50	2 (12.5%)	14 (14.7%)	6 (10.9%)	22 (13.3%)	>0.1
>50	14 (87.5%)	81 (85.3%)	49 (89.1%)	144 (86.7%)	
**Gender**					
Female	9 (56.2%)	41 (43.2%)	25 (45.5%)	75 (45.2%)	>0.1
Male	7 (43.8%)	54 (56.8%)	30 (54.5%)	91 (54.8%)	

* Statistically significant

Cancer-associated fibroblasts infiltration of SARC

	Medium(n=89)	High (n=174)	Total (n=263)	*P* Value
**TP53**				
Wildtype	43 (53.8%)	110 (69.2%)	153 (64.0%)	<0.05*
Mutant	37 (46.2%)	49 (30.8%)	86 (36.0%)	
**BRAF**				
Wildtype	80 (100.0%)	158 (99.4%)	238 (99.6%)	>0.1
Mutant	0 (0.0%)	1 (0.6%)	1 (0.4%)	
**EGFR**				
Wildtype	80 (100.0%)	157 (98.7%)	237 (99.2%)	>0.1
Mutant	0 (0.0%)	2 (1.3%)	2 (0.8%)	
**KRAS**				
Wildtype	80 (100.0%)	158 (99.4%)	238 (99.6%)	>0.1
Mutant	0 (0.0%)	1 (0.6%)	1 (0.4%)	
**Gender**				
Female	56 (62.9%)	88 (50.6%)	144 (54.8%)	<0.1
Male	33 (37.1%)	86 (49.4%)	119 (45.2%)	

* Statistically significant

Cancer-associated fibroblasts infiltration of SKCM

	Low (n=121)	Medium(n=276)	High (n=74)	Total (n=471)	*P* Value
**T stage**					
T1-2	30 (26.5%)	64 (24.7%)	26 (37.7%)	120 (27.2%)	<0.1
T3-4	83 (73.5%)	195 (75.3%)	43 (62.3%)	321 (72.8%)	
**LN metastasis**					
N+	54 (47.0%)	131 (49.4%)	29 (42.0%)	214 (47.7%)	>0.1
N0	61 (53.0%)	134 (50.6%)	40 (58.0%)	235 (52.3%)	
**Pathological stage**					
Stage I-II	64 (57.7%)	133 (53.6%)	34 (51.5%)	231 (54.4%)	>0.1
Stage III-IV	47 (42.3%)	115 (46.4%)	32 (48.5%)	194 (45.6%)	
**TP53**					
Wildtype	96 (80.0%)	241 (88.0%)	66 (90.4%)	403 (86.3%)	<0.1
Mutant	24 (20.0%)	33 (12.0%)	7 (9.6%)	64 (13.7%)	
**BRAF**					
Wildtype	76 (63.3%)	125 (45.6%)	31 (42.5%)	232 (49.7%)	<0.01*
Mutant	44 (36.7%)	149 (54.4%)	42 (57.5%)	235 (50.3%)	
**EGFR**					
Wildtype	113 (94.2%)	256 (93.4%)	70 (95.9%)	439 (94.0%)	>0.1
Mutant	7 (5.8%)	18 (6.6%)	3 (4.1%)	28 (6.0%)	
**KRAS**					
Wildtype	114 (95.0%)	271 (98.9%)	72 (98.6%)	457 (97.9%)	<0.05*
Mutant	6 (5.0%)	3 (1.1%)	1 (1.4%)	10 (2.1%)	
**Age**					
<=50	31 (26.1%)	82 (30.3%)	28 (38.9%)	141 (30.5%)	>0.1
>50	88 (73.9%)	189 (69.7%)	44 (61.1%)	321 (69.5%)	
**Gender**					
Female	44 (36.4%)	115 (41.8%)	20 (27.0%)	179 (38.1%)	<0.1
Male	77 (63.6%)	160 (58.2%)	54 (73.0%)	291 (61.9%)	

* Statistically significant

Cancer-associated fibroblasts infiltration of STAD

	Low (n=12)	Medium(n=128)	High (n=235)	Total (n=375)	*P* Value
**T stage**					
T1-2	9 (75.0%)	44 (34.4%)	46 (19.6%)	99 (26.4%)	<0.01*
T3-4	3 (25.0%)	84 (65.6%)	189 (80.4%)	276 (73.6%)	
**LN metastasis**					
N+	5 (41.7%)	92 (72.4%)	165 (70.5%)	262 (70.2%)	<0.1
N0	7 (58.3%)	35 (27.6%)	69 (29.5%)	111 (29.8%)	
**Pathological stage**					
Stage I-II	7 (63.6%)	62 (50.8%)	95 (43.4%)	164 (46.6%)	>0.1
Stage III-IV	4 (36.4%)	60 (49.2%)	124 (56.6%)	188 (53.4%)	
**TP53**					
Wildtype	7 (63.6%)	61 (49.2%)	138 (59.2%)	206 (56.0%)	>0.1
Mutant	4 (36.4%)	63 (50.8%)	95 (40.8%)	162 (44.0%)	
**BRAF**					
Wildtype	10 (90.9%)	120 (96.8%)	223 (95.7%)	353 (95.9%)	>0.1
Mutant	1 (9.1%)	4 (3.2%)	10 (4.3%)	15 (4.1%)	
**EGFR**					
Wildtype	11 (100.0%)	117 (94.4%)	225 (96.6%)	353 (95.9%)	>0.1
Mutant	0 (0.0%)	7 (5.6%)	8 (3.4%)	15 (4.1%)	
**KRAS**					
Wildtype	10 (90.9%)	114 (91.9%)	218 (93.6%)	342 (92.9%)	>0.1
Mutant	1 (9.1%)	10 (8.1%)	15 (6.4%)	26 (7.1%)	
**Age**					
<=50	1 (8.3%)	9 (7.0%)	19 (8.2%)	29 (7.8%)	>0.1
>50	11 (91.7%)	119 (93.0%)	212 (91.8%)	342 (92.2%)	
**Gender**					
Female	3 (25.0%)	53 (41.4%)	78 (33.2%)	134 (35.7%)	>0.1
Male	9 (75.0%)	75 (58.6%)	157 (66.8%)	241 (64.3%)	

* Statistically significant

Cancer-associated fibroblasts infiltration of TGCT

	Medium(n=119)	High (n=37)	Total (n=156)	*P* Value
**T stage**				
T1-2	111 (98.2%)	23 (88.5%)	134 (96.4%)	<0.1
T3-4	2 (1.8%)	3 (11.5%)	5 (3.6%)	
**LN metastasis**				
N+	44 (38.9%)	10 (38.5%)	54 (38.8%)	>0.1
N0	69 (61.1%)	16 (61.5%)	85 (61.2%)	
**Pathological stage**				
Stage I-II	60 (85.7%)	8 (66.7%)	68 (82.9%)	>0.1
Stage III-IV	10 (14.3%)	4 (33.3%)	14 (17.1%)	
**TP53**				
Wildtype	108 (99.1%)	25 (100.0%)	133 (99.3%)	>0.1
Mutant	1 (0.9%)	0 (0.0%)	1 (0.7%)	
**EGFR**				
Wildtype	109 (100.0%)	24 (96.0%)	133 (99.3%)	>0.1
Mutant	0 (0.0%)	1 (4.0%)	1 (0.7%)	
**KRAS**				
Wildtype	99 (90.8%)	25 (100.0%)	124 (92.5%)	>0.1
Mutant	10 (9.2%)	0 (0.0%)	10 (7.5%)	

* Statistically significant

Cancer-associated fibroblasts infiltration of THCA

	Low (n=62)	Medium(n=400)	High (n=48)	Total (n=510)	*P* Value
**T stage**					
T1-2	32 (51.6%)	257 (64.2%)	21 (43.8%)	310 (60.8%)	<0.01*
T3-4	30 (48.4%)	143 (35.8%)	27 (56.2%)	200 (39.2%)	
**LN metastasis**					
N+	40 (64.5%)	205 (51.2%)	36 (75.0%)	281 (55.1%)	<0.01*
N0	22 (35.5%)	195 (48.8%)	12 (25.0%)	229 (44.9%)	
**Pathological stage**					
Stage I-II	37 (60.7%)	279 (69.9%)	22 (45.8%)	338 (66.5%)	<0.01*
Stage III-IV	24 (39.3%)	120 (30.1%)	26 (54.2%)	170 (33.5%)	
**TP53**					
Wildtype	61 (100.0%)	381 (99.5%)	46 (100.0%)	488 (99.6%)	>0.1
Mutant	0 (0.0%)	2 (0.5%)	0 (0.0%)	2 (0.4%)	
**BRAF**					
Wildtype	22 (36.1%)	166 (43.3%)	9 (19.6%)	197 (40.2%)	<0.01*
Mutant	39 (63.9%)	217 (56.7%)	37 (80.4%)	293 (59.8%)	
**KRAS**					
Wildtype	60 (98.4%)	380 (99.2%)	46 (100.0%)	486 (99.2%)	>0.1
Mutant	1 (1.6%)	3 (0.8%)	0 (0.0%)	4 (0.8%)	
**Age**					
<=50	34 (54.8%)	232 (58.0%)	26 (54.2%)	292 (57.3%)	>0.1
>50	28 (45.2%)	168 (42.0%)	22 (45.8%)	218 (42.7%)	
**Gender**					
Female	44 (71.0%)	292 (73.0%)	35 (72.9%)	371 (72.7%)	>0.1
Male	18 (29.0%)	108 (27.0%)	13 (27.1%)	139 (27.3%)	

* Statistically significant

Cancer-associated fibroblasts infiltration of THYM

	Low (n=28)	Medium(n=88)	High (n=3)	Total (n=119)	*P* Value
**TP53**					
Wildtype	26 (96.3%)	84 (96.6%)	3 (100.0%)	113 (96.6%)	>0.1
Mutant	1 (3.7%)	3 (3.4%)	0 (0.0%)	4 (3.4%)	
**BRAF**					
Wildtype	26 (96.3%)	87 (100.0%)	3 (100.0%)	116 (99.1%)	>0.1
Mutant	1 (3.7%)	0 (0.0%)	0 (0.0%)	1 (0.9%)	
**KRAS**					
Wildtype	27 (100.0%)	86 (98.9%)	3 (100.0%)	116 (99.1%)	>0.1
Mutant	0 (0.0%)	1 (1.1%)	0 (0.0%)	1 (0.9%)	
**Gender**					
Female	14 (50.0%)	43 (48.9%)	0 (0.0%)	57 (47.9%)	>0.1
Male	14 (50.0%)	45 (51.1%)	3 (100.0%)	62 (52.1%)	

* Statistically significant

Cancer-associated fibroblasts infiltration of UCEC

	Low (n=97)	Medium(n=370)	High (n=85)	Total (n=552)	*P* Value
**TP53**					
Wildtype	56 (61.5%)	221 (61.0%)	55 (67.9%)	332 (62.2%)	>0.1
Mutant	35 (38.5%)	141 (39.0%)	26 (32.1%)	202 (37.8%)	
**BRAF**					
Wildtype	89 (97.8%)	341 (94.2%)	74 (91.4%)	504 (94.4%)	>0.1
Mutant	2 (2.2%)	21 (5.8%)	7 (8.6%)	30 (5.6%)	
**EGFR**					
Wildtype	86 (94.5%)	339 (93.6%)	77 (95.1%)	502 (94.0%)	>0.1
Mutant	5 (5.5%)	23 (6.4%)	4 (4.9%)	32 (6.0%)	
**KRAS**					
Wildtype	75 (82.4%)	298 (82.3%)	65 (80.2%)	438 (82.0%)	>0.1
Mutant	16 (17.6%)	64 (17.7%)	16 (19.8%)	96 (18.0%)	
**Age**					
<=50	1 (1.0%)	31 (8.4%)	17 (20.2%)	49 (8.9%)	<0.01*
>50	95 (99.0%)	338 (91.6%)	67 (79.8%)	500 (91.1%)	

* Statistically significant

Cancer-associated fibroblasts infiltration of UCS

	Low (n=4)	Medium(n=21)	High (n=31)	Total (n=56)	*P* Value
**TP53**					
Wildtype	0 (0.0%)	4 (19.0%)	1 (3.2%)	5 (8.9%)	>0.1
Mutant	4 (100.0%)	17 (81.0%)	30 (96.8%)	51 (91.1%)	
**BRAF**					
Mutant	0 (0.0%)	1 (4.8%)	0 (0.0%)	1 (1.8%)	>0.1
Wildtype	4 (100.0%)	20 (95.2%)	31 (100.0%)	55 (98.2%)	
**KRAS**					
Wildtype	3 (75.0%)	16 (76.2%)	30 (96.8%)	49 (87.5%)	<0.05*
Mutant	1 (25.0%)	5 (23.8%)	1 (3.2%)	7 (12.5%)	

* Statistically significant

Cancer-associated fibroblasts infiltration of UVM

	Low (n=61)	Medium(n=19)	Total (n=80)	*P* Value
**T stage**				
T1-2	4 (6.6%)	1 (5.3%)	5 (6.2%)	>0.1
T3-4	57 (93.4%)	18 (94.7%)	75 (93.8%)	
**LN metastasis**				
N+	3 (4.9%)	1 (5.3%)	4 (5.0%)	>0.1
N0	58 (95.1%)	18 (94.7%)	76 (95.0%)	
**Pathological stage**				
Stage I-II	32 (53.3%)	7 (36.8%)	39 (49.4%)	>0.1
Stage III-IV	28 (46.7%)	12 (63.2%)	40 (50.6%)	
**Gender**				
Female	26 (42.6%)	9 (47.4%)	35 (43.8%)	>0.1
Male	35 (57.4%)	10 (52.6%)	45 (56.2%)	

* Statistically significant

Pan-cancer cancer-associated fibroblasts infiltration

	Low (n=1223)	Medium (n=3868)	High (n=2713)	Total (n=7804)	*P* Value
**T stage**					
T1-2	809 (66.1%)	2339 (60.5%)	1552 (57.2%)	4700 (60.2%)	<0.01*
T3-4	414 (33.9%)	1529 (39.5%)	1161 (42.8%)	3104 (39.8%)	
**LN metastasis**					
N+	463 (38.6%)	1670 (43.7%)	1422 (52.5%)	3555 (46.0%)	<0.01*
N0	738 (61.4%)	2153 (56.3%)	1288 (47.5%)	4179 (54.0%)	
**Pathological stage**					
Stage I-II	638 (68.2%)	2021 (62.3%)	1577 (61.5%)	4236 (62.8%)	<0.01*
Stage III-IV	298 (31.8%)	1225 (37.7%)	989 (38.5%)	2512 (37.2%)	
**TP53**					
Wildtype	1157 (77.3%)	3363 (65.4%)	1631 (56.0%)	6151 (64.4%)	<0.01*
Mutant	339 (22.7%)	1782 (34.6%)	1284 (44.0%)	3405 (35.6%)	
**BRAF**					
Wildtype	1391 (93.0%)	4664 (90.7%)	2763 (94.8%)	8818 (92.3%)	<0.01*
Mutant	105 (7.0%)	481 (9.3%)	152 (5.2%)	738 (7.7%)	
**Gender**					
Female	677 (42.3%)	2776 (50.0%)	1905 (59.9%)	5358 (51.9%)	<0.01*
Male	924 (57.7%)	2777 (50.0%)	1274 (40.1%)	4975 (48.1%)	
**Cancer type**					
Adenocarcinomas	442 (27.6%)	1934 (34.7%)	2014 (63.1%)	4390 (42.4%)	<0.01*
Kidney	228 (14.2%)	557 (10.0%)	108 (3.4%)	893 (8.6%)	
Ly-Hem	182 (11.4%)	128 (2.3%)	8 (0.3%)	318 (3.1%)	
Misc	376 (23.5%)	821 (14.7%)	266 (8.3%)	1463 (14.1%)	
Neuronal	206 (12.9%)	1128 (20.3%)	98 (3.1%)	1432 (13.8%)	
Sar	4 (0.2%)	110 (2.0%)	205 (6.4%)	319 (3.1%)	
Squamous	163 (10.2%)	772 (13.9%)	457 (14.3%)	1392 (13.4%)	
SC	0 (0.0%)	119 (2.1%)	37 (1.2%)	156 (1.5%)	

* significant
